# Supplementary material for: Catalytic asymmetric [4 + 2] dearomative photocycloadditions of anthracene and its derivatives with alkenylazaarenes
Source: Nat Commun. 2024 May 29;15:4563. doi: 10.1038/s41467-024-48982-y (PMC11137010; doi:10.1038/s41467-024-48982-y)
Supplement: Supplementary file 1 — Supplementary Information [file 41467_2024_48982_MOESM1_ESM.pdf]

# Supplementary Information

## Catalytic Asymmetric [4 + 2] Dearomative Photocycloadditions of Anthracene and Its Derivatives with Alkenylazaarenes

Dong Tian,[1] Wenshuo Shi,[1] Xin Sun,[1,*] Xiaowei Zhao,[1] Yanli Yin,[2,3,*] and Zhiyong Jiang[1,2,*]

[1]*Key Laboratory of Natural Medicine and Immuno-Engineering of Henan Province, Henan University, Jinming Campus, Kaifeng, Henan, P. R. China 475004*

[2]*School of Chemistry and Chemical Engineering, Henan Normal University, Xinxiang, Henan, P. R. China 453007*

[3]*College of Advanced Interdisciplinary Science and Technology, Henan University of Technology, Zhengzhou, Henan, P. R. China 451001*

**E-mail: jiangzhiyong@htu.edu.cn; yinzihust@163.com; sunxin0910@qq.com*

# Table of Contents

1. General information ........ S3

2. Optimization of reaction conditions ........ S5

3. General experimental procedures ........ S6

4. Mechanistic studies ........ S11

5. Synthetic applications ........ S21

6. Determination of the absolute configurations ........ S22

7. Characterization of adducts ........ S56

8. Copies of NMR spectra ........ S117

9. References ........ S186

## 1. General information

### General procedures and methods

Experiments involving moisture and/or air sensitive components were performed under a positive pressure of argon in oven-dried glassware equipped with a rubber septum inlet. Dried solvents and liquid reagents were transferred by oven-dried syringes or hypodermic syringe cooled to ambient temperature in a desiccator. Reactions mixtures were stirred in 10 mL sample vial with Teflon-coated magnetic stirring bars unless otherwise stated. Moisture in non-volatile reagents/compounds was removed in high *vacuo* by means of an oil pump and subsequent purging with nitrogen. Solvents were removed *in vacuo* under ~30 mmHg and heated with a water bath at 30–35 °C using rotary evaporator with aspirator. The condenser was cooled with running water at 0 °C.

All experiments were monitored by analytical thin layer chromatography (TLC). TLC was performed on pre-coated plates, 60 $F_{254}$. After elution, plate was visualized under UV illumination at 254 nm for UV active material. Further visualization was achieved by staining $Ce(SO_4)_2$ and phosphomolybdic acid solution. For those using the aqueous stains, the TLC plates were heated on a hot plate.

Columns for flash chromatography (FC) contained *silica gel* 200–300 mesh. Columns were packed as slurry of *silica gel* in petroleum ether and equilibrated solution using the appropriate solvent system. The elution was assisted by applying pressure of about 2 atm with an air pump.

### Instrumentations

$^1$H NMR and $^{13}$C NMR spectra were recorded on a Bruker 300 MHz NMR spectrometer operating at 300 MHz for proton and 75 MHz for carbon in $CDCl_3$ or $CD_2Cl_2$. $^{19}$F NMR spectra were recorded on a Bruker 400 MHz NMR spectrometer operating at 376 MHz for fluorine in $CDCl_3$ or $CD_2Cl_2$. $^{19}$F NMR spectra were recorded on a Bruker 600 MHz NMR spectrometer operating at 565 MHz for fluorine in $CDCl_3$ or $CD_2Cl_2$.Chemical shifts are reported in parts per million (ppm), using the residual solvent signal as an internal standard: $CDCl_3$ ($^1$H NMR: $\delta$ 7.26, singlet; $^{13}$C NMR: $\delta$ 77.0, triplet); $CD_2Cl_2$ ($^1$H NMR: $\delta$ 5.32, singlet; $^{13}$C NMR: $\delta$ 53.8, quintet). Multiplicities were given as: *s* (singlet), *d* (doublet), *t* (triplet), *q* (quartet), *quintet*, *m* (multiplets), *dd* (doublet of doublets), *dt* (doublet of triplets), and *br* (broad). Coupling

constants ($J$) were recorded in hertz (Hz). The number of proton atoms ($n$) for a given resonance was indicated by $n$H. The number of carbon atoms ($n$) for a given resonance was indicated by $n$C. HRMS (Analyzer: TOF) was reported in units of mass of charge ratio (m/z). Mass samples were dissolved in $CH_3CN$ (HPLC Grade) unless otherwise stated. Optical rotations were recorded on a polarimeter with a sodium lamp of wavelength 589 nm and reported as follows; $[\alpha]_{\lambda}^{T°C}$ ($c$ = g/100 mL, solvent). Melting points were determined on a melting point apparatus. Enantiomeric excesses were determined by chiral High Performance Liquid Chromatography (HPLC) analysis. UV detection was monitored at 254 nm and 210 nm at the same time. HPLC samples were dissolved in HPLC grade isopropanol (IPA) unless otherwise stated.

**Materials**

All commercial reagents were purchased with the highest purity grade. They were used without further purification unless specified. All solvents used, mainly petroleum ether (PE) and ethyl acetate (EtOAc) were distilled. Anhydrous dichloromethane (DCM), $CH_3CN$, $CHCl_3$ were freshly distilled from $CaH_2$ and stored under $N_2$ atmosphere. THF and toluene were freshly distilled from sodium/benzophenone before use. All compounds synthesized were stored in a −20 °C freezer and light-sensitive compounds were protected with aluminium foil.

## 2. Optimization of reaction conditions

**Supplementary Table 1. Optimization of Reaction Conditions of Anthracene 1a with 2a**[a]

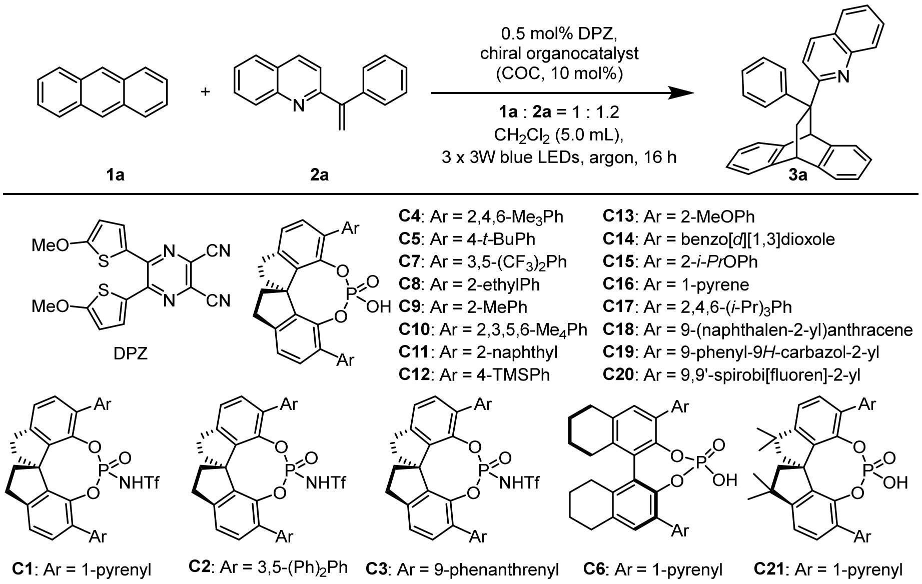


| entry | COC | $T$ (°C) | ee (%)[b] |
|---|---|---|---|
| 1[c] | (PhO)2POOH | 25 | N.A. |
| 2 | **C1** | 25 | 68 |
| 3 | **C1** | –35 | 90[d] |
| 4 | **C2** | –35 | 75 |
| 5 | **C3** | –35 | 58 |
| 6 | **C4** | –35 | N.D. |
| 7 | **C5** | –35 | N.D. |
| 8 | **C6** | –35 | 17 |
| 9 | **C7** | –35 | 0 |
| 10 | **C8** | –35 | 51 |
| 11 | **C9** | –35 | 13 |
| 12 | **C10** | –35 | 60 |
| 13 | **C11** | –35 | 68 |
| 14 | **C12** | –35 | N.D. |
| 15 | **C13** | –35 | 5 |
| 16 | **C14** | –35 | 43 |
| 17 | **C15** | –35 | 27 |
| 18 | **C16** | –35 | 72 |
| 19 | **C17** | –35 | 0 |
| 20 | **C18** | –35 | N.D. |
| 21 | **C19** | –35 | 33 |
| 22 | **C20** | –35 | 45 |

[a]0.01 mmol scale in 0.5 mL solvent. Irradiation distance = 3 cm. [b]Determined by HPLC analysis on a chiral stationary phase. [c]0.1 mmol scale, 36 h, 39% yield. [d]0.1 mmol scale, with irradiation by 3 x 3W blue LEDs, after 36 h, adduct **3a** was obtained in 83% yield with 90% ee. N.A.= not available. N.D. = not determined (for poor conversion).

## 3. General experimental procedures

### (1) General procedure for addition of Anthracene and Its Derivatives with Vinylazaarenes

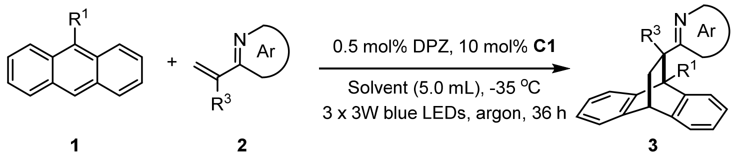


35.0 $\mu$L (0.001 mmol, 0.005 equiv.) of DPZ solution (1.0 mg of DPZ in 200 $\mu$L of toluene) was added into a 25 mL Schlenk tube, and then solvent was removed in *vacuo*.

**For 3a, 3l:** **1** (0.10 mmol, 1.0 equiv.), **2** (0.12 mmol, 1.2 equiv.), **C1** (0.01 mmol, 0.1 equiv.), $CH_2Cl_2$ (5.0 mL) were sequentially added, degassed three times by freeze-pump-thaw method. The reaction mixture was stirred under an argon atmosphere at −35 °C.

**For 3b, 3c, 3e, 3o, 3p:** **1** (0.10 mmol, 1.0 equiv.), **2** (0.12 mmol, 1.2 equiv.), **C1** (0.01 mmol, 0.1 equiv.), toluene (5.0 mL) were sequentially added, degassed three times by freeze-pump-thaw method. The reaction mixture was stirred under an argon atmosphere at −35 °C.

**For 3d, 3r:** **1** (0.10 mmol, 1.0 equiv.), **2** (0.12 mmol, 1.2 equiv.), **C1** (0.01 mmol, 0.1 equiv.), $CHCl_3$ (5.0 mL) were sequentially added, degassed three times by freeze-pump-thaw method. The reaction mixture was stirred under an argon atmosphere at −35 °C.

**For 3f:** **1** (0.10 mmol, 1.0 equiv.), **2** (0.12 mmol, 1.2 equiv.), **C1** (0.01 mmol, 0.1 equiv.), *m*-xylene:$CH_2Cl_2$ = 4:1 (5.0 mL) were sequentially added, degassed three times by freeze-pump-thaw method. The reaction mixture was stirred under an argon atmosphere at −35 °C.

**For 3g:** **1** (0.10 mmol, 1.0 equiv.), **2** (0.12 mmol, 1.2 equiv.), **C1** (0.01 mmol, 0.1 equiv.), *m*-xylene (5.0 mL) were sequentially added, degassed three times by freeze-pump-thaw method. The reaction mixture was stirred under an argon atmosphere at −35 °C.

**For 3h, 3i, 3j, 3k, 3q:** **1** (0.10 mmol, 1.0 equiv.), **2** (0.12 mmol, 1.2 equiv.), **C1** (0.01 mmol, 0.1 equiv.), Mesitylene (5.0 mL) were sequentially added, degassed three times by freeze-pump-thaw method. The reaction mixture was stirred under an argon atmosphere at −35 °C.

**For 3g, 3w:** **1** (0.10 mmol, 1.0 equiv.), **2** (0.12 mmol, 1.2 equiv.), **C16** (0.01 mmol, 0.1 equiv.), $CH_2Cl_2$ (5.0 mL) were sequentially added, degassed three times by freeze-pump-thaw method. The reaction mixture was stirred under an argon atmosphere at −35 °C.

**For 3n:** **1** (0.10 mmol, 1.0 equiv.), **2** (0.12 mmol, 1.2 equiv.), **C21** (0.01 mmol, 0.1 equiv.),

$CH_2Cl_2$ (5.0 mL) were sequentially added, degassed three times by freeze-pump-thaw method. The reaction mixture was stirred under an argon atmosphere at −35 ºC.

**For 3i:** **1** (0.10 mmol, 1.0 equiv.), **2** (0.12 mmol, 1.2 equiv.), **C1** (0.01 mmol, 0.1 equiv.), $CHCl_3$ (5.0 mL) were sequentially added, degassed three times by freeze-pump-thaw method. The reaction mixture was stirred under an argon atmosphere at −55 ºC.

**For 3t:** **1** (0.10 mmol, 1.0 equiv.), **2** (0.12 mmol, 1.2 equiv.), **C2** (0.01 mmol, 0.1 equiv.), $CH_2Cl_2$ (5.0 mL) were sequentially added, degassed three times by freeze-pump-thaw method. The reaction mixture was stirred under an argon atmosphere at −55 ºC.

**For 3v:** **1** (0.10 mmol, 1.0 equiv.), **2** (0.12 mmol, 1.2 equiv.), **C18** (0.01 mmol, 0.1 equiv.), $CH_2Cl_2$ (5.0 mL) were sequentially added, degassed three times by freeze-pump-thaw method. The reaction mixture was stirred under an argon atmosphere at −35 ºC, 48 h.

**For 3m, 3x:** **1** (0.10 mmol, 1.0 equiv.), **2** (0.12 mmol, 1.2 equiv.), **C18** (0.01 mmol, 0.1 equiv.), $CH_2Cl_2$ (5.0 mL) were sequentially added, degassed three times by freeze-pump-thaw method. The reaction mixture was stirred under an argon atmosphere at −35 ºC.

**For 3y, 3za:** **1** (0.10 mmol, 1.0 equiv.), **2** (0.12 mmol, 1.2 equiv.), **C16** (0.01 mmol, 0.1 equiv.), $CHCl_3$ (5.0 mL) were sequentially added, degassed three times by freeze-pump-thaw method. The reaction mixture was stirred under an argon atmosphere at −35 ºC.

**For 3zc:** **1** (0.10 mmol, 1.0 equiv.), **2** (0.12 mmol, 1.2 equiv.), **C3** (0.01 mmol, 0.1 equiv.), $CHCl_3$ (5.0 mL) were sequentially added, degassed three times by freeze-pump-thaw method. The reaction mixture was stirred under an argon atmosphere at −35 ºC.

**For 3zd, 3zf, 3zg:** **1** (0.10 mmol, 1.0 equiv.), **2** (0.12 mmol, 1.2 equiv.), **C16** (0.01 mmol, 0.1 equiv.), $CH_2Cl_2$ (5.0 mL) were sequentially added, degassed three times by freeze-pump-thaw method. The reaction mixture was stirred under an argon atmosphere at −35 ºC, 48 h.

**For 3ze:** **1** (0.10 mmol, 1.0 equiv.), **2** (0.12 mmol, 1.2 equiv.), **C16** (0.01 mmol, 0.1 equiv.), $CH_2Cl_2$ (5.0 mL) were sequentially added, degassed three times by freeze-pump-thaw method. The reaction mixture was stirred under an argon atmosphere at −35 ºC, 60 h.

The reaction mixture was stirred under an argon atmosphere at -35 ºC (the temperature was maintained in an incubator) and irradiated by 3 x 3 W blue LEDs ($\lambda_{max}^{Em}$ = 450−455 nm) from a 3.0 cm distance for 36 h. After completion of the reaction, the reaction mixture was removing

the solvent and purification by a short *silica gel* column, followed by gradient elution with petroleum ether/ethyl acetate (30/1–8/1 ratio). Removing the solvent *in vacuo*, afforded products **3**.

**(2) General procedure for the synthesis of 5**

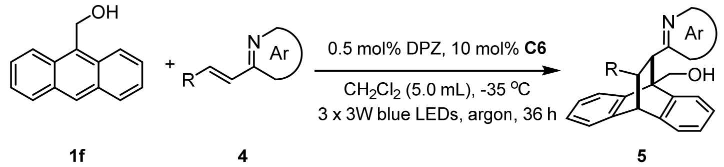


35.0 $\mu$L (0.001 mmol, 0.005 equiv.) of DPZ solution (1.0 mg of DPZ in 200 $\mu$L of toluene) was added into a 25 mL Schlenk tube, and then solvent was removed in *vacuo*.

**For 51:** **1f** (0.10 mmol, 1.0 equiv.), **4** (0.12 mmol, 1.2 equiv.), **C6** (0.01 mmol, 0.1 equiv.) and $CH_2Cl_2$ (5.0 mL) were sequentially added, degassed three times by freeze-pump-thaw method. The reaction mixture was stirred under an argon atmosphere at −35 °C.

**For 52:** **1f** (0.10 mmol, 1.0 equiv.), **4** (0.12 mmol, 1.2 equiv.), **C16** (0.01 mmol, 0.1 equiv.) and $CH_2Cl_2$ (5.0 mL) were sequentially added, degassed three times by freeze-pump-thaw method. The reaction mixture was stirred under an argon atmosphere at −35 °C.

The reaction mixture was stirred under an argon atmosphere at −35 °C (the temperature was maintained in an incubator) and irradiated by 3 x 3 W blue LED (λ = 450−455 nm) from a 3.0 cm distance for 36 h. After completion of the reaction, the reaction mixture was removing the solvent and purification by a short *silica gel* column, followed by gradient elution with petroleum ether/ethyl acetate (30/1–8/1 ratio). Removing the solvent *in vacuo*, afforded products **5**.

**(3) General procedure for the synthesis of 7**

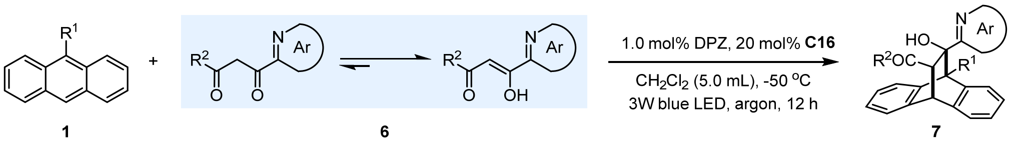


70.0 $\mu$L (0.001 mmol, 0.01 equiv.) of DPZ solution (1.0 mg of DPZ in 200 $\mu$L of toluene) was added into a 25 mL Schlenk tube, and then solvent was removed in *vacuo*.

**For 7a-7e, 7g, 7i, 7k:** **1** (0.11 mmol, 1.1 equiv.), **6** (0.10 mmol, 1.0 equiv.), **C16** (0.02 mmol, 0.2 equiv.) and $CH_2Cl_2$ (5.0 mL) were sequentially added, degassed three times by freeze-pump-

thaw method. The reaction mixture was stirred under an argon atmosphere at −50 °C.

**For 7f:** **1** (0.11 mmol, 1.1 equiv.), **6** (0.10 mmol, 1.0 equiv.), **C16** (0.02 mmol, 0.2 equiv.) and $CH_2Cl_2$ (5.0 mL) were sequentially added, degassed three times by freeze-pump-thaw method. The reaction mixture was stirred under an argon atmosphere at −50 °C, 48 h.

**For 7h:** **1** (0.11 mmol, 1.1 equiv.), **6** (0.10 mmol, 1.0 equiv.), **C16** (0.02 mmol, 0.2 equiv.) and PhCl (5.0 mL) were sequentially added, degassed three times by freeze-pump-thaw method. The reaction mixture was stirred under an argon atmosphere at −50 °C.

**For 7j:** **1** (0.11 mmol, 1.1 equiv.), **6** (0.10 mmol, 1.0 equiv.), **C16** (0.02 mmol, 0.2 equiv.) and $CH_2Cl_2$ (5.0 mL) were sequentially added, degassed three times by freeze-pump-thaw method. The reaction mixture was stirred under an argon atmosphere at 25 °C.

**For 7l:** **1** (0.11 mmol, 1.1 equiv.), **6** (0.10 mmol, 1.0 equiv.), **C16** (0.02 mmol, 0.2 equiv.) and toluene (5.0 mL) were sequentially added, degassed three times by freeze-pump-thaw method. The reaction mixture was stirred under an argon atmosphere at −50 °C, 48 h.

**(4) Experimental Procedure for the Synthesis of DPZ**

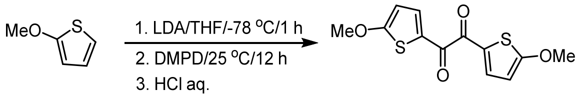


A solution of 2-methoxythiophene (1.14 g; 0.01 mol) in dry THF (30 mL) was treated with lithium diisopropylamide (LDA, 7.5 mL; 0.015 mol; 2.0 M sol. in THF/heptane/ethylbenzene) at −78 °C under argon. The reaction mixture was stirred 1 h at −78 °C and subsequently transferred into a flask containing 1,4-dimethylpiperazine-2,3-dione (DMDP, 0.71 g; 0.005 mol) in dry THF (30 mL). The resulting reaction mixture was stirred for 12 h at 25 °C whereupon aq. HCl (5%; 50 mL) and $CH_2Cl_2$ (100 mL) were added. The organic phase was separated and the water layer was extracted with $CH_2Cl_2$ (2×100 mL). Combined organic extracts were dried ($Na_2SO_4$), filtered and the solvents were evaporated in vacuo. Crude product was purified by column chromatography ($SiO_2$; $CH_2Cl_2$/hexane 1:1 to 1:0) to afford title compound as yellow solid (0.81 g; 57%).

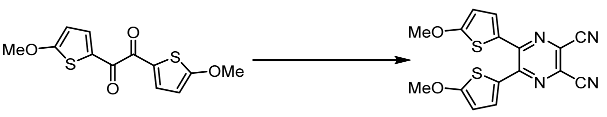


2,2’-thienil (5.0 mmol) and diaminomaleonitrile (DAMN, 1.62 g; 15.0 mmol) were heated in

glacial acetic acid (10 mL) in a sealed pressure tube at 150 °C for 5 h. The cold reaction mixture was diluted with water (100 mL) and extracted with $CH_2Cl_2$ (3×100 mL). The combined organic layers were washed with water (3×300 mL), dried ($Na_2SO_4$) and the solvent was evaporated in vacuo. The resulting crude product was purified by filtration through a plug ($SiO_2$; $CH_2Cl_2$) to afford title compound as orange solid (5.0 mg; 3%).

**(5) Incubator and setup of reactions**

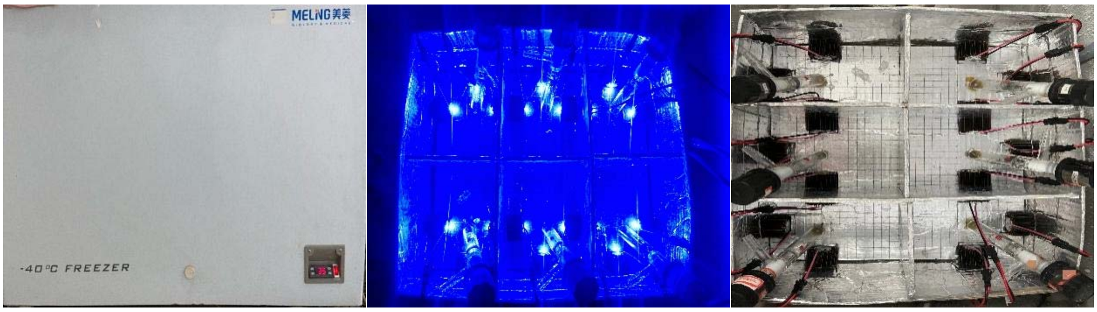

## 4. Mechanistic studies

### UV-vis absorption spectra

UV-vis absorption spectroscopy was performed using a spectrophotometer, equipped with a temperature control unit at 25 °C. The samples were measured in a 1.5 mL quartz cuvettes fitted with a PTFE stopper, **1a** and **2a** were prepared a 10 μM solution and used fresh $CH_2Cl_2$ for measurement.

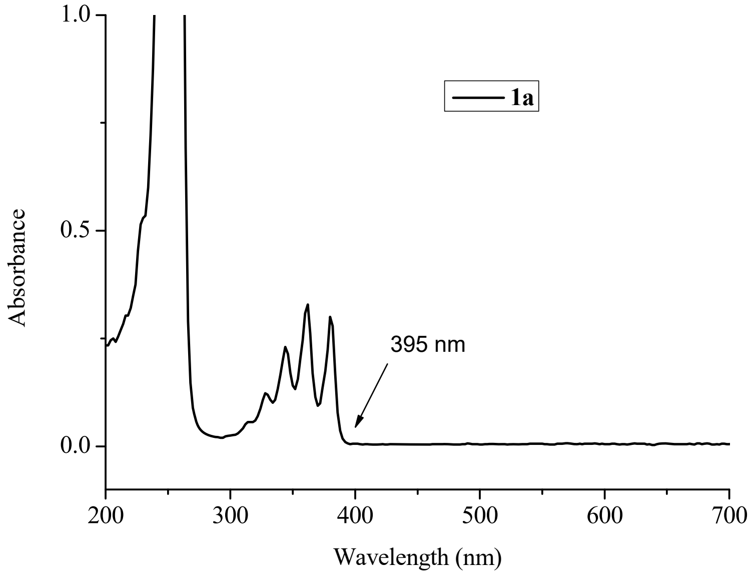


**Supplementary Figure 1.** UV-Vis absorption spectra of anthracene **1a** in $CH_2Cl_2$ (*Conc.* = 10 μM).

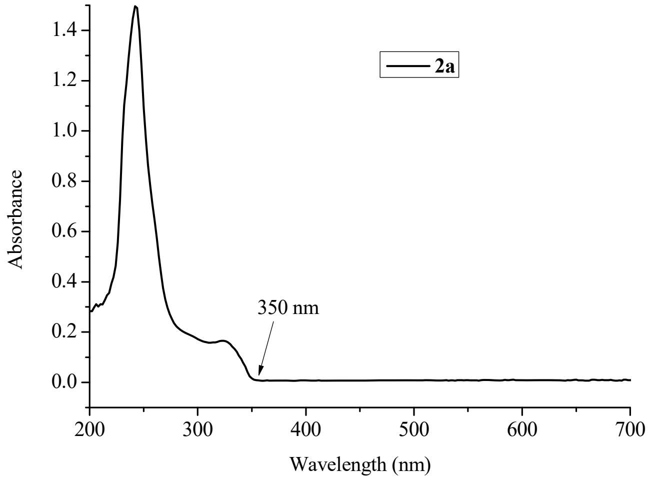


**Supplementary Figure 2.** UV-Vis absorption spectra of **2a** in $CH_2Cl_2$ (*Conc.* = 10 μM).

**Emission spectrum of the LED light**

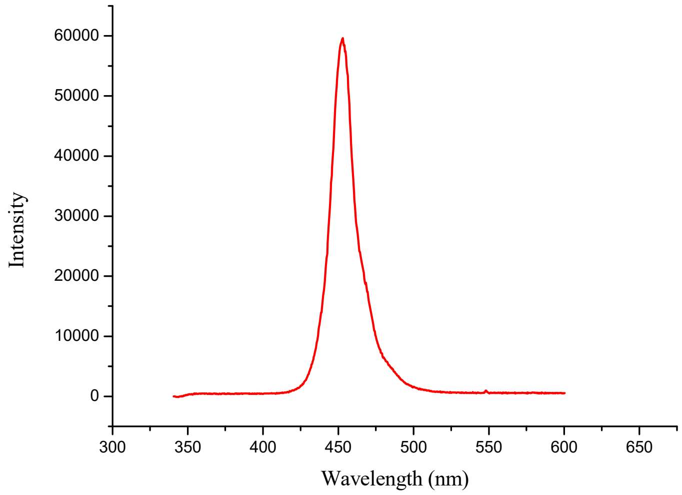


**Supplementary Figure 3.** Emission spectrum of the 3 W LED light of 455 nm

**Emission spectrum of the DPZ**

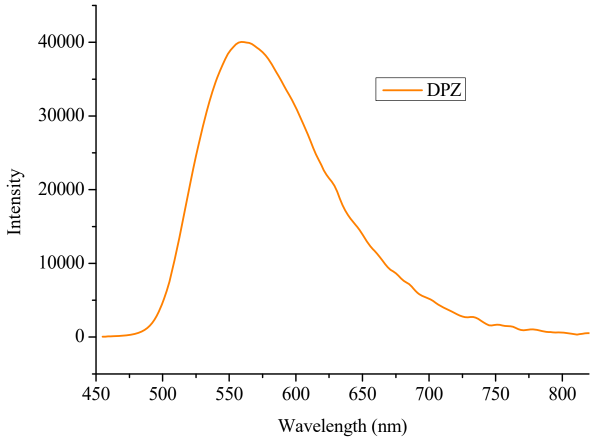


**Supplementary Figure 4.** The emission of DPZ in $CH_2Cl_2$ at 298K. DPZ solution was excited at 448 nm and the emission intensity at 560 nm was observed. [DPZ] = $1.0\times10^{-5}$ M.

**Cyclic voltammetry data**

Electrochemical potentials were obtained with a standard set of conditions to main internal consistency. Cyclic voltammograms were collected with a potentiostat. Samples were prepared with 0.01 mmol of **1a**, **2a** or **2a**+**C1** in 10 mL of 0.1 M tetrabutylammonium hexafluorophosphate in anhydrous acetonitrile. Measurements employed a radium glassy

carbon working electrode, platinum wire counter electrode, saturated KCl silver-silver chloride reference electrode. The obtained value was referenced to Ag/AgCl. The obtained value was referenced to Ag/AgCl and converted to SCE by adding 0.03 V.

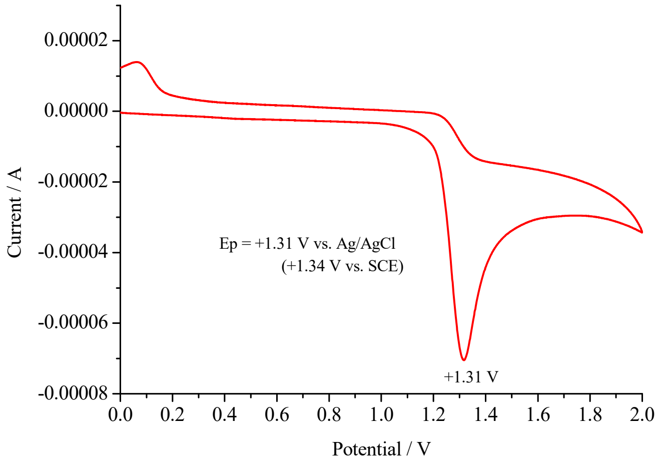


**Supplementary Figure 5.** Cyclic voltammogram of anthracene **1a** in MeCN.

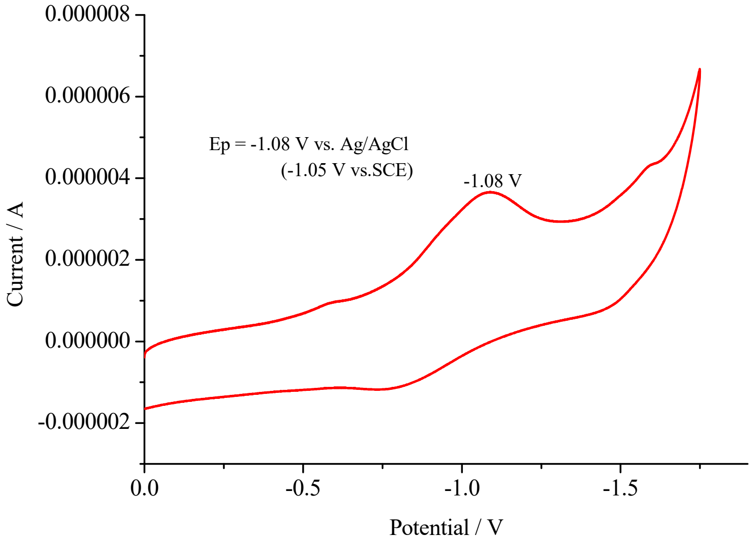


**Supplementary Figure 6.** Cyclic voltammogram of **2a** in MeCN.

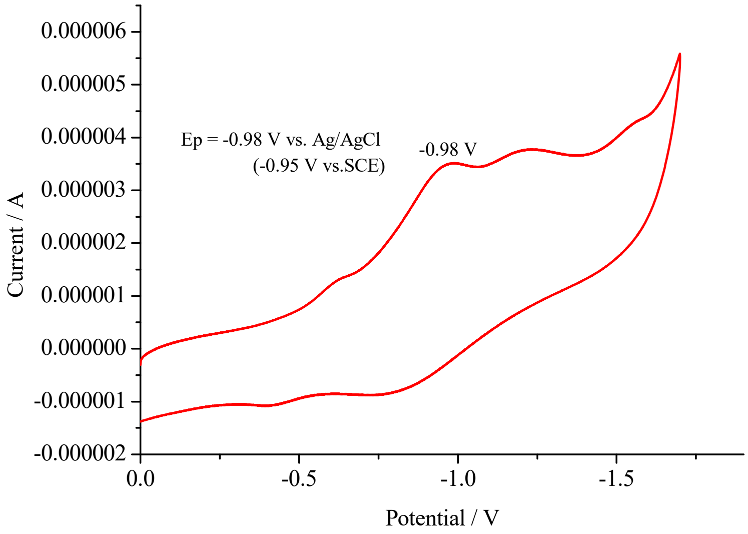


**Supplementary Figure 7.** Cyclic voltammogram of **2a** + **C1** in MeCN.

**Emission quenching experiments and Stern–Volmer quenching experiments**

Emission intensities were recorded on a spectrofluorometer. DPZ solution was excited at 448 nm and the emission intensity at 560 nm was observed. A solution of DPZ ($5.0\times10^{-5}$ M) in $CH_2Cl_2$ was added to the appropriate amount of quencher in 3.0 mL volumetric flask under $N_2$. The solution was transferred to a 3.0 mL quartz cell and the emission spectrum of the sample was collected.

Stern–Volmer experiments were conducted as previously described. Five separate solutions were prepared containing DPZ ($1.0 \times 10^{-5}$ M) and substrates (ranging from 0 to $3.2 \times 10^{-3}$ M) in $CH_2Cl_2$, and each was sparged with argon for 20 min. The solutions were irradiated at 448 nm, and the emission intensity (I) at 560 nm was compared to the initial emission intensity ($I_0$) at the same wavelength. These data result in a linear Stern–Volmer relationship, consistent with the proposed mechanism in which the photoexcited catalyst is not quenched by substrates.

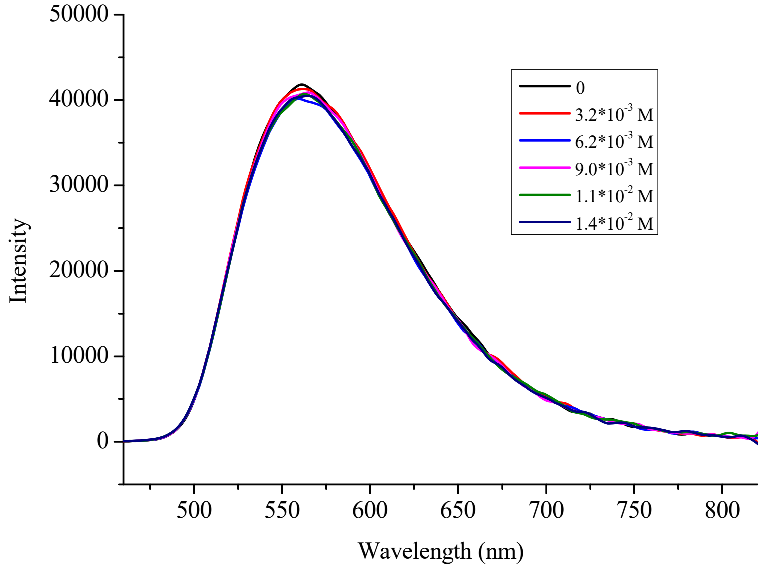


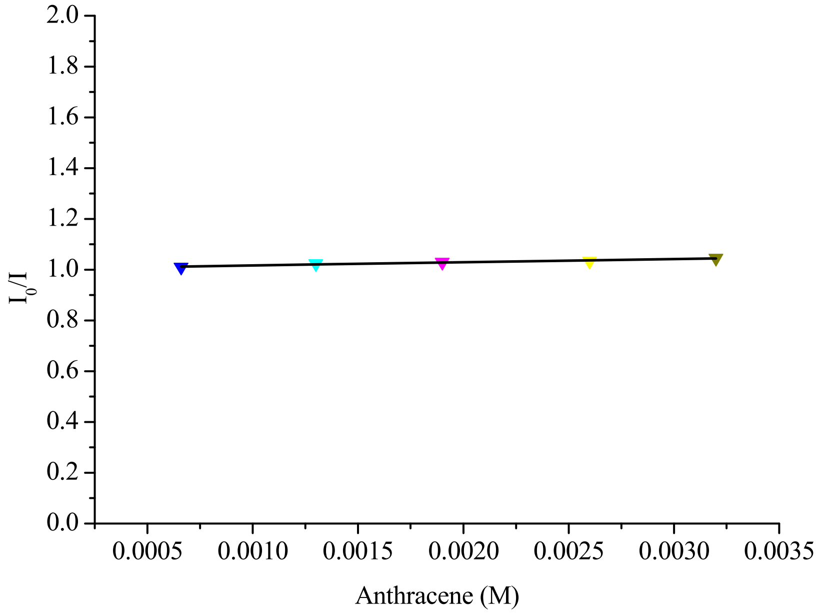


**Supplementary Figure 8.** The luminescence quenching and corresponding Stern–Volmer plots of DPZ by anthracene **1a** in deoxygenated Dichloromethane. No quenching observed.

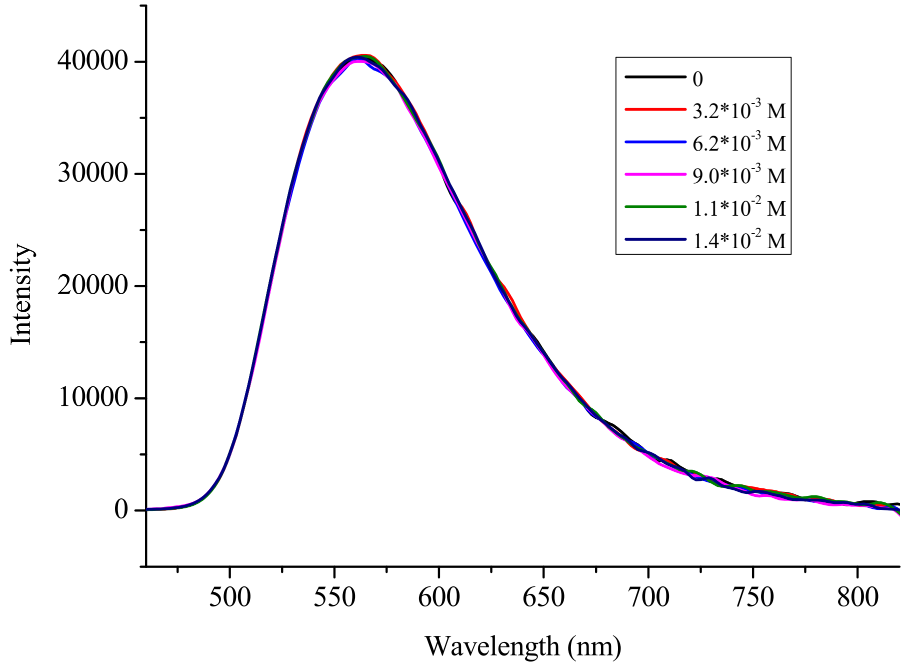


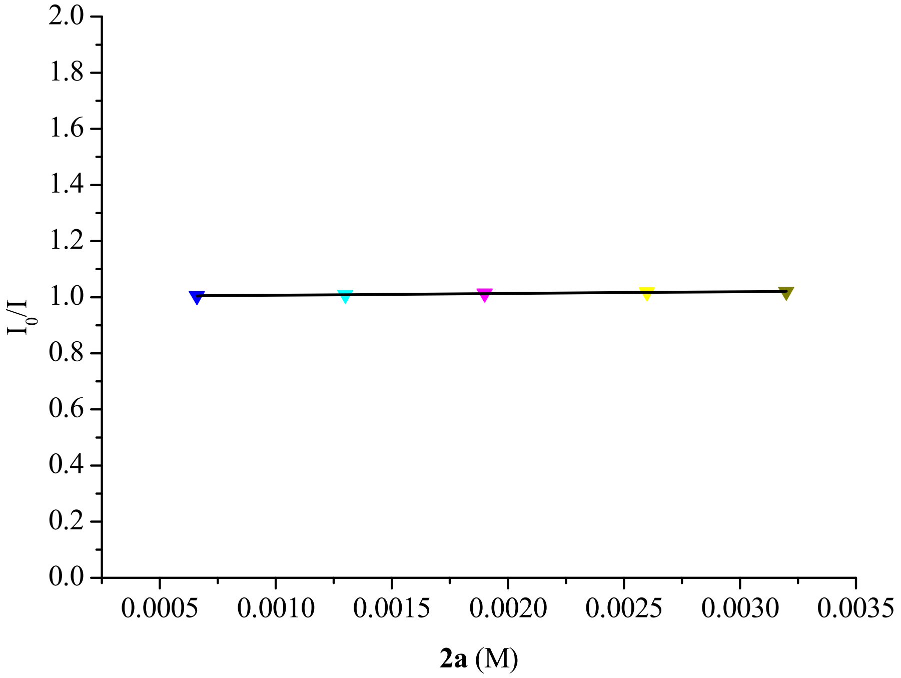


**Supplementary Figure 9.** The luminescence quenching and corresponding Stern–Volmer plots of DPZ by **2a** in deoxygenated Dichloromethane. No quenching observed.

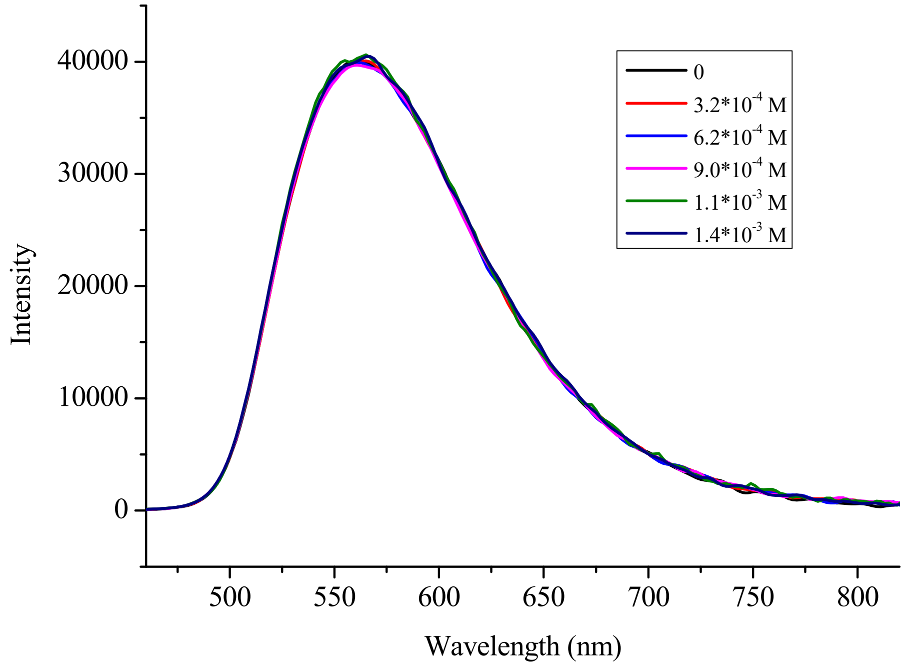


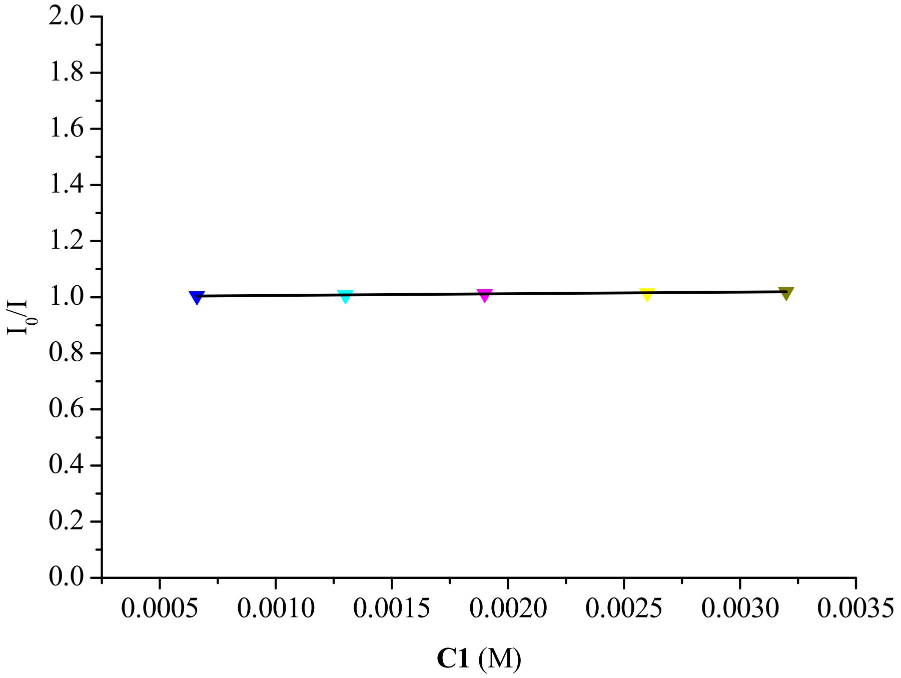


**Supplementary Figure 10.** The luminescence quenching and corresponding Stern–Volmer plots of DPZ by **C1** in deoxygenated Dichloromethane. No quenching observed.

**Determination of the oxidation potential of the triplet excited derived from 1a**

Excited state potentials are estimated using the equations as given:

$$E^{T*}Ox = E^{0'}Ox - E_T^{0-0}$$

Where $E^{T*}$ represents the triplet excited state potential, $E^{0'}$ represents the ground state potential, and $E_T^{0-0}$ refers to the energy gap between the zeroeth level vibrational levels of the ground and triplet excited state. $E_T^{0-0}$: Excited State Energy of the First Triplet Excited State $T_1$. Note that we present $E_T^{0-0}$ in units of eV to allow for easy combination with electrochemical potential (in units of V) in order to estimate excited state redox potentials.[1]

$$E_T^{0-0}(\mathbf{1a}) = +1.8\text{ eV}$$ [2]

$$E^{T*}Ox = E^{0'}Ox - E_T^{0-0}$$

$$E^{T*}Ox(\mathbf{1a}) = +1.34 - 1.80 = -0.46\text{ V}$$

**Triplet-triplet energy transfer study**

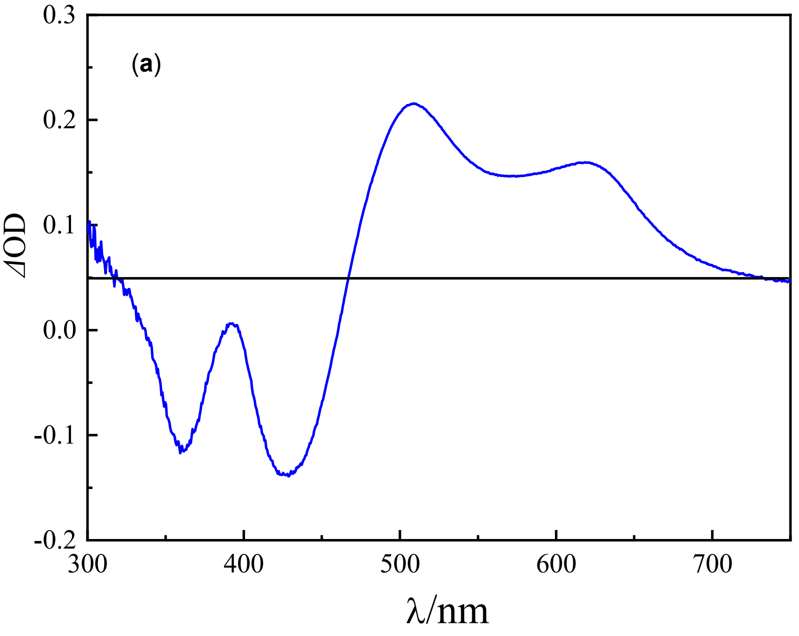

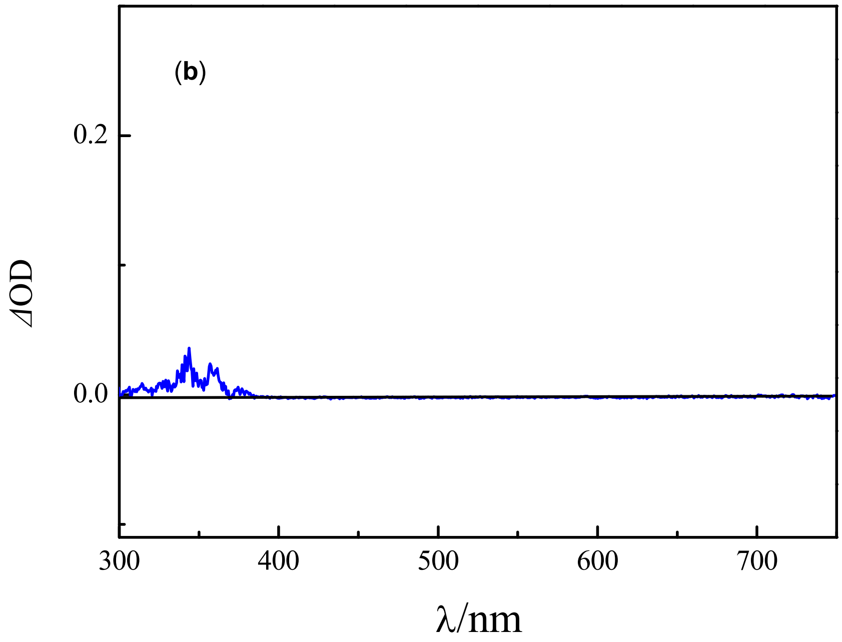


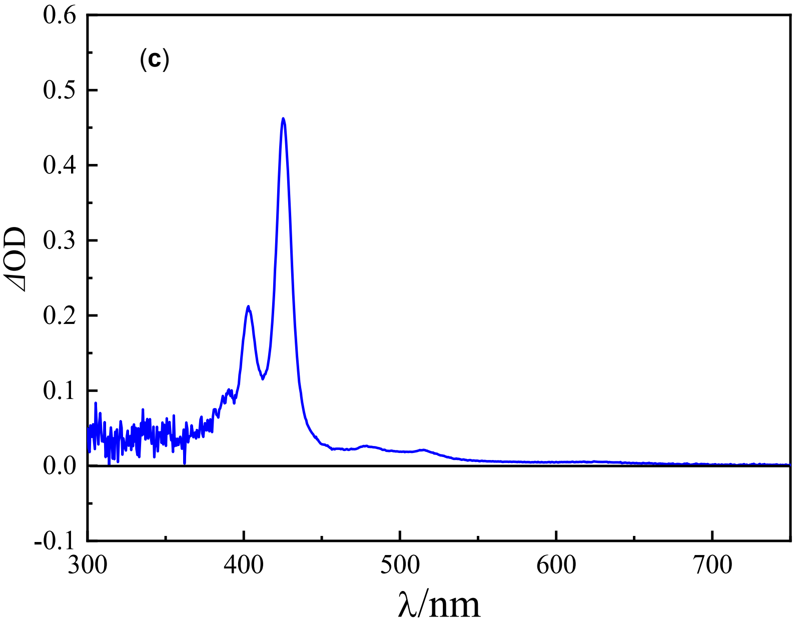


**Supplementary Figure 11.** Transient UV-Vis absorption spectrum of a deaerated aqueous solution containing 50 μM DPZ (Figure **a**), 10 mM anthracene (Figure **b**) and a THF solution containing 50 μM DPZ and 10 mM anthracene (Figure **c**), were recorded. The transient UV-Vis spectra (Figure **a** & **b** & **c**) were recorded with a time delay of 5 ns and a time integration of 200 ns upon 450 nm pulsed excitation (2 mJ per pulse).

**Comments:** When adding anthracene, two peaks (400 nm and 423 nm) can be obsereved. Accordingly, triplet-triplet energy transfer (TTET) between $^{3}$DPZ and anthracene is plausible.

## 5. Synthetic applications

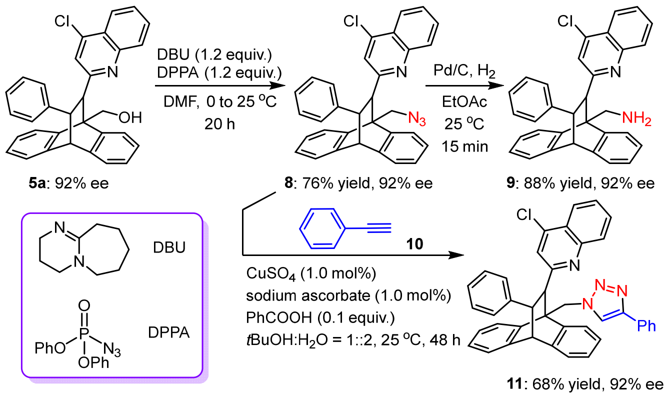


From **5a** to **8**: A mixture of **5a** (50 mg, 0.1 mmol, 1 equiv.) and DPPA (0.12 mmol, 1.2 equiv.) were dissolved in dry DMF (3 mL). To the mixture at 0°C, under $N_2$ was added DBU (0.12 mmol, 1.2 equiv.). The reaction was warmed to room temperature and stirred 20 h. After reaction completed, the mixture was washed with $H_2O$ (3 x 5 mL). The organic phase were then dried over $Na_2SO_4$ and concentrated in *vacuo*. The mixture was purified by column chromatography (silica gel, EtOAc/PE = 10:1), affording **8** as a yellow solid in 76% yield (37.9 mg).

From **8** to **9**: A solution of **8** (35 mg, 0.07 mmol) dissolved in ethyl acetate (5 mL) was stirred for 15 min in the presence of 10 percent w/w palladium-on-charcoal (5 mg) in an atmosphere of hydrogen under atmospheric pressure. At the end of this time, the rection mixture was filtered and the filtrate was concentrated by evaporation under reduced pressure. The mixture was purified by column chromatography (silica gel, $CH_2Cl_2$/MeOH = 30:1), affording **9** as a white solid in 88% yield (41.6 mg).

From **8** to **11**: To a solution of $CuSO_4$ (0.00067 mmol, 0.01 equiv.), sodium ascorbate (0.00067 mmol, 0.01 equiv.), and $PhCO_2H$ (0.0067 mmol, 0.1 equiv.) in *t*-BuOH/$H_2O$ (1:2 v/v, 2.0 mL) was added a mixture of phenylethyne (**10**, 7 mg, 0.067 mmol, 1 equiv.) and **8** (35 mg, 0.07 mmol, 1.05 equiv.) at room temperature. The resultant mixture was stirred continuously until the reaction system solidified completely. Then $CH_2Cl_2$(20 mL) was added to dissolve the crude product. The organic layer was washed with $H_2O$ and brine and dried over anhydrous $Na_2SO_4$. Removal of the solvent yielded a residue, which was purified by a short chromatography (silica gel, EtOAc/PE = 1:5) to give **11** (40.8 mg, 68%) as a yellow oil.

## 6. Determination of the absolute configurations

(1) Absolute configurations of products **3a-3r** and **3zd-3zg** are determined by *X*-ray structure analysis of product **3f**.

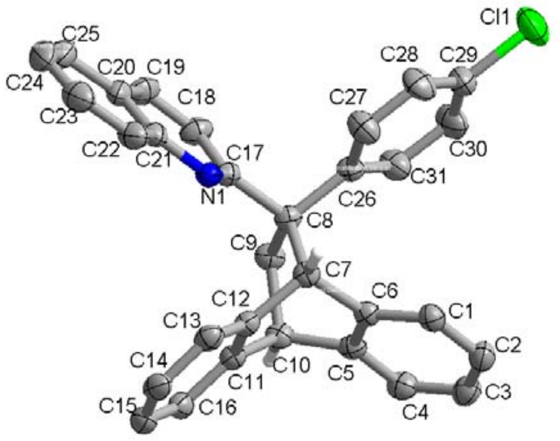


**Supplementary Figure 12.** Absolute configuration of **3f** (CCDC 2302299)

Displacement ellipsoids are drawn at the 30% probability level.

(Solvents: Chloroform/petroleum ether = 1:5)

**Supplementary Table 2. Crystal data and structure refinement for TD39082B_auto.**

| | |
|---|---|
| Identification code | TD39082B_auto |
| Empirical formula | $C_{32}H_{23}Cl_4N$ |
| Formula weight | 563.31 |
| Temperature/K | 293(2) |
| Crystal system | monoclinic |
| Space group | $P2_1$ |
| a/Å | 12.7174(8) |
| b/Å | 6.2835(6) |
| c/Å | 16.9484(10) |
| α/° | 90 |
| β/° | 96.370(6) |
| γ/° | 90 |
| Volume/Å$^3$ | 1345.97(16) |
| Z | 2 |
| $\rho_{calc}$g/cm$^3$ | 1.390 |
| μ/mm$^{-1}$ | 4.164 |
| F(000) | 580.0 |
| Crystal size/mm$^3$ | 0.15 × 0.12 × 0.1 |
| Radiation | CuKα (λ = 1.54184) |
| 2Θ range for data collection/° | 6.994 to 140.938 |

| | |
|---|---|
| Index ranges | -15 ≤ h ≤ 15, -7 ≤ k ≤ 6, -20 ≤ l ≤ 20 |
| Reflections collected | 10586 |
| Independent reflections | 4506 [$R_{int}$ = 0.0374, $R_{sigma}$ = 0.0538] |
| Data/restraints/parameters | 4506/1/338 |
| Goodness-of-fit on $F^2$ | 1.046 |
| Final R indexes [I>=2σ (I)] | $R_1$ = 0.0570, $wR_2$ = 0.1467 |
| Final R indexes [all data] | $R_1$ = 0.0697, $wR_2$ = 0.1613 |
| Largest diff. peak/hole / e $Å^{-3}$ | 0.39/-0.32 |
| Flack parameter | 0.021(14) |

**Supplementary Table 3. Fractional Atomic Coordinates (×$10^4$) and Equivalent Isotropic Displacement Parameters ($Å^2$×$10^3$) for TD39082B_auto. $U_{eq}$ is defined as 1/3 of of the trace of the orthogonalised $U_{IJ}$ tensor.**

| Atom | *x* | *y* | *z* | U(eq) |
|---|---|---|---|---|
| Cl1 | 3989.4(18) | 3337(5) | 4008.6(10) | 105.1(8) |
| N1 | 3967(3) | 4645(8) | 8201(2) | 46.0(9) |
| C1 | 546(4) | 3416(10) | 6253(3) | 51.4(11) |
| C2 | -354(4) | 3869(12) | 5739(3) | 62.0(15) |
| C3 | -827(4) | 5856(12) | 5768(3) | 65.4(16) |
| C4 | -410(4) | 7400(10) | 6294(3) | 57.8(14) |
| C5 | 483(4) | 6932(9) | 6807(3) | 46.9(11) |
| C6 | 954(3) | 4910(9) | 6798(3) | 43.2(10) |
| C7 | 1923(3) | 4673(9) | 7404(3) | 43.5(10) |
| C8 | 2749(3) | 6375(8) | 7176(3) | 42.1(10) |
| C9 | 2216(4) | 8605(9) | 7220(3) | 49.7(11) |
| C10 | 1052(4) | 8393(9) | 7424(3) | 47.0(10) |
| C11 | 1113(4) | 7260(9) | 8217(3) | 47.3(11) |
| C12 | 1589(3) | 5259(8) | 8206(3) | 42.6(10) |
| C13 | 1701(4) | 4017(10) | 8881(3) | 53.5(12) |
| C14 | 1312(4) | 4751(12) | 9567(3) | 61.2(15) |
| C15 | 834(4) | 6738(12) | 9574(3) | 63.4(16) |
| C16 | 737(4) | 7995(10) | 8902(3) | 53.0(12) |
| C17 | 3769(3) | 6305(9) | 7742(3) | 43.3(10) |
| C18 | 4495(4) | 8021(10) | 7716(3) | 59.3(13) |
| C19 | 5409(4) | 8005(11) | 8199(4) | 63.5(14) |
| C20 | 5627(4) | 6312(12) | 8730(3) | 58.8(14) |
| C21 | 4892(4) | 4607(10) | 8701(3) | 50.9(12) |
| C22 | 5104(4) | 2851(12) | 9199(3) | 63.5(16) |
| C23 | 6016(5) | 2771(15) | 9719(3) | 79(2) |
| C24 | 6735(5) | 4477(16) | 9752(4) | 83(2) |
| C25 | 6548(5) | 6167(14) | 9279(4) | 73.4(19) |
| C26 | 3061(4) | 5737(9) | 6351(3) | 46.1(11) |
| C27 | 3650(5) | 3881(11) | 6301(3) | 64.6(15) |
| C28 | 3931(5) | 3153(12) | 5588(4) | 72.0(17) |

| | | | | |
|---|---|---|---|---|
| C29 | 3630(4) | 4275(12) | 4911(3) | 66.6(17) |
| C30 | 3049(6) | 6052(15) | 4933(3) | 81(2) |
| C31 | 2772(5) | 6803(12) | 5656(3) | 66.3(16) |
| Cl2 | 6862(2) | 3610(6) | 7206.3(15) | 139.7(13) |
| Cl3 | 8118.7(19) | 49(5) | 7815.7(19) | 119.2(9) |
| Cl4 | 8830(2) | 4292(6) | 8183.3(19) | 138.1(12) |
| C32 | 7722(8) | 2718(19) | 7959(5) | 75(3) |
| C32A | 8161(18) | 2770(40) | 7495(12) | 75(3) |

**Supplementary Table 4. Anisotropic Displacement Parameters (Å$^2$×10$^3$) for TD39082B_auto. The Anisotropic displacement factor exponent takes the form: -2π$^2$[h$^2$a*$^2$U$_{11}$+2hka*b*U$_{12}$+…].**

| Atom | $U_{11}$ | $U_{22}$ | $U_{33}$ | $U_{23}$ | $U_{13}$ | $U_{12}$ |
|---|---|---|---|---|---|---|
| Cl1 | 126.6(15) | 130(2) | 62.5(8) | -22.4(11) | 25.9(9) | 11.8(15) |
| N1 | 42.3(18) | 50(3) | 46.2(18) | 2.3(19) | 8.0(14) | 1.9(17) |
| C1 | 55(2) | 50(3) | 49(2) | 2(2) | 6.5(18) | -1(2) |
| C2 | 61(3) | 72(4) | 52(3) | -5(3) | -2(2) | -8(3) |
| C3 | 54(3) | 85(5) | 55(3) | 9(3) | -4(2) | 3(3) |
| C4 | 57(3) | 60(4) | 57(3) | 12(3) | 9(2) | 12(3) |
| C5 | 43(2) | 50(3) | 48(2) | 10(2) | 7.4(18) | 1(2) |
| C6 | 45(2) | 42(3) | 43(2) | 6(2) | 6.1(17) | -2(2) |
| C7 | 45(2) | 39(3) | 47(2) | 4(2) | 4.0(17) | 2.3(19) |
| C8 | 41(2) | 38(3) | 49(2) | 3(2) | 6.7(17) | -0.3(19) |
| C9 | 52(2) | 39(3) | 59(2) | 5(2) | 11.9(19) | 3(2) |
| C10 | 49(2) | 41(3) | 51(2) | 5(2) | 6.7(18) | 9(2) |
| C11 | 40(2) | 54(3) | 48(2) | -2(2) | 3.7(17) | -3(2) |
| C12 | 39(2) | 42(3) | 46(2) | 3.4(19) | 1.9(16) | -1.8(19) |
| C13 | 46(2) | 57(4) | 57(3) | 11(2) | 4.8(19) | -6(2) |
| C14 | 48(2) | 90(5) | 45(2) | 16(3) | 3.0(19) | -3(3) |
| C15 | 49(3) | 92(5) | 49(3) | -5(3) | 6(2) | -5(3) |
| C16 | 46(2) | 57(4) | 57(3) | -9(3) | 4.9(18) | -3(2) |
| C17 | 42(2) | 43(3) | 46(2) | -1(2) | 7.3(17) | 1(2) |
| C18 | 51(3) | 50(4) | 77(3) | 3(3) | 9(2) | -3(2) |
| C19 | 50(3) | 52(4) | 89(4) | -7(3) | 10(2) | -11(2) |
| C20 | 44(2) | 74(4) | 58(3) | -20(3) | 5(2) | 3(3) |
| C21 | 48(2) | 62(4) | 44(2) | -4(2) | 9.6(18) | 10(2) |
| C22 | 56(3) | 81(5) | 55(3) | 13(3) | 9(2) | 11(3) |
| C23 | 71(4) | 111(7) | 56(3) | 16(4) | 7(3) | 29(4) |
| C24 | 57(3) | 130(8) | 59(3) | -12(4) | -11(2) | 23(4) |
| C25 | 51(3) | 88(5) | 79(4) | -22(4) | -6(3) | 6(3) |
| C26 | 45(2) | 45(3) | 48(2) | 4(2) | 6.7(18) | 0(2) |
| C27 | 86(4) | 55(4) | 54(3) | 9(3) | 16(2) | 19(3) |
| C28 | 87(4) | 61(4) | 70(3) | -5(3) | 20(3) | 22(4) |
| C29 | 63(3) | 85(5) | 52(3) | -11(3) | 9(2) | 4(3) |

| | | | | | | |
|---|---|---|---|---|---|---|
| C30 | 88(4) | 109(7) | 45(3) | 15(3) | 8(3) | 22(4) |
| C31 | 71(3) | 73(5) | 56(3) | 12(3) | 9(2) | 22(3) |
| Cl2 | 142(2) | 168(3) | 101.5(15) | -27.3(18) | -21.4(14) | 67(2) |
| Cl3 | 92.8(13) | 97.5(18) | 168(2) | -10.8(17) | 18.4(14) | 11.1(13) |
| Cl4 | 102.5(15) | 146(3) | 164(2) | 3(2) | 6.9(15) | -58.4(18) |
| C32 | 77(5) | 85(7) | 65(4) | -8(4) | 24(3) | -9(5) |
| C32A | 77(5) | 85(7) | 65(4) | -8(4) | 24(3) | -9(5) |

**Supplementary Table 5. Bond Lengths for TD39082B_auto.**

| **Atom** | **Atom** | **Length/Å** | **Atom** | **Atom** | **Length/Å** |
|---|---|---|---|---|---|
| Cl1 | C29 | 1.747(6) | C15 | C16 | 1.381(9) |
| N1 | C17 | 1.308(7) | C17 | C18 | 1.424(8) |
| N1 | C21 | 1.371(6) | C18 | C19 | 1.346(8) |
| C1 | C2 | 1.389(8) | C19 | C20 | 1.402(9) |
| C1 | C6 | 1.377(8) | C20 | C21 | 1.419(9) |
| C2 | C3 | 1.389(10) | C20 | C25 | 1.415(8) |
| C3 | C4 | 1.383(9) | C21 | C22 | 1.398(8) |
| C4 | C5 | 1.383(7) | C22 | C23 | 1.378(9) |
| C5 | C6 | 1.406(8) | C23 | C24 | 1.406(12) |
| C5 | C10 | 1.514(7) | C24 | C25 | 1.336(12) |
| C6 | C7 | 1.522(6) | C26 | C27 | 1.394(8) |
| C7 | C8 | 1.577(6) | C26 | C31 | 1.369(8) |
| C7 | C12 | 1.514(6) | C27 | C28 | 1.377(8) |
| C8 | C9 | 1.562(7) | C28 | C29 | 1.364(9) |
| C8 | C17 | 1.526(6) | C29 | C30 | 1.341(10) |
| C8 | C26 | 1.548(6) | C30 | C31 | 1.395(9) |
| C9 | C10 | 1.562(6) | Cl2 | C32 | 1.682(11) |
| C10 | C11 | 1.515(7) | Cl2 | C32A | 1.75(2) |
| C11 | C12 | 1.396(8) | Cl3 | C32 | 1.776(12) |
| C11 | C16 | 1.383(7) | Cl3 | C32A | 1.80(3) |
| C12 | C13 | 1.379(7) | Cl4 | C32 | 1.729(10) |
| C13 | C14 | 1.392(8) | Cl4 | C32A | 1.67(2) |
| C14 | C15 | 1.389(10) | | | |

**Supplementary Table 6. Bond Angles for TD39082B_auto.**

| **Atom** | **Atom** | **Atom** | **Angle/°** | **Atom** | **Atom** | **Atom** | **Angle/°** |
|---|---|---|---|---|---|---|---|
| C17 | N1 | C21 | 118.5(5) | C15 | C16 | C11 | 119.7(6) |
| C6 | C1 | C2 | 120.2(6) | N1 | C17 | C8 | 119.4(4) |
| C1 | C2 | C3 | 119.5(6) | N1 | C17 | C18 | 122.7(4) |
| C4 | C3 | C2 | 121.1(5) | C18 | C17 | C8 | 117.8(5) |
| C5 | C4 | C3 | 119.0(6) | C19 | C18 | C17 | 119.6(6) |
| C4 | C5 | C6 | 120.4(5) | C18 | C19 | C20 | 119.5(6) |
| C4 | C5 | C10 | 127.0(5) | C19 | C20 | C21 | 117.9(5) |

| | | | | | | | |
|---|---|---|---|---|---|---|---|
| C6 | C5 | C10 | 112.6(4) | C19 | C20 | C25 | 124.1(7) |
| C1 | C6 | C5 | 119.7(4) | C25 | C20 | C21 | 117.9(7) |
| C1 | C6 | C7 | 127.0(5) | N1 | C21 | C20 | 121.7(5) |
| C5 | C6 | C7 | 113.3(4) | N1 | C21 | C22 | 118.8(5) |
| C6 | C7 | C8 | 106.2(4) | C22 | C21 | C20 | 119.5(5) |
| C12 | C7 | C6 | 107.2(4) | C23 | C22 | C21 | 120.5(7) |
| C12 | C7 | C8 | 108.4(4) | C22 | C23 | C24 | 119.7(7) |
| C9 | C8 | C7 | 107.0(4) | C25 | C24 | C23 | 120.7(6) |
| C17 | C8 | C7 | 111.7(4) | C24 | C25 | C20 | 121.7(8) |
| C17 | C8 | C9 | 109.8(4) | C27 | C26 | C8 | 117.7(4) |
| C17 | C8 | C26 | 106.0(3) | C31 | C26 | C8 | 125.4(5) |
| C26 | C8 | C7 | 106.9(4) | C31 | C26 | C27 | 116.9(5) |
| C26 | C8 | C9 | 115.5(4) | C28 | C27 | C26 | 121.6(5) |
| C8 | C9 | C10 | 111.1(4) | C29 | C28 | C27 | 119.4(6) |
| C5 | C10 | C9 | 106.9(4) | C28 | C29 | Cl1 | 119.0(5) |
| C5 | C10 | C11 | 107.5(4) | C30 | C29 | Cl1 | 120.2(5) |
| C11 | C10 | C9 | 106.3(4) | C30 | C29 | C28 | 120.8(5) |
| C12 | C11 | C10 | 113.0(4) | C29 | C30 | C31 | 120.0(6) |
| C16 | C11 | C10 | 126.8(5) | C26 | C31 | C30 | 121.3(6) |
| C16 | C11 | C12 | 120.1(5) | Cl2 | C32 | Cl3 | 112.5(6) |
| C11 | C12 | C7 | 113.3(4) | Cl2 | C32 | Cl4 | 114.9(7) |
| C13 | C12 | C7 | 126.4(5) | Cl4 | C32 | Cl3 | 109.5(6) |
| C13 | C12 | C11 | 120.3(5) | Cl2 | C32A | Cl3 | 108.2(12) |
| C12 | C13 | C14 | 119.4(6) | Cl4 | C32A | Cl2 | 114.5(14) |
| C15 | C14 | C13 | 120.1(5) | Cl4 | C32A | Cl3 | 111.3(13) |
| C16 | C15 | C14 | 120.3(5) | | | | |

**Supplementary Table 7. Torsion Angles for TD39082B_auto.**

| A | B | C | D | Angle/° | A | B | C | D | Angle/° |
|---|---|---|---|---|---|---|---|---|---|
| Cl1 | C29 | C30 | C31 | -179.5(6) | C10 | C5 | C6 | C1 | -178.1(4) |
| N1 | C17 | C18 | C19 | -2.5(8) | C10 | C5 | C6 | C7 | -0.3(5) |
| N1 | C21 | C22 | C23 | -178.9(5) | C10 | C11 | C12 | C7 | -0.7(6) |
| C1 | C2 | C3 | C4 | 0.8(9) | C10 | C11 | C12 | C13 | -179.7(4) |
| C1 | C6 | C7 | C8 | 116.4(5) | C10 | C11 | C16 | C15 | 178.5(5) |
| C1 | C6 | C7 | C12 | -127.9(5) | C11 | C12 | C13 | C14 | 1.5(7) |
| C2 | C1 | C6 | C5 | -3.0(7) | C12 | C7 | C8 | C9 | -55.1(5) |
| C2 | C1 | C6 | C7 | 179.5(5) | C12 | C7 | C8 | C17 | 65.1(5) |
| C2 | C3 | C4 | C5 | -1.2(8) | C12 | C7 | C8 | C26 | -179.4(4) |
| C3 | C4 | C5 | C6 | -0.4(7) | C12 | C11 | C16 | C15 | -0.2(7) |
| C3 | C4 | C5 | C10 | -179.7(5) | C12 | C13 | C14 | C15 | -1.2(8) |
| C4 | C5 | C6 | C1 | 2.5(7) | C13 | C14 | C15 | C16 | 0.2(8) |
| C4 | C5 | C6 | C7 | -179.7(4) | C14 | C15 | C16 | C11 | 0.5(8) |
| C4 | C5 | C10 | C9 | -121.4(5) | C16 | C11 | C12 | C7 | 178.2(4) |

| | | | | | | | | | |
|---|---|---|---|---|---|---|---|---|---|
| C4 | C5 | C10 | C11 | 124.7(5) | C16 | C11 | C12 | C13 | -0.9(7) |
| C5 | C6 | C7 | C8 | -61.2(5) | C17 | N1 | C21 | C20 | 0.1(7) |
| C5 | C6 | C7 | C12 | 54.5(5) | C17 | N1 | C21 | C22 | 179.3(4) |
| C5 | C10 | C11 | C12 | 55.5(5) | C17 | C8 | C9 | C10 | -124.4(4) |
| C5 | C10 | C11 | C16 | -123.3(5) | C17 | C8 | C26 | C27 | 50.7(6) |
| C6 | C1 | C2 | C3 | 1.4(8) | C17 | C8 | C26 | C31 | -132.0(6) |
| C6 | C5 | C10 | C9 | 59.2(5) | C17 | C18 | C19 | C20 | -1.0(8) |
| C6 | C5 | C10 | C11 | -54.6(5) | C18 | C19 | C20 | C21 | 3.7(8) |
| C6 | C7 | C8 | C9 | 59.9(5) | C18 | C19 | C20 | C25 | -178.1(6) |
| C6 | C7 | C8 | C17 | -179.9(4) | C19 | C20 | C21 | N1 | -3.4(7) |
| C6 | C7 | C8 | C26 | -64.4(5) | C19 | C20 | C21 | C22 | 177.4(5) |
| C6 | C7 | C12 | C11 | -53.9(5) | C19 | C20 | C25 | C24 | -177.4(6) |
| C6 | C7 | C12 | C13 | 125.0(5) | C20 | C21 | C22 | C23 | 0.4(8) |
| C7 | C8 | C9 | C10 | -3.0(5) | C21 | N1 | C17 | C8 | 179.5(4) |
| C7 | C8 | C17 | N1 | 16.1(6) | C21 | N1 | C17 | C18 | 2.9(7) |
| C7 | C8 | C17 | C18 | -167.1(4) | C21 | C20 | C25 | C24 | 0.7(9) |
| C7 | C8 | C26 | C27 | -68.5(6) | C21 | C22 | C23 | C24 | 0.4(9) |
| C7 | C8 | C26 | C31 | 108.8(6) | C22 | C23 | C24 | C25 | -0.6(10) |
| C7 | C12 | C13 | C14 | -177.3(5) | C23 | C24 | C25 | C20 | 0.0(10) |
| C8 | C7 | C12 | C11 | 60.4(5) | C25 | C20 | C21 | N1 | 178.3(5) |
| C8 | C7 | C12 | C13 | -120.7(5) | C25 | C20 | C21 | C22 | -0.9(7) |
| C8 | C9 | C10 | C5 | -55.6(5) | C26 | C8 | C9 | C10 | 115.9(4) |
| C8 | C9 | C10 | C11 | 59.0(5) | C26 | C8 | C17 | N1 | -99.9(5) |
| C8 | C17 | C18 | C19 | -179.2(5) | C26 | C8 | C17 | C18 | 76.9(6) |
| C8 | C26 | C27 | C28 | 177.7(6) | C26 | C27 | C28 | C29 | 0.3(11) |
| C8 | C26 | C31 | C30 | -176.9(6) | C27 | C26 | C31 | C30 | 0.5(10) |
| C9 | C8 | C17 | N1 | 134.7(4) | C27 | C28 | C29 | Cl1 | -180.0(6) |
| C9 | C8 | C17 | C18 | -48.6(5) | C27 | C28 | C29 | C30 | -1.2(11) |
| C9 | C8 | C26 | C27 | 172.5(5) | C28 | C29 | C30 | C31 | 1.8(12) |
| C9 | C8 | C26 | C31 | -10.1(7) | C29 | C30 | C31 | C26 | -1.4(12) |
| C9 | C10 | C11 | C12 | -58.7(5) | C31 | C26 | C27 | C28 | 0.1(10) |
| C9 | C10 | C11 | C16 | 122.5(5) | | | | | |

**Supplementary Table 8. Hydrogen Atom Coordinates (Å×$10^4$) and Isotropic Displacement Parameters (Å$^2$×$10^3$) for TD39082B_auto.**

| **Atom** | ***x*** | ***y*** | ***z*** | **U(eq)** |
|---|---|---|---|---|
| H1 | 875 | 2099 | 6228 | 62 |
| H2 | -638 | 2850 | 5379 | 74 |
| H3 | -1435 | 6152 | 5429 | 78 |
| H4 | -725 | 8735 | 6302 | 69 |
| H7 | 2213 | 3228 | 7405 | 52 |
| H9A | 2218 | 9325 | 6713 | 60 |
| H9B | 2620 | 9462 | 7622 | 60 |
| H10 | 703 | 9780 | 7439 | 56 |

| | | | | |
|---|---|---|---|---|
| H13 | 2033 | 2699 | 8878 | 64 |
| H14 | 1373 | 3911 | 10021 | 73 |
| H15 | 577 | 7224 | 10034 | 76 |
| H16 | 422 | 9329 | 8909 | 64 |
| H18 | 4338 | 9148 | 7366 | 71 |
| H19 | 5893 | 9109 | 8180 | 76 |
| H22 | 4626 | 1727 | 9180 | 76 |
| H23 | 6155 | 1595 | 10047 | 95 |
| H24 | 7348 | 4428 | 10106 | 100 |
| H25 | 7035 | 7276 | 9310 | 88 |
| H27 | 3859 | 3115 | 6762 | 77 |
| H28 | 4322 | 1908 | 5567 | 86 |
| H30 | 2832 | 6783 | 4466 | 97 |
| H31 | 2382 | 8053 | 5666 | 80 |
| H32 | 7343 | 2716 | 8432 | 90 |
| H32A | 8540 | 2801 | 7022 | 90 |

(2) Relative configurations of products **3o-3r** are determined by *X*-ray structure analysis of product **3o**

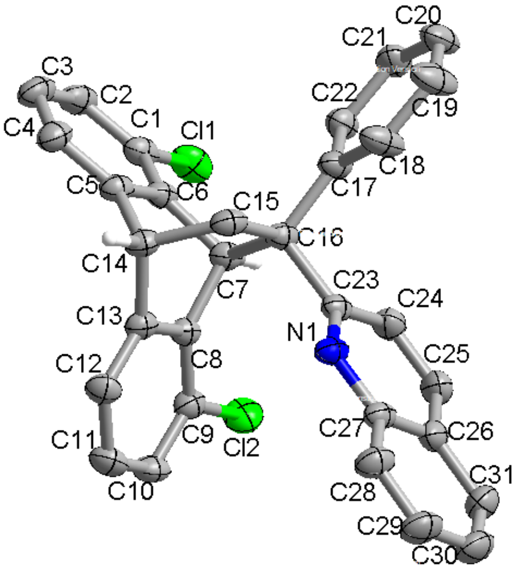


**Supplementary Figure 13.** Relative configuration of **3o** (CCDC 2301849)

Displacement ellipsoids are drawn at the 30% probability level.

(Solvents: ethyl acetate/petroleum ether = 1:10)

**Supplementary Table 9. Crystal data and structure refinement for TD39066_auto.**

| | |
|---|---|
| Identification code | TD39066_auto |
| Empirical formula | $C_{31}H_{21}Cl_2N$ |
| Formula weight | 478.39 |
| Temperature/K | 293(2) |
| Crystal system | triclinic |

| | |
|---|---|
| Space group | P-1 |
| a/Å | 8.9338(4) |
| b/Å | 12.2732(5) |
| c/Å | 12.3252(5) |
| α/° | 92.544(4) |
| β/° | 100.966(4) |
| γ/° | 94.279(4) |
| Volume/Å$^3$ | 1320.66(10) |
| Z | 2 |
| $\rho_{calc}$g/cm$^3$ | 1.203 |
| μ/mm$^{-1}$ | 2.340 |
| F(000) | 496.0 |
| Crystal size/mm$^3$ | 0.13 × 0.12 × 0.1 |
| Radiation | CuKα (λ = 1.54184) |
| 2Θ range for data collection/° | 7.236 to 134.154 |
| Index ranges | -10 ≤ h ≤ 10, -14 ≤ k ≤ 14, -13 ≤ l ≤ 14 |
| Reflections collected | 18101 |
| Independent reflections | 4719 [$R_{int}$ = 0.0400, $R_{sigma}$ = 0.0311] |
| Data/restraints/parameters | 4719/0/307 |
| Goodness-of-fit on F$^2$ | 1.039 |
| Final R indexes [I>=2σ (I)] | $R_1$ = 0.0450, w$R_2$ = 0.1282 |
| Final R indexes [all data] | $R_1$ = 0.0543, w$R_2$ = 0.1368 |
| Largest diff. peak/hole / e Å$^{-3}$ | 0.21/-0.39 |

**Supplementary Table 10. Fractional Atomic Coordinates (×10$^4$) and Equivalent Isotropic Displacement Parameters (Å$^2$×10$^3$) for TD39066_auto. $U_{eq}$ is defined as 1/3 of of the trace of the orthogonalised $U_{IJ}$ tensor.**

| Atom | *x* | *y* | *z* | U(eq) |
|---|---|---|---|---|
| Cl1 | 1660.5(8) | 7238.6(5) | 4785.4(5) | 72.4(2) |
| Cl2 | 4901.4(6) | 6142.8(5) | 7722.2(6) | 70.4(2) |
| N1 | 2148.3(19) | 7465.9(14) | 10151.4(13) | 51.4(4) |
| C1 | 98(3) | 6634.0(17) | 5263.0(17) | 55.0(5) |
| C2 | -1178(3) | 6212(2) | 4493.8(18) | 67.3(6) |
| C3 | -2383(3) | 5698(2) | 4857(2) | 71.8(7) |
| C4 | -2341(3) | 5569(2) | 5978.7(19) | 61.4(6) |
| C5 | -1058(2) | 5984.7(17) | 6735.4(16) | 50.3(5) |
| C6 | 163(2) | 6545.3(16) | 6387.2(15) | 47.2(4) |
| C7 | 1491(2) | 6915.3(16) | 7320.8(15) | 45.5(4) |
| C8 | 1921(2) | 5898.2(15) | 7921.6(15) | 43.5(4) |
| C9 | 3342(2) | 5495.1(17) | 8170.9(17) | 49.3(4) |
| C10 | 3568(3) | 4569.0(19) | 8764.3(19) | 59.8(5) |
| C11 | 2349(3) | 4046.9(19) | 9127(2) | 62.9(6) |
| C12 | 904(3) | 4434.0(18) | 8896.0(17) | 54.2(5) |
| C13 | 707(2) | 5347.1(16) | 8291.2(15) | 46.8(4) |
| C14 | -775(2) | 5887.9(18) | 7973.0(16) | 49.8(4) |
| C15 | -507(2) | 7067.9(18) | 8507.4(16) | 51.5(5) |
| C16 | 928(2) | 7682.0(16) | 8207.2(15) | 48.0(4) |
| C17 | 557(3) | 8821.6(17) | 7825.6(17) | 54.9(5) |
| C18 | -39(4) | 9505(2) | 8538(2) | 80.5(9) |
| C19 | -360(4) | 10560(3) | 8276(2) | 96.2(11) |

| | | | | |
|---|---|---|---|---|
| C20 | -105(4) | 10950(2) | 7302(2) | 84.1(9) |
| C21 | 463(3) | 10297(2) | 6588(2) | 71.2(7) |
| C22 | 806(3) | 9233.0(18) | 6847.1(18) | 59.0(5) |
| C23 | 2283(2) | 7864.3(16) | 9202.7(16) | 47.7(4) |
| C24 | 3639(3) | 8450.7(18) | 9035.5(19) | 60.8(5) |
| C25 | 4871(3) | 8602.8(19) | 9874(2) | 66.1(6) |
| C26 | 4774(3) | 8191.7(17) | 10911.1(19) | 59.7(5) |
| C27 | 3386(2) | 7619.2(17) | 11016.8(17) | 53.7(5) |
| C28 | 3253(3) | 7194(2) | 12037.1(18) | 66.4(6) |
| C29 | 4469(3) | 7322(3) | 12915(2) | 77.9(7) |
| C30 | 5832(3) | 7889(3) | 12811(2) | 82.4(8) |
| C31 | 6003(3) | 8329(2) | 11838(2) | 75.7(7) |

**Supplementary Table 11. Anisotropic Displacement Parameters (Å$^2$×10$^3$) for TD39066_auto. The Anisotropic displacement factor exponent takes the form: -2$\pi^2$[h$^2$a*$^2$U$_{11}$+2hka*b*U$_{12}$+…].**

| Atom | $U_{11}$ | $U_{22}$ | $U_{33}$ | $U_{23}$ | $U_{13}$ | $U_{12}$ |
|---|---|---|---|---|---|---|
| Cl1 | 104.0(5) | 71.6(4) | 51.0(3) | 14.3(3) | 31.9(3) | 18.5(3) |
| Cl2 | 48.5(3) | 78.2(4) | 89.1(4) | 5.0(3) | 22.2(3) | 12.4(2) |
| N1 | 54.9(9) | 60.0(9) | 39.5(9) | -0.7(7) | 4.4(7) | 21.0(7) |
| C1 | 73.0(13) | 56.3(11) | 39.2(11) | 4.2(9) | 11.2(9) | 26.4(10) |
| C2 | 91.9(18) | 72.9(14) | 36.3(11) | -0.5(10) | 1.3(11) | 33.4(13) |
| C3 | 71.6(15) | 87.1(17) | 48.9(13) | -10.3(12) | -11.4(11) | 24.3(13) |
| C4 | 52.6(11) | 73.4(14) | 55.2(13) | -5.7(11) | 0.3(9) | 17.2(10) |
| C5 | 50.7(10) | 59.3(11) | 42.0(10) | 1.0(8) | 6.3(8) | 20.4(9) |
| C6 | 57.3(11) | 50.2(10) | 35.9(10) | 2.2(8) | 7.7(8) | 20.7(8) |
| C7 | 49.9(10) | 52.1(10) | 37.0(10) | 3.3(8) | 10.9(8) | 14.2(8) |
| C8 | 47.5(9) | 49.2(9) | 34.8(9) | -1.1(7) | 7.6(7) | 14.9(8) |
| C9 | 46.5(10) | 57.5(11) | 44.3(10) | -4.8(8) | 8.2(8) | 13.7(8) |
| C10 | 57.6(12) | 66.4(13) | 57.1(13) | 6.8(10) | 5.9(10) | 29.7(10) |
| C11 | 72.9(14) | 62.8(12) | 57.2(13) | 16.3(10) | 13.3(11) | 24.6(11) |
| C12 | 59.8(11) | 60.1(11) | 46.1(11) | 10.7(9) | 13.9(9) | 13.0(9) |
| C13 | 48.0(10) | 57.1(11) | 36.6(9) | 0.6(8) | 8.4(7) | 13.7(8) |
| C14 | 44.0(10) | 65.0(12) | 42.3(10) | 5.6(9) | 8.8(8) | 14.2(8) |
| C15 | 48.8(10) | 71.4(12) | 37.0(10) | 2.5(9) | 7.9(8) | 23.6(9) |
| C16 | 54.6(10) | 54.7(11) | 35.9(10) | 1.5(8) | 6.0(8) | 20.5(8) |
| C17 | 66.9(12) | 57.0(11) | 39.9(11) | 0.2(9) | 1.0(9) | 25.0(9) |
| C18 | 121(2) | 80.8(16) | 46.9(13) | 8.8(12) | 15.6(13) | 58.4(17) |
| C19 | 146(3) | 83.1(18) | 65.2(17) | 1.0(14) | 13.1(17) | 70(2) |
| C20 | 124(2) | 62.2(14) | 62.0(16) | 5.4(12) | -6.8(15) | 42.4(15) |
| C21 | 96.0(18) | 62.4(13) | 49.2(13) | 8.4(11) | -4.6(12) | 12.5(12) |
| C22 | 75.3(14) | 54.1(11) | 46.5(12) | -0.4(9) | 5.8(10) | 14.2(10) |
| C23 | 58.2(11) | 45.4(9) | 39.5(10) | -1.8(8) | 4.9(8) | 18.4(8) |
| C24 | 72.9(14) | 53.4(11) | 53.3(13) | 6.7(10) | 4.0(10) | 6.0(10) |
| C25 | 69.0(14) | 53.4(12) | 70.6(15) | 3.6(11) | 2.9(11) | -0.6(10) |
| C26 | 67.7(13) | 48.7(10) | 56.7(13) | -6.9(9) | -4.5(10) | 13.8(9) |
| C27 | 60.7(12) | 56.6(11) | 41.8(11) | -3.9(9) | 0.2(9) | 22.2(9) |
| C28 | 66.7(14) | 87.0(16) | 44.6(12) | 1.2(11) | 4.1(10) | 19.9(12) |
| C29 | 85.2(18) | 100.1(19) | 43.5(13) | 3.6(12) | -5.7(12) | 22.8(15) |
| C30 | 80.8(18) | 93.4(18) | 60.3(16) | -8.0(14) | -20.5(13) | 20.4(15) |

| | | | | | | |
|---|---|---|---|---|---|---|
| C31 | 75.7(16) | 70.5(14) | 69.2(17) | -6.5(12) | -13.1(13) | 6.8(12) |

**Supplementary Table 12. Bond Lengths for TD39066_auto.**

| Atom | Atom | Length/Å | Atom | Atom | Length/Å |
|---|---|---|---|---|---|
| Cl1 | C1 | 1.745(2) | C13 | C14 | 1.518(3) |
| Cl2 | C9 | 1.745(2) | C14 | C15 | 1.547(3) |
| N1 | C23 | 1.310(3) | C15 | C16 | 1.552(3) |
| N1 | C27 | 1.378(3) | C16 | C17 | 1.535(3) |
| C1 | C2 | 1.387(4) | C16 | C23 | 1.546(3) |
| C1 | C6 | 1.385(3) | C17 | C18 | 1.391(3) |
| C2 | C3 | 1.366(4) | C17 | C22 | 1.378(3) |
| C3 | C4 | 1.392(4) | C18 | C19 | 1.387(4) |
| C4 | C5 | 1.381(3) | C19 | C20 | 1.364(4) |
| C5 | C6 | 1.393(3) | C20 | C21 | 1.358(4) |
| C5 | C14 | 1.510(3) | C21 | C22 | 1.400(3) |
| C6 | C7 | 1.514(3) | C23 | C24 | 1.416(3) |
| C7 | C8 | 1.516(3) | C24 | C25 | 1.354(3) |
| C7 | C16 | 1.588(3) | C25 | C26 | 1.411(4) |
| C8 | C9 | 1.382(3) | C26 | C27 | 1.410(3) |
| C8 | C13 | 1.396(3) | C26 | C31 | 1.421(3) |
| C9 | C10 | 1.385(3) | C27 | C28 | 1.405(3) |
| C10 | C11 | 1.379(3) | C28 | C29 | 1.374(3) |
| C11 | C12 | 1.392(3) | C29 | C30 | 1.387(4) |
| C12 | C13 | 1.377(3) | C30 | C31 | 1.367(4) |

**Supplementary Table 13. Bond Angles for TD39066_auto.**

| Atom | Atom | Atom | Angle/° | Atom | Atom | Atom | Angle/° |
|---|---|---|---|---|---|---|---|
| C23 | N1 | C27 | 118.3(2) | C14 | C15 | C16 | 111.03(15) |
| C2 | C1 | Cl1 | 118.62(17) | C15 | C16 | C7 | 107.08(16) |
| C6 | C1 | Cl1 | 120.22(18) | C17 | C16 | C7 | 114.76(16) |
| C6 | C1 | C2 | 121.1(2) | C17 | C16 | C15 | 110.10(16) |
| C3 | C2 | C1 | 119.1(2) | C17 | C16 | C23 | 106.45(16) |
| C2 | C3 | C4 | 121.3(2) | C23 | C16 | C7 | 105.82(15) |
| C5 | C4 | C3 | 119.0(2) | C23 | C16 | C15 | 112.68(16) |
| C4 | C5 | C6 | 120.7(2) | C18 | C17 | C16 | 117.4(2) |
| C4 | C5 | C14 | 126.7(2) | C22 | C17 | C16 | 125.18(19) |
| C6 | C5 | C14 | 112.62(18) | C22 | C17 | C18 | 117.4(2) |
| C1 | C6 | C5 | 118.71(19) | C19 | C18 | C17 | 121.3(3) |
| C1 | C6 | C7 | 127.4(2) | C20 | C19 | C18 | 120.3(3) |
| C5 | C6 | C7 | 113.66(16) | C21 | C20 | C19 | 119.6(2) |
| C6 | C7 | C8 | 106.10(16) | C20 | C21 | C22 | 120.6(2) |
| C6 | C7 | C16 | 109.84(15) | C17 | C22 | C21 | 120.8(2) |
| C8 | C7 | C16 | 104.73(15) | N1 | C23 | C16 | 119.57(19) |
| C9 | C8 | C7 | 128.25(19) | N1 | C23 | C24 | 122.75(19) |
| C9 | C8 | C13 | 118.02(18) | C24 | C23 | C16 | 117.67(18) |
| C13 | C8 | C7 | 113.68(16) | C25 | C24 | C23 | 119.8(2) |
| C8 | C9 | Cl2 | 120.14(16) | C24 | C25 | C26 | 119.3(2) |

| | | | | | | | |
|---|---|---|---|---|---|---|---|
| C8 | C9 | C10 | 121.5(2) | C25 | C26 | C31 | 123.0(2) |
| C10 | C9 | Cl2 | 118.34(16) | C27 | C26 | C25 | 117.7(2) |
| C11 | C10 | C9 | 119.17(19) | C27 | C26 | C31 | 119.4(2) |
| C10 | C11 | C12 | 120.8(2) | N1 | C27 | C26 | 122.1(2) |
| C13 | C12 | C11 | 118.8(2) | N1 | C27 | C28 | 118.7(2) |
| C8 | C13 | C14 | 112.31(17) | C28 | C27 | C26 | 119.2(2) |
| C12 | C13 | C8 | 121.66(18) | C29 | C28 | C27 | 120.3(3) |
| C12 | C13 | C14 | 126.02(19) | C28 | C29 | C30 | 120.5(3) |
| C5 | C14 | C13 | 107.27(16) | C31 | C30 | C29 | 121.1(2) |
| C5 | C14 | C15 | 106.73(17) | C30 | C31 | C26 | 119.5(3) |
| C13 | C14 | C15 | 107.51(16) | | | | |

**Supplementary Table 14. Torsion Angles for TD39066_auto.**

| A | B | C | D | Angle/° | A | B | C | D | Angle/° |
|---|---|---|---|---|---|---|---|---|---|
| Cl1 | C1 | C2 | C3 | 177.37(18) | C11 | C12 | C13 | C14 | -179.3(2) |
| Cl1 | C1 | C6 | C5 | -175.18(14) | C12 | C13 | C14 | C5 | -127.1(2) |
| Cl1 | C1 | C6 | C7 | -0.5(3) | C12 | C13 | C14 | C15 | 118.5(2) |
| Cl2 | C9 | C10 | C11 | 179.90(18) | C13 | C8 | C9 | Cl2 | 179.51(14) |
| N1 | C23 | C24 | C25 | 1.0(3) | C13 | C8 | C9 | C10 | 0.3(3) |
| N1 | C27 | C28 | C29 | 179.4(2) | C13 | C14 | C15 | C16 | 52.8(2) |
| C1 | C2 | C3 | C4 | -1.4(4) | C14 | C5 | C6 | C1 | 175.98(17) |
| C1 | C6 | C7 | C8 | -120.4(2) | C14 | C5 | C6 | C7 | 0.6(2) |
| C1 | C6 | C7 | C16 | 127.0(2) | C14 | C15 | C16 | C7 | 7.2(2) |
| C2 | C1 | C6 | C5 | 2.4(3) | C14 | C15 | C16 | C17 | 132.54(17) |
| C2 | C1 | C6 | C7 | 177.07(19) | C14 | C15 | C16 | C23 | -108.79(18) |
| C2 | C3 | C4 | C5 | 0.9(4) | C15 | C16 | C17 | C18 | 54.9(3) |
| C3 | C4 | C5 | C6 | 1.3(3) | C15 | C16 | C17 | C22 | -126.9(2) |
| C3 | C4 | C5 | C14 | -177.4(2) | C15 | C16 | C23 | N1 | 3.0(2) |
| C4 | C5 | C6 | C1 | -2.9(3) | C15 | C16 | C23 | C24 | -177.89(17) |
| C4 | C5 | C6 | C7 | -178.30(18) | C16 | C7 | C8 | C9 | -116.9(2) |
| C4 | C5 | C14 | C13 | 123.1(2) | C16 | C7 | C8 | C13 | 60.5(2) |
| C4 | C5 | C14 | C15 | -121.9(2) | C16 | C17 | C18 | C19 | 177.9(3) |
| C5 | C6 | C7 | C8 | 54.5(2) | C16 | C17 | C22 | C21 | -178.3(2) |
| C5 | C6 | C7 | C16 | -58.2(2) | C16 | C23 | C24 | C25 | -178.07(19) |
| C5 | C14 | C15 | C16 | -62.04(19) | C17 | C16 | C23 | N1 | 123.77(19) |
| C6 | C1 | C2 | C3 | -0.3(3) | C17 | C16 | C23 | C24 | -57.1(2) |
| C6 | C5 | C14 | C13 | -55.7(2) | C17 | C18 | C19 | C20 | 0.5(5) |
| C6 | C5 | C14 | C15 | 59.2(2) | C18 | C17 | C22 | C21 | -0.2(4) |
| C6 | C7 | C8 | C9 | 126.9(2) | C18 | C19 | C20 | C21 | 0.1(5) |
| C6 | C7 | C8 | C13 | -55.7(2) | C19 | C20 | C21 | C22 | -0.8(5) |
| C6 | C7 | C16 | C15 | 50.89(19) | C20 | C21 | C22 | C17 | 0.8(4) |
| C6 | C7 | C16 | C17 | -71.6(2) | C22 | C17 | C18 | C19 | -0.4(4) |
| C6 | C7 | C16 | C23 | 171.31(16) | C23 | N1 | C27 | C26 | 0.3(3) |
| C7 | C8 | C9 | Cl2 | -3.2(3) | C23 | N1 | C27 | C28 | -179.94(19) |
| C7 | C8 | C9 | C10 | 177.63(19) | C23 | C16 | C17 | C18 | -67.5(3) |
| C7 | C8 | C13 | C12 | -177.08(18) | C23 | C16 | C17 | C22 | 110.7(2) |
| C7 | C8 | C13 | C14 | 1.5(2) | C23 | C24 | C25 | C26 | -1.3(3) |
| C7 | C16 | C17 | C18 | 175.8(2) | C24 | C25 | C26 | C27 | 1.1(3) |

| | | | | | | | | | |
|---|---|---|---|---|---|---|---|---|---|
| C7 | C16 | C17 | C22 | -6.0(3) | C24 | C25 | C26 | C31 | -179.1(2) |
| C7 | C16 | C23 | N1 | -113.71(19) | C25 | C26 | C27 | N1 | -0.6(3) |
| C7 | C16 | C23 | C24 | 65.4(2) | C25 | C26 | C27 | C28 | 179.7(2) |
| C8 | C7 | C16 | C15 | -62.67(18) | C25 | C26 | C31 | C30 | -178.8(2) |
| C8 | C7 | C16 | C17 | 174.81(17) | C26 | C27 | C28 | C29 | -0.9(3) |
| C8 | C7 | C16 | C23 | 57.8(2) | C27 | N1 | C23 | C16 | 178.54(16) |
| C8 | C9 | C10 | C11 | -0.9(3) | C27 | N1 | C23 | C24 | -0.5(3) |
| C8 | C13 | C14 | C5 | 54.4(2) | C27 | C26 | C31 | C30 | 1.1(4) |
| C8 | C13 | C14 | C15 | -60.0(2) | C27 | C28 | C29 | C30 | 1.1(4) |
| C9 | C8 | C13 | C12 | 0.6(3) | C28 | C29 | C30 | C31 | -0.2(4) |
| C9 | C8 | C13 | C14 | 179.19(17) | C29 | C30 | C31 | C26 | -0.9(4) |
| C9 | C10 | C11 | C12 | 0.6(4) | C31 | C26 | C27 | N1 | 179.5(2) |
| C10 | C11 | C12 | C13 | 0.3(4) | C31 | C26 | C27 | C28 | -0.2(3) |
| C11 | C12 | C13 | C8 | -0.9(3) | | | | | |

**Supplementary Table 15. Hydrogen Atom Coordinates (Å×$10^4$) and Isotropic Displacement Parameters (Å$^2$×$10^3$) for TD39066_auto.**

| Atom | *x* | *y* | *z* | U(eq) |
|---|---|---|---|---|
| H2 | -1213 | 6279 | 3741 | 81 |
| H3 | -3250 | 5428 | 4345 | 86 |
| H4 | -3163 | 5208 | 6215 | 74 |
| H7 | 2355 | 7271 | 7048 | 55 |
| H10 | 4528 | 4303 | 8916 | 72 |
| H11 | 2495 | 3429 | 9532 | 75 |
| H12 | 86 | 4082 | 9145 | 65 |
| H14 | -1631 | 5475 | 8203 | 60 |
| H15A | -372 | 7044 | 9305 | 62 |
| H15B | -1397 | 7462 | 8252 | 62 |
| H18 | -226 | 9248 | 9202 | 97 |
| H19 | -751 | 11005 | 8766 | 115 |
| H20 | -319 | 11659 | 7128 | 101 |
| H21 | 625 | 10559 | 5919 | 85 |
| H22 | 1206 | 8799 | 6353 | 71 |
| H24 | 3683 | 8730 | 8352 | 73 |
| H25 | 5772 | 8975 | 9766 | 79 |
| H28 | 2338 | 6824 | 12119 | 80 |
| H29 | 4377 | 7027 | 13583 | 94 |
| H30 | 6643 | 7970 | 13413 | 99 |
| H31 | 6919 | 8715 | 11783 | 91 |

(3) Absolute configurations of products **3s-3zc** are determined by *X*-ray structure analysis of product **3u**.

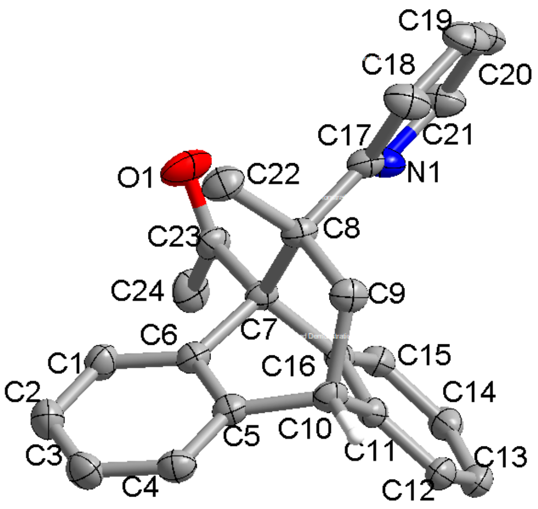


**Supplementary Figure 14.** Absolute configuration of **3u** (CCDC 2309906)

Displacement ellipsoids are drawn at the 30% probability level.

(Solvents: ethyl acetate/petroleum ether = 1:5)

**Supplementary Table 16. Crystal data and structure refinement for TD38050_auto.**

| | |
|---|---|
| Identification code | TD38050_auto |
| Empirical formula | $C_{24}H_{21}NO$ |
| Formula weight | 339.42 |
| Temperature/K | 293(2) |
| Crystal system | orthorhombic |
| Space group | $P2_12_12_1$ |
| a/Å | 8.32073(13) |
| b/Å | 8.78889(18) |
| c/Å | 24.6329(4) |
| α/° | 90 |
| β/° | 90 |
| γ/° | 90 |
| Volume/Å$^3$ | 1801.41(6) |
| Z | 4 |
| $\rho_{calc}$g/cm$^3$ | 1.252 |
| μ/mm$^{-1}$ | 0.588 |
| F(000) | 720.0 |
| Crystal size/mm$^3$ | 0.15 × 0.12 × 0.1 |
| Radiation | CuKα (λ = 1.54184) |
| 2Θ range for data collection/° | 7.178 to 141.042 |

| | |
|---|---|
| Index ranges | -10 ≤ h ≤ 9, -10 ≤ k ≤ 10, -30 ≤ l ≤ 30 |
| Reflections collected | 18087 |
| Independent reflections | 3432 [$R_{int}$ = 0.0424, $R_{sigma}$ = 0.0283] |
| Data/restraints/parameters | 3432/0/237 |
| Goodness-of-fit on $F^2$ | 1.045 |
| Final R indexes [I>=2σ (I)] | $R_1$ = 0.0448, $wR_2$ = 0.1182 |
| Final R indexes [all data] | $R_1$ = 0.0469, $wR_2$ = 0.1206 |
| Largest diff. peak/hole / e Å$^{-3}$ | 0.15/-0.29 |
| Flack parameter | 0.0(2) |

**Supplementary Table 17. Fractional Atomic Coordinates (×10$^4$) and Equivalent Isotropic Displacement Parameters (Å$^2$×10$^3$) for TD38050_auto. $U_{eq}$ is defined as 1/3 of of the trace of the orthogonalised $U_{IJ}$ tensor.**

| **Atom** | ***x*** | ***y*** | ***z*** | **U(eq)** |
|---|---|---|---|---|
| O1 | 9650(2) | 2891(4) | 3654.8(10) | 77.1(7) |
| N1 | 8538(3) | 6047(3) | 3614.1(8) | 55.0(5) |
| C1 | 6522(3) | 469(3) | 3947.7(11) | 51.2(5) |
| C2 | 5491(4) | -776(3) | 3916.3(13) | 60.8(7) |
| C3 | 3880(4) | -558(3) | 3813.1(12) | 58.4(6) |
| C4 | 3268(3) | 900(3) | 3740.9(10) | 50.3(5) |
| C5 | 4292(3) | 2136(3) | 3773.7(8) | 42.0(4) |
| C6 | 5929(3) | 1928(3) | 3880.7(8) | 41.5(5) |
| C7 | 6901(2) | 3416(2) | 3875.7(9) | 37.9(5) |
| C8 | 6648(3) | 4100(3) | 3283.9(8) | 41.8(5) |
| C9 | 4804(3) | 4458(3) | 3220.4(9) | 46.2(5) |
| C10 | 3828(2) | 3783(3) | 3699.5(10) | 42.8(5) |
| C11 | 4408(3) | 4625(2) | 4191.3(9) | 40.2(4) |
| C12 | 3459(3) | 5513(3) | 4526.8(10) | 47.4(5) |
| C13 | 4138(3) | 6278(3) | 4959.0(11) | 52.8(6) |
| C14 | 5780(3) | 6180(3) | 5041.2(10) | 50.3(5) |
| C15 | 6745(3) | 5308(2) | 4701.6(9) | 41.8(5) |
| C16 | 6068(2) | 4491(2) | 4280.9(8) | 36.8(4) |
| C17 | 7627(3) | 5559(3) | 3208.1(9) | 43.4(5) |
| C18 | 7568(3) | 6346(4) | 2717.8(12) | 61.7(7) |
| C19 | 8464(4) | 7653(4) | 2651.6(13) | 69.6(8) |
| C20 | 9398(4) | 8153(4) | 3074.6(13) | 65.6(7) |
| C21 | 9394(4) | 7326(4) | 3541.7(12) | 66.2(8) |
| C22 | 7101(3) | 2942(4) | 2835.2(10) | 57.4(6) |
| C23 | 8664(3) | 3039(3) | 4007.3(10) | 45.8(5) |
| C24 | 9133(3) | 2691(3) | 4586.8(12) | 58.2(6) |

**Supplementary Table 18. Anisotropic Displacement Parameters (Å$^2$×10$^3$) for TD38050_auto. The Anisotropic displacement factor exponent takes the form: -2π$^2$[h$^2$a*$^2$U$_{11}$+2hka*b*U$_{12}$+…].**

| Atom | $U_{11}$ | $U_{22}$ | $U_{33}$ | $U_{23}$ | $U_{13}$ | $U_{12}$ |
|---|---|---|---|---|---|---|
| O1 | 39.0(9) | 120(2) | 72.2(13) | 9.3(13) | 10.4(9) | 22.6(11) |
| N1 | 49.5(10) | 76.6(15) | 38.8(9) | 5.7(9) | 1.3(8) | -17.0(10) |
| C1 | 43.1(11) | 50.3(12) | 60.2(13) | -2.3(11) | 0.6(10) | 6.5(10) |
| C2 | 65.6(16) | 44.3(12) | 72.4(16) | -1.4(11) | 4.5(13) | 4.9(11) |
| C3 | 58.9(14) | 50.2(13) | 66.1(15) | -2.8(11) | 3.9(12) | -11.6(11) |
| C4 | 41.6(10) | 59.2(14) | 50.0(12) | -2.3(10) | 1.4(10) | -6.3(10) |
| C5 | 38.0(10) | 49.9(11) | 38.1(9) | -2.0(9) | 1.4(8) | 0.2(9) |
| C6 | 37.8(10) | 47.5(11) | 39.3(10) | -1.4(8) | 2.5(8) | 2.3(9) |
| C7 | 31.2(9) | 46.3(11) | 36.2(9) | 2.4(8) | -0.2(7) | 3.0(8) |
| C8 | 35.5(9) | 55.3(12) | 34.6(10) | 1.2(8) | 1.3(8) | 0.4(9) |
| C9 | 37.0(10) | 60.1(13) | 41.5(11) | 6.5(10) | -7.2(8) | -0.3(9) |
| C10 | 28.1(9) | 52.4(11) | 48.0(11) | 2.6(9) | -2.6(8) | 2.0(8) |
| C11 | 37.2(10) | 40.6(10) | 42.8(10) | 6.5(8) | 4.1(8) | 2.7(8) |
| C12 | 41.4(10) | 43.6(11) | 57.3(13) | 5.2(10) | 8.2(10) | 7.2(9) |
| C13 | 61.0(14) | 42.9(11) | 54.5(13) | -2.0(9) | 16.9(11) | 6.5(10) |
| C14 | 63.4(14) | 44.9(11) | 42.5(11) | -4.1(9) | 6.2(10) | -6.3(10) |
| C15 | 42.0(10) | 42.6(11) | 40.8(11) | 4.1(8) | 1.4(9) | -4.3(8) |
| C16 | 36.1(10) | 39.5(10) | 34.8(9) | 6.3(7) | 2.6(7) | 0.7(8) |
| C17 | 34.2(9) | 59.1(13) | 36.9(10) | 4.3(9) | 3.0(8) | 2.0(9) |
| C18 | 52.1(13) | 82.7(19) | 50.3(14) | 18.8(13) | -8.9(11) | -8.7(13) |
| C19 | 56.3(15) | 87(2) | 64.9(17) | 34.3(15) | 0.6(13) | -7.1(15) |
| C20 | 53.4(14) | 72.1(17) | 71.4(17) | 10.0(14) | 13.3(13) | -13.6(13) |
| C21 | 61.0(15) | 88(2) | 49.4(13) | -1.8(13) | 3.8(12) | -27.5(15) |
| C22 | 56.4(14) | 74.0(16) | 41.7(12) | -9.1(12) | 3.5(10) | 1.2(12) |
| C23 | 32.8(10) | 51.5(11) | 53.2(12) | 3.7(10) | -1.0(9) | 3.9(9) |
| C24 | 48.9(13) | 61.7(14) | 63.9(15) | 9.0(11) | -16.2(12) | 10.8(11) |

**Supplementary Table 19. Bond Lengths for TD38050_auto.**

| Atom | Atom | Length/Å | Atom | Atom | Length/Å |
|---|---|---|---|---|---|
| O1 | C23 | 1.202(3) | C8 | C17 | 1.531(3) |
| N1 | C17 | 1.326(3) | C8 | C22 | 1.549(3) |
| N1 | C21 | 1.342(4) | C9 | C10 | 1.550(3) |
| C1 | C2 | 1.393(4) | C10 | C11 | 1.499(3) |
| C1 | C6 | 1.384(3) | C11 | C12 | 1.384(3) |
| C2 | C3 | 1.378(4) | C11 | C16 | 1.404(3) |
| C3 | C4 | 1.390(4) | C12 | C13 | 1.380(4) |
| C4 | C5 | 1.383(3) | C13 | C14 | 1.384(4) |
| C5 | C6 | 1.399(3) | C14 | C15 | 1.390(3) |
| C5 | C10 | 1.509(3) | C15 | C16 | 1.381(3) |
| C6 | C7 | 1.538(3) | C17 | C18 | 1.393(3) |

| | | | | | |
|---|---|---|---|---|---|
| C7 | C8 | 1.591(3) | C18 | C19 | 1.379(4) |
| C7 | C16 | 1.539(3) | C19 | C20 | 1.372(5) |
| C7 | C23 | 1.538(3) | C20 | C21 | 1.361(4) |
| C8 | C9 | 1.574(3) | C23 | C24 | 1.511(4) |

**Supplementary Table 20. Bond Angles for TD38050_auto.**

| Atom | Atom | Atom | Angle/° | Atom | Atom | Atom | Angle/° |
|---|---|---|---|---|---|---|---|
| C17 | N1 | C21 | 118.3(2) | C5 | C10 | C9 | 108.95(19) |
| C6 | C1 | C2 | 120.1(2) | C11 | C10 | C5 | 107.05(17) |
| C3 | C2 | C1 | 120.0(2) | C11 | C10 | C9 | 104.94(17) |
| C2 | C3 | C4 | 120.6(2) | C12 | C11 | C10 | 125.3(2) |
| C5 | C4 | C3 | 119.4(2) | C12 | C11 | C16 | 121.0(2) |
| C4 | C5 | C6 | 120.5(2) | C16 | C11 | C10 | 113.72(18) |
| C4 | C5 | C10 | 126.1(2) | C13 | C12 | C11 | 120.1(2) |
| C6 | C5 | C10 | 113.40(19) | C12 | C13 | C14 | 119.1(2) |
| C1 | C6 | C5 | 119.4(2) | C13 | C14 | C15 | 121.1(2) |
| C1 | C6 | C7 | 126.94(19) | C16 | C15 | C14 | 120.2(2) |
| C5 | C6 | C7 | 113.57(19) | C11 | C16 | C7 | 113.12(18) |
| C6 | C7 | C8 | 105.02(17) | C15 | C16 | C7 | 128.46(19) |
| C6 | C7 | C16 | 106.23(16) | C15 | C16 | C11 | 118.42(19) |
| C6 | C7 | C23 | 108.49(18) | N1 | C17 | C8 | 118.9(2) |
| C16 | C7 | C8 | 107.63(16) | N1 | C17 | C18 | 120.9(2) |
| C23 | C7 | C8 | 113.61(18) | C18 | C17 | C8 | 120.2(2) |
| C23 | C7 | C16 | 115.14(19) | C19 | C18 | C17 | 119.8(3) |
| C9 | C8 | C7 | 107.18(16) | C20 | C19 | C18 | 118.9(3) |
| C17 | C8 | C7 | 110.98(18) | C21 | C20 | C19 | 118.0(3) |
| C17 | C8 | C9 | 109.83(19) | N1 | C21 | C20 | 124.1(3) |
| C17 | C8 | C22 | 109.52(19) | O1 | C23 | C7 | 121.5(2) |
| C22 | C8 | C7 | 111.9(2) | O1 | C23 | C24 | 119.0(2) |
| C22 | C8 | C9 | 107.29(19) | C24 | C23 | C7 | 119.3(2) |
| C10 | C9 | C8 | 111.02(17) | | | | |

**Supplementary Table 21. Torsion Angles for TD38050_auto.**

| A | B | C | D | Angle/° | A | B | C | D | Angle/° |
|---|---|---|---|---|---|---|---|---|---|
| N1 | C17 | C18 | C19 | -0.1(4) | C8 | C17 | C18 | C19 | -179.5(3) |
| C1 | C2 | C3 | C4 | -0.2(5) | C9 | C8 | C17 | N1 | 119.3(2) |
| C1 | C6 | C7 | C8 | 117.0(2) | C9 | C8 | C17 | C18 | -61.3(3) |
| C1 | C6 | C7 | C16 | -129.1(2) | C9 | C10 | C11 | C12 | 119.5(2) |
| C1 | C6 | C7 | C23 | -4.8(3) | C9 | C10 | C11 | C16 | -58.1(2) |
| C2 | C1 | C6 | C5 | -0.9(4) | C10 | C5 | C6 | C1 | -178.8(2) |
| C2 | C1 | C6 | C7 | -176.9(2) | C10 | C5 | C6 | C7 | -2.4(3) |
| C2 | C3 | C4 | C5 | 0.0(4) | C10 | C11 | C12 | C13 | -177.8(2) |
| C3 | C4 | C5 | C6 | -0.3(4) | C10 | C11 | C16 | C7 | -4.0(2) |

| | | | | | | | | | |
|---|---|---|---|---|---|---|---|---|---|
| C3 | C4 | C5 | C10 | 179.3(2) | C10 | C11 | C16 | C15 | 175.60(18) |
| C4 | C5 | C6 | C1 | 0.7(3) | C11 | C12 | C13 | C14 | 1.9(4) |
| C4 | C5 | C6 | C7 | 177.21(19) | C12 | C11 | C16 | C7 | 178.2(2) |
| C4 | C5 | C10 | C9 | -120.4(2) | C12 | C11 | C16 | C15 | -2.2(3) |
| C4 | C5 | C10 | C11 | 126.6(2) | C12 | C13 | C14 | C15 | -1.1(4) |
| C5 | C6 | C7 | C8 | -59.1(2) | C13 | C14 | C15 | C16 | -1.5(3) |
| C5 | C6 | C7 | C16 | 54.7(2) | C14 | C15 | C16 | C7 | -177.4(2) |
| C5 | C6 | C7 | C23 | 179.07(19) | C14 | C15 | C16 | C11 | 3.1(3) |
| C5 | C10 | C11 | C12 | -124.8(2) | C16 | C7 | C8 | C9 | -49.9(2) |
| C5 | C10 | C11 | C16 | 57.6(2) | C16 | C7 | C8 | C17 | 70.0(2) |
| C6 | C1 | C2 | C3 | 0.6(4) | C16 | C7 | C8 | C22 | -167.27(18) |
| C6 | C5 | C10 | C9 | 59.1(2) | C16 | C7 | C23 | O1 | -143.5(3) |
| C6 | C5 | C10 | C11 | -53.9(2) | C16 | C7 | C23 | C24 | 42.8(3) |
| C6 | C7 | C8 | C9 | 63.0(2) | C16 | C11 | C12 | C13 | -0.3(3) |
| C6 | C7 | C8 | C17 | -177.08(17) | C17 | N1 | C21 | C20 | -0.6(5) |
| C6 | C7 | C8 | C22 | -54.4(2) | C17 | C8 | C9 | C10 | -130.5(2) |
| C6 | C7 | C16 | C11 | -51.1(2) | C17 | C18 | C19 | C20 | -0.2(5) |
| C6 | C7 | C16 | C15 | 129.4(2) | C18 | C19 | C20 | C21 | 0.1(5) |
| C6 | C7 | C23 | O1 | 97.6(3) | C19 | C20 | C21 | N1 | 0.3(5) |
| C6 | C7 | C23 | C24 | -76.1(3) | C21 | N1 | C17 | C8 | 179.8(2) |
| C7 | C8 | C9 | C10 | -9.8(3) | C21 | N1 | C17 | C18 | 0.5(4) |
| C7 | C8 | C17 | N1 | 1.0(3) | C22 | C8 | C9 | C10 | 110.5(2) |
| C7 | C8 | C17 | C18 | -179.6(2) | C22 | C8 | C17 | N1 | -123.1(2) |
| C8 | C7 | C16 | C11 | 61.0(2) | C22 | C8 | C17 | C18 | 56.3(3) |
| C8 | C7 | C16 | C15 | -118.6(2) | C23 | C7 | C8 | C9 | -178.61(19) |
| C8 | C7 | C23 | O1 | -18.8(4) | C23 | C7 | C8 | C17 | -58.7(2) |
| C8 | C7 | C23 | C24 | 167.6(2) | C23 | C7 | C8 | C22 | 64.0(3) |
| C8 | C9 | C10 | C5 | -49.8(2) | C23 | C7 | C16 | C11 | -171.16(18) |
| C8 | C9 | C10 | C11 | 64.5(2) | C23 | C7 | C16 | C15 | 9.3(3) |

**Supplementary Table 22. Hydrogen Atom Coordinates (Å×$10^4$) and Isotropic Displacement Parameters (Å$^2$×$10^3$) for TD38050_auto.**

| Atom | *x* | *y* | *z* | U(eq) |
|---|---|---|---|---|
| H1 | 7611 | 320 | 4014 | 61 |
| H2 | 5890 | -1755 | 3965 | 73 |
| H3 | 3196 | -1393 | 3792 | 70 |
| H4 | 2180 | 1043 | 3671 | 60 |
| H9A | 4647 | 5551 | 3207 | 55 |
| H9B | 4416 | 4031 | 2882 | 55 |
| H10 | 2668 | 3904 | 3646 | 51 |
| H12 | 2362 | 5594 | 4461 | 57 |
| H13 | 3500 | 6851 | 5192 | 63 |
| H14 | 6246 | 6707 | 5328 | 60 |

| | | | | |
|---|---|---|---|---|
| H15 | 7850 | 5275 | 4758 | 50 |
| H18 | 6927 | 5992 | 2436 | 74 |
| H19 | 8436 | 8186 | 2326 | 83 |
| H20 | 10015 | 9031 | 3043 | 79 |
| H21 | 10023 | 7668 | 3829 | 79 |
| H22A | 6485 | 2028 | 2883 | 86 |
| H22B | 8226 | 2709 | 2859 | 86 |
| H22C | 6872 | 3373 | 2485 | 86 |
| H24A | 9743 | 1764 | 4597 | 87 |
| H24B | 8181 | 2577 | 4803 | 87 |
| H24C | 9772 | 3509 | 4728 | 87 |

(4) Absolute configurations of products **5a-5r**, **8**, **9** and **11** are determined by *X*-ray structure analysis of product **5k**.

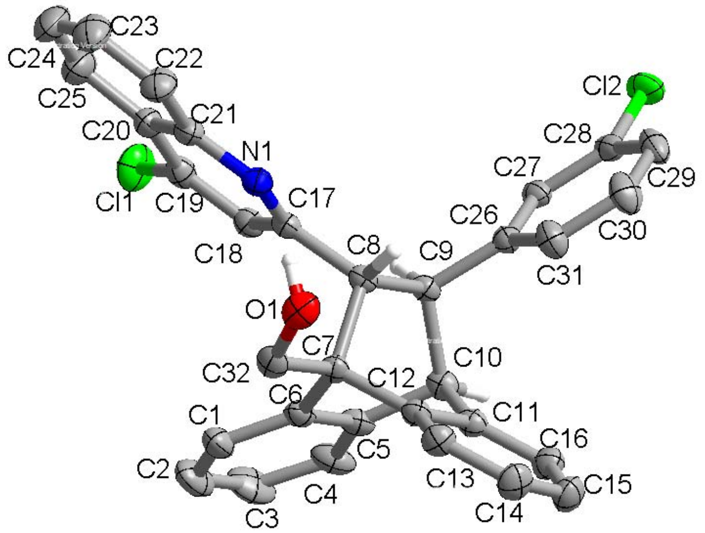


**Supplementary Figure 15.** Absolute configuration of **5k** (CCDC 2301851)

Displacement ellipsoids are drawn at the 30% probability level.

(Solvents: ethyl acetate/petroleum ether = 1:10)

**Supplementary Table 23. Crystal data and structure refinement for TD3908B.**

| | |
|---|---|
| Identification code | TD3908B |
| Empirical formula | $C_{32}H_{23}Cl_2NO$ |
| Formula weight | 508.41 |
| Temperature/K | 293(2) |
| Crystal system | orthorhombic |
| Space group | $P2_12_12$ |
| a/Å | 19.6917(2) |
| b/Å | 17.7545(2) |
| c/Å | 7.43769(9) |

| | |
|---|---|
| α/° | 90 |
| β/° | 90 |
| γ/° | 90 |
| Volume/Å$^3$ | 2600.34(6) |
| Z | 4 |
| $\rho_{calc}$g/cm$^3$ | 1.299 |
| μ/mm$^{-1}$ | 2.438 |
| F(000) | 1056.0 |
| Crystal size/mm$^3$ | 0.16 × 0.12 × 0.1 |
| Radiation | CuKα (λ = 1.54184) |
| 2Θ range for data collection/° | 6.704 to 140.548 |
| Index ranges | -19 ≤ h ≤ 23, -19 ≤ k ≤ 21, -8 ≤ l ≤ 8 |
| Reflections collected | 17126 |
| Independent reflections | 4898 [$R_{int}$ = 0.0376, $R_{sigma}$ = 0.0358] |
| Data/restraints/parameters | 4898/0/326 |
| Goodness-of-fit on F$^2$ | 1.037 |
| Final R indexes [I>=2σ (I)] | $R_1$ = 0.0465, w$R_2$ = 0.1256 |
| Final R indexes [all data] | $R_1$ = 0.0511, w$R_2$ = 0.1306 |
| Largest diff. peak/hole / e Å$^{-3}$ | 0.20/-0.26 |
| Flack parameter | -0.007(10) |

**Supplementary Table 24. Fractional Atomic Coordinates (×10$^4$) and Equivalent Isotropic Displacement Parameters (Å$^2$×10$^3$) for TD3908B. $U_{eq}$ is defined as 1/3 of of the trace of the orthogonalised $U_{IJ}$ tensor.**

| **Atom** | ***x*** | ***y*** | ***z*** | **U(eq)** |
|---|---|---|---|---|
| Cl1 | 4045.3(7) | 6484.2(7) | 10595.8(16) | 75.0(4) |
| Cl2 | 4756.9(5) | 11003.0(7) | 12150.0(16) | 66.5(3) |
| O1 | 3537.4(15) | 8396.8(17) | 2444(3) | 55.6(7) |
| N1 | 4352.2(14) | 7831.8(16) | 5514(4) | 40.2(6) |
| C1 | 2117(2) | 7481(2) | 6412(7) | 56.8(10) |
| C2 | 1717(3) | 7206(3) | 7786(9) | 78.9(15) |
| C3 | 1564(3) | 7649(3) | 9243(8) | 81.0(16) |
| C4 | 1804(2) | 8380(3) | 9352(6) | 60.3(11) |
| C5 | 2216.2(16) | 8655(2) | 8000(5) | 42.9(7) |
| C6 | 2375.7(16) | 8207.2(19) | 6518(5) | 41.3(7) |
| C7 | 2858.4(16) | 8581.7(18) | 5191(4) | 36.8(7) |
| C8 | 3526.7(15) | 8764.0(17) | 6323(4) | 33.1(6) |
| C9 | 3342.8(15) | 9252.6(17) | 7989(4) | 34.5(6) |
| C10 | 2555.9(17) | 9419.3(19) | 7970(4) | 39.5(7) |
| C11 | 2386.3(17) | 9783.3(19) | 6202(5) | 39.4(7) |
| C12 | 2560.5(16) | 9347.0(18) | 4717(4) | 37.5(7) |
| C13 | 2470.0(19) | 9638(2) | 3005(5) | 45.7(8) |
| C14 | 2170(2) | 10343(2) | 2794(6) | 56.4(10) |
| C15 | 1977(2) | 10758(2) | 4263(7) | 57.9(10) |

| | | | | |
|---|---|---|---|---|
| C16 | 2094(2) | 10487(2) | 5981(6) | 49.9(8) |
| C17 | 3911.7(15) | 8041.9(17) | 6750(4) | 34.9(6) |
| C18 | 3799.5(17) | 7625.0(19) | 8338(5) | 40.9(7) |
| C19 | 4173(2) | 6992(2) | 8622(5) | 46.1(8) |
| C20 | 4663.4(17) | 6744(2) | 7374(6) | 47.1(8) |
| C21 | 4722.7(17) | 7190.9(19) | 5799(5) | 44.4(7) |
| C22 | 5196(2) | 6972(3) | 4469(7) | 61.2(11) |
| C23 | 5586(2) | 6349(3) | 4689(9) | 76.4(15) |
| C24 | 5530(3) | 5912(3) | 6244(9) | 82.9(17) |
| C25 | 5084(2) | 6100(3) | 7565(8) | 68.3(12) |
| C26 | 3763.0(16) | 9968.0(18) | 8165(5) | 37.5(7) |
| C27 | 4023.9(16) | 10151.3(19) | 9841(5) | 40.0(7) |
| C28 | 4414.4(17) | 10792(2) | 10035(5) | 46.5(8) |
| C29 | 4548(2) | 11264(2) | 8621(6) | 58.3(11) |
| C30 | 4276(2) | 11090(2) | 6966(6) | 61.6(11) |
| C31 | 3888(2) | 10447(2) | 6726(5) | 50.7(9) |
| C32 | 3019(2) | 8095(2) | 3558(5) | 47.1(8) |

**Supplementary Table 25. Anisotropic Displacement Parameters (Å$^2$×10$^3$) for TD3908B. The Anisotropic displacement factor exponent takes the form: -2π$^2$[h$^2$a*$^2$U$_{11}$+2hka*b*U$_{12}$+…].**

| Atom | $U_{11}$ | $U_{22}$ | $U_{33}$ | $U_{23}$ | $U_{13}$ | $U_{12}$ |
|---|---|---|---|---|---|---|
| Cl1 | 102.8(9) | 62.7(6) | 59.4(6) | 25.8(5) | 3.4(6) | 5.4(6) |
| Cl2 | 51.2(5) | 76.2(6) | 72.0(7) | -36.2(6) | -17.0(4) | 3.7(5) |
| O1 | 65.0(16) | 67.2(17) | 34.6(13) | -4.2(11) | 2.1(11) | 10.9(14) |
| N1 | 36.9(13) | 43.7(14) | 39.9(14) | 0.5(12) | 2.4(11) | -1.7(11) |
| C1 | 47(2) | 48(2) | 76(3) | 3.9(19) | -10.7(19) | -11.2(16) |
| C2 | 65(3) | 65(3) | 108(4) | 25(3) | -9(3) | -30(2) |
| C3 | 61(3) | 101(4) | 81(4) | 34(3) | 4(3) | -36(3) |
| C4 | 44.9(19) | 89(3) | 47(2) | 10(2) | -0.7(16) | -20(2) |
| C5 | 33.1(14) | 57(2) | 38.8(17) | 6.6(15) | -4.6(13) | -6.0(13) |
| C6 | 34.3(15) | 44.2(17) | 45.6(18) | 5.9(14) | -9.2(13) | -4.2(13) |
| C7 | 39.8(16) | 38.5(16) | 32.0(15) | -2.2(13) | -6.7(13) | -0.4(12) |
| C8 | 33.8(14) | 36.9(14) | 28.6(14) | 2.8(11) | -0.4(11) | -4.5(12) |
| C9 | 36.2(14) | 38.8(15) | 28.5(14) | 0.5(12) | -2.3(12) | -3.4(12) |
| C10 | 40.1(16) | 48.0(17) | 30.5(15) | -3.3(13) | 3.3(13) | -3.1(14) |
| C11 | 37.1(15) | 43.2(16) | 37.8(17) | -1.6(14) | 1.1(13) | -1.9(13) |
| C12 | 35.5(15) | 41.3(16) | 35.8(16) | -0.6(13) | -4.8(13) | -1.4(13) |
| C13 | 50.6(18) | 51.8(19) | 34.8(17) | 1.8(15) | -2.7(15) | 1.6(15) |
| C14 | 68(2) | 53(2) | 48(2) | 13.8(17) | -9.9(18) | 5.4(18) |
| C15 | 65(2) | 43.4(18) | 65(3) | 5.3(18) | -9(2) | 10.1(17) |
| C16 | 48.6(19) | 49.9(19) | 51(2) | -7.4(17) | -1.0(17) | 6.6(15) |
| C17 | 32.1(13) | 38.4(15) | 34.3(15) | -2.0(12) | -1.8(12) | -5.7(12) |

| | | | | | | |
|---|---|---|---|---|---|---|
| C18 | 42.0(17) | 43.6(16) | 37.1(17) | -1.3(13) | -0.4(13) | -5.0(13) |
| C19 | 52.2(19) | 42.3(16) | 43.7(19) | 4.9(14) | -7.0(15) | -8.0(15) |
| C20 | 40.6(16) | 40.3(16) | 61(2) | 4.0(15) | -7.1(16) | -3.6(14) |
| C21 | 34.9(15) | 43.4(16) | 55(2) | -4.6(15) | -0.3(15) | -3.5(13) |
| C22 | 48(2) | 61(2) | 75(3) | -3(2) | 19(2) | 4.4(18) |
| C23 | 54(2) | 67(3) | 107(4) | -5(3) | 25(3) | 8(2) |
| C24 | 60(3) | 60(3) | 129(5) | 6(3) | 6(3) | 21(2) |
| C25 | 59(2) | 54(2) | 92(3) | 11(2) | -4(2) | 10.6(19) |
| C26 | 35.0(14) | 38.1(15) | 39.4(17) | -1.7(13) | 2.3(13) | 0.4(12) |
| C27 | 33.2(15) | 42.6(16) | 44.2(18) | -8.6(13) | -0.3(13) | 2.7(13) |
| C28 | 32.8(15) | 49.5(18) | 57(2) | -22.3(17) | -1.0(14) | 4.1(13) |
| C29 | 50(2) | 49(2) | 75(3) | -22(2) | 17.6(19) | -16.5(16) |
| C30 | 72(3) | 54(2) | 58(2) | -1.0(19) | 17(2) | -19(2) |
| C31 | 62(2) | 48.9(19) | 41.3(19) | -3.3(15) | 5.4(16) | -12.4(17) |
| C32 | 54(2) | 47.9(17) | 39.0(18) | -7.1(15) | -10.2(15) | 1.0(16) |

**Supplementary Table 26. Bond Lengths for TD3908B.**

| Atom | Atom | Length/Å | Atom | Atom | Length/Å |
|---|---|---|---|---|---|
| Cl1 | C19 | 1.742(4) | C11 | C12 | 1.392(5) |
| Cl2 | C28 | 1.752(4) | C11 | C16 | 1.385(5) |
| O1 | C32 | 1.420(5) | C12 | C13 | 1.385(5) |
| N1 | C17 | 1.318(4) | C13 | C14 | 1.394(5) |
| N1 | C21 | 1.368(5) | C14 | C15 | 1.371(6) |
| C1 | C2 | 1.380(7) | C15 | C16 | 1.385(6) |
| C1 | C6 | 1.389(5) | C17 | C18 | 1.411(5) |
| C2 | C3 | 1.373(9) | C18 | C19 | 1.359(5) |
| C3 | C4 | 1.383(7) | C19 | C20 | 1.410(6) |
| C4 | C5 | 1.381(5) | C20 | C21 | 1.419(5) |
| C5 | C6 | 1.395(5) | C20 | C25 | 1.419(5) |
| C5 | C10 | 1.513(5) | C21 | C22 | 1.413(6) |
| C6 | C7 | 1.523(5) | C22 | C23 | 1.357(6) |
| C7 | C8 | 1.595(4) | C23 | C24 | 1.397(8) |
| C7 | C12 | 1.522(4) | C24 | C25 | 1.360(8) |
| C7 | C32 | 1.524(5) | C26 | C27 | 1.387(5) |
| C8 | C9 | 1.555(4) | C26 | C31 | 1.389(5) |
| C8 | C17 | 1.523(4) | C27 | C28 | 1.380(5) |
| C9 | C10 | 1.578(4) | C28 | C29 | 1.371(6) |
| C9 | C26 | 1.522(4) | C29 | C30 | 1.377(7) |
| C10 | C11 | 1.503(5) | C30 | C31 | 1.385(5) |

**Supplementary Table 27. Bond Angles for TD3908B.**

| Atom | Atom | Atom | Angle/° | Atom | Atom | Atom | Angle/° |
|---|---|---|---|---|---|---|---|
| C17 | N1 | C21 | 118.5(3) | C12 | C13 | C14 | 119.5(3) |
| C2 | C1 | C6 | 119.7(5) | C15 | C14 | C13 | 120.7(4) |
| C3 | C2 | C1 | 120.4(4) | C14 | C15 | C16 | 120.1(3) |
| C2 | C3 | C4 | 120.6(4) | C15 | C16 | C11 | 119.5(4) |
| C5 | C4 | C3 | 119.3(5) | N1 | C17 | C8 | 114.8(3) |
| C4 | C5 | C6 | 120.4(4) | N1 | C17 | C18 | 122.6(3) |
| C4 | C5 | C10 | 126.0(4) | C18 | C17 | C8 | 122.6(3) |
| C6 | C5 | C10 | 113.6(3) | C19 | C18 | C17 | 118.6(3) |
| C1 | C6 | C5 | 119.4(4) | C18 | C19 | Cl1 | 118.7(3) |
| C1 | C6 | C7 | 126.7(4) | C18 | C19 | C20 | 121.8(3) |
| C5 | C6 | C7 | 113.8(3) | C20 | C19 | Cl1 | 119.5(3) |
| C6 | C7 | C8 | 105.2(2) | C19 | C20 | C21 | 115.2(3) |
| C6 | C7 | C32 | 113.5(3) | C19 | C20 | C25 | 125.8(4) |
| C12 | C7 | C6 | 107.4(3) | C25 | C20 | C21 | 119.0(4) |
| C12 | C7 | C8 | 105.0(2) | N1 | C21 | C20 | 123.3(3) |
| C12 | C7 | C32 | 113.7(3) | N1 | C21 | C22 | 118.1(4) |
| C32 | C7 | C8 | 111.4(3) | C22 | C21 | C20 | 118.6(4) |
| C9 | C8 | C7 | 110.0(2) | C23 | C22 | C21 | 120.9(4) |
| C17 | C8 | C7 | 110.5(2) | C22 | C23 | C24 | 120.4(4) |
| C17 | C8 | C9 | 114.8(3) | C25 | C24 | C23 | 120.9(4) |
| C8 | C9 | C10 | 109.0(2) | C24 | C25 | C20 | 120.2(5) |
| C26 | C9 | C8 | 114.1(3) | C27 | C26 | C9 | 118.4(3) |
| C26 | C9 | C10 | 112.2(3) | C27 | C26 | C31 | 118.9(3) |
| C5 | C10 | C9 | 105.4(3) | C31 | C26 | C9 | 122.7(3) |
| C11 | C10 | C5 | 107.5(3) | C28 | C27 | C26 | 119.6(3) |
| C11 | C10 | C9 | 107.9(3) | C27 | C28 | Cl2 | 119.0(3) |
| C12 | C11 | C10 | 113.6(3) | C29 | C28 | Cl2 | 119.0(3) |
| C16 | C11 | C10 | 125.7(3) | C29 | C28 | C27 | 122.0(3) |
| C16 | C11 | C12 | 120.7(3) | C28 | C29 | C30 | 118.3(3) |
| C11 | C12 | C7 | 114.1(3) | C29 | C30 | C31 | 121.0(4) |
| C13 | C12 | C7 | 126.5(3) | C30 | C31 | C26 | 120.2(4) |
| C13 | C12 | C11 | 119.4(3) | O1 | C32 | C7 | 113.6(3) |

**Supplementary Table 28. Hydrogen Bonds for TD3908B.**

| D | H | A | d(D-H)/Å | d(H-A)/Å | d(D-A)/Å | D-H-A/° |
|---|---|---|---|---|---|---|
| O1 | H1 | N1 | 0.82 | 2.31 | 2.965(4) | 137.1 |

**Supplementary Table 29. Torsion Angles for TD3908B.**

| A | B | C | D | Angle/° | A | B | C | D | Angle/° |
|---|---|---|---|---|---|---|---|---|---|
| Cl1 | C19 | C20 | C21 | 179.2(3) | C10 | C5 | C6 | C1 | 177.6(3) |
| Cl1 | C19 | C20 | C25 | -0.3(5) | C10 | C5 | C6 | C7 | 0.2(4) |

| | | | | | | | | | |
|---|---|---|---|---|---|---|---|---|---|
| Cl2 | C28 | C29 | C30 | 179.9(3) | C10 | C9 | C26 | C27 | 100.5(3) |
| N1 | C17 | C18 | C19 | 1.7(5) | C10 | C9 | C26 | C31 | -78.7(4) |
| N1 | C21 | C22 | C23 | 178.9(4) | C10 | C11 | C12 | C7 | 2.4(4) |
| C1 | C2 | C3 | C4 | 0.6(8) | C10 | C11 | C12 | C13 | -176.0(3) |
| C1 | C6 | C7 | C8 | -118.1(4) | C10 | C11 | C16 | C15 | 178.8(4) |
| C1 | C6 | C7 | C12 | 130.4(4) | C11 | C12 | C13 | C14 | -3.7(5) |
| C1 | C6 | C7 | C32 | 3.9(5) | C12 | C7 | C8 | C9 | 57.2(3) |
| C2 | C1 | C6 | C5 | -1.2(6) | C12 | C7 | C8 | C17 | -175.1(3) |
| C2 | C1 | C6 | C7 | 176.0(4) | C12 | C7 | C32 | O1 | 64.9(4) |
| C2 | C3 | C4 | C5 | -1.9(7) | C12 | C11 | C16 | C15 | -0.2(6) |
| C3 | C4 | C5 | C6 | 1.6(6) | C12 | C13 | C14 | C15 | 1.3(6) |
| C3 | C4 | C5 | C10 | -175.9(4) | C13 | C14 | C15 | C16 | 1.7(7) |
| C4 | C5 | C6 | C1 | -0.1(5) | C14 | C15 | C16 | C11 | -2.2(6) |
| C4 | C5 | C6 | C7 | -177.6(3) | C16 | C11 | C12 | C7 | -178.5(3) |
| C4 | C5 | C10 | C9 | 116.5(4) | C16 | C11 | C12 | C13 | 3.1(5) |
| C4 | C5 | C10 | C11 | -128.6(4) | C17 | N1 | C21 | C20 | -0.9(5) |
| C5 | C6 | C7 | C8 | 59.2(3) | C17 | N1 | C21 | C22 | -179.7(3) |
| C5 | C6 | C7 | C12 | -52.3(3) | C17 | C8 | C9 | C10 | -126.6(3) |
| C5 | C6 | C7 | C32 | -178.8(3) | C17 | C8 | C9 | C26 | 107.1(3) |
| C5 | C10 | C11 | C12 | -55.2(4) | C17 | C18 | C19 | Cl1 | 179.0(2) |
| C5 | C10 | C11 | C16 | 125.7(4) | C17 | C18 | C19 | C20 | -0.2(5) |
| C6 | C1 | C2 | C3 | 0.9(7) | C18 | C19 | C20 | C21 | -1.5(5) |
| C6 | C5 | C10 | C9 | -61.1(3) | C18 | C19 | C20 | C25 | 179.0(4) |
| C6 | C5 | C10 | C11 | 53.8(4) | C19 | C20 | C21 | N1 | 2.1(5) |
| C6 | C7 | C8 | C9 | -56.0(3) | C19 | C20 | C21 | C22 | -179.0(4) |
| C6 | C7 | C8 | C17 | 71.7(3) | C19 | C20 | C25 | C24 | 178.7(5) |
| C6 | C7 | C12 | C11 | 51.1(4) | C20 | C21 | C22 | C23 | 0.0(6) |
| C6 | C7 | C12 | C13 | -130.7(4) | C21 | N1 | C17 | C8 | 179.6(3) |
| C6 | C7 | C32 | O1 | -171.9(3) | C21 | N1 | C17 | C18 | -1.1(5) |
| C7 | C8 | C9 | C10 | -1.3(3) | C21 | C20 | C25 | C24 | -0.7(7) |
| C7 | C8 | C9 | C26 | -127.7(3) | C21 | C22 | C23 | C24 | -0.3(8) |
| C7 | C8 | C17 | N1 | 88.0(3) | C22 | C23 | C24 | C25 | 0.1(9) |
| C7 | C8 | C17 | C18 | -91.3(3) | C23 | C24 | C25 | C20 | 0.4(8) |
| C7 | C12 | C13 | C14 | 178.2(3) | C25 | C20 | C21 | N1 | -178.3(4) |
| C8 | C7 | C12 | C11 | -60.5(4) | C25 | C20 | C21 | C22 | 0.5(5) |
| C8 | C7 | C12 | C13 | 117.7(4) | C26 | C9 | C10 | C5 | -173.8(3) |
| C8 | C7 | C32 | O1 | -53.5(4) | C26 | C9 | C10 | C11 | 71.5(3) |
| C8 | C9 | C10 | C5 | 58.8(3) | C26 | C27 | C28 | Cl2 | -178.5(2) |
| C8 | C9 | C10 | C11 | -55.8(3) | C26 | C27 | C28 | C29 | 0.9(5) |
| C8 | C9 | C26 | C27 | -134.9(3) | C27 | C26 | C31 | C30 | 0.9(6) |
| C8 | C9 | C26 | C31 | 45.9(4) | C27 | C28 | C29 | C30 | 0.5(6) |
| C8 | C17 | C18 | C19 | -179.1(3) | C28 | C29 | C30 | C31 | -1.2(6) |
| C9 | C8 | C17 | N1 | -147.0(3) | C29 | C30 | C31 | C26 | 0.5(7) |
| C9 | C8 | C17 | C18 | 33.8(4) | C31 | C26 | C27 | C28 | -1.6(5) |

| | | | | | | | | | |
|---|---|---|---|---|---|---|---|---|---|
| C9 | C10 | C11 | C12 | 58.0(4) | C32 | C7 | C8 | C9 | -179.3(3) |
| C9 | C10 | C11 | C16 | -121.1(4) | C32 | C7 | C8 | C17 | -51.6(3) |
| C9 | C26 | C27 | C28 | 179.2(3) | C32 | C7 | C12 | C11 | 177.5(3) |
| C9 | C26 | C31 | C30 | -179.9(4) | C32 | C7 | C12 | C13 | -4.3(5) |

**Supplementary Table 30. Hydrogen Atom Coordinates (Å×$10^4$) and Isotropic Displacement Parameters (Å$^2$×$10^3$) for TD3908B.**

| **Atom** | ***x*** | ***y*** | ***z*** | **U(eq)** |
|---|---|---|---|---|
| H1 | 3910 | 8268 | 2830 | 83 |
| H1A | 2214 | 7181 | 5420 | 68 |
| H2 | 1549 | 6717 | 7724 | 95 |
| H3 | 1297 | 7457 | 10166 | 97 |
| H4 | 1688 | 8684 | 10325 | 72 |
| H8 | 3822 | 9070 | 5553 | 40 |
| H9 | 3435 | 8946 | 9057 | 41 |
| H10 | 2417 | 9732 | 8992 | 47 |
| H13 | 2608 | 9364 | 2004 | 55 |
| H14 | 2101 | 10535 | 1645 | 68 |
| H15 | 1767 | 11222 | 4105 | 69 |
| H16 | 1978 | 10775 | 6979 | 60 |
| H18 | 3477 | 7780 | 9172 | 49 |
| H22 | 5240 | 7259 | 3430 | 73 |
| H23 | 5894 | 6211 | 3797 | 92 |
| H24 | 5801 | 5487 | 6378 | 100 |
| H25 | 5054 | 5806 | 8596 | 82 |
| H27 | 3936 | 9845 | 10828 | 48 |
| H29 | 4815 | 11691 | 8774 | 70 |
| H30 | 4353 | 11409 | 5996 | 74 |
| H31 | 3711 | 10335 | 5598 | 61 |
| H32A | 3157 | 7599 | 3966 | 56 |
| H32B | 2609 | 8035 | 2850 | 56 |

(5) Absolute configurations of products **7a-7l** are determined by *X*-ray structure analysis of product **7g**.

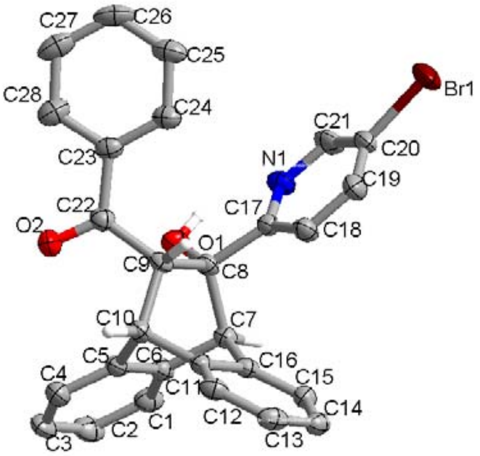


**Supplementary Figure 16.** Absolute configuration of **7g** (CCDC 2309908)

Displacement ellipsoids are drawn at the 30% probability level.

(Solvents: ethyl acetate/petroleum ether = 1:10)

**Supplementary Table 31. Crystal data and structure refinement for SX2502_auto.**

| | |
|---|---|
| Identification code | SX2502_auto |
| Empirical formula | $C_{28}H_{20}BrNO_2$ |
| Formula weight | 482.36 |
| Temperature/K | 293(2) |
| Crystal system | monoclinic |
| Space group | $P2_1$ |
| a/Å | 9.51661(10) |
| b/Å | 26.0392(3) |
| c/Å | 9.65233(11) |
| α/° | 90 |
| β/° | 111.1641(13) |
| γ/° | 90 |
| Volume/Å$^3$ | 2230.57(5) |
| Z | 4 |
| $\rho_{calc}$g/cm$^3$ | 1.436 |
| μ/mm$^{-1}$ | 2.710 |
| F(000) | 984.0 |
| Crystal size/mm$^3$ | 0.15 × 0.1 × 0.08 |
| Radiation | CuKα (λ = 1.54184) |
| 2Θ range for data collection/° | 6.79 to 140.806 |
| Index ranges | $-9 \le h \le 11$, $-31 \le k \le 31$, $-11 \le l \le 10$ |
| Reflections collected | 15852 |

| | |
|---|---|
| Independent reflections | 8286 [$R_{int}$ = 0.0318, $R_{sigma}$ = 0.0453] |
| Data/restraints/parameters | 8286/2/582 |
| Goodness-of-fit on $F^2$ | 1.038 |
| Final R indexes [I>=2σ (I)] | $R_1$ = 0.0483, $wR_2$ = 0.1274 |
| Final R indexes [all data] | $R_1$ = 0.0537, $wR_2$ = 0.1347 |
| Largest diff. peak/hole / e Å$^{-3}$ | 0.62/-0.58 |
| Flack parameter | -0.008(10) |

**Supplementary Table 32. Fractional Atomic Coordinates (×10$^4$) and Equivalent Isotropic Displacement Parameters (Å$^2$×10$^3$) for SX2502_auto. $U_{eq}$ is defined as 1/3 of of the trace of the orthogonalised $U_{IJ}$ tensor.**

| Atom | *x* | *y* | *z* | U(eq) |
|---|---|---|---|---|
| Br1 | 12761.6(11) | 2889.6(4) | 13030.3(9) | 91.5(3) |
| O1 | 10136(4) | 3202.6(18) | 5739(5) | 50.8(9) |
| O2 | 8941(6) | 4390(2) | 4526(6) | 70.6(13) |
| N1 | 11237(5) | 2927(2) | 8479(6) | 54.8(11) |
| C1 | 7147(7) | 2705(3) | 2883(7) | 54.9(14) |
| C2 | 6581(8) | 2889(4) | 1418(7) | 66.8(18) |
| C3 | 6091(8) | 3390(3) | 1137(7) | 67.4(19) |
| C4 | 6164(7) | 3716(3) | 2316(7) | 54.7(14) |
| C5 | 6711(6) | 3531(2) | 3744(6) | 44.0(11) |
| C6 | 7192(6) | 3024(2) | 4020(6) | 42.9(11) |
| C7 | 7721(5) | 2887(2) | 5668(5) | 40.0(10) |
| C8 | 9088(5) | 3254(2) | 6458(6) | 40.3(10) |
| C9 | 8476(5) | 3824(2) | 6246(5) | 38.4(10) |
| C10 | 6817(6) | 3827(2) | 5136(6) | 41.2(10) |
| C11 | 5954(6) | 3520(2) | 5898(6) | 40.8(10) |
| C12 | 4793(6) | 3703(2) | 6310(7) | 49.7(13) |
| C13 | 4102(7) | 3365(3) | 6977(7) | 57.2(15) |
| C14 | 4545(7) | 2863(3) | 7235(6) | 52.9(13) |
| C15 | 5737(6) | 2683(2) | 6839(6) | 46.6(11) |
| C16 | 6434(5) | 3011(2) | 6169(5) | 42.2(11) |
| C17 | 9851(6) | 3119(2) | 8112(6) | 42.1(11) |
| C18 | 9191(7) | 3198(3) | 9160(7) | 53.0(13) |
| C19 | 10025(8) | 3117(3) | 10641(7) | 59.4(15) |
| C20 | 11498(7) | 2941(3) | 11001(7) | 58.3(16) |
| C21 | 12043(7) | 2844(3) | 9911(8) | 63.8(17) |
| C22 | 9451(7) | 4202(2) | 5754(6) | 46.8(12) |
| C23 | 10943(6) | 4374(2) | 6824(7) | 47.5(12) |
| C24 | 11527(7) | 4219(2) | 8296(8) | 54.9(14) |
| C25 | 12915(8) | 4398(3) | 9225(9) | 70.4(19) |
| C26 | 13708(8) | 4745(4) | 8719(11) | 80(3) |
| C27 | 13120(9) | 4910(3) | 7256(12) | 80(2) |

| | | | | |
|---|---|---|---|---|
| C28 | 11753(8) | 4728(3) | 6313(9) | 62.4(16) |
| Br1' | 14933.8(8) | 6536.9(4) | 10893.6(11) | 89.4(3) |
| O1' | 7590(5) | 6088.7(17) | 8287(5) | 48.6(9) |
| O2' | 6434(5) | 4905.1(19) | 6890(6) | 63.9(12) |
| N1' | 10315(6) | 6438(2) | 9349(6) | 56.7(13) |
| C1' | 4623(6) | 6563(2) | 5367(6) | 49.2(12) |
| C2' | 3183(6) | 6350(3) | 4767(8) | 58.8(16) |
| C3' | 2986(6) | 5857(3) | 4211(8) | 61.2(16) |
| C4' | 4225(7) | 5564(3) | 4249(7) | 53.0(13) |
| C5' | 5649(6) | 5778(2) | 4824(6) | 44.1(11) |
| C6' | 5841(6) | 6276(2) | 5373(6) | 42.7(11) |
| C7' | 7484(6) | 6447.1(19) | 5942(6) | 40.9(10) |
| C8' | 8329(5) | 6072(2) | 7255(6) | 39.2(10) |
| C9' | 8175(5) | 5509(2) | 6601(6) | 39.5(10) |
| C10' | 7105(6) | 5522(2) | 4927(6) | 42.7(11) |
| C11' | 7858(6) | 5870(2) | 4142(6) | 44.0(11) |
| C12' | 8340(7) | 5726(3) | 3011(7) | 55.0(14) |
| C13' | 8951(7) | 6097(4) | 2366(7) | 66.9(19) |
| C14' | 9102(7) | 6588(4) | 2865(8) | 66.3(19) |
| C15' | 8660(6) | 6739(3) | 4048(7) | 54.6(14) |
| C16' | 8035(5) | 6368(2) | 4680(6) | 42.1(11) |
| C17' | 9986(6) | 6235.8(19) | 8012(6) | 40.5(10) |
| C18' | 11061(6) | 6151(2) | 7387(6) | 47.5(12) |
| C19' | 12563(7) | 6254(3) | 8234(7) | 52.8(13) |
| C20' | 12887(6) | 6442(2) | 9640(7) | 54.3(14) |
| C21' | 11764(7) | 6536(3) | 10164(7) | 65.9(17) |
| C22' | 7642(7) | 5120(2) | 7476(7) | 45.8(11) |
| C23' | 8653(7) | 4962(2) | 9003(7) | 48.8(12) |
| C24' | 10126(7) | 5132(2) | 9661(7) | 54.9(14) |
| C25' | 11007(9) | 4961(3) | 11069(8) | 69.5(19) |
| C26' | 10426(11) | 4618(4) | 11814(8) | 78(2) |
| C27' | 8989(12) | 4438(4) | 11159(9) | 80(2) |
| C28' | 8089(9) | 4611(3) | 9753(8) | 62.5(16) |

**Supplementary Table 33. Anisotropic Displacement Parameters (Å$^2$×10$^3$) for SX2502_auto. The Anisotropic displacement factor exponent takes the form: -2π$^2$[h$^2$a*$^2$U$_{11}$+2hka*b*U$_{12}$+…].**

| Atom | $U_{11}$ | $U_{22}$ | $U_{33}$ | $U_{23}$ | $U_{13}$ | $U_{12}$ |
|---|---|---|---|---|---|---|
| Br1 | 89.9(6) | 91.4(6) | 53.9(4) | 18.1(4) | -21.3(4) | -12.8(5) |
| O1 | 40.1(19) | 64(2) | 52(2) | -2.7(19) | 20.7(17) | 3.8(17) |
| O2 | 67(3) | 84(3) | 52(3) | 20(2) | 11(2) | -16(3) |
| N1 | 42(2) | 56(3) | 57(3) | 2(2) | 8(2) | 10(2) |
| C1 | 52(3) | 63(3) | 52(3) | -13(3) | 21(3) | -8(3) |

| | | | | | | |
|---|---|---|---|---|---|---|
| C2 | 65(4) | 95(5) | 42(3) | -20(4) | 20(3) | -15(4) |
| C3 | 64(4) | 93(5) | 34(3) | -1(3) | 4(3) | -19(4) |
| C4 | 50(3) | 64(3) | 43(3) | 5(3) | 9(3) | -5(3) |
| C5 | 37(2) | 55(3) | 35(2) | -1(2) | 7(2) | -3(2) |
| C6 | 39(2) | 53(3) | 36(2) | -2(2) | 12(2) | -2(2) |
| C7 | 39(2) | 40(2) | 39(2) | -2(2) | 11.0(19) | 2(2) |
| C8 | 35(2) | 45(3) | 39(3) | 3(2) | 10(2) | 5(2) |
| C9 | 38(2) | 45(3) | 30(2) | -3.4(19) | 8.8(19) | 2(2) |
| C10 | 38(2) | 47(3) | 35(2) | 3(2) | 8(2) | 4(2) |
| C11 | 36(2) | 49(3) | 33(2) | -3(2) | 7.7(19) | -3(2) |
| C12 | 39(3) | 57(3) | 50(3) | -10(2) | 11(2) | 5(2) |
| C13 | 42(3) | 83(4) | 47(3) | -13(3) | 17(3) | -3(3) |
| C14 | 49(3) | 68(4) | 41(3) | -4(3) | 16(2) | -14(3) |
| C15 | 46(3) | 52(3) | 38(3) | 0(2) | 11(2) | -6(2) |
| C16 | 34(2) | 54(3) | 33(2) | -5(2) | 5.6(19) | -1(2) |
| C17 | 39(2) | 42(2) | 40(3) | 2(2) | 7(2) | -2(2) |
| C18 | 40(3) | 69(4) | 46(3) | 4(3) | 10(2) | 3(3) |
| C19 | 60(4) | 72(4) | 41(3) | 4(3) | 12(3) | -8(3) |
| C20 | 54(3) | 57(3) | 45(3) | 13(3) | -6(3) | -9(3) |
| C21 | 44(3) | 64(4) | 64(4) | 7(3) | -3(3) | 11(3) |
| C22 | 49(3) | 48(3) | 42(3) | -4(2) | 14(2) | -2(2) |
| C23 | 38(3) | 45(3) | 58(3) | -8(2) | 17(2) | 1(2) |
| C24 | 44(3) | 51(3) | 60(4) | -8(3) | 9(3) | -1(2) |
| C25 | 48(3) | 71(4) | 73(4) | -15(4) | -2(3) | 4(3) |
| C26 | 35(3) | 93(6) | 103(7) | -35(5) | 16(4) | -9(3) |
| C27 | 61(4) | 78(5) | 115(7) | -24(5) | 48(5) | -24(4) |
| C28 | 59(4) | 60(4) | 76(4) | -14(3) | 33(3) | -11(3) |
| Br1' | 46.9(3) | 106.6(7) | 84.8(6) | -20.0(5) | -12.3(3) | 0.2(4) |
| O1' | 50(2) | 57(2) | 43(2) | -6.1(17) | 21.4(18) | -2.6(17) |
| O2' | 51(2) | 63(3) | 67(3) | 5(2) | 9(2) | -15(2) |
| N1' | 51(3) | 67(3) | 46(3) | -16(2) | 11(2) | -6(2) |
| C1' | 47(3) | 56(3) | 47(3) | 6(3) | 19(2) | 8(3) |
| C2' | 36(3) | 77(4) | 59(4) | 13(3) | 13(3) | 13(3) |
| C3' | 34(3) | 78(4) | 62(4) | 7(3) | 6(3) | -6(3) |
| C4' | 41(3) | 61(3) | 47(3) | 1(3) | 3(2) | -1(2) |
| C5' | 40(3) | 53(3) | 35(2) | -2(2) | 9(2) | 1(2) |
| C6' | 40(3) | 49(3) | 38(2) | 0(2) | 13(2) | 1(2) |
| C7' | 41(2) | 37(2) | 41(2) | -0.8(19) | 10(2) | -2.2(19) |
| C8' | 34(2) | 46(3) | 35(2) | -4(2) | 9(2) | -3(2) |
| C9' | 32(2) | 44(3) | 38(2) | -3(2) | 7.9(19) | 1.1(18) |
| C10' | 45(3) | 40(2) | 39(3) | -4(2) | 10(2) | -3(2) |
| C11' | 33(2) | 59(3) | 34(2) | 3(2) | 4(2) | 5(2) |
| C12' | 45(3) | 76(4) | 40(3) | 2(3) | 10(2) | 14(3) |
| C13' | 47(3) | 112(6) | 42(3) | 16(4) | 16(3) | 15(3) |

| | | | | | | |
|---|---|---|---|---|---|---|
| C14' | 49(3) | 95(6) | 58(4) | 26(4) | 22(3) | 6(4) |
| C15' | 38(3) | 62(3) | 55(3) | 16(3) | 7(2) | 2(2) |
| C16' | 32(2) | 50(3) | 39(2) | 5(2) | 6.5(19) | 4.1(19) |
| C17' | 41(3) | 38(2) | 37(2) | -3.2(19) | 8(2) | -3.7(19) |
| C18' | 45(3) | 55(3) | 40(3) | -2(2) | 12(2) | -4(2) |
| C19' | 43(3) | 62(3) | 50(3) | 1(3) | 12(2) | -3(3) |
| C20' | 36(2) | 55(3) | 55(3) | 0(3) | -4(2) | -3(2) |
| C21' | 58(3) | 79(4) | 49(3) | -20(3) | 6(3) | -5(4) |
| C22' | 48(3) | 42(3) | 48(3) | -2(2) | 18(2) | 0(2) |
| C23' | 60(3) | 42(3) | 44(3) | -3(2) | 19(3) | 7(2) |
| C24' | 58(3) | 52(3) | 48(3) | 4(3) | 10(3) | 3(3) |
| C25' | 74(4) | 75(4) | 45(3) | -7(3) | 3(3) | 7(4) |
| C26' | 104(6) | 89(5) | 38(3) | 7(3) | 22(4) | 22(5) |
| C27' | 109(7) | 83(5) | 57(4) | 21(4) | 41(5) | 11(5) |
| C28' | 67(4) | 68(4) | 60(4) | 10(3) | 30(3) | 5(3) |

**Supplementary Table 34. Bond Lengths for SX2502_auto.**

| tom | Atom | Length/Å | Atom | Atom | Length/Å |
|---|---|---|---|---|---|
| Br1 | C20 | 1.897(6) | Br1' | C20' | 1.902(5) |
| O1 | C8 | 1.413(6) | O1' | C8' | 1.413(6) |
| O2 | C22 | 1.211(8) | O2' | C22' | 1.219(8) |
| N1 | C17 | 1.335(7) | N1' | C17' | 1.323(7) |
| N1 | C21 | 1.334(8) | N1' | C21' | 1.342(8) |
| C1 | C2 | 1.403(10) | C1' | C2' | 1.397(9) |
| C1 | C6 | 1.365(8) | C1' | C6' | 1.378(8) |
| C2 | C3 | 1.380(12) | C2' | C3' | 1.376(11) |
| C3 | C4 | 1.402(11) | C3' | C4' | 1.393(9) |
| C4 | C5 | 1.373(8) | C4' | C5' | 1.383(8) |
| C5 | C6 | 1.393(8) | C5' | C6' | 1.387(8) |
| C5 | C10 | 1.521(7) | C5' | C10' | 1.509(8) |
| C6 | C7 | 1.527(7) | C6' | C7' | 1.524(7) |
| C7 | C8 | 1.571(7) | C7' | C8' | 1.572(7) |
| C7 | C16 | 1.505(7) | C7' | C16' | 1.504(7) |
| C8 | C9 | 1.581(7) | C8' | C9' | 1.581(7) |
| C8 | C17 | 1.537(7) | C8' | C17' | 1.539(7) |
| C9 | C10 | 1.555(7) | C9' | C10' | 1.569(7) |
| C9 | C22 | 1.540(8) | C9' | C22' | 1.518(8) |
| C10 | C11 | 1.514(7) | C10' | C11' | 1.517(8) |
| C11 | C12 | 1.387(8) | C11' | C12' | 1.380(8) |
| C11 | C16 | 1.396(8) | C11' | C16' | 1.385(8) |
| C12 | C13 | 1.388(10) | C12' | C13' | 1.384(11) |
| C13 | C14 | 1.368(10) | C13' | C14' | 1.357(13) |
| C14 | C15 | 1.402(9) | C14' | C15' | 1.408(10) |

| | | | | | |
|---|---|---|---|---|---|
| C15 | C16 | 1.377(8) | C15' | C16' | 1.386(8) |
| C17 | C18 | 1.385(9) | C17' | C18' | 1.380(8) |
| C18 | C19 | 1.378(9) | C18' | C19' | 1.392(8) |
| C19 | C20 | 1.394(10) | C19' | C20' | 1.370(9) |
| C20 | C21 | 1.355(11) | C20' | C21' | 1.360(10) |
| C22 | C23 | 1.491(8) | C22' | C23' | 1.498(9) |
| C23 | C24 | 1.386(10) | C23' | C24' | 1.386(9) |
| C23 | C28 | 1.400(9) | C23' | C28' | 1.389(9) |
| C24 | C25 | 1.383(9) | C24' | C25' | 1.388(10) |
| C25 | C26 | 1.375(13) | C25' | C26' | 1.381(12) |
| C26 | C27 | 1.386(14) | C26' | C27' | 1.366(14) |
| C27 | C28 | 1.375(11) | C27' | C28' | 1.392(11) |

**Supplementary Table 35. Bond Angles for SX2502_auto.**

| **Atom** | **Atom** | **Atom** | **Angle/°** | **Atom** | **Atom** | **Atom** | **Angle/°** |
|---|---|---|---|---|---|---|---|
| C21 | N1 | C17 | 118.6(6) | C17' | N1' | C21' | 118.6(5) |
| C6 | C1 | C2 | 119.4(7) | C6' | C1' | C2' | 118.9(6) |
| C3 | C2 | C1 | 120.1(6) | C3' | C2' | C1' | 120.5(6) |
| C2 | C3 | C4 | 120.0(6) | C2' | C3' | C4' | 120.4(6) |
| C5 | C4 | C3 | 119.5(7) | C5' | C4' | C3' | 119.2(6) |
| C4 | C5 | C6 | 120.3(5) | C4' | C5' | C6' | 120.3(5) |
| C4 | C5 | C10 | 126.1(5) | C4' | C5' | C10' | 126.4(5) |
| C6 | C5 | C10 | 113.5(5) | C6' | C5' | C10' | 113.3(5) |
| C1 | C6 | C5 | 120.7(5) | C1' | C6' | C5' | 120.7(5) |
| C1 | C6 | C7 | 126.8(5) | C1' | C6' | C7' | 126.4(5) |
| C5 | C6 | C7 | 112.4(5) | C5' | C6' | C7' | 112.9(5) |
| C6 | C7 | C8 | 105.6(4) | C6' | C7' | C8' | 105.6(4) |
| C16 | C7 | C6 | 106.5(4) | C16' | C7' | C6' | 106.3(4) |
| C16 | C7 | C8 | 110.0(4) | C16' | C7' | C8' | 109.9(4) |
| O1 | C8 | C7 | 108.2(4) | O1' | C8' | C7' | 108.0(4) |
| O1 | C8 | C9 | 108.5(4) | O1' | C8' | C9' | 108.2(4) |
| O1 | C8 | C17 | 109.7(4) | O1' | C8' | C17' | 110.0(4) |
| C7 | C8 | C9 | 107.8(4) | C7' | C8' | C9' | 108.0(4) |
| C17 | C8 | C7 | 111.3(4) | C17' | C8' | C7' | 110.4(4) |
| C17 | C8 | C9 | 111.3(4) | C17' | C8' | C9' | 112.2(4) |
| C10 | C9 | C8 | 109.2(4) | C10' | C9' | C8' | 108.8(4) |
| C22 | C9 | C8 | 113.9(4) | C22' | C9' | C8' | 113.4(4) |
| C22 | C9 | C10 | 111.1(4) | C22' | C9' | C10' | 111.4(4) |
| C5 | C10 | C9 | 109.5(4) | C5' | C10' | C9' | 108.5(4) |
| C11 | C10 | C5 | 106.7(4) | C5' | C10' | C11' | 107.0(4) |
| C11 | C10 | C9 | 104.9(4) | C11' | C10' | C9' | 106.2(4) |
| C12 | C11 | C10 | 125.9(5) | C12' | C11' | C10' | 125.9(6) |
| C12 | C11 | C16 | 120.9(5) | C12' | C11' | C16' | 121.4(6) |

| | | | | | | | |
|---|---|---|---|---|---|---|---|
| C16 | C11 | C10 | 113.3(5) | C16' | C11' | C10' | 112.8(5) |
| C11 | C12 | C13 | 118.4(6) | C11' | C12' | C13' | 118.7(7) |
| C14 | C13 | C12 | 121.6(6) | C14' | C13' | C12' | 120.4(6) |
| C13 | C14 | C15 | 119.7(6) | C13' | C14' | C15' | 121.8(7) |
| C16 | C15 | C14 | 119.7(6) | C16' | C15' | C14' | 117.6(7) |
| C11 | C16 | C7 | 113.0(5) | C11' | C16' | C7' | 113.6(5) |
| C15 | C16 | C7 | 127.2(5) | C11' | C16' | C15' | 120.1(6) |
| C15 | C16 | C11 | 119.7(5) | C15' | C16' | C7' | 126.3(5) |
| N1 | C17 | C8 | 114.3(5) | N1' | C17' | C8' | 114.5(5) |
| N1 | C17 | C18 | 121.8(5) | N1' | C17' | C18' | 122.5(5) |
| C18 | C17 | C8 | 123.8(5) | C18' | C17' | C8' | 122.8(5) |
| C19 | C18 | C17 | 119.4(6) | C17' | C18' | C19' | 118.6(5) |
| C18 | C19 | C20 | 117.4(6) | C20' | C19' | C18' | 117.8(6) |
| C19 | C20 | Br1 | 119.1(5) | C19' | C20' | Br1' | 119.3(5) |
| C21 | C20 | Br1 | 120.7(5) | C21' | C20' | Br1' | 120.1(5) |
| C21 | C20 | C19 | 120.0(6) | C21' | C20' | C19' | 120.5(5) |
| N1 | C21 | C20 | 122.5(6) | N1' | C21' | C20' | 121.8(6) |
| O2 | C22 | C9 | 119.1(5) | O2' | C22' | C9' | 119.8(6) |
| O2 | C22 | C23 | 120.1(6) | O2' | C22' | C23' | 119.6(6) |
| C23 | C22 | C9 | 120.5(5) | C23' | C22' | C9' | 120.4(5) |
| C24 | C23 | C22 | 123.5(6) | C24' | C23' | C22' | 123.2(6) |
| C24 | C23 | C28 | 119.0(6) | C24' | C23' | C28' | 119.3(6) |
| C28 | C23 | C22 | 117.4(6) | C28' | C23' | C22' | 117.5(6) |
| C25 | C24 | C23 | 120.1(7) | C23' | C24' | C25' | 119.9(7) |
| C26 | C25 | C24 | 120.7(8) | C26' | C25' | C24' | 120.3(8) |
| C25 | C26 | C27 | 119.6(7) | C27' | C26' | C25' | 120.2(7) |
| C28 | C27 | C26 | 120.3(8) | C26' | C27' | C28' | 120.0(8) |
| C27 | C28 | C23 | 120.2(8) | C23' | C28' | C27' | 120.3(8) |

**Supplementary Table 36. Hydrogen Bonds for SX2502_auto.**

| D | H | A | d(D-H)/Å | d(H-A)/Å | d(D-A)/Å | D-H-A/° |
|---|---|---|---|---|---|---|
| O1 | H1 | N1 | 0.82 | 2.04 | 2.571(7) | 121.7 |
| O1' | H1' | N1' | 0.88(3) | 2.06(10) | 2.585(7) | 117(9) |

**Supplementary Table 37. Torsion Angles for SX2502_auto.**

| A | B | C | D | Angle/˚ | A | B | C | D | Angle/˚ |
|---|---|---|---|---|---|---|---|---|---|
| Br1 | C20 | C21 | N1 | -172.9(5) | Br1' | C20' | C21' | N1' | -175.1(6) |
| O1 | C8 | C9 | C10 | 108.2(5) | O1' | C8' | C9' | C10' | 111.0(5) |
| O1 | C8 | C9 | C22 | -16.6(6) | O1' | C8' | C9' | C22' | -13.6(6) |
| O1 | C8 | C17 | N1 | -7.7(7) | O1' | C8' | C17' | N1' | -10.8(7) |
| O1 | C8 | C17 | C18 | 170.9(5) | O1' | C8' | C17' | C18' | 165.5(5) |
| O2 | C22 | C23 | C24 | 172.8(6) | O2' | C22' | C23' | C24' | 170.4(6) |
| O2 | C22 | C23 | C28 | -4.3(9) | O2' | C22' | C23' | C28' | -6.8(9) |

| | | | | | | | | | |
|---|---|---|---|---|---|---|---|---|---|
| N1 | C17 | C18 | C19 | 5.4(10) | N1' | C17' | C18' | C19' | 4.0(9) |
| C1 | C2 | C3 | C4 | 0.2(11) | C1' | C2' | C3' | C4' | 0.4(10) |
| C1 | C6 | C7 | C8 | 119.4(6) | C1' | C6' | C7' | C8' | 118.2(6) |
| C1 | C6 | C7 | C16 | -123.6(6) | C1' | C6' | C7' | C16' | -125.0(6) |
| C2 | C1 | C6 | C5 | -1.3(9) | C2' | C1' | C6' | C5' | -1.5(9) |
| C2 | C1 | C6 | C7 | 178.0(6) | C2' | C1' | C6' | C7' | 178.4(5) |
| C2 | C3 | C4 | C5 | -0.6(10) | C2' | C3' | C4' | C5' | -1.3(10) |
| C3 | C4 | C5 | C6 | 0.1(9) | C3' | C4' | C5' | C6' | 0.8(9) |
| C3 | C4 | C5 | C10 | -177.9(6) | C3' | C4' | C5' | C10' | -178.8(6) |
| C4 | C5 | C6 | C1 | 0.9(8) | C4' | C5' | C6' | C1' | 0.6(8) |
| C4 | C5 | C6 | C7 | -178.5(5) | C4' | C5' | C6' | C7' | -179.3(5) |
| C4 | C5 | C10 | C9 | -123.4(6) | C4' | C5' | C10' | C9' | -121.1(6) |
| C4 | C5 | C10 | C11 | 123.6(6) | C4' | C5' | C10' | C11' | 124.6(6) |
| C5 | C6 | C7 | C8 | -61.2(5) | C5' | C6' | C7' | C8' | -61.8(6) |
| C5 | C6 | C7 | C16 | 55.8(6) | C5' | C6' | C7' | C16' | 54.9(6) |
| C5 | C10 | C11 | C12 | -125.7(6) | C5' | C10' | C11' | C12' | -126.1(6) |
| C5 | C10 | C11 | C16 | 54.1(5) | C5' | C10' | C11' | C16' | 54.1(6) |
| C6 | C1 | C2 | C3 | 0.8(10) | C6' | C1' | C2' | C3' | 0.9(10) |
| C6 | C5 | C10 | C9 | 58.5(6) | C6' | C5' | C10' | C9' | 59.2(6) |
| C6 | C5 | C10 | C11 | -54.4(6) | C6' | C5' | C10' | C11' | -55.0(6) |
| C6 | C7 | C8 | O1 | -53.2(5) | C6' | C7' | C8' | O1' | -55.0(5) |
| C6 | C7 | C8 | C9 | 63.9(5) | C6' | C7' | C8' | C9' | 61.8(5) |
| C6 | C7 | C8 | C17 | -173.8(4) | C6' | C7' | C8' | C17' | -175.2(4) |
| C6 | C7 | C16 | C11 | -56.3(6) | C6' | C7' | C16' | C11' | -56.0(6) |
| C6 | C7 | C16 | C15 | 124.1(6) | C6' | C7' | C16' | C15' | 123.5(5) |
| C7 | C8 | C9 | C10 | -8.8(5) | C7' | C8' | C9' | C10' | -5.6(5) |
| C7 | C8 | C9 | C22 | -133.6(4) | C7' | C8' | C9' | C22' | -130.2(5) |
| C7 | C8 | C17 | N1 | 111.9(5) | C7' | C8' | C17' | N1' | 108.2(5) |
| C7 | C8 | C17 | C18 | -69.5(7) | C7' | C8' | C17' | C18' | -75.5(6) |
| C8 | C7 | C16 | C11 | 57.7(5) | C8' | C7' | C16' | C11' | 57.8(5) |
| C8 | C7 | C16 | C15 | -121.9(6) | C8' | C7' | C16' | C15' | -122.8(5) |
| C8 | C9 | C10 | C5 | -50.4(5) | C8' | C9' | C10' | C5' | -53.4(5) |
| C8 | C9 | C10 | C11 | 63.7(5) | C8' | C9' | C10' | C11' | 61.3(5) |
| C8 | C9 | C22 | O2 | 112.7(6) | C8' | C9' | C22' | O2' | 116.0(6) |
| C8 | C9 | C22 | C23 | -73.2(6) | C8' | C9' | C22' | C23' | -69.8(6) |
| C8 | C17 | C18 | C19 | -173.1(6) | C8' | C17' | C18' | C19' | -172.0(5) |
| C9 | C8 | C17 | N1 | -127.8(5) | C9' | C8' | C17' | N1' | -131.3(5) |
| C9 | C8 | C17 | C18 | 50.8(7) | C9' | C8' | C17' | C18' | 45.0(7) |
| C9 | C10 | C11 | C12 | 118.2(5) | C9' | C10' | C11' | C12' | 118.2(6) |
| C9 | C10 | C11 | C16 | -62.0(5) | C9' | C10' | C11' | C16' | -61.7(5) |
| C9 | C22 | C23 | C24 | -1.3(8) | C9' | C22' | C23' | C24' | -3.8(8) |
| C9 | C22 | C23 | C28 | -178.4(5) | C9' | C22' | C23' | C28' | 179.1(5) |
| C10 | C5 | C6 | C1 | 179.1(5) | C10' | C5' | C6' | C1' | -179.7(5) |
| C10 | C5 | C6 | C7 | -0.3(6) | C10' | C5' | C6' | C7' | 0.3(7) |

| | | | | | | | | | |
|---|---|---|---|---|---|---|---|---|---|
| C10 | C9 | C22 | O2 | -11.1(8) | C10' | C9' | C22' | O2' | -7.2(7) |
| C10 | C9 | C22 | C23 | 163.0(5) | C10' | C9' | C22' | C23' | 167.0(5) |
| C10 | C11 | C12 | C13 | 178.8(5) | C10' | C11' | C12' | C13' | 177.3(5) |
| C10 | C11 | C16 | C7 | 1.2(6) | C10' | C11' | C16' | C7' | 1.6(6) |
| C10 | C11 | C16 | C15 | -179.2(5) | C10' | C11' | C16' | C15' | -177.9(5) |
| C11 | C12 | C13 | C14 | 0.2(9) | C11' | C12' | C13' | C14' | 1.6(9) |
| C12 | C11 | C16 | C7 | -179.0(5) | C12' | C11' | C16' | C7' | -178.3(5) |
| C12 | C11 | C16 | C15 | 0.7(8) | C12' | C11' | C16' | C15' | 2.3(8) |
| C12 | C13 | C14 | C15 | 0.9(9) | C12' | C13' | C14' | C15' | 0.4(10) |
| C13 | C14 | C15 | C16 | -1.2(8) | C13' | C14' | C15' | C16' | -1.0(9) |
| C14 | C15 | C16 | C7 | -179.9(5) | C14' | C15' | C16' | C7' | -179.7(5) |
| C14 | C15 | C16 | C11 | 0.5(8) | C14' | C15' | C16' | C11' | -0.3(8) |
| C16 | C7 | C8 | O1 | -167.8(4) | C16' | C7' | C8' | O1' | -169.2(4) |
| C16 | C7 | C8 | C9 | -50.6(5) | C16' | C7' | C8' | C9' | -52.4(5) |
| C16 | C7 | C8 | C17 | 71.7(5) | C16' | C7' | C8' | C17' | 70.6(5) |
| C16 | C11 | C12 | C13 | -1.0(8) | C16' | C11' | C12' | C13' | -2.9(8) |
| C17 | N1 | C21 | C20 | 1.0(11) | C17' | N1' | C21' | C20' | 1.2(11) |
| C17 | C8 | C9 | C10 | -131.1(4) | C17' | C8' | C9' | C10' | -127.5(5) |
| C17 | C8 | C9 | C22 | 104.1(5) | C17' | C8' | C9' | C22' | 107.9(5) |
| C17 | C18 | C19 | C20 | -2.0(10) | C17' | C18' | C19' | C20' | -1.0(9) |
| C18 | C19 | C20 | Br1 | 173.6(5) | C18' | C19' | C20' | Br1' | 175.1(5) |
| C18 | C19 | C20 | C21 | -1.7(11) | C18' | C19' | C20' | C21' | -1.7(10) |
| C19 | C20 | C21 | N1 | 2.3(11) | C19' | C20' | C21' | N1' | 1.7(12) |
| C21 | N1 | C17 | C8 | 173.7(6) | C21' | N1' | C17' | C8' | 172.2(6) |
| C21 | N1 | C17 | C18 | -4.9(10) | C21' | N1' | C17' | C18' | -4.1(10) |
| C22 | C9 | C10 | C5 | 76.0(5) | C22' | C9' | C10' | C5' | 72.3(5) |
| C22 | C9 | C10 | C11 | -169.9(4) | C22' | C9' | C10' | C11' | -173.0(4) |
| C22 | C23 | C24 | C25 | -179.5(6) | C22' | C23' | C24' | C25' | -178.6(6) |
| C22 | C23 | C28 | C27 | 178.5(6) | C22' | C23' | C28' | C27' | 178.1(7) |
| C23 | C24 | C25 | C26 | 2.4(11) | C23' | C24' | C25' | C26' | 0.5(11) |
| C24 | C23 | C28 | C27 | 1.2(10) | C24' | C23' | C28' | C27' | 0.9(10) |
| C24 | C25 | C26 | C27 | -1.2(12) | C24' | C25' | C26' | C27' | 1.2(13) |
| C25 | C26 | C27 | C28 | 0.0(12) | C25' | C26' | C27' | C28' | -1.8(13) |
| C26 | C27 | C28 | C23 | -0.1(11) | C26' | C27' | C28' | C23' | 0.8(12) |
| C28 | C23 | C24 | C25 | -2.4(9) | C28' | C23' | C24' | C25' | -1.5(10) |

**Supplementary Table 38. Hydrogen Atom Coordinates (Å×10$^4$) and Isotropic Displacement Parameters (Å$^2$×10$^3$) for SX2502_auto.**

| **Atom** | ***x*** | ***y*** | ***z*** | **U(eq)** |
|---|---|---|---|---|
| H1 | 10894 | 3056 | 6293 | 76 |
| H1A | 7489 | 2368 | 3077 | 66 |
| H2 | 6536 | 2673 | 636 | 80 |
| H3 | 5713 | 3511 | 165 | 81 |
| H4 | 5844 | 4055 | 2133 | 66 |

| | | | | |
|---|---|---|---|---|
| H7 | 8016 | 2526 | 5840 | 48 |
| H9 | 8472 | 3943 | 7208 | 46 |
| H10 | 6423 | 4177 | 4905 | 49 |
| H12 | 4484 | 4043 | 6143 | 60 |
| H13 | 3319 | 3482 | 7255 | 69 |
| H14 | 4057 | 2642 | 7671 | 63 |
| H15 | 6057 | 2344 | 7029 | 56 |
| H18 | 8193 | 3306 | 8867 | 64 |
| H19 | 9621 | 3177 | 11371 | 71 |
| H21 | 13016 | 2715 | 10170 | 77 |
| H24 | 10984 | 3994 | 8660 | 66 |
| H25 | 13317 | 4283 | 10202 | 84 |
| H26 | 14632 | 4869 | 9355 | 96 |
| H27 | 13652 | 5145 | 6910 | 96 |
| H28 | 11364 | 4840 | 5333 | 75 |
| H1' | 8110(100) | 6280(40) | 9060(80) | 100(30) |
| H1'A | 4758 | 6894 | 5757 | 59 |
| H2' | 2350 | 6541 | 4743 | 71 |
| H3' | 2022 | 5720 | 3807 | 73 |
| H4' | 4095 | 5229 | 3891 | 64 |
| H7' | 7590 | 6806 | 6272 | 49 |
| H9' | 9174 | 5401 | 6637 | 47 |
| H10' | 6932 | 5177 | 4491 | 51 |
| H12' | 8257 | 5387 | 2688 | 66 |
| H13' | 9258 | 6008 | 1586 | 80 |
| H14' | 9509 | 6832 | 2413 | 80 |
| H15' | 8783 | 7076 | 4393 | 66 |
| H18' | 10788 | 6029 | 6420 | 57 |
| H19' | 13321 | 6197 | 7856 | 63 |
| H21' | 12006 | 6672 | 11113 | 79 |
| H24' | 10524 | 5361 | 9160 | 66 |
| H25' | 11992 | 5077 | 11512 | 83 |
| H26' | 11016 | 4509 | 12764 | 94 |
| H27' | 8611 | 4199 | 11652 | 96 |
| H28' | 7107 | 4491 | 9315 | 75 |

## 7. Characterization of adducts

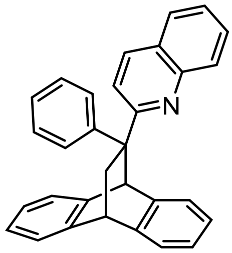


**3a:** White solid; Mp 161.8−163.4 °C; 34.0 mg, 83% yield, 90% ee; $[\alpha]_D^{22}$ –14.6 (*c* 2.0, $CHCl_3$); $^1$H NMR (300 MHz, Methylene Chloride-$d_2$) δ 8.03 (d, *J* = 8.6 Hz, 1H), 7.91 (d, *J* = 8.7 Hz, 1H), 7.68 (d, *J* = 7.8 Hz, 2H), 7.46 (t, *J* = 7.6 Hz, 1H), 7.37 (dd, *J* = 11.4, 7.3 Hz, 2H), 7.25 (t, *J* = 7.4 Hz, 2H), 7.18 (d, *J* = 7.6 Hz, 3H), 7.09 (t, *J* = 7.6 Hz, 3H), 7.00 (q, *J* = 7.2 Hz, 2H), 6.88 (dt, *J* = 19.7, 7.3 Hz, 2H), 5.60 (s, 1H), 4.51 (t, *J* = 2.6 Hz, 1H), 3.48 (d, *J* = 12.9 Hz, 1H), 2.88 (d, *J* = 12.8 Hz, 1H); $^{13}$C NMR (75 MHz, Methylene Chloride-$d_2$) δ 166.4, 147.9, 146.8, 145.6, 145.2, 142.7, 142.4, 136.2, 129.5, 129.5, 128.2, 128.1, 127.6, 127.1, 126.6, 126.5, 126.4, 126.1, 126.0, 125.7, 125.6, 125.2, 123.5, 123.1, 122.2, 56.8, 53.4, 45.4, 40.7; HRMS (ESI) m/z 410.1906 $(M + H)^+$, calc. for $C_{31}H_{24}N$ 410.1903.

The ee was determined by HPLC analysis: IF*2 (4.6 mm i.d. x 250 mm); hexane/2–propanol = 95/5; flow rate 1.0 mL/min; 25 °C; 210 nm; retention time: 10.6 min (minor) and 11.2 min (major).

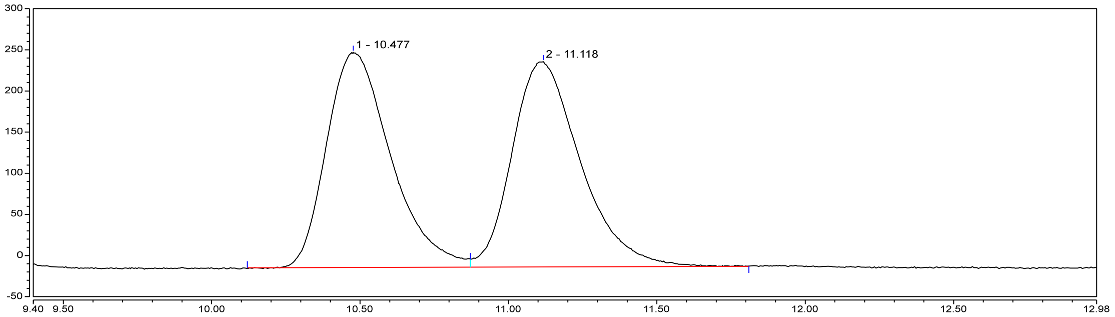


| Entry | Retention | Area | Height | %Area |
|---|---|---|---|---|
| 1 | 10.477 | 65.4892 | 261.60 | 49.62 |
| 2 | 11.118 | 66.4824 | 249.50 | 50.38 |

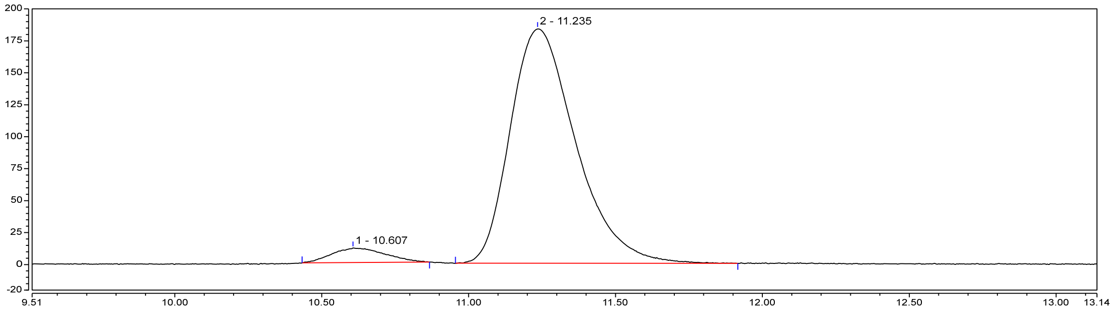


| Entry | Retention | Area | Height | %Area |
|---|---|---|---|---|
| 1 | 10.607 | 2.3823 | 11.42 | 4.78 |
| 2 | 11.235 | 47.4042 | 183.63 | 95.22 |

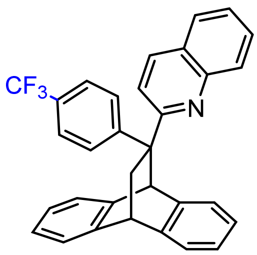


**3b:** White solid; Mp 165.7−167.5 ºC; 31.9 mg, 67% yield, 84% ee; $[\alpha]^{22}_{D}$ −9.1 (*c* 2.0, $CHCl_3$); $^1$H NMR (300 MHz, Chloroform-*d*) δ 8.09 (d, *J* = 8.4 Hz, 1H), 7.88 (d, *J* = 8.7 Hz, 1H), 7.68 (t, *J* = 9.4 Hz, 2H), 7.47 (t, *J* = 7.3 Hz, 2H), 7.36 (d, *J* = 7.3 Hz, 1H), 7.29 (d, *J* = 8.3 Hz, 2H), 7.21 (d, *J* = 7.4 Hz, 1H), 7.11 (d, *J* = 8.7 Hz, 3H), 7.09 – 6.99 (m, 2H), 6.96 (d, *J* = 7.4 Hz, 1H), 6.89 (t, *J* = 6.4 Hz, 2H), 5.52 (s, 1H), 4.52 (d, *J* = 2.6 Hz, 1H), 3.27 (dd, *J* = 12.9, 2.5 Hz, 1H), 2.92 (dd, *J* = 12.9, 2.9 Hz, 1H); $^{19}$F NMR (565 MHz, Chloroform-*d*) δ -62.45; $^{13}$C NMR (75 MHz, Chloroform-*d*) δ 165.3, 151.4, 146.4, 144.5, 144.3, 141.8, 141.5, 135.9, 129.4, 129.3 (d, $J_{F\text{-}C}$ = 31.4 Hz), 129.2, 127.9, 127.2, 127.1, 126.2, 126.1, 126.0, 125.9, 125.5, 125.3, 125.0, 124.1 (q, $J_{F\text{-}C}$ = 264.4 Hz), 124.5 (q, $J_{F\text{-}C}$ = 3.7 Hz), 123.1, 122.5, 121.9, 56.4, 53.4, 44.9, 40.7; HRMS (ESI) m/z 478.1779 $(M + H)^+$, calc. for $C_{32}H_{23}F_3N$ 478.1777.

The ee was determined by HPLC analysis: (Amylose-1) * 2 (4.6 mm i.d. x 250 mm); hexane/2–propanol = 98/2; flow rate 1.0 mL/min; 25 ºC; 210 nm; retention time: 12.7 min (minor) and 13.5 min (major).

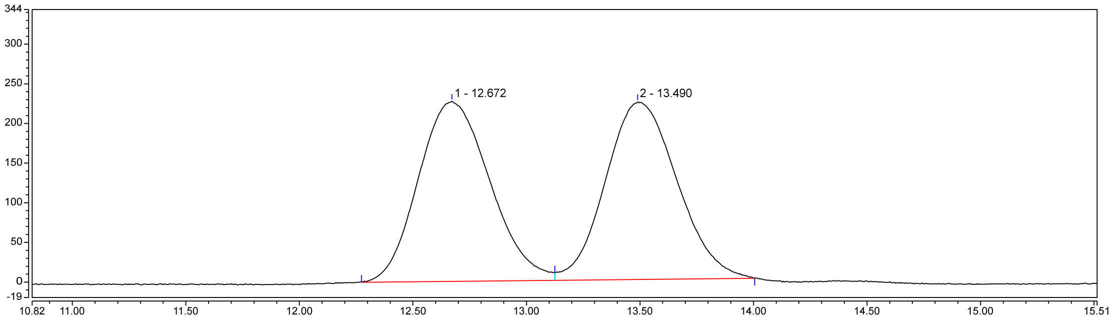


| Entry | Retention | Area | Height | %Area |
|---|---|---|---|---|
| 1 | 12.672 | 82.8258 | 226.99 | 50.28 |
| 2 | 13.490 | 81.9006 | 224.28 | 49.72 |

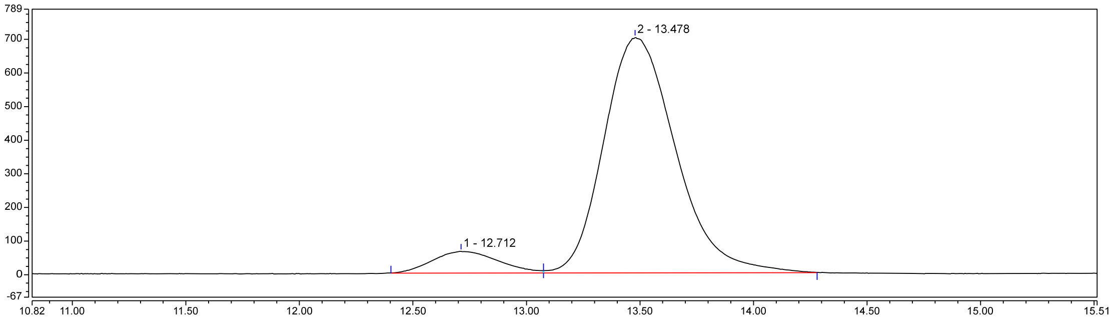


| Entry | Retention | Area | Height | %Area |
|---|---|---|---|---|
| 1 | 12.712 | 21.5602 | 64.59 | 7.75 |
| 2 | 13.478 | 256.6465 | 700.39 | 92.25 |

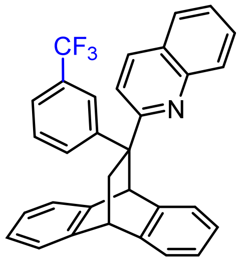


**3c:** White solid; Mp 165.5−167.1 °C; 33.8 mg, 71% yield, 92% ee; $[\alpha]_D^{22}$ −12.8 (*c* 2.0, $CHCl_3$); $^1H$ NMR (300 MHz, Chloroform-*d*) δ 8.11 (d, *J* = 8.4 Hz, 1H), 7.89 (d, *J* = 8.6 Hz, 1H), 7.69 (t, *J* = 9.2 Hz, 2H), 7.48 (t, *J* = 7.6 Hz, 2H), 7.38 (d, *J* = 7.4 Hz, 1H), 7.25 (d, *J* = 7.5 Hz, 2H), 7.21 – 7.14 (m, 3H), 7.14 – 7.06 (m, 2H), 7.01 – 6.86 (m, 4H), 5.52 (s, 1H), 4.53 (s, 1H), 3.24 (d, *J* = 12.9 Hz, 1H), 2.94 (d, *J* = 12.7 Hz, 1H); $^{19}F$ NMR (565 MHz, Chloroform-*d*) δ -62.50; $^{13}C$ NMR (75 MHz, Chloroform-*d*) δ 165.3, 148.5, 146.4, 144.4, 144.3, 141.8, 141.5, 135.9, 131.4, 129.7 (d, $J_{F\text{-}C}$ = 31.9 Hz), 129.4, 129.2, 128.1, 127.2, 127.1, 126.2, 126.1, 126.0, 125.9, 125.6, 125.3, 125.0, 124.1 (d, $J_{F\text{-}C}$ = 272.6 Hz), 123.9 (q, $J_{F\text{-}C}$ = 3.8 Hz), 123.2, 122.5, 122.4 (q, $J_{F\text{-}C}$ = 4.0 Hz), 122.0, 56.3, 53.5, 44.8, 41.0; HRMS (ESI) m/z 478.1780 $(M + H)^+$, calc. for $C_{32}H_{23}F_3N$ 478.1777.

The ee was determined by HPLC analysis: (Amylose-1) *2 (4.6 mm i.d. x 250 mm); hexane/2–propanol = 90/10; flow rate 1.0 mL/min; 25 °C; 210 nm; retention time: 12.3 min (minor) and 13.4 min (major).

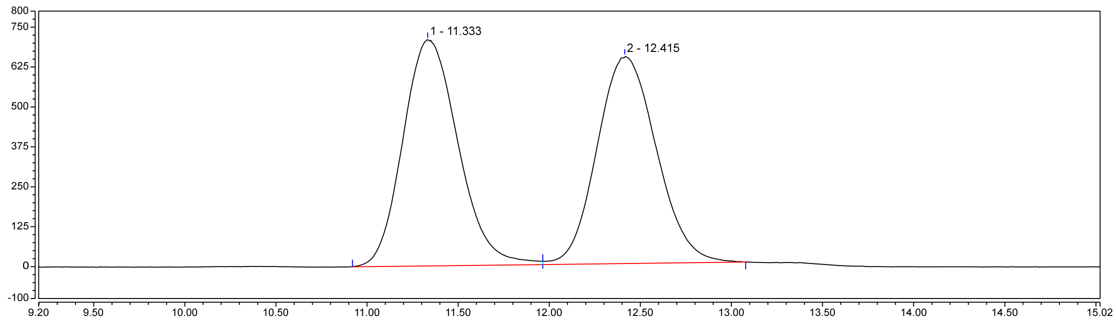


| Entry | Retention | Area | Height | %Area |
|---|---|---|---|---|
| 1 | 11.333 | 248.9458 | 708.51 | 50.38 |
| 2 | 12.415 | 245.1635 | 648.79 | 49.62 |

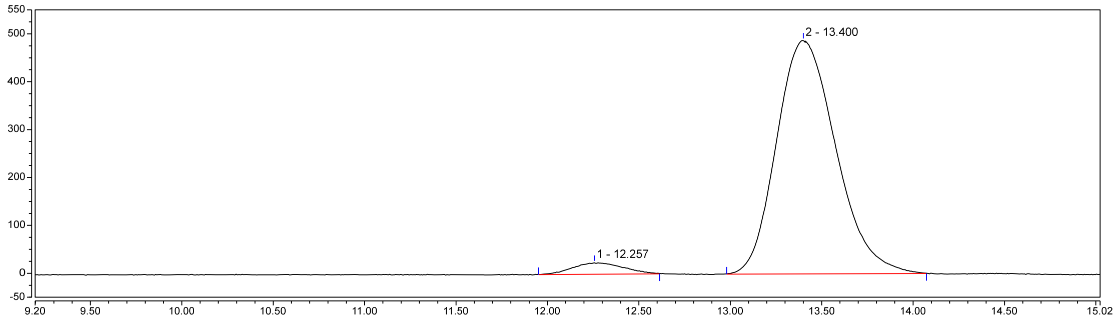


| Entry | Retention | Area | Height | %Area |
|---|---|---|---|---|
| 1 | 12.257 | 7.6486 | 24.06 | 4.01 |
| 2 | 13.400 | 183.2937 | 487.76 | 95.99 |

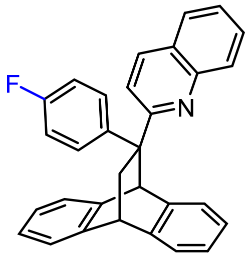

**3d:** White solid; Mp 128.2−130.1 °C; 35.0 mg, 82% yield, 86% ee; $[\alpha]_D^{22}$ −15.7 (*c* 2.0, $CHCl_3$); $^1H$ NMR (300 MHz, Chloroform-*d*) δ 8.12 (d, *J* = 8.4 Hz, 1H), 7.87 (d, *J* = 8.7 Hz, 1H), 7.72 – 7.65 (m, 2H), 7.53 – 7.44 (m, 2H), 7.37 (d, *J* = 7.3 Hz, 1H), 7.21 (d, *J* = 5.6 Hz, 1H), 7.16 – 7.06 (m, 2H), 7.06 – 6.84 (m, 6H), 6.73 (t, *J* = 8.5 Hz, 2H), 5.51 (s, 1H), 4.52 (s, 1H), 3.17 (d, *J* = 13.0 Hz, 1H), 2.95 (d, *J* = 12.9 Hz, 1H); $^{19}F$ NMR (376 MHz, Chloroform-*d*) δ -117.76; $^{13}C$ NMR (75 MHz, Chloroform-*d*) δ 166.0, 160.7 (d, $J_{F\text{-}C}$ = 244.9 Hz), 146.3, 144.4, 144.3, 143.0 (d, $J_{F\text{-}C}$ = 3.3 Hz), 142.1, 141.9, 135.7, 129.3, 129.1, 129.0 (d, $J_{F\text{-}C}$ = 7.9 Hz), 127.2, 127.2, 126.1, 126.0, 125.7, 125.4, 125.2, 124.9, 123.1, 122.4, 122.2, 114.3 (d, $J_{F\text{-}C}$ = 21.1 Hz), 55.7, 53.7, 44.9, 40.9; HRMS (ESI) m/z 428.1812 $(M + H)^+$, calc. for $C_{31}H_{23}FN$ 428.1809.

The ee was determined by HPLC analysis: IF*2 (4.6 mm i.d. x 250 mm); hexane/2–propanol = 99/1; flow rate 1.0 mL/min; 25 °C; 210 nm; retention time: 12.9 min (minor) and 13.9 min (major).

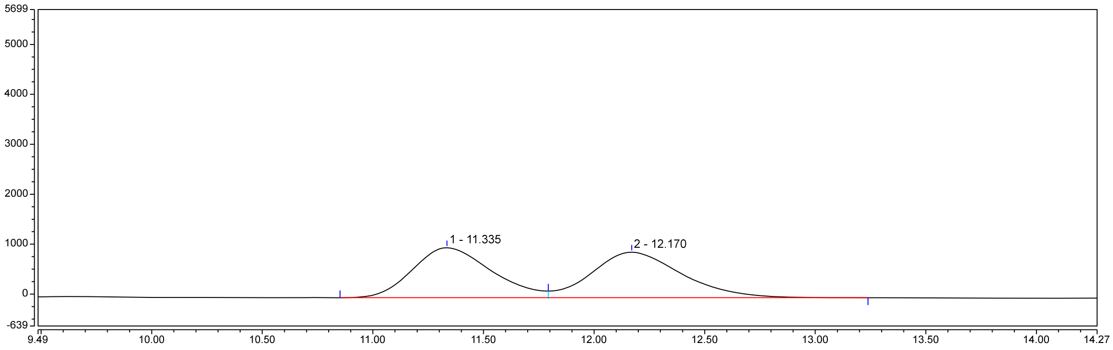


| Entry | Retention | Area | Height | %Area |
|---|---|---|---|---|
| 1 | 11.335 | 413.0745 | 999.32 | 49.08 |
| 2 | 12.170 | 428.5169 | 910.64 | 50.92 |

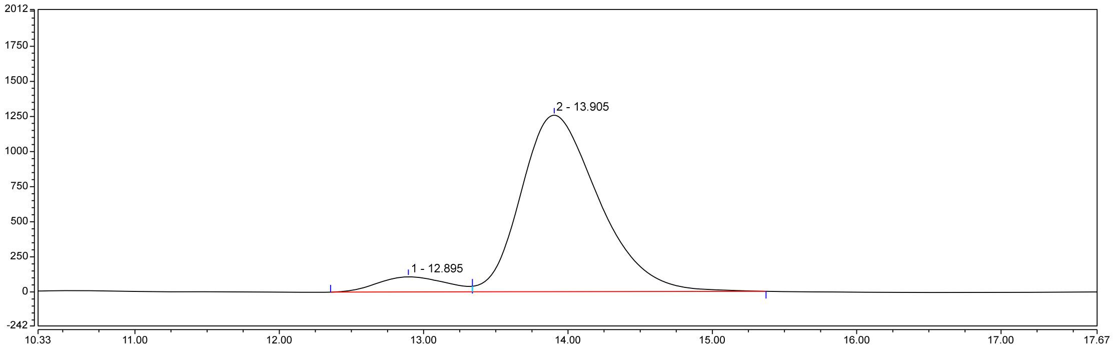


| Entry | Retention | Area | Height | %Area |
|---|---|---|---|---|
| 1 | 12.895 | 59.3744 | 107.93 | 7.05 |

| | | | | |
|---|---|---|---|---|
| 2 | 13.905 | 783.2614 | 1256.76 | 92.95 |

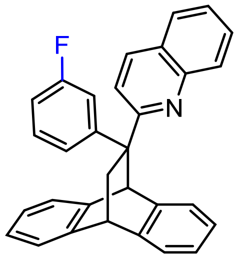


**3e:** White solid; Mp 182.7−184.1 °C; 38.0 mg, 89% yield, 90% ee; $[\alpha]_D^{22}$ –5.6 (*c* 2.0, $CHCl_3$); $^1$H NMR (300 MHz, Chloroform-*d*) δ 8.09 (d, *J* = 8.4 Hz, 1H), 7.87 (d, *J* = 8.7 Hz, 1H), 7.68 (t, *J* = 9.5 Hz, 2H), 7.46 (t, *J* = 7.6 Hz, 2H), 7.36 (d, *J* = 7.3 Hz, 1H), 7.20 (d, *J* = 7.7 Hz, 1H), 7.17 – 7.02 (m, 3H), 6.97 (q, *J* = 7.4, 6.6 Hz, 2H), 6.90 – 6.84 (m, 2H), 6.81 – 6.64 (m, 3H), 5.51 (s, 1H), 4.50 (d, *J* = 2.6 Hz, 1H), 3.19 (dd, *J* = 12.9, 2.5 Hz, 1H), 2.92 (dd, *J* = 12.9, 3.0 Hz, 1H); $^{19}$F NMR (565 MHz, Chloroform-*d*) δ -113.65; $^{13}$C NMR (75 MHz, Chloroform-*d*) δ 165.5, 162.4 (d, $J_{F\text{-}C}$ = 244.6 Hz), 150.1 (d, $J_{F\text{-}C}$ = 6.2 Hz), 146.3, 144.4, 142.0, 141.7, 135.8, 129.4, 129.1, 128.9 (d, $J_{F\text{-}C}$ = 8.3 Hz), 127.1, 126.1, 126.0, 126.0, 125.8, 125.5, 125.2, 125.0, 123.2 (d, $J_{F\text{-}C}$ = 2.5 Hz), 123.1, 122.4, 122.0, 114.7 (d, $J_{F\text{-}C}$ = 22.7 Hz), 112.4 (d, $J_{F\text{-}C}$ = 20.9 Hz), 56.2, 53.5, 44.9, 40.7; HRMS (ESI) m/z 428.1811 $(M + H)^+$, calc. for $C_{31}H_{23}FN$ 428.1809.

The ee was determined by HPLC analysis: IF*2 (4.6 mm i.d. x 250 mm); hexane/2–propanol = 99/01; flow rate 1.0 mL/min; 25 °C; 210 nm; retention time: 12.4 min (minor) and 13.1 min (major).

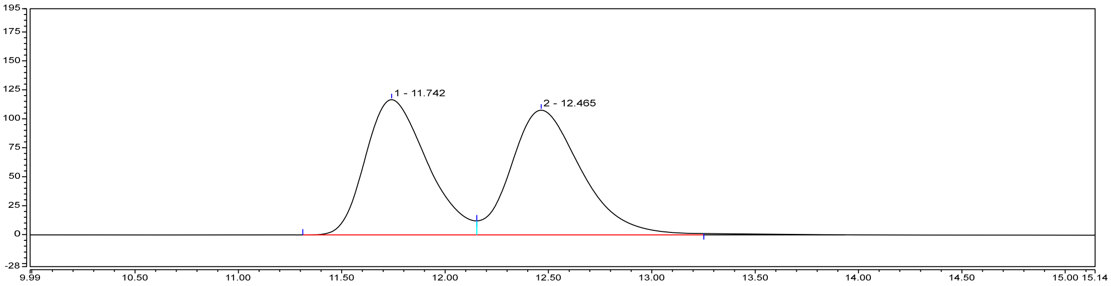


| Entry | Retention | Area | Height | %Area |
|---|---|---|---|---|
| 1 | 11.742 | 40.4009 | 116.71 | 48.84 |
| 2 | 12.465 | 42.3215 | 107.71 | 51.16 |

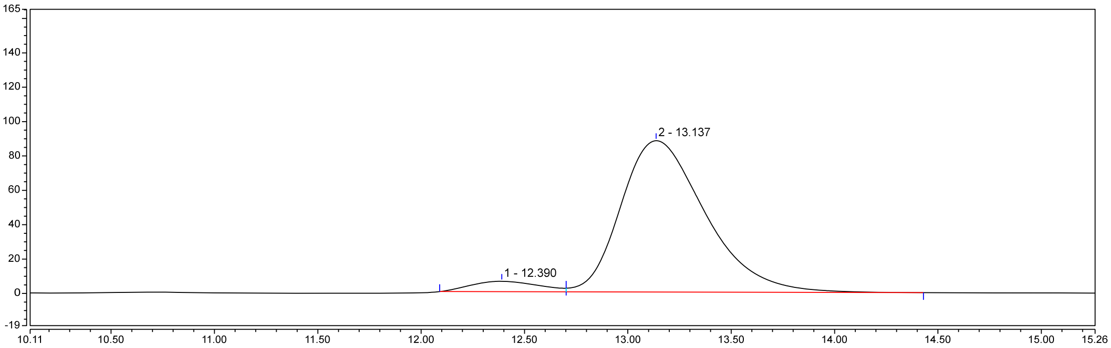


| Entry | Retention | Area | Height | %Area |
|---|---|---|---|---|
| 1 | 12.390 | 2.3096 | 6.05 | 5.24 |

| | | | | |
|---|---|---|---|---|
| 2 | 13.137 | 41.7306 | 88.08 | 94.76 |

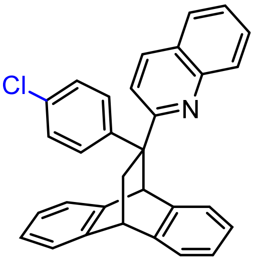


**3f:** White solid; Mp 140.1−142.0 ºC; 39.8 mg, 90% yield, 86% ee; $[\alpha]_D^{22}$ −18.7 (*c* 2.0, $CHCl_3$); $^1$H NMR (300 MHz, Chloroform-*d*) δ 8.13 (d, *J* = 8.4 Hz, 1H), 7.88 (d, *J* = 8.7 Hz, 1H), 7.70 (dd, *J* = 13.3, 7.6 Hz, 2H), 7.58 – 7.43 (m, 2H), 7.38 (d, *J* = 7.2 Hz, 1H), 7.26 – 7.20 (m, 1H), 7.12 (t, *J* = 8.3 Hz, 2H), 7.04 (d, *J* = 8.7 Hz, 3H), 7.01 – 6.83 (m, 5H), 5.53 (s, 1H), 4.53 (d, *J* = 2.7 Hz, 1H), 3.21 (dd, *J* = 12.9, 2.5 Hz, 1H), 2.94 (dd, *J* = 12.9, 3.0 Hz, 1H); $^{13}$C NMR (75 MHz, Chloroform-*d*) δ 165.7, 146.3, 145.9, 144.4, 144.3, 142.0, 141.7, 135.8, 131.4, 129.3, 129.1, 129.0, 127.7, 127.2, 126.1, 126.0, 126.0, 125.8, 125.5, 125.2, 125.0, 123.1, 122.4, 122.1, 55.9, 53.5, 44.9, 40.8; HRMS (ESI) m/z 444.1517 $(M + H)^+$, calc. for $C_{31}H_{23}ClN$ 444.1514.

The ee was determined by HPLC analysis: CHIRALPAK IF*2 (4.6 mm i.d. x 250 mm); hexane/2–propanol = 99/1; flow rate 1.0 mL/min; 25 ºC; 210 nm; retention time: 11.8 min (minor) and 12.4 min (major).

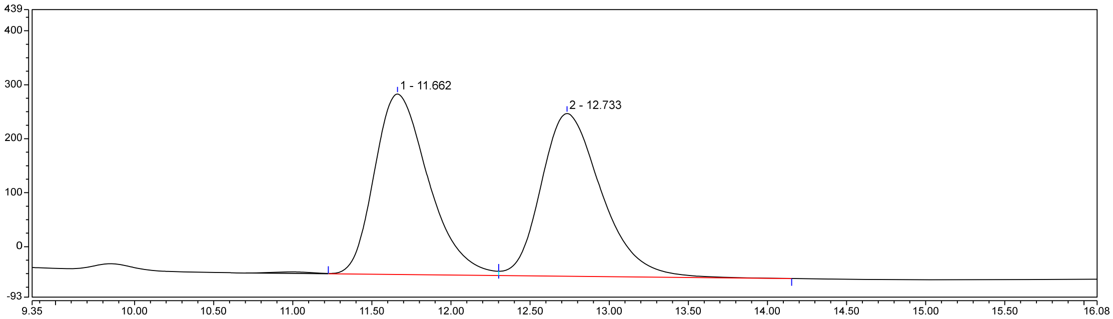


| Entry | Retention | Area | Height | %Area |
|---|---|---|---|---|
| 1 | 11.662 | 132.6773 | 334.34 | 49.82 |
| 2 | 12.733 | 133.6475 | 301.41 | 50.18 |

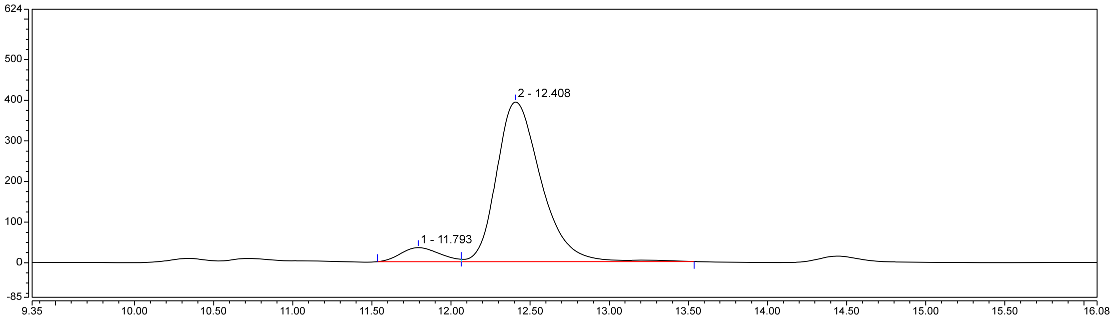


| Entry | Retention | Area | Height | %Area |
|---|---|---|---|---|
| 1 | 11.793 | 9.7505 | 34.44 | 7.10 |
| 2 | 12.408 | 127.6386 | 393.22 | 92.90 |

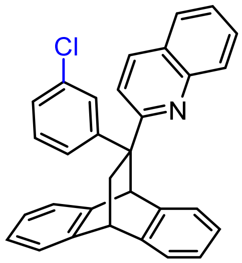


**3g:** White solid; Mp 145.3−147.1 °C; 42.0 mg, 95% yield, 90% ee; $[\alpha]_D^{22}$ –20.9 (*c* 2.0, $CHCl_3$); $^1H$ NMR (300 MHz, Chloroform-*d*) δ 8.10 (d, *J* = 8.4 Hz, 1H), 7.87 (d, *J* = 8.7 Hz, 1H), 7.71 – 7.65 (m, 2H), 7.48 – 7.44 (m, 2H), 7.37 (d, *J* = 7.2 Hz, 1H), 7.22 (d, *J* = 6.8 Hz, 1H), 7.15 (d, *J* = 8.4 Hz, 1H), 7.12 – 7.05 (m, 2H), 7.01 – 6.94 (m, 4H), 6.91 – 6.85 (m, 3H), 5.52 (s, 1H), 4.51 (s, 1H), 3.24 (dd, *J* = 12.8, 2.5 Hz, 1H), 2.90 (dd, *J* = 12.8, 3.0 Hz, 1H); $^{13}C$ NMR (75 MHz, Chloroform-*d*) δ 165.4, 149.5, 146.4, 144.4, 144.3, 141.9, 141.6, 135.8, 133.6, 129.4, 129.1, 128.8, 127.9, 127.1, 127.1, 126.1, 126.1, 125.8, 125.7, 125.5, 125.3, 125.0, 123.1, 122.5, 122.0, 56.2, 53.4, 44.8, 40.7; HRMS (ESI) m/z 444.1516 $(M + H)^+$, calc. for $C_{31}H_{23}ClN$ 444.1514.

The ee was determined by HPLC analysis: CHIRALPAK IF*2 (4.6 mm i.d. x 250 mm); hexane/2–propanol = 99/01; flow rate 1.0 mL/min; 25 °C; 210 nm; retention time: 13.0 min (minor) and 14.0 min (major).

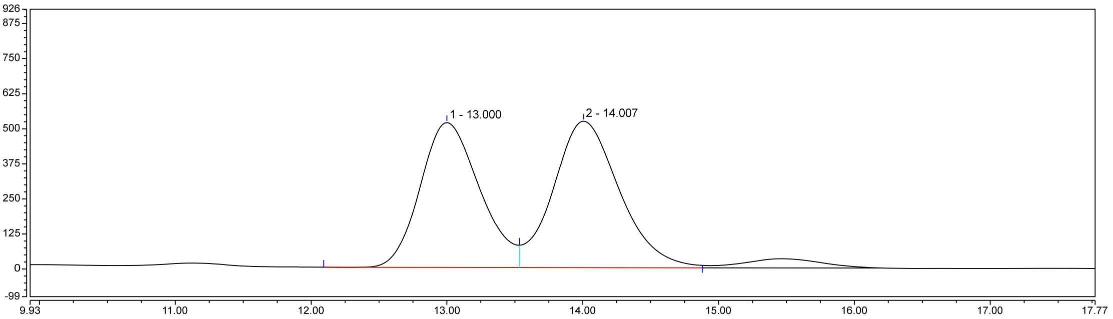


| Entry | Retention | Area | Height | %Area |
|---|---|---|---|---|
| 1 | 13.000 | 262.1476 | 516.01 | 47.11 |
| 2 | 14.007 | 294.2952 | 522.09 | 52.89 |

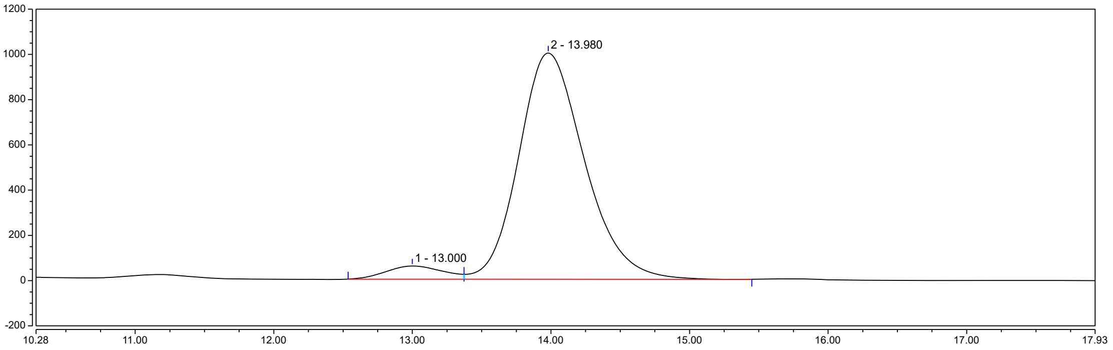


| Entry | Retention | Area | Height | %Area |
|---|---|---|---|---|
| 1 | 13.000 | 27.5062 | 58.05 | 4.77 |
| 2 | 13.980 | 548.9626 | 999.63 | 95.23 |

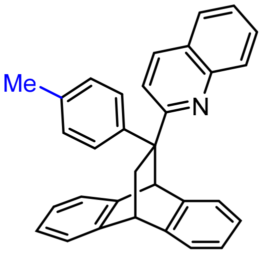


**3h:** White solid; Mp 207.5−209.1 °C; 28.7 mg, 68% yield, 89% ee; $[\alpha]_D^{22}$ –6.7 ($c$ 2.0, $CHCl_3$); $^1$H NMR (300 MHz, Chloroform-$d$) δ 8.06 (d, $J$ = 8.4 Hz, 1H), 7.83 (d, $J$ = 8.7 Hz, 1H), 7.64 (d, $J$ = 8.1 Hz, 2H), 7.43 (t, $J$ = 7.0 Hz, 2H), 7.32 (d, $J$ = 7.3 Hz, 1H), 7.17 (dd, $J$ = 11.5, 7.8 Hz, 2H), 7.07 (t, $J$ = 7.4 Hz, 2H), 6.95 (d, $J$ = 7.5 Hz, 1H), 6.90 – 6.81 (m, 6H), 5.50 (s, 1H), 4.47 (d, $J$ = 2.8 Hz, 1H), 3.25 (dd, $J$ = 12.8, 2.6 Hz, 1H), 2.90 (dd, $J$ = 12.8, 2.9 Hz, 1H), 2.17 (s, 3H); $^{13}$C NMR (75 MHz, Chloroform-$d$) δ 166.4, 146.3, 144.7, 144.5, 144.4, 142.4, 142.1, 135.5, 134.9, 129.4, 128.9, 128.4, 127.5, 127.1, 127.0, 126.2, 126.0, 125.8, 125.5, 125.3, 125.1, 124.8, 123.0, 122.4, 122.2, 55.9, 53.4, 45.0, 40.6, 20.8; HRMS (ESI) m/z 424.2064 $(M + H)^+$, calc. for $C_{32}H_{26}N$ 424.206.

The ee was determined by HPLC analysis: CHIRALPAK IF*2 (4.6 mm i.d. x 250 mm); hexane/2–propanol = 98/2; flow rate 1.0 mL/min; 25 °C; 210 nm; retention time: 12.1 min (minor) and 13.1 min (major).

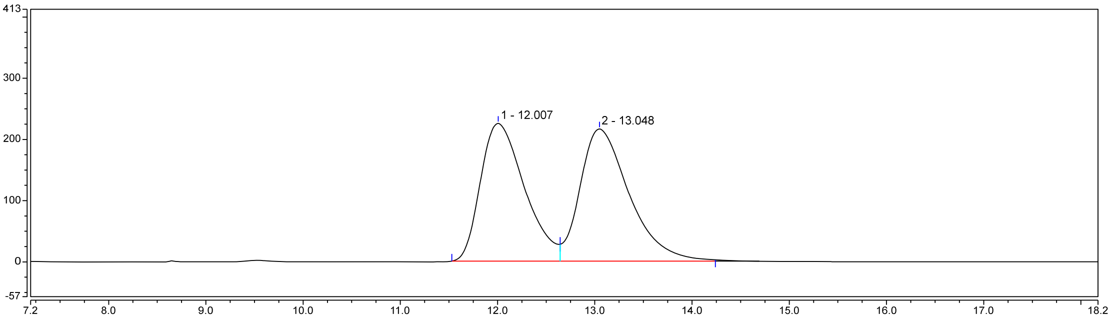


| Entry | Retention | Area | Height | %Area |
|---|---|---|---|---|
| 1 | 12.007 | 118.0229 | 225.07 | 48.29 |
| 2 | 13.048 | 126.3599 | 215.96 | 51.71 |

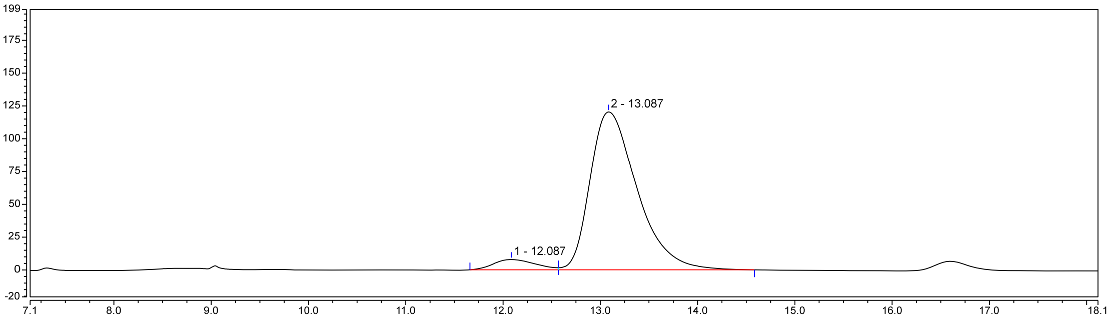


| Entry | Retention | Area | Height | %Area |
|---|---|---|---|---|
| 1 | 12.087 | 3.9326 | 7.91 | 5.51 |
| 2 | 13.087 | 67.4567 | 120.43 | 94.49 |

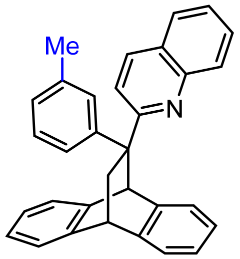

**3i:** White solid; Mp 197.2−198.7 °C; 30.4 mg, 72% yield, 90% ee; $[\alpha]_D^{22}$ –5.9 (*c* 2.0, $CHCl_3$); $^1H$ NMR (300 MHz, Chloroform-*d*) δ 8.11 (d, *J* = 8.4 Hz, 1H), 7.87 (d, *J* = 8.6 Hz, 1H), 7.69 (t, *J* = 8.5 Hz, 2H), 7.46 (t, *J* = 6.2 Hz, 2H), 7.38 (d, *J* = 7.3 Hz, 1H), 7.27 – 7.19 (m, 2H), 7.11 (t, *J* = 7.8 Hz, 2H), 6.99 (t, *J* = 7.8 Hz, 2H), 6.94 – 6.77 (m, 5H), 5.54 (s, 1H), 4.53 (s, 1H), 3.34 (d, *J* = 12.7 Hz, 1H), 2.94 (d, *J* = 12.6 Hz, 1H), 2.17 (s, 3H); $^{13}C$ NMR (75 MHz, Chloroform-*d*) δ 166.2, 147.3, 146.3, 144.8, 144.5, 142.3, 142.1, 137.0, 135.5, 129.4, 128.8, 128.6, 127.4, 127.1, 126.9, 126.2, 126.2, 126.0, 125.8, 125.5, 125.2, 125.1, 124.8, 124.6, 123.0, 122.4, 122.2, 56.2, 53.5, 45.0, 40.6, 21.4; HRMS (ESI) m/z 424.2061 $(M + H)^+$, calc. for $C_{32}H_{26}N$ 424.206.

The ee was determined by HPLC analysis: CHIRALPAK IF*2 (4.6 mm i.d. x 250 mm); hexane/2–propanol = 99/1; flow rate 1.0 mL/min; 25 °C; 254 nm; retention time: 9.9 min (minor) and 11.1 min (major).

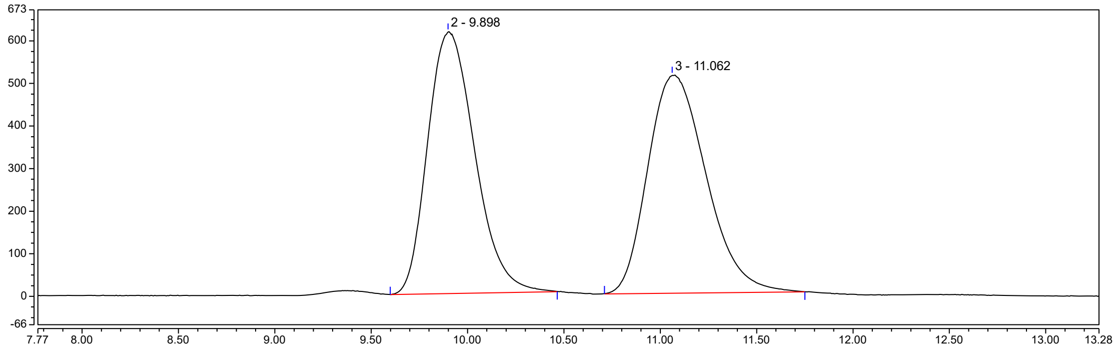

| Entry | Retention | Area | Height | %Area |
|---|---|---|---|---|
| 1 | 9.898 | 174.4366 | 615.82 | 49.24 |
| 2 | 11.062 | 179.8388 | 513.15 | 50.76 |

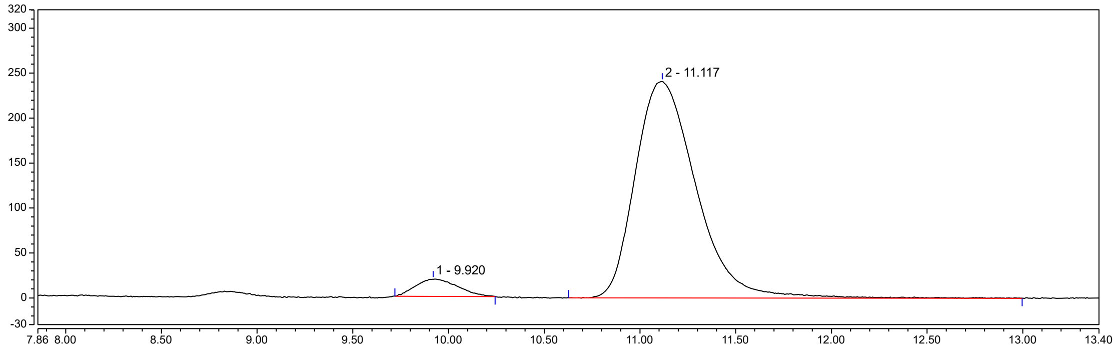

| Entry | Retention | Area | Height | %Area |
|---|---|---|---|---|
| 1 | 9.920 | 4.9372 | 19.27 | 5.28 |
| 2 | 11.117 | 88.6345 | 240.96 | 94.72 |

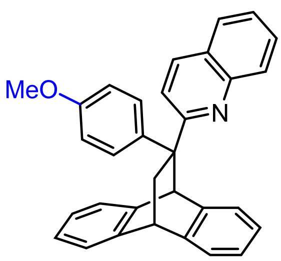


**3j:** White solid; Mp 183.3−185.2 °C; 31.6 mg, 72% yield, 90% ee; $[\alpha]_D^{22}$ −14.3 (*c* 2.0, $CHCl_3$); $^1H$ NMR (300 MHz, Chloroform-*d*) δ 8.08 (d, *J* = 8.4 Hz, 1H), 7.85 (d, *J* = 8.6 Hz, 1H), 7.66 (t, *J* = 8.7 Hz, 2H), 7.48 – 7.41 (m, 2H), 7.34 (d, *J* = 7.2 Hz, 1H), 7.23 – 7.12 (m, 2H), 7.09 – 7.02 (m, 2H), 6.96 (d, *J* = 7.3 Hz, 1H), 6.91 – 6.83 (m, 4H), 6.57 (d, *J* = 8.6 Hz, 2H), 5.49 (s, 1H), 4.48 (d, *J* = 2.8 Hz, 1H), 3.66 (s, 3H), 3.18 (dd, *J* = 12.9, 2.5 Hz, 1H), 2.92 (dd, *J* = 12.9, 3.0 Hz, 1H); $^{13}C$ NMR (75 MHz, Chloroform-*d*) δ 166.5, 157.1, 146.2, 144.5, 144.5, 142.4, 142.2, 139.5, 135.5, 129.3, 128.9, 128.6, 127.1, 126.2, 126.0, 125.8, 125.5, 125.3, 125.1, 124.9, 123.0, 122.4, 122.3, 112.9, 55.5, 55.0, 53.6, 45.0, 40.8; HRMS (ESI) m/z 440.2012 $(M + H)^+$, calc. for $C_{32}H_{26}NO$ 440.2009.

The ee was determined by HPLC analysis: CHIRALPAK Amylose-1 * Cellulose-4 (4.6 mm i.d. x 250 mm); hexane/2–propanol = 99/1; flow rate 1.0 mL/min; 25 °C; 254 nm; retention time: 50.8 min (major) and 54.3 min (minor).

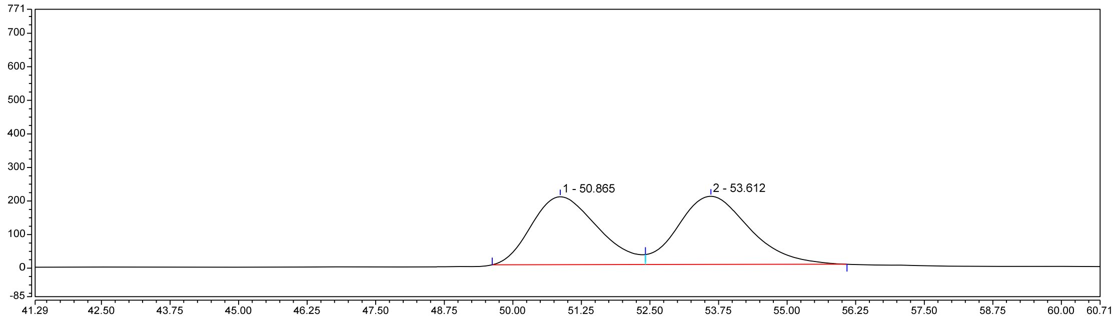


| Entry | Retention | Area | Height | %Area |
|---|---|---|---|---|
| 1 | 50.865 | 283.8959 | 202.24 | 48.07 |
| 2 | 53.612 | 306.6500 | 202.91 | 51.93 |

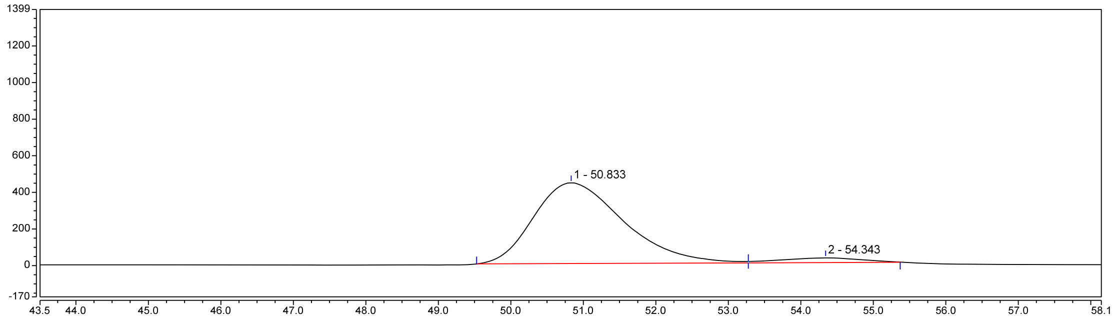


| Entry | Retention | Area | Height | %Area |
|---|---|---|---|---|
| 1 | 50.833 | 623.7312 | 437.03 | 94.78 |
| 2 | 54.343 | 34.3637 | 26.89 | 5.22 |

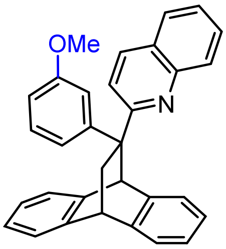


**3k:** White solid; Mp 190.5−191.7 °C; 33.4 mg, 76% yield, 90% ee; $[\alpha]_D^{22}$ −13.8 (*c* 2.0, $CHCl_3$); $^1$H NMR (300 MHz, Chloroform-*d*) δ 8.09 (d, *J* = 8.3 Hz, 1H), 7.86 (d, *J* = 8.7 Hz, 1H), 7.67 (t, *J* = 8.2 Hz, 2H), 7.45 (t, *J* = 7.1 Hz, 2H), 7.37 (d, *J* = 7.2 Hz, 1H), 7.20 (t, *J* = 8.5 Hz, 2H), 7.10 (t, *J* = 8.1 Hz, 2H), 6.98 (t, *J* = 7.8 Hz, 2H), 6.91 – 6.83 (m, 2H), 6.67 – 6.52 (m, 3H), 5.52 (s, 1H), 4.51 (s, 1H), 3.61 (s, 3H), 3.28 (dd, *J* = 12.9, 2.5 Hz, 1H), 2.93 (dd, *J* = 12.9, 2.9 Hz, 1H); $^{13}$C NMR (75 MHz, Chloroform-*d*) δ 165.9, 158.9, 149.0, 146.3, 144.7, 144.5, 142.2, 142.0, 135.6, 129.4, 128.9, 128.4, 127.1, 127.0, 126.2, 126.0, 125.8, 125.6, 125.4, 125.1, 124.9, 123.0, 122.4, 122.1, 120.4, 113.8, 110.7, 56.2, 55.0, 53.5, 45.0, 40.6; HRMS (ESI) m/z 440.2011 $(M + H)^+$, calc. for $C_{32}H_{26}NO$ 440.2009.

The ee was determined by HPLC analysis: CHIRALPAK Amylose-1 * Cellulose-4 (4.6 mm i.d. x 250 mm); hexane/2–propanol = 99/1; flow rate 1.0 mL/min; 25 °C; 254 nm; retention time: 29.3 min (major) and 31.9 min (minor).

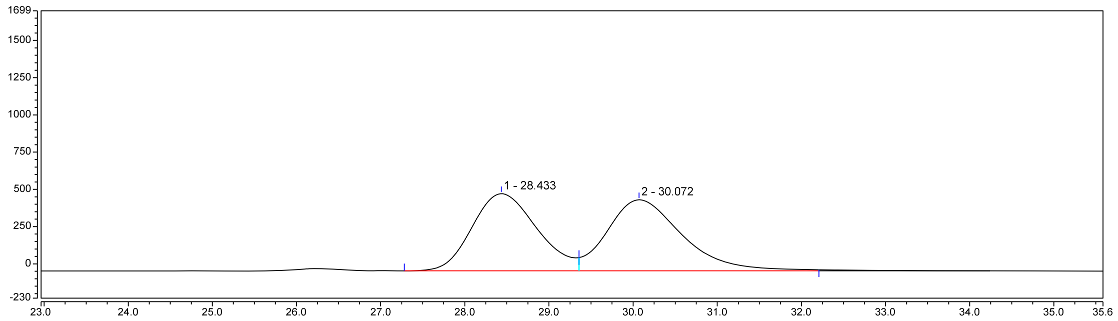


| Entry | Retention | Area | Height | %Area |
|---|---|---|---|---|
| 1 | 28.433 | 461.7740 | 517.38 | 48.54 |
| 2 | 30.072 | 489.6422 | 476.54 | 51.46 |

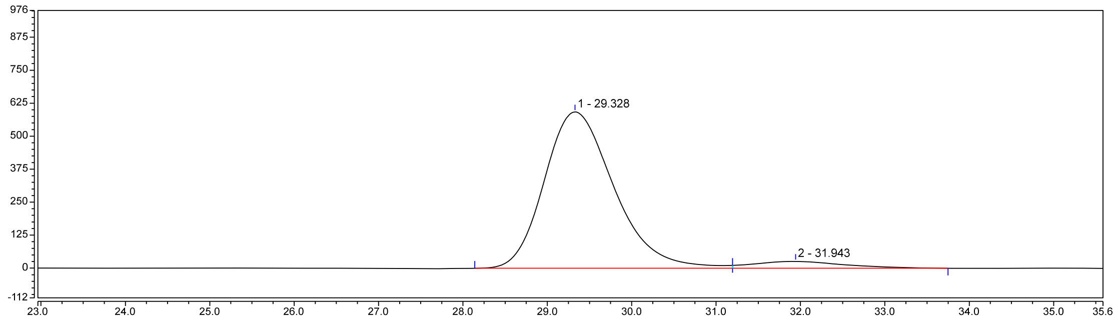


| Entry | Retention | Area | Height | %Area |
|---|---|---|---|---|
| 1 | 29.328 | 567.1023 | 592.46 | 94.74 |
| 2 | 31.943 | 31.4700 | 25.86 | 5.26 |

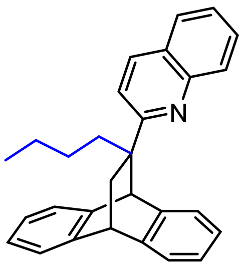

**3l:** Pale yellow oil; 19.8 mg, 51% yield, 83% ee; $[\alpha]_D^{22}$ +18.1 (*c* 2.0, $CHCl_3$); $^1$H NMR (300 MHz, Chloroform-*d*) δ 8.01 (d, *J* = 8.4 Hz, 1H), 7.94 (d, *J* = 8.7 Hz, 1H), 7.69 (d, *J* = 8.1 Hz, 1H), 7.62 (d, *J* = 7.9 Hz, 1H), 7.42 (d, *J* = 6.3 Hz, 2H), 7.34 (t, *J* = 7.3 Hz, 2H), 7.17 – 7.10 (m, 4H), 6.86 (t, *J* = 7.4 Hz, 1H), 6.75 (t, *J* = 7.4 Hz, 1H), 5.02 (s, 1H), 4.37 (s, 1H), 3.03 (d, *J* = 12.4 Hz, 1H), 1.89 (d, *J* = 11.1 Hz, 1H), 1.63 – 1.55 (m, 1H), 1.40 – 1.18 (m, 2H), 1.03 – 0.92 (m, 3H), 0.64 (t, *J* = 6.9 Hz, 3H); $^{13}$C NMR (75 MHz, Chloroform-*d*) δ 165.8, 146.8, 144.3, 144.0, 142.4, 142.2, 135.2, 129.3, 128.7, 127.1, 126.0, 125.8, 125.6, 125.5, 125.3, 125.0, 124.8, 123.0, 122.7, 120.2, 52.4, 51.5, 45.0, 43.8, 39.9, 26.6, 23.0, 13.9; HRMS (ESI) m/z 390.2215 $(M + H)^+$, calc. for $C_{29}H_{28}N$ 390.2216.

The ee was determined by HPLC analysis: CHIRALPAK IF*IG (4.6 mm i.d. x 250 mm); hexane/2–propanol = 99/1; flow rate 1.0 mL/min; 25 °C; 210 nm; retention time: 9.1 min (minor) and 9.6 min (major).

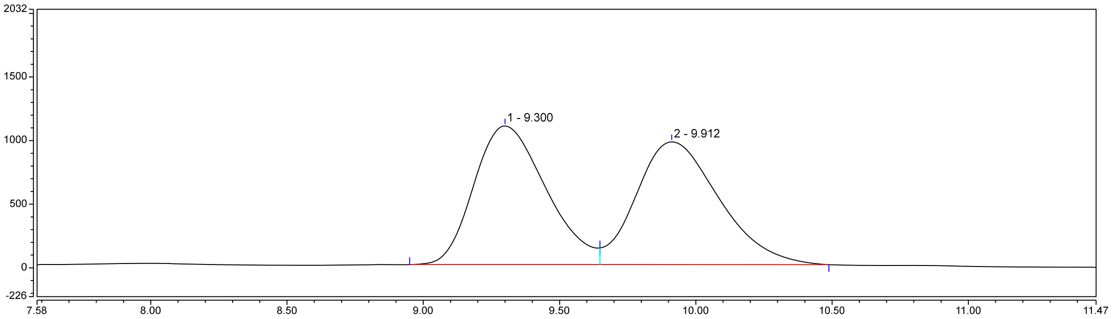


| Entry | Retention | Area | Height | %Area |
|---|---|---|---|---|
| 1 | 9.300 | 333.9850 | 1089.00 | 49.56 |
| 2 | 9.912 | 339.9133 | 965.62 | 50.44 |

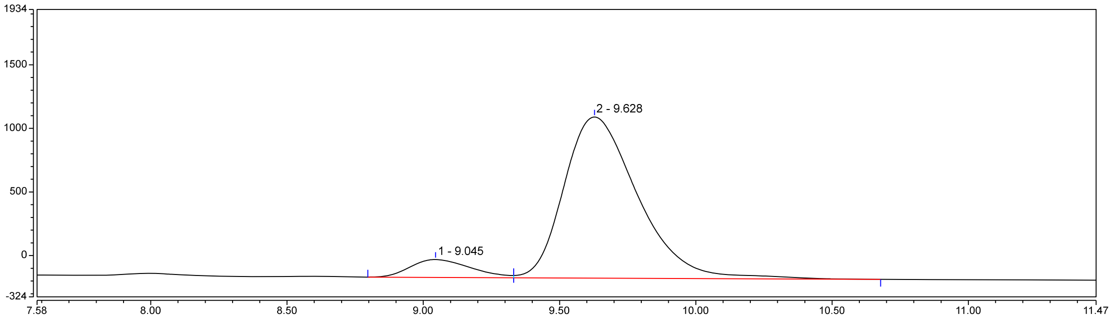


| Entry | Retention | Area | Height | %Area |
|---|---|---|---|---|
| 1 | 9.045 | 35.8813 | 140.82 | 8.28 |
| 2 | 9.628 | 397.7182 | 1266.03 | 91.72 |

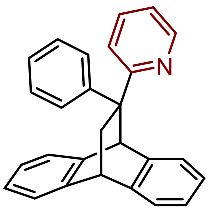

**3m:** White solid; Mp 143.5−145.2 °C; 21.2 mg, 59% yield, 92% ee; $[\alpha]_D^{22}$ +8.6 (*c* 2.0, $CHCl_3$); $^1$H NMR (300 MHz, Methylene Chloride-$d_2$) δ 8.32 (d, *J* = 4.9 Hz, 1H), 7.45 (td, *J* = 7.8, 1.9 Hz, 1H), 7.32 – 7.18 (m, 6H), 7.12 (t, *J* = 7.7 Hz, 3H), 7.07 – 6.95 (m, 4H), 6.89 (q, *J* = 7.9, 7.1 Hz, 2H), 5.44 (s, 1H), 4.46 (d, *J* = 2.9 Hz, 1H), 3.46 (dd, *J* = 12.9, 2.9 Hz, 1H), 2.70 (dd, *J* = 12.9, 2.7 Hz, 1H); $^{13}$C NMR (75 MHz, Methylene Chloride-$d_2$) δ 166.9, 148.8, 148.1, 145.7, 145.0, 142.5, 142.1, 136.3, 128.2, 128.1, 126.7, 126.5, 126.1, 125.8, 125.6, 125.4, 123.4, 123.3, 120.7, 56.2, 53.3, 45.4, 40.9; HRMS (ESI) m/z 360.1746 $(M + H)^+$, calc. for $C_{27}H_{22}N$ 360.1747.

The ee was determined by HPLC analysis: CHIRALPAK IF (4.6 mm i.d. x 250 mm); hexane/2–propanol = 95/5; flow rate 1.0 mL/min; 25 °C; 210 nm; retention time: 5.5 min (minor) and 6.3 min (major).

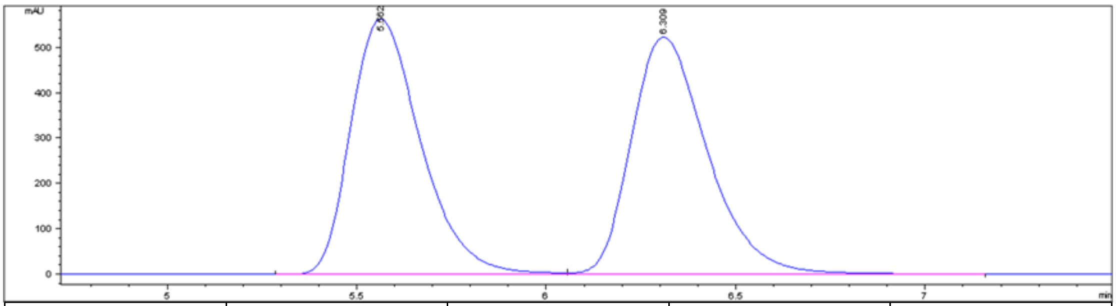

| Entry | Retention | Area | Height | %Area |
|---|---|---|---|---|
| 1 | 5.562 | 7248.3 | 564.2 | 49.625 |
| 2 | 6.309 | 7357.8 | 523.3 | 50.375 |

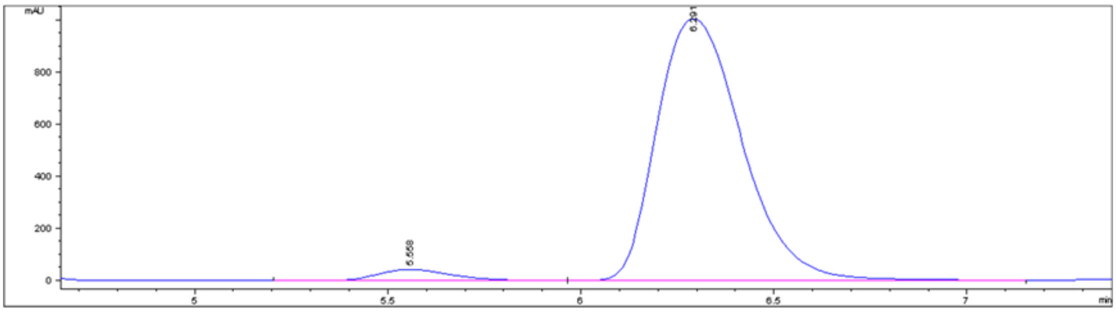

| Entry | Retention | Area | Height | %Area |
|---|---|---|---|---|
| 1 | 5.558 | 615.6 | 43.9 | 3.825 |
| 2 | 6.291 | 15480.4 | 1007 | 96.175 |

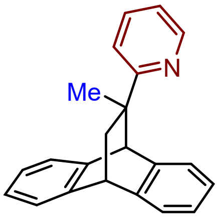


**3n:** White solid; Mp 138.6−139.8 °C; 21.4 mg, 72% yield, 85% ee; $[\alpha]_D^{22}$ +25.9 (*c* 2.0, $CHCl_3$); $^1$H NMR (300 MHz, Chloroform-*d*) δ 8.46 (d, *J* = 3.1 Hz, 1H), 7.48 – 7.36 (m, 2H), 7.34 – 7.29 (m, 1H), 7.23 (d, *J* = 7.2 Hz, 1H), 7.15 (q, *J* = 5.1, 4.1 Hz, 2H), 7.03 (d, *J* = 8.1 Hz, 1H), 7.00 – 6.89 (m, 3H), 6.89 – 6.79 (m, 1H), 4.63 (s, 1H), 4.36 (d, *J* = 2.8 Hz, 1H), 2.87 (dd, *J* = 12.7, 2.9 Hz, 1H),

1.78 (dd, $J$ = 12.7, 2.6 Hz, 1H), 1.16 (s, 3H); $^{13}$C NMR (75 MHz, Chloroform-$d$) δ 167.3, 148.1, 143.7, 143.3, 142.3, 142.1, 135.7, 125.9, 125.8, 125.4, 125.4, 125.0, 123.0, 122.9, 120.9, 120.4, 54.9, 47.2, 45.0, 40.6, 31.4; HRMS (ESI) m/z 298.1593 $(M + H)^+$, calc. for $C_{22}H_{20}N$ 298.159. The ee was determined by HPLC analysis: CHIRALPAK Amylose-1 (4.6 mm i.d. x 250 mm); hexane/2–propanol = 97/3; flow rate 1.0 mL/min; 25 °C; 210 nm; retention time: 5.3 min (major) and 9.1 min (minor).

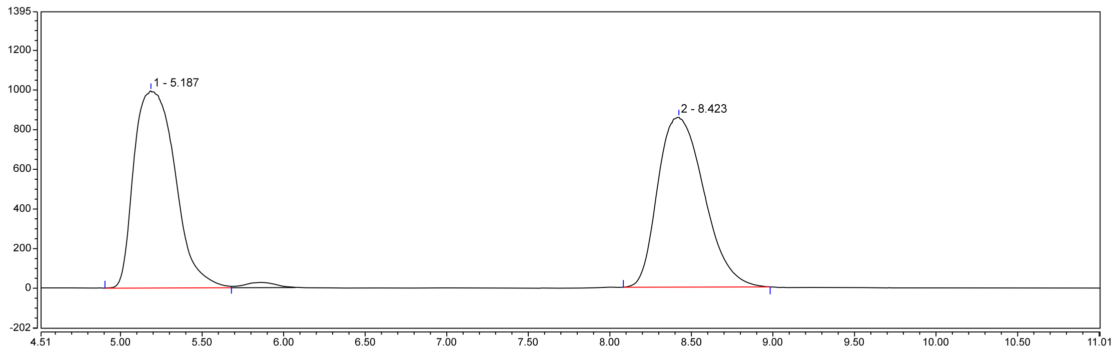


| Entry | Retention | Area | Height | %Area |
|---|---|---|---|---|
| 1 | 5.187 | 291.5286 | 995.44 | 50.78 |
| 2 | 8.423 | 282.6185 | 858.45 | 49.22 |

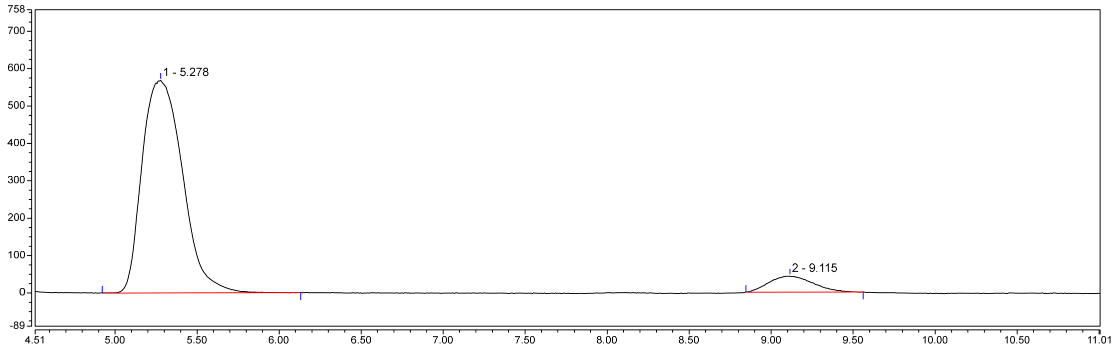


| Entry | Retention | Area | Height | %Area |
|---|---|---|---|---|
| 1 | 5.278 | 162.6182 | 568.82 | 92.45 |
| 2 | 9.115 | 13.2810 | 42.90 | 7.55 |

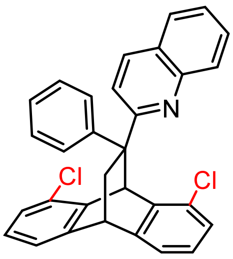


**3o:** Pale yellow oil; 33.4 mg, 70% yield, 93% ee, > 20:1 rr; $[\alpha]_D^{22}$ –19.9 ($c$ 2.0, $CHCl_3$); $^1$H NMR (300 MHz, Chloroform-$d$) δ 8.16 (d, $J$ = 8.7 Hz, 1H), 7.88 (d, $J$ = 8.7 Hz, 1H), 7.66 – 7.62 (m, 2H), 7.44 (t, $J$ = 7.5 Hz, 1H), 7.25 – 7.18 (m, 2H), 7.15 – 7.10 (m, 4H), 7.08 –7.02 (m, 2H), 7.01 – 6.97 (m, 2H), 6.93 (d, $J$ = 8.0 Hz, 1H), 6.84 (t, $J$ = 7.6 Hz, 1H), 6.33 (s, 1H), 4.52 (d, $J$ = 3.0 Hz, 1H), 3.48 (dd, $J$ = 12.9, 2.7 Hz, 1H), 2.88 (dd, $J$ = 12.9, 2.9 Hz, 1H); $^{13}$C NMR (75 MHz, Chloroform-$d$) δ 165.3, 147.0, 146.5, 146.4, 146.1, 139.6, 139.1, 135.6, 131.7, 131.6, 129.6, 128.9, 127.8, 127.7, 127.1, 127.0, 126.5, 126.3, 126.2, 126.2, 126.0, 125.9,

122.3, 121.7, 121.4, 56.9, 46.4, 45.6, 39.6; HRMS (ESI) m/z 478.1128 $(M + H)^+$, calc. for $C_{31}H_{22}Cl_2N$ 478.1124.

The ee was determined by HPLC analysis: (Amylose-1) *2 (4.6 mm i.d. x 250 mm); hexane/2–propanol = 97/3; flow rate 1.0 mL/min; 25 ºC; 210 nm; retention time: 11.1 min (minor) and 12.3 min (major).

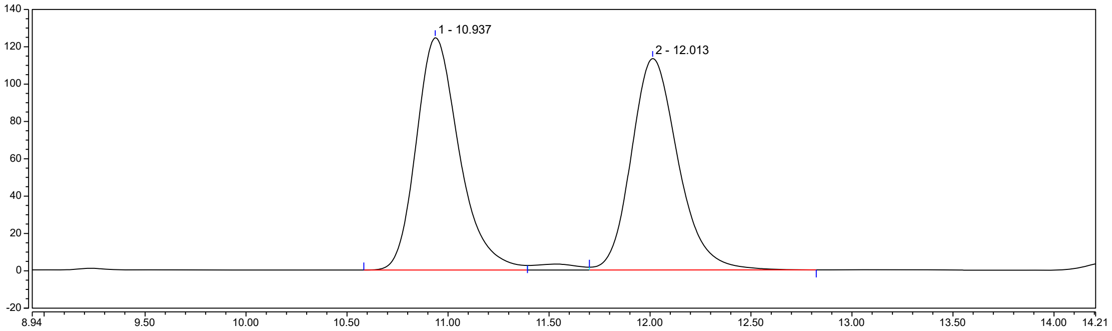

| Entry | Retention | Area | Height | %Area |
|---|---|---|---|---|
| 1 | 10.937 | 30.7860 | 124.41 | 50.34 |
| 2 | 12.013 | 30.3746 | 113.21 | 49.66 |

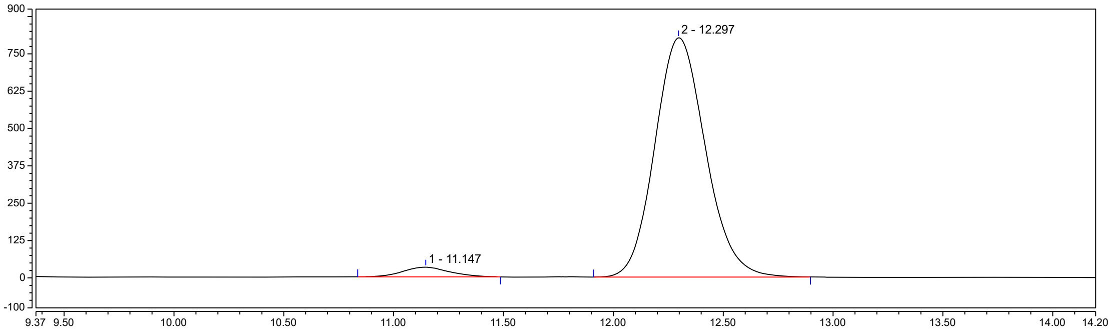

| Entry | Retention | Area | Height | %Area |
|---|---|---|---|---|
| 1 | 11.147 | 8.1370 | 32.39 | 3.66 |
| 2 | 12.297 | 213.9448 | 800.38 | 96.34 |

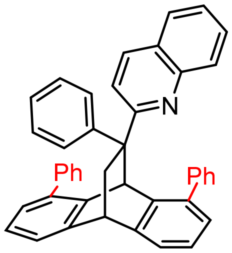


**3p:** Pale yellow solid; 43.7 mg, 78% yield, 90% ee, > 20:1 rr; $[\alpha]_D^{22}$ –88.7 (*c* 2.0, $CHCl_3$); $^1H$ NMR (300 MHz, Chloroform-*d*) δ 7.76 (t, *J* = 8.5 Hz, 2H), 7.68 (d, *J* = 8.0 Hz, 1H), 7.58 (d, *J* = 7.2 Hz, 1H), 7.51 (d, *J* = 7.7 Hz, 1H), 7.44 (d, *J* = 7.7 Hz, 1H), 7.40 (s, 1H), 7.34 (d, *J* = 6.6 Hz, 1H), 7.30 (d, *J* = 7.0 Hz, 2H), 7.26 – 7.20 (m, 2H), 7.17 (d, *J* = 12.5 Hz, 1H), 7.13 (s, 1H), 7.09 (d, *J* = 5.6 Hz, 1H), 7.07 – 6.94 (m, 6H), 6.93 – 6.85 (m, 2H), 6.47 – 6.42 (m, 4H), 5.74 (s, 1H), 4.69 (s, 1H), 3.73 (d, *J* = 13.4 Hz, 1H), 3.12 (d, *J* = 13.3 Hz, 1H); $^{13}C$ NMR (75 MHz, Chloroform-*d*) δ 166.3, 147.7, 147.0, 145.8, 145.1, 140.7, 140.6, 139.8, 139.7, 139.0, 138.8, 134.6, 129.7, 129.7, 129.2, 128.6, 128.4, 128.3, 128.0, 127.9, 127.7, 127.5, 127.2, 126.9,

126.7, 126.5, 126.2, 125.9, 125.5, 123.0, 122.6, 122.5, 57.4, 46.8, 46.6, 43.0; HRMS (ESI) m/z 562.2531 $(M + H)^+$, calc. for $C_{43}H_{32}N$ 562.2529.

The ee was determined by HPLC analysis: Amylose-1 (4.6 mm i.d. x 250 mm); hexane/2–propanol = 97/3; flow rate 1.0 mL/min; 25 °C; 210 nm; retention time: 5.3 min (minor) and 7.6 min (major).

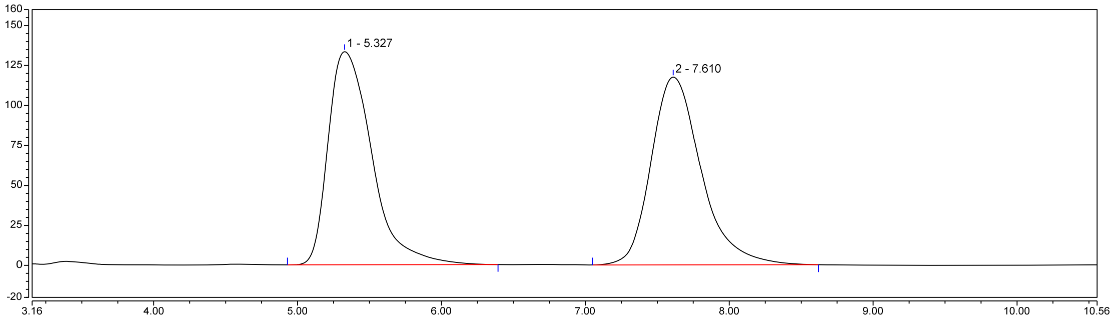


| Entry | Retention | Area | Height | %Area |
|---|---|---|---|---|
| 1 | 5.327 | 46.9229 | 133.37 | 49.76 |
| 2 | 7.610 | 47.3806 | 117.46 | 50.24 |

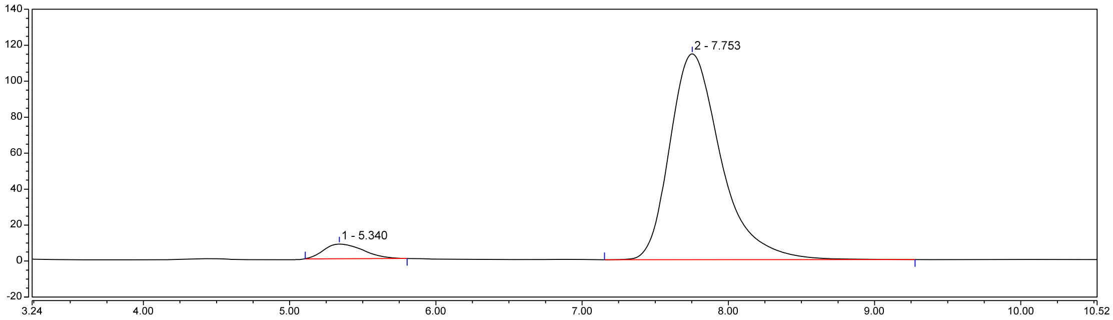


| Entry | Retention | Area | Height | %Area |
|---|---|---|---|---|
| 1 | 5.340 | 2.5513 | 8.15 | 5.22 |
| 2 | 7.753 | 46.2934 | 114.40 | 94.78 |

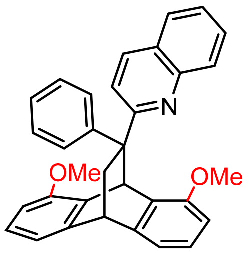


**3q:** Pale yellow solid; 40.8 mg, 87% yield, 97% ee, > 20:1 rr; $[\alpha]_D^{22}$ +53.7 (*c* 2.0, $CHCl_3$); $^1H$ NMR (300 MHz, Chloroform-*d*) δ 7.88 (t, *J* = 8.5 Hz, 2H), 7.64 (d, *J* = 8.1 Hz, 2H), 7.60 – 7.54 (m, 1H), 7.50 (d, *J* = 8.5 Hz, 2H), 7.40 (t, *J* = 7.6 Hz, 1H), 7.14 (t, *J* = 7.8 Hz, 2H), 7.04 (q, *J* = 7.7 Hz, 2H), 7.00 – 6.91 (m, 2H), 6.85 (t, *J* = 7.7 Hz, 1H), 6.66 (d, *J* = 8.1 Hz, 1H), 6.34 (s, 1H), 6.27 (d, *J* = 8.2 Hz, 1H), 4.50 (t, *J* = 2.7 Hz, 1H), 4.28 (dd, *J* = 12.5, 3.2 Hz, 1H), 3.91 (s, 3H), 3.57 (s, *J* = 1.5 Hz, 3H), 2.47 (dd, *J* = 12.5, 2.4 Hz, 1H); $^{13}C$ NMR (75 MHz, Chloroform-*d*) δ 165.5, 155.2, 154.3, 148.5, 148.0, 146.5, 146.1, 134.5, 129.6, 129.4, 129.2, 128.4, 127.9, 127.7, 126.8, 126.5, 126.2, 126.0, 125.5, 125.3, 121.8, 116.2, 115.9, 108.6,

107.9, 57.7, 55.7, 55.2, 45.3, 40.0, 38.0; HRMS (ESI) m/z 470.2117 $(M + H)^+$, calc. for $C_{33}H_{28}NO_2$ 470.2115.

The ee was determined by HPLC analysis: Amylose-1* i-Amylose-1 (4.6 mm i.d. x 250 mm); hexane/2–propanol = 95/5; flow rate 1.0 mL/min; 25 ºC; 210 nm; retention time: 13.2 min (minor) and 18.6 min (major).

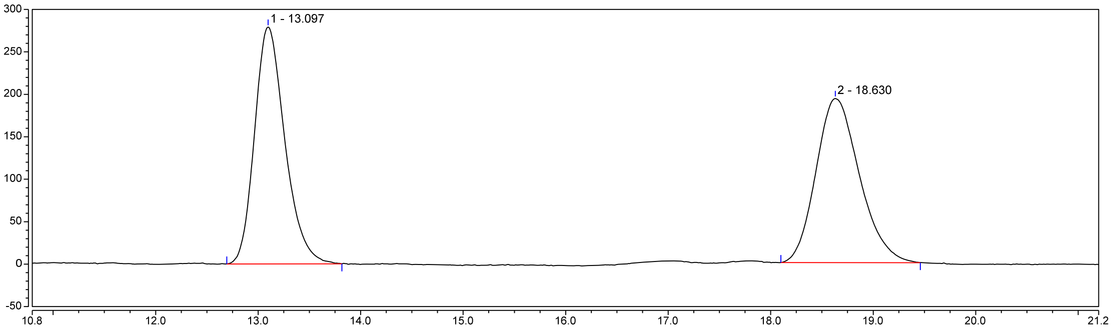


| Entry | Retention | Area | Height | %Area |
|---|---|---|---|---|
| 1 | 13.097 | 95.7554 | 278.98 | 50.20 |
| 2 | 18.630 | 94.9758 | 193.37 | 49.80 |

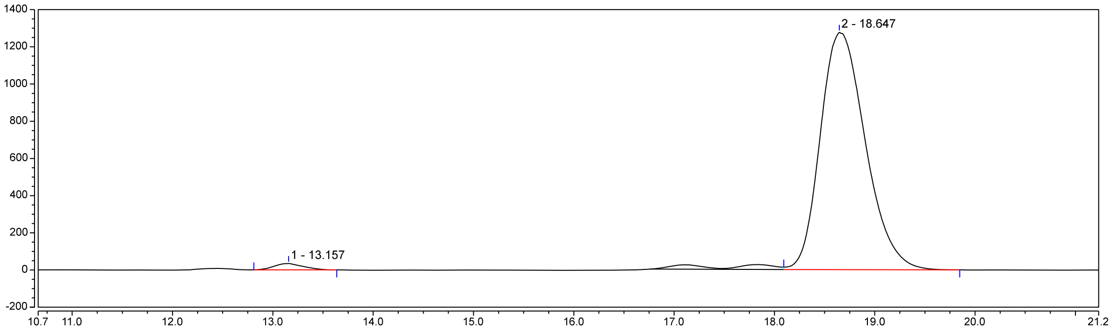


| Entry | Retention | Area | Height | %Area |
|---|---|---|---|---|
| 1 | 13.157 | 11.5742 | 34.41 | 1.71 |
| 2 | 18.647 | 663.9890 | 1275.64 | 98.29 |

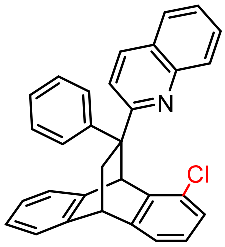


**3r**: White solid; Mp 188.2−190.3 ºC; 31.0 mg, 70% yield, 89%/88% ee; 2.5:1 dr; > 20:1 rr; $[\alpha]^{22}_{D}$ −17.4 (*c* 2.0, $CHCl_3$); $^1$H NMR (300 MHz, Chloroform-*d*) δ 8.20 (d, *J* = 8.6 Hz, 1H), 7.88 (d, *J* = 8.1 Hz, 1H), 7.67 (t, *J* = 7.0 Hz, 2H), 7.60 – 7.31 (m, 3H), 7.24 – 7.00 (m, 9H), 6.93 – 6.81 (m, 2H), 5.88 (s, 1H), 4.54 (s, 1H), 3.56 (d, *J* = 12.7 Hz, 1H), 2.87 (d, *J* = 12.8 Hz, 1H); $^{13}$C NMR (75 MHz, Chloroform-*d*) δ 165.5, 147.2, 146.5, 144.7, 141.1, 140.1, 135.5, 131.2, 129.6, 128.8, 127.7, 127.6, 127.6, 127.0, 126.5, 126.3, 126.0, 125.9, 125.8, 125.7, 125.5, 123.0, 122.3, 121.4, 56.7, 49.9, 45.3, 40.0; HRMS (ESI) m/z 444.1518 $(M + H)^+$, calc. for $C_{31}H_{23}ClN$ 444.1514.

The ee was determined by HPLC analysis: Amylose-1*2 (4.6 mm i.d. x 250 mm); hexane/2–

propanol = 98/2; flow rate 1.0 mL/min; 25 °C; 210 nm; retention time: 11.3 min (minor) and 14.7 min (major).

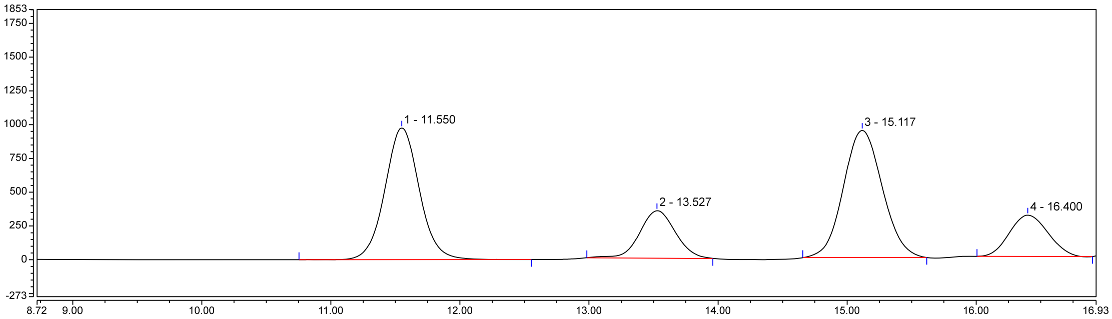


| Entry | Retention | Area | Height | %Area |
|---|---|---|---|---|
| 1 | 11.550 | 301.4628 | 973.10 | 35.53 |
| 2 | 13.527 | 112.3651 | 351.39 | 13.25 |
| 3 | 15.117 | 328.8371 | 941.16 | 38.76 |
| 4 | 16.400 | 105.6932 | 305.51 | 12.46 |

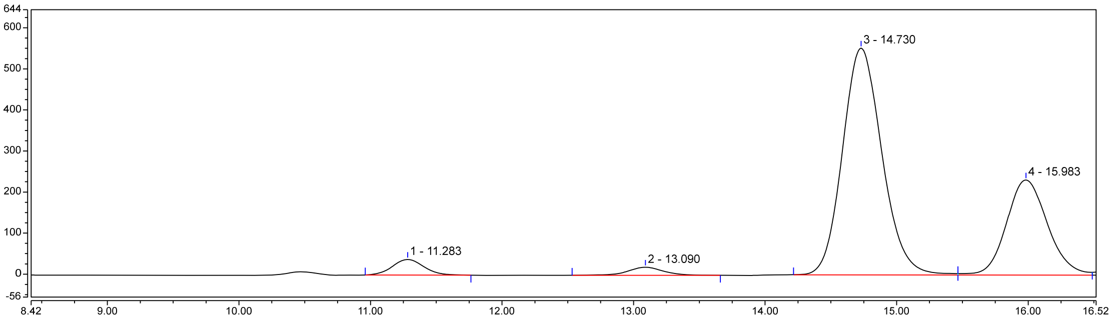


| Entry | Retention | Area | Height | %Area |
|---|---|---|---|---|
| 1 | 11.283 | 10.5345 | 38.15 | 3.60 |
| 2 | 13.090 | 6.1575 | 19.54 | 2.10 |
| 3 | 14.730 | 192.1202 | 552.08 | 65.67 |
| 4 | 15.983 | 83.7258 | 231.55 | 28.62 |

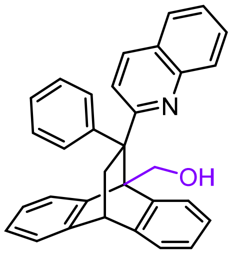


**3s:** Pale yellow oil; 31.6 mg, 72% yield, 88% ee; $[\alpha]_D^{22}$ –148.6 (*c* 2.0, $CHCl_3$); $^1$H NMR (300 MHz, Chloroform-*d*) δ 8.57 (d, *J* = 7.7 Hz, 1H), 8.01 (d, *J* = 8.3 Hz, 1H), 7.86 (d, *J* = 8.8 Hz, 1H), 7.69 (m, 2H), 7.56 – 7.45 (m, 1H), 7.41 (d, *J* = 7.3 Hz, 1H), 7.24 – 7.10 (m, 3H), 7.07 (d, *J* = 7.2 Hz, 1H), 7.03 – 6.84 (m, 5H), 6.68 (d, *J* = 7.7 Hz, 1H), 6.41 (d, *J* = 7.7 Hz, 2H), 4.86 (dd, *J* = 11.5, 4.9 Hz, 1H), 4.51 – 4.28 (m, 2H), 3.31 (dd, *J* = 13.2, 3.0 Hz, 1H), 2.64 (dd, *J* = 13.2, 2.7 Hz, 1H); $^{13}$C NMR (75 MHz, Chloroform-*d*) δ 168.8, 144.7, 144.0, 143.9, 143.3, 143.1, 142.3, 136.2, 129.9, 129.1, 127.6, 127.1, 127.0, 126.9, 126.3, 126.1, 125.8, 125.6, 125.5, 125.4, 125.3, 123.9, 122.7, 122.6, 62.5, 61.4, 54.1, 48.1, 44.7; HRMS (ESI) m/z 440.2006 $(M + H)^+$, calc. for $C_{32}H_{26}NO$ 440.2008.

The ee was determined by HPLC analysis: CHIRALPAK INC (4.6 mm i.d. x 250 mm);

hexane/2–propanol = 90/10; flow rate 1.0 mL/min; 25 °C; 210 nm; retention time: 12.8 min (minor) and 18.2 min (major).

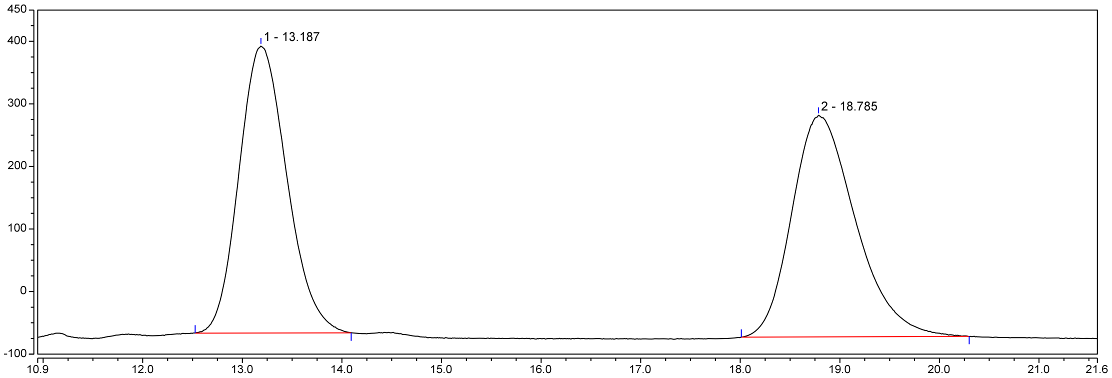


| Entry | Retention | Area | Height | %Area |
|---|---|---|---|---|
| 1 | 13.187 | 255.6495 | 459.07 | 49.32 |
| 2 | 18.785 | 262.6737 | 354.41 | 50.68 |

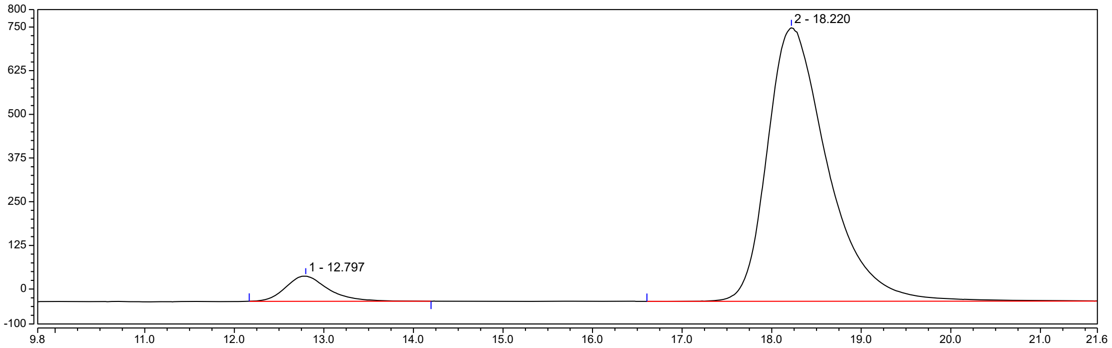


| Entry | Retention | Area | Height | %Area |
|---|---|---|---|---|
| 1 | 12.797 | 39.6929 | 72.02 | 6.08 |
| 2 | 18.220 | 613.4655 | 782.77 | 93.92 |

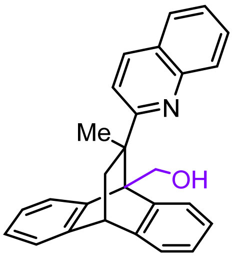


**3t:** White solid; Mp 123.6−125.7 °C; 30.5 mg, 81% yield, 82% ee; $[\alpha]^{22}_{D}$ +59.6 (*c* 2.0, $CHCl_3$); $^1$H NMR (300 MHz, Chloroform-*d*) δ 8.00 (d, *J* = 8.6 Hz, 1H), 7.73 – 7.63 (m, 3H), 7.63 – 7.56 (m, 1H), 7.51 – 7.46 (m, 1H), 7.46 – 7.34 (m, 3H), 7.26 – 7.15 (m, 4H), 7.02 (td, *J* = 7.6, 1.3 Hz, 1H), 6.13 (d, *J* = 8.8 Hz, 1H), 4.69 (d, *J* = 12.2 Hz, 1H), 4.52 (d, *J* = 12.2 Hz, 1H), 4.44 (t, *J* = 2.8 Hz, 1H), 3.29 (s, 1H), 2.70 (dd, *J* = 12.9, 2.9 Hz, 1H), 1.96 (dd, *J* = 13.0, 2.7 Hz, 1H), 1.42 (s, 3H); $^{13}$C NMR (75 MHz, Chloroform-*d*) δ 166.1, 146.1, 144.6, 144.1, 142.1, 141.5, 134.8, 129.2, 129.0, 127.0, 126.2, 126.1, 126.0, 126.0, 125.6, 125.45 125.0, 124.2, 123.2, 123.1, 120.7, 61.6, 54.5, 49.7, 46.8, 44.8, 26.5; HRMS (ESI) m/z 378.1848 (M + H)$^+$, calc. for $C_{27}H_{24}NO$ 378.1852.

The ee was determined by HPLC analysis: CHIRALPAK IF (4.6 mm i.d. x 250 mm); hexane/2–propanol = 90/10; flow rate 1.0 mL/min; 25 °C; 210 nm; retention time: 15.1 min (major) and

20.0 min (minor).

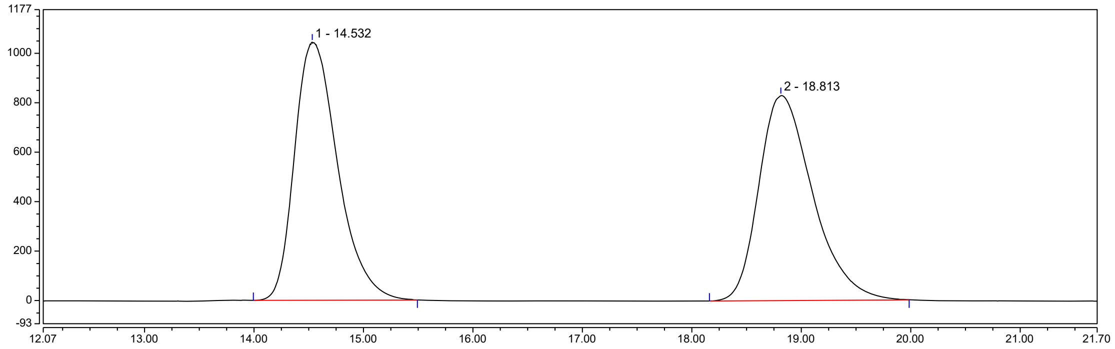

| Entry | Retention | Area | Height | %Area |
|---|---|---|---|---|
| 1 | 14.532 | 477.3687 | 1044.31 | 50.23 |
| 2 | 18.813 | 472.9942 | 829.69 | 49.77 |

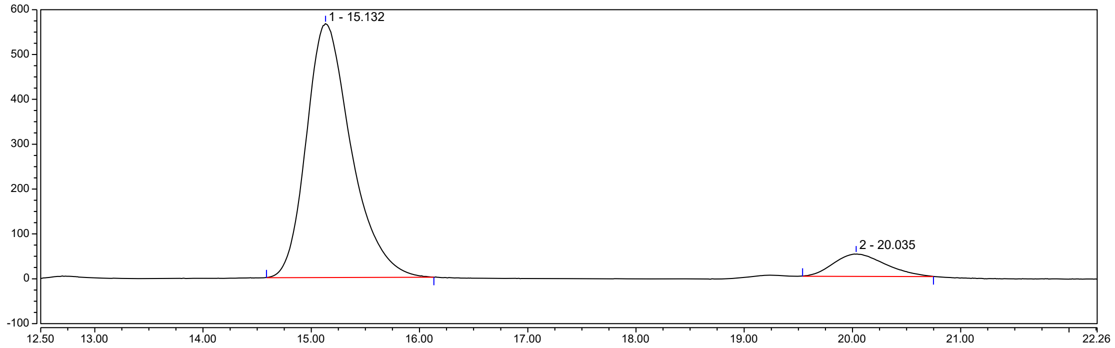

| Entry | Retention | Area | Height | %Area |
|---|---|---|---|---|
| 1 | 15.132 | 272.3866 | 566.13 | 90.76 |
| 2 | 20.035 | 27.7459 | 50.13 | 9.24 |

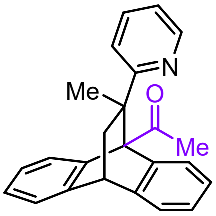


**3u:** Pale yellow oil; 28.4 mg, 84% yield, 84% ee; $[\alpha]_D^{22}$ +28.5 (*c* 2.0, $CHCl_3$); $^1$H NMR (300 MHz, Chloroform-*d*) δ 8.19 (d, *J* = 4.7 Hz, 1H), 7.46 – 7.31 (m, 3H), 7.26 – 7.14 (m, 4H), 7.09 – 6.95 (m, 2H), 6.89 – 6.86 (m, 2H), 4.36 (d, *J* = 2.9 Hz, 1H), 2.57 (dd, *J* = 12.4, 2.3 Hz, 1H), 2.48 (s, 3H), 1.76 (dd, *J* = 12.3, 2.3 Hz, 1H), 1.33 (s, 3H); $^{13}$C NMR (75 MHz, Chloroform-*d*) δ 207.7, 165.3, 146.5, 144.1, 143.4, 140.3, 139.7, 134.6, 126.7, 126.7, 126.3, 125.4, 125.1, 124.8, 123.5, 123.4, 123.0, 120.3, 69.3, 49.2, 48.8, 45.2, 33.0, 27.1; HRMS (ESI) m/z 340.1698 $(M + H)^+$, calc. for $C_{24}H_{22}NO$ 340.1696.

The ee was determined by HPLC analysis: Amylose-1 (4.6 mm i.d. x 250 mm); hexane/2–propanol = 90/10; flow rate 1.0 mL/min; 25 °C; 210 nm; retention time: 9.3 min (minor) and 17.9 min (major).

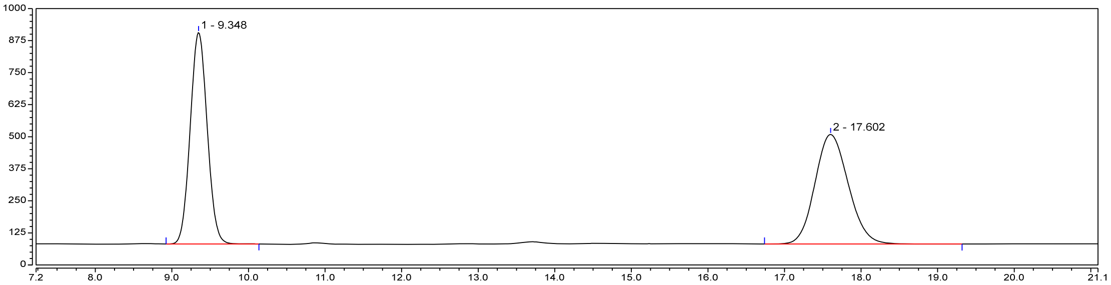


| Entry | Retention | Area | Height | %Area |
|---|---|---|---|---|
| 1 | 9.348 | 212.2547 | 824.47 | 49.63 |
| 2 | 17.602 | 215.4023 | 426.97 | 50.37 |

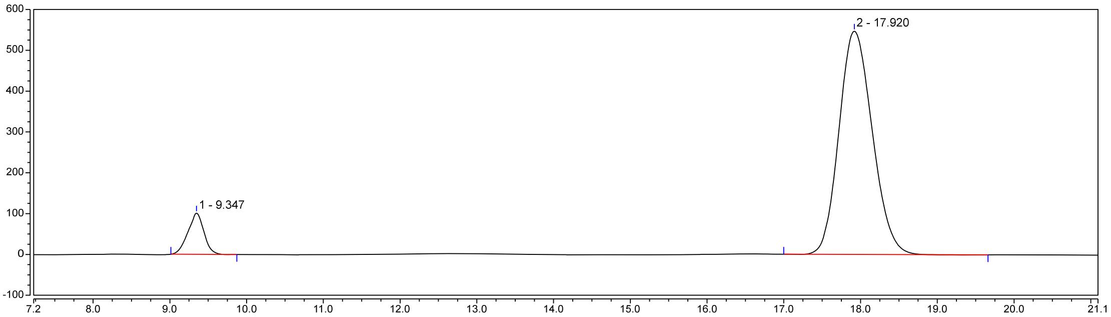


| Entry | Retention | Area | Height | %Area |
|---|---|---|---|---|
| 1 | 9.347 | 24.3324 | 101.13 | 8.13 |
| 2 | 17.920 | 275.0256 | 546.87 | 91.87 |

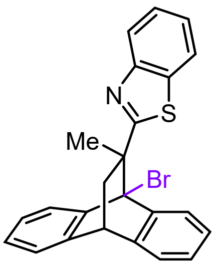


**3v:** Pale yellow solid; Mp 158.2−159.7 ºC; 24.2 mg, 56% yield, 90% ee; $[\alpha]_D^{22}$ +21.8 (*c* 2.0, $CHCl_3$); $^1$H NMR (300 MHz, Chloroform-*d*) δ 7.91 (d, *J* = 8.2 Hz, 1H), 7.89 – 7.83 (m, 1H), 7.70 – 7.63 (m, 2H), 7.41 (t, *J* = 6.2 Hz, 2H), 7.36 – 7.23 (m, 5H), 7.17 (t, *J* = 7.6 Hz, 1H), 4.43 (s, 1H), 2.83 (d, *J* = 12.8 Hz, 1H), 2.20 (d, *J* = 12.6 Hz, 1H), 1.53 (s, 3H); $^{13}$C NMR (75 MHz, Chloroform-*d*) δ 176.6, 151.6, 142.7, 141.3, 141.2, 140.1, 136.0, 128.3, 127.5, 127.3, 126.4, 126.2, 126.0, 125.4, 124.7, 123.0, 122.9, 122.8, 121.0, 53.1, 47.2, 44.3, 27.9; HRMS (ESI) m/z 434.0398 $(M + H)^+$, calc. for $C_{24}H_{19}BrNS$ 434.0396.

The ee was determined by HPLC analysis: CHIRALPAK IC*2 (4.6 mm i.d. x 250 mm); hexane/2–propanol = 95/5; flow rate 1.0 mL/min; 25 ºC; 210 nm; retention time: 10.1 min (minor) and 10.9 min (major).

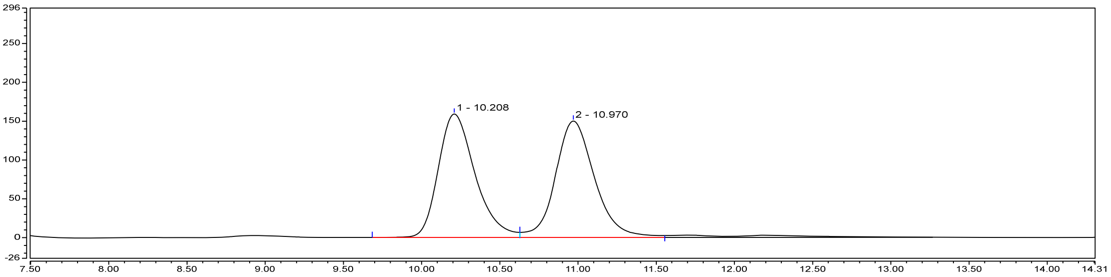

| Entry | Retention | Area | Height | %Area |
|---|---|---|---|---|
| 1 | 10.208 | 43.3544 | 158.90 | 49.94 |
| 2 | 10.970 | 43.4582 | 149.81 | 50.06 |

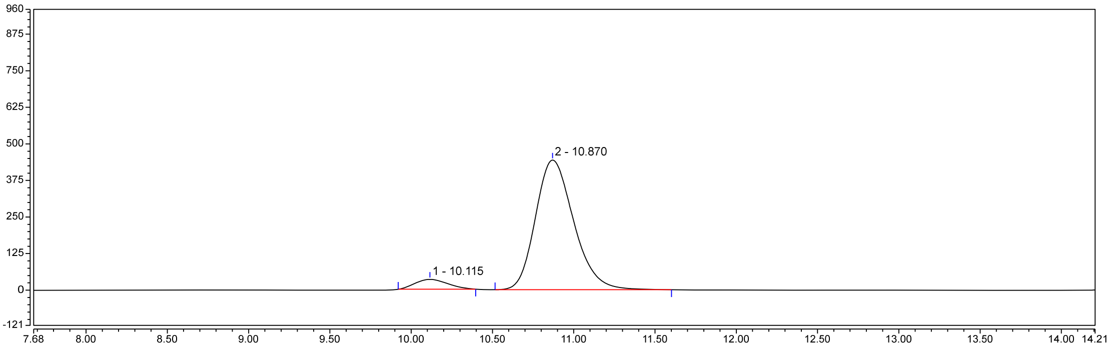


| Entry | Retention | Area | Height | %Area |
|---|---|---|---|---|
| 1 | 10.115 | 6.7239 | 31.00 | 5.27 |
| 2 | 10.870 | 120.8613 | 443.01 | 94.73 |

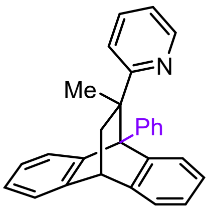


**3w:** Pale yellow oil; 26.4 mg, 71% yield, 89% ee; $[\alpha]_D^{22}$ +42.4 (*c* 2.0, $CHCl_3$); $^1$H NMR (300 MHz, Chloroform-*d*) δ 8.59 (s, 1H), 7.60 (s, 2H), 7.48 – 7.41(m, 2H), 7.36 – 7.20 (m, 5H), 7.13 – 7.03 (m, 3H), 6.97 (d, *J* = 7.1 Hz, 2H), 6.77 (d, *J* = 7.5 Hz, 1H), 5.57 (d, *J* = 8.1 Hz, 1H), 4.49 (s, 1H), 2.27 (d, *J* = 13.1 Hz, 1H), 2.12 (d, *J* = 12.7 Hz, 1H), 1.50 (s, 3H); $^{13}$C NMR (75 MHz, Chloroform-*d*) δ 167.04 146.8, 146.1, 145.0, 144.6, 142.7, 137.6, 135.1, 133.0, 128.0, 127.2, 126.9, 126.3, 126.2, 126.0, 125.1, 125.0, 124.1, 123.0, 122.6, 120.7, 62.0, 52.5, 49.8, 45.1, 26.9; HRMS (ESI) m/z 374.1905 $(M + H)^+$, calc. for $C_{28}H_{24}N$ 374.1903.

The ee was determined by HPLC analysis: CHIRALPAK IF (4.6 mm i.d. x 250 mm); hexane/2–propanol = 99/1; flow rate 1.0 mL/min; 25 °C; 210 nm; retention time: 4.7 min (major) and 6.2 min (minor).

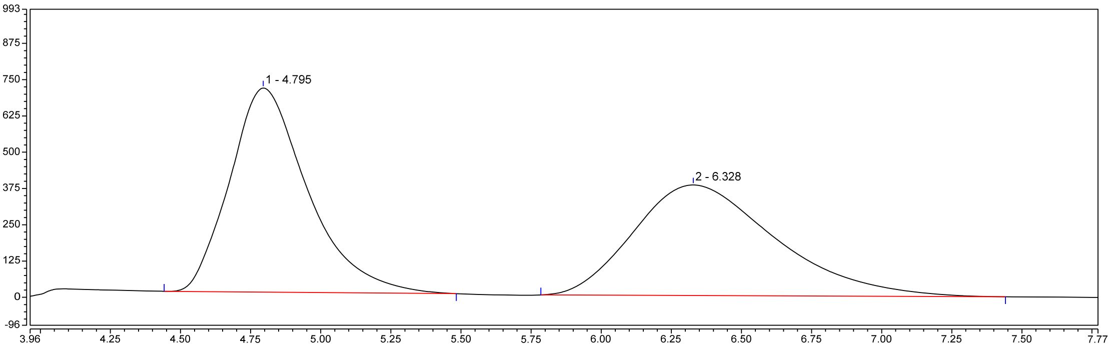


| Entry | Retention | Area | Height | %Area |
|---|---|---|---|---|
| 1 | 4.795 | 226.7666 | 702.89 | 50.32 |
| 2 | 6.328 | 223.8426 | 381.03 | 49.68 |

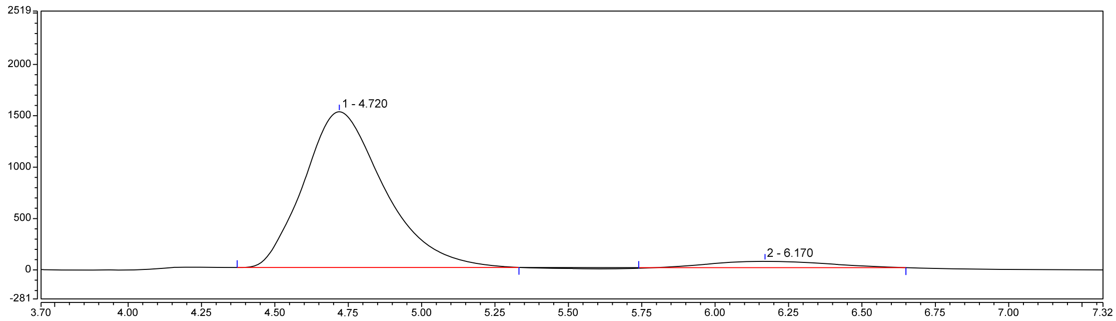

| Entry | Retention | Area | Height | %Area |
|---|---|---|---|---|
| 1 | 4.720 | 480.5641 | 1514.40 | 94.43 |
| 2 | 6.170 | 28.3302 | 62.22 | 5.57 |

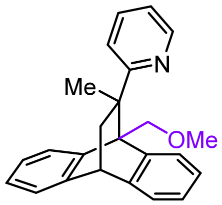


**3x:** Pale yellow oil; 25.9 mg, 76% yield, 94% ee; $[\alpha]_D^{22}$ +31.2 (*c* 2.0, $CHCl_3$); $^1H$ NMR (300 MHz, Chloroform-*d*) δ 8.47 (d, *J* = 4.8 Hz, 1H), 7.49 (d, *J* = 7.1 Hz, 1H), 7.37 (d, *J* = 7.3 Hz, 1H), 7.32 (d, *J* = 7.0 Hz, 1H), 7.25 – 7.13 (m, 4H), 7.01 (q, *J* = 7.3 Hz, 2H), 6.92 (d, *J* = 7.6 Hz, 1H), 5.69 (d, *J* = 8.2 Hz, 1H), 4.44 (d, *J* = 10.2 Hz, 1H), 4.39 (t, *J* = 2.8 Hz, 1H), 3.60 (d, *J* = 10.1 Hz, 1H), 3.47 (s, 3H), 2.44 (dd, *J* = 12.9, 2.8 Hz, 1H), 1.86 (dd, *J* = 12.9, 2.7 Hz, 1H), 1.21 (s, 3H); $^{13}C$ NMR (75 MHz, Chloroform-*d*) δ 164.8, 146.9, 144.4, 143.4, 141.8, 141.1, 134.7, 125.9, 125.7, 125.6, 125.5, 125.3, 125.1, 122.9, 122.7, 122.6, 120.8, 70.1, 59.0, 54.6, 48.7, 47.4, 44.9, 25.2; HRMS (ESI) m/z 342.1855 $(M + H)^+$, calc. for $C_{24}H_{24}NO$ 342.1853.

The ee was determined by HPLC analysis: CHIRALPAK Amylose-1 (4.6 mm i.d. x 250 mm); hexane/2–propanol = 95/5; flow rate 1.0 mL/min; 25 °C; 210 nm; retention time: 4.9 min (minor) and 6.3 min (major).

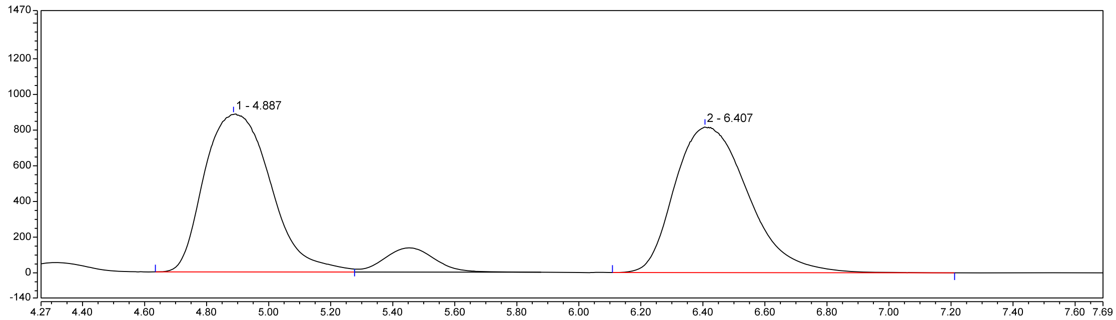

| Entry | Retention | Area | Height | %Area |
|---|---|---|---|---|
| 1 | 4.887 | 219.4438 | 884.76 | 49.52 |
| 2 | 6.407 | 223.6904 | 818.64 | 50.48 |

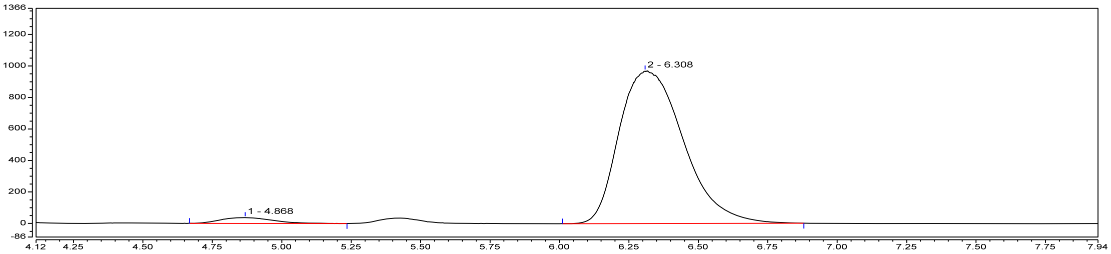

| Entry | Retention | Area | Height | %Area |
|---|---|---|---|---|
| 1 | 4.868 | 8.0425 | 37.46 | 2.99 |
| 2 | 6.308 | 260.7257 | 970.51 | 97.01 |

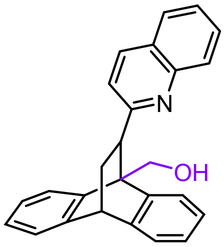

**3y:** Pale yellow oil; 33.4 mg, 92% yield, 87% ee; $[\alpha]_D^{22}$ –18.0 (*c* 2.0, $CHCl_3$); $^1H$ NMR (300 MHz, Chloroform-*d*) δ 8.04 (d, *J* = 8.4 Hz, 1H), 7.83 – 7.69 (m, 4H), 7.54 – 7.47 (m, 2H), 7.38 (d, *J* = 7.2 Hz, 1H), 7.28 – 7.16 (m, 2H), 7.05 (t, *J* = 7.4 Hz, 1H), 6.72 (d, *J* = 7.4 Hz, 1H), 5.96 (d, *J* = 8.4 Hz, 1H), 4.82 (s, 1H), 4.64 (d, *J* = 12.4 Hz, 1H), 4.54 – 4.43 (m, 1H), 3.99 – 3.89 (m, 1H), 3.83 (d, *J* = 13.0 Hz, 1H), 2.61 – 2.45 (m, 1H), 2.21 – 2.07 (m, 1H); $^{13}C$ NMR (75 MHz, Chloroform-*d*) δ 162.9, 146.6, 144.7, 144.3, 143.6, 140.1, 136.4, 129.7, 128.6, 127.4, 127.0, 126.4, 126.2, 125.8, 125.7, 125.6, 123.3, 123.3, 123.1, 122.6, 119.1, 60.7, 51.4, 47.3, 44.5, 35.0; HRMS (ESI) m/z 364.1692 $(M + H)^+$, calc. for $C_{26}H_{22}NO$ 364.1695.

The ee was determined by HPLC analysis: CHIRALPAK IF (4.6 mm i.d. x 250 mm); hexane/2–propanol = 90/10; flow rate 1.0 mL/min; 25 °C; 210 nm; retention time: 22.7 min (minor) and 25.4 min (major).

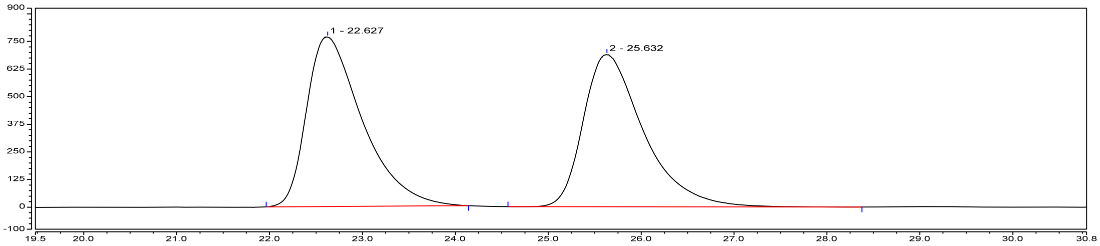

| Entry | Retention | Area | Height | %Area |
|---|---|---|---|---|
| 1 | 22.627 | 534.1290 | 768.22 | 50.73 |
| 2 | 25.632 | 518.6695 | 690.02 | 49.27 |

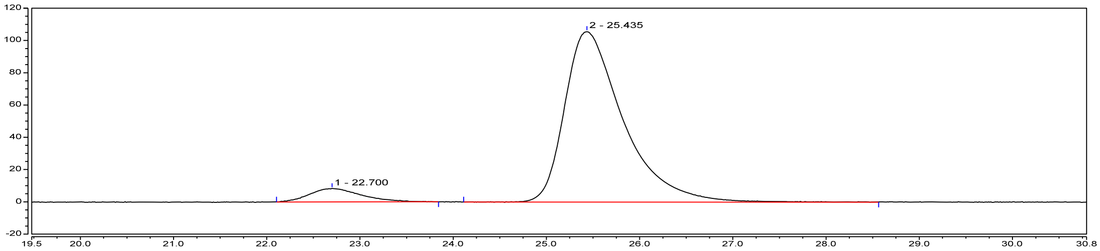

| Entry | Retention | Area | Height | %Area |
|---|---|---|---|---|
| 1 | 22.700 | 5.3362 | 8.36 | 6.50 |
| 2 | 25.435 | 76.7003 | 105.75 | 93.50 |

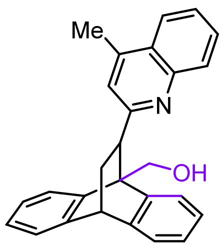

**3za:** Colorless oil; 32.0 mg, 87% yield, 85% ee; $[\alpha]_D^{22}$ –54.2 (*c* 2.0, $CHCl_3$); $^1$H NMR (300 MHz, Chloroform-*d*) δ 8.01 (d, *J* = 8.4 Hz, 1H), 7.89 (d, *J* = 8.4 Hz, 1H), 7.75 (d, *J* = 7.4 Hz, 1H), 7.69 (m, 1H), 7.56 – 7.46 (m, 2H), 7.37 (d, *J* = 7.1 Hz, 1H), 7.26 – 7.13 (m, 3H), 7.04 (td, *J* = 7.6, 1.3 Hz, 1H), 6.70 (d, *J* = 7.5 Hz, 1H), 5.69 (s, 1H), 4.87 (s, 1H), 4.62 (d, *J* = 12.5 Hz, 1H), 4.55 – 4.47 (m, 1H), 3.87 (dd, *J* = 10.6, 5.0 Hz, 1H), 3.80 (d, *J* = 12.4 Hz, 1H), 2.51 (m, 1H), 2.38 (s, 3H), 2.14 (m, 1H); $^{13}$C NMR (75 MHz, Chloroform-*d*) δ 162.5, 146.5, 144.6, 144.3, 143.6, 140.3, 129.3, 129.2, 127.0, 126.1, 125.8, 125.6, 125.4, 123.6, 123.3, 123.3, 123.0, 122.6, 119.9, 60.6, 51.4, 47.3, 44.5, 34.9, 18.8; HRMS (ESI) m/z 378.1850 (M + H)$^+$, calc. for $C_{27}H_{24}NO$ 378.1852.

The ee was determined by HPLC analysis: CHIRALPAK IF (4.6 mm i.d. x 250 mm); hexane/2–propanol = 90/10; flow rate 1.0 mL/min; 25 °C; 254 nm; retention time: 18.5 min (minor) and 20.1 min (major).

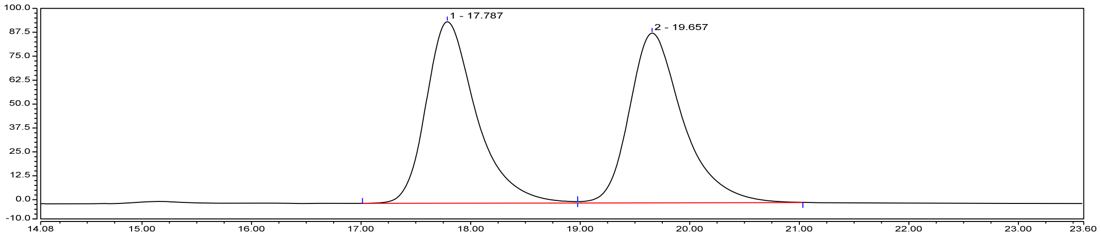

| Entry | Retention | Area | Height | %Area |
|---|---|---|---|---|
| 1 | 17.787 | 50.4965 | 94.81 | 50.13 |
| 2 | 19.657 | 50.2374 | 88.62 | 49.87 |

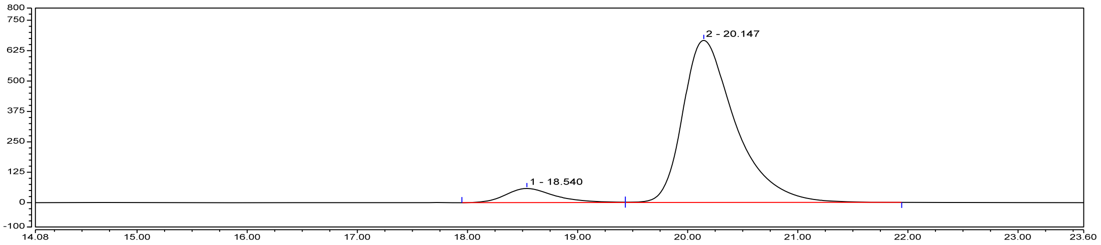

| Entry | Retention | Area | Height | %Area |
|---|---|---|---|---|
| 1 | 18.540 | 30.0591 | 58.13 | 7.51 |
| 2 | 20.147 | 370.0082 | 667.04 | 92.49 |

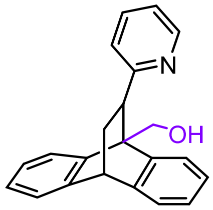

**3zb:** Yellow oil; 23.7 mg, 76% yield, 66% ee; $[\alpha]_D^{22}$ –25.8 (*c* 2.0, $CHCl_3$); $^1$H NMR (300 MHz, Chloroform-*d*) δ 8.47 (d, *J* = 5.0 Hz, 1H), 7.72 (d, *J* = 7.4 Hz, 1H), 7.42 (d, *J* = 7.4 Hz, 1H), 7.40 – 7.30 (m, 2H), 7.27 – 6.99 (m, 5H), 6.72 (d, *J* = 7.6 Hz, 1H), 5.88 (d, *J* = 8.0 Hz, 1H), 4.61 (d, *J* = 12.5 Hz, 1H), 4.45 (d, *J* = 2.9 Hz, 1H), 3.81 (d, *J* = 12.5 Hz, 1H), 3.78 – 3.67 (m, 1H), 2.47 (td, *J* =

11.7, 10.6, 2.7 Hz, 1H), 2.00 (m, 1H); $^{13}$C NMR (75 MHz, Chloroform-*d*) δ 162.4, 148.1, 144.7, 144.3, 143.5, 140.0, 136.5, 126.1, 125.8, 125.6, 125.4, 123.3, 123.1, 123.1, 122.6, 121.9, 121.3, 60.6, 51.6, 46.4, 44.5, 35.4; HRMS (ESI) m/z 314.1534 (M + H)$^+$, calc. for $C_{22}H_{20}NO$ 314.1539. The ee was determined by HPLC analysis: CHIRALPAK IF (4.6 mm i.d. x 250 mm); hexane/2–propanol = 90/10; flow rate 1.0 mL/min; 25 °C; 254 nm; retention time: 13.9 min (major) and 17.6 min (minor).

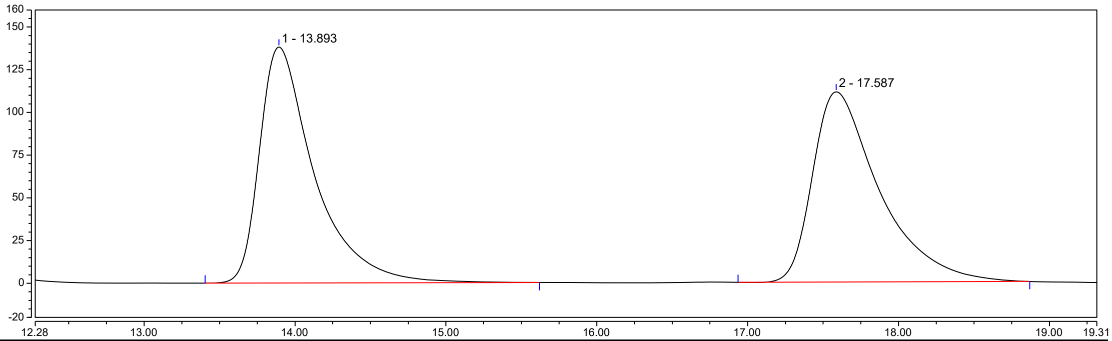


| Entry | Retention | Area | Height | %Area |
|---|---|---|---|---|
| 1 | 13.893 | 58.0864 | 138.07 | 50.48 |
| 2 | 17.587 | 56.9843 | 111.30 | 49.52 |

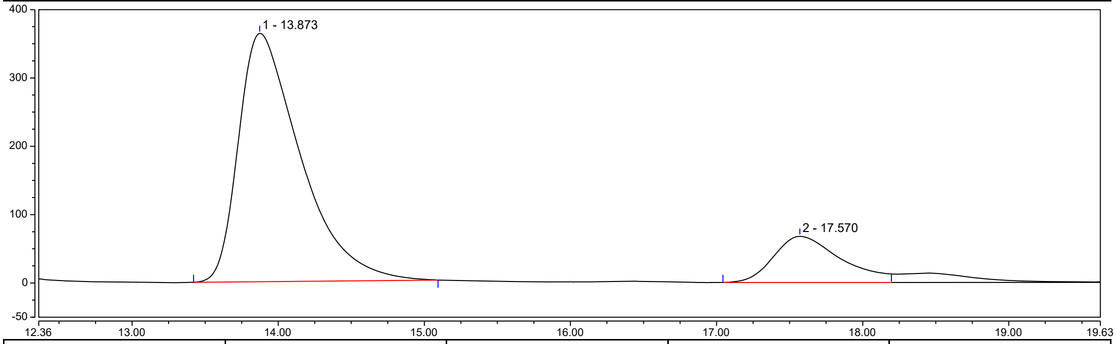


| Entry | Retention | Area | Height | %Area |
|---|---|---|---|---|
| 1 | 13.873 | 178.5991 | 363.26 | 83.20 |
| 2 | 17.570 | 36.0592 | 67.60 | 16.80 |

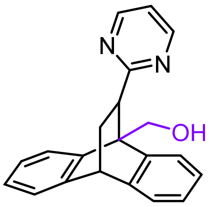


**3zc:** Orange solid; Mp 114.7−116.3 °C; 13.5 mg, 43% yield, 90% ee; $[\alpha]_D^{22}$ −14.5 (*c* 2.0, $CHCl_3$); $^1$H NMR (300 MHz, Chloroform-*d*) δ 8.53 (d, *J* = 4.9 Hz, 2H), 7.75 (d, *J* = 7.6 Hz, 1H), 7.42 (d, *J* = 7.2 Hz, 1H), 7.35 (d, *J* = 7.1 Hz, 1H), 7.22 (t, *J* = 7.3 Hz, 1H), 7.15 (t, *J* = 7.2 Hz, 2H), 7.06 (t, *J* = 4.9 Hz, 1H), 6.98 (t, *J* = 7.6 Hz, 1H), 6.68 (d, *J* = 7.6 Hz, 1H), 4.72 (d, *J* = 12.7 Hz, 1H), 4.46 (d, *J* = 2.8 Hz, 1H), 4.05 (d, *J* = 12.6 Hz, 1H), 3.81 (dd, *J* = 10.2, 5.1 Hz, 1H), 2.75 (m, 1H), 2.27 (ddd, *J* = 12.5, 10.2, 2.9 Hz, 1H); $^{13}$C NMR (75 MHz, Chloroform-*d*) δ 170.3, 156.4, 144.9, 144.9, 143.7, 139.5, 125.9, 125.7, 125.6, 125.1, 123.2, 123.1, 122.9, 121.6, 118.7, 60.6, 51.6, 46.8, 44.4, 32.4; HRMS (ESI) m/z 315.1489 (M + H)$^+$, calc. for $C_{21}H_{19}N_2O$ 315.1491.

The ee was determined by HPLC analysis: CHIRALPAK IF (4.6 mm i.d. x 250 mm); hexane/2–propanol = 80/20; flow rate 1.0 mL/min; 25 °C; 210 nm; retention time: 9.9 min (major) and 13.2 min (minor).

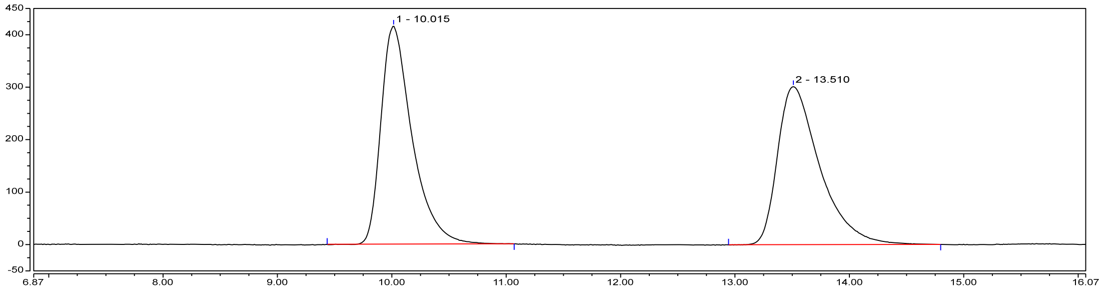

| Entry | Retention | Area | Height | %Area |
|---|---|---|---|---|
| 1 | 10.015 | 130.9722 | 415.79 | 50.37 |
| 2 | 13.510 | 129.0438 | 301.77 | 49.63 |

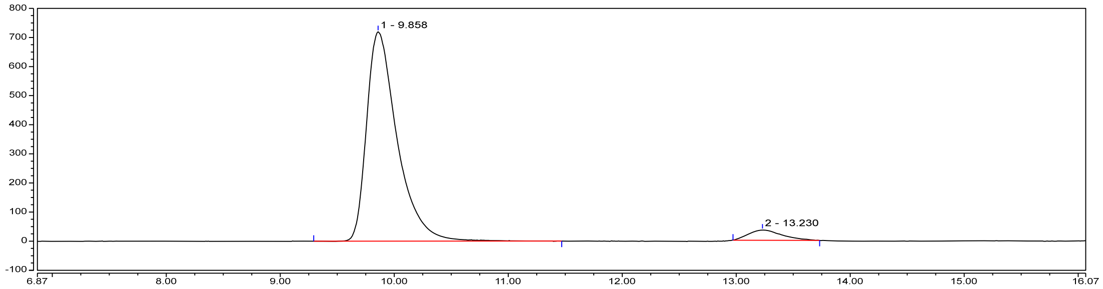

| Entry | Retention | Area | Height | %Area |
|---|---|---|---|---|
| 1 | 9.858 | 223.4267 | 719.98 | 94.96 |
| 2 | 13.230 | 11.8699 | 35.22 | 5.04 |

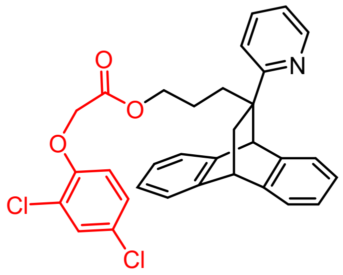


**3zd:** Colorless oil; 33.6 mg, 62% yield, 85% ee; $[\alpha]_D^{22}$ +17.5 (*c* 2.0, $CHCl_3$); $^1$H NMR (300 MHz, Chloroform-*d*) δ 8.47 (d, *J* = 4.7 Hz, 1H), 7.45 – 7.34 (m, 3H), 7.31 (t, *J* = 4.1 Hz, 1H), 7.20 (d, *J* = 7.3 Hz, 1H), 7.17 – 7.07 (m, 3H), 7.01 (d, *J* = 8.0 Hz, 1H), 6.93 (t, *J* = 7.8 Hz, 3H), 6.81 (t, *J* = 7.4 Hz, 1H), 6.66 (d, *J* = 8.8 Hz, 1H), 4.71 (s, 1H), 4.52 (s, 2H), 4.36 (s, 1H), 3.96 – 3.81 (m, 2H), 2.78 (d, *J* = 12.7 Hz, 1H), 1.78 (s, 1H), 1.36 – 1.31 (m, 1H), 1.19 – 1.09 (m, 2H), 0.89 – 0.84 (m, 1H); $^{13}$C NMR (75 MHz, Chloroform-*d*) δ 167.8, 164.9, 152.3, 148.3, 143.9, 143.4, 141.8, 141.5, 135.6, 130.2, 127.5, 126.9, 126.0, 125.4, 125.4, 125.0, 124.1, 123.1, 122.9, 121.7, 120.5, 114.6, 66.1, 65.5, 53.5, 50.5, 44.7, 39.5, 39.2, 23.9; HRMS (ESI) m/z 544.1443 $(M + H)^+$, calc. for $C_{32}H_{28}Cl_2NO_3$ 544.1441.

The ee was determined by HPLC analysis: CHIRALPAK Amylose-1 (4.6 mm i.d. x 250 mm); hexane/2–propanol = 70/30; flow rate 1.0 mL/min; 25 °C; 210 nm; retention time: 10.0 min

(major) and 18.1 min (minor).

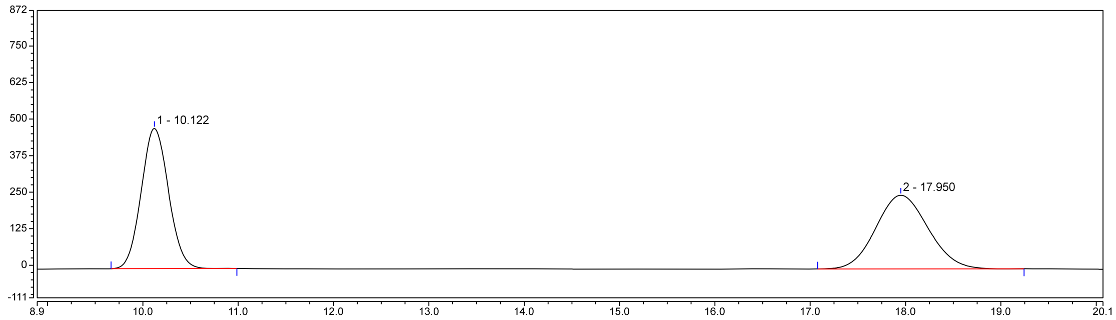


| Entry | Retention | Area | Height | %Area |
|---|---|---|---|---|
| 1 | 10.122 | 159.3409 | 479.54 | 49.84 |
| 2 | 17.950 | 160.3883 | 251.90 | 50.16 |

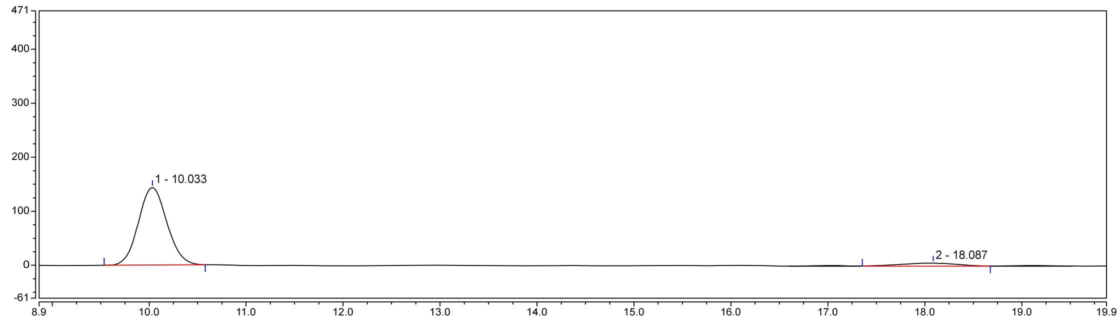


| Entry | Retention | Area | Height | %Area |
|---|---|---|---|---|
| 1 | 10.033 | 48.7095 | 143.36 | 92.74 |
| 2 | 18.087 | 3.8154 | 5.71 | 7.26 |

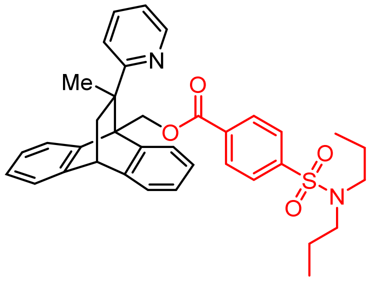


**3ze:** White solid; Mp 98.2−100.1 °C; 36.8 mg, 62% yield, 90% ee; $[\alpha]_D^{22}$ +18.2 (*c* 2.0, $CHCl_3$); $^1$H NMR (300 MHz, Chloroform-*d*) δ 8.45 (d, *J* = 6.0 Hz, 1H), 7.95 (d, *J* = 8.2 Hz, 2H), 7.73 (d, *J* = 8.2 Hz, 2H), 7.46 (d, *J* = 7.3 Hz, 1H), 7.43 – 7.35 (m, 2H), 7.24 – 7.15 (m, 4H), 6.98 (q, *J* = 7.7, 7.2 Hz, 2H), 6.83 (d, *J* = 7.7 Hz, 1H), 5.79 (d, *J* = 8.2 Hz, 1H), 5.48 (d, *J* = 12.4 Hz, 1H), 4.75 (d, *J* = 12.4 Hz, 1H), 4.48 (t, *J* = 2.8 Hz, 1H), 3.17 – 2.91 (m, 4H), 2.56 (dd, *J* = 12.9, 2.8 Hz, 1H), 1.91 (dd, *J* = 12.9, 2.6 Hz, 1H), 1.54 – 1.44 (m, 4H), 0.83 (t, *J* = 7.4 Hz, 6H); $^{13}$C NMR (75 MHz, Chloroform-*d*) δ 165.3, 164.0, 147.4, 144.2, 143.5, 141.0, 140.5, 134.9, 133.1, 130.3, 126.9, 126.3, 126.2, 125.7, 125.4, 124.6, 124.1, 123.4, 123.2, 122.4, 121.0, 63.4, 53.9, 50.0, 49.1, 46.9, 45.0, 25.2, 21.9, 11.1; HRMS (ESI) m/z 595.2627 $(M + H)^+$, calc. for $C_{36}H_{39}N_2O_4S$ 595.2625.

The ee was determined by HPLC analysis: CHIRALPAK Amylose-1 (4.6 mm i.d. x 250 mm);

hexane/2–propanol = 90/10; flow rate 1.0 mL/min; 25 °C; 210 nm; retention time: 8.3 min (minor) and 9.2 min (major).

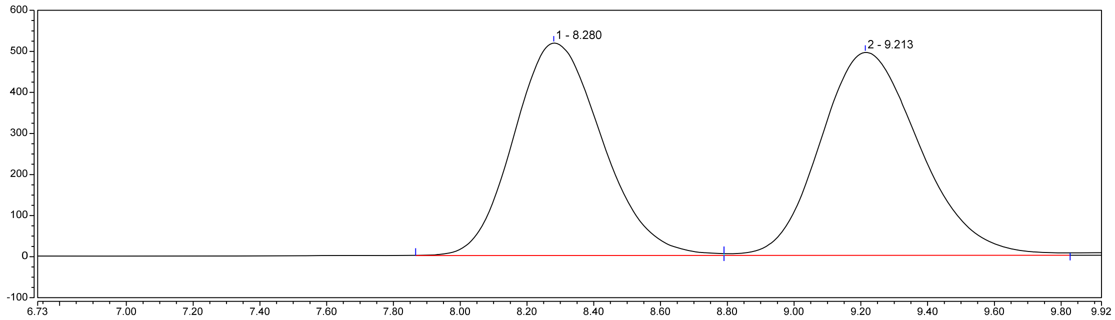

| Entry | Retention | Area | Height | %Area |
|---|---|---|---|---|
| 1 | 8.280 | 158.7650 | 516.60 | 48.18 |
| 2 | 9.213 | 170.7844 | 493.71 | 51.82 |

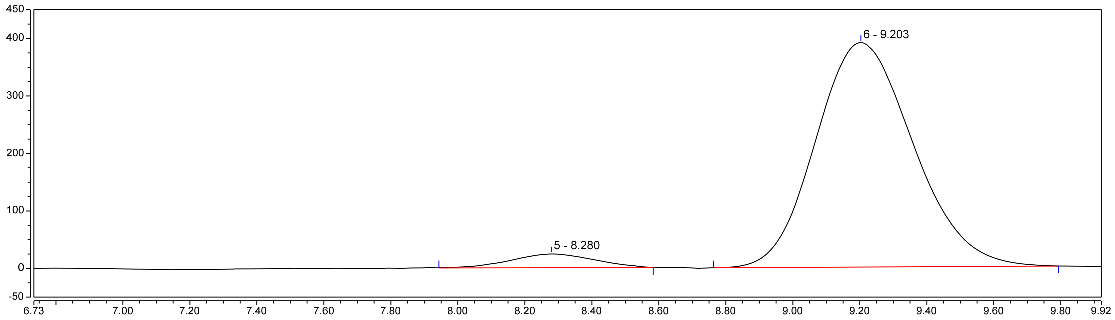

| Entry | Retention | Area | Height | %Area |
|---|---|---|---|---|
| 1 | 8.280 | 6.8957 | 24.01 | 4.94 |
| 2 | 9.203 | 132.5665 | 391.28 | 95.06 |

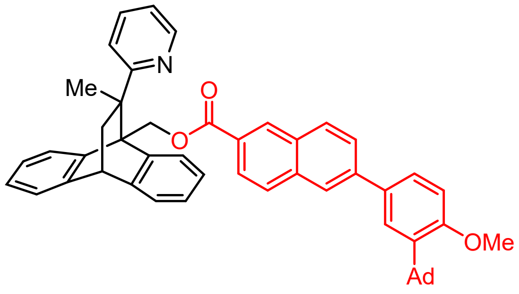


**3zf:** Pale yellow solid; Mp 125.8−127.3 °C; 60.0 mg, 83% yield, 90% ee; $[\alpha]_D^{22}$ +39.5 (*c* 2.0, $CHCl_3$); $^1$H NMR (300 MHz, Chloroform-*d*) δ 8.50 (s, 1H), 8.43 (s, 1H), 7.97 (s, 1H), 7.93 – 7.72 (m, 4H), 7.60 (s, 1H), 7.53 (t, *J* = 8.1 Hz, 3H), 7.45 (d, *J* = 6.4 Hz, 1H), 7.29 – 7.16 (m, 4H), 7.06 – 6.97 (m, 4H), 5.86 (d, *J* = 8.2 Hz, 1H), 5.55 (d, *J* = 12.5 Hz, 1H), 4.80 (d, *J* = 13.0 Hz, 1H), 4.52 (s, 1H), 3.90 (s, 3H), 2.62 (d, *J* = 12.9 Hz, 1H), 2.20 (s, 6H), 2.12 (s, 3H), 1.96 (d, *J* = 13.0 Hz, 1H), 1.82 (s, 6H), 1.36 (s, 3H); $^{13}$C NMR (75 MHz, Chloroform-*d*) δ 166.9, 164.13, 158.8, 147.4, 144.3, 143.4, 141.4, 141.3, 140.8, 138.9, 135.9, 134.8, 132.4, 131.1, 130.9, 129.7, 128.1, 126.7, 126.2, 126.2, 126.1, 125.9, 125.7, 125.7, 125.5, 125.0, 124.6, 124.4, 123.3, 123.1, 122.5, 121.0, 112.0, 62.9, 55.1, 53.9, 49.1, 47.1, 45.0, 40.5, 37.1, 37.1, 29.0, 25.3; HRMS (ESI) m/z 722.3632 $(M + H)^+$, calc. for $C_{51}H_{48}NO_3$ 722.3629.

The ee was determined by HPLC analysis: CHIRALPAK i-Amylose-3 (4.6 mm i.d. x 250 mm); hexane/2–propanol = 95/5; flow rate 1.0 mL/min; 25 °C; 210 nm; retention time: 41.1 min (major) and 50.3 min (minor).

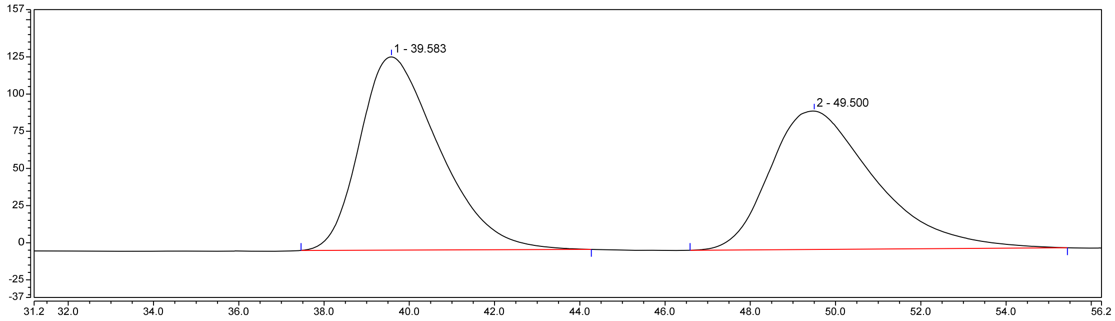


| Entry | Retention | Area | Height | %Area |
|---|---|---|---|---|
| 1 | 39.583 | 281.0955 | 129.89 | 51.27 |
| 2 | 49.500 | 267.1429 | 93.11 | 48.73 |

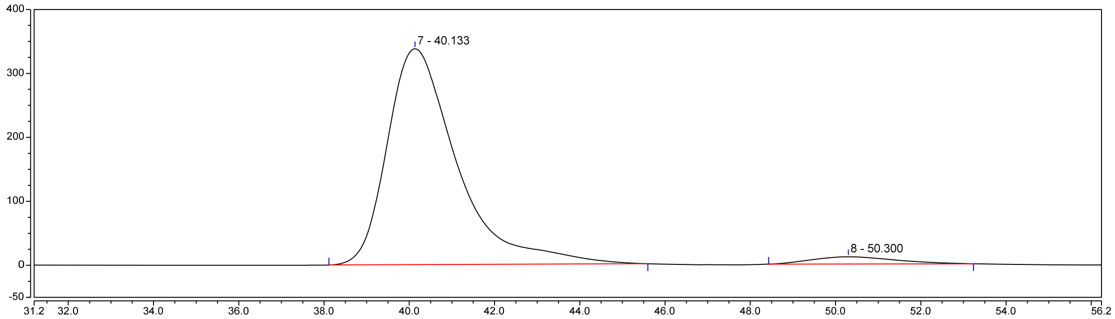


| Entry | Retention | Area | Height | %Area |
|---|---|---|---|---|
| 1 | 40.133 | 705.7215 | 365.86 | 95.04 |
| 2 | 50.300 | 36.8086 | 13.99 | 4.96 |

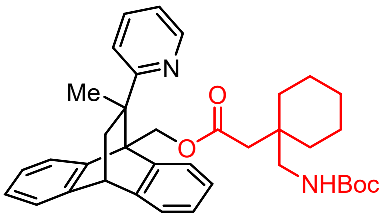


**3zg:** Pale yellow solid; Mp 78.5−80.3 °C; 40.6 mg, 70% yield, 88% ee; $[\alpha]_D^{22}$ +24.7 (*c* 2.0, $CHCl_3$); $^1$H NMR (300 MHz, Chloroform-*d*) δ 8.45 (d, *J* = 4.6 Hz, 1H), 7.42 (d, *J* = 7.4 Hz, 1H), 7.36 (d, *J* = 6.1 Hz, 1H), 7.31 (d, *J* = 6.2 Hz, 1H), 7.19 (q, *J* = 9.9, 9.2 Hz, 4H), 6.97 (t, *J* = 7.6 Hz, 2H), 6.72 (d, *J* = 7.7 Hz, 1H), 5.74 (d, *J* = 8.2 Hz, 1H), 5.18 (d, *J* = 12.3 Hz, 1H), 5.02 (t, *J* = 7.0 Hz, 1H), 4.48 (d, *J* = 12.5 Hz, 1H), 4.43 (s, 1H), 3.11 (d, *J* = 6.8 Hz, 2H), 2.50 (d, *J* = 13.0 Hz, 1H), 2.23 (s, 2H), 1.84 (d, *J* = 12.9 Hz, 1H), 1.40 (s, 10H), 1.32 (s, 8H), 1.23 (s, 3H); $^{13}$C NMR (75 MHz, Chloroform-*d*) δ 172.7, 164.0, 156.4, 147.3, 144.1, 143.3, 140.9, 140.3, 134.8, 126.3, 126.1, 125.5, 125.3, 124.6, 124.2, 123.3, 123.1, 122.4, 121.0, 78.7, 62.0, 53.6, 49.0, 47.1, 46.9, 44.9, 40.6, 38.0, 34.0, 28.4, 25.8, 25.2, 21.3; HRMS (ESI) m/z 581.3376 (M + H)$^+$, calc. for $C_{37}H_{45}N_2O_{43}$ 581.3374.

The ee was determined by HPLC analysis: CHIRALPAK i-Amylose-1*2 (4.6 mm i.d. x 250 mm); hexane/2–propanol = 90/10; flow rate 1.0 mL/min; 25 °C; 210 nm; retention time: 16.0 min (minor) and 24.7 min (major).

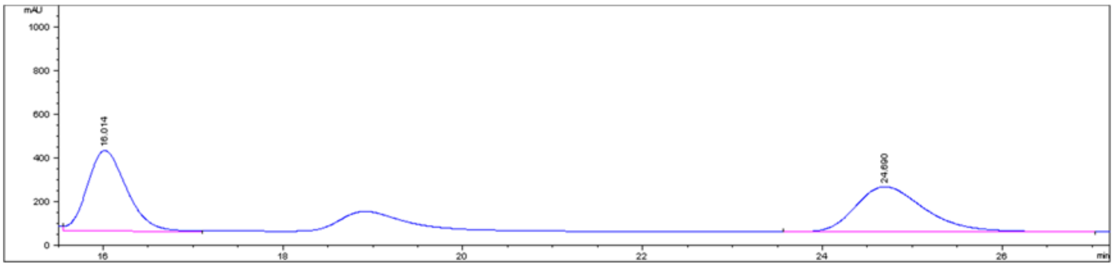

| Entry | Retention | Area | Height | %Area |
|---|---|---|---|---|
| 1 | 16.014 | 11348.3 | 370.8 | 50.736 |
| 2 | 24.69 | 11019.2 | 206.5 | 49.264 |

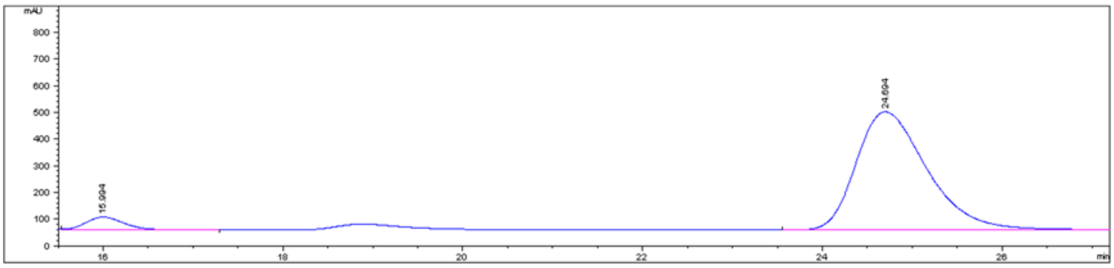

| Entry | Retention | Area | Height | %Area |
|---|---|---|---|---|
| 1 | 15.994 | 1576.7 | 49.8 | 6.097 |
| 2 | 24.694 | 24281.6 | 443.2 | 93.903 |

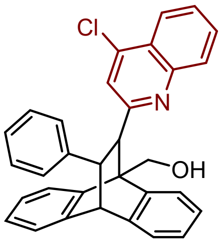


**5a:** White solid; Mp 129.7−131.2 °C; 43.5 mg, 92% yield, 92% ee, > 20:1 dr; $[\alpha]_D^{22}$ +133.1 (*c* 2.0, $CHCl_3$); $^1$H NMR (300 MHz, Chloroform-*d*) δ 8.13 (d, *J* = 8.4 Hz, 1H), 7.95 (d, *J* = 8.4 Hz, 1H), 7.85 (d, *J* = 7.6 Hz, 1H), 7.72 (m, 1H), 7.64 – 7.51 (m, 2H), 7.33 (m, 2H), 7.14 (m, 6H), 6.82 (d, *J* = 7.5 Hz, 1H), 6.76 – 6.62 (m, 2H), 6.19 (s, 1H), 4.68 (t, *J* = 11.6 Hz, 1H), 4.52 (s, 1H), 4.41 (d, *J* = 2.3 Hz, 1H), 4.01 (d, *J* = 6.0 Hz, 1H), 3.81 (d, *J* = 9.2 Hz, 1H), 3.56 (dd, *J* = 6.1, 2.3 Hz, 1H); $^{13}$C NMR (75 MHz, Chloroform-*d*) δ 162.2, 147.6, 144.8, 143.4, 143.1, 142.8, 141.0, 139.7, 130.7, 129.1, 128.1, 128.0, 127.5, 126.7, 126.6, 126.3, 126.2, 125.9, 125.7, 125.3, 123.9, 123.5, 123.0, 122.7, 119.4, 60.8, 56.3, 53.0, 52.1, 52.1; HRMS (ESI) m/z 474.1614 $(M + H)^+$, calc. for $C_{32}H_{25}ClNO$ 474.1619.

The ee was determined by HPLC analysis: Amylose-1 (4.6 mm i.d. x 250 mm); hexane/2–propanol = 90/10; flow rate 1.0 mL/min; 25 °C; 210 nm; retention time: 8.7 min (minor) and 9.7 min (major).

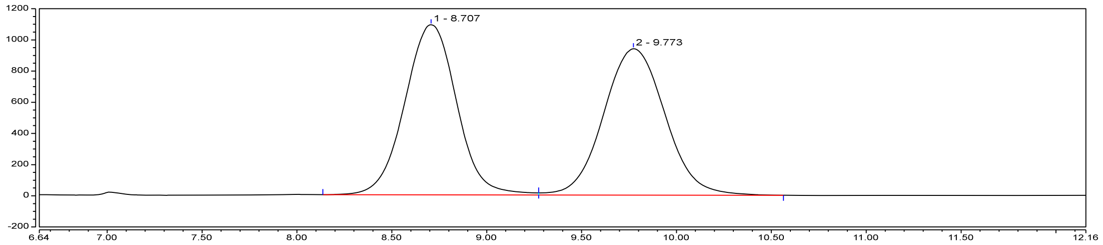

| Entry | Retention | Area | Height | %Area |
|---|---|---|---|---|
| 1 | 8.707 | 346.1201 | 1090.43 | 49.52 |
| 2 | 9.773 | 352.8365 | 938.80 | 50.48 |

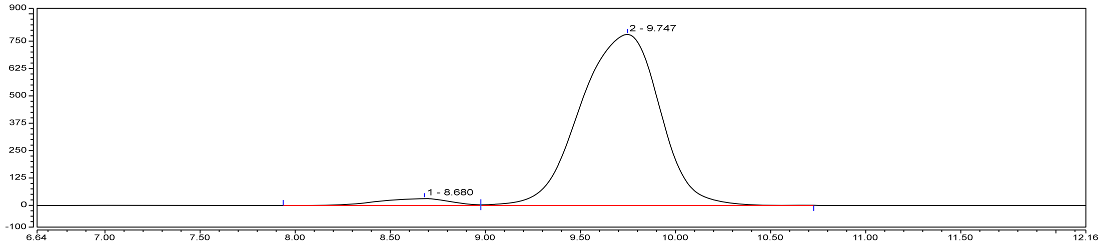

| Entry | Retention | Area | Height | %Area |
|---|---|---|---|---|
| 1 | 8.680 | 13.9386 | 31.65 | 3.56 |
| 2 | 9.747 | 378.0094 | 781.69 | 96.44 |

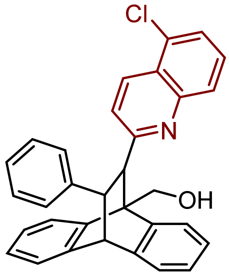


**5b:** White solid; Mp 93.7−95.1 °C; 39.7 mg, 84% yield, 96% ee, > 20:1 dr; $[\alpha]_D^{22}$ +104.2 (*c* 2.0, $CHCl_3$); $^1$H NMR (300 MHz, Chloroform-*d*) δ 8.30 (d, *J* = 8.9 Hz, 1H), 7.86 (d, *J* = 7.0 Hz, 2H), 7.61 – 7.51 (m, 3H), 7.32 (dd, *J* = 15.6, 7.7 Hz, 2H), 7.20 – 7.03 (m, 6H), 6.81 (d, *J* = 7.6 Hz, 1H), 6.71 – 6.68 (m, 2H), 6.31 (d, *J* = 8.9 Hz, 1H), 4.68 (d, *J* = 12.7 Hz, 1H), 4.56 (s, 1H), 4.42 (s, 1H), 4.04 (d, *J* = 6.1 Hz, 1H), 3.79 (d, *J* = 11.9 Hz, 1H), 3.64 (d, *J* = 4.1 Hz, 1H); $^{13}$C NMR (75 MHz, Chloroform-*d*) δ 162.9, 147.5, 145.1, 143.6, 142.9, 141.1, 139.9, 133.5, 131.1, 129.5, 128.1, 127.9, 126.7, 126.5, 126.3, 126.2, 125.9, 125.7, 125.3, 123.4, 123.0, 122.7, 119.9, 60.8, 56.4, 52.9, 52.1, 52.1; HRMS (ESI) m/z 474.1614 $(M + H)^+$, calc. for $C_{32}H_{25}ClNO$ 474.1619.

The ee was determined by HPLC analysis: Amylose-1 (4.6 mm i.d. x 250 mm); hexane/2–propanol = 90/10; flow rate 1.0 mL/min; 25 °C; 210 nm; retention time: 14.1 min (minor) and 15.5 min (major).

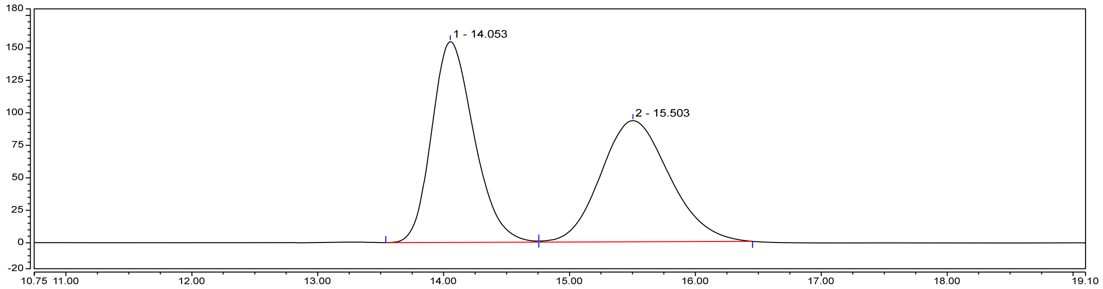

| Entry | Retention | Area | Height | %Area |
|---|---|---|---|---|
| 1 | 14.053 | 60.2741 | 154.44 | 50.49 |
| 2 | 15.503 | 59.1136 | 93.22 | 49.51 |

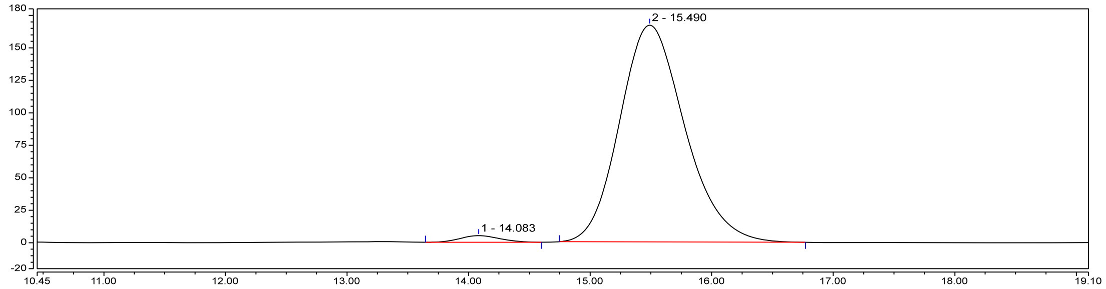


| Entry | Retention | Area | Height | %Area |
|---|---|---|---|---|
| 1 | 14.083 | 1.9347 | 5.13 | 1.83 |
| 2 | 15.490 | 103.8680 | 166.83 | 98.17 |

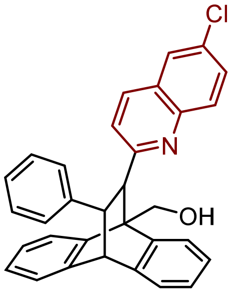


**5c:** Yellow oil; 28.8 mg, 61% yield, 92% ee, > 20:1 dr; $[\alpha]_D^{22}$ +143.3 (*c* 2.0, $CHCl_3$); $^1$H NMR (300 MHz, Chloroform-*d*) δ 7.94 – 7.83 (m, 2H), 7.79 (d, *J* = 8.7 Hz, 1H), 7.70 (s, 1H), 7.60 (d, *J* = 8.9 Hz, 1H), 7.53 (d, *J* = 7.4 Hz, 1H), 7.40 – 7.26 (m, 2H), 7.18 – 7.09 (m, 6H), 6.80 (d, *J* = 7.6 Hz, 1H), 6.71 – 6.68 (m, 2H), 6.23 (d, *J* = 8.7 Hz, 1H), 4.67 (d, *J* = 13.4 Hz, 1H), 4.55 (s, 1H), 4.41 (s, 1H), 4.02 (d, *J* = 6.2 Hz, 1H), 3.79 (d, *J* = 11.7 Hz, 1H), 3.62 (d, *J* = 6.2 Hz, 1H); $^{13}$C NMR (75 MHz, Chloroform-*d*) δ 162.3, 145.2, 145.1, 143.6, 142.9, 141.0, 139.9, 135.7, 132.1, 130.7, 130.3, 128.1, 127.9, 127.6, 126.7, 126.4, 126.3, 126.1, 126.0, 125.9, 125.6, 123.4, 123.0, 122.7, 119.8, 60.8, 56.4, 52.8, 52.1; HRMS (ESI) m/z 474.1614 $(M + H)^+$, calc. for $C_{32}H_{25}ClNO$ 474.1619.

The ee was determined by HPLC analysis: Amylose-1 (4.6 mm i.d. x 250 mm); hexane/2–propanol = 90/10; flow rate 1.0 mL/min; 25 °C; 210 nm; retention time: 27.8 min (major) and 30.0 min (minor).

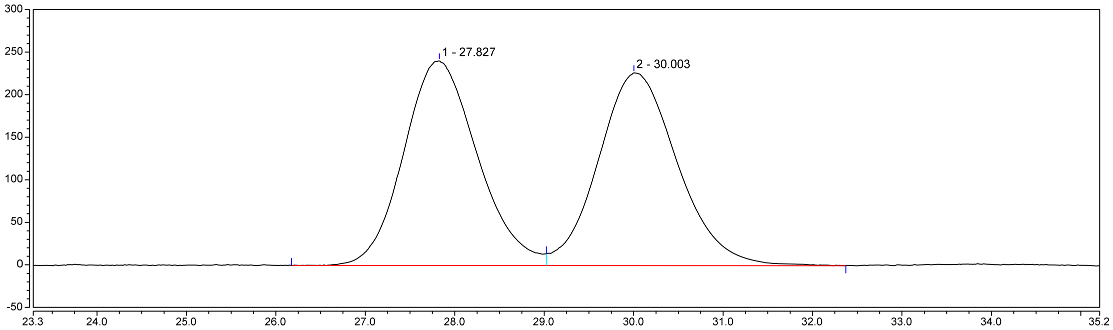


| Entry | Retention | Area | Height | %Area |
|---|---|---|---|---|
| 1 | 27.827 | 230.5003 | 240.65 | 49.80 |
| 2 | 30.003 | 232.3396 | 226.66 | 50.20 |

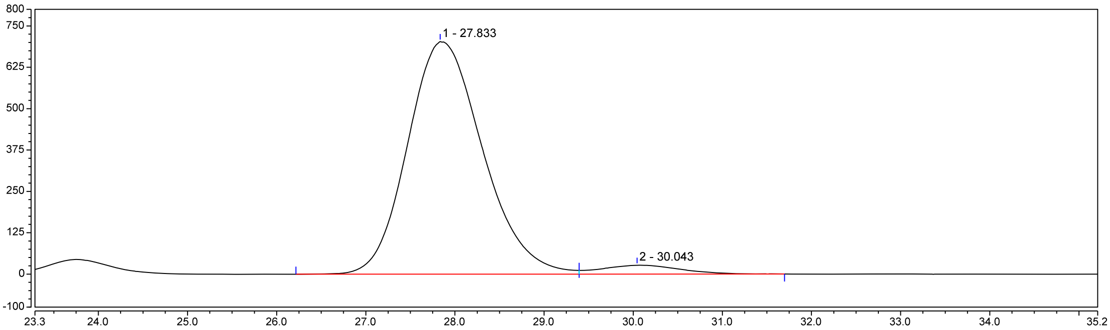


| Entry | Retention | Area | Height | %Area |
| --- | --- | --- | --- | --- |
| 1 | 27.833 | 685.8060 | 703.23 | 96.20 |
| 2 | 30.043 | 27.0720 | 26.88 | 3.80 |

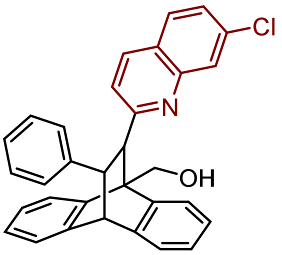


**5d:** Colorless oil; 33.1 mg, 70% yield, 95% ee, > 20:1 dr; $[\alpha]_D^{22}$ +78.2 (*c* 2.0, $CHCl_3$); $^1$H NMR (300 MHz, Chloroform-*d*) δ 7.93 (d, *J* = 2.0 Hz, 1H), 7.87 (s, 1H), 7.84 (s, 1H), 7.65 (d, *J* = 8.7 Hz, 1H), 7.52 (d, *J* = 7.3 Hz, 1H), 7.44 (dd, *J* = 8.7, 2.1 Hz, 1H), 7.33 (td, *J* = 7.4, 1.8 Hz, 1H), 7.28 (d, *J* = 7.4 Hz, 1H), 7.19 – 7.03 (m, 6H), 6.79 (d, *J* = 7.5 Hz, 1H), 6.69 (m, 2H), 6.20 (d, *J* = 8.7 Hz, 1H), 4.63 (m, 2H), 4.41 (d, *J* = 2.2 Hz, 1H), 4.00 (d, *J* = 6.1 Hz, 1H), 3.78 (d, *J* = 11.3 Hz, 1H), 3.61 (dd, *J* = 6.2, 2.3 Hz, 1H); $^{13}$C NMR (75 MHz, Chloroform-*d*) δ 163.2, 147.2, 145.1, 143.6, 142.9, 141.0, 139.9, 136.4, 135.6, 128.6, 128.1, 127.9, 127.7, 127.5, 126.7, 126.4, 126.3, 126.1, 125.8, 125.6, 125.4, 123.4, 123.0, 122.7, 119.2, 60.8, 56.5, 52.8, 52.1, 52.0; HRMS (ESI) m/z 474.1614 $(M + H)^+$, calc. for $C_{32}H_{25}ClNO$ 474.1619.

The ee was determined by HPLC analysis: Amylose-1 (4.6 mm i.d. x 250 mm); hexane/2–propanol = 90/10; flow rate 1.0 mL/min; 25 °C; 210 nm; retention time: 16.8 min (minor) and 17.7 min (major).

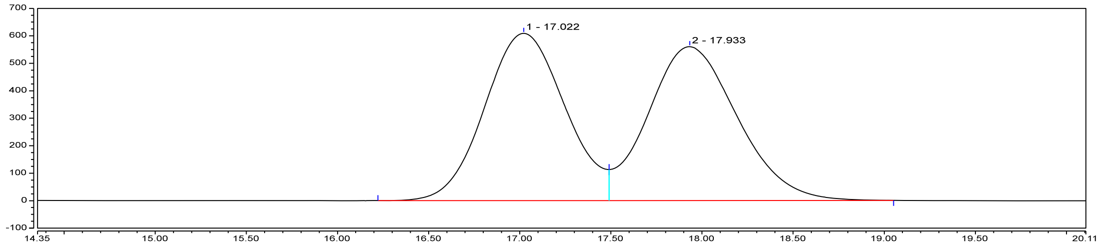


| Entry | Retention | Area | Height | %Area |
| --- | --- | --- | --- | --- |
| 1 | 17.022 | 312.0543 | 608.70 | 49.46 |
| 2 | 17.933 | 318.8576 | 560.10 | 50.54 |

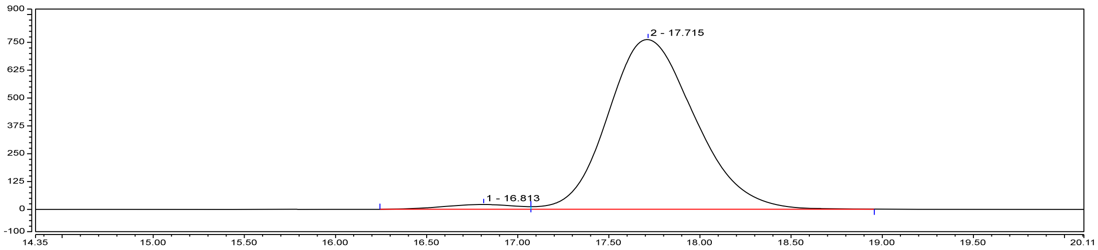

| Entry | Retention | Area | Height | %Area |
|---|---|---|---|---|
| 1 | 16.813 | 9.6856 | 21.41 | 2.24 |
| 2 | 17.715 | 421.8487 | 762.49 | 97.76 |

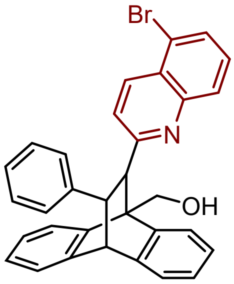


**5e:** Yellow oil; 50.6 mg, 98% yield, 90% ee, > 20:1 dr; $[\alpha]_D^{22}$ +51.9 (*c* 2.0, $CHCl_3$); $^1$H NMR (300 MHz, Chloroform-*d*) δ 8.26 (d, *J* = 8.9 Hz, 1H), 7.94 – 7.85 (m, 2H), 7.76 (d, *J* = 7.5 Hz, 1H), 7.52 (q, *J* = 8.0 Hz, 2H), 7.39 – 7.33 (m, 1H), 7.33 – 7.27 (m, 1H), 7.22 – 7.07 (m, 6H), 6.81 (d, *J* = 7.5 Hz, 1H), 6.77 – 6.64 (m, 2H), 6.31 (d, *J* = 8.9 Hz, 1H), 4.68 (m, 2H), 4.43 (d, *J* = 2.2 Hz, 1H), 4.06 (d, *J* = 6.1 Hz, 1H), 3.79 (d, *J* = 11.1 Hz, 1H), 3.65 (dd, *J* = 6.2, 2.3 Hz, 1H); $^{13}$C NMR (75 MHz, Chloroform-*d*) δ 162.9, 147.5, 145.1, 143.6, 142.9, 141.1, 139.8, 136.1, 130.1, 130.0, 128.7, 128.1, 127.9, 126.7, 126.5, 126.5, 126.3, 126.1, 125.9, 125.6, 123.4, 123.0, 122.7, 121.6, 120.2, 77.4, 77.0, 76.6, 60.8, 56.3, 52.8, 52.1, 52.0; HRMS (ESI) m/z 518.1112 $(M + H)^+$, calc. for $C_{32}H_{25}BrNO$ 518.1114.

The ee was determined by HPLC analysis: Amylose-1 (4.6 mm i.d. x 250 mm); hexane/2–propanol = 95/5; flow rate 1.0 mL/min; 25 °C; 210 nm; retention time: 14.4 min (minor) and 15.7 min (major).

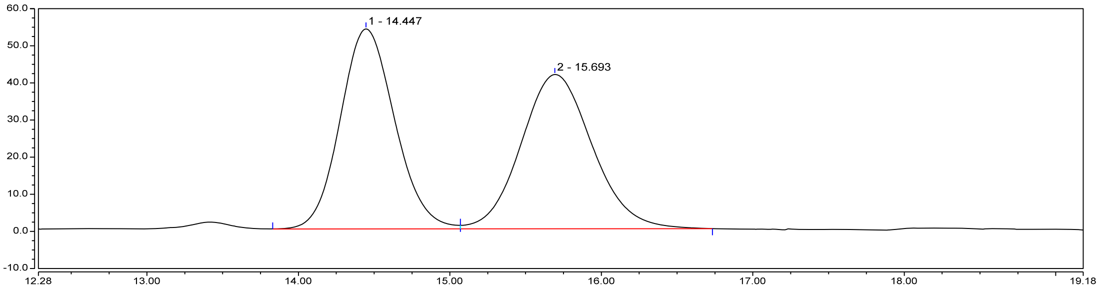

| Entry | Retention | Area | Height | %Area |
|---|---|---|---|---|
| 1 | 14.447 | 22.4971 | 53.85 | 49.82 |
| 2 | 15.693 | 22.6605 | 41.53 | 50.18 |

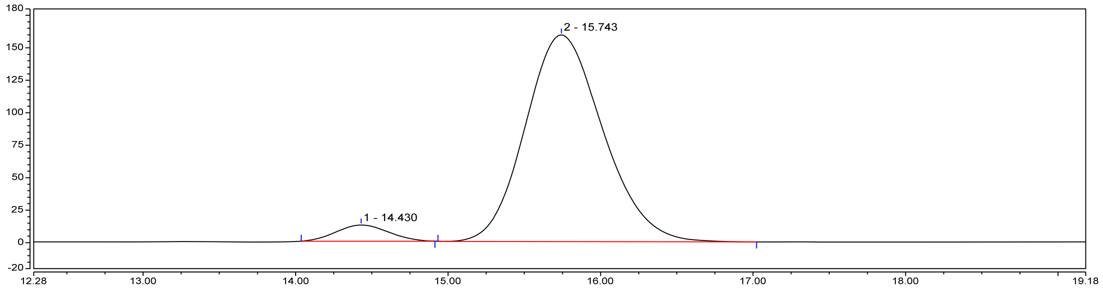

| Entry | Retention | Area | Height | %Area |
|---|---|---|---|---|
| 1 | 14.430 | 4.9487 | 12.47 | 5.00 |
| 2 | 15.743 | 93.9439 | 158.99 | 95.00 |

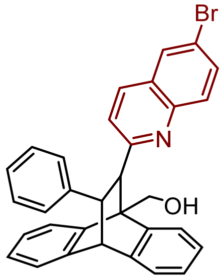

**5f:** Pale yellow solid; Mp 115.7−116.1 °C; 28.4 mg, 55% yield, 91% ee, > 20:1 dr; $[\alpha]_D^{22}$ +87.5 (*c* 2.0, $CHCl_3$); $^1$H NMR (300 MHz, Chloroform-*d*) δ 7.87 – 7.77 (m, 4H), 7.72 (dd, *J* = 9.0, 2.1 Hz, 1H), 7.53 (d, *J* = 7.3 Hz, 1H), 7.38 – 7.26 (m, 2H), 7.18 – 7.09 (m, 6H), 6.80 (d, *J* = 7.6 Hz, 1H), 6.75 – 6.60 (m, 2H), 6.23 (d, *J* = 8.7 Hz, 1H), 4.68 (d, *J* = 12.6 Hz, 1H), 4.54 (s, 1H), 4.41 (s, 1H), 4.02 (d, *J* = 6.2 Hz, 1H), 3.79 (d, *J* = 12.0 Hz, 1H), 3.62 (d, *J* = 6.2 Hz, 1H); $^{13}$C NMR (75 MHz, Chloroform-*d*) δ 162.5, 145.4, 145.1, 143.6, 142.9, 141.0, 139.9, 135.6, 133.2, 130.4, 129.4, 128.2, 128.1, 127.9, 126.7, 126.4, 126.3, 126.1, 125.9, 125.6, 123.3, 123.0, 122.7, 120.3, 119.8, 60.8, 56.5, 52.8, 52.1; HRMS (ESI) m/z 518.1112 $(M + H)^+$, calc. for $C_{32}H_{25}BrNO$ 518.1114.

The ee was determined by HPLC analysis: Amylose-1 (4.6 mm i.d. x 250 mm); hexane/2–propanol = 90/10; flow rate 1.0 mL/min; 25 °C; 210 nm; retention time: 12.3 min (major) and 21.3 min (minor).

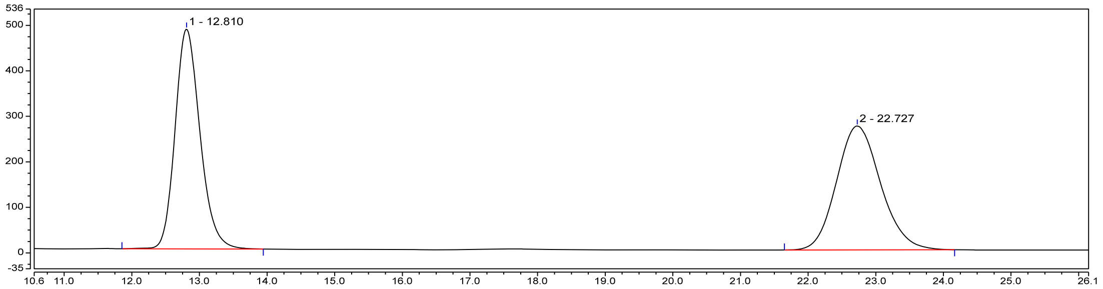

| Entry | Retention | Area | Height | %Area |
|---|---|---|---|---|
| 1 | 12.810 | 206.2803 | 482.99 | 50.09 |
| 2 | 22.727 | 205.5454 | 272.15 | 49.91 |

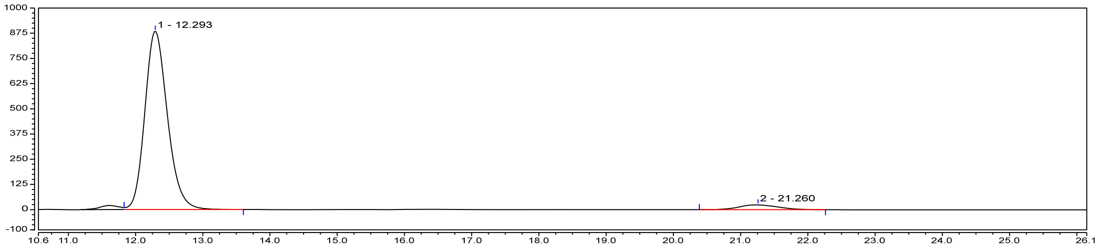


| Entry | Retention | Area | Height | %Area |
|---|---|---|---|---|
| 1 | 12.293 | 344.8734 | 884.76 | 95.53 |
| 2 | 21.260 | 16.1480 | 24.18 | 4.47 |

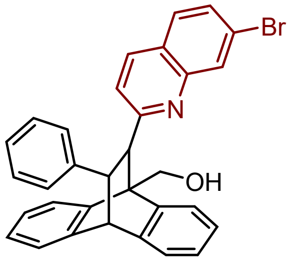


**5g:** Pale yellow solid; Mp 124.3−125.9 °C; 45.0 mg, 87% yield, 96% ee, > 20:1 dr; $[\alpha]_D^{22}$ +107.2 (*c* 2.0, $CHCl_3$); $^1$H NMR (300 MHz, Chloroform-*d*) δ 8.13 (s, 1H), 7.86 (t, *J* = 8.0 Hz, 2H), 7.57 (d, *J* = 1.2 Hz, 2H), 7.53 (d, *J* = 6.1 Hz, 1H), 7.35 (td, *J* = 7.4, 1.8 Hz, 1H), 7.29 (dd, *J* = 7.5, 1.1 Hz, 1H), 7.20 – 7.08 (m, 6H), 6.80 (d, *J* = 7.5 Hz, 1H), 6.71 (dd, *J* = 6.6, 2.9 Hz, 2H), 6.23 (d, *J* = 8.7 Hz, 1H), 4.68 (t, *J* = 11.2 Hz, 1H), 4.62 – 4.48 (m, 1H), 4.42 (d, *J* = 2.2 Hz, 1H), 4.01 (d, *J* = 6.1 Hz, 1H), 3.79 (d, *J* = 11.8 Hz, 1H), 3.62 (dd, *J* = 6.2, 2.2 Hz, 1H); $^{13}$C NMR (75 MHz, Chloroform-*d*) δ 163.1, 147.4, 145.1, 143.6, 142.9, 141.0, 139.9, 136.5, 131.0, 130.0, 128.6, 128.1, 127.9, 126.7, 126.4, 126.3, 126.1, 125.8, 125.6, 123.9, 123.3, 123.0, 122.7, 119.3, 60.8, 56.5, 52.8, 52.1, 52.0; HRMS (ESI) m/z 518.1112 $(M + H)^+$, calc. for $C_{32}H_{25}BrNO$ 518.1114.

The ee was determined by HPLC analysis: Amylose-1 (4.6 mm i.d. x 250 mm); hexane/2–propanol = 90/10; flow rate 1.0 mL/min; 25 °C; 210 nm; retention time: 16.0 min (minor) and 18.9 min (major).

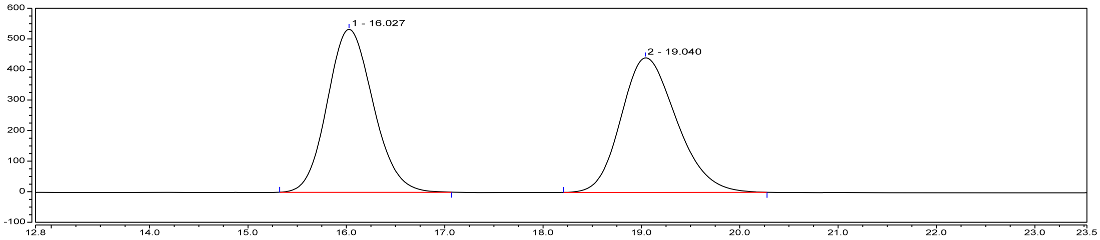


| Entry | Retention | Area | Height | %Area |
|---|---|---|---|---|
| 1 | 16.027 | 288.2066 | 533.16 | 49.98 |
| 2 | 19.040 | 288.4806 | 440.07 | 50.02 |

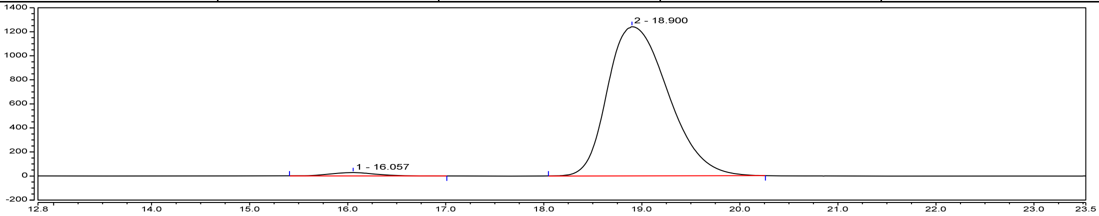

| Entry | Retention | Area | Height | %Area |
|---|---|---|---|---|
| 1 | 16.057 | 15.0622 | 27.97 | 1.66 |
| 2 | 18.900 | 890.6975 | 1242.63 | 98.34 |

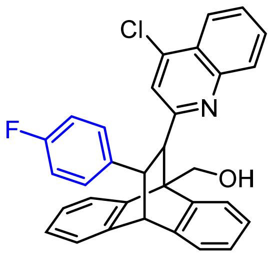


**5h:** Colorless oil; 38.8 mg, 79% yield, 92% ee, > 20:1 dr; $[\alpha]_D^{22}$ +99.2 ($c$ 2.0, $CHCl_3$); $^{1}$H NMR (300 MHz, Chloroform-*d*) δ 8.13 (d, $J$ = 8.3 Hz, 1H), 7.96 (d, $J$ = 9.0 Hz, 1H), 7.85 (d, $J$ = 7.6 Hz, 1H), 7.79 – 7.69 (m, 1H), 7.65 – 7.50 (m, 2H), 7.33 (m, 2H), 7.22 – 7.04 (m, 3H), 6.91 – 6.74 (m, 3H), 6.63 (m, 2H), 6.17 (s, 1H), 4.67 (t, $J$ = 11.5 Hz, 1H), 4.43 (s, 1H), 4.36 (d, $J$ = 2.3 Hz, 1H), 3.93 (d, $J$ = 5.9 Hz, 1H), 3.82 (d, $J$ = 13.4 Hz, 1H), 3.55 (dd, $J$ = 6.0, 2.4 Hz, 1H); $^{19}$F NMR (565 MHz, Chloroform-*d*) δ -116.25; $^{13}$C NMR (75 MHz, Chloroform-*d*) δ 161.7 (d, $J_{F\text{-}C}$ = 245.1 Hz), 162.0, 147.6, 144.6, 143.4, 143.2, 140.8, 139.6, 138.5 (d, $J_{F\text{-}C}$ = 3.2 Hz), 130.7, 129.4, 129.4 (d, $J_{F\text{-}C}$ = 7.9 Hz), 127.5, 126.6, 126.5, 126.2, 126.0, 125.8, 125.3, 123.9, 123.5, 123.1, 122.8, 119.3, 114.9 (d, $J_{F\text{-}C}$ = 21.1 Hz), 60.8, 56.6, 52.3, 52.2, 52.1; HRMS (ESI) m/z 492.1520 $(M + H)^+$, calc. for $C_{32}H_{24}ClFNO$ 492.1524.

The ee was determined by HPLC analysis: Amylose-1 (4.6 mm i.d. x 250 mm); hexane/2–propanol = 95/5; flow rate 1.0 mL/min; 25 °C; 210 nm; retention time: 13.1 min (minor) and 14.5 min (major).

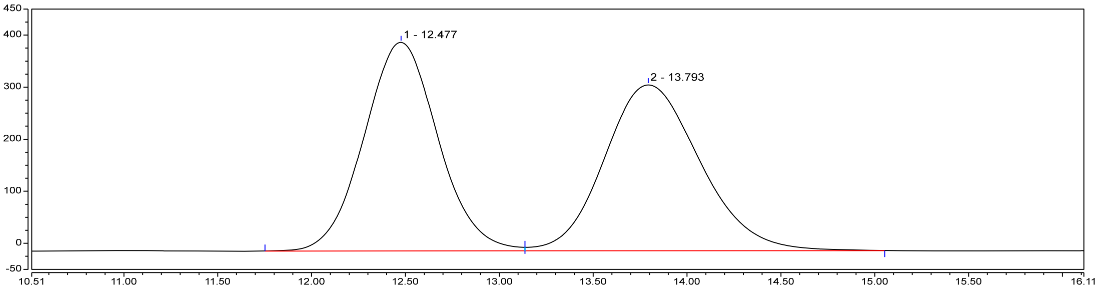


| Entry | Retention | Area | Height | %Area |
|---|---|---|---|---|
| 1 | 12.477 | 183.6215 | 400.76 | 49.35 |
| 2 | 13.793 | 188.4503 | 318.83 | 50.65 |

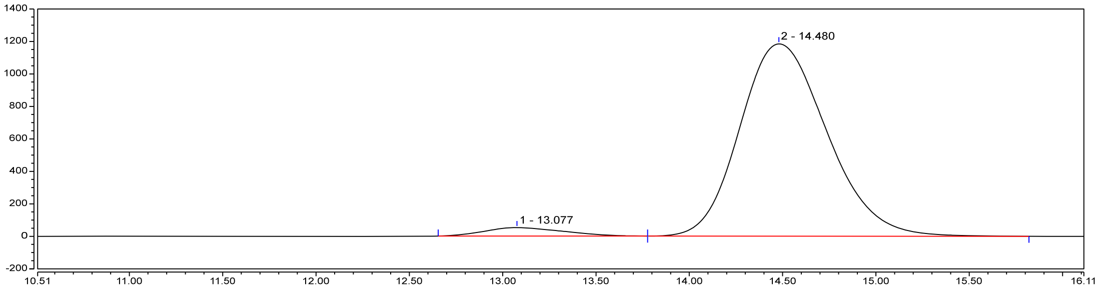


| Entry | Retention | Area | Height | %Area |
|---|---|---|---|---|
| 1 | 13.077 | 25.2273 | 52.34 | 3.78 |
| 2 | 14.480 | 642.0066 | 1184.96 | 96.22 |

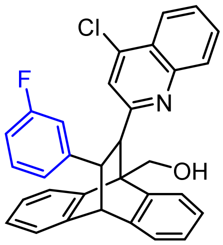


**5i:** Pale yellow oil; 37.7 mg, 81% yield, 87% ee, > 20:1 dr; $[\alpha]^{22}_{D}$ +77.4 (*c* 2.0, $CHCl_3$); $^1$H NMR (300 MHz, Chloroform-*d*) δ 8.13 (d, *J* = 8.4 Hz, 1H), 7.97 (d, *J* = 8.7 Hz, 1H), 7.85 (d, *J* = 7.6 Hz, 1H), 7.73 (m, 1H), 7.60 (m, 1H), 7.55 (d, *J* = 7.3 Hz, 1H), 7.33 (dtd, *J* = 8.7, 7.4, 1.4 Hz, 2H), 7.23 – 7.00 (m, 4H), 6.88 – 6.77 (m, 2H), 6.50 (d, *J* = 8.0 Hz, 1H), 6.37 (dt, *J* = 10.5, 2.1 Hz, 1H), 6.16 (s, 1H), 4.67 (d, *J* = 12.6 Hz, 1H), 4.41 (d, *J* = 2.3 Hz, 2H), 3.95 (d, *J* = 6.0 Hz, 1H), 3.81 (d, *J* = 12.4 Hz, 1H), 3.56 (dd, *J* = 6.1, 2.3 Hz, 1H); $^{19}$F NMR (565 MHz, Chloroform-*d*) δ -113.11; $^{13}$C NMR (75 MHz, Chloroform-*d*) δ 162.5 (d, $J_{F\text{-}C}$ = 245.2 Hz), 147.5, 145.4, 144.5, 143.3 (d, $J_{F\text{-}C}$ = 4.2 Hz), 140.7, 139.6, 130.8, 129.5 (d, $J_{F\text{-}C}$ = 8.2 Hz), 129.1, 127.6, 126.7, 126.5, 126.1, 126.1, 125.8, 125.3, 123.9, 123.6 (d, $J_{F\text{-}C}$ = 2.8 Hz), 123.5, 123.1, 122.8, 119.3, 114.8 (d, $J_{F\text{-}C}$ = 21.4 Hz), 113.6 (d, $J_{F\text{-}C}$ = 21.2 Hz), 60.7, 56.4, 52.7, 52.0, 51.8; HRMS (ESI) m/z 492.1520 $(M + H)^+$, calc. for $C_{32}H_{24}ClFNO$ 492.1524.

The ee was determined by HPLC analysis: Amylose-1 (4.6 mm i.d. x 250 mm); hexane/2–propanol = 95/5; flow rate 1.0 mL/min; 25 °C; 230 nm; retention time: 14.1 min (minor) and 15.8 min (major).

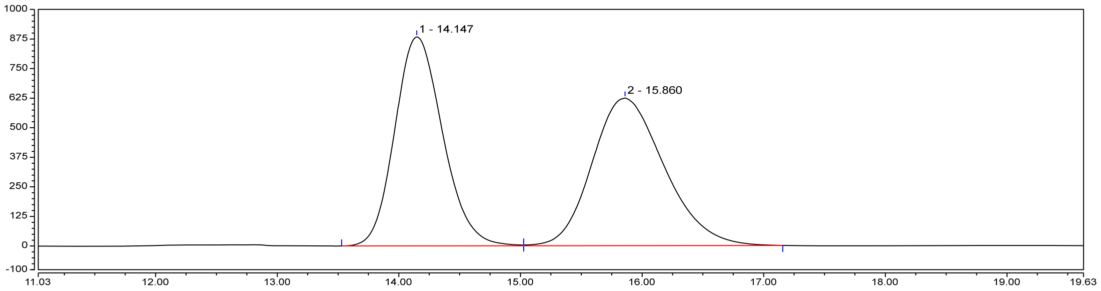


| Entry | Retention | Area | Height | %Area |
|---|---|---|---|---|
| 1 | 14.147 | 403.4443 | 883.35 | 48.45 |
| 2 | 15.860 | 429.1782 | 624.13 | 51.55 |

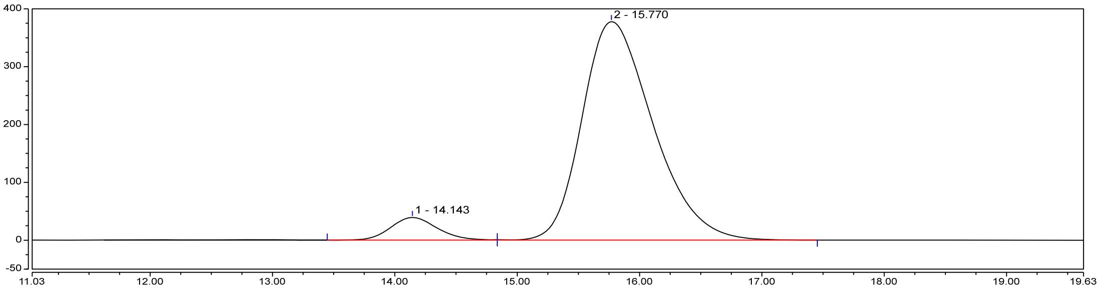


| Entry | Retention | Area | Height | %Area |
|---|---|---|---|---|
| 1 | 14.143 | 17.3122 | 39.05 | 6.38 |
| 2 | 15.770 | 254.0813 | 377.73 | 93.62 |

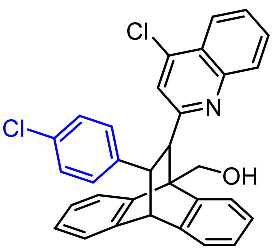


**5j:** Pale yellow oil; 39.0 mg, 77% yield, 91% ee, > 20:1 dr; $[\alpha]_D^{22}$ +127.5 (*c* 2.0, $CHCl_3$); $^1H$ NMR (300 MHz, Chloroform-*d*) δ 8.13 (d, *J* = 7.0 Hz, 1H), 7.96 (d, *J* = 8.7 Hz, 1H), 7.85 (d, *J* = 7.6 Hz, 1H), 7.73 (m, 1H), 7.60 (m, 1H), 7.55 (d, *J* = 7.3 Hz, 1H), 7.33 (m, 2H), 7.16 (t, *J* = 7.4 Hz, 2H), 7.09 (m, 3H), 6.83 (d, *J* = 7.5 Hz, 1H), 6.72 – 6.52 (m, 2H), 6.16 (s, 1H), 4.67 (t, *J* = 11.3 Hz, 1H), 4.43 (s, 1H), 4.36 (d, *J* = 2.2 Hz, 1H), 3.93 (d, *J* = 5.9 Hz, 1H), 3.81 (d, *J* = 12.3 Hz, 1H), 3.54 (dd, *J* = 6.0, 2.3 Hz, 1H); $^{13}C$ NMR (75 MHz, Chloroform-*d*) δ 161.8, 147.5, 144.5, 143.3, 143.2, 141.3, 140.7, 139.6, 132.5, 130.7, 129.3, 129.1, 128.2, 127.5, 126.7, 126.5, 126.2, 126.1, 125.8, 125.3, 123.9, 123.6, 123.1, 122.8, 119.2, 60.7, 56.5, 52.4, 52.0, 51.9; HRMS (ESI) m/z 508.1224 $(M + H)^+$, calc. for $C_{32}H_{24}Cl_2NO$ 508.1229.

The ee was determined by HPLC analysis: Amylose-1 (4.6 mm i.d. x 250 mm); hexane/2–propanol = 95/5; flow rate 1.0 mL/min; 25 °C; 210 nm; retention time: 12.6 min (major) and 14.3 min (minor).

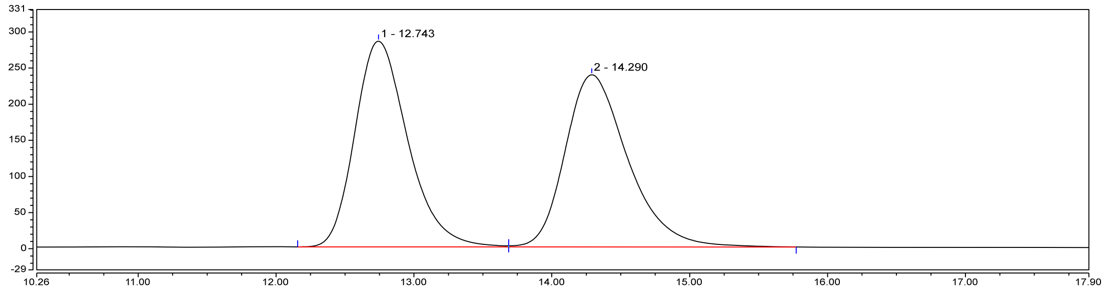


| Entry | Retention | Area | Height | %Area |
|---|---|---|---|---|
| 1 | 12.743 | 126.6333 | 284.63 | 50.13 |
| 2 | 14.290 | 125.9587 | 238.14 | 49.87 |

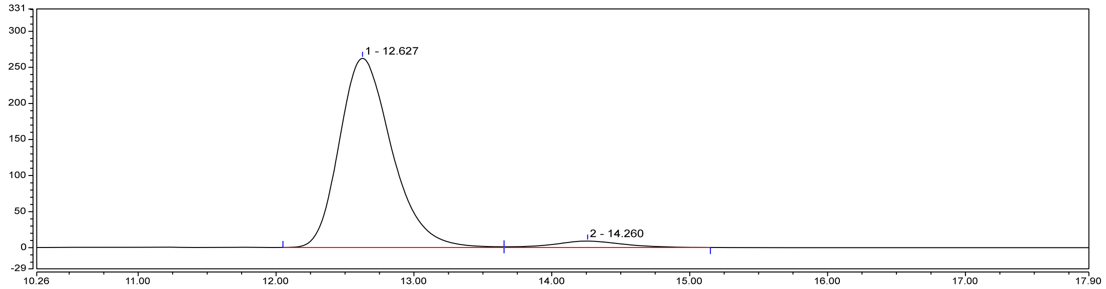


| Entry | Retention | Area | Height | %Area |
|---|---|---|---|---|
| 1 | 12.627 | 115.5926 | 262.06 | 95.76 |
| 2 | 14.260 | 5.1205 | 8.85 | 4.24 |

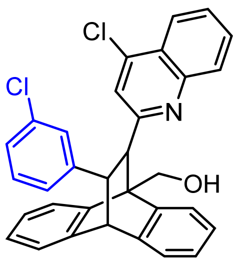


**5k:** White solid; Mp 124.7−126.1 °C; 43.1 mg, 85% yield, 86% ee, > 20:1 dr; $[\alpha]_D^{22}$ +85.1 (*c* 2.0, $CHCl_3$); $^1$H NMR (300 MHz, Chloroform-*d*) δ 8.14 (d, *J* = 8.4 Hz, 1H), 7.97 (d, *J* = 8.4 Hz, 1H), 7.87 (d, *J* = 7.6 Hz, 1H), 7.73 (t, *J* = 7.7 Hz, 1H), 7.58 (dd, *J* = 17.0, 7.9 Hz, 2H), 7.38 – 7.29 (m, 2H), 7.24 – 6.95 (m, 5H), 6.84 (d, *J* = 7.5 Hz, 1H), 6.74 (s, 1H), 6.50 (d, *J* = 7.7 Hz, 1H), 6.17 (s, 1H), 4.68 (d, *J* = 12.4 Hz, 1H), 4.39 (s, 2H), 3.96 (d, *J* = 5.9 Hz, 1H), 3.84 (d, *J* = 12.4 Hz, 1H), 3.54 (dd, *J* = 6.1, 2.4 Hz, 1H); $^{13}$C NMR (75 MHz, Chloroform-*d*) δ 161.8, 147.6, 144.9, 144.5, 143.3, 143.2, 140.6, 139.6, 133.9, 130.8, 129.4, 129.1, 128.3, 127.6, 126.9, 126.7, 126.6, 126.2, 126.1, 126.0, 125.8, 125.4, 123.9, 123.6, 123.2, 122.8, 119.3, 60.8, 56.4, 52.8, 52.1, 51. 9; HRMS (ESI) m/z 508.1225 $(M + H)^+$, calc. for $C_{32}H_{24}Cl_2NO$ 508.1229.

The ee was determined by HPLC analysis: Amylose-1 (4.6 mm i.d. x 250 mm); hexane/2–propanol = 95/5; flow rate 1.0 mL/min; 25 °C; 230 nm; retention time: 13.0 min (minor) and 15.7 min (major).

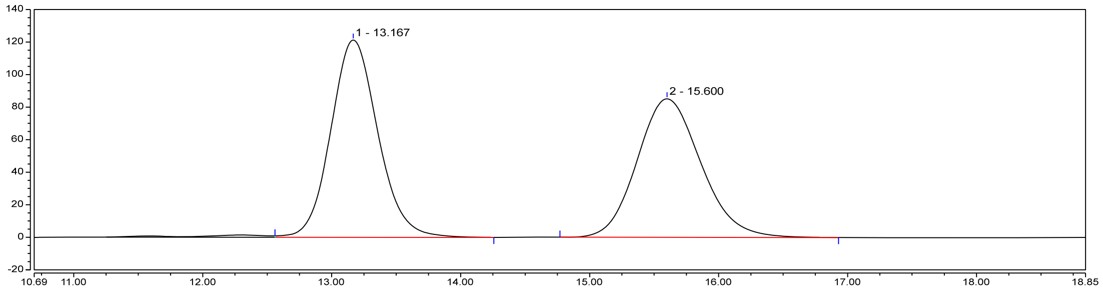


| Entry | Retention | Area | Height | %Area |
|---|---|---|---|---|
| 1 | 13.167 | 51.2841 | 121.23 | 51.24 |
| 2 | 15.600 | 48.8069 | 85.12 | 48.76 |

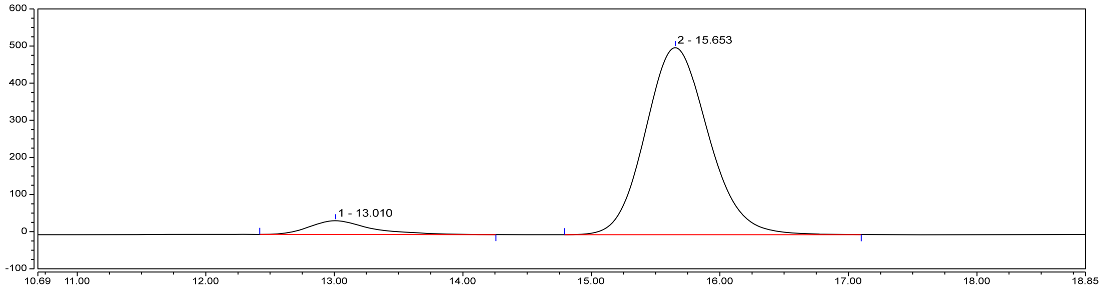


| Entry | Retention | Area | Height | %Area |
|---|---|---|---|---|
| 1 | 13.010 | 20.5189 | 37.05 | 6.69 |
| 2 | 15.653 | 286.3568 | 503.83 | 93.31 |

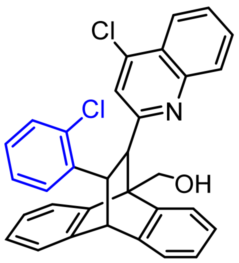


**5l:** White solid; Mp 109.0−110.5 ºC; 40.0 mg, 79% yield, 92% ee, > 20:1 dr; $[\alpha]_D^{22}$ +42.2 (*c* 2.0, $CHCl_3$); $^1H$ NMR (300 MHz, Chloroform-*d*) δ 8.12 (d, *J* = 8.3 Hz, 1H), 7.97 (d, *J* = 8.4 Hz, 1H), 7.87 (d, *J* = 7.6 Hz, 1H), 7.72 (t, *J* = 7.7 Hz, 1H), 7.64 – 7.50 (m, 2H), 7.36 – 7.25 (m, 3H), 7.19 – 7.10 (m, 2H), 7.04 (d, *J* = 8.2 Hz, 1H), 6.99 (d, *J* = 7.3 Hz, 1H), 6.93 (t, *J* = 7.6 Hz, 1H), 6.84 (d, *J* = 7.6 Hz, 1H), 6.30 – 5.97 (m, 2H), 4.71 (d, *J* = 12.5 Hz, 1H), 4.53 (s, 1H), 4.38 (s, 1H), 4.31 – 4.22 (m, 1H), 4.06 (d, *J* = 5.8 Hz, 1H), 3.87 (d, *J* = 12.2 Hz, 1H); $^{13}C$ NMR (75 MHz, Chloroform-*d*) δ 161.8, 147.4, 144.5, 143.2, 143.1, 140.7, 139.5, 139.5, 134.0, 130.7, 129.1, 129.0, 128.4, 127.7, 127.5, 126.7, 126.5, 126.5, 126.1, 126.0, 125.7, 124.0, 123.7, 123.1, 122.7, 119.4, 60.7, 55.0, 52.2, 50.5, 47.6; HRMS (ESI) m/z 508.1224 (M + H)$^+$, calc. for $C_{32}H_{24}Cl_2NO$ 508.1229.

The ee was determined by HPLC analysis: Amylose-1 (4.6 mm i.d. x 250 mm); hexane/2–propanol = 95/5; flow rate 1.0 mL/min; 25 ºC; 230 nm; retention time: 11.1 min (minor) and 16.8 min (major).

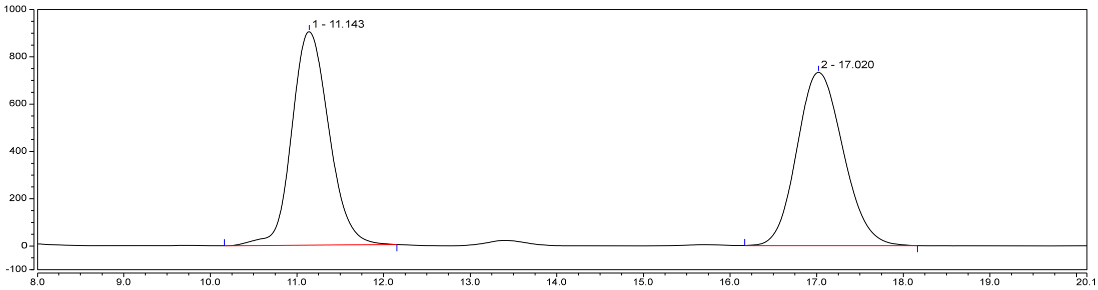


| Entry | Retention | Area | Height | %Area |
|---|---|---|---|---|
| 1 | 11.143 | 449.5921 | 902.76 | 49.82 |
| 2 | 17.020 | 452.8182 | 732.46 | 50.18 |

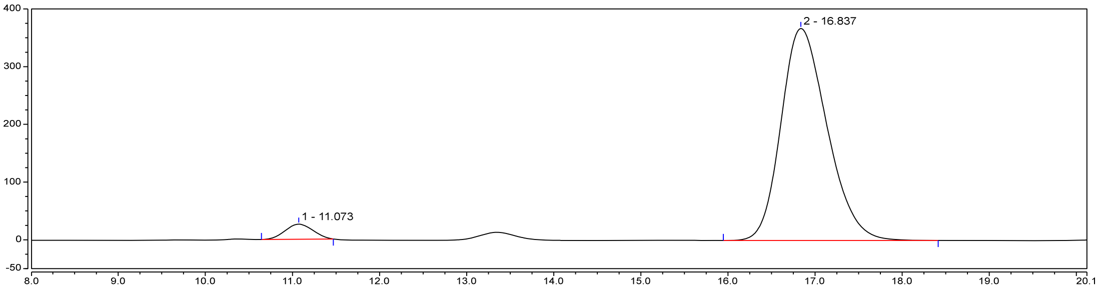


| Entry | Retention | Area | Height | %Area |
|---|---|---|---|---|
| 1 | 11.073 | 9.7119 | 26.12 | 4.13 |
| 2 | 16.837 | 225.3572 | 367.37 | 95.87 |

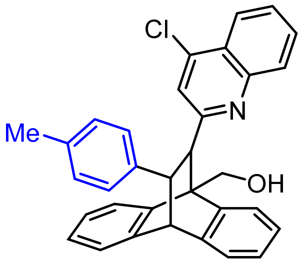


**5m:** Colorless oil; 31.1 mg, 64% yield, 91% ee, > 20:1 dr; $[\alpha]^{22}_{D}$ +113.0 (*c* 2.0, $CHCl_3$); $^1H$ NMR (300 MHz, Chloroform-*d*) δ 8.13 (d, *J* = 8.4 Hz, 1H), 7.95 (d, *J* = 7.6 Hz, 1H), 7.86 (d, *J* = 7.6 Hz, 1H), 7.72 (m, 1H), 7.58 (m, 2H), 7.40 – 7.28 (m, 2H), 7.23 – 7.08 (m, 3H), 6.94 (d, *J* = 7.9 Hz, 2H), 6.83 (d, *J* = 7.5 Hz, 1H), 6.59 (d, *J* = 8.1 Hz, 2H), 6.20 (s, 1H), 4.69 (t, *J* = 11.4 Hz, 1H), 4.57 (s, 1H), 4.39 (d, *J* = 2.2 Hz, 1H), 3.99 (d, *J* = 6.0 Hz, 1H), 3.82 (d, *J* = 12.5 Hz, 1H), 3.54 (dd, *J* = 6.1, 2.3 Hz, 1H), 2.24 (s, 3H); $^{13}C$ NMR (75 MHz, Chloroform-*d*) δ 162.2, 147.5, 144.9, 143.4, 143.1, 141.1, 139.7, 139.6, 136.2, 130.6, 129.1, 128.8, 127.8, 127.4, 126.5, 126.3, 126.2, 125.9, 125.6, 125.3, 123.9, 123.5, 123.0, 122.7, 119.3, 60.8, 56.5, 52.6, 52.2, 52.1, 20.9; HRMS (ESI) m/z 488.1772 $(M + H)^+$, calc. for $C_{33}H_{27}ClNO$ 488.1775.

The ee was determined by HPLC analysis: INA (4.6 mm i.d. x 250 mm); hexane/2–propanol = 95/5; flow rate 1.0 mL/min; 25 °C; 210 nm; retention time: 6.9 min (major) and 8.5 min (minor).

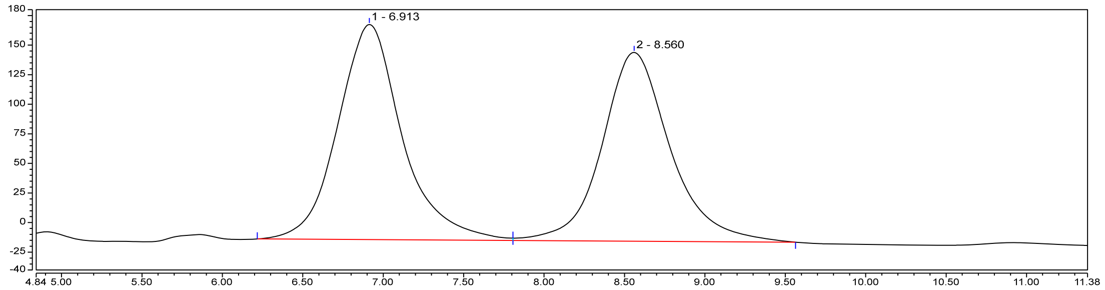


| Entry | Retention | Area | Height | %Area |
|---|---|---|---|---|
| 1 | 6.913 | 84.4834 | 181.76 | 51.83 |
| 2 | 8.560 | 78.5192 | 159.51 | 48.17 |

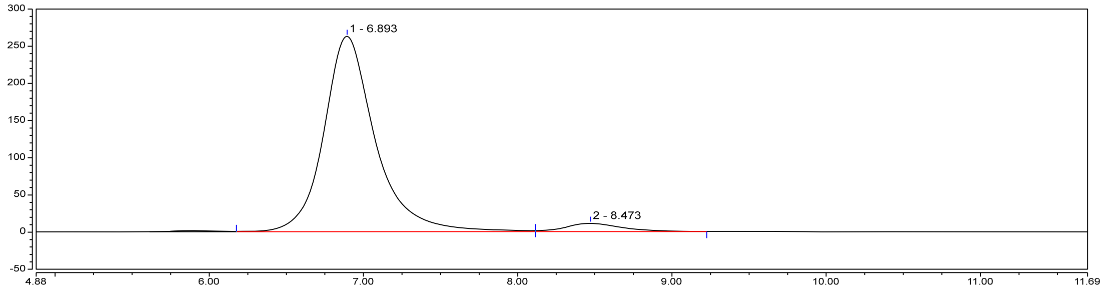


| Entry | Retention | Area | Height | %Area |
|---|---|---|---|---|
| 1 | 6.893 | 101.9083 | 262.61 | 95.66 |
| 2 | 8.473 | 4.6236 | 11.17 | 4.34 |

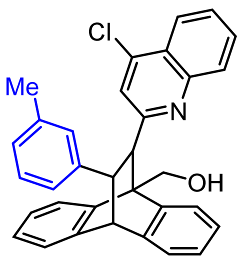


**5n:** Pale yellow oil; 29.2 mg, 60% yield, 91% ee, > 20:1 dr; $[\alpha]_D^{22}$ +86.0 (*c* 2.0, $CHCl_3$); $^1H$ NMR (300 MHz, Chloroform-*d*) δ 8.14 (d, *J* = 8.3 Hz, 1H), 7.97 (d, *J* = 8.4 Hz, 1H), 7.88 (d, *J* = 7.6 Hz, 1H), 7.76 – 7.68 (m, 1H), 7.61 – 7.54 (m, 2H), 7.38 – 7.30 (m, 2H), 7.21 – 7.10 (m, 3H), 7.06 – 6.93 (m, 2H), 6.86 (d, *J* = 7.6 Hz, 1H), 6.53 (s, 1H), 6.48 (d, *J* = 7.7 Hz, 1H), 6.22 (s, 1H), 4.70 (d, *J* = 12.4 Hz, 1H), 4.50 (s, 1H), 4.40 (s, 1H), 4.03 (d, *J* = 6.0 Hz, 1H), 3.86 (d, *J* = 12.3 Hz, 1H), 3.55 (d, *J* = 7.4 Hz, 1H), 2.20 (s, 3H); $^{13}C$ NMR (75 MHz, Chloroform-*d*) δ 162.2, 147.5, 144.9, 143.4, 143.1, 142.7, 141.1, 139.7, 137.6, 130.6, 129.1, 129.0, 128.0, 127.4, 126.6, 126.3, 126.2, 125.9, 125.6, 125.3, 124.9, 123.9, 123.5, 123.0, 122.7, 119.4, 60.8, 56.3, 53.0, 52.2, 52.1, 21.3; HRMS (ESI) m/z 488.1771 $(M + H)^+$, calc. for $C_{33}H_{27}ClNO$ 488.1775.

The ee was determined by HPLC analysis: Amylose-1 (4.6 mm i.d. x 250 mm); hexane/2–propanol = 95/5; flow rate 1.0 mL/min; 25 °C; 210 nm; retention time: 10.6 min (minor) and 13.3 min (major).

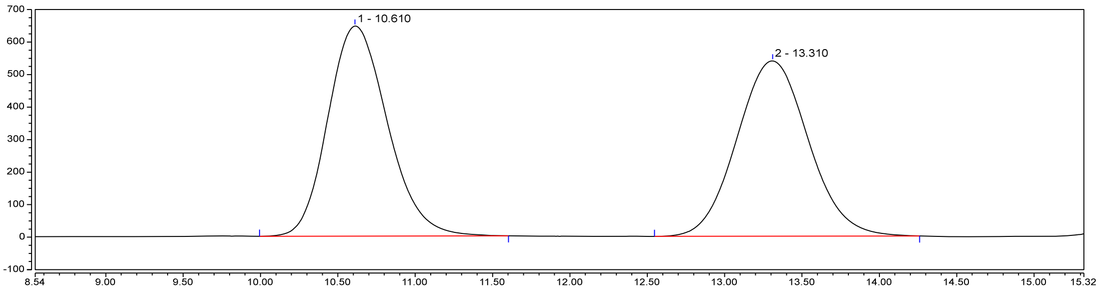


| Entry | Retention | Area | Height | %Area |
|---|---|---|---|---|
| 1 | 10.610 | 288.7458 | 645.87 | 49.95 |
| 2 | 13.310 | 289.3039 | 539.33 | 50.05 |

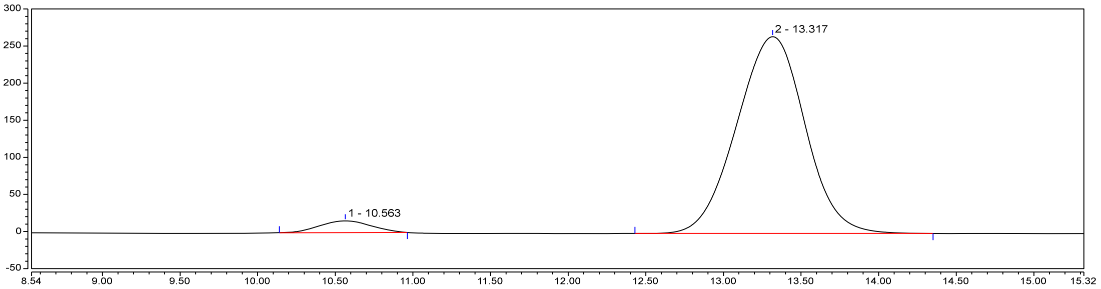


| Entry | Retention | Area | Height | %Area |
|---|---|---|---|---|
| 1 | 10.563 | 6.0899 | 15.82 | 4.30 |
| 2 | 13.317 | 135.5185 | 265.26 | 95.70 |

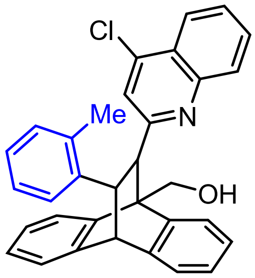

**5o:** Pale yellow solid; Mp 122.4−123.8 °C; 35.1 mg, 72% yield, 92% ee, > 20:1 dr; $[\alpha]_D^{22}$ +157.8 (*c* 2.0, $CHCl_3$); $^1H$ NMR (300 MHz, Chloroform-*d*) δ 8.12 (d, *J* = 8.4 Hz, 1H), 7.95 (d, *J* = 8.4 Hz, 1H), 7.88 (d, *J* = 7.6 Hz, 1H), 7.72 (m, 1H), 7.63 – 7.52 (m, 2H), 7.34 (q, *J* = 8.1, 7.2 Hz, 2H), 7.16 (q, *J* = 7.6 Hz, 2H), 7.10 – 6.97 (m, 3H), 6.87 (m, 2H), 6.23 (s, 1H), 6.10 (d, *J* = 7.8 Hz, 1H), 4.69 (q, *J* = 10.3, 9.8 Hz, 1H), 4.52 (s, 1H), 4.27 (d, *J* = 2.1 Hz, 1H), 4.07 (d, *J* = 6.0 Hz, 1H), 3.93 – 3.78 (m, 2H), 2.36 (s, 3H); $^{13}C$ NMR (75 MHz, Chloroform-*d*) δ 162.3, 147.5, 145.1, 143.3, 143.1, 141.0, 140.5, 139.7, 135.4, 130.7, 130.0, 129.1, 127.4, 126.8, 126.6, 126.4, 126.3, 126.2, 125.9, 125.8, 125.7, 125.3, 123.9, 123.2, 123.0, 122.8, 119.2, 60.8, 55.7, 52.2, 51.1, 47.6, 20.2; HRMS (ESI) m/z 488.1771 $(M + H)^+$, calc. for $C_{33}H_{27}ClNO$ 488.1775.

The ee was determined by HPLC analysis: Amylose-1 (4.6 mm i.d. x 250 mm); hexane/2–propanol = 95/5; flow rate 1.0 mL/min; 25 °C; 230 nm; retention time: 10.3 min (minor) and 16.5 min (major).

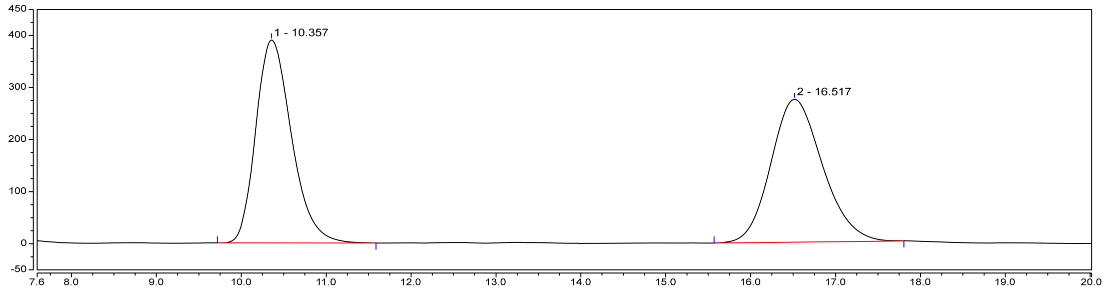

| Entry | Retention | Area | Height | %Area |
|---|---|---|---|---|
| 1 | 10.357 | 190.1858 | 389.88 | 49.60 |
| 2 | 16.517 | 193.2720 | 274.27 | 50.40 |

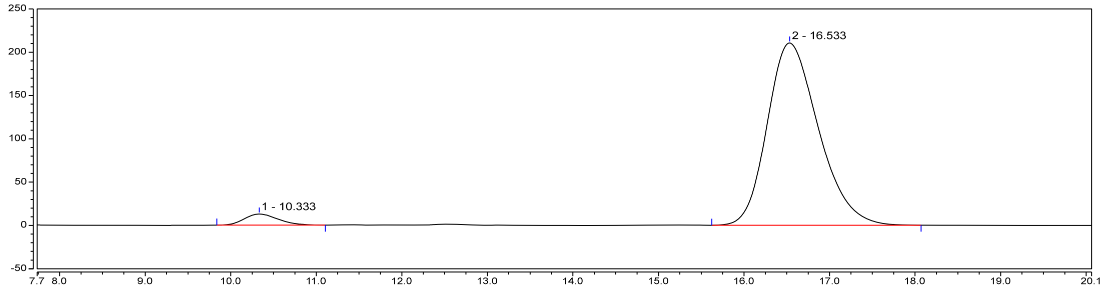

| Entry | Retention | Area | Height | %Area |
|---|---|---|---|---|
| 1 | 10.333 | 5.8605 | 12.80 | 3.86 |
| 2 | 16.533 | 145.8677 | 210.32 | 96.14 |

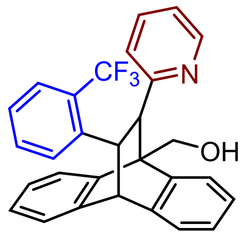


**5p:** Yellow oil; 30.1 mg, 66% yield, 94% ee, > 20:1 dr; $[\alpha]_D^{22}$ +107.7 (*c* 2.0, $CHCl_3$); $^1$H NMR (300 MHz, Chloroform-*d*) δ 8.40 (dd, *J* = 4.9, 1.7 Hz, 1H), 7.88 (d, *J* = 7.6 Hz, 1H), 7.56 (dd, *J* = 7.1, 2.2 Hz, 1H), 7.45 (m, 2H), 7.36 (td, *J* = 7.6, 1.3 Hz, 1H), 7.27 (d, *J* = 5.6 Hz, 1H), 7.22 – 7.15 (m, 2H), 7.15 – 7.04 (m, 3H), 6.99 (d, *J* = 7.3 Hz, 1H), 6.82 (d, *J* = 7.5 Hz, 1H), 6.14 (t, *J* = 6.9 Hz, 2H), 4.68 (d, *J* = 12.5 Hz, 1H), 4.26 (s, 1H), 3.99 – 3.85 (m, 2H), 3.80 (d, *J* = 12.5 Hz, 1H); $^{19}$F NMR (565 MHz, Chloroform-*d*) δ -58.16; $^{13}$C NMR (75 MHz, Chloroform-*d*) δ 160.4, 148.3, 144.7, 143.6, 141.4 (q, $J_{F\text{-}C}$ = 8.2 Hz), 140.4, 139.5, 136.7, 131.4, 129.0, 128.5 (d, $J_{F\text{-}C}$ = 28.8 Hz), 126.6, 126.5, 126.4, 126.3, 126.2 (q, $J_{F\text{-}C}$ = 271.4 Hz), 125.8, 125.6, 125.4 (q, $J_{F\text{-}C}$ = 6.1 Hz), 123.3, 123.2, 122.8, 122.3, 121.3, 60.6, 56.0, 52.5, 51.8, 47.1; HRMS (ESI) m/z 458.1721 $(M + H)^+$, calc. for $C_{29}H_{23}F_3NO$ 458.1726.

The ee was determined by HPLC analysis: Cellulose-4 (4.6 mm i.d. x 250 mm); hexane/2–propanol = 95/5; flow rate 1.0 mL/min; 25 °C; 210 nm; retention time: 12.2 min (minor) and 19.1 min (major).

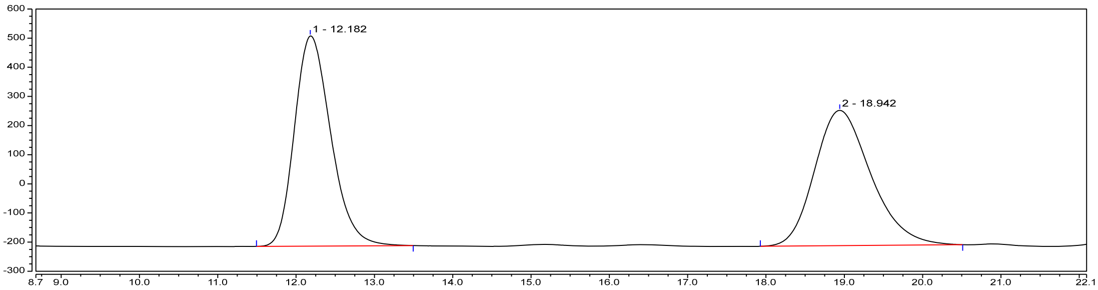


| Entry | Retention | Area | Height | %Area |
|---|---|---|---|---|
| 1 | 12.182 | 388.0258 | 721.12 | 50.09 |
| 2 | 18.942 | 386.5981 | 464.38 | 49.91 |

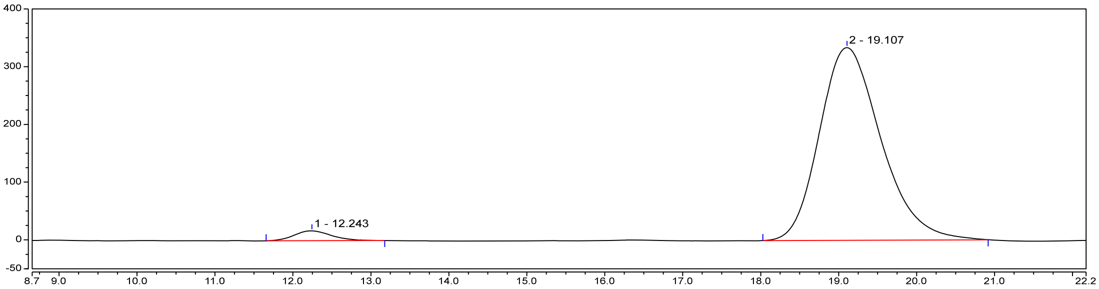


| Entry | Retention | Area | Height | %Area |
|---|---|---|---|---|
| 1 | 12.243 | 9.3807 | 17.10 | 2.98 |
| 2 | 19.107 | 305.6784 | 334.07 | 97.02 |

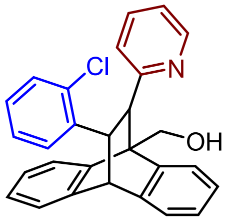


**5q:** Pale yellow oil; 30.8 mg, 73% yield, 91% ee, > 20:1 dr; $[\alpha]_D^{22}$ +100.7 (*c* 2.0, $CHCl_3$); $^1H$ NMR (300 MHz, Chloroform-*d*) δ 8.42 (d, *J* = 5.0 Hz, 1H), 7.85 (d, *J* = 7.6 Hz, 1H), 7.52 (d, *J* = 7.3 Hz, 1H), 7.42 (t, *J* = 7.8 Hz, 1H), 7.33 – 7.25 (m, 3H), 7.16 – 7.00 (m, 4H), 6.92 (m, 2H), 6.81 (d, *J* = 7.6 Hz, 1H), 6.18 (dd, *J* = 11.7, 8.0 Hz, 2H), 4.67 (d, *J* = 12.4 Hz, 1H), 4.29 (s, 1H), 4.20 (dd, *J* = 6.2, 2.2 Hz, 1H), 3.94 – 3.77 (m, 2H); $^{13}C$ NMR (75 MHz, Chloroform-*d*) δ 161.0, 148.2, 144.8, 143.5, 140.6, 139.8, 139.7, 136.8, 134.0, 129.1, 128.4, 127.6, 126.5, 126.4, 125.9, 125.8, 125.5, 123.3, 123.1, 122.8, 122.2, 121.3, 60.6, 54.2, 52.4, 50.6, 47.7; HRMS (ESI) m/z 424.1460 $(M + H)^+$, calc. for $C_{28}H_{23}ClNO$ 424.1462.

The ee was determined by HPLC analysis: Amylose-1 (4.6 mm i.d. x 250 mm); hexane/2–propanol = 90/10; flow rate 1.0 mL/min; 25 °C; 210 nm; retention time: 7.6 min (minor) and 9.3 min (major).

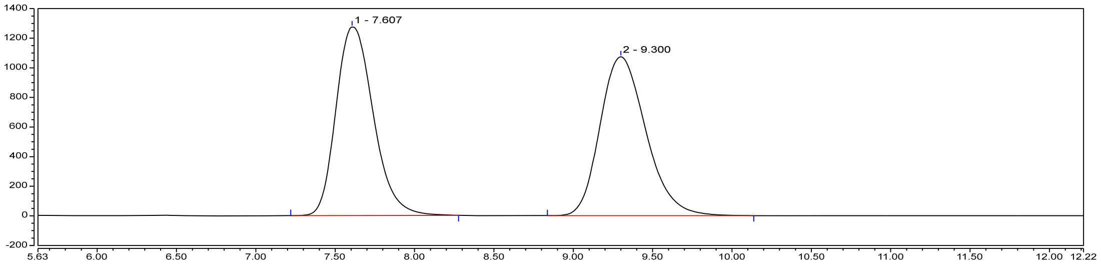


| Entry | Retention | Area | Height | %Area |
|---|---|---|---|---|
| 1 | 7.607 | 347.6033 | 1273.84 | 49.10 |
| 2 | 9.300 | 360.3029 | 1073.42 | 50.90 |

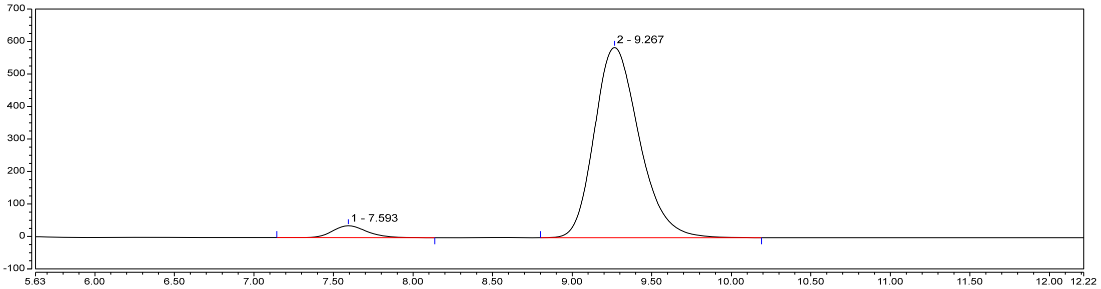


| Entry | Retention | Area | Height | %Area |
|---|---|---|---|---|
| 1 | 7.593 | 9.1985 | 36.56 | 4.59 |
| 2 | 9.267 | 191.1599 | 585.89 | 95.41 |

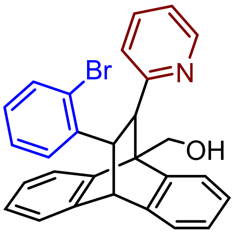


**5r:** Pale yellow oil; 27.1 mg, 58% yield, 92% ee, > 20:1 dr; $[\alpha]_D^{22}$ +93.3 (*c* 2.0, $CHCl_3$); $^1H$ NMR (300 MHz, Chloroform-*d*) δ 8.42 (d, *J* = 5.1 Hz, 1H), 7.85 (d, *J* = 7.6 Hz, 1H), 7.60 – 7.48 (m, 2H), 7.43 (t, *J* = 7.9 Hz, 1H), 7.35 – 7.26 (m, 2H), 7.09 (q, *J* = 7.4 Hz, 3H), 7.02 – 6.90 (m, 3H),

6.81 (d, *J* = 7.6 Hz, 1H), 6.21 (d, *J* = 8.1 Hz, 1H), 6.17 – 6.07 (m, 1H), 4.68 (d, *J* = 12.5 Hz, 1H), 4.31 (s, 1H), 4.19 (dd, *J* = 6.2, 2.2 Hz, 1H), 3.92 – 3.74 (m, 2H); $^{13}$C NMR (75 MHz, Chloroform-*d*) δ 160.9, 148.2, 144.7, 143.5, 141.3, 140.5, 139.7, 136.8, 132.4, 128.6, 128.0, 127.1, 126.4, 125.9, 125.8, 125.5, 125.4, 123.3, 123.1, 122.8, 122.2, 121.4, 60.6, 54.3, 52.5, 50.7, 50.6; HRMS (ESI) m/z 468.0953 (M + H)$^+$, calc. for $C_{28}H_{23}BrNO$ 468.0957.

The ee was determined by HPLC analysis: Amylose-1 (4.6 mm i.d. x 250 mm); hexane/2–propanol = 95/5; flow rate 1.0 mL/min; 25 °C; 230 nm; retention time: 10.5 min (minor) and 14.2 min (major).

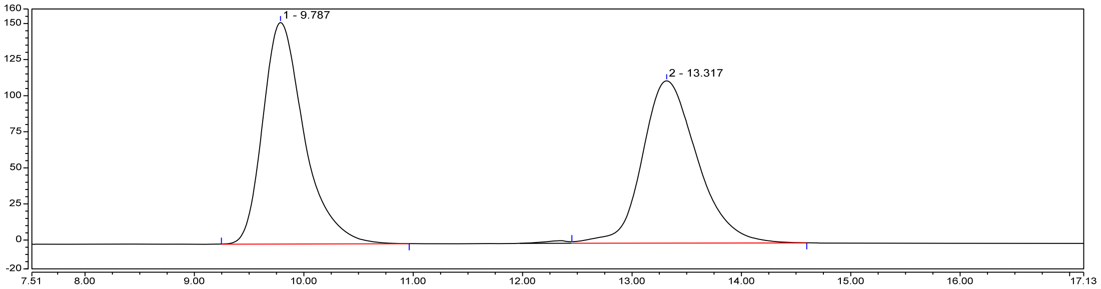


| Entry | Retention | Area | Height | %Area |
|---|---|---|---|---|
| 1 | 9.787 | 67.2642 | 153.39 | 50.68 |
| 2 | 13.317 | 65.4618 | 112.42 | 49.32 |

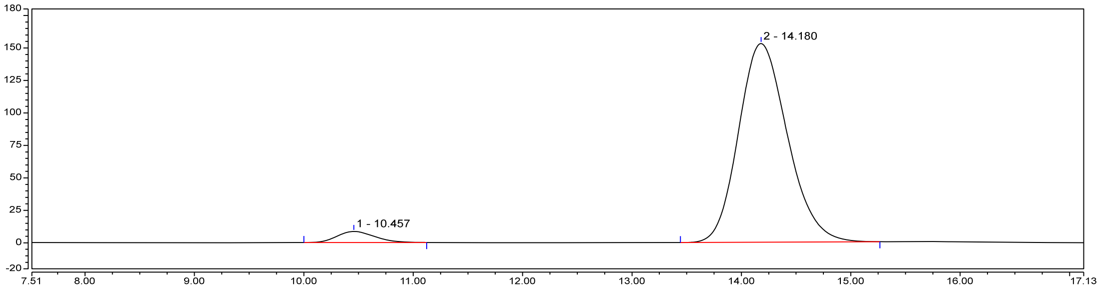


| Entry | Retention | Area | Height | %Area |
|---|---|---|---|---|
| 1 | 10.457 | 3.3852 | 8.51 | 4.10 |
| 2 | 14.180 | 79.2791 | 152.91 | 95.90 |

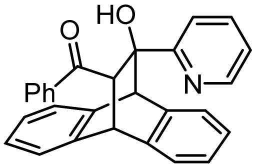


**7a:** White solid; Mp 171.4−173.1 °C; 36.6 mg, 91% yield, 98% ee, >20:1 dr; $[\alpha]_D^{22}$ +35.8 (*c* 2.0, $CHCl_3$); $^1$H NMR (300 MHz, Chloroform-*d*) δ 8.37 (d, *J* = 4.8 Hz, 1H), 7.75 (d, *J* = 7.4 Hz, 1H), 7.62 (t, *J* = 7.8 Hz, 1H), 7.46 (d, *J* = 7.4 Hz, 1H), 7.39 – 7.20 (m, 8H), 7.10 (q, *J* = 7.4 Hz, 3H), 6.98 (d, *J* = 7.4 Hz, 1H), 6.60 (d, *J* = 8.1 Hz, 1H), 5.24 (s, 1H), 4.83 (s, 1H), 4.52 (s, 1H), 4.16 (s, 1H); $^{13}$C NMR (75 MHz, Chloroform-*d*) δ 198.8, 162.2, 146.8, 143.8, 141.3, 140.8, 140.5, 138.3, 136.7, 132.1, 127.9, 127.8, 126.7, 126.7, 126.2, 126.1, 125.8, 125.7, 123.1, 122.3, 120.5, 79.2, 59.7, 57.9, 46.9; HRMS (ESI) m/z 404.1646 (M + H)$^+$, calc. for $C_{28}H_{22}NO_2$ 404.1645.

The ee was determined by HPLC analysis: Amylose-1 (4.6 mm i.d. x 250 mm); hexane/2–propanol = 70/30; flow rate 1.0 mL/min; 25 °C; 210 nm; retention time: 13.9 min (minor) and 15.5 min (major).

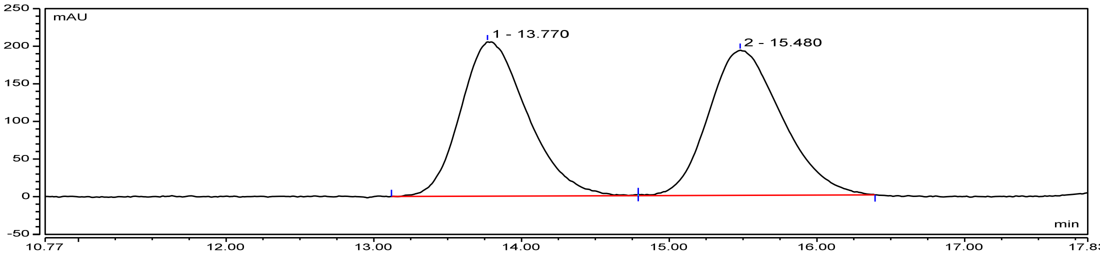


| Entry | Retention | Area | Height | %Area |
|---|---|---|---|---|
| 1 | 13.770 | 107.6116 | 205.21 | 49.19 |
| 2 | 15.480 | 111.1722 | 192.74 | 50.81 |

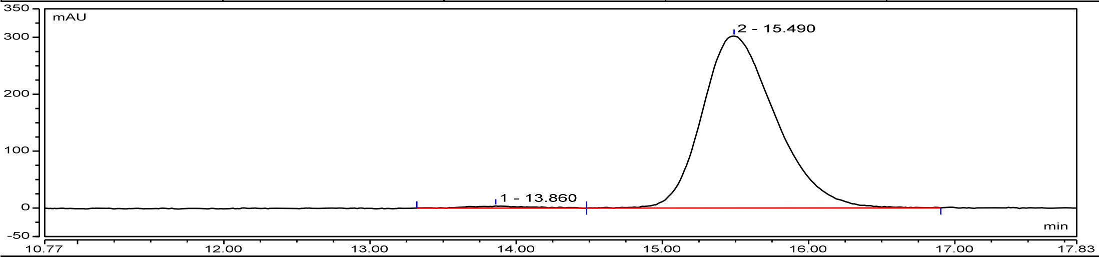


| Entry | Retention | Area | Height | %Area |
|---|---|---|---|---|
| 1 | 13.860 | 0.4582 | 0.90 | 0.87 |
| 2 | 15.490 | 52.4441 | 90.52 | 99.13 |

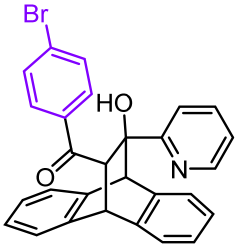


**7b:** White solid; Mp 179.3−181.1 °C; 41.0 mg, 85% yield, 98% ee, >20:1 dr; $[\alpha]_D^{22}$ +37.3 (*c* 2.0, $CHCl_3$); $^1H$ NMR (300 MHz, Chloroform-*d*) δ 8.38 (d, *J* = 4.8 Hz, 1H), 7.72 (d, *J* = 7.3 Hz, 1H), 7.59 (t, *J* = 7.8 Hz, 1H), 7.42 (d, *J* = 7.3 Hz, 1H), 7.32 (t, *J* = 7.7 Hz, 2H), 7.24 – 7.15 (m, 4H), 7.09 (d, *J* = 8.1 Hz, 3H), 6.94 (d, *J* = 7.3 Hz, 1H), 6.50 (d, *J* = 8.1 Hz, 1H), 5.16 (s, 1H), 4.79 (s, 1H), 4.37 (s, 1H), 4.10 (s, 1H); $^{13}C$ NMR (75 MHz, Chloroform-*d*) δ 197.6, 161.9, 146.9, 143.7, 141.2, 140.7, 140.4, 137.1, 136.9, 131.0, 129.3, 127.1, 126.8, 126.8, 126.3, 126.2, 125.9, 125.7, 123.2, 122.5, 120.5, 79.2, 59.6, 58.2, 46.7; HRMS (ESI) m/z 482.0754 $(M + H)^+$, calc. for $C_{28}H_{21}BrNO_2$ 482.0750.

The ee was determined by HPLC analysis: CHIRALPAK INB (4.6 mm i.d. x 250 mm); hexane/2–propanol = 70/30; flow rate 1.0 mL/min; 25 °C; 210 nm; retention time: 7.6 min (major) and 12.0 min (minor).

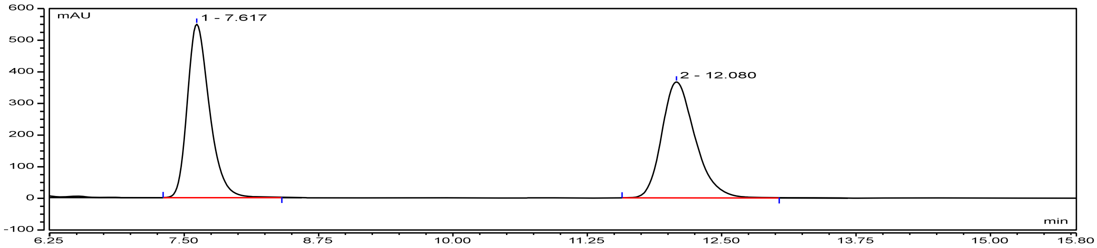


| Entry | Retention | Area | Height | %Area |
|---|---|---|---|---|
| 1 | 7.617 | 131.6997 | 548.39 | 49.92 |
| 2 | 12.080 | 132.1387 | 366.94 | 50.08 |

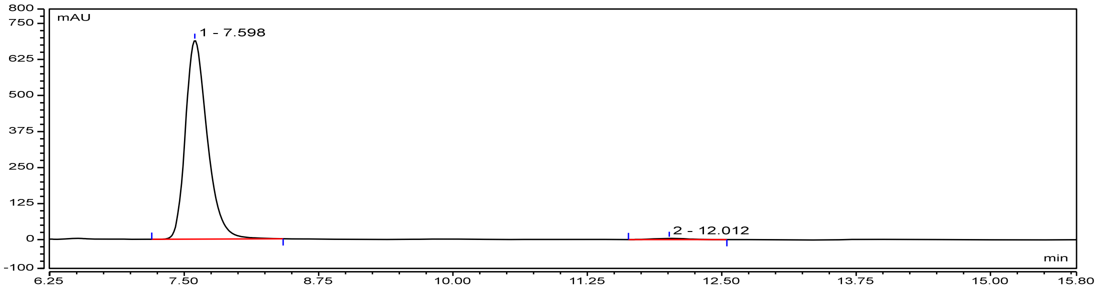


| Entry | Retention | Area | Height | %Area |
|---|---|---|---|---|
| 1 | 7.598 | 162.8977 | 689.63 | 99.10 |
| 2 | 12.012 | 1.4859 | 4.20 | 0.90 |

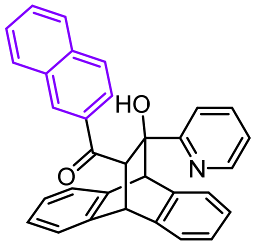


**7c:** White solid; Mp 198.2−199.7 °C; 42.6 mg, 94% yield, 97% ee, >20:1 dr; $[\alpha]^{22}_{D}$ –5.2 (*c* 2.0, $CHCl_3$);; $^1$H NMR (300 MHz, Chloroform-*d*) δ 8.30 (d, *J* = 5.9 Hz, 1H), 7.78 (d, *J* = 7.4 Hz, 1H), 7.71 (d, *J* = 8.2 Hz, 1H), 7.69 – 7.60 (m, 3H), 7.50 – 7.38 (m, 3H), 7.33 (q, *J* = 7.5, 7.0 Hz, 3H), 7.28 – 7.19 (m, 3H), 7.13 (dd, *J* = 18.6, 8.5 Hz, 2H), 6.99 (d, *J* = 7.4 Hz, 1H), 6.60 (d, *J* = 8.1 Hz, 1H), 5.16 (s, 1H), 4.87 (s, 1H), 4.59 (s, 1H), 4.16 (s, 1H); $^{13}$C NMR (75 MHz, Chloroform-*d*) δ 198.3, 162.4, 146.9, 143.9, 141.4, 140.9, 140.5, 136.8, 135.5, 135.0, 132.0, 129.6, 129.0, 128.0, 127.8, 127.5, 126.9, 126.7, 126.3, 126.2, 126.1, 125.9, 125.8, 125.7, 124.1, 123.2, 122.4, 120.7, 79.4, 7 59.6, 58.5, 46.9; HRMS (ESI) m/z 454.1799 $(M + H)^+$, calc. for $C_{32}H_{24}NO_2$ 454.1802.

The ee was determined by HPLC analysis: CHIRALPAK INB (4.6 mm i.d. x 250 mm); hexane/2–propanol = 50/50; flow rate 1.0 mL/min; 25 ºC; 210 nm; retention time: 7.9 min (major) and 11.8 min (minor).

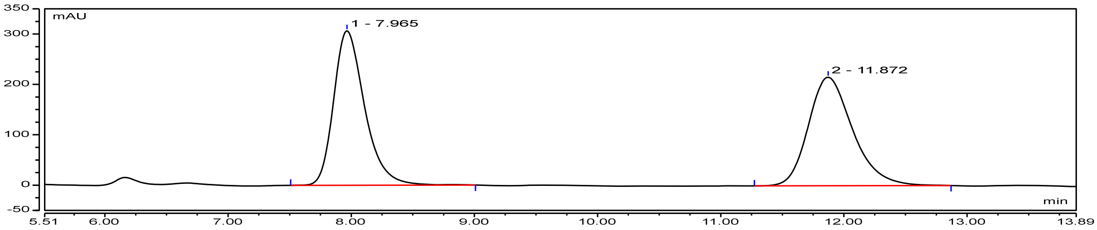

| Entry | Retention | Area | Height | %Area |
|---|---|---|---|---|
| 1 | 7.965 | 89.4750 | 306.60 | 49.89 |
| 2 | 11.872 | 89.8619 | 215.42 | 50.11 |

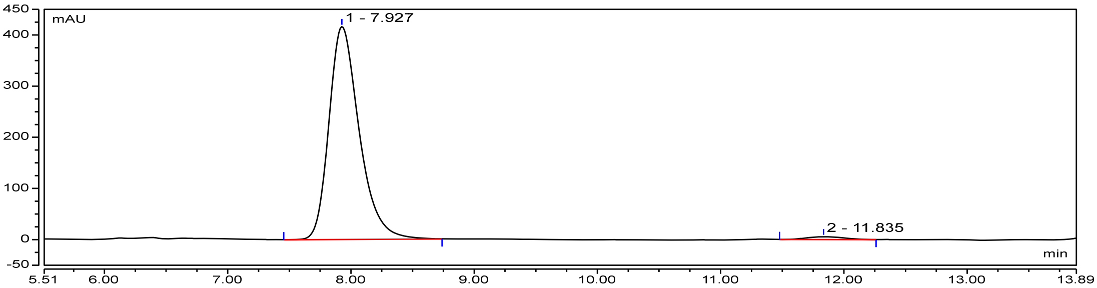


| Entry | Retention | Area | Height | %Area |
|---|---|---|---|---|
| 1 | 7.927 | 118.1597 | 415.85 | 98.51 |
| 2 | 11.835 | 1.7873 | 5.32 | 1.49 |

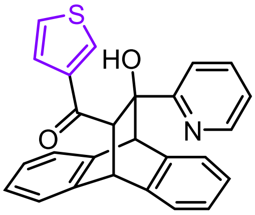

**7d:** Pale yellow solid; Mp 170.2−172.1 °C; 37.2 mg, 91% yield, 99% ee, >20:1 dr; $[\alpha]_D^{22}$ +58.9 (*c* 2.0, $CHCl_3$); $^1H$ NMR (300 MHz, Chloroform-*d*) δ 8.40 (d, *J* = 4.8 Hz, 1H), 7.70 (d, *J* = 7.4 Hz, 1H), 7.61 (t, *J* = 7.9 Hz, 1H), 7.39 (dd, *J* = 13.5, 7.3 Hz, 2H), 7.30 (t, *J* = 7.7 Hz, 1H), 7.25 – 7.16 (m, 3H), 7.15 – 7.07 (m, 2H), 7.08 – 6.97 (m, 2H), 6.92 (d, *J* = 7.4 Hz, 1H), 6.70 (d, *J* = 8.1 Hz, 1H), 4.95 (s, 1H), 4.76 (s, 1H), 4.43 (s, 1H), 4.16 (s, 1H); $^{13}C$ NMR (75 MHz, Chloroform-*d*) δ 192.8, 162.3, 146.9, 143.6, 143.2, 141.3, 140.6, 140.2, 136.8, 131.3, 127.0, 126.6, 126.3, 126.1, 125.8, 125.8, 125.4, 123.1, 122.5, 120.6, 79.6, 59.8, 58.8, 47.0; HRMS (ESI) m/z 410.1212 $(M + H)^+$, calc. for $C_{26}H_{20}NO_2S$ 410.1209.

The ee was determined by HPLC analysis: CHIRALPAK INB (4.6 mm i.d. x 250 mm); hexane/2–propanol = 70/30; flow rate 1.0 mL/min; 25 °C; 210 nm; retention time: 8.2 min (major) and 12.4 min (minor).

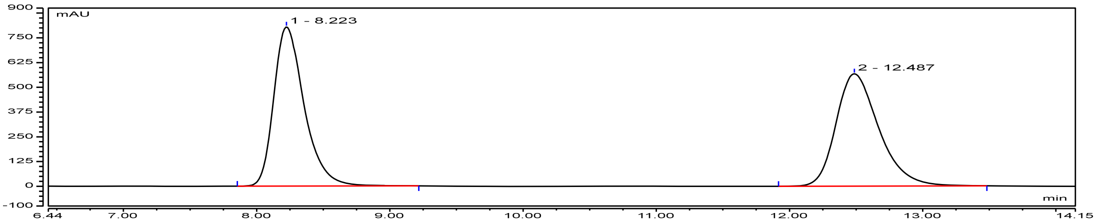


| Entry | Retention | Area | Height | %Area |
|---|---|---|---|---|
| 1 | 8.223 | 212.8346 | 803.78 | 50.12 |
| 2 | 12.487 | 211.8065 | 567.51 | 49.88 |

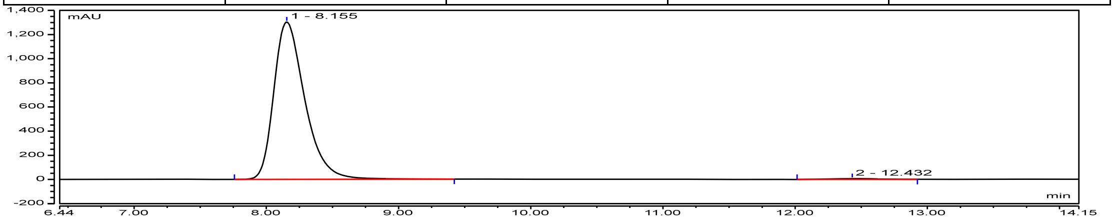

| Entry | Retention | Area | Height | %Area |
|---|---|---|---|---|
| 1 | 8.155 | 353.9295 | 1303.39 | 99.37 |
| 2 | 12.432 | 2.2323 | 6.47 | 0.63 |

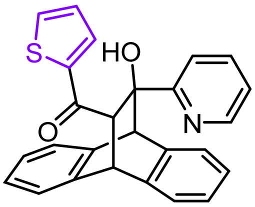


**7e:** Pale yellow solid; Mp 166.7−168.1 °C; 38.8 mg, 95% yield, 96% ee, >20:1 dr; $[\alpha]_D^{22}$ +72.7 (*c* 2.0, $CHCl_3$); $^1$H NMR (300 MHz, Chloroform-*d*) δ 8.40 (d, *J* = 4.8 Hz, 1H), 7.68 (d, *J* = 7.3 Hz, 1H), 7.61 (t, *J* = 7.8 Hz, 1H), 7.45 – 7.34 (m, 3H), 7.30 (d, *J* = 7.5 Hz, 1H), 7.25 – 7.16 (m, 3H), 7.05 (t, *J* = 7.5 Hz, 1H), 6.91 (d, *J* = 7.4 Hz, 1H), 6.75 – 6.67 (m, 3H), 4.96 (s, 1H), 4.75 (s, 1H), 4.48 (s, 1H), 4.17 (s, 1H); $^{13}$C NMR (75 MHz, Chloroform-*d*) δ 191.2, 162.1, 146.8, 145.5, 143.4, 141.2, 140.6, 140.1, 136.8, 133.1, 131.3, 127.4, 126.7, 126.6, 126.3, 126.1, 125.9, 125.9, 125.8, 123.1, 122.5, 120.7, 79.7, 59.8, 58.3, 47.2; HRMS (ESI) m/z 410.1211 (M + H)$^+$, calc. for $C_{26}H_{20}NO_2S$ 410.1209.

The ee was determined by HPLC analysis: CHIRALPAK INB (4.6 mm i.d. x 250 mm); hexane/2–propanol = 70/30; flow rate 1.0 mL/min; 25 °C; 210 nm; retention time: 8.3 min (major) and 12.1 min (minor).

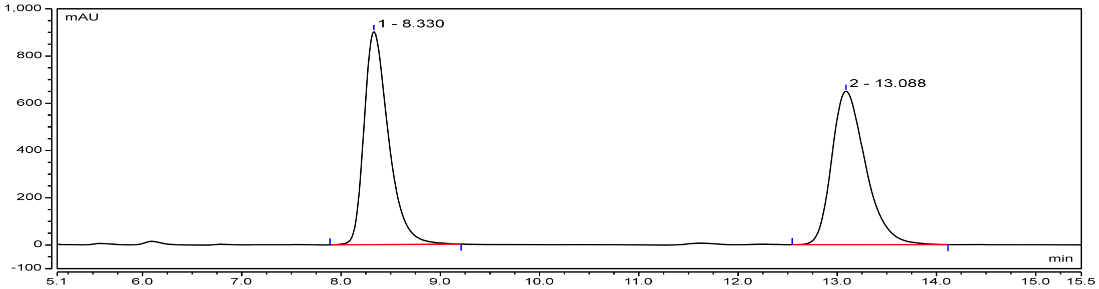


| Entry | Retention | Area | Height | %Area |
|---|---|---|---|---|
| 1 | 8.330 | 258.0053 | 901.82 | 50.15 |
| 2 | 13.088 | 256.5039 | 648.82 | 49.85 |

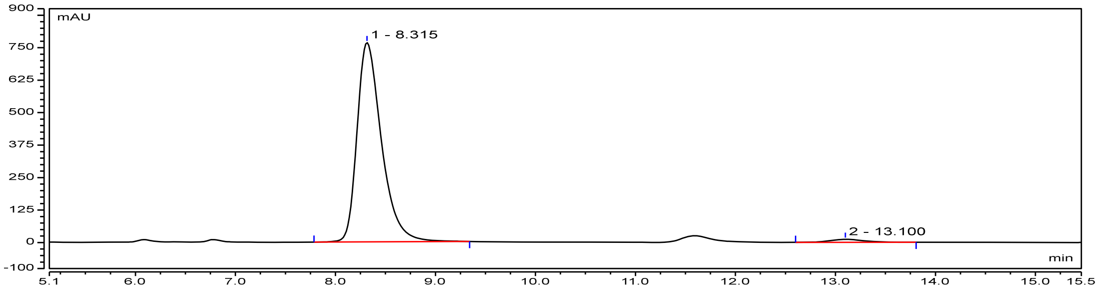


| Entry | Retention | Area | Height | %Area |
|---|---|---|---|---|
| 1 | 8.315 | 217.7783 | 767.10 | 98.00 |
| 2 | 13.100 | 4.4365 | 11.43 | 2.00 |

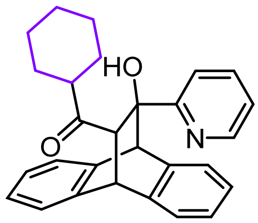


**7f:** White solid; Mp 90.7−92.2 °C; 36.4 mg, 89% yield, 97% ee, >20:1 dr; $[\alpha]_D^{22}$ +27.8 (*c* 2.0, $CHCl_3$); $^1H$ NMR (300 MHz, Methylene Chloride-*d*$_2$) δ 8.50 (d, *J* = 4.8 Hz, 1H), 7.60 – 7.50 (m, 2H), 7.36 (d, *J* = 7.4 Hz, 1H), 7.29 – 7.11 (m, 5H), 7.04 (t, *J* = 7.5 Hz, 1H), 6.90 (d, *J* = 7.4 Hz, 1H), 6.39 (d, *J* = 8.1 Hz, 1H), 5.54 (s, 1H), 4.62 (s, 1H), 4.04 (s, 1H), 3.71 (s, 1H), 1.78 (t, *J* = 11.6 Hz, 1H), 1.52 (q, *J* = 17.3, 15.6 Hz, 5H), 1.34 – 1.12 (m, 2H), 1.03 – 0.91 (m, 2H), 0.74 (q, *J* = 12.5 Hz, 1H); $^{13}C$ NMR (75 MHz, Methylene Chloride-*d*$_2$) δ 211.2, 162.6, 147.3, 144.7, 141.8, 141.8, 141.0, 137.2, 126.9, 126.8, 126.5, 126.2, 126.1, 126.0, 125.9, 123.4, 123.0, 120.8, 79.4, 60.7, 60.0, 51.5, 46.4, 30.1, 27.0, 26.5, 26.2, 25.50; HRMS (ESI) m/z 410.1212 $(M + H)^+$, calc. for $C_{28}H_{28}NO_2$ 410.2115.

The ee was determined by HPLC analysis: CHIRALPAK Cellulose-4 (4.6 mm i.d. x 250 mm); hexane/2–propanol = 70/30; flow rate 1.0 mL/min; 25 °C; 210 nm; retention time: 5.7 min (minor) and 8.3 min (major).

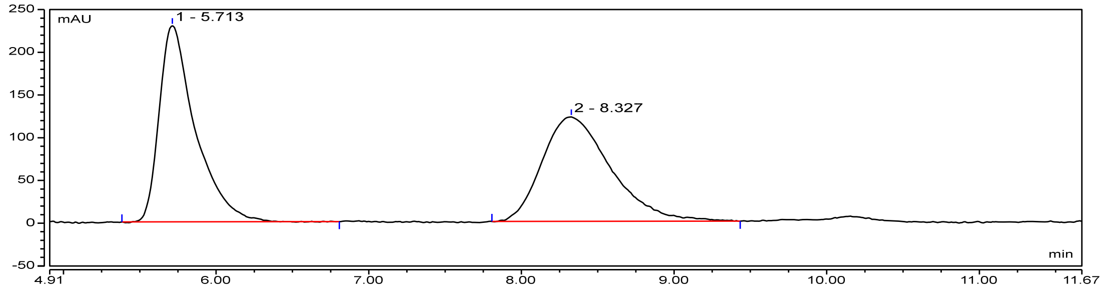


| Entry | Retention | Area | Height | %Area |
|---|---|---|---|---|
| 1 | 5.713 | 62.6353 | 229.50 | 49.62 |
| 2 | 8.327 | 63.5871 | 122.19 | 50.38 |

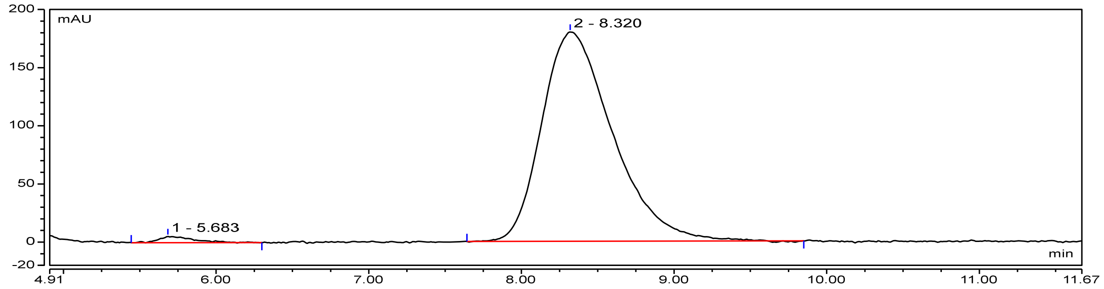


| Entry | Retention | Area | Height | %Area |
|---|---|---|---|---|
| 1 | 5.683 | 1.4213 | 5.28 | 1.45 |
| 2 | 8.320 | 96.6924 | 179.91 | 98.55 |

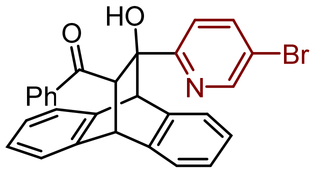


**7g:** White solid; Mp 159.1−161.2 °C; 47.2 mg, 98% yield, 97% ee, >20:1 dr; $[\alpha]_D^{22}$ +104.9 (*c* 2.0, $CHCl_3$); $^1H$ NMR (300 MHz, Chloroform-*d*) δ 8.38 (d, *J* = 2.1 Hz, 1H), 7.74 (dd, *J* = 8.6, 2.2 Hz,

1H), 7.64 (d, *J* = 7.3 Hz, 1H), 7.48 – 7.35 (m, 5H), 7.30 (t, *J* = 7.4 Hz, 1H), 7.26 – 7.13 (m, 4H), 7.06 (t, *J* = 7.5 Hz, 1H), 6.92 – 6.86 (m, 2H), 4.75 (d, *J* = 7.6 Hz, 2H), 4.42 (s, 1H), 4.16 (s, 1H); $^{13}$C NMR (75 MHz, Chloroform-*d*) δ 199.5, 161.2, 147.9, 143.3, 141.1, 140.3, 139.8, 139.2, 138.0, 132.5, 128.1, 128.0, 126.7, 126.6, 126.5, 126.02, 125.9, 123.1, 122.1, 119.4, 79.8, 60.0, 55.9, 47.3; HRMS (ESI) m/z 482.0754 (M + H)$^+$, calc. for $C_{28}H_{21}BrNO_2$ 482.0750.

The ee was determined by HPLC analysis: CHIRALPAK Amylose-1 (4.6 mm i.d. x 250 mm); hexane/2–propanol = 70/30; flow rate 1.0 mL/min; 25 °C; 210 nm; retention time: 8.4 min (major) and 14.3 min (minor).

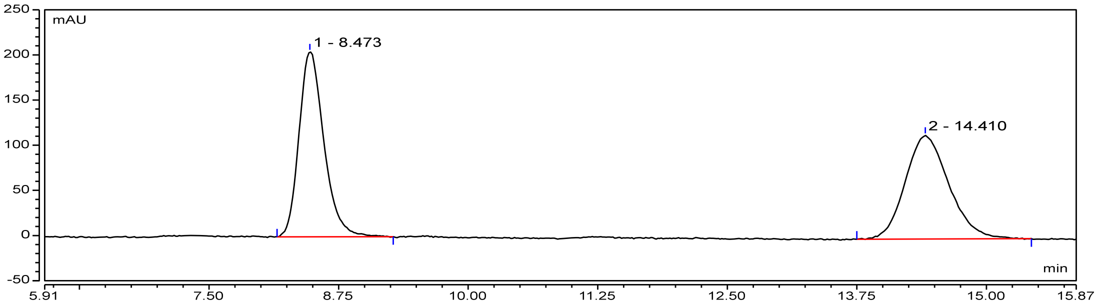


| Entry | Retention | Area | Height | %Area |
|---|---|---|---|---|
| 1 | 8.473 | 57.0831 | 204.81 | 50.52 |
| 2 | 14.410 | 55.9181 | 114.82 | 49.48 |

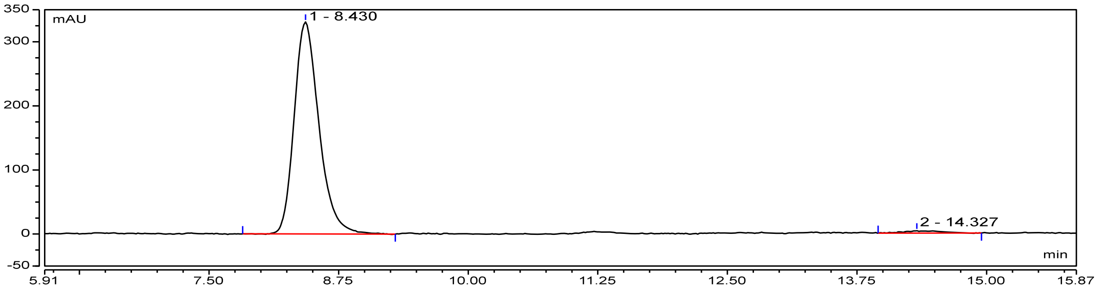


| Entry | Retention | Area | Height | %Area |
|---|---|---|---|---|
| 1 | 8.427 | 29.0229 | 105.73 | 98.41 |
| 2 | 14.383 | 0.4687 | 1.07 | 1.59 |

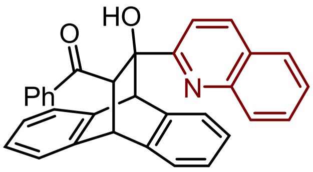


**7h:** White solid; Mp 98.5−100.4 °C; 36.7 mg, 81% yield, 90% ee, >20:1 dr; [α]$^{22}_{D}$ +58.8 (*c* 2.0, $CHCl_3$); $^1$H NMR (300 MHz, Methylene Chloride-$d_2$) δ 8.07 (d, *J* = 8.7 Hz, 1H), 7.85 (t, *J* = 8.8 Hz, 2H), 7.70 (t, *J* = 8.3 Hz, 2H), 7.58 (t, *J* = 7.6 Hz, 1H), 7.48 (d, *J* = 7.4 Hz, 1H), 7.35 – 7.24 (m, 3H), 7.21 – 7.17 (m, 4H), 7.10 (t, *J* = 7.5 Hz, 1H), 6.96 – 6.86 (m, 3H), 6.47 (d, *J* = 8.7 Hz, 1H), 5.73 (s, 1H), 4.85 (s, 1H), 4.52 (s, 1H), 4.17 (s, 1H); $^{13}$C NMR (75 MHz, Methylene Chloride-$d_2$) δ 198.7, 162.5, 145.8, 144.5, 142.2, 141.8, 141.4, 138.8, 137.5, 132.3, 130.4, 128.9,

128.2, 128.0, 127.8, 127.6, 127.3, 127.2, 127.1, 126.7, 126.4, 126.2, 126.1, 126.0, 123.7, 118.5, 79.9, 59.8, 58.2, 47.1; HRMS (ESI) m/z 454.1803 $(M + H)^+$, calc. for $C_{32}H_{24}NO_2$ 454.1802.

The ee was determined by HPLC analysis: CHIRALPAK INB (4.6 mm i.d. x 250 mm); hexane/2–propanol = 70/30; flow rate 1.0 mL/min; 25 °C; 210 nm; retention time: 7.0 min (major) and 10.8 min (minor).

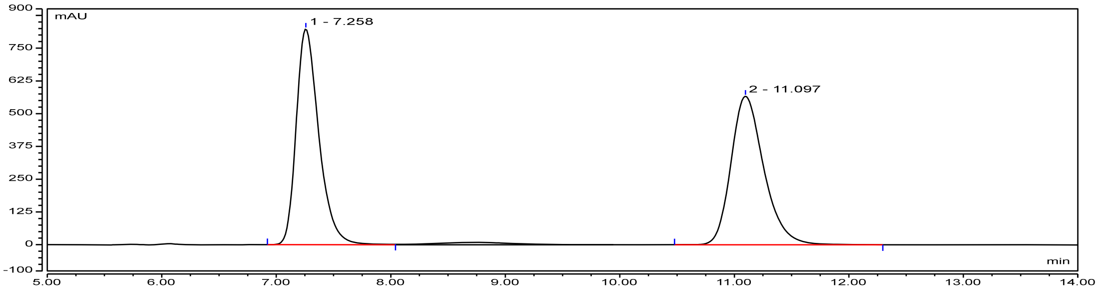


| Entry | Retention | Area | Height | %Area |
|---|---|---|---|---|
| 1 | 7.258 | 188.5295 | 823.28 | 50.01 |
| 2 | 11.097 | 188.4801 | 566.67 | 49.99 |

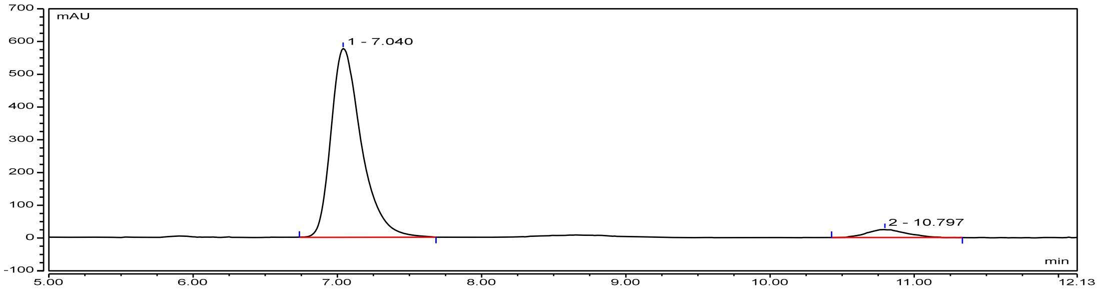


| Entry | Retention | Area | Height | %Area |
|---|---|---|---|---|
| 1 | 7.040 | 140.7404 | 576.23 | 94.83 |
| 2 | 10.797 | 7.6753 | 24.21 | 5.17 |

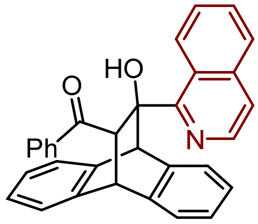


**7i:** White solid; Mp 146.5−148.4 °C; 44.8 mg, 99% yield, 91% ee, >20:1 dr; $[\alpha]^{22}_{D}$ +95.7 (*c* 2.0, $CHCl_3$); $^1H$ NMR (300 MHz, Methylene Chloride-$d_2$) δ 9.11 (s, 1H), 8.17 (d, *J* = 5.6 Hz, 1H), 7.97 (d, *J* = 7.7 Hz, 2H), 7.86 (d, *J* = 8.1 Hz, 1H), 7.70 (t, *J* = 7.4 Hz, 1H), 7.66 – 7.58 (m, 2H), 7.54 – 7.44 (m, 3H), 7.39 – 7.27 (m, 5H), 7.09 (t, *J* = 7.5 Hz, 1H), 6.80 (t, *J* = 7.5 Hz, 1H), 6.51 (d, *J* = 7.4 Hz, 1H), 6.32 (s, 1H), 5.09 (s, 1H), 4.65 (s, 1H), 4.37 (s, 1H); $^{13}C$ NMR (75 MHz, Methylene Chloride-$d_2$) δ 203.0, 160.0, 143.0, 142.0, 141.0, 139.3, 138.5, 138.3, 133.4, 130.0, 129.1, 128.8, 128.5, 128.1, 127.1, 127.1, 126.9, 126.7, 126.7, 126.5, 126.5, 126.1, 125.5, 123.3, 121.6, 85.3, 59.1, 51.7, 48.6; HRMS (ESI) m/z 454.1804 $(M + H)^+$, calc. for $C_{32}H_{24}NO_2$ 454.1802.

The ee was determined by HPLC analysis: CHIRALPAK Amylose-1 (4.6 mm i.d. x 250 mm);

hexane/2–propanol = 70/30; flow rate 1.0 mL/min; 25 °C; 210 nm; retention time: 9.2 min (minor) and 21.5 min (major).

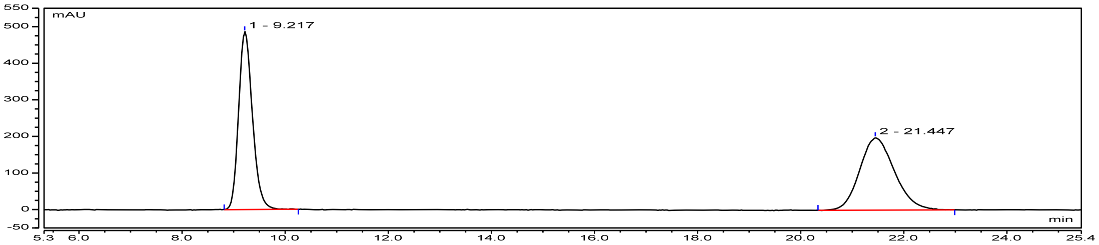


| Entry | Retention | Area | Height | %Area |
|---|---|---|---|---|
| 1 | 9.217 | 151.3787 | 486.13 | 49.54 |
| 2 | 21.447 | 154.2149 | 198.28 | 50.46 |

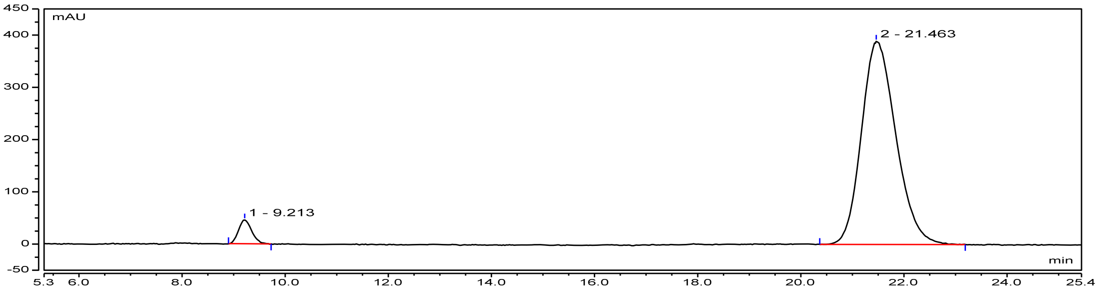


| Entry | Retention | Area | Height | %Area |
|---|---|---|---|---|
| 1 | 9.213 | 13.6150 | 45.74 | 4.32 |
| 2 | 21.463 | 301.6341 | 389.16 | 95.68 |

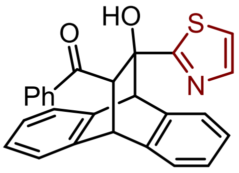


**7j:** Pale yellow oil; 40.1 mg, 98% yield, 85% ee, >20:1 dr; $[\alpha]_D^{22}$ +47.9 (*c* 2.0, $CHCl_3$); $^1$H NMR (300 MHz, Chloroform-*d*) δ 7.71 (d, *J* = 7.7 Hz, 2H), 7.54 – 7.49 (m, 2H), 7.45 (dd, *J* = 7.1, 4.3 Hz, 2H), 7.39 (d, *J* = 7.3 Hz, 1H), 7.34 – 7.26 (m, 4H), 7.26 – 7.17 (m, 2H), 7.08 (t, *J* = 7.4 Hz, 1H), 7.01 (d, *J* = 7.3 Hz, 1H), 5.05 (s, 1H), 4.64 (s, 1H), 4.38 (s, 1H), 3.92 (s, 1H); $^{13}$C NMR (75 MHz, Chloroform-*d*) δ 200.3, 175.4, 142.7, 141.4, 140.7, 139.6, 139.0, 137.4, 133.2, 128.4, 127.0, 126.8, 126.5, 126.4, 126.3, 126.2, 126.0, 123.0, 120.0, 80.1, 60.7, 55.6, 48.0; HRMS (ESI) m/z 410.2117 (M + H)$^+$, calc. for $C_{26}H_{20}NO_2S$ 410.1209.

The ee was determined by HPLC analysis: CHIRALPAK INB (4.6 mm i.d. x 250 mm); hexane/2–propanol = 70/30; flow rate 1.0 mL/min; 25 °C; 210 nm; retention time: 6.4 min (major) and 17.9 min (minor).

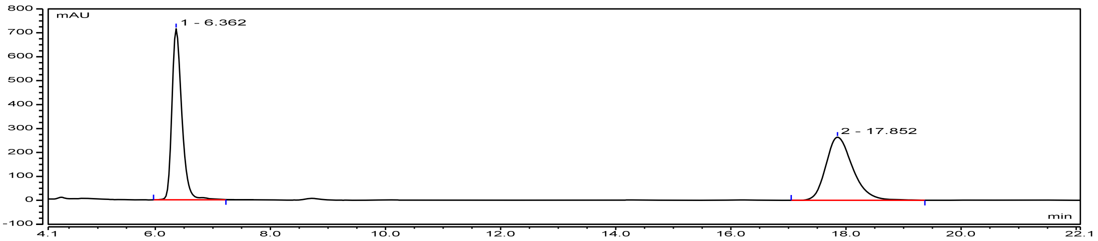

| Entry | Retention | Area | Height | %Area |
|---|---|---|---|---|
| 1 | 6.362 | 143.1095 | 714.83 | 50.14 |
| 2 | 17.852 | 142.3092 | 263.26 | 49.86 |

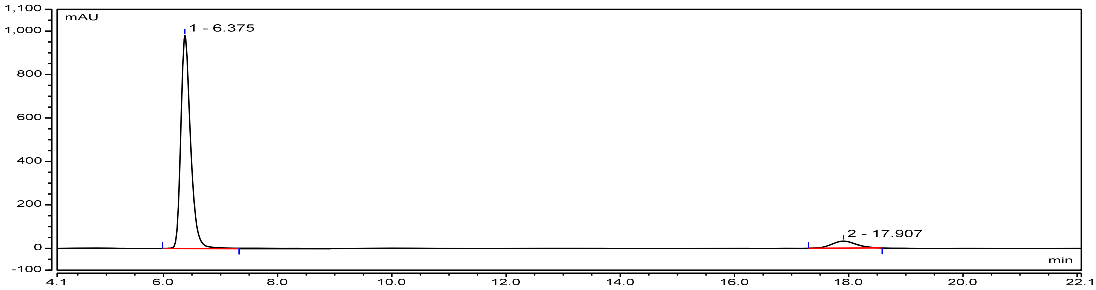


| Entry | Retention | Area | Height | %Area |
|---|---|---|---|---|
| 1 | 6.375 | 195.1305 | 981.13 | 92.33 |
| 2 | 17.907 | 16.2112 | 32.26 | 7.67 |

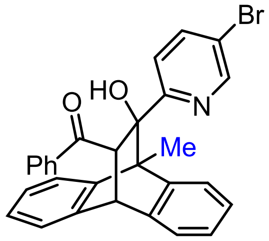


**7k:** White solid; Mp 103.5−105.2 °C; 49.1 mg, 99% yield, 92% ee, >20:1 dr; $[\alpha]^{22}_{D}$ +74.7 (*c* 2.0, $CHCl_3$); $^1$H NMR (300 MHz, Methylene Chloride-$d_2$) δ 8.37 (s, 1H), 7.71 (t, *J* = 6.1 Hz, 2H), 7.46 (d, *J* = 7.3 Hz, 1H), 7.36 – 7.26 (m, 4H), 7.21 (t, *J* = 7.6 Hz, 2H), 7.15 – 7.0 (m, 5H), 6.19 (d, *J* = 8.5 Hz, 1H), 5.04 (s, 1H), 4.79 (s, 1H), 4.31 (s, 1H), 1.39 (s, 3H); $^{13}$C NMR (75 MHz, Methylene Chloride-$d_2$) δ 198.7, 160.4, 147.8, 145.1, 143.7, 143.3, 142.5, 139.7, 138.9, 132.3, 128.2, 127.9, 127.0, 126.3, 125.9, 125.8, 124.4, 123.3, 123.3, 122.4, 119.9, 80.9, 60.8, 53.6, 46.4, 11.9; HRMS (ESI) m/z 496.0908 $(M + H)^+$, calc. for $C_{29}H_{23}BrNO_2$ 496.0907.

The ee was determined by HPLC analysis: CHIRALPAK INB (4.6 mm i.d. x 250 mm); hexane/2–propanol = 70/30; flow rate 1.0 mL/min; 25 °C; 210 nm; retention time: 7.0 min (major) and 18.6 min (minor).

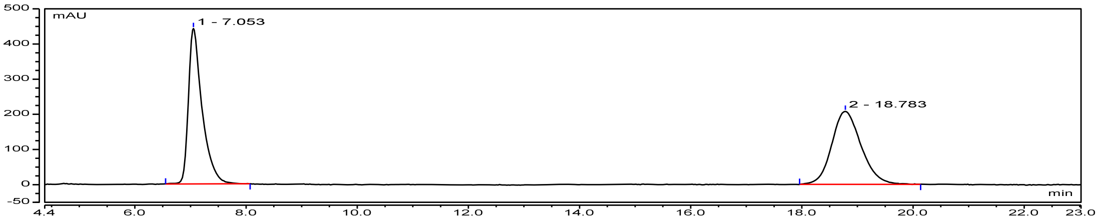


| Entry | Retention | Area | Height | %Area |
|---|---|---|---|---|
| 1 | 7.053 | 126.6105 | 441.93 | 49.87 |
| 2 | 18.783 | 127.2620 | 207.81 | 50.13 |

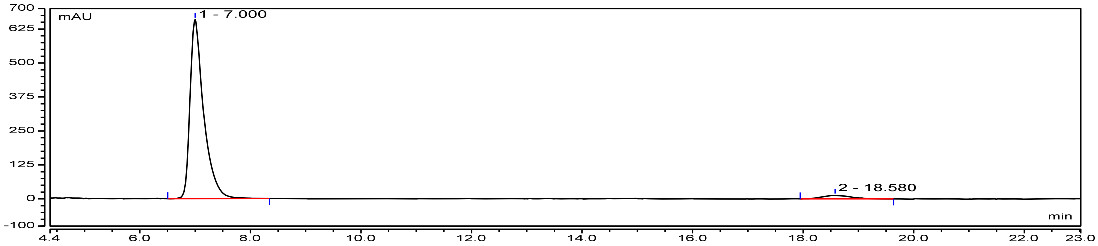

| Entry | Retention | Area | Height | %Area |
|---|---|---|---|---|
| 1 | 7.000 | 189.6402 | 659.61 | 96.07 |
| 2 | 18.580 | 7.7549 | 13.46 | 3.93 |

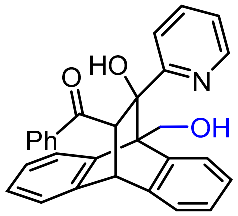

**7l:** Yellow solid; Mp 135.8−137.4 °C; 37.6 mg, 87% yield, 96% ee, >20:1 dr; $[\alpha]_D^{22}$ +37.1 (*c* 2.0, $CHCl_3$); $^1$H NMR (300 MHz, Methylene Chloride-$d_2$) δ 8.27 (s, 1H), 7.71 (t, $J$ = 7.2 Hz, 2H), 7.58 (t, $J$ = 8.0 Hz, 1H), 7.46 (d, $J$ = 7.4 Hz, 1H), 7.31 – 7.26 (m, 6H), 7.13 – 6.91 (m, 5H), 6.34 (d, $J$ = 8.1 Hz, 1H), 5.78 (s, 1H), 4.76 (s, 1H), 4.34 (d, $J$ = 11.1 Hz, 1H), 4.27 (s, 1H), 4.17 (d, $J$ = 11.4 Hz, 1H), 2.83 (s, 1H); $^{13}$C NMR (75 MHz, Methylene Chloride-$d_2$) δ 198.7, 161.1, 146.9, 145.5, 143.1, 141.3, 141.0, 138.8, 137.5, 132.4, 128.2, 127.9, 127.2, 127.2, 126.3, 126.0, 125.8, 125.2, 124.7, 123.5, 123.1, 121.3, 81.8, 61.9, 61.8, 56.4, 46.72; HRMS (ESI) m/z 434.1752 $(M + H)^+$, calc. for $C_{29}H_{24}NO_3$ 434.1751.

The ee was determined by HPLC analysis: CHIRALPAK Amylose-1 (4.6 mm i.d. x 250 mm); hexane/2–propanol = 30/70; flow rate 1.0 mL/min; 25 °C; 210 nm; retention time: 6.7 min (minor) and 11.4 min (major).

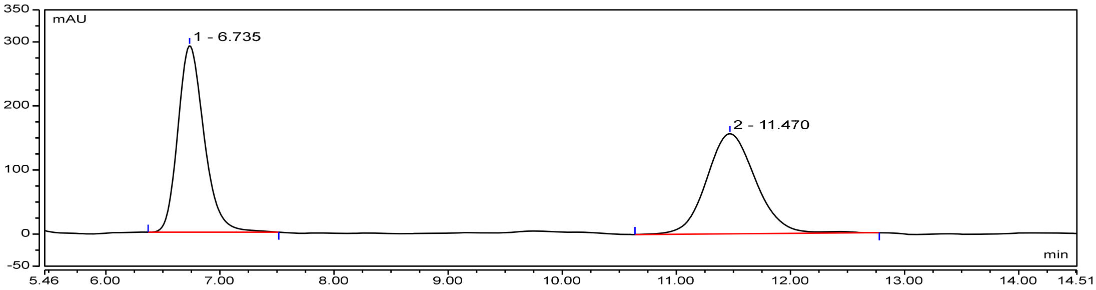

| Entry | Retention | Area | Height | %Area |
|---|---|---|---|---|
| 1 | 6.735 | 77.6154 | 290.99 | 49.89 |
| 2 | 11.470 | 77.9677 | 156.09 | 50.11 |

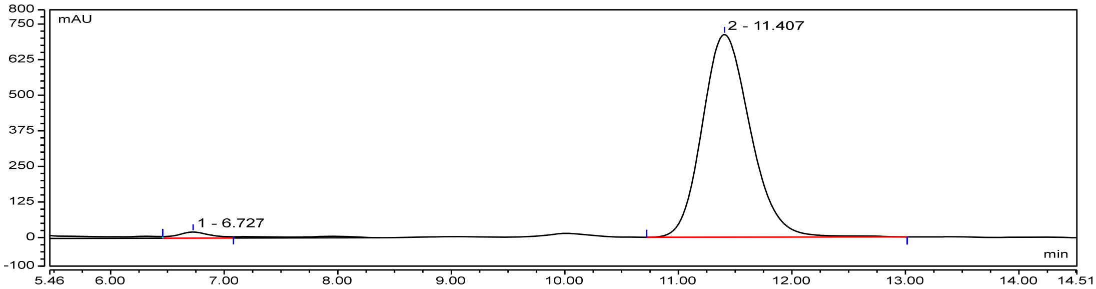

| Entry | Retention | Area | Height | %Area |
| --- | --- | --- | --- | --- |
| 1 | 6.727 | 7.2823 | 21.39 | 2.04 |
| 2 | 11.407 | 349.8162 | 711.24 | 97.96 |

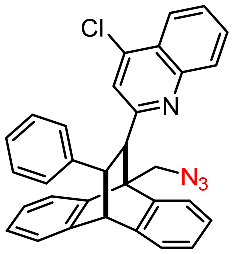


**8:** Pale yellow oil; 38.0 mg, 76% yield, 92% ee, >20:1 dr; $[\alpha]_D^{22}$ +167.0 (*c* 2.0, $CHCl_3$); $^1$H NMR (300 MHz, Chloroform-*d*) δ 7.94 (t, *J* = 9.2 Hz, 2H), 7.87 (d, *J* = 7.6 Hz, 1H), 7.69 (t, *J* = 7.8 Hz, 1H), 7.57 (d, *J* = 7.4 Hz, 1H), 7.50 (d, *J* = 7.8 Hz, 1H), 7.37 – 7.30 (m, 3H), 7.19 – 7.11 (m, 5H), 6.84 (d, *J* = 7.7 Hz, 1H), 6.72 (s, 2H), 5.68 (s, 1H), 4.69 (d, *J* = 12.5 Hz, 1H), 4.44 (s, 1H), 4.04 (d, *J* = 5.7 Hz, 1H), 3.79 (d, *J* = 12.4 Hz, 1H), 3.56 (d, *J* = 5.6 Hz, 1H); $^{13}$C NMR (75 MHz, Chloroform-*d*) δ 162.6, 146.4, 144.8, 143.1, 142.7, 141.0, 140.0, 130.7, 129.8, 128.5, 128.1, 128.0, 126.7, 126.4, 126.2, 125.9, 125.7, 123.5, 123.0, 123.0, 121.9, 120.4, 120.0, 106.4, 60.7, 56.1, 53.4, 52.1; HRMS (ESI) m/z 499.1686 (M + H)$^+$, calc. for $C_{32}H_{24}ClN_4$ 499.1684.

The ee was determined by HPLC analysis: CHIRALPAK Amylose-1 (4.6 mm i.d. x 250 mm); hexane/2–propanol = 90/10; flow rate 1.0 mL/min; 25 °C; 210 nm; retention time: 9.3 min (minor) and 12.6 min (major).

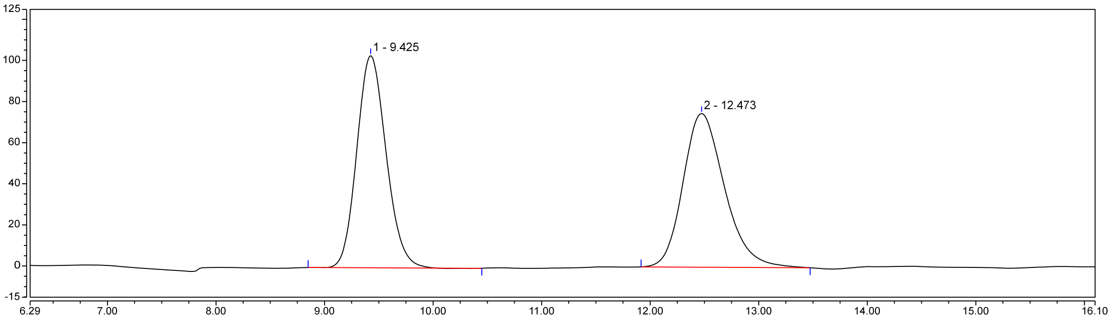


| Entry | Retention | Area | Height | %Area |
| --- | --- | --- | --- | --- |
| 1 | 9.425 | 32.2628 | 103.12 | 49.36 |
| 2 | 12.473 | 33.1042 | 74.72 | 50.64 |

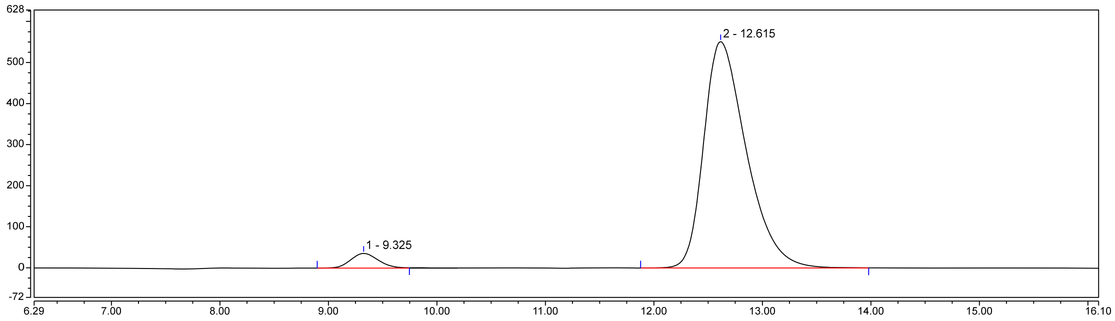


| Entry | Retention | Area | Height | %Area |
| --- | --- | --- | --- | --- |
| 1 | 9.325 | 10.5652 | 35.77 | 4.04 |
| 2 | 12.615 | 250.8381 | 551.09 | 95.96 |

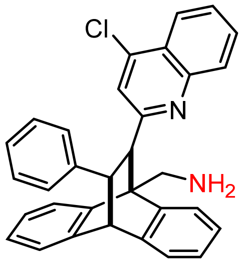


**9:** Pale yellow solid; Mp 158.2−160.1 °C; 41.6 mg, 88% yield, 92% ee, >20:1 dr; $[\alpha]^{22}_{D}$ +60.3 (*c* 2.0, $CHCl_3$); $^1$H NMR (300 MHz, Chloroform-*d*) δ 7.83 (t, *J* = 7.7 Hz, 2H), 7.61 (t, *J* = 6.9 Hz, 2H), 7.49 (d, *J* = 7.3 Hz, 1H), 7.39 – 7.31 (m, 2H), 7.22 (d, *J* = 7.4 Hz, 1H), 7.16 –7.04 (m, 6H), 6.84 (d, *J* = 7.5 Hz, 1H), 6.72 (s, 2H), 5.35 (s, 1H), 4.65 (d, *J* = 12.4 Hz, 1H), 4.51 (s, 2H), 4.37 (s, 1H), 3.92 – 3.81 (m, 2H), 3.53 (d, *J* = 6.2 Hz, 1H); $^{13}$C NMR (75 MHz, Chloroform-*d*) δ 162.2, 150.2, 147.3, 145.4, 144.2, 143.4, 141.2, 140.6, 129.8, 128.9, 128.1, 128.0, 126.6, 126.3, 126.1, 125.7, 125.5, 124.8, 123.2, 123.0, 120.1, 117.6, 101.3, 60.8, 56.4, 52.8, 52.2, 52.1; HRMS (ESI) m/z 473.1782 (M + H)$^+$, calc. for $C_{32}H_{26}ClN_2$ 473.1779.

The ee was determined by HPLC analysis: CHIRALPAK Amylose-1 (4.6 mm i.d. x 250 mm); hexane/2–propanol = 80/20; flow rate 1.0 mL/min; 25 °C; 210 nm; retention time: 9.7 min (minor) and 12.2 min (major).

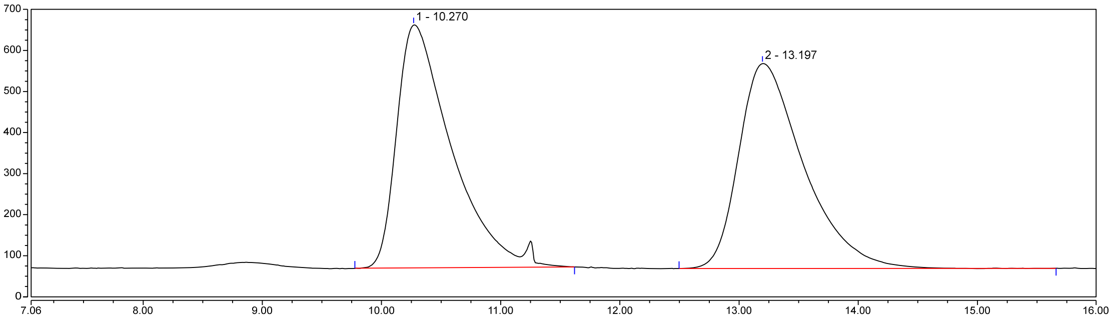


| Entry | Retention | Area | Height | %Area |
|---|---|---|---|---|
| 1 | 10.270 | 315.7968 | 591.88 | 50.08 |
| 2 | 13.197 | 314.7336 | 499.45 | 49.92 |

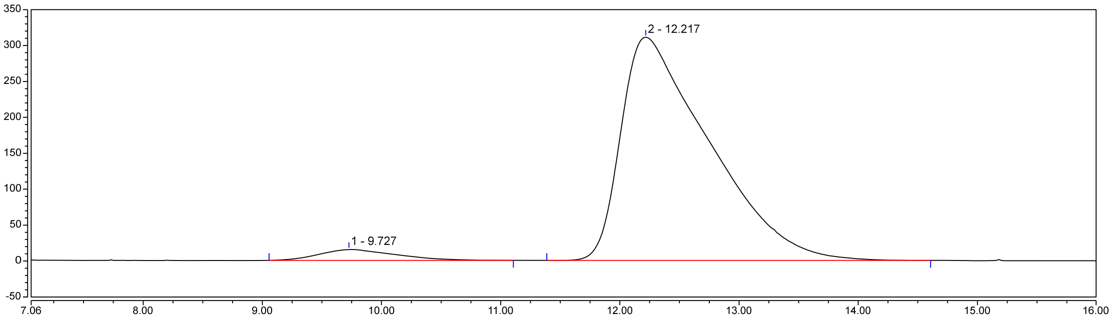


| Entry | Retention | Area | Height | %Area |
|---|---|---|---|---|
| 1 | 9.727 | 11.1263 | 15.01 | 4.01 |
| 2 | 12.217 | 266.5885 | 310.67 | 95.99 |

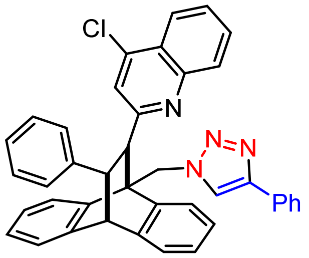


**11:** Pale yellow oil; 40.8 mg, 68% yield, 92% ee, >20:1 dr; $[\alpha]_D^{22}$ +20.2 (*c* 2.0, $CHCl_3$); $^1H$ NMR (300 MHz, Chloroform-*d*) δ 8.22 (t, *J* = 7.7 Hz, 1H), 8.10 (t, *J* = 9.1 Hz, 2H), 8.00 – 7.75 (m, 4H), 7.73 – 7.67 (m, 1H), 7.64 – 7.44 (m, 6H), 7.36 (t, *J* = 7.0 Hz, 2H), 7.16 – 7.04 (m, 4H), 6.90 (d, *J* = 7.3 Hz, 1H), 6.66 (s, 2H), 6.11 (s, 1H), 4.72 (d, *J* = 12.4 Hz, 1H), 4.43 (s, 1H), 4.14 (s, 1H), 3.84 (d, *J* = 12.5 Hz, 1H), 3.61 (s, 1H); $^{13}C$ NMR (75 MHz, Chloroform-*d*) δ 162.7, 148.1, 145.2, 142.7, 142.4, 141.0, 140.9, 140.4, 131.1, 129.7, 129.1, 128.8, 128.5, 128.3, 128.2, 128.0, 126.9, 126.7, 126.6, 126.3, 125.9, 125.8, 125.7, 124.2, 123.5, 123.2, 123.0, 120.9, 113.6, 60.8, 56.0, 53.3, 52.3, 52.2; HRMS (ESI) m/z 601.2156 $(M + H)^+$, calc. for $C_{40}H_{30}ClN_4$ 601.2154.

The ee was determined by HPLC analysis: CHIRALPAK i-Amylose-1 (4.6 mm i.d. x 250 mm); hexane/2–propanol = 80/20; flow rate 1.0 mL/min; 25 °C; 210 nm; retention time: 11.7 min (minor) and 18.1 min (major).

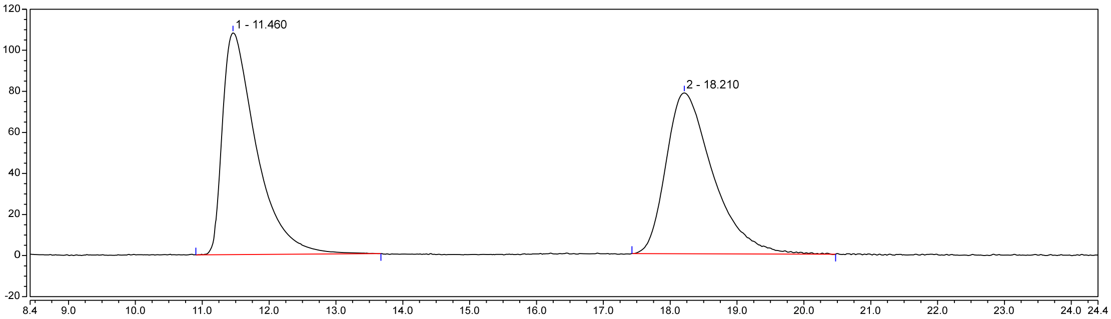


| Entry | Retention | Area | Height | %Area |
|---|---|---|---|---|
| 1 | 11.460 | 63.3814 | 107.97 | 49.87 |
| 2 | 18.210 | 63.7091 | 78.33 | 50.13 |

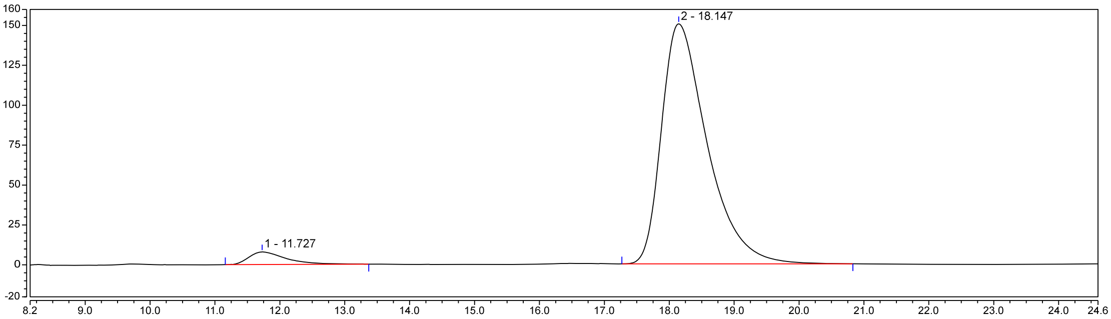


| Entry | Retention | Area | Height | %Area |
|---|---|---|---|---|
| 1 | 11.727 | 5.3005 | 7.95 | 4.15 |
| 2 | 18.147 | 122.5452 | 150.38 | 95.85 |

## 8. Copies of NMR spectra

$^{1}$H NMR (300 MHz, Methylene Chloride-$d_2$) of compound **3a**

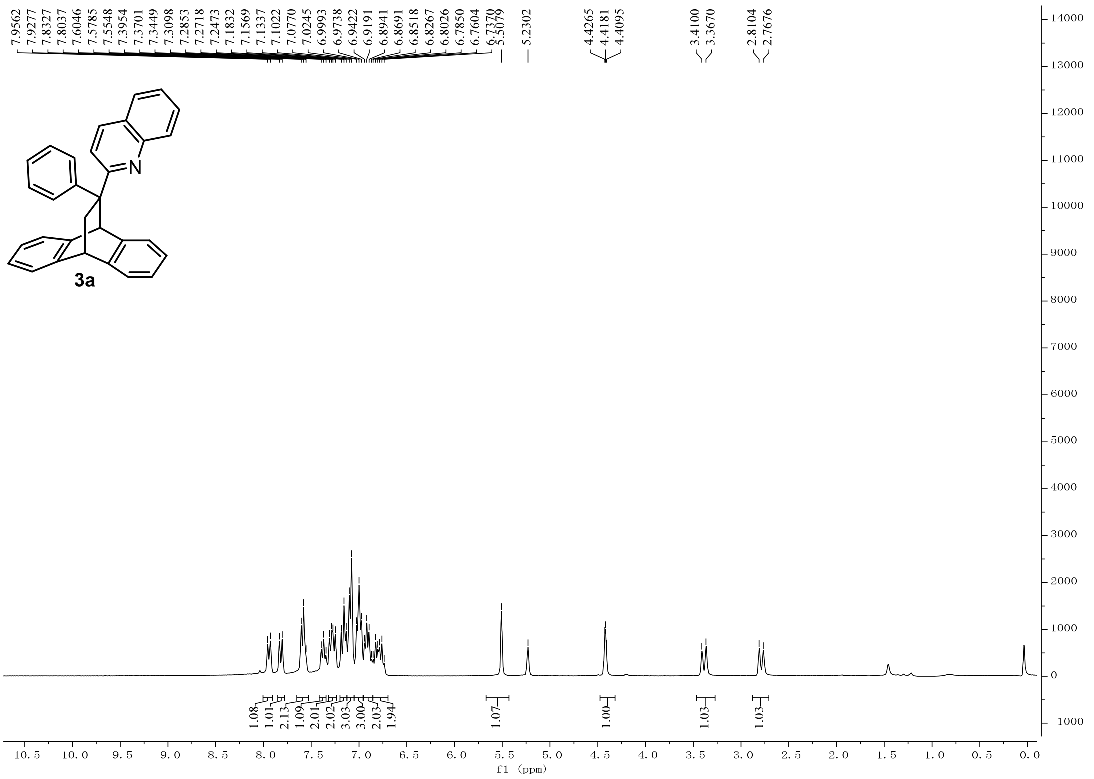


$^{13}$C NMR (75 MHz, Methylene Chloride-$d_2$) of compound **3a**

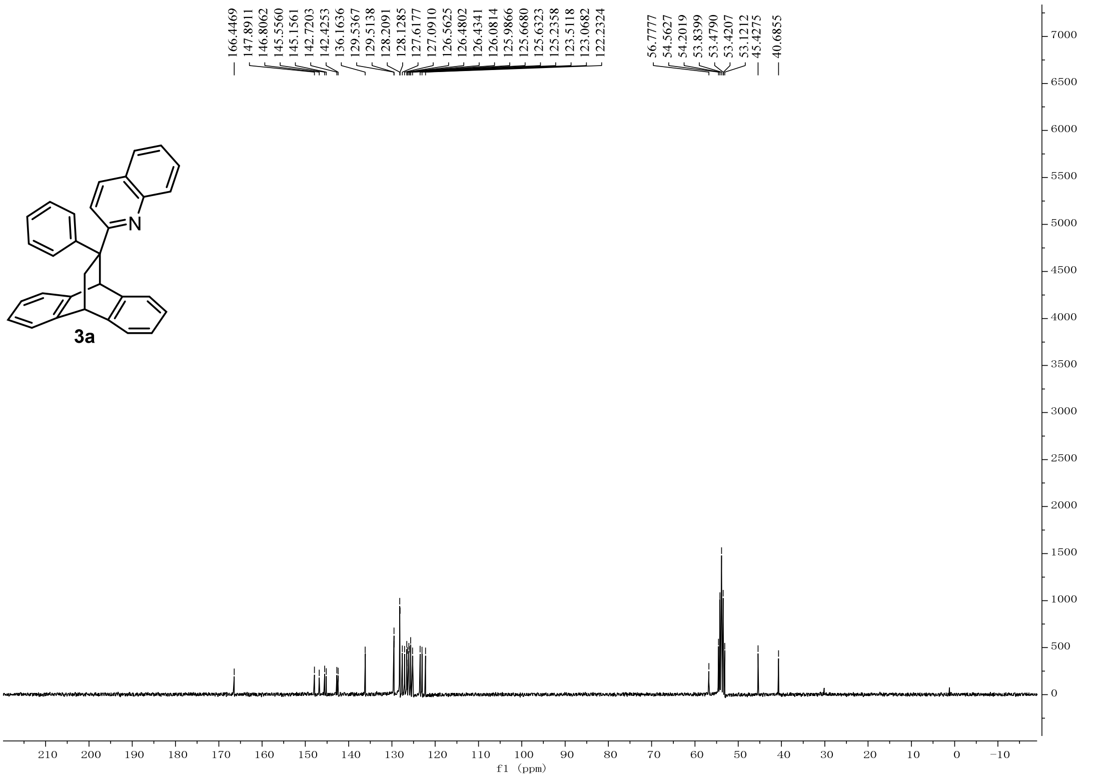

$^{1}H$ NMR (300 MHz, Chloroform-*d*) of compound **3b**

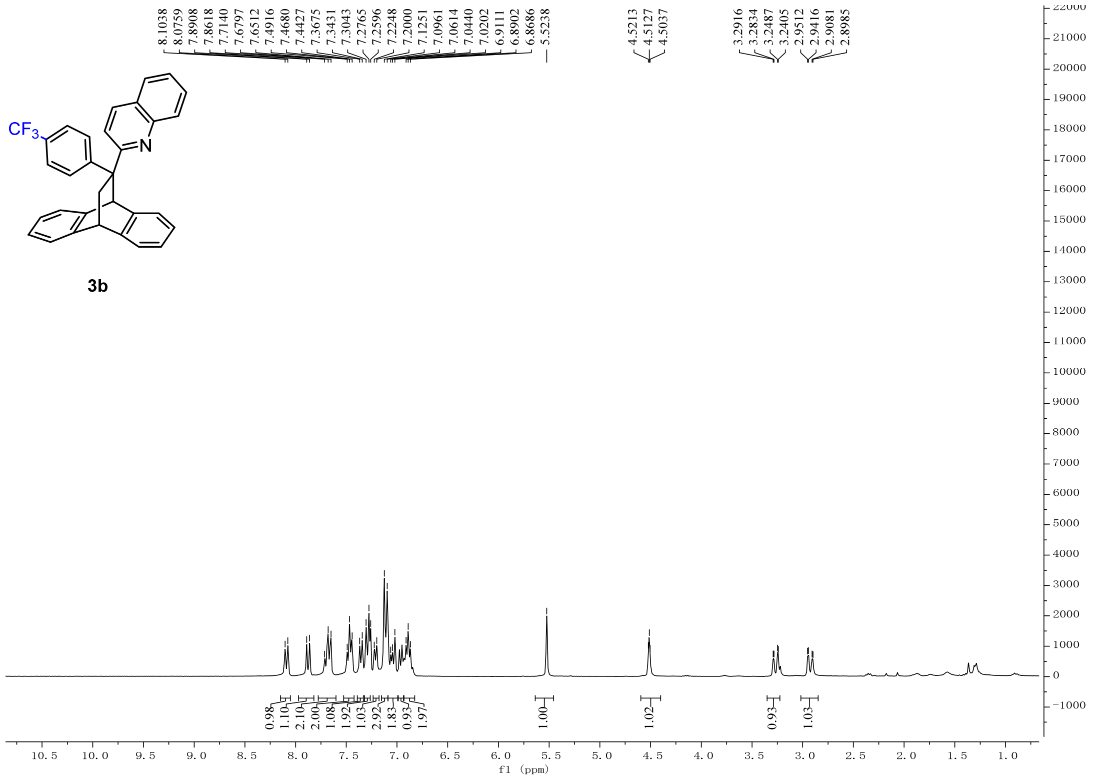


$^{13}C$ NMR (75 MHz, Chloroform-*d*) of compound **3b**

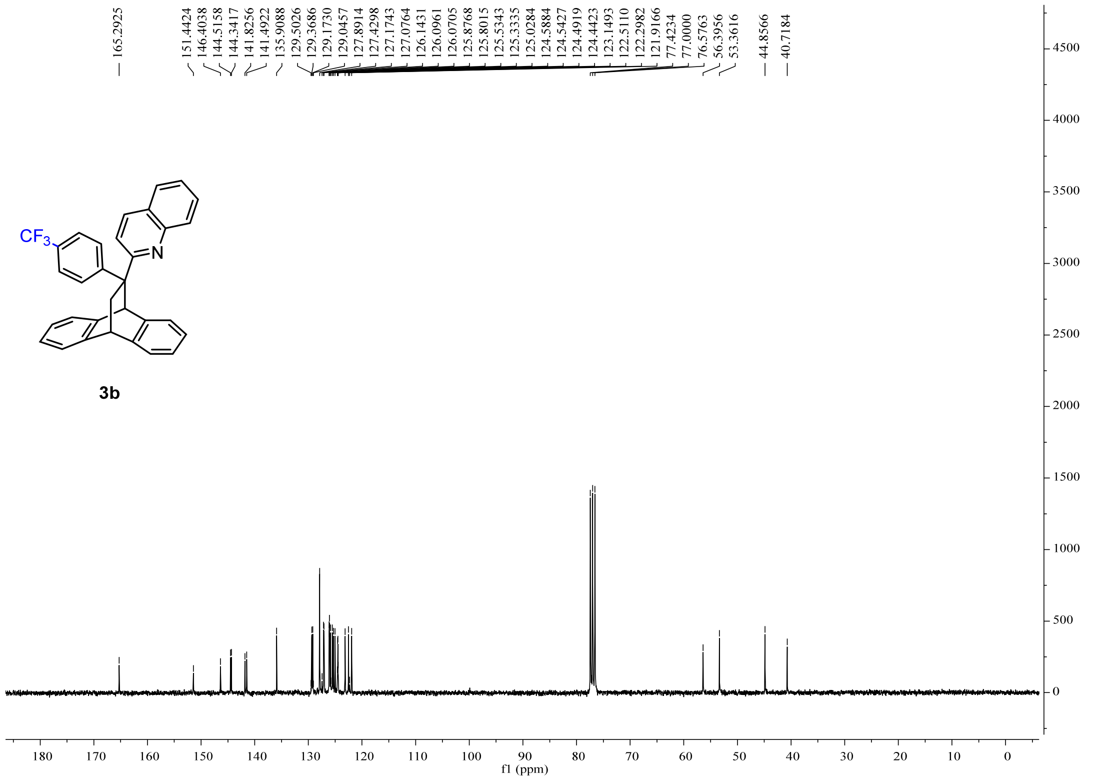

$^{19}F$ NMR (565 MHz, Chloroform-*d*) of compound **3b**

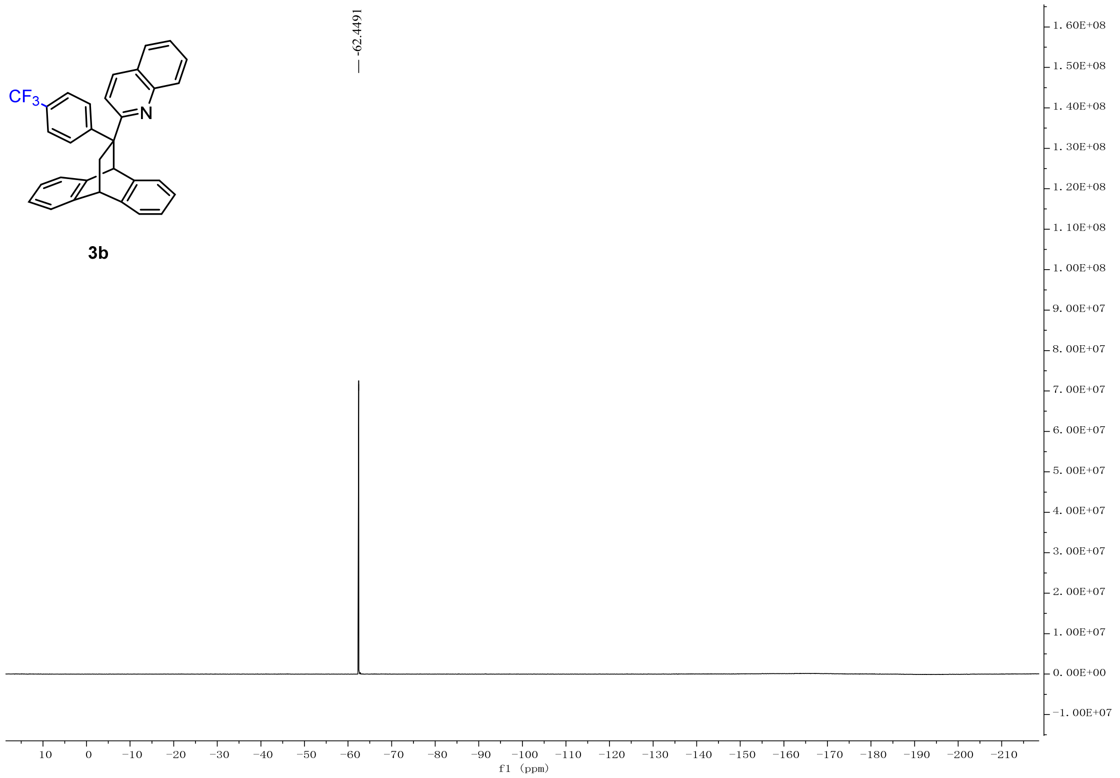


$^{1}H$ NMR (300 MHz, Chloroform-*d*) of compound **3c**

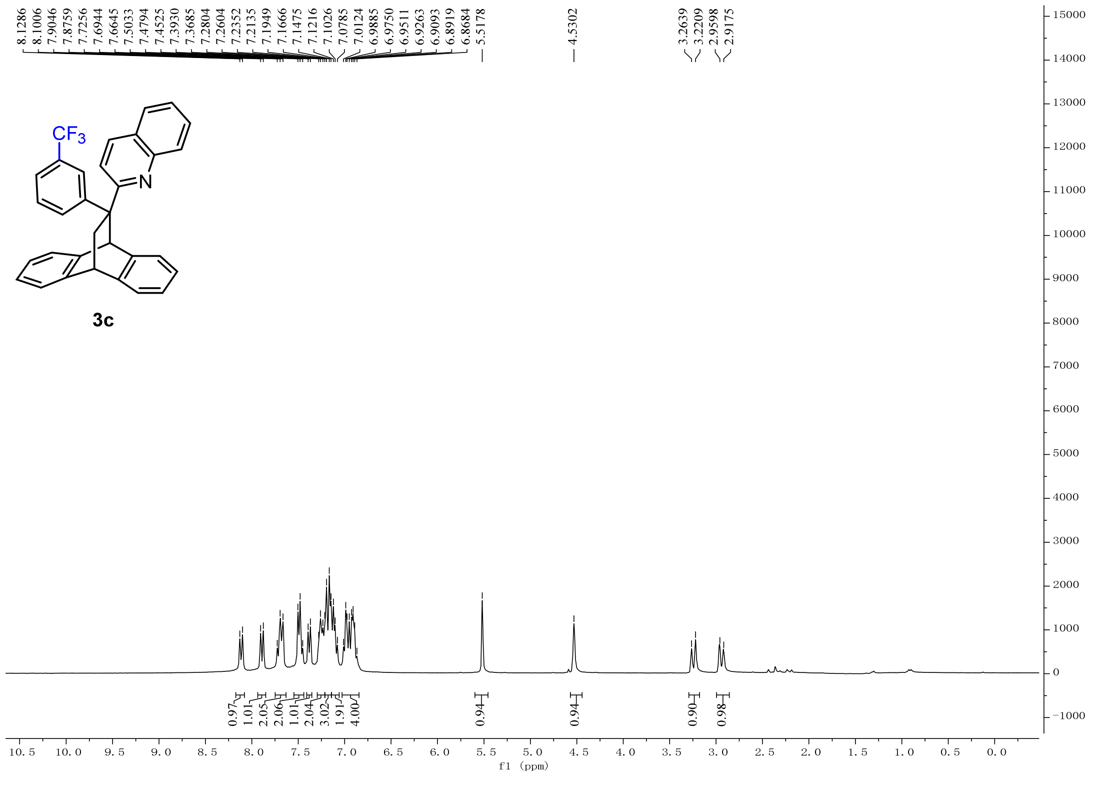

$^{13}$C NMR (75 MHz, Chloroform-*d*) of compound **3c**

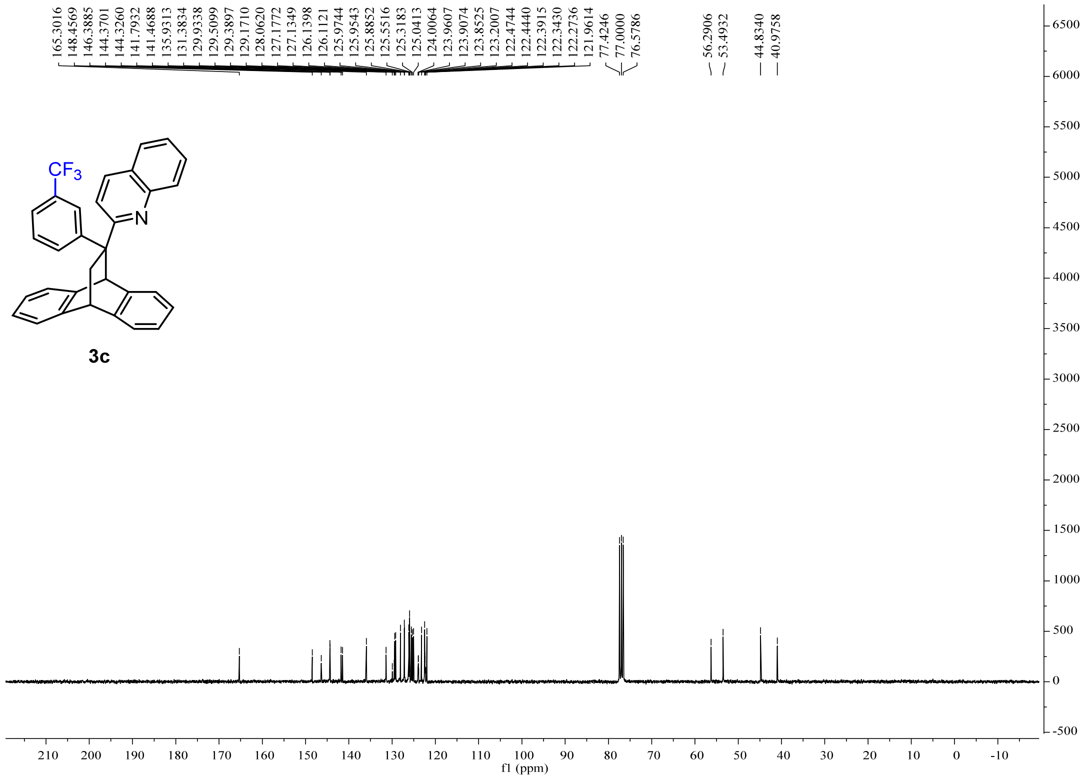


$^{19}$F NMR (565 MHz, Chloroform-*d*) of compound **3c**

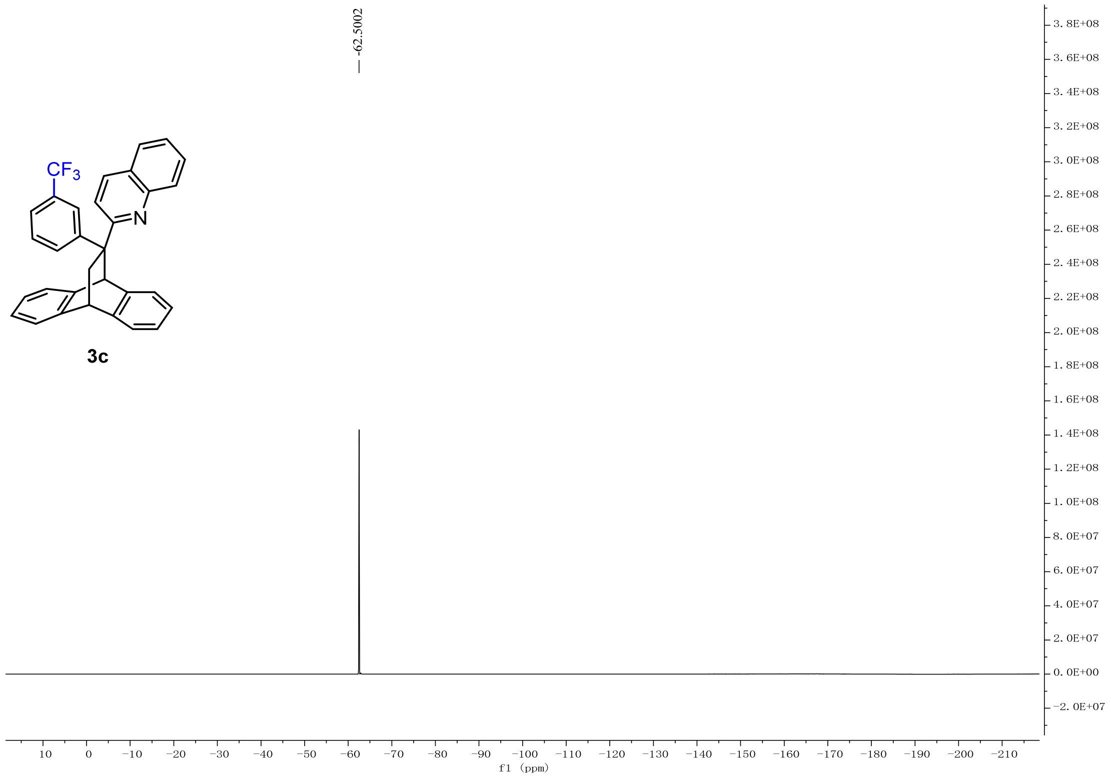

$^{1}$H NMR (300 MHz, Chloroform-*d*) of compound **3d**

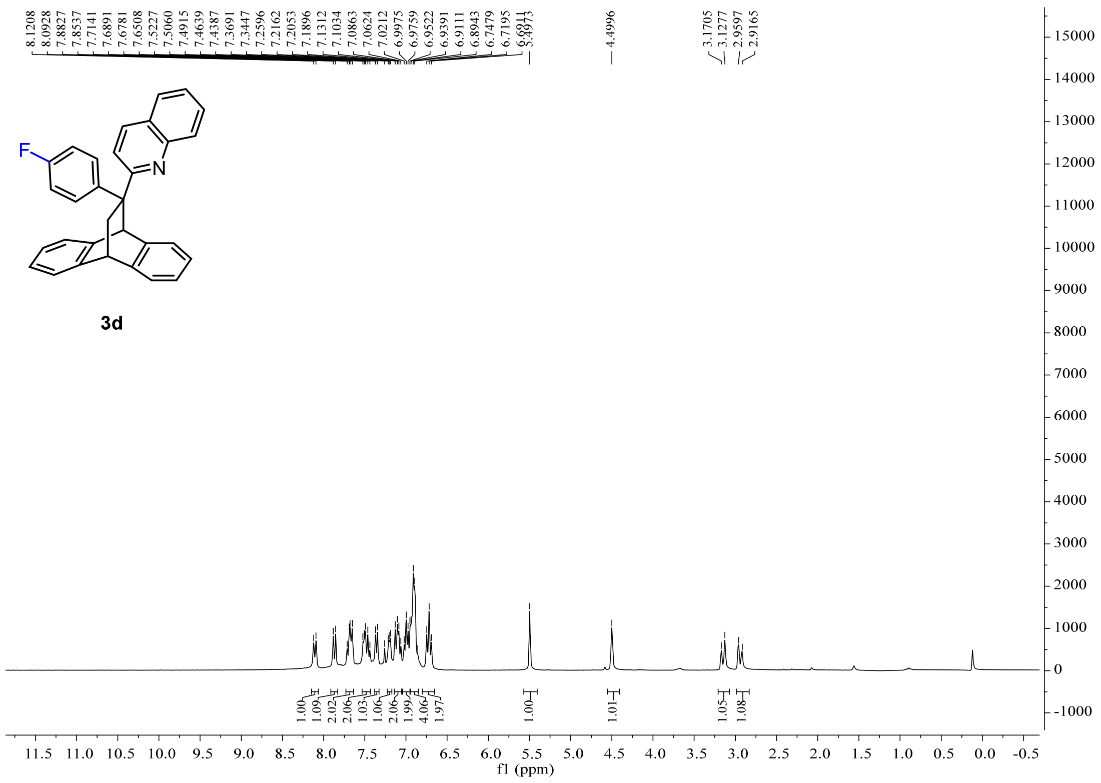

$^{13}$C NMR (75 MHz, Chloroform-*d*) of compound **3d**

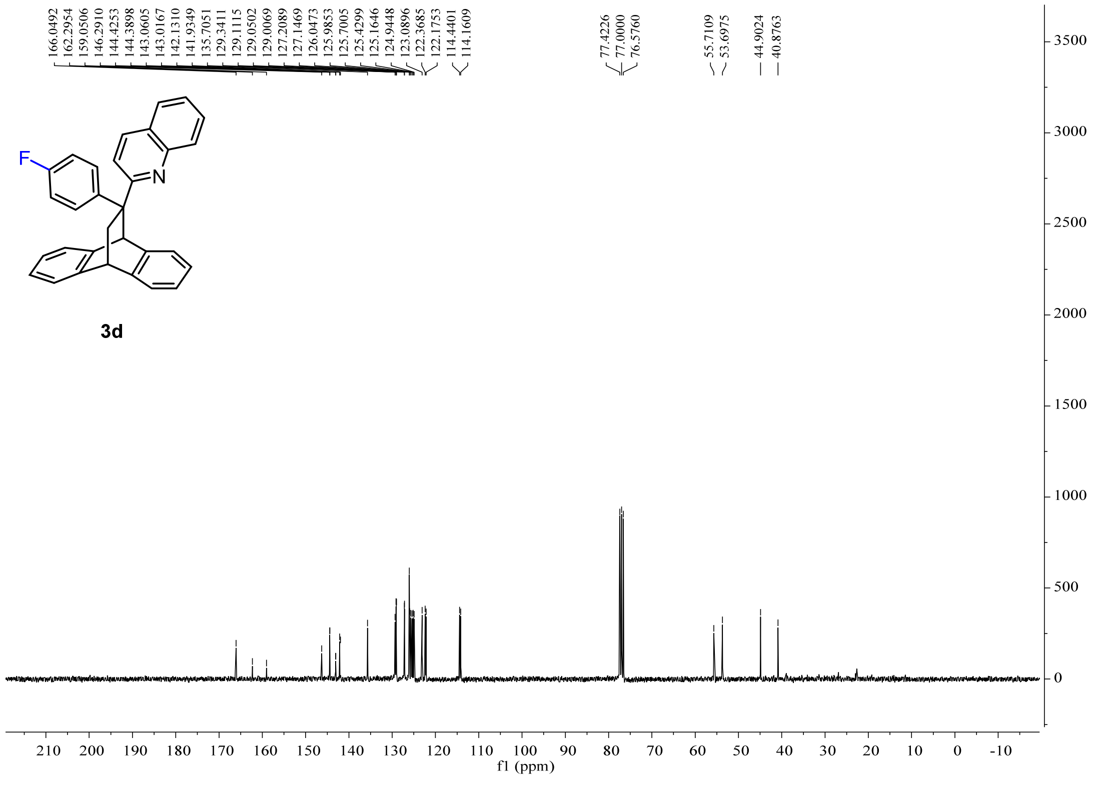

$^{19}$F NMR (376 MHz, Chloroform-*d*) of compound **3d**

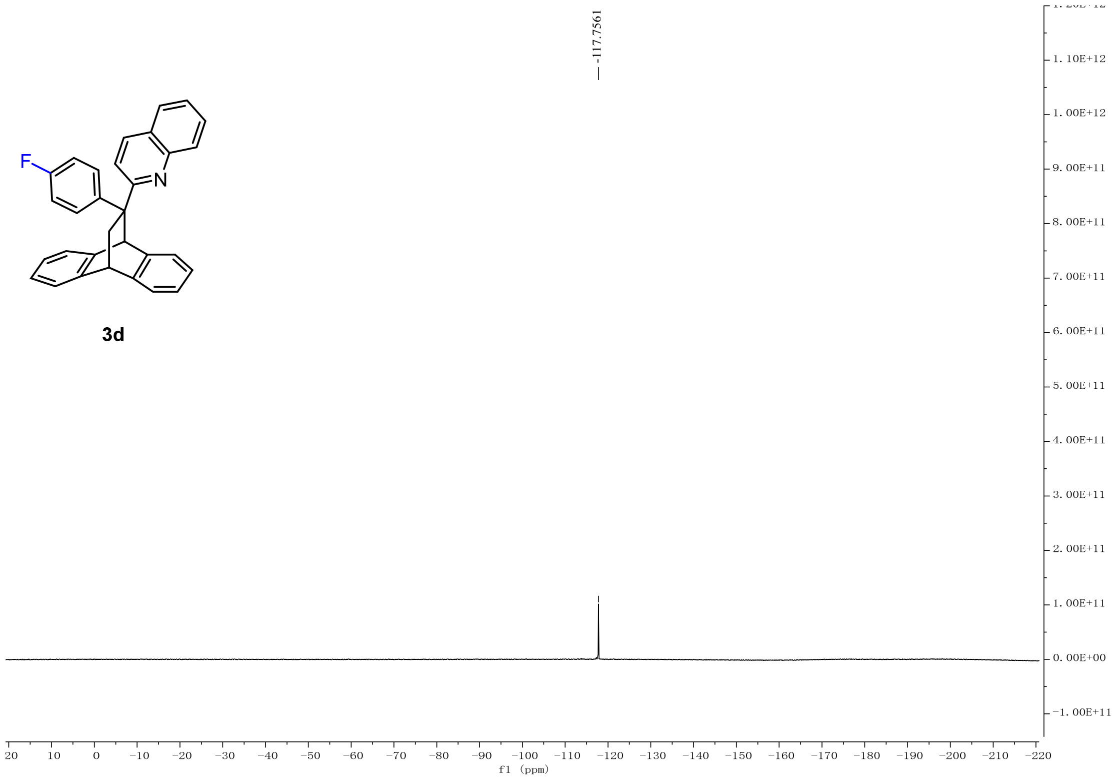

$^{1}$H NMR (300 MHz, Chloroform-*d*) of compound **3e**

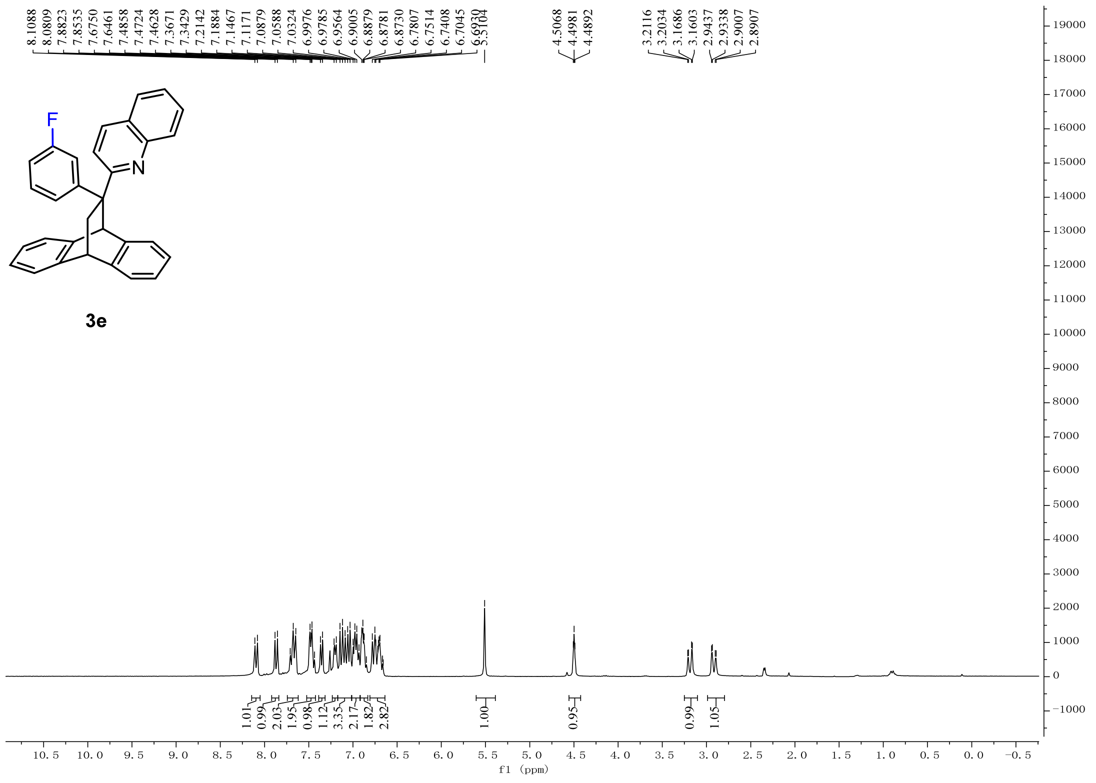

$^{13}$C NMR (75 MHz, Chloroform-*d*) of compound **3e**

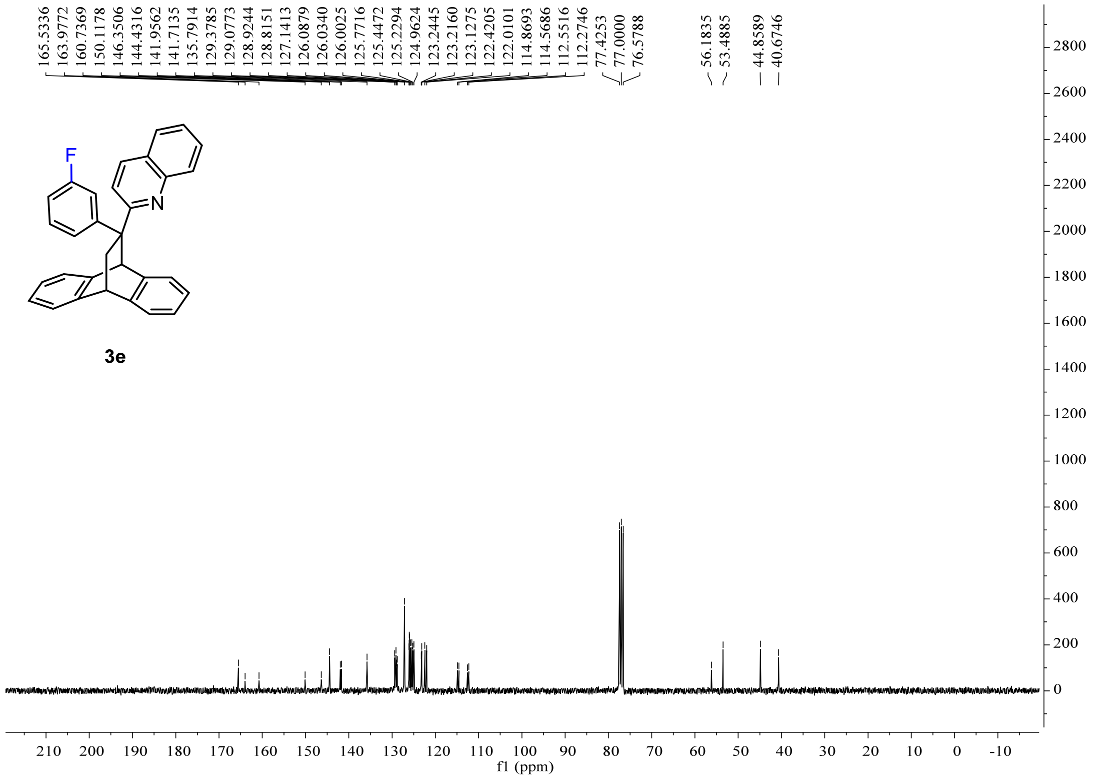


$^{19}$F NMR (565 MHz, Chloroform-*d*) of compound **3e**

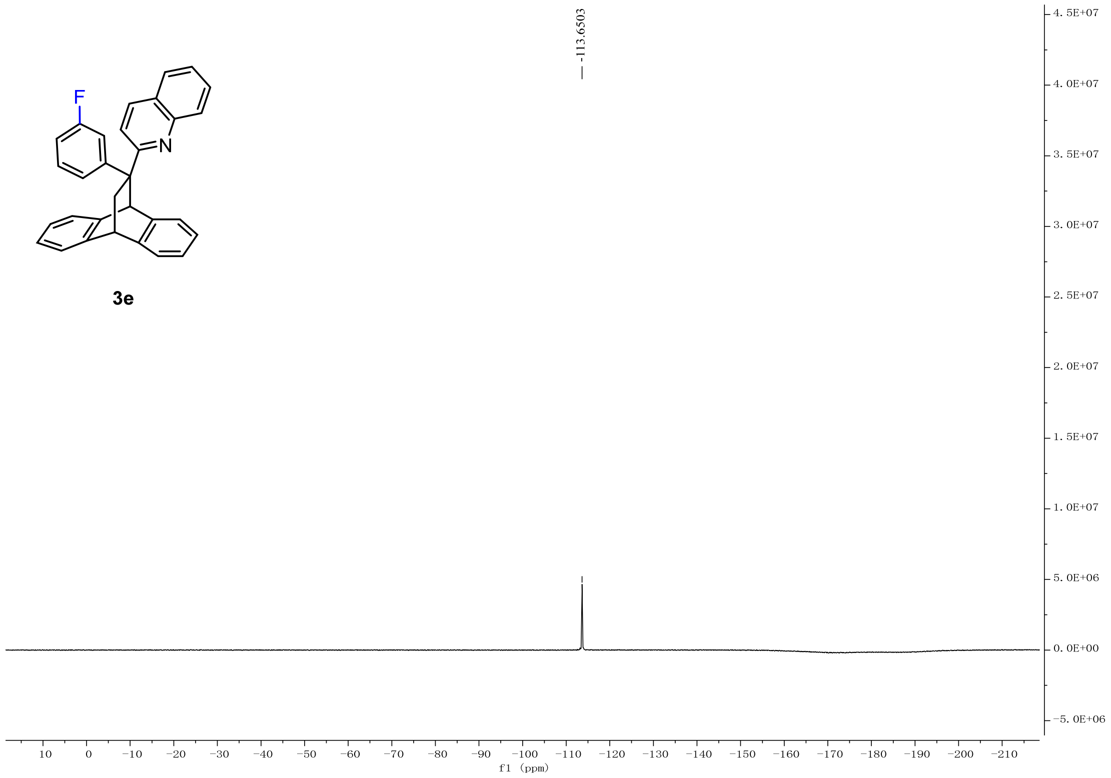

$^{1}$H NMR (300 MHz, Chloroform-*d*) of compound **3f**

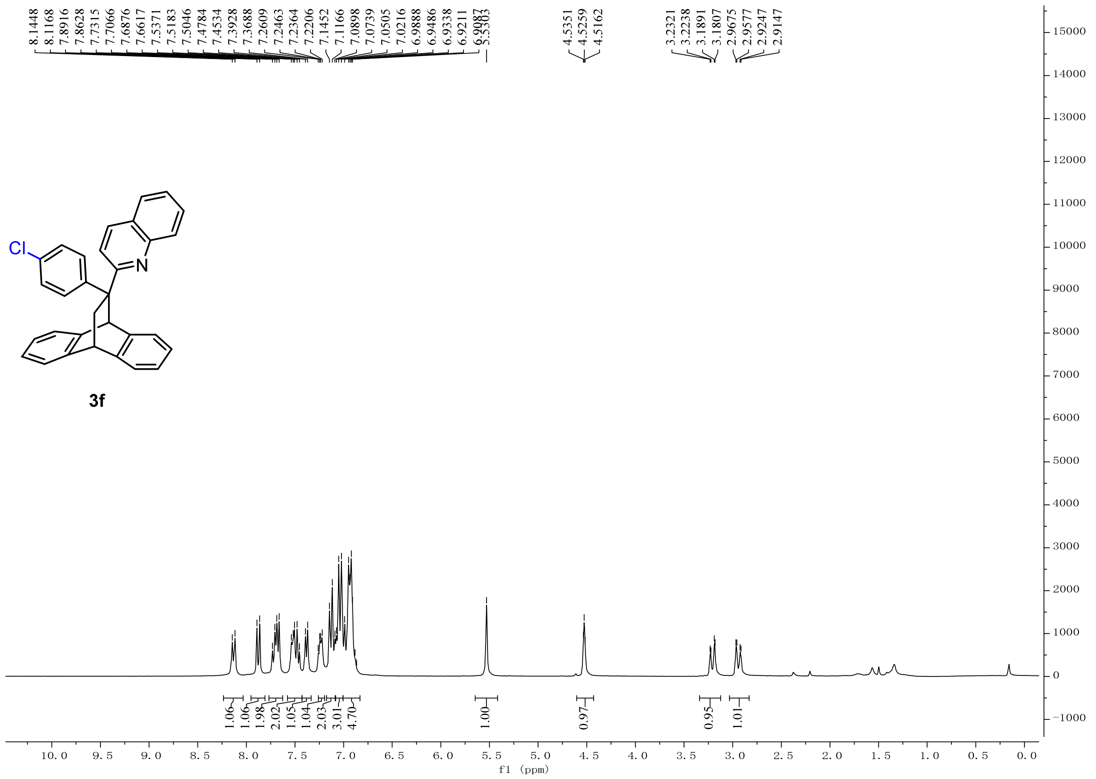


$^{13}$C NMR (75 MHz, Chloroform-*d*) of compound **3f**

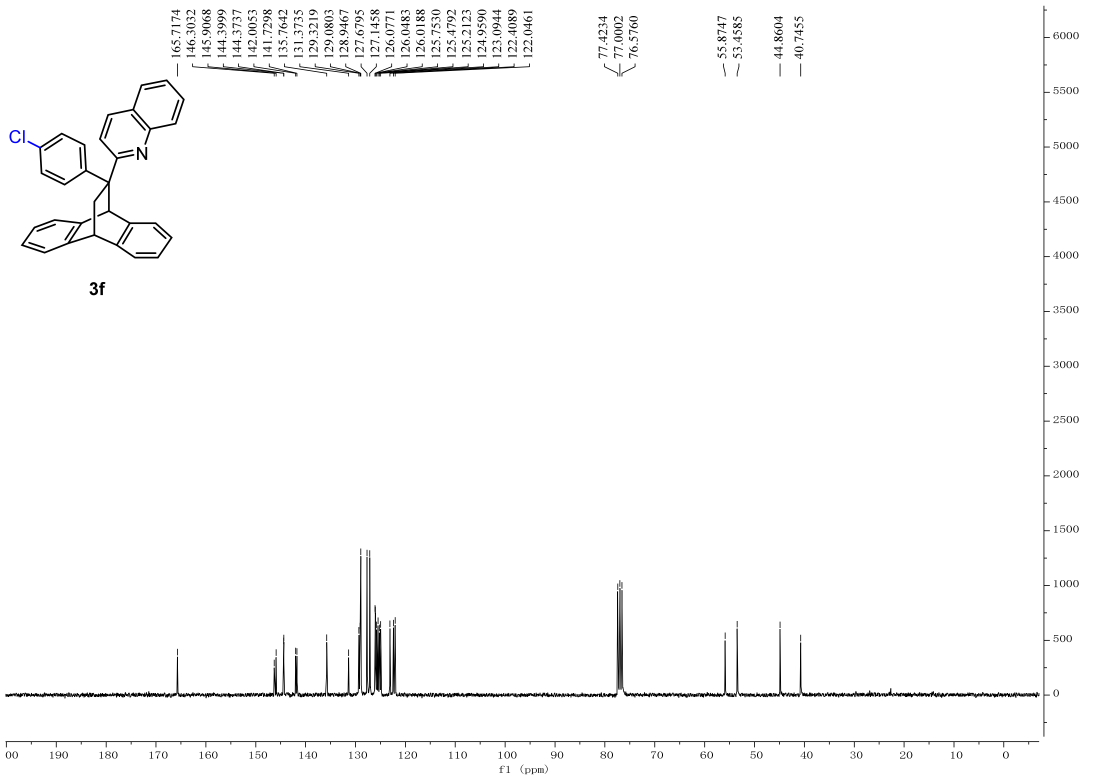

$^{1}$H NMR (300 MHz, Chloroform-*d*) of compound **3g**

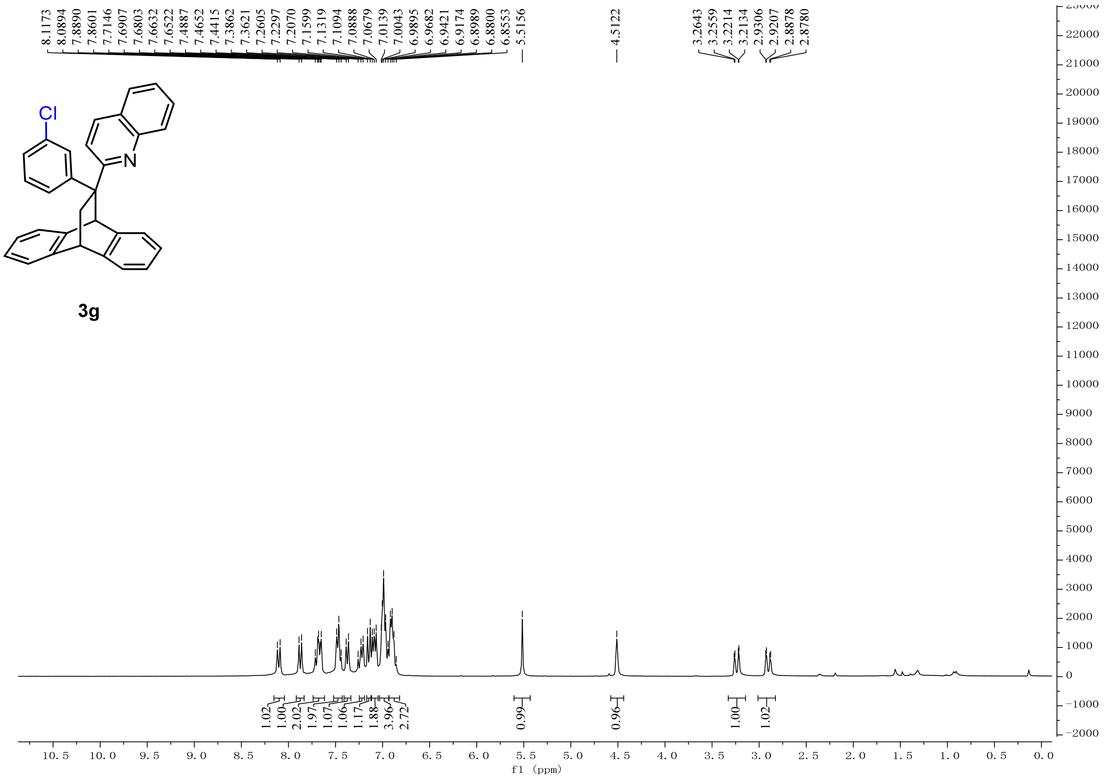


$^{13}$C NMR (75 MHz, Chloroform-*d*) of compound **3g**

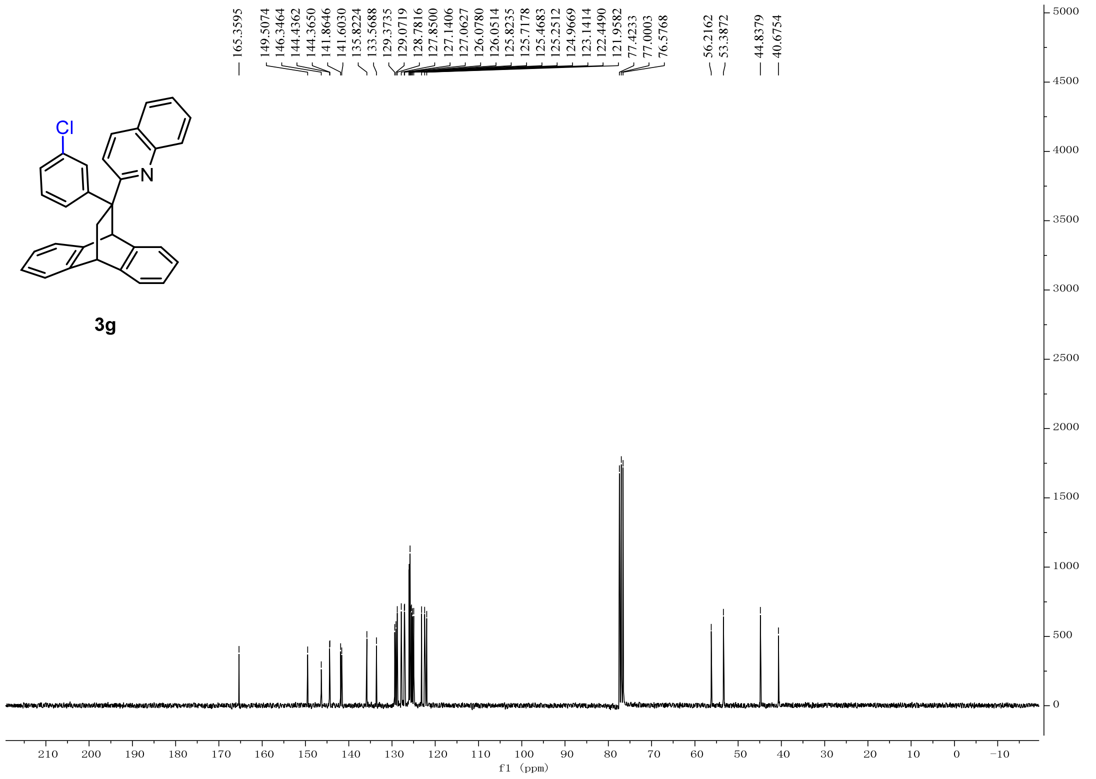

$^{1}$H NMR (300 MHz, Chloroform-*d*) of compound **3h**

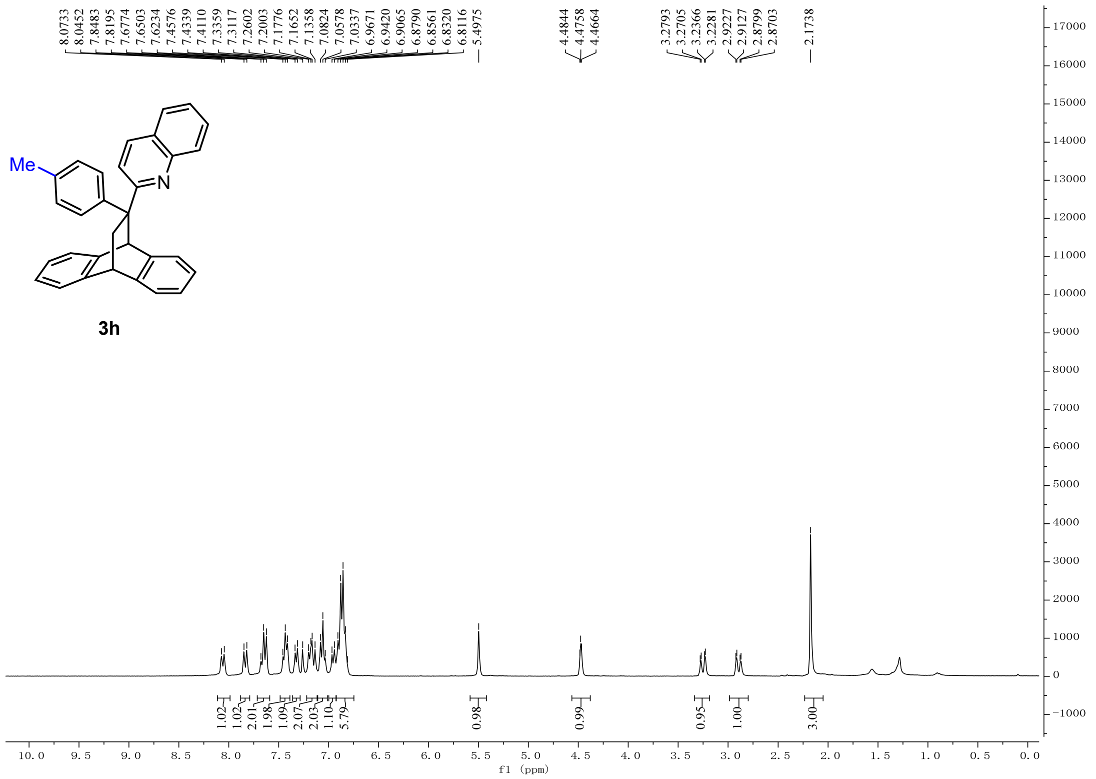


$^{13}$C NMR (75 MHz, Chloroform-*d*) of compound **3h**

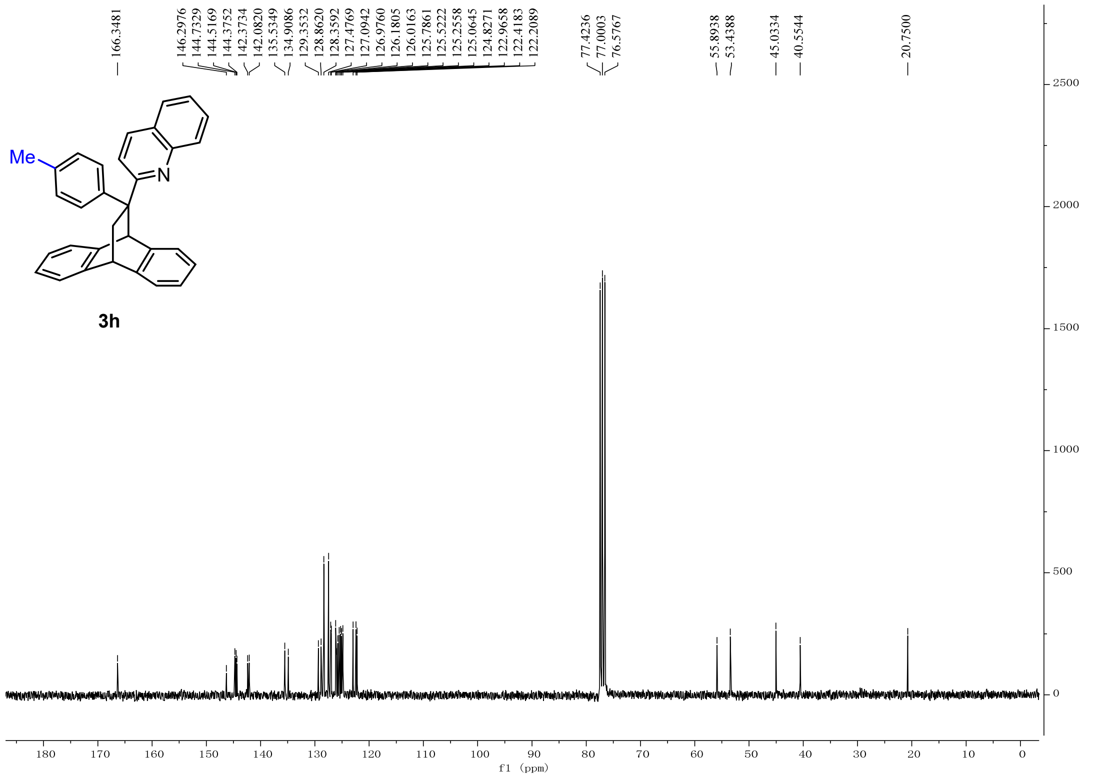

$^{1}$H NMR (300 MHz, Chloroform-*d*) of compound **3i**

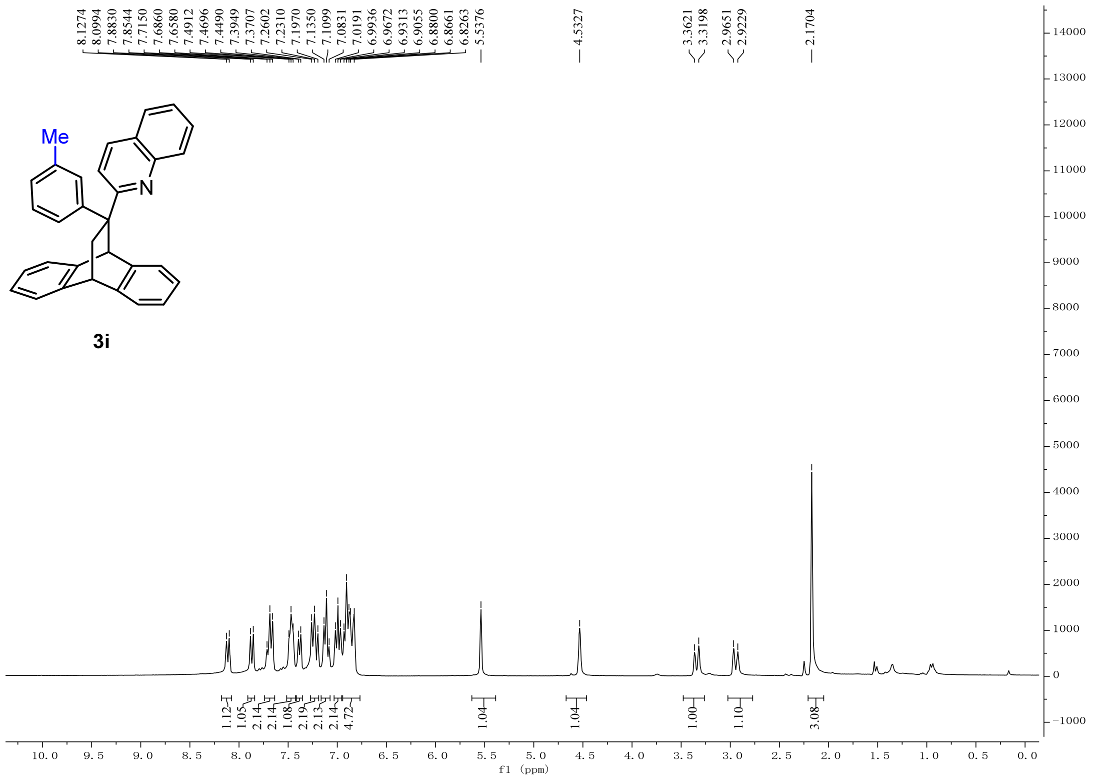

$^{13}$C NMR (75 MHz, Chloroform-*d*) of compound **3i**

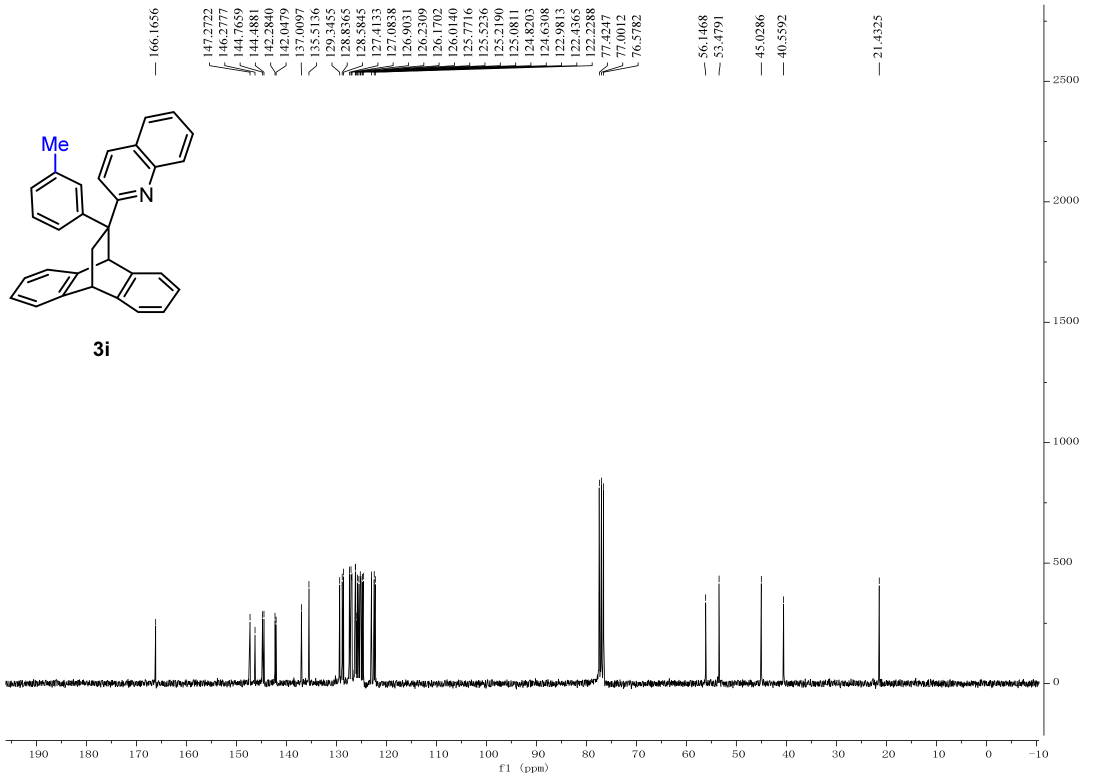

$^{1}$H NMR (300 MHz, Chloroform-*d*) of compound **3j**

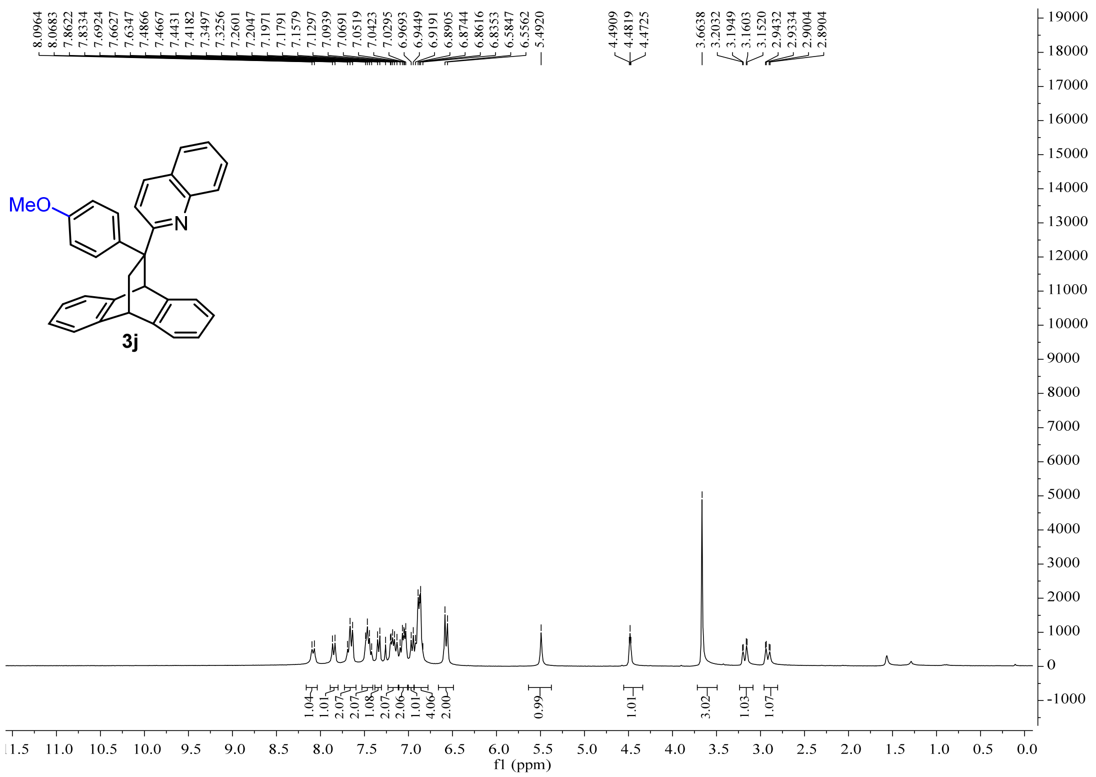


$^{13}$C NMR (75 MHz, Chloroform-*d*) of compound **3j**

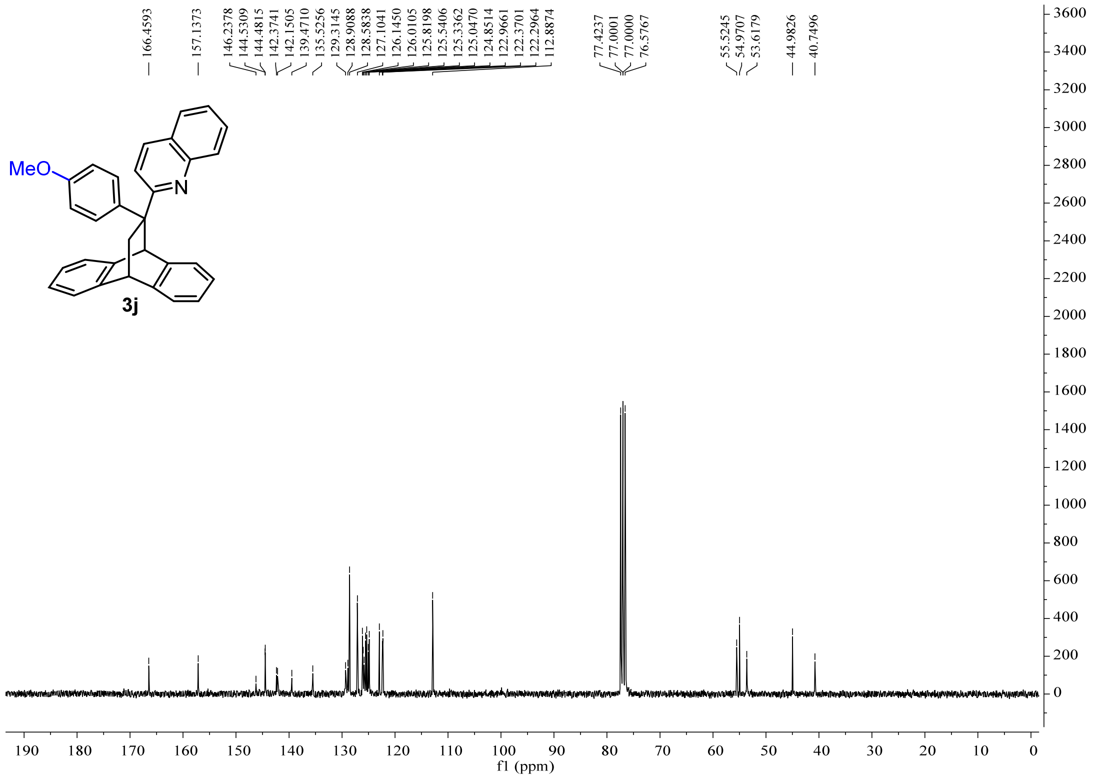

$^{1}$H NMR (300 MHz, Chloroform-*d*) of compound **3k**

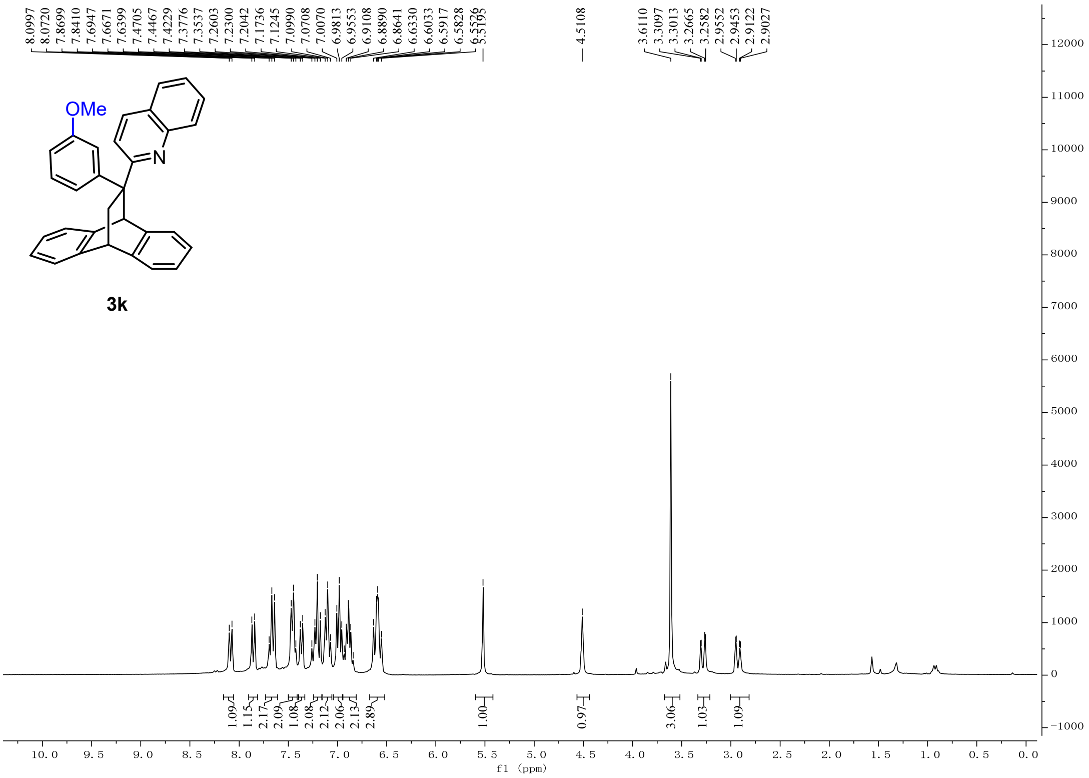


$^{13}$C NMR (75 MHz, Chloroform-*d*) of compound **3k**

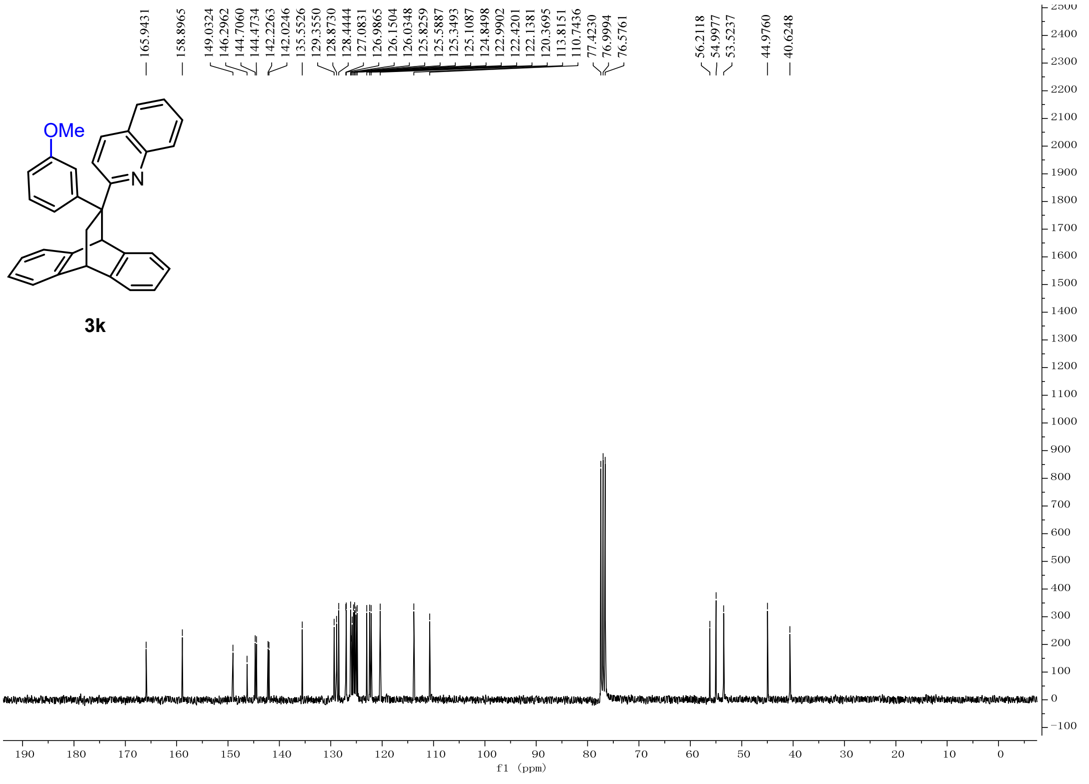

$^{1}$H NMR (300 MHz, Chloroform-*d*) of compound **3l**

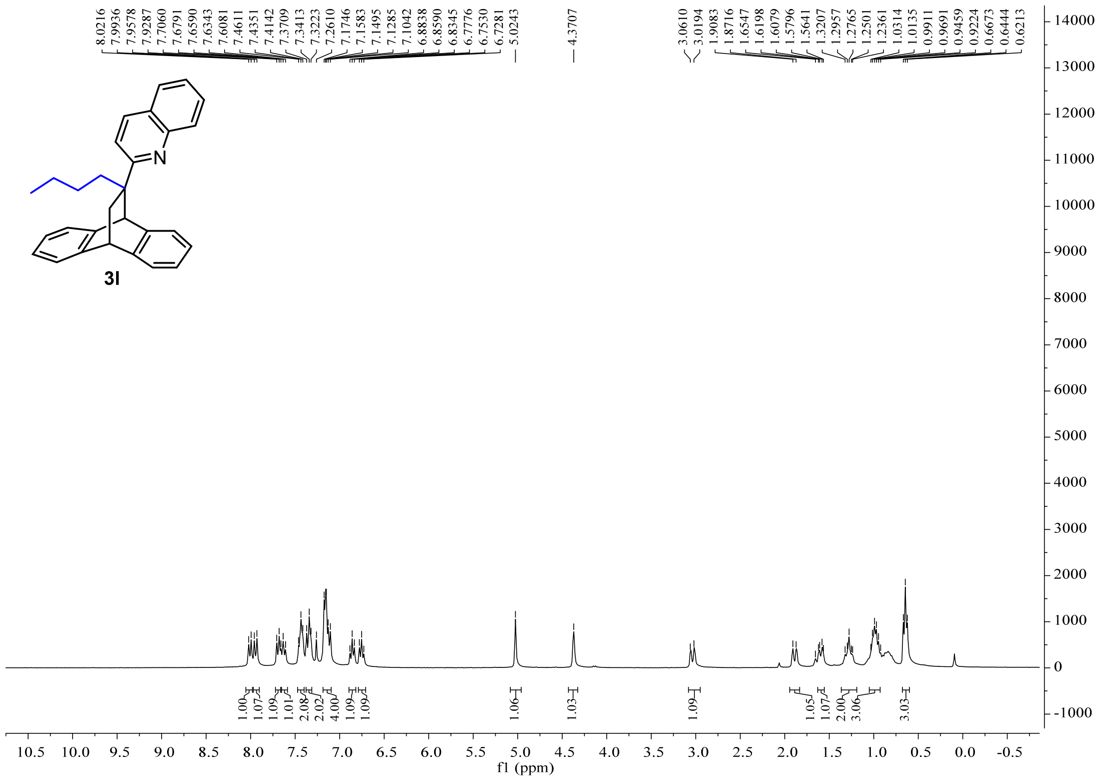

$^{13}$C NMR (75 MHz, Chloroform-*d*) of compound **3l**

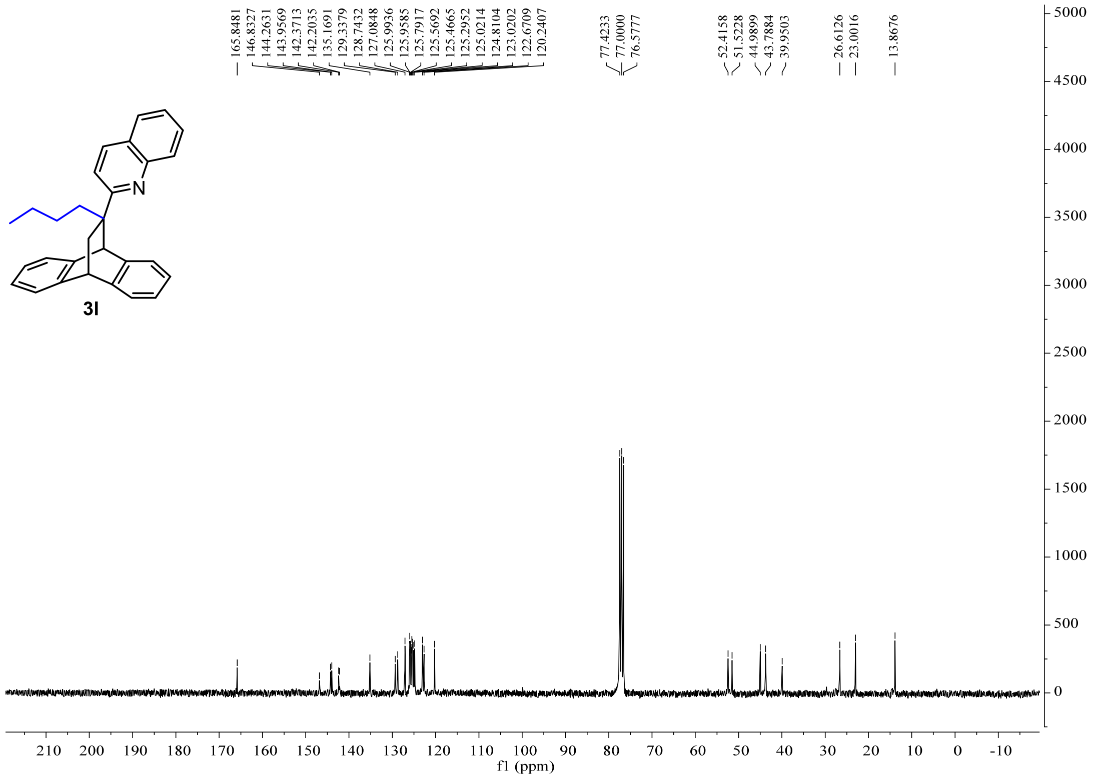

$^{1}$H NMR (300 MHz, Methylene Chloride-$d_2$) of compound **3m**

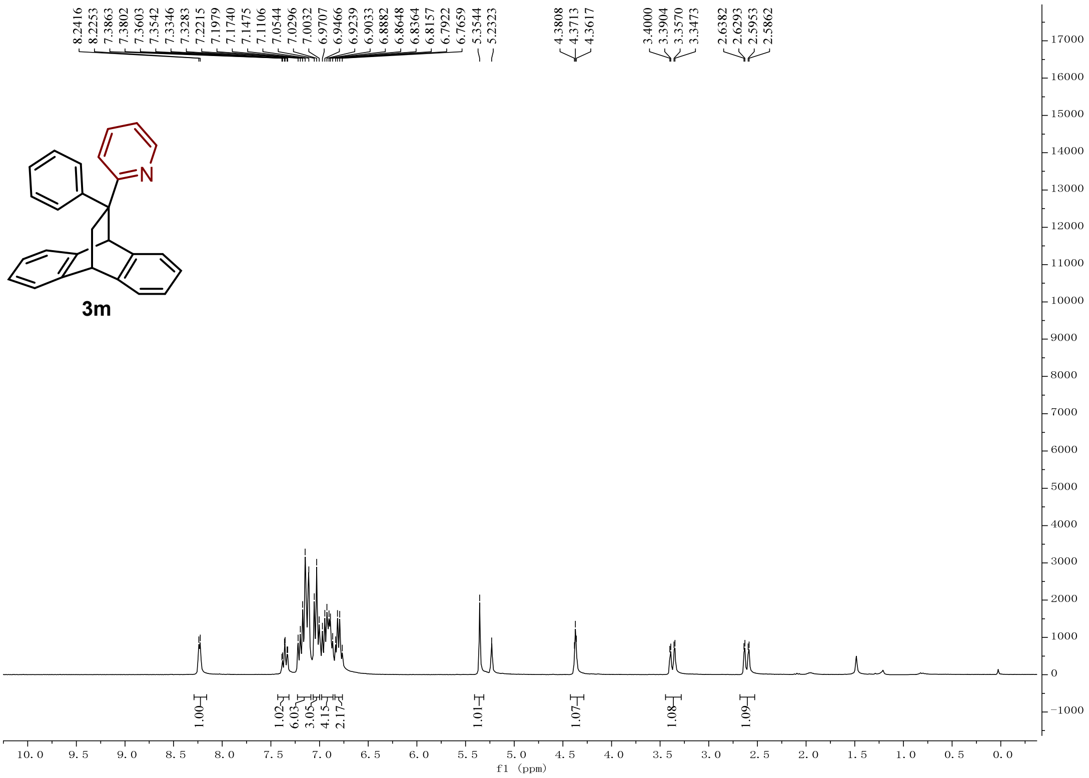


$^{13}$C NMR (75 MHz, Methylene Chloride-$d_2$) of compound **3m**

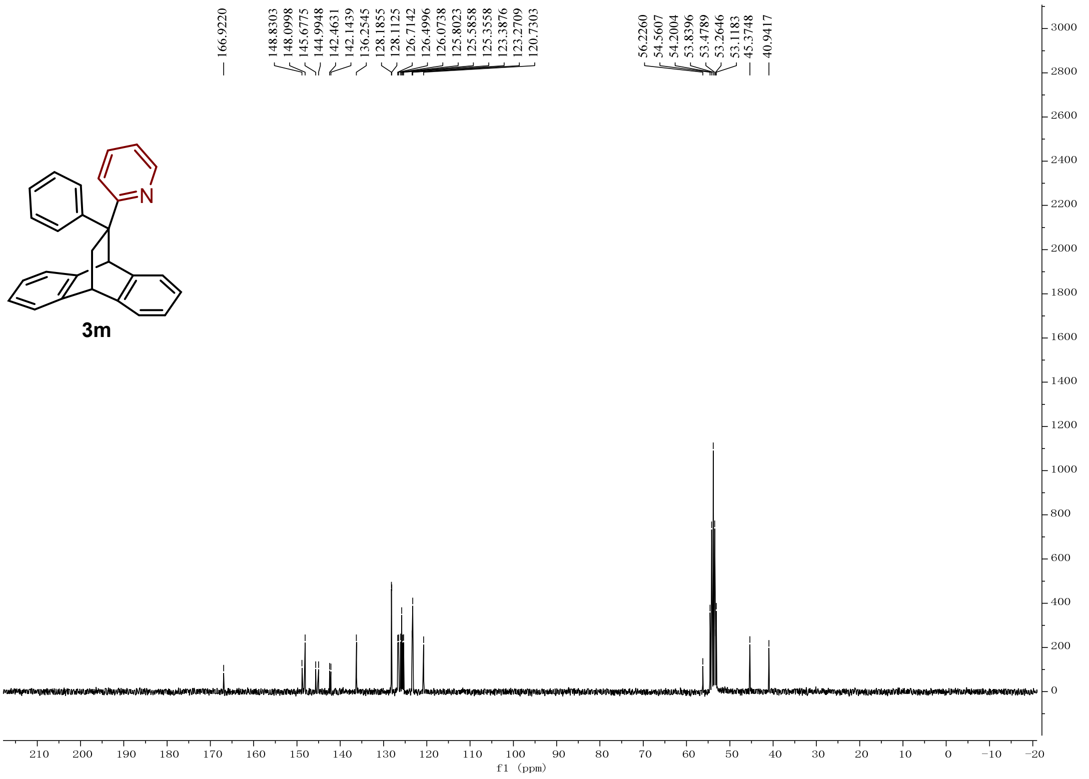

$^{1}$H NMR (300 MHz, Chloroform-*d*) of compound **3n**

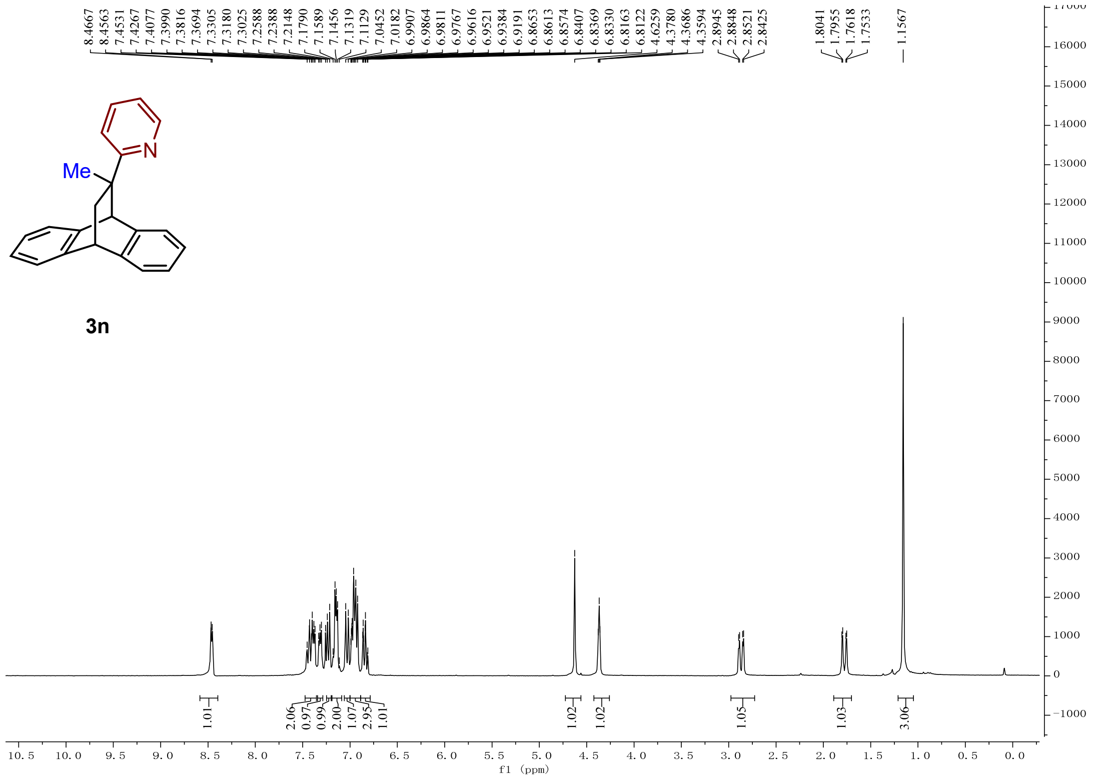


$^{13}$C NMR (75 MHz, Chloroform-*d*) of compound **3n**

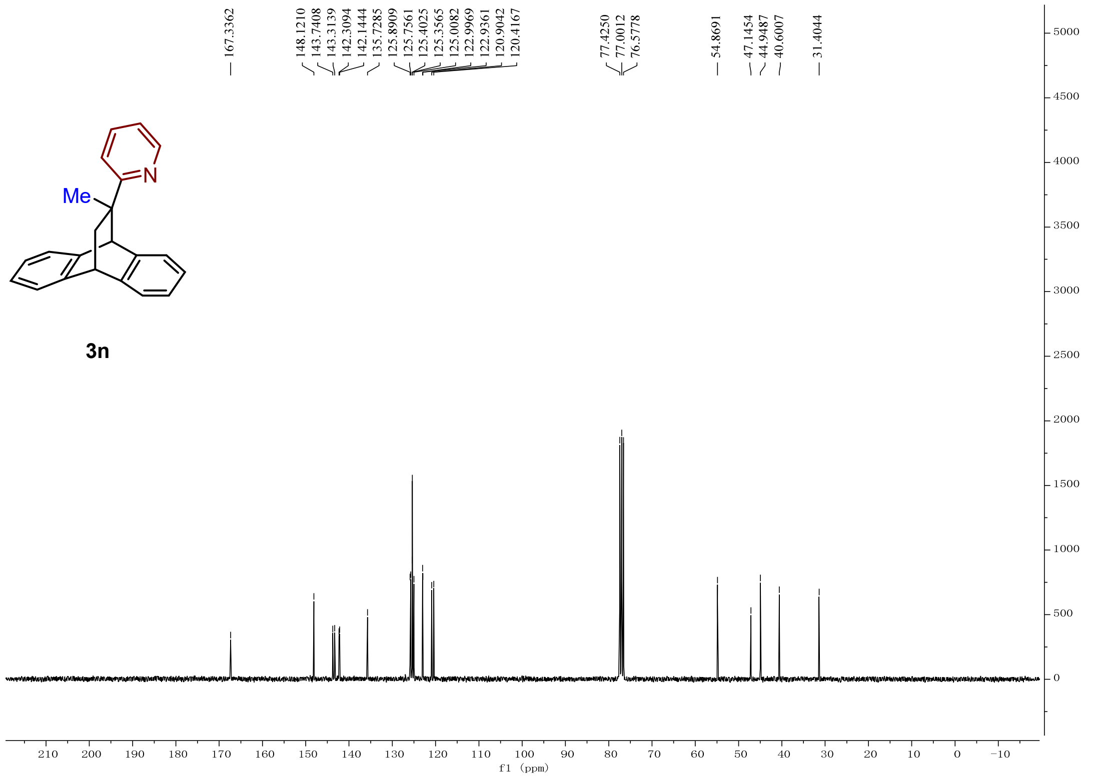

$^{1}$H NMR (300 MHz, Chloroform-*d*) of compound **3o**

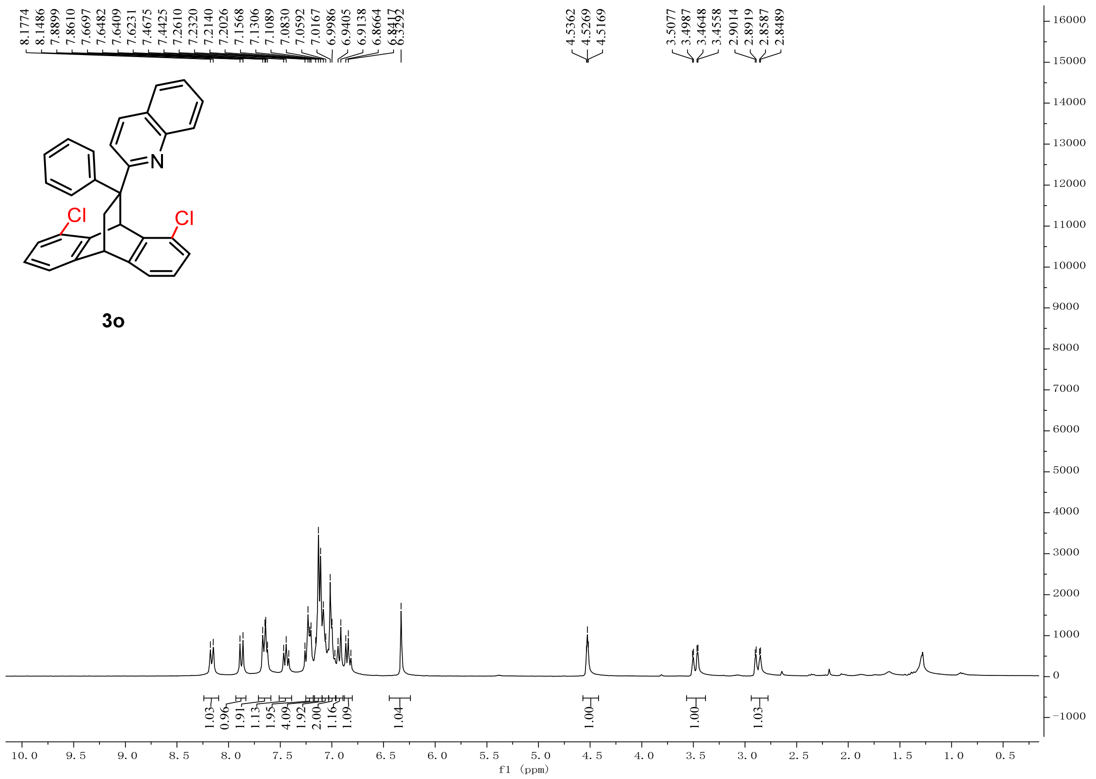


$^{13}$C NMR (75 MHz, Chloroform-*d*) of compound **3o**

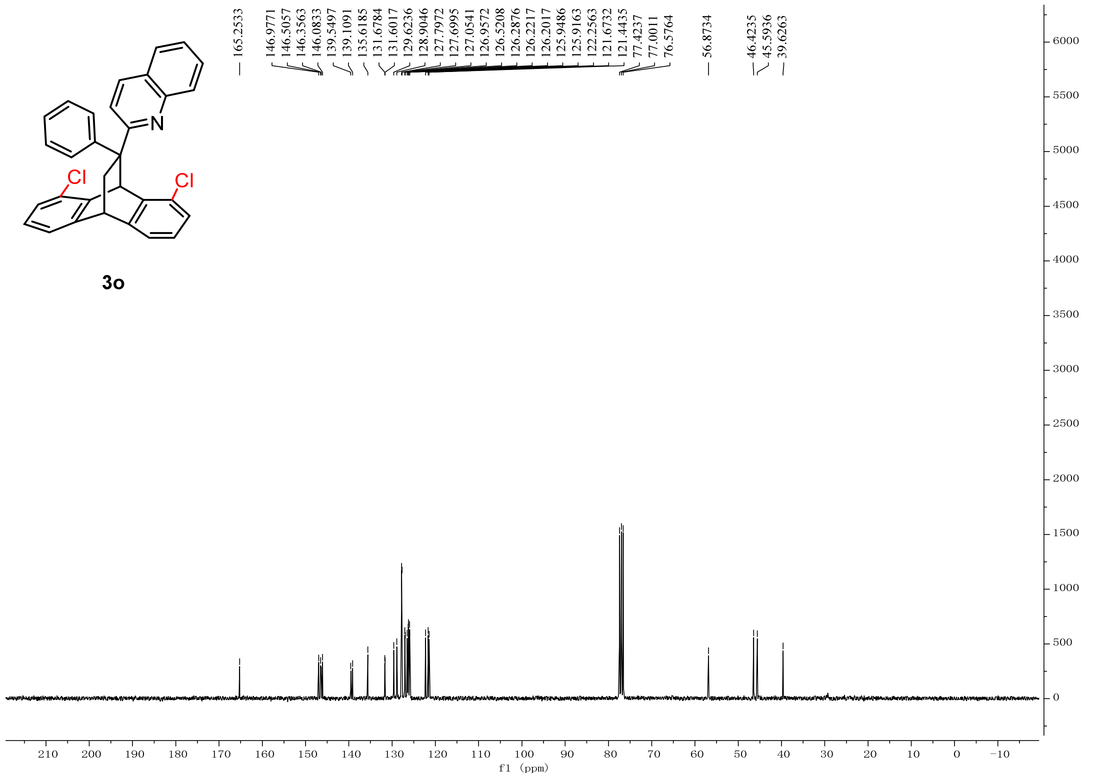

$^{1}$H NMR (300 MHz, Chloroform-*d*) of compound **3p**

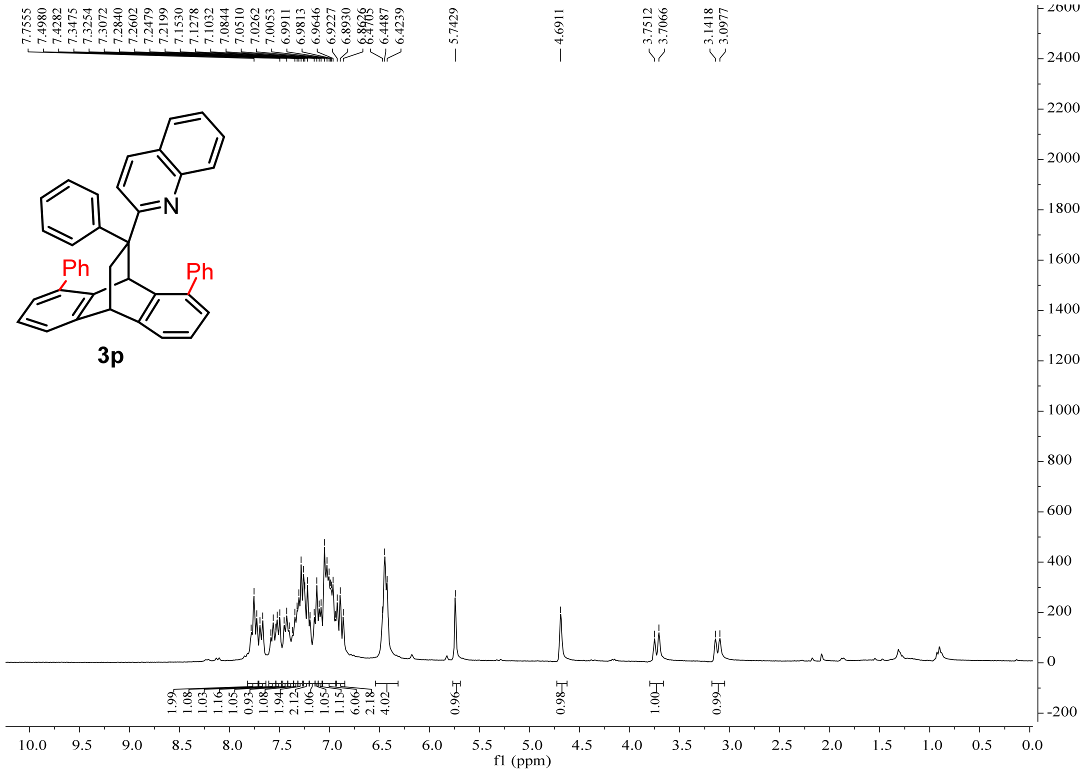

$^{13}$C NMR (75 MHz, Chloroform-*d*) of compound **3p**

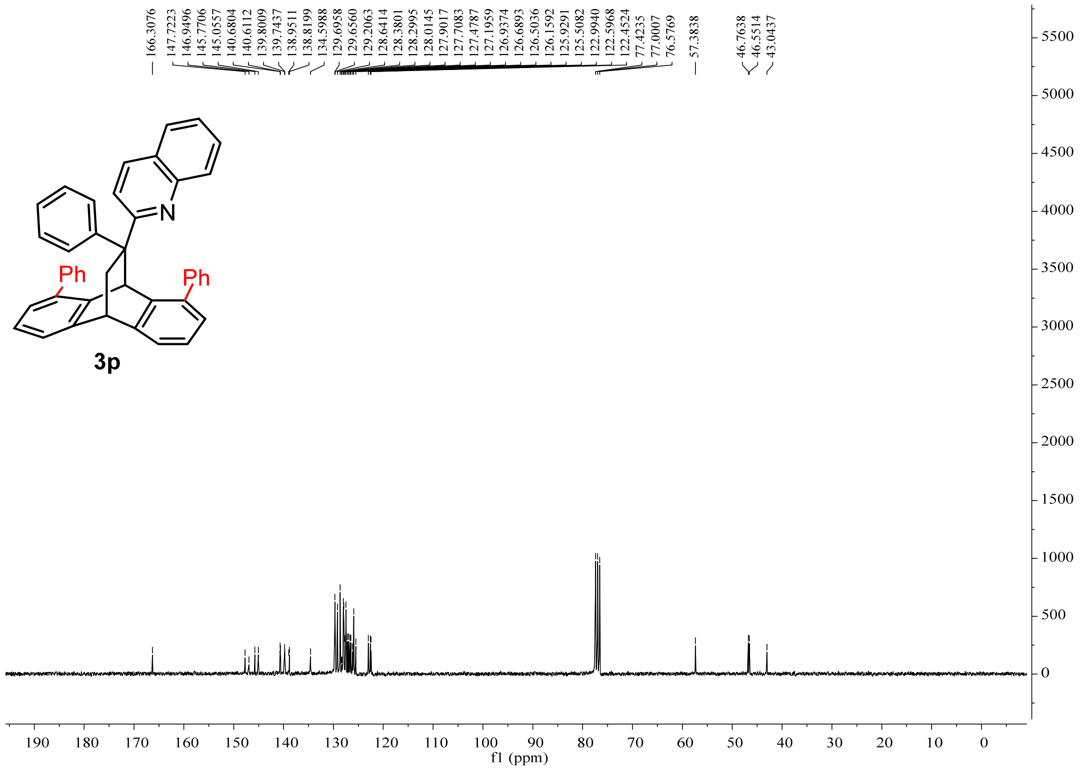

$^{1}$H NMR (300 MHz, Chloroform-*d*) of compound **3q**

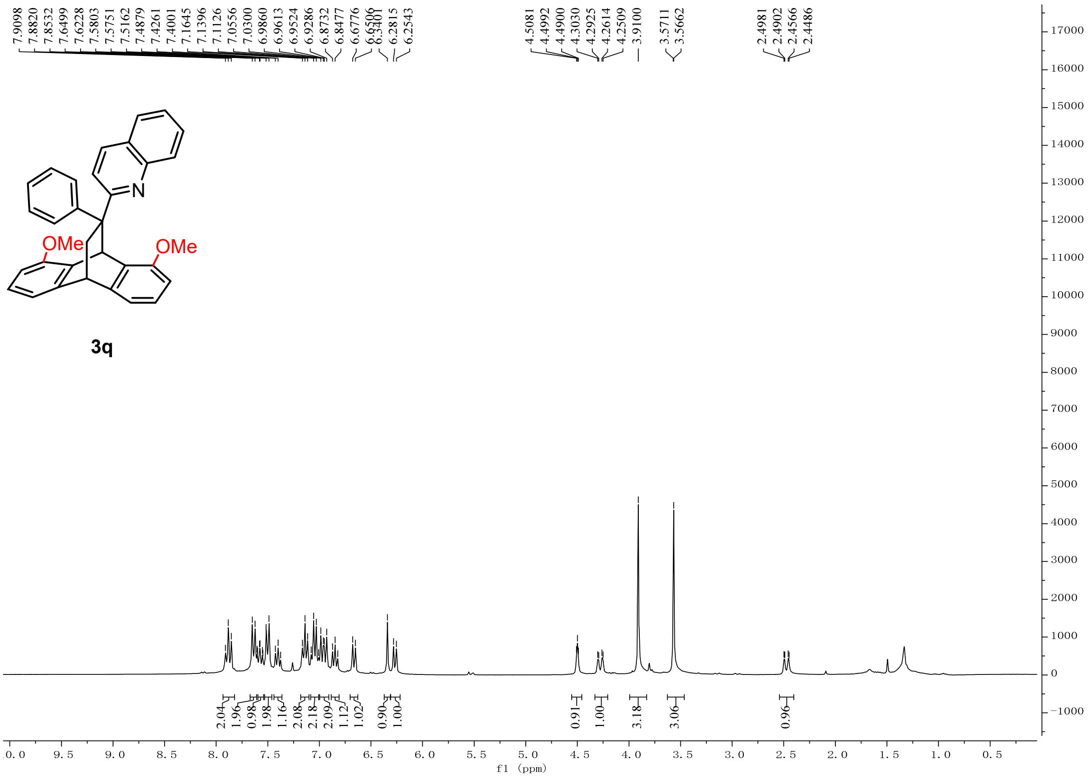


$^{13}$C NMR (75 MHz, Chloroform-*d*) of compound **3q**

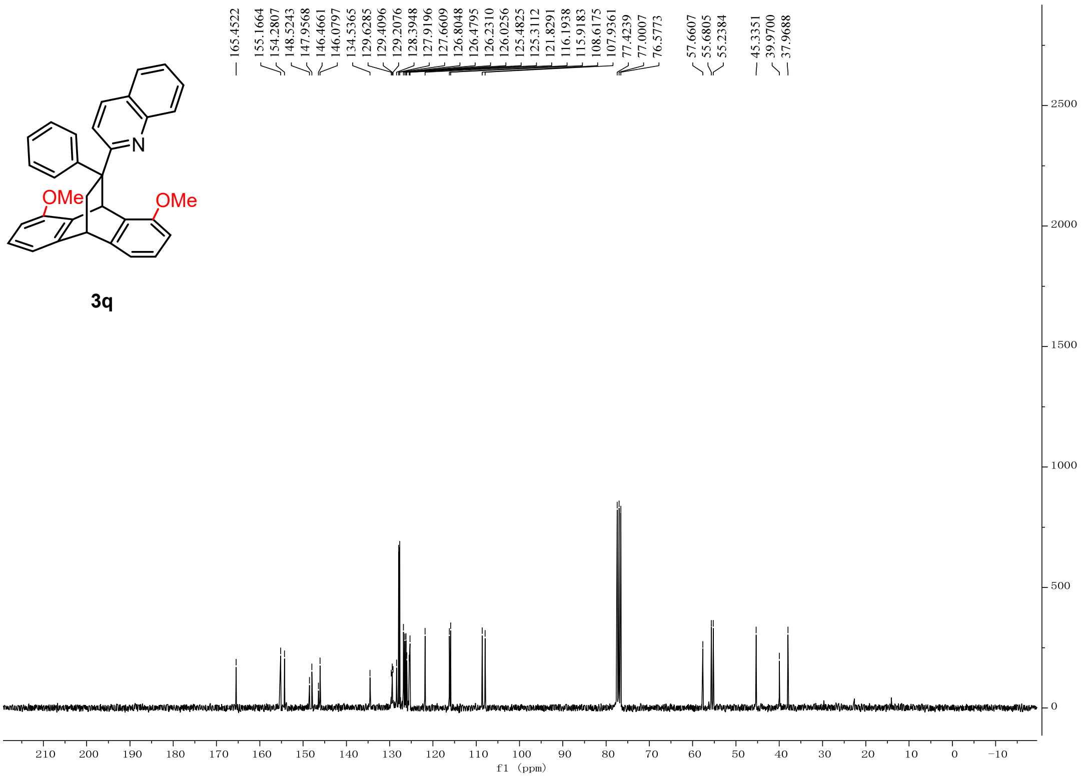

$^{1}$H NMR (300 MHz, Chloroform-*d*) of compound **3r**

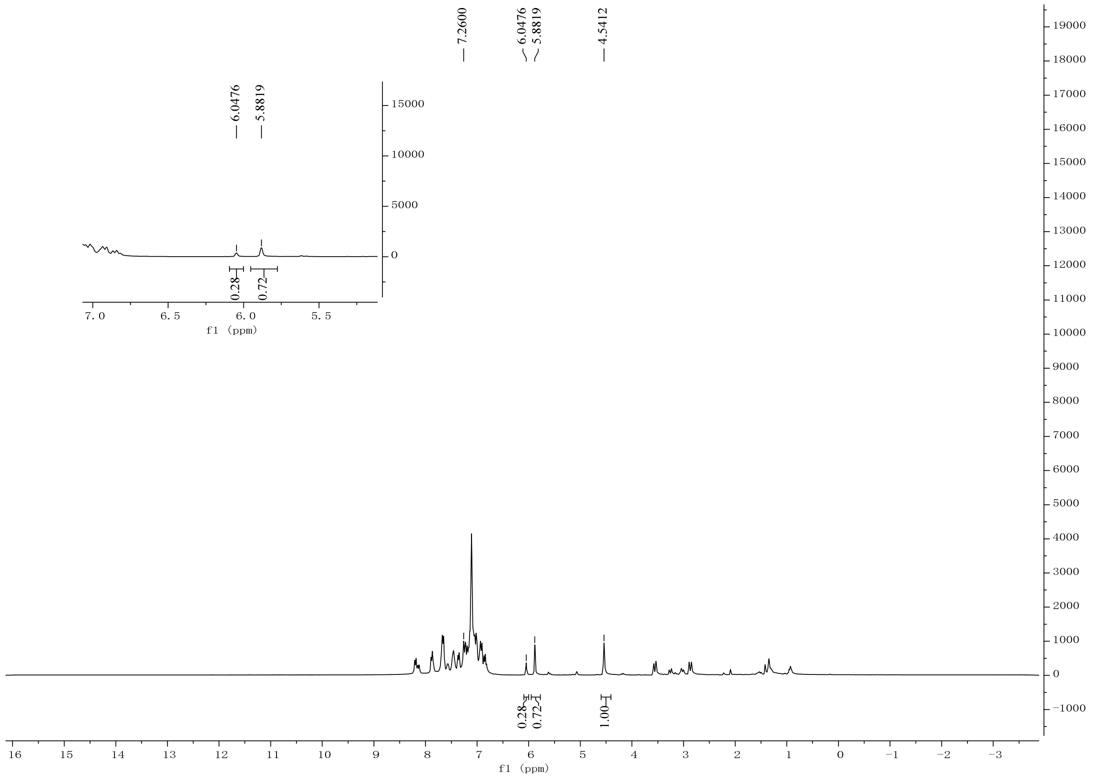


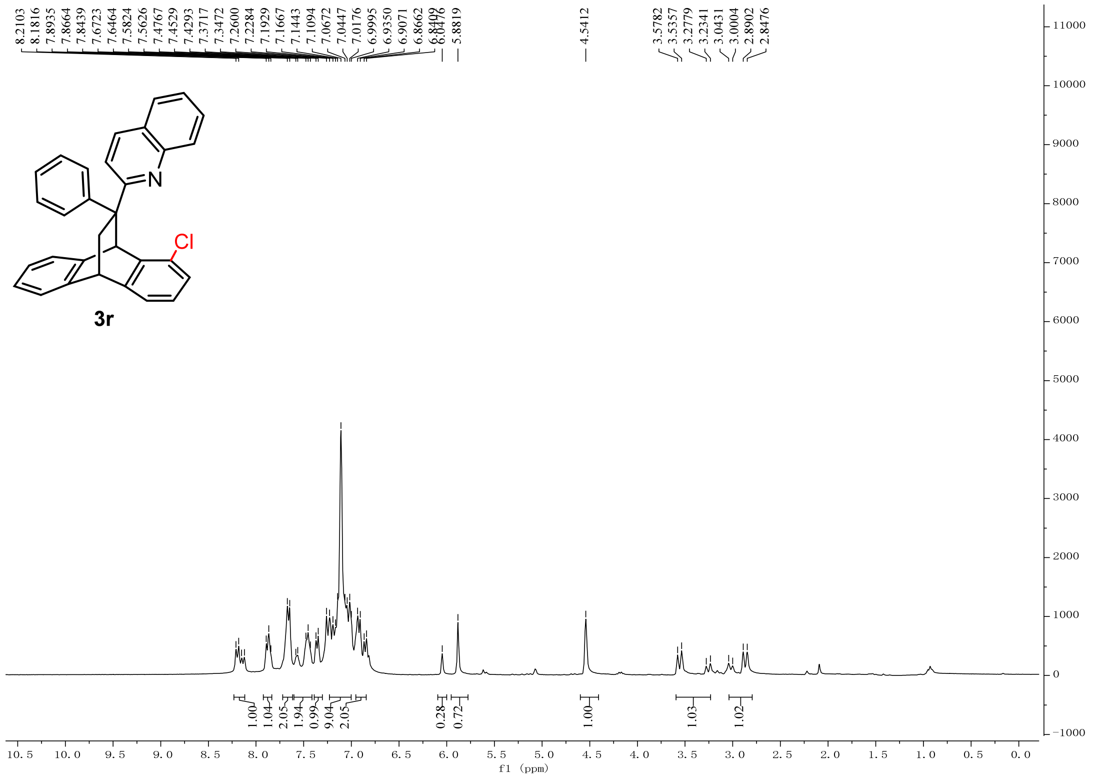

## $^{13}$C NMR (75 MHz, Chloroform-*d*) of compound **3r**

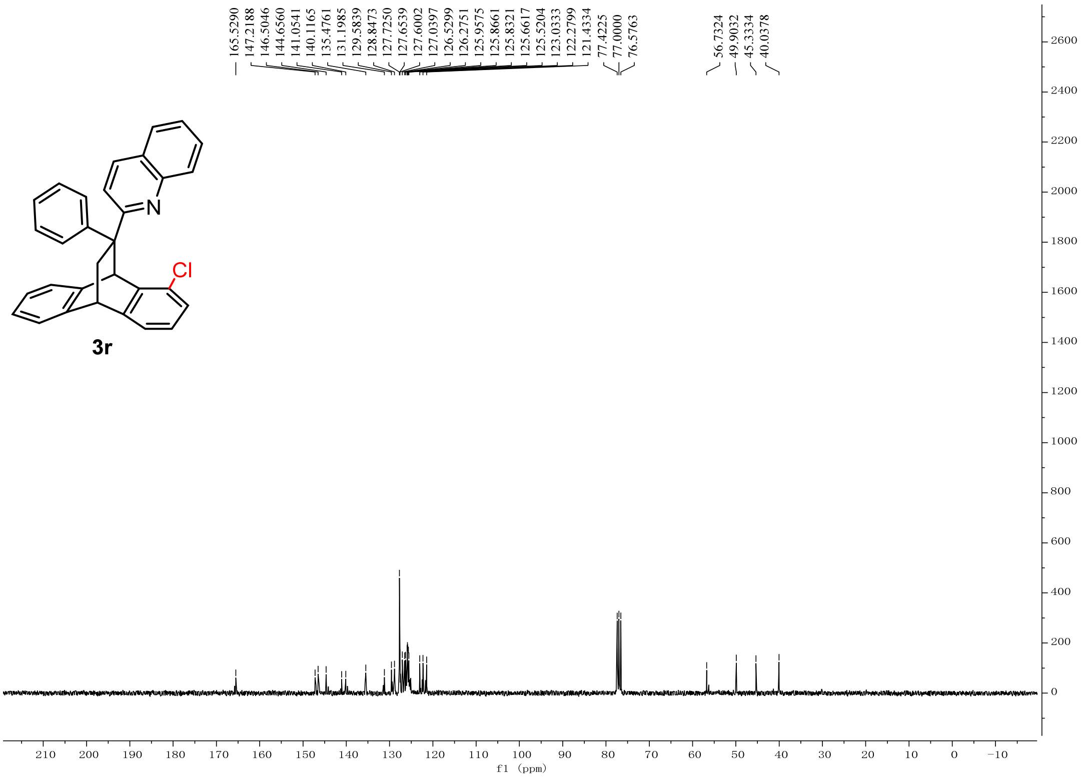


## $^{1}$H NMR (300 MHz, Chloroform-*d*) of compound **3s**

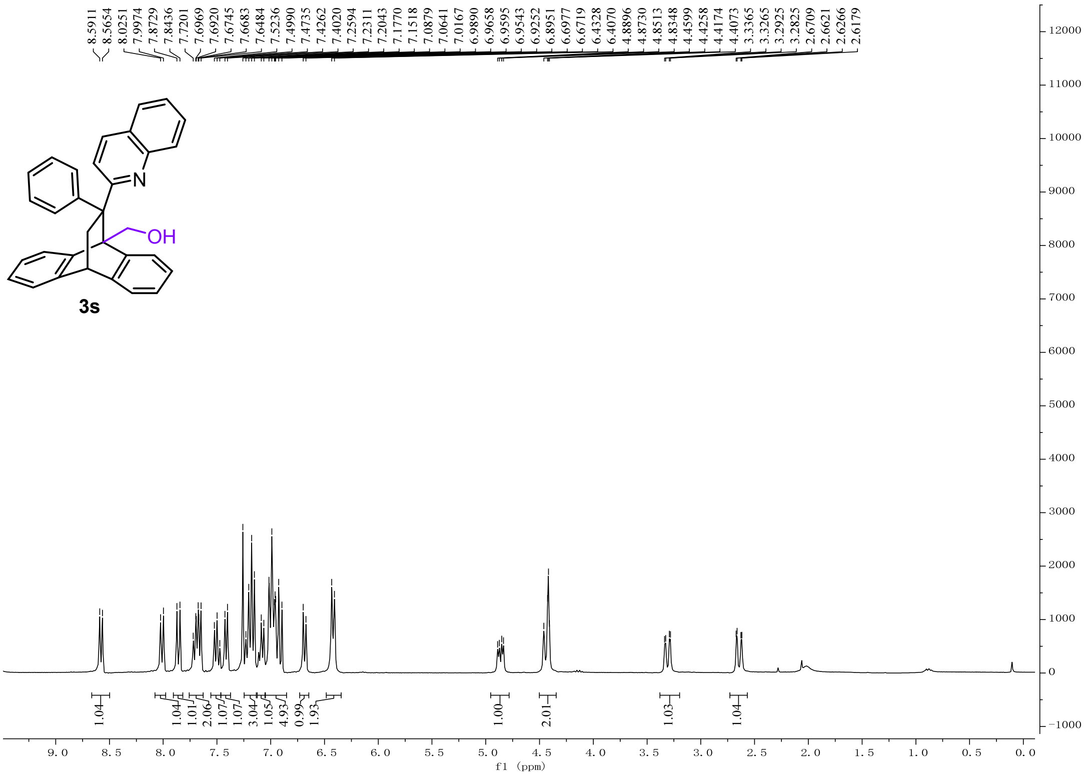

$^{13}C$ NMR (75 MHz, Chloroform-*d*) of compound **3s**

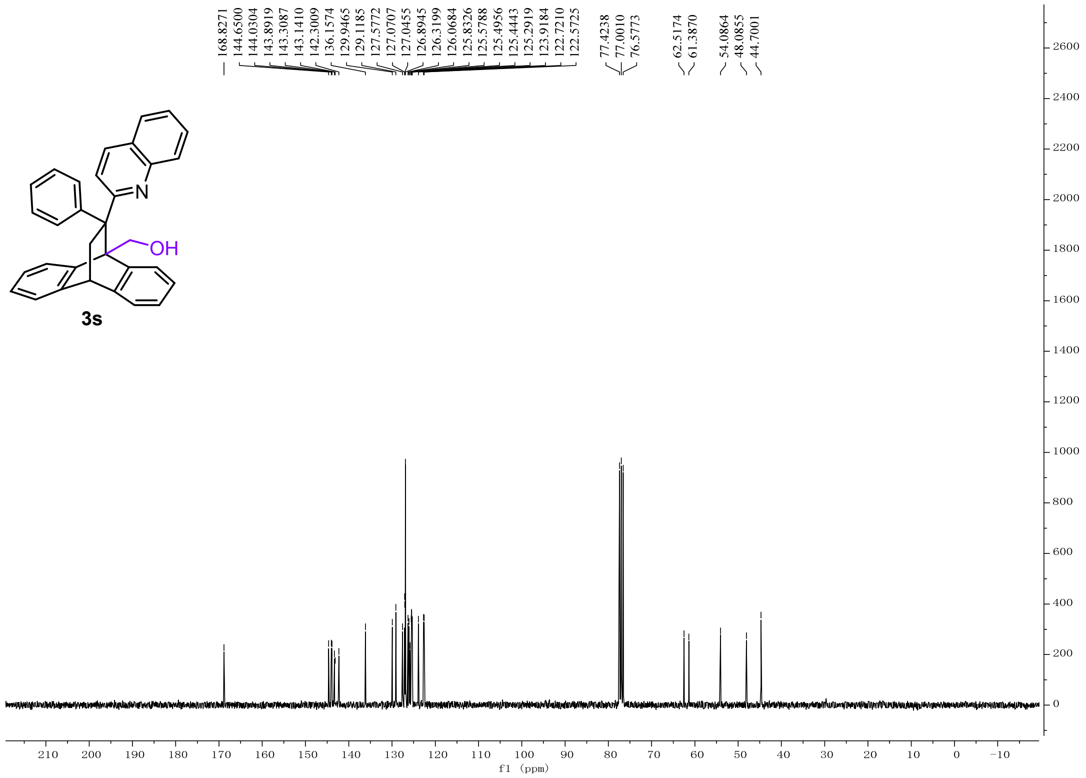


$^{1}H$ NMR (300 MHz, Chloroform-*d*) of compound **3t**

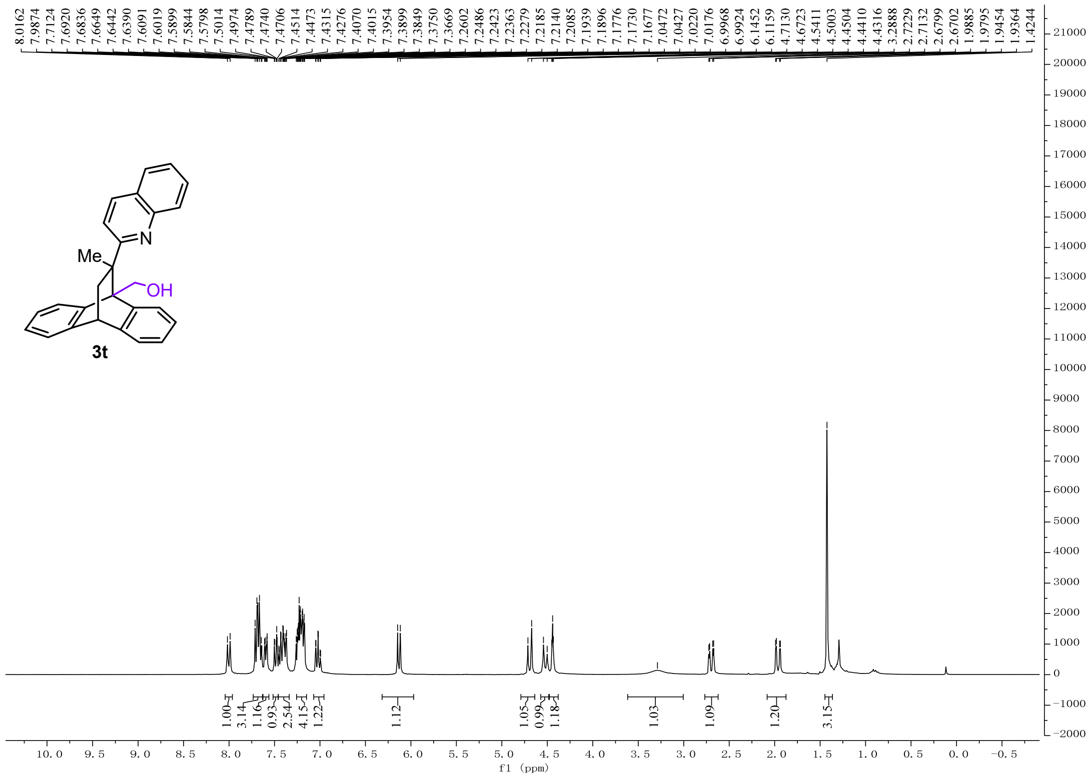

## $^{13}$C NMR (75 MHz, Chloroform-*d*) of compound **3t**

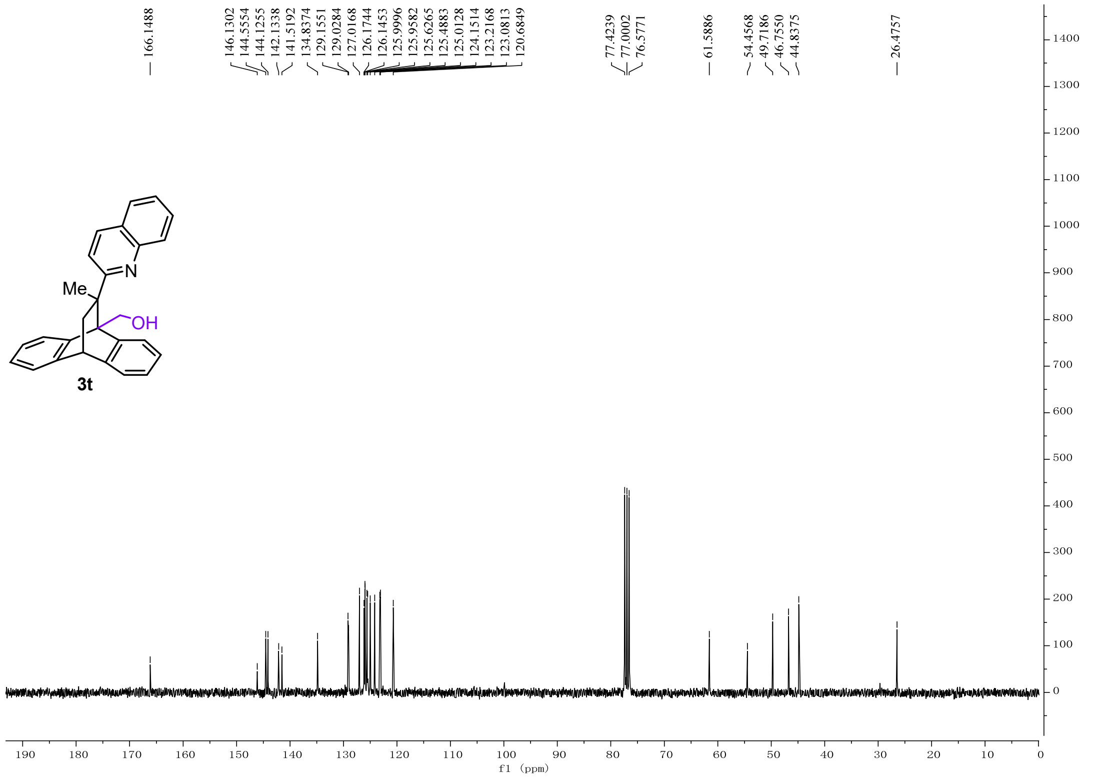


## $^{1}$H NMR (300 MHz, Chloroform-*d*) of compound **3u**

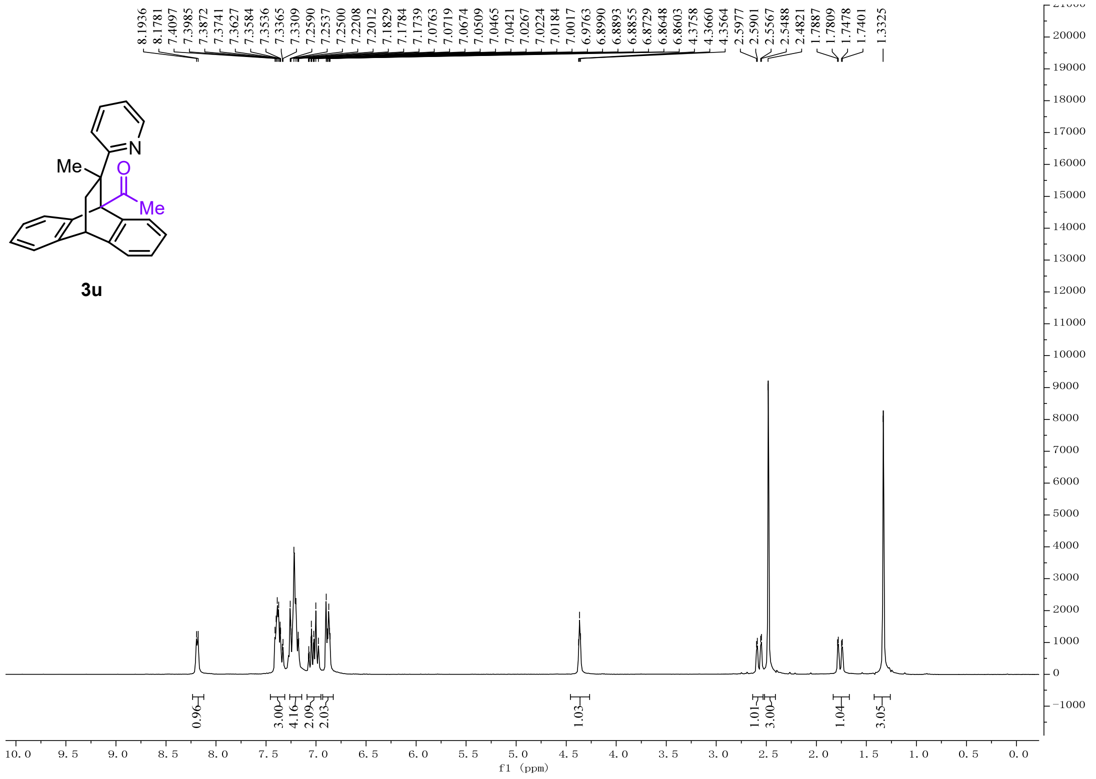

$^{13}$C NMR (75 MHz, Chloroform-*d*) of compound **3u**

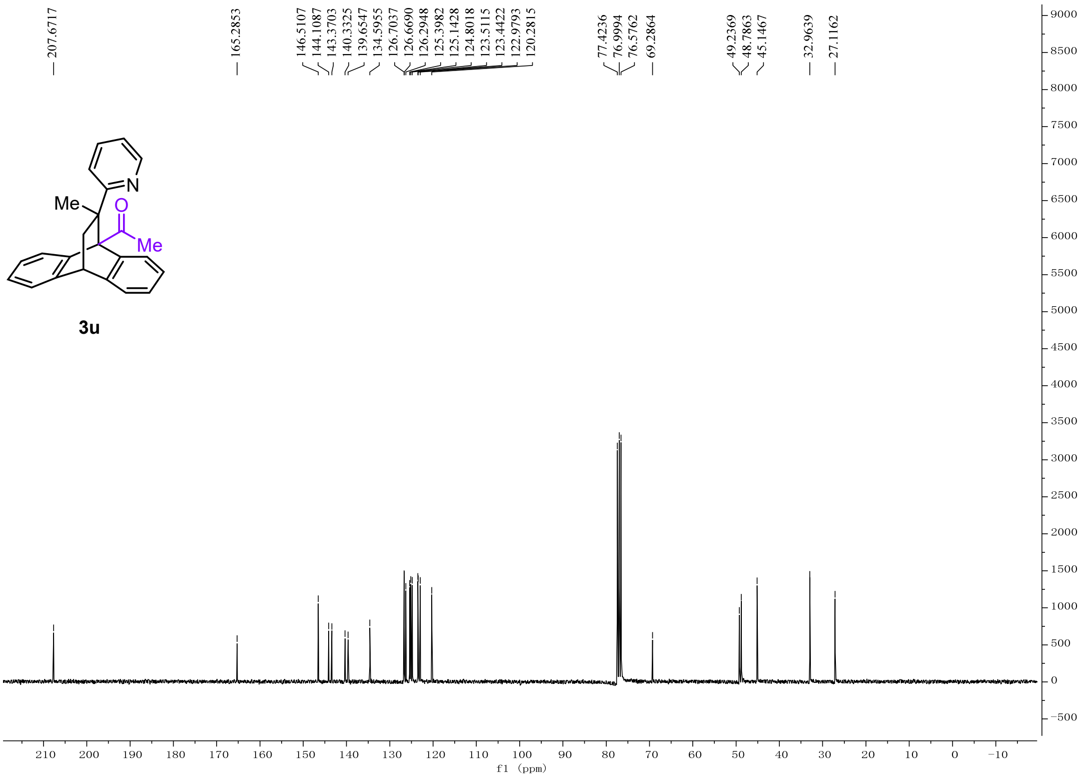


$^{1}$H NMR (300 MHz, Chloroform-*d*) of compound **3v**

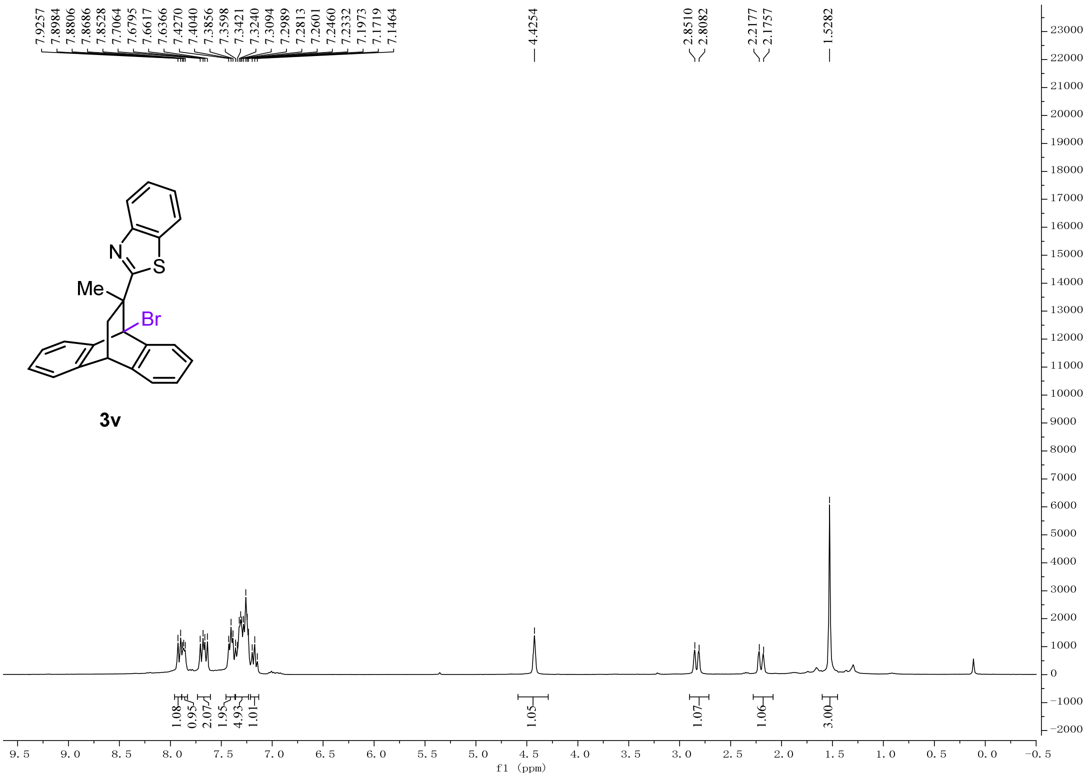

$^{13}$C NMR (75 MHz, Chloroform-*d*) of compound **3v**

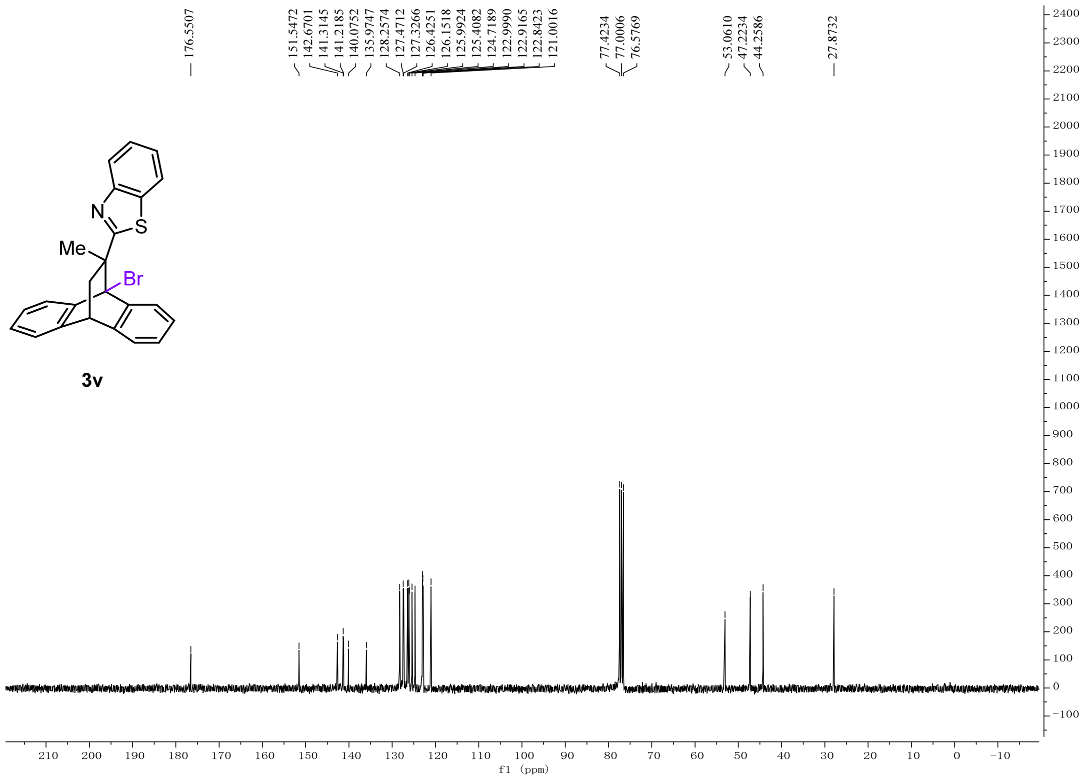


$^{1}$H NMR (300 MHz, Chloroform-*d*) of compound **3w**

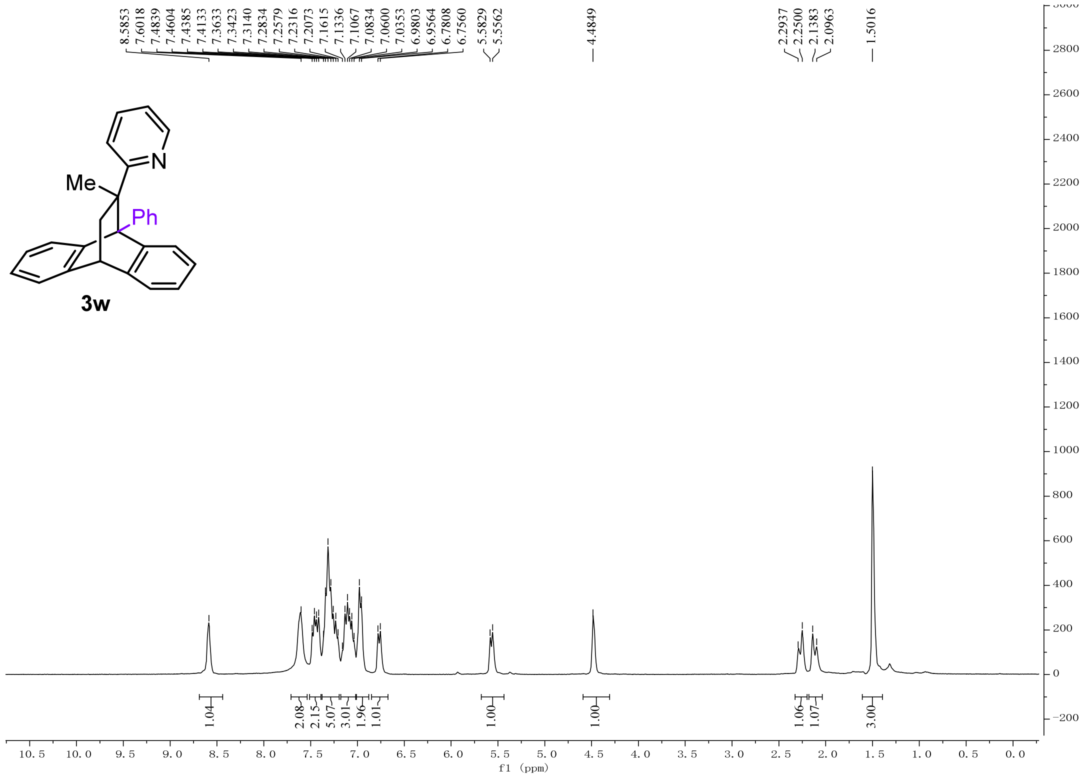

$^{13}$C NMR (75 MHz, Chloroform-*d*) of compound **3w**

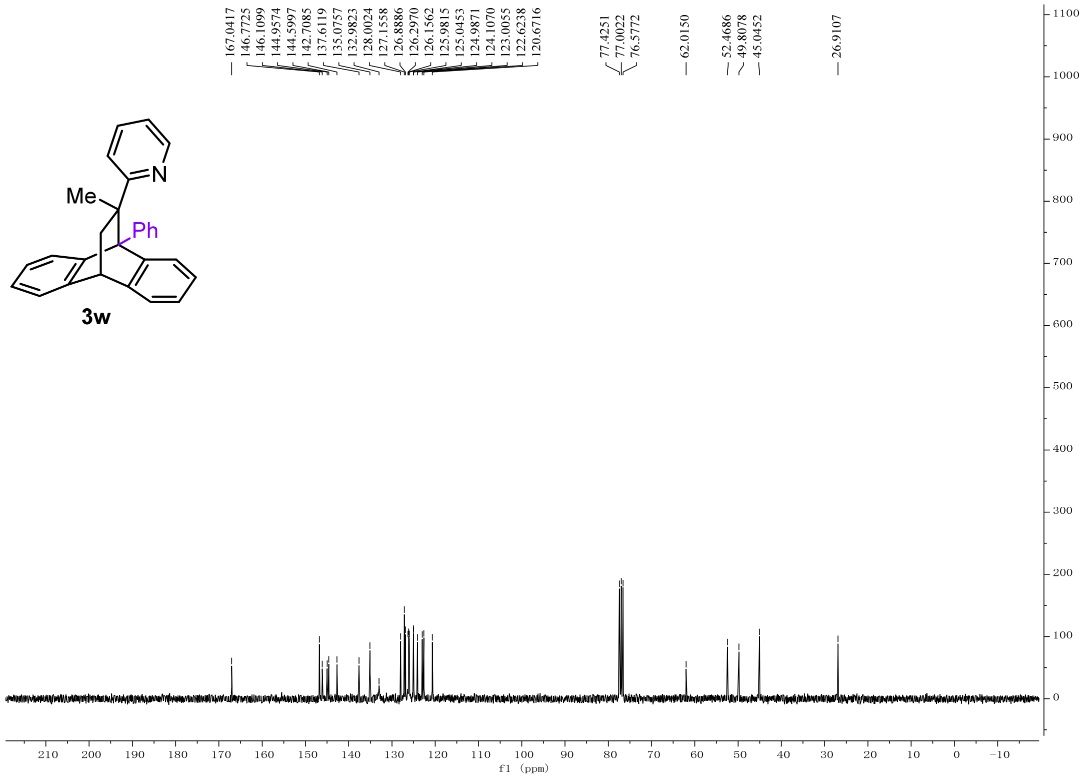


$^{1}$H NMR (300 MHz, Chloroform-*d*) of compound **3x**

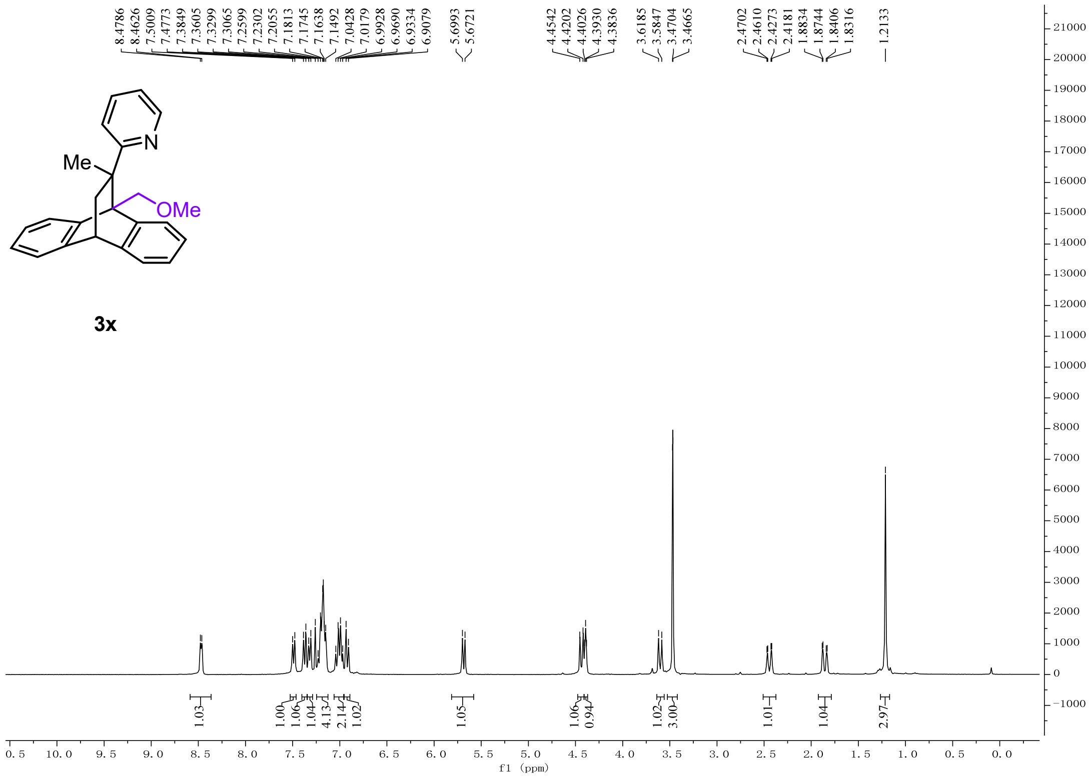

$^{13}C$ NMR (75 MHz, Chloroform-*d*) of compound **3x**

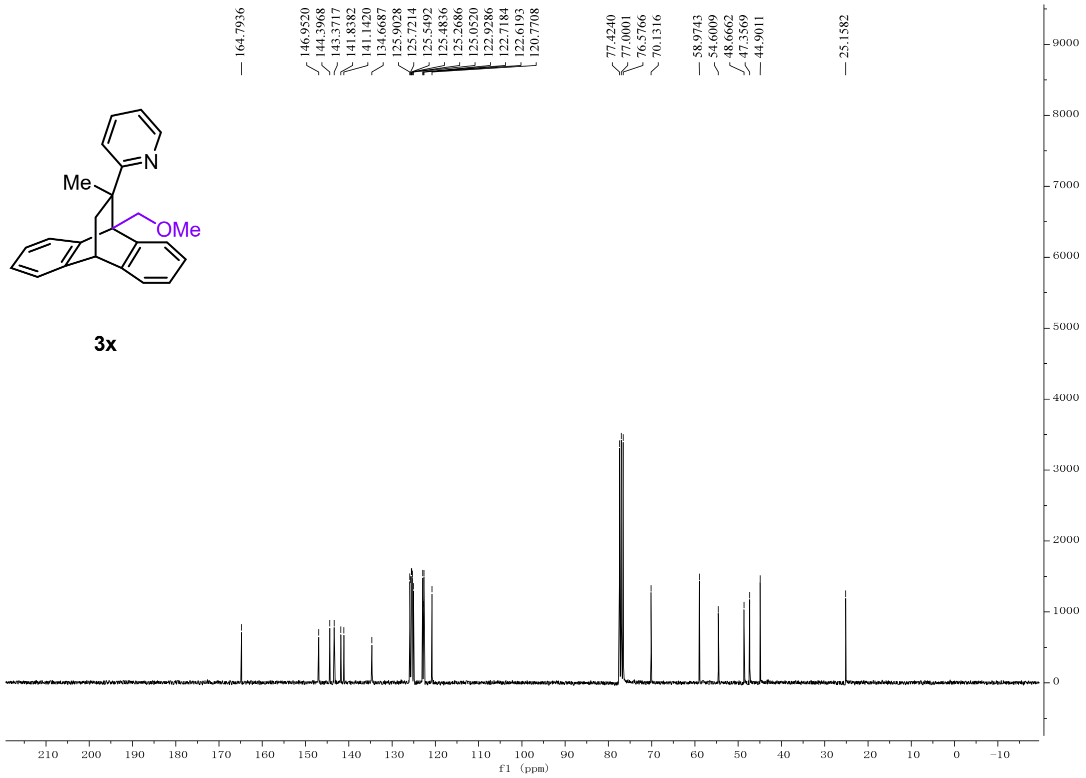


$^{1}H$ NMR (300 MHz, Chloroform-*d*) of compound **3y**

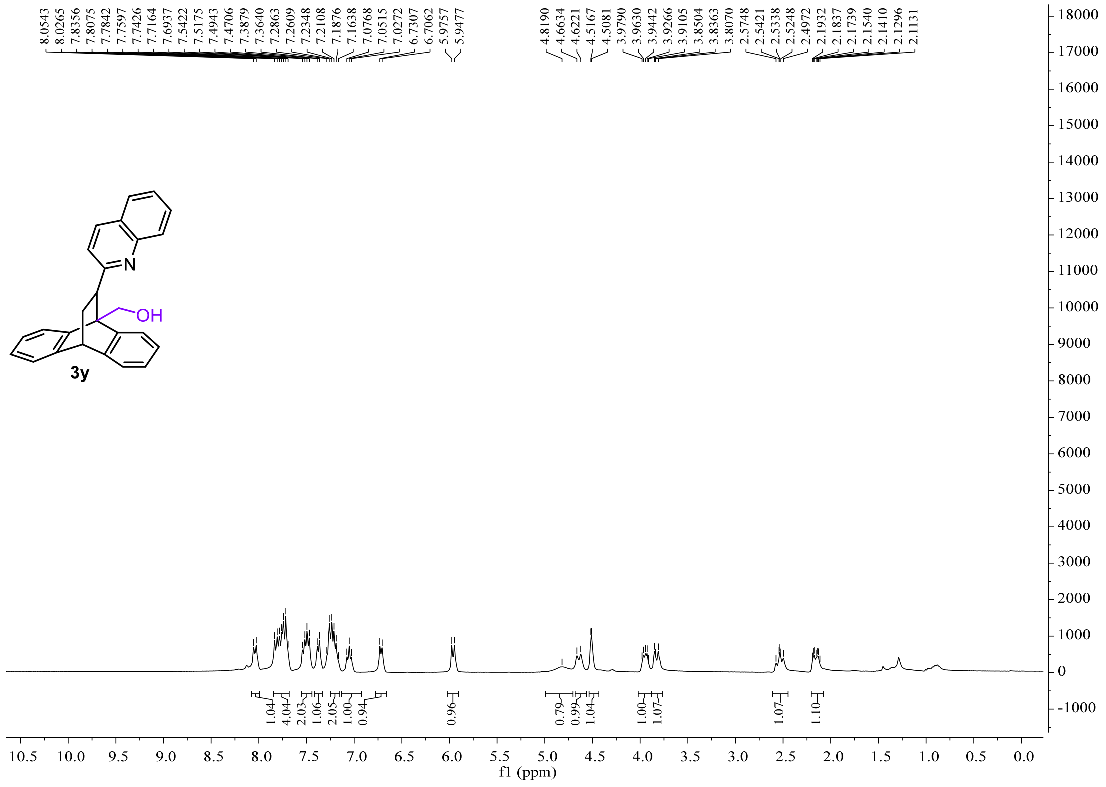

$^{13}C$ NMR (75 MHz, Chloroform-*d*) of compound **3y**

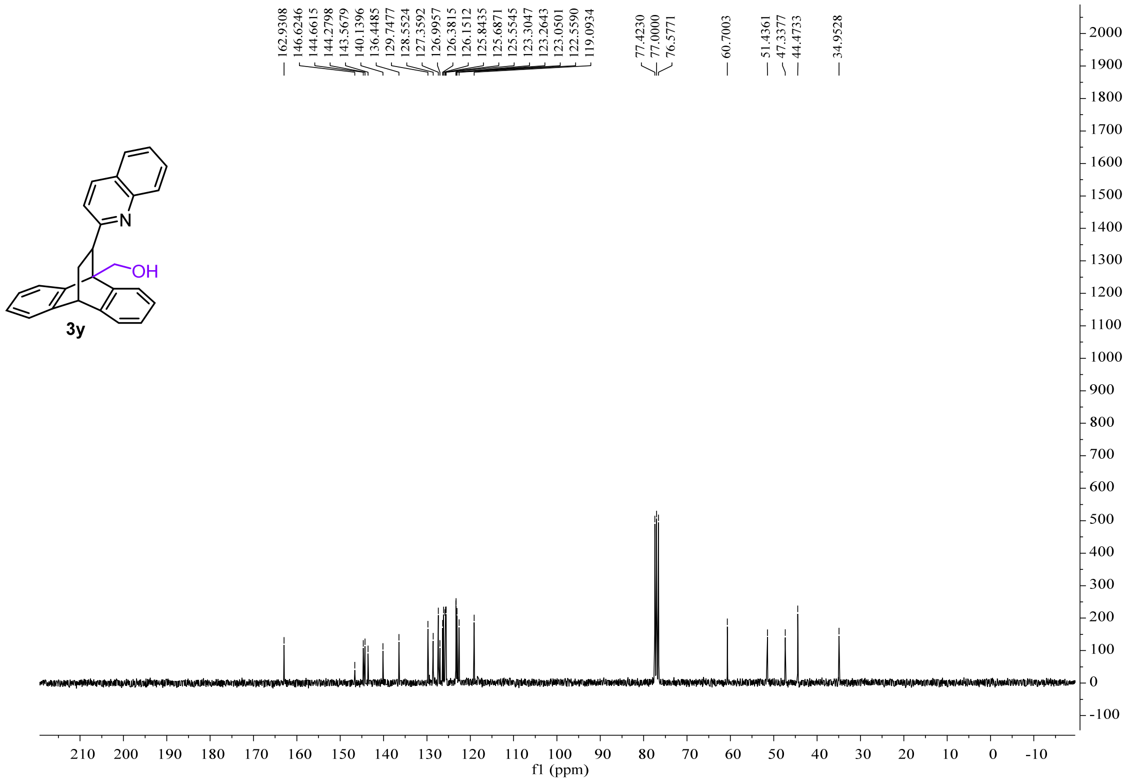


$^{1}H$ NMR (300 MHz, Chloroform-*d*) of compound **3za**

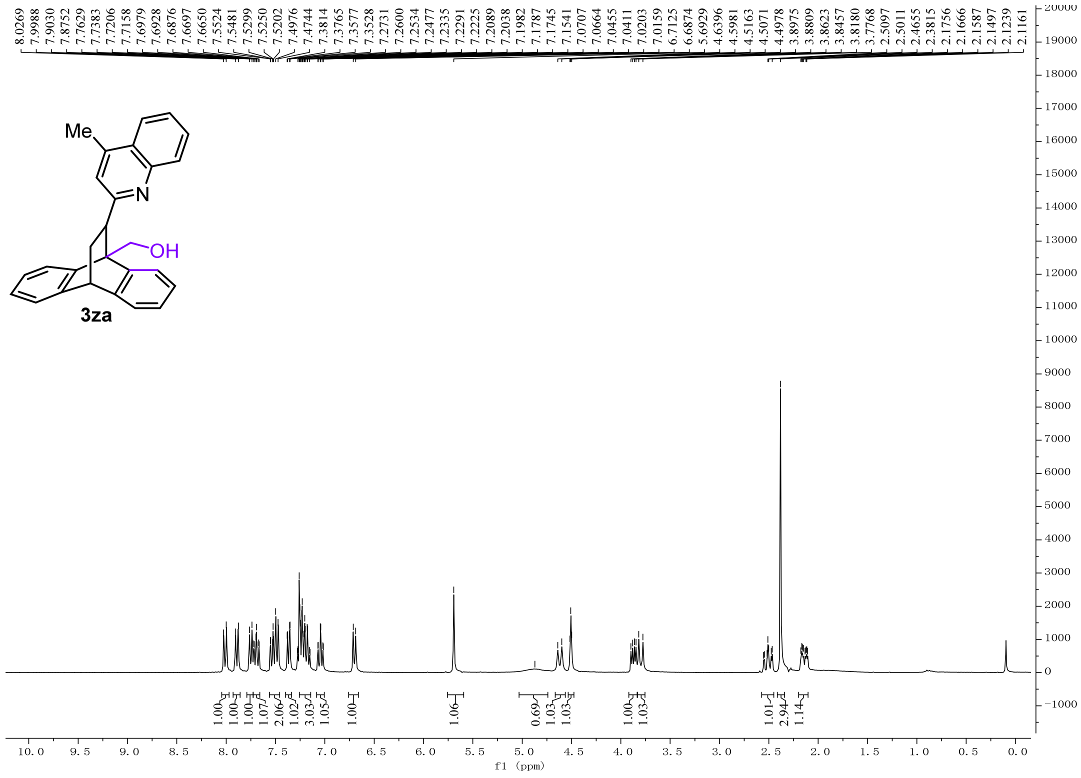

$^{13}$C NMR (75 MHz, Chloroform-*d*) of compound **3za**

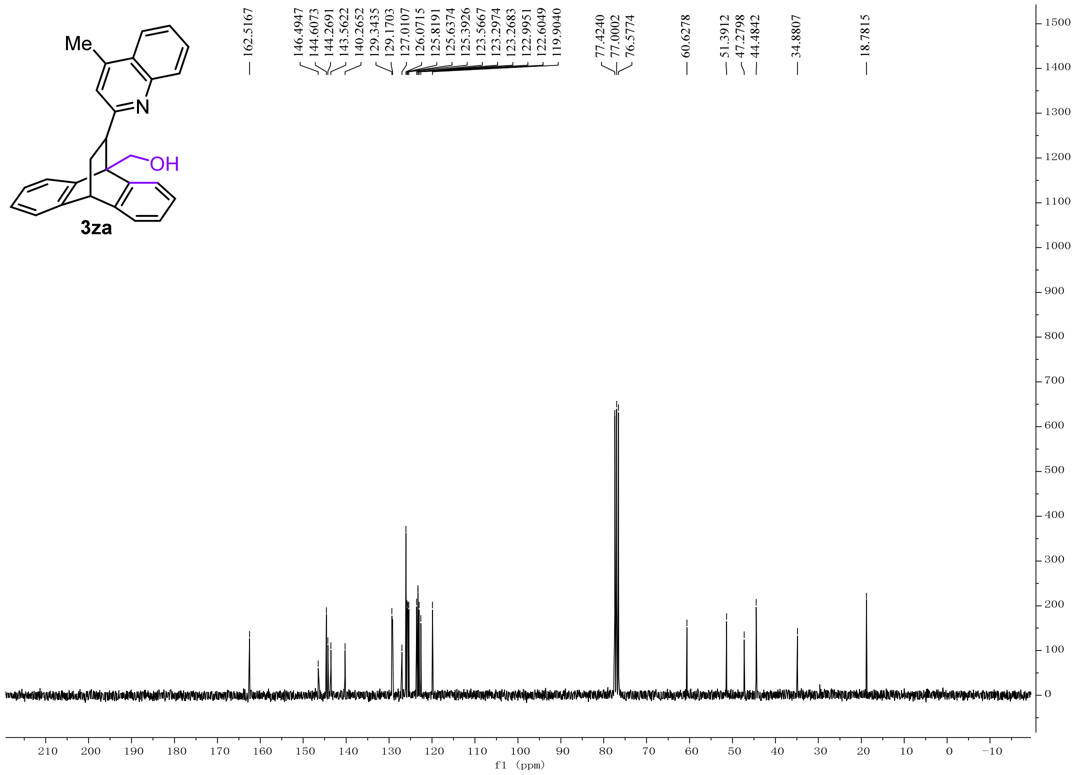

$^{1}$H NMR (300 MHz, Chloroform-*d*) of compound **3zb**

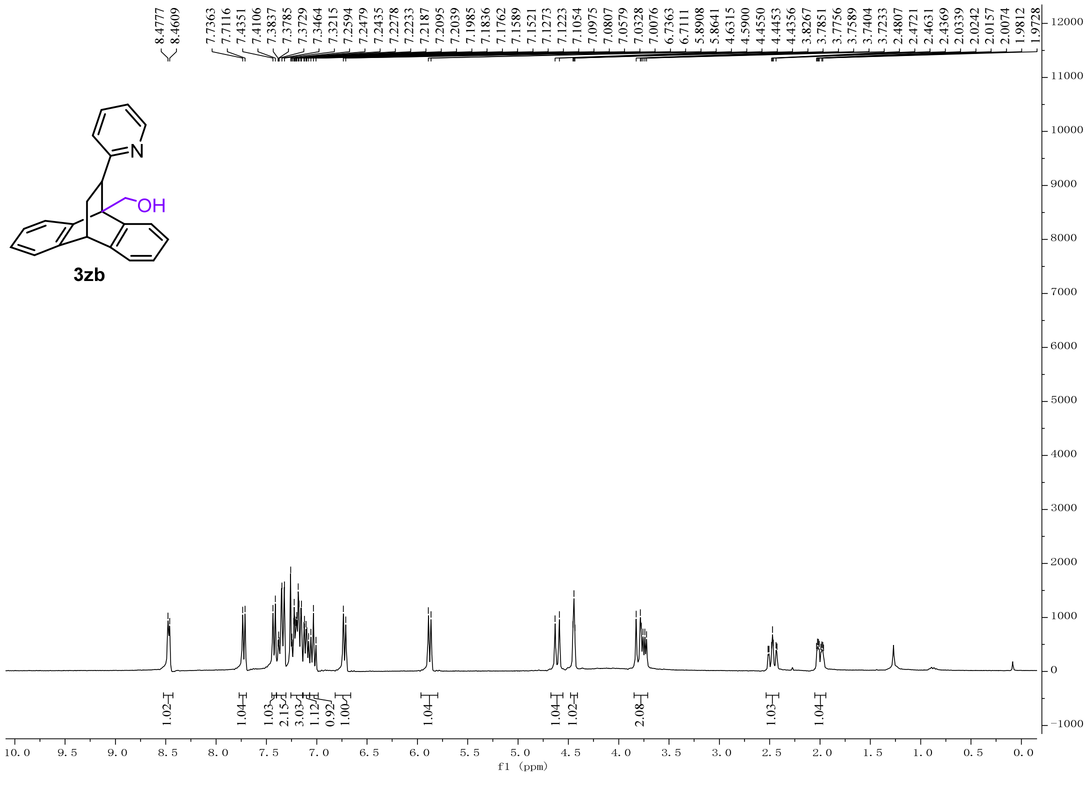

$^{13}C$ NMR (75 MHz, Chloroform-*d*) of compound **3zb**

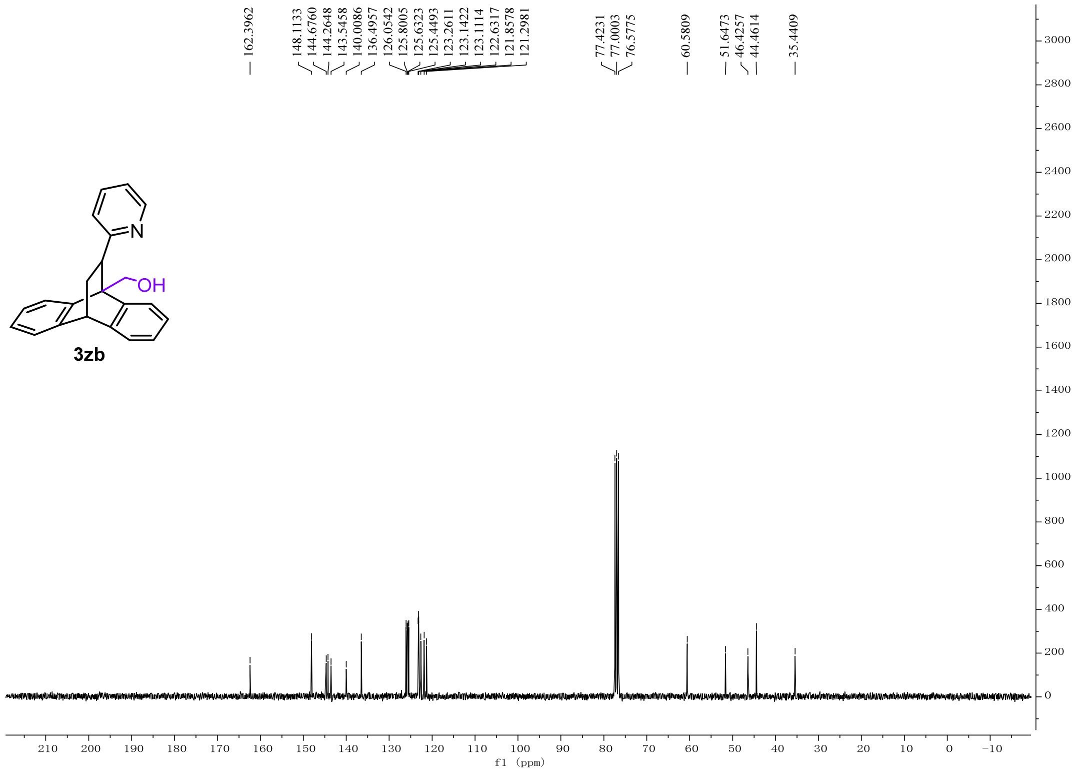


$^{1}H$ NMR (300 MHz, Chloroform-*d*) of compound **3zc**

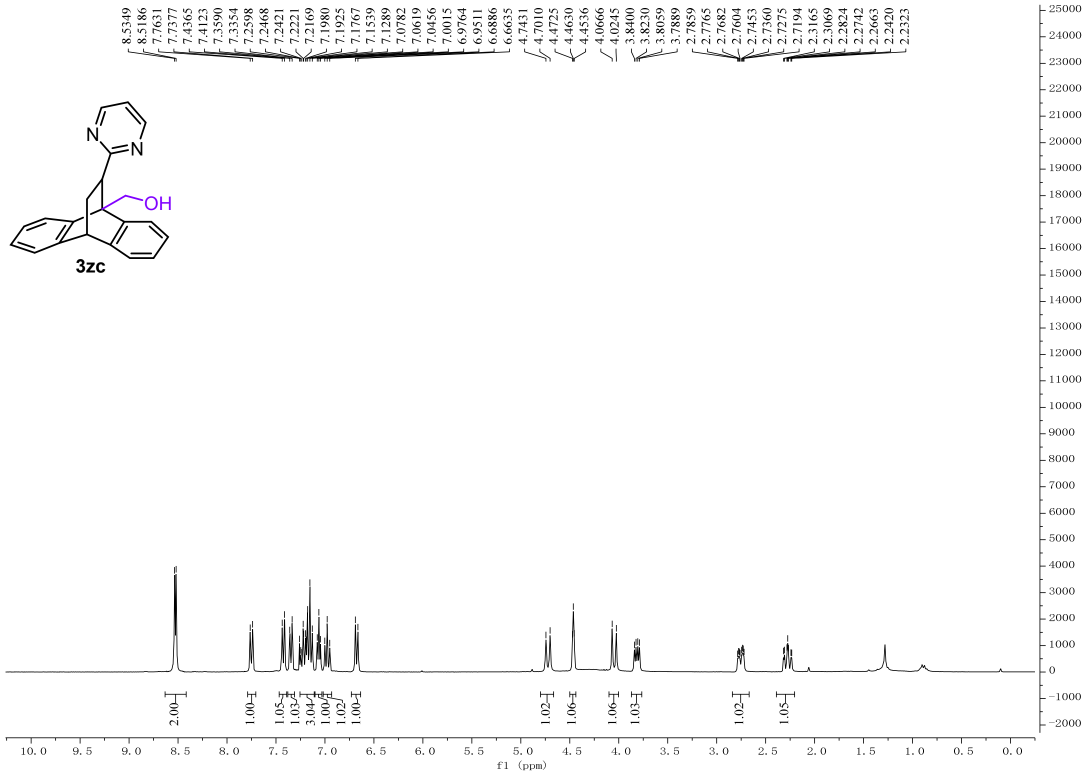

$^{13}C$ NMR (75 MHz, Chloroform-*d*) of compound **3zc**

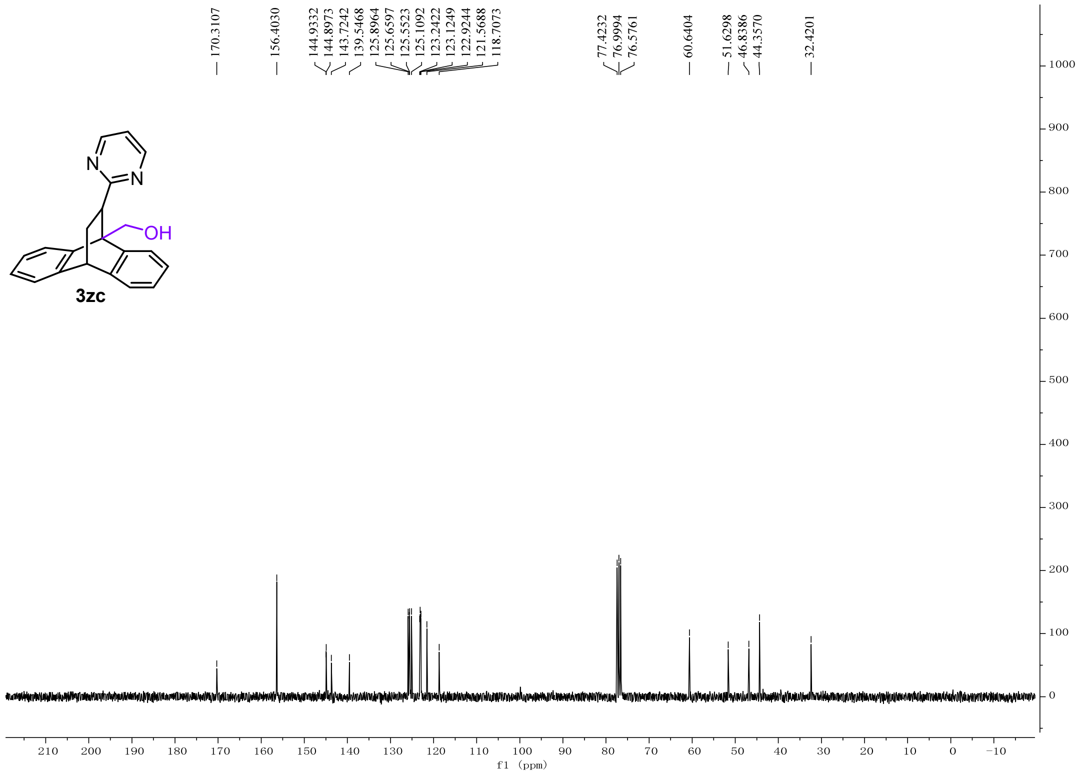


$^{1}H$ NMR (300 MHz, Chloroform-*d*) of compound **3zd**

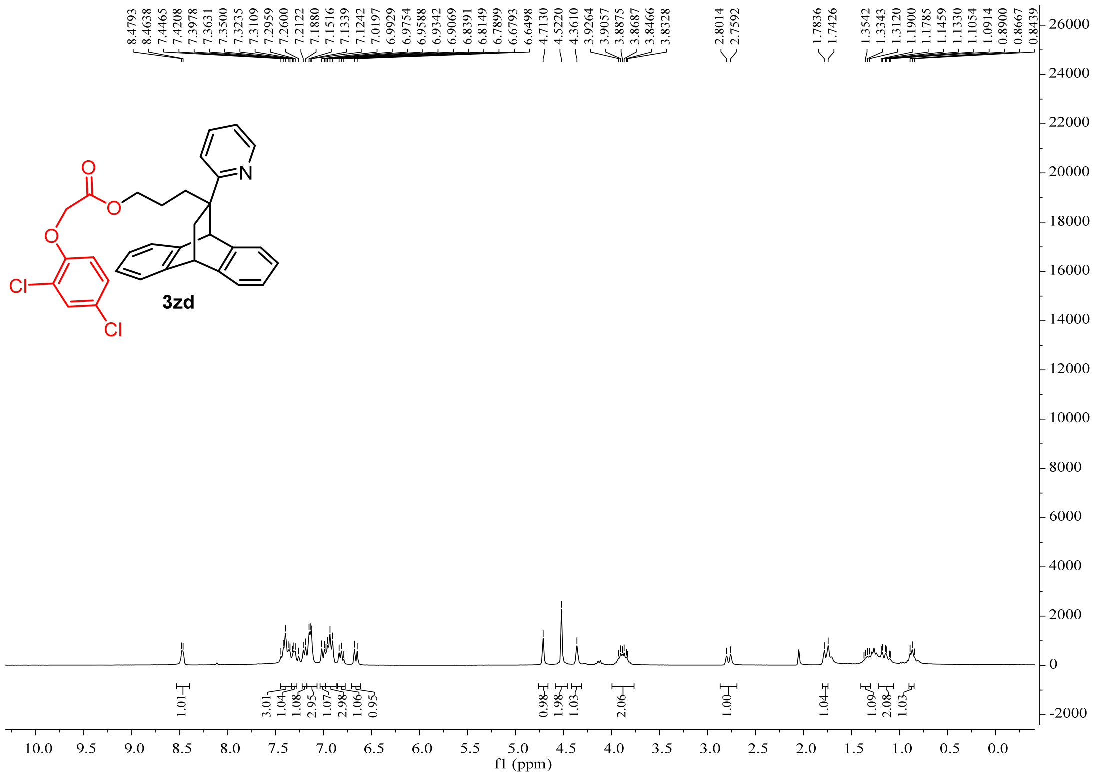

$^{13}$C NMR (75 MHz, Chloroform-*d*) of compound **3zd**

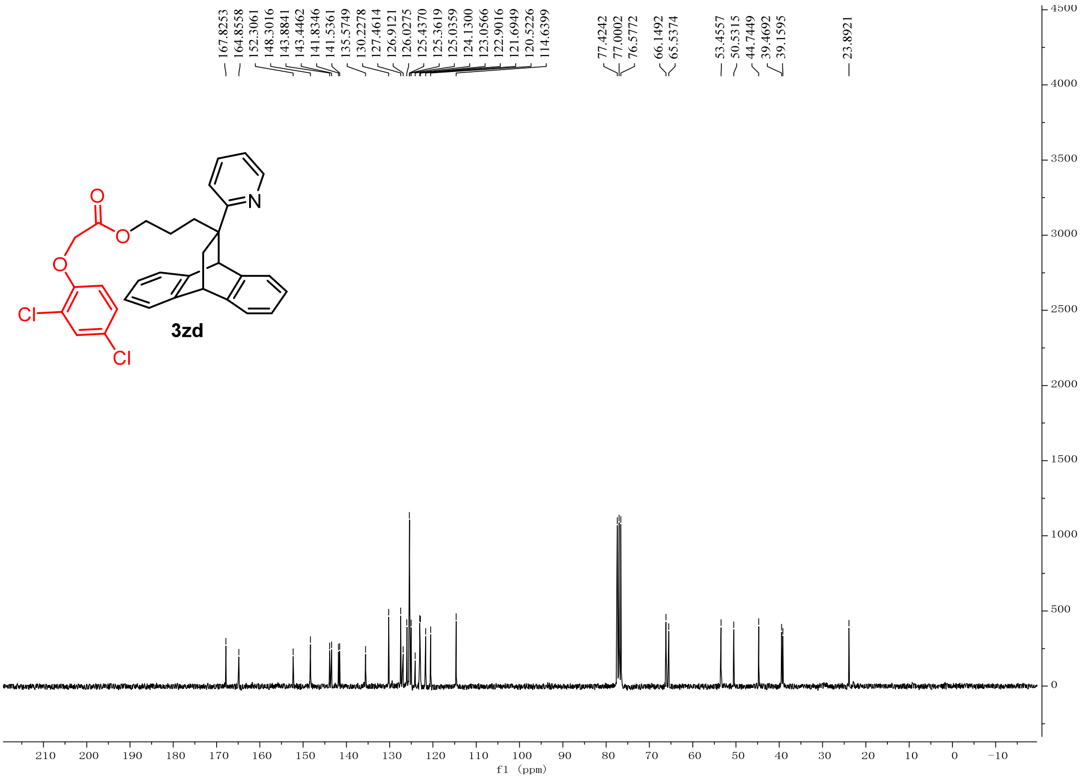


$^{1}$H NMR (300 MHz, Chloroform-*d*) of compound **3ze**

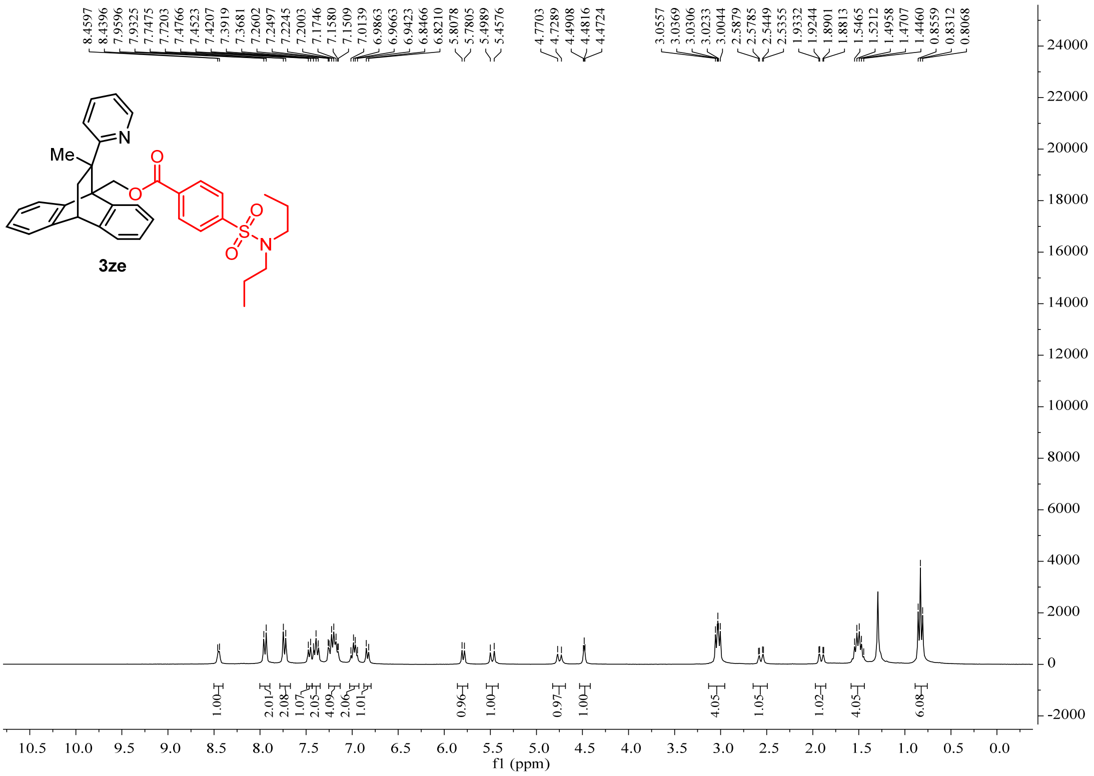

$^{13}$C NMR (75 MHz, Chloroform-*d*) of compound **3ze**

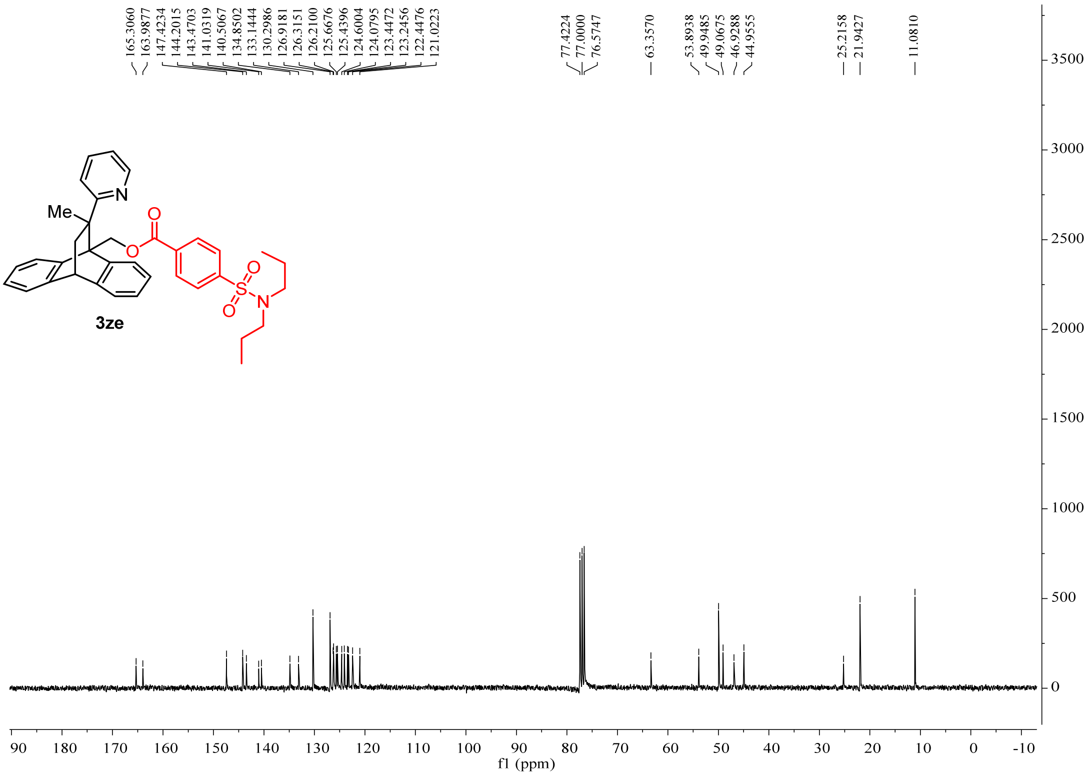


$^{1}$H NMR (300 MHz, Chloroform-*d*) of compound **3zf**

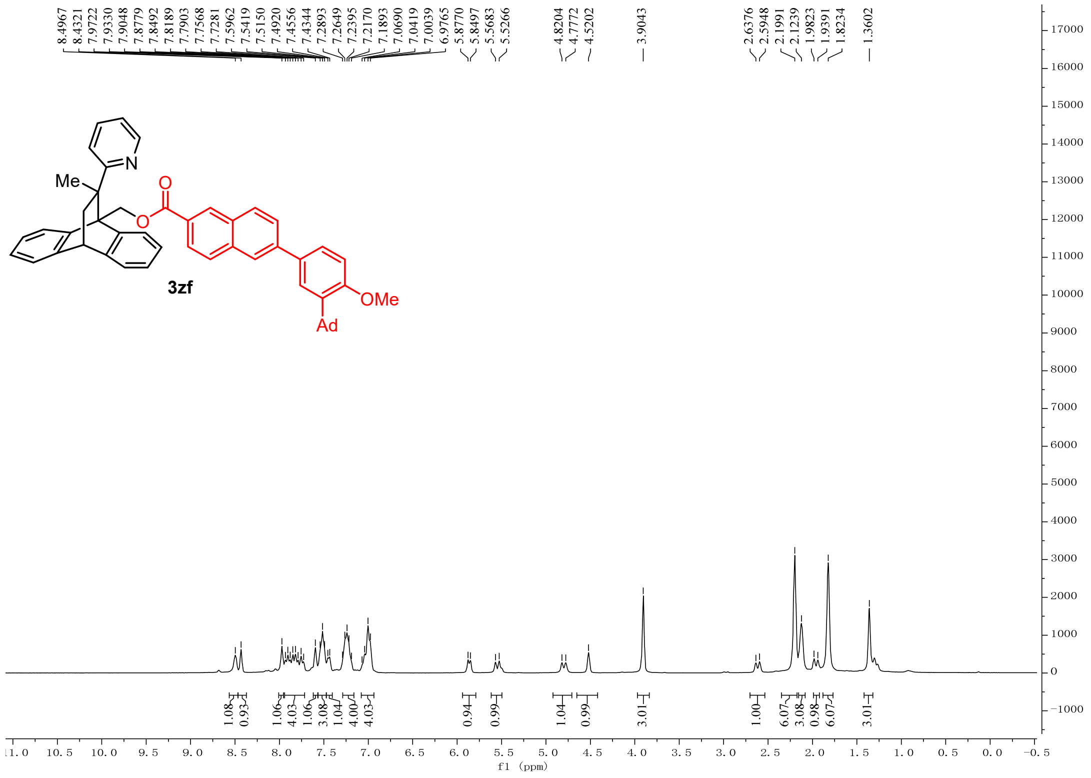

$^{13}C$ NMR (75 MHz, Chloroform-*d*) of compound **3zf**

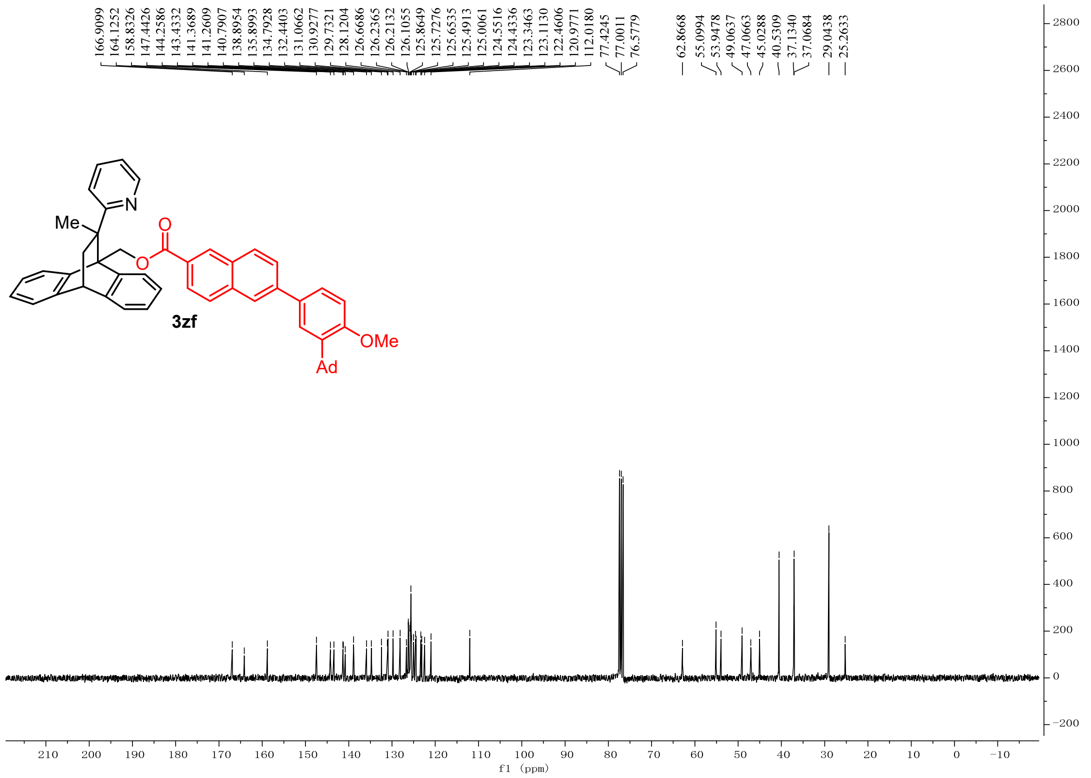


$^{1}H$ NMR (300 MHz, Chloroform-*d*) of compound **3zg**

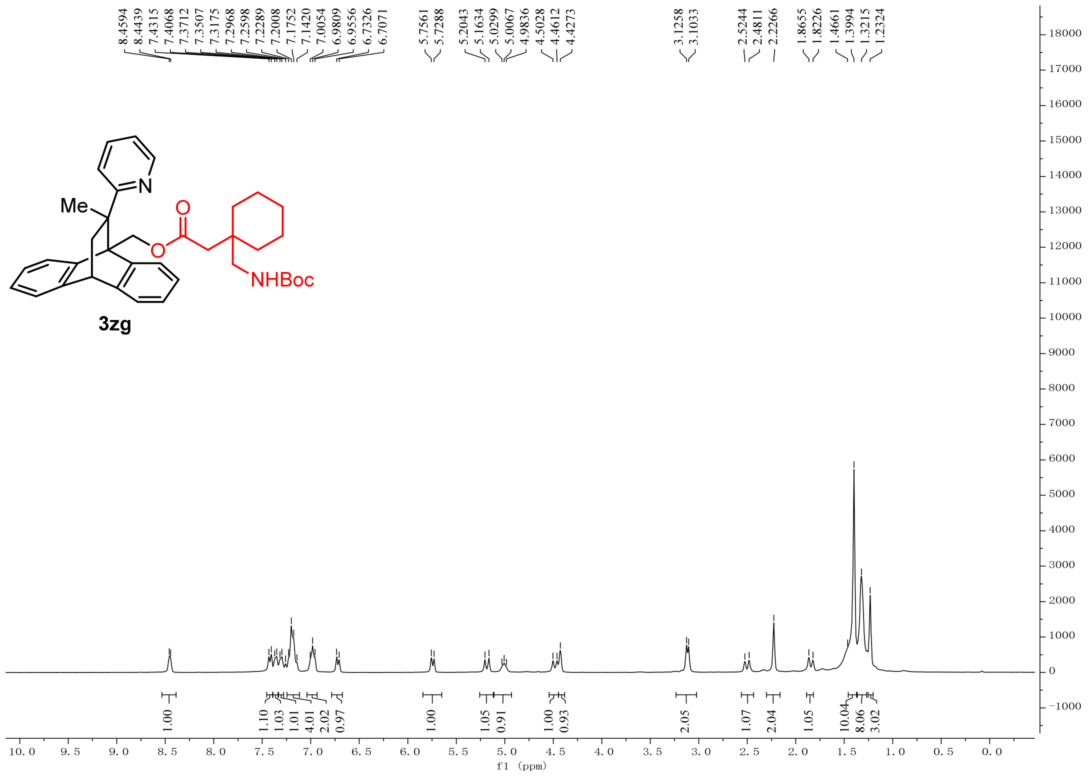

$^{13}$C NMR (75 MHz, Chloroform-*d*) of compound **3zg**

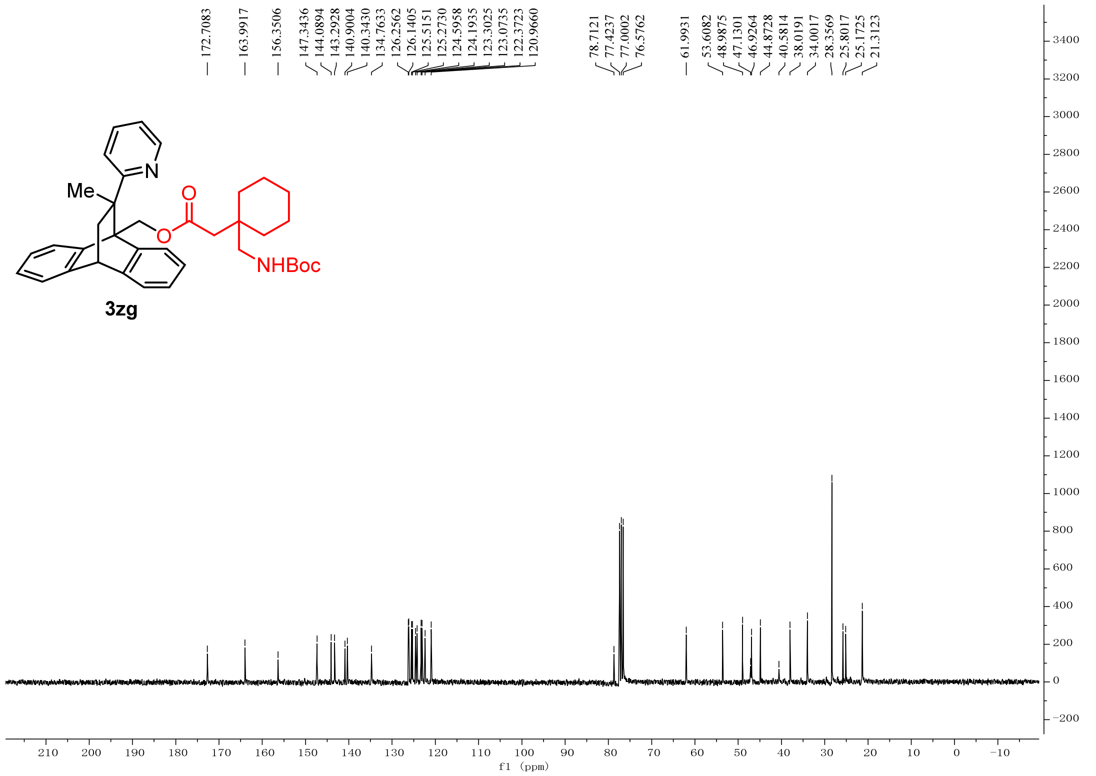


$^{1}$H NMR (300 MHz, Chloroform-*d*) of compound **5a**

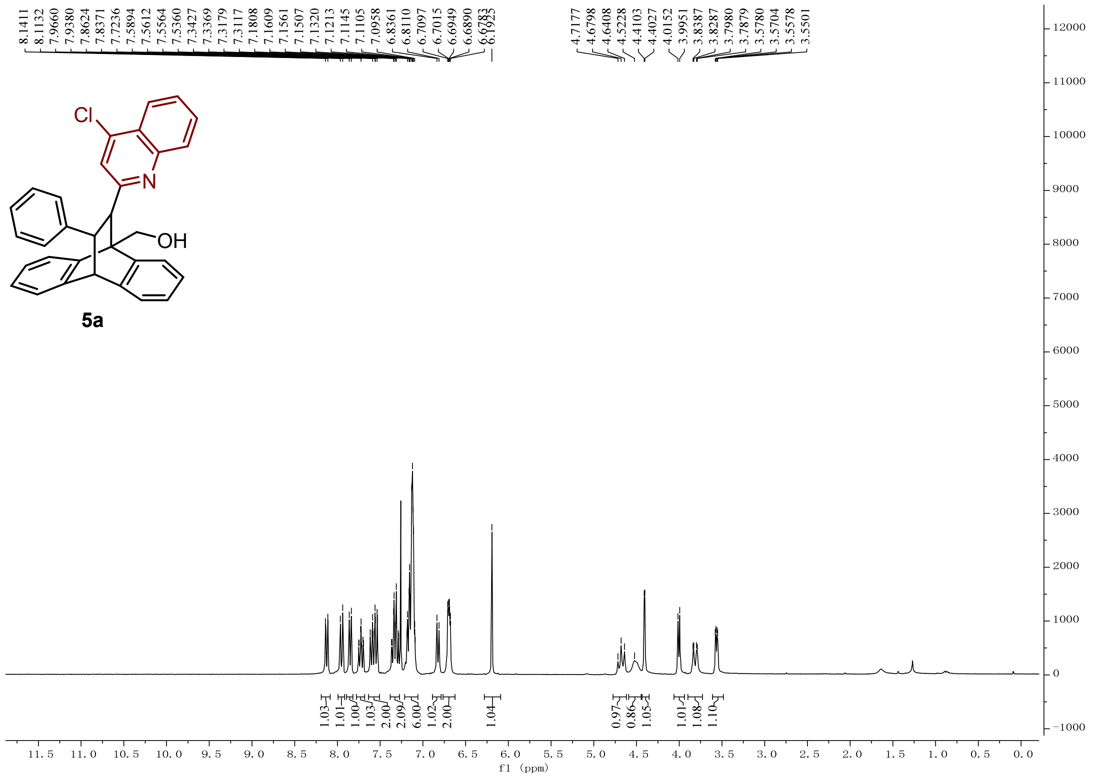

$^{13}$C NMR (75 MHz, Chloroform-*d*) of compound **5a**

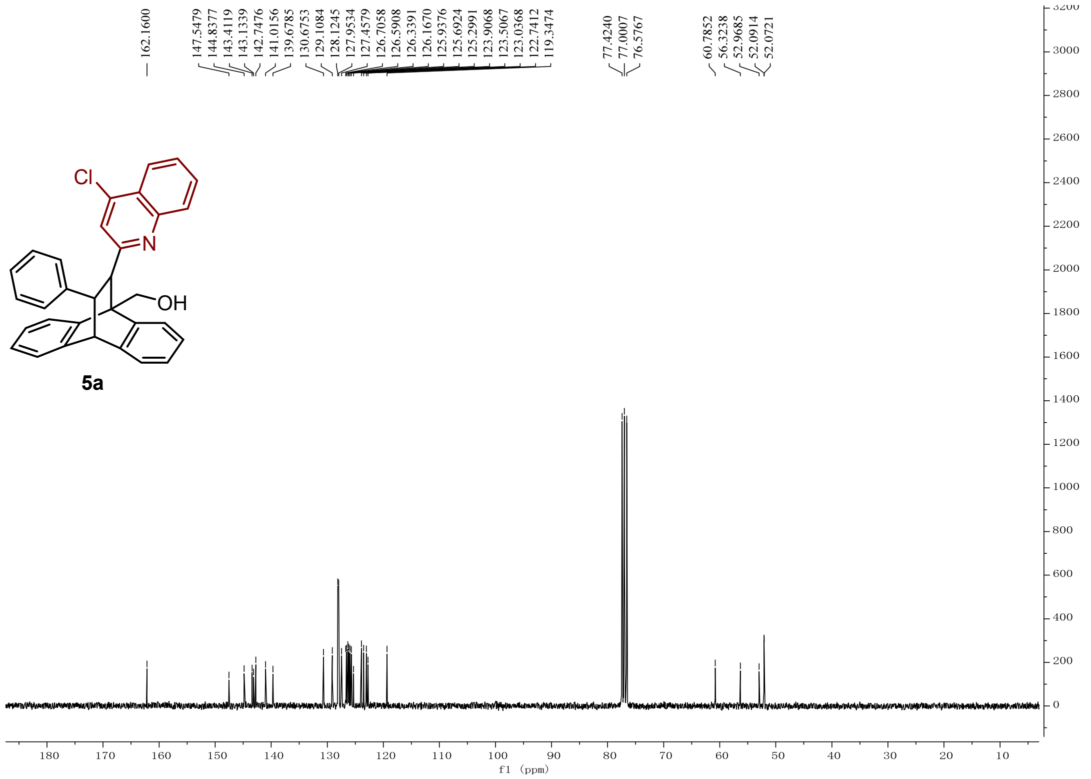


$^{1}$H NMR (300 MHz, Chloroform-*d*) of compound **5b**

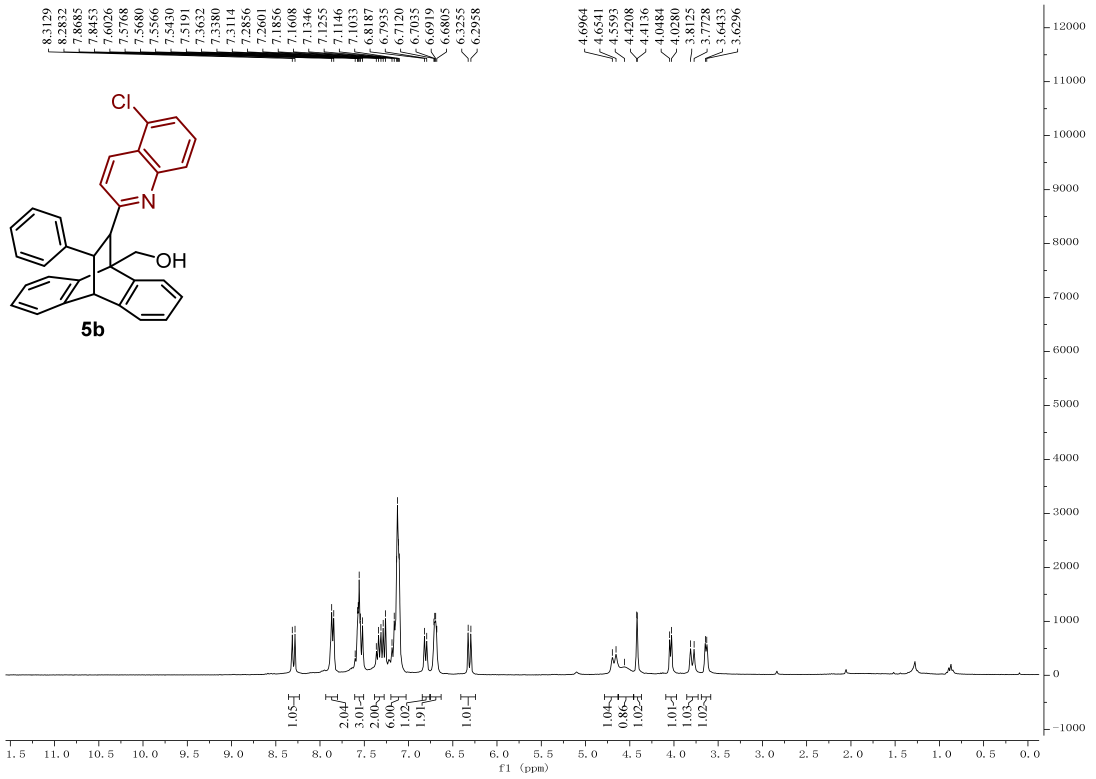

$^{13}$C NMR (75 MHz, Chloroform-*d*) of compound **5b**

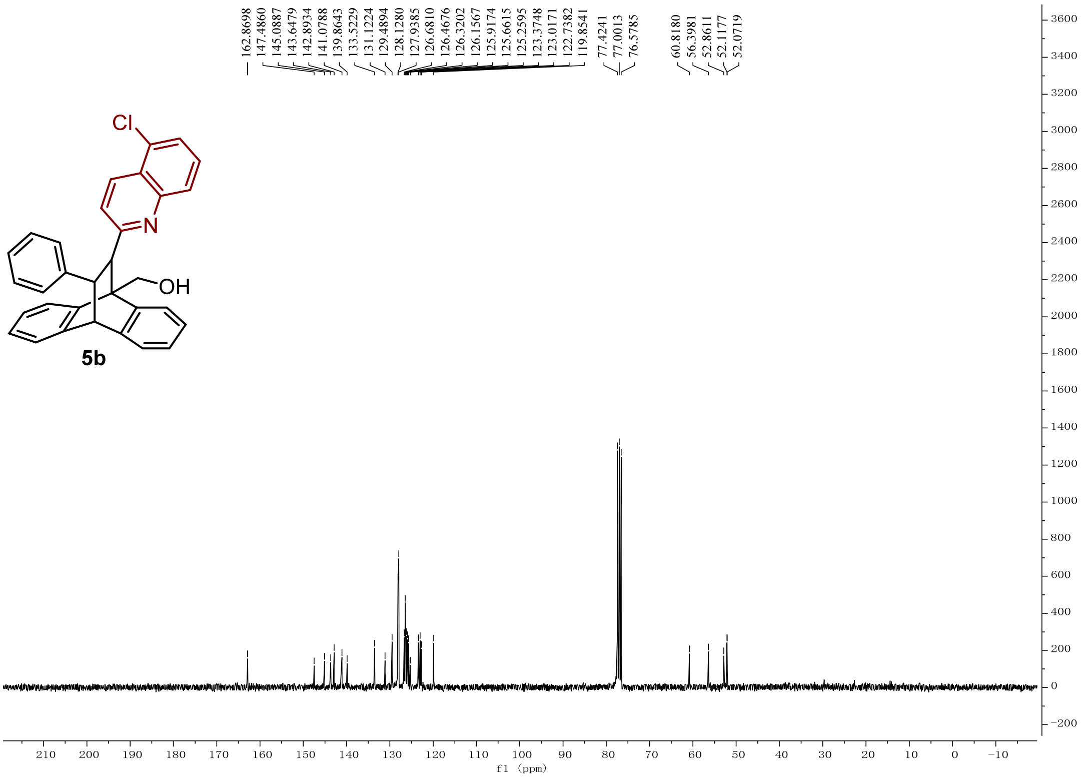


$^{1}$H NMR (300 MHz, Chloroform-*d*) of compound **5c**

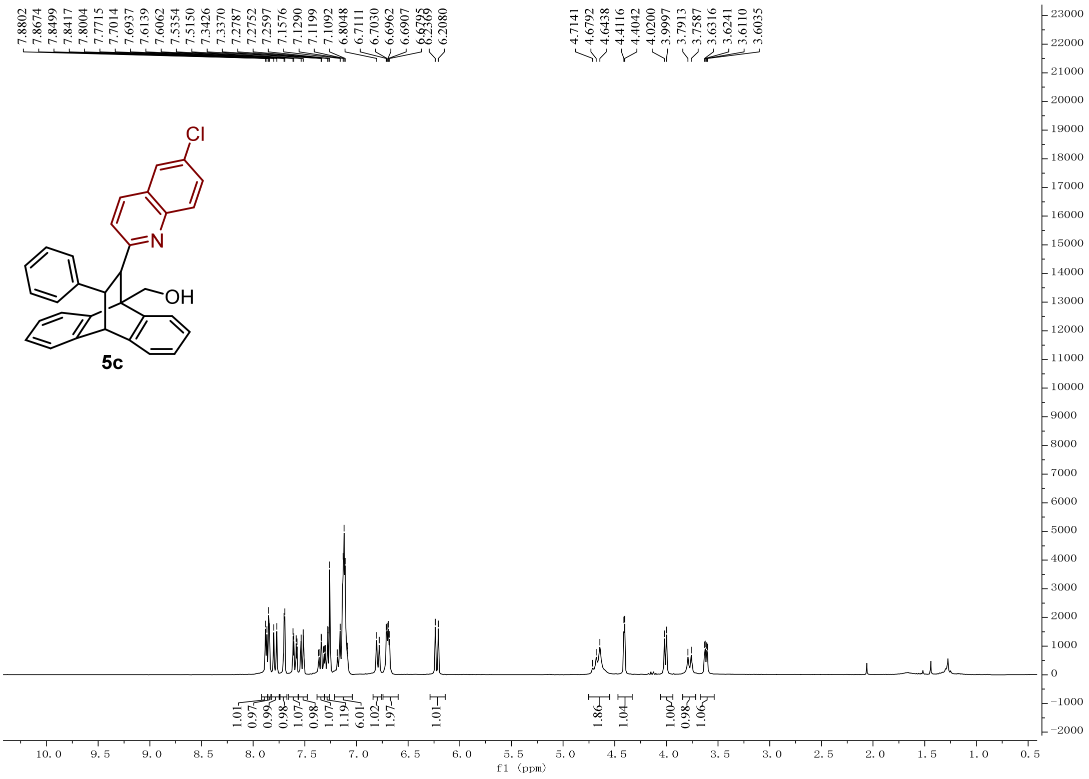

$^{13}$C NMR (75 MHz, Chloroform-*d*) of compound **5c**

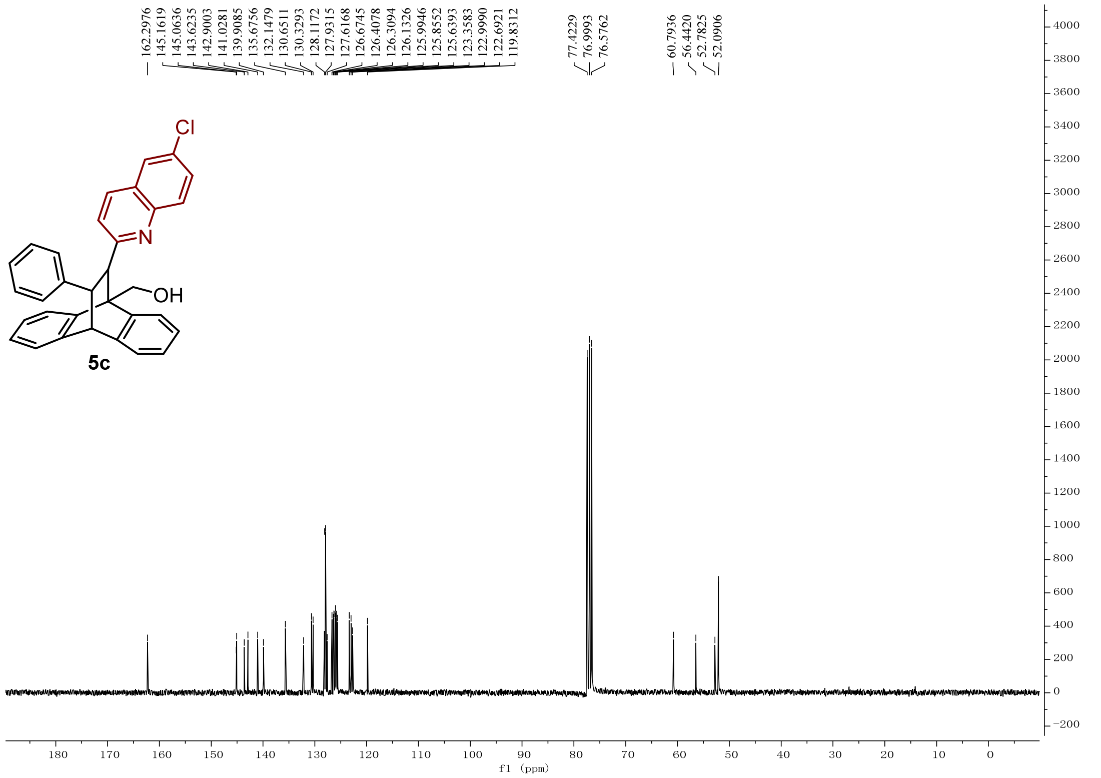


$^{1}$H NMR (300 MHz, Chloroform-*d*) of compound **5d**

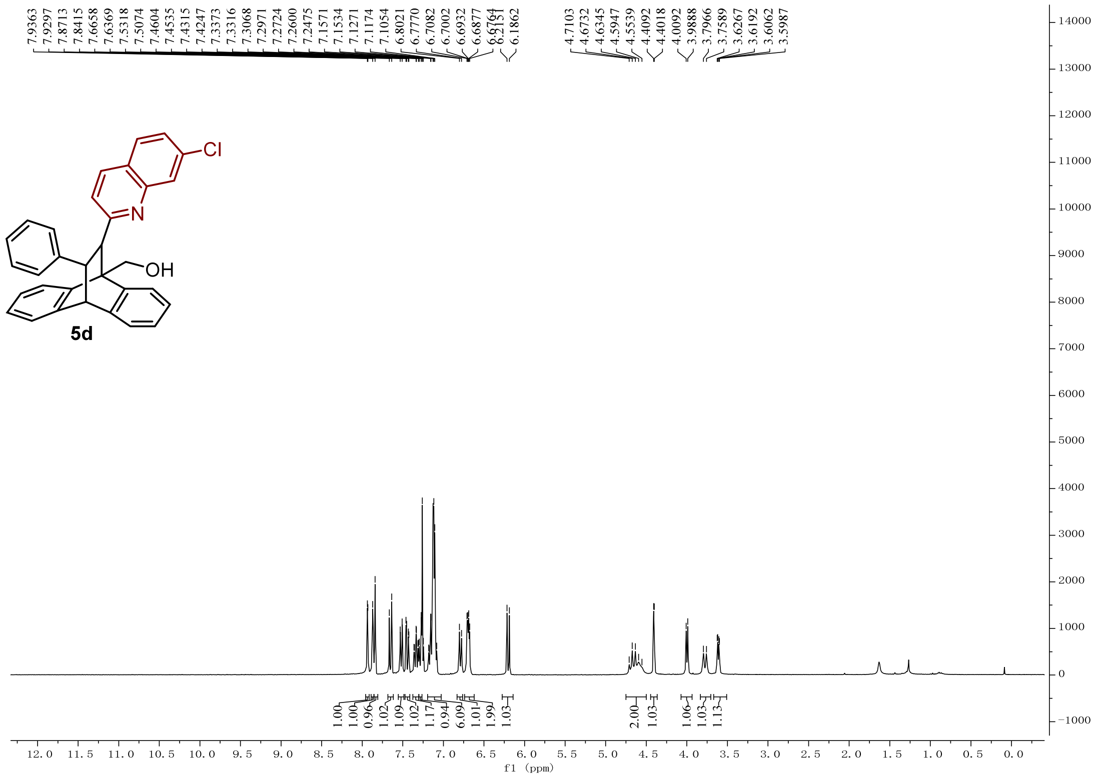

$^{13}$C NMR (75 MHz, Chloroform-*d*) of compound **5d**

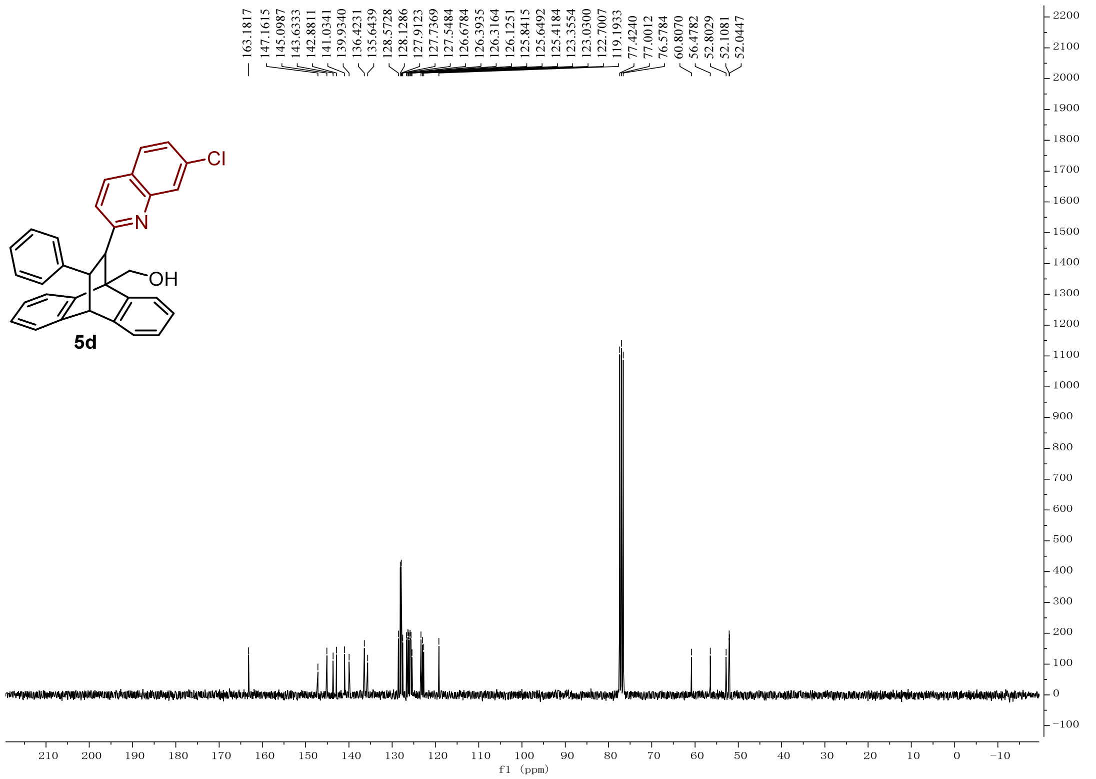


$^{1}$H NMR (300 MHz, Chloroform-*d*) of compound **5e**

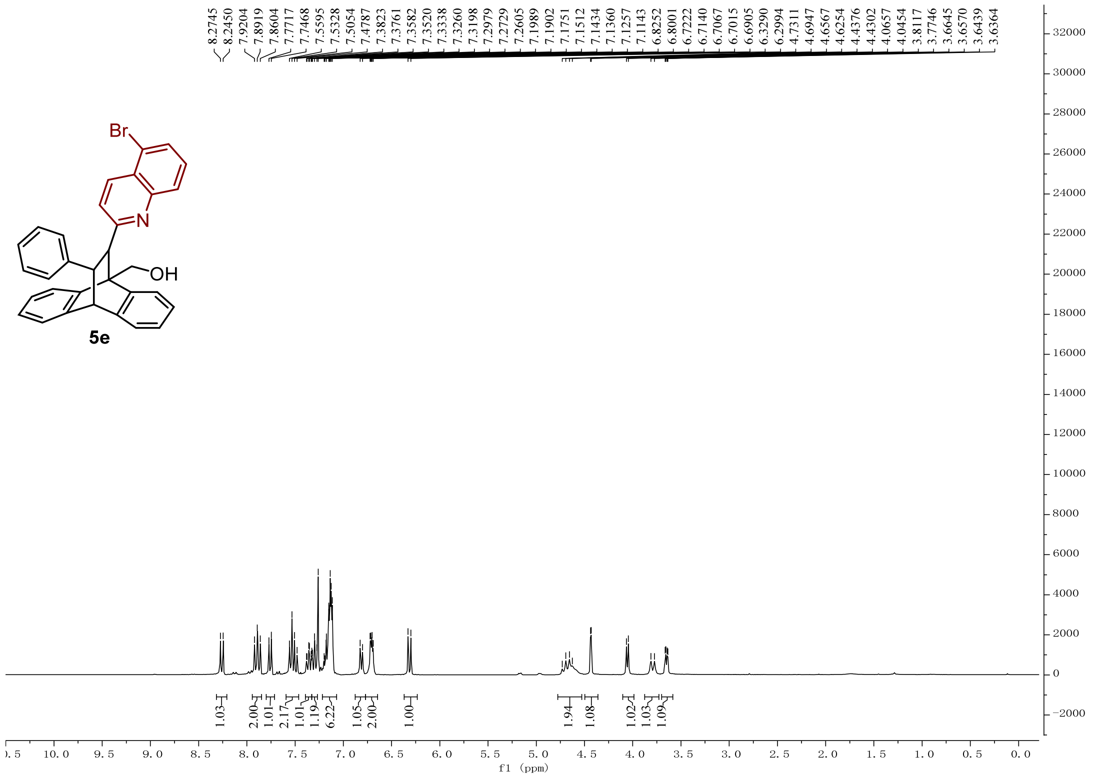

$^{13}$C NMR (75 MHz, Chloroform-*d*) of compound **5e**

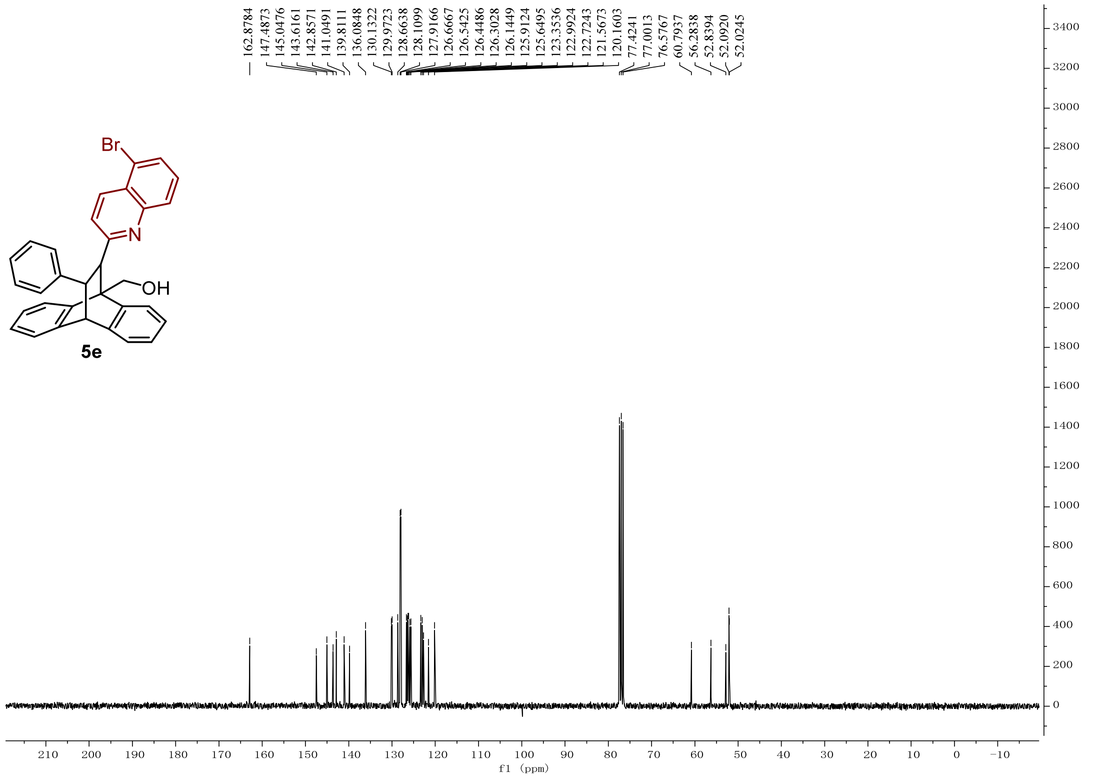

$^{1}$H NMR (300 MHz, Chloroform-*d*) of compound **5f**

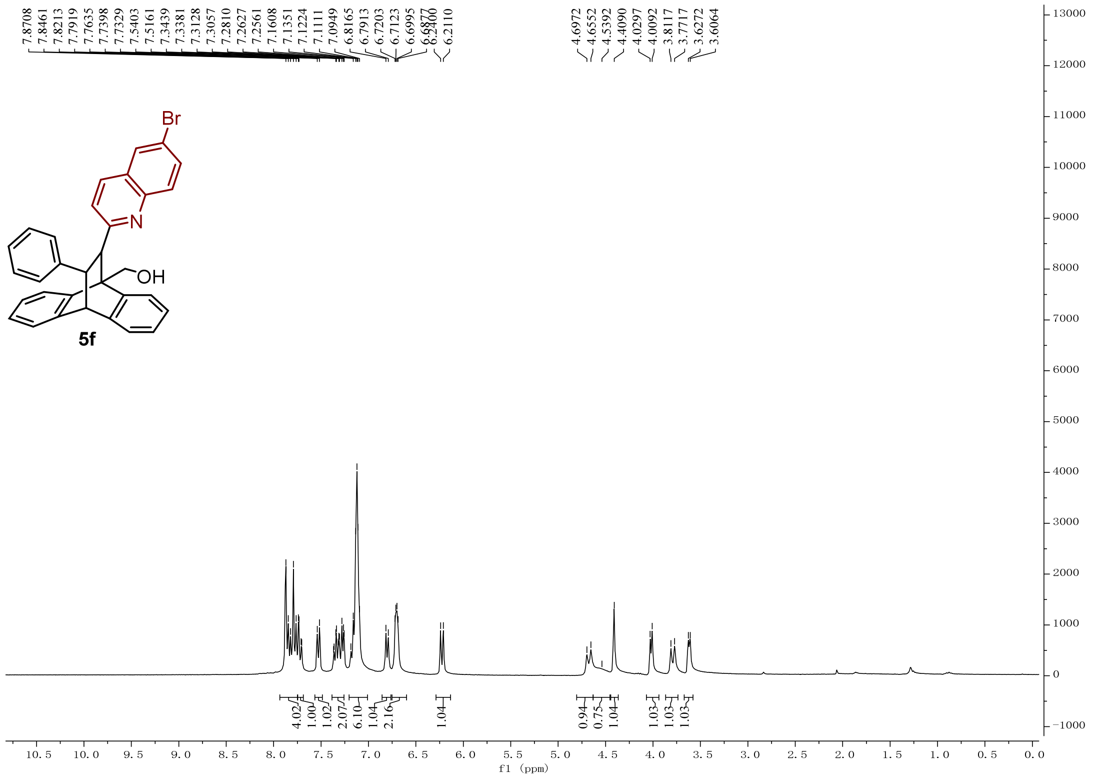

$^{13}$C NMR (75 MHz, Chloroform-*d*) of compound **5f**

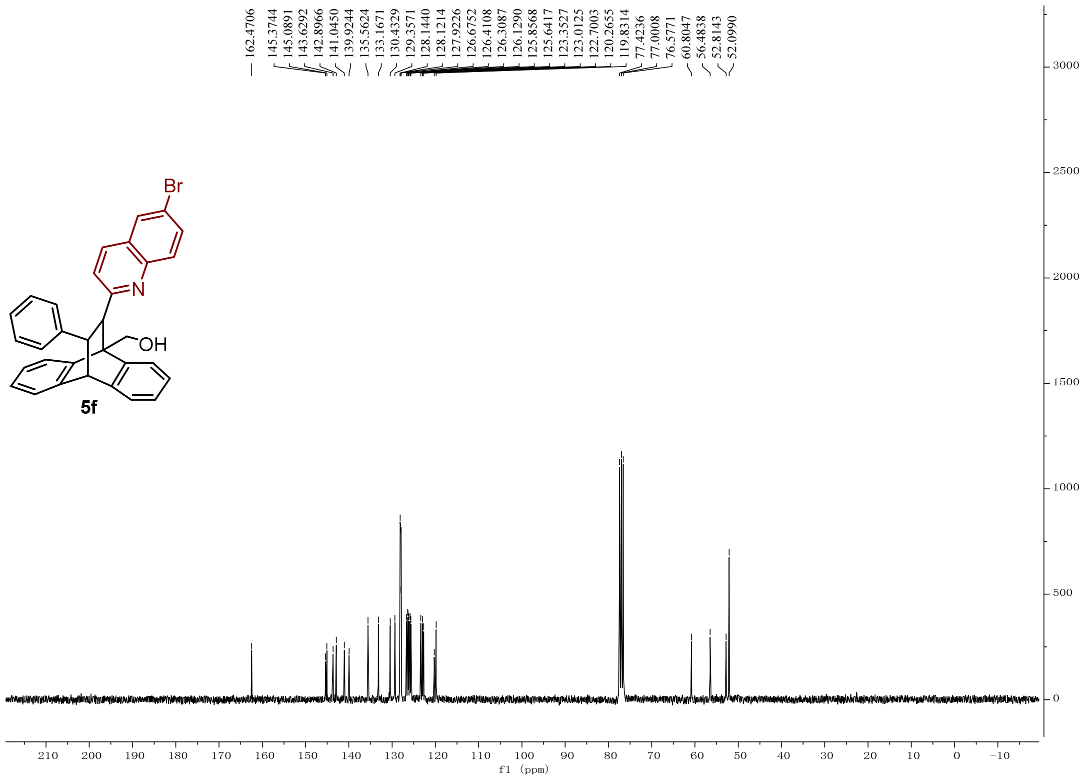


$^{1}$H NMR (300 MHz, Chloroform-*d*) of compound **5g**

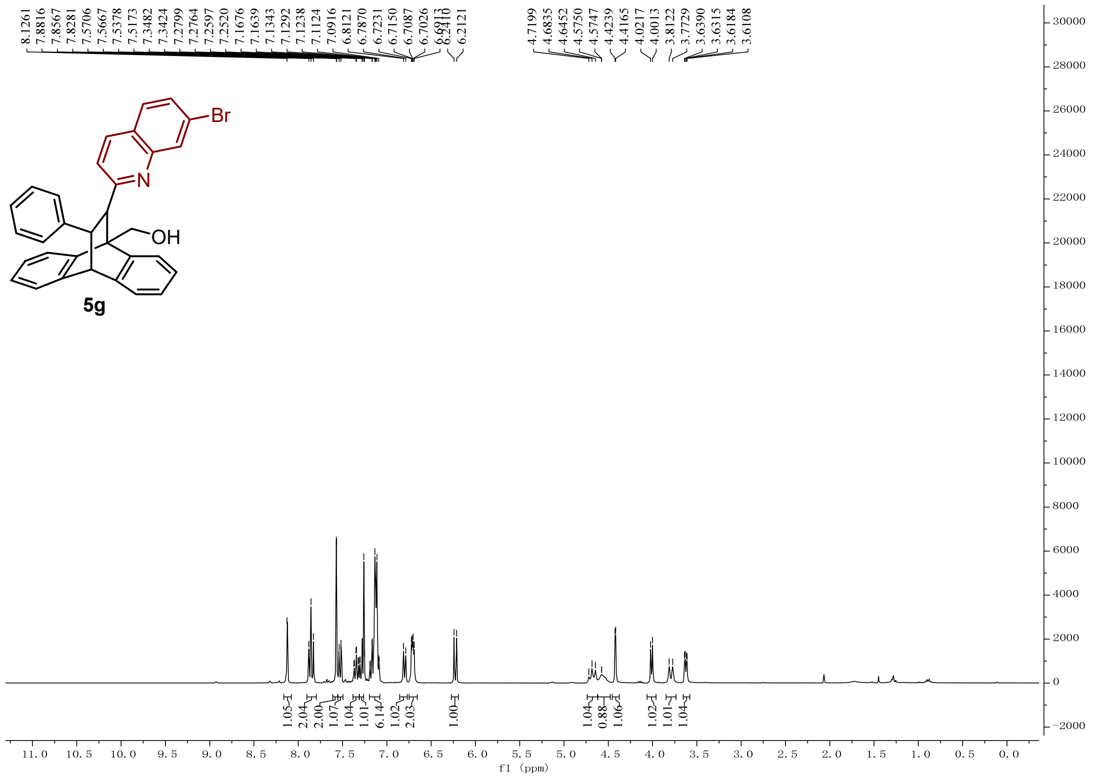

$^{13}$C NMR (75 MHz, Chloroform-*d*) of compound **5g**

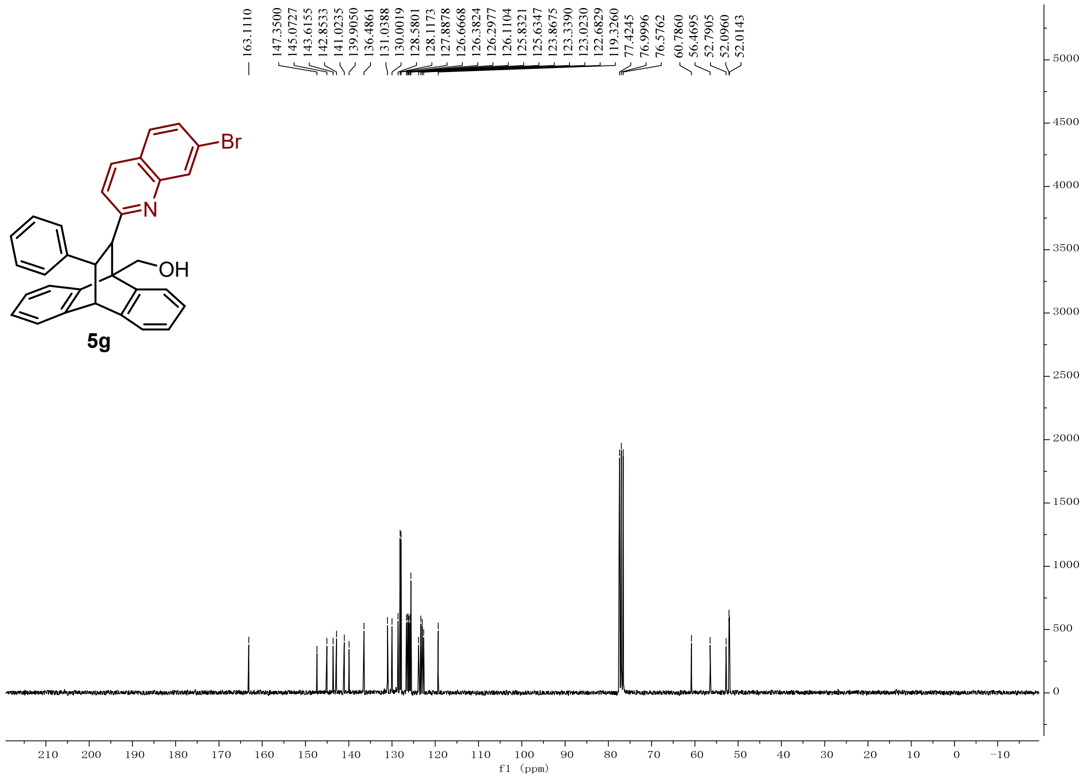


$^{1}$H NMR (300 MHz, Chloroform-*d*) of compound **5h**

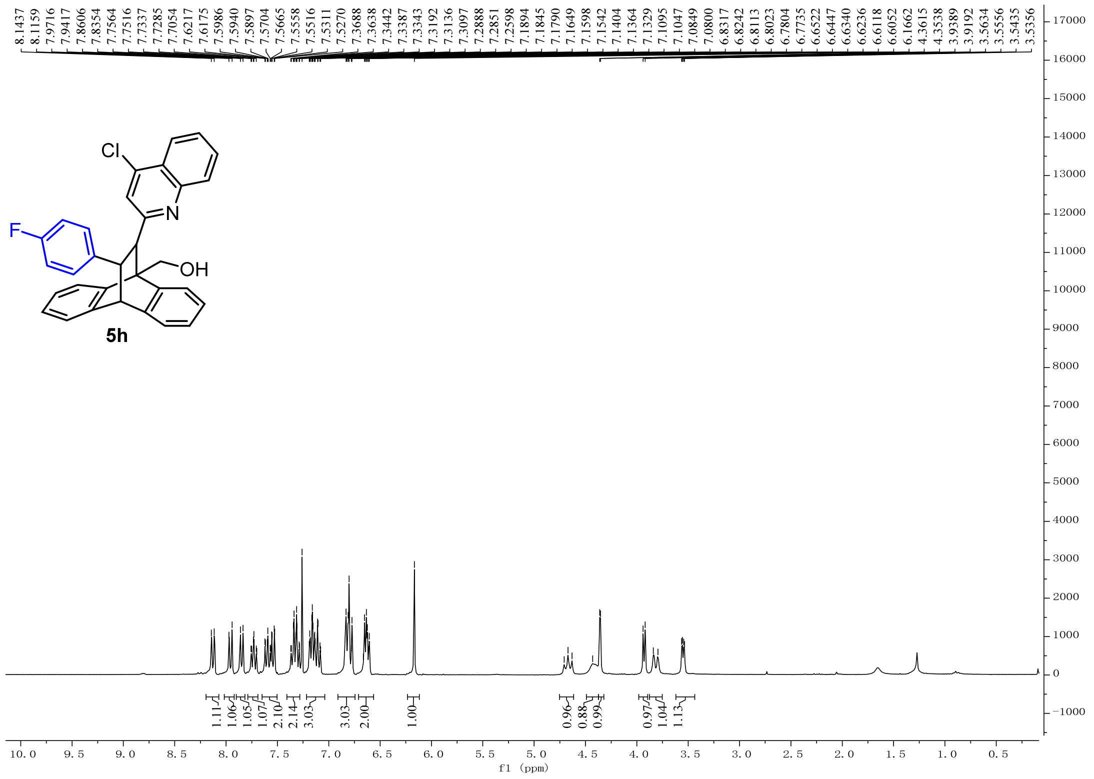

$^{13}C$ NMR (75 MHz, Chloroform-*d*) of compound **5h**

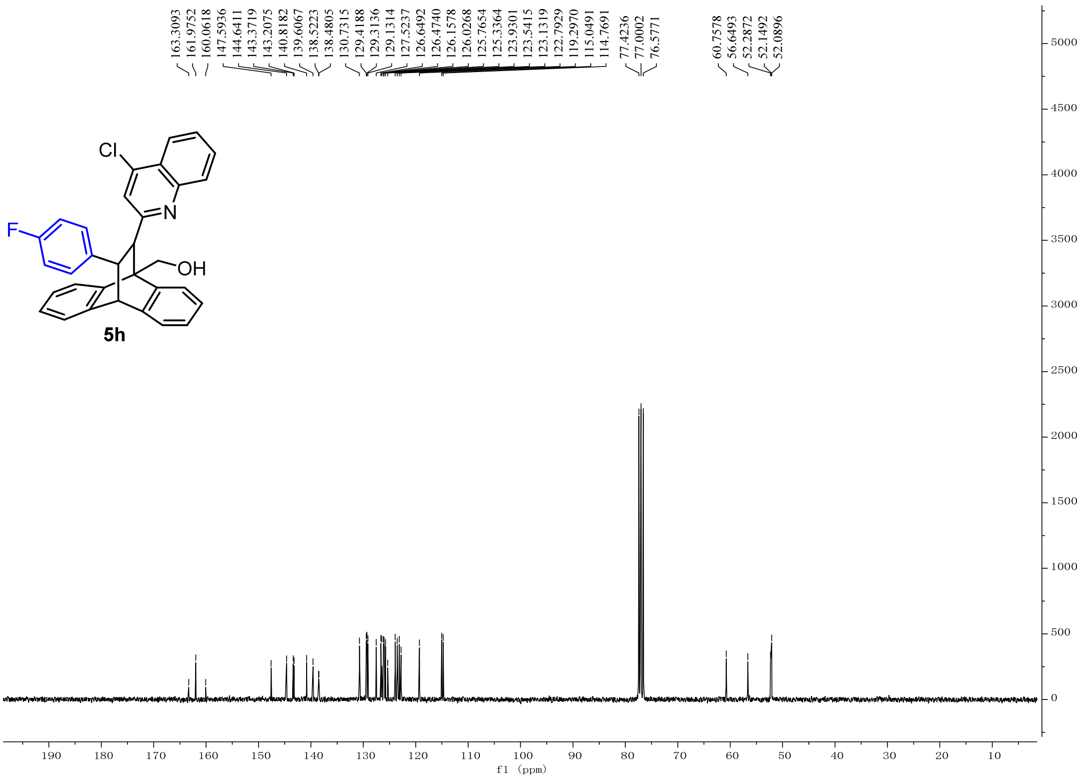

$^{19}F$ NMR (565 MHz, Chloroform-*d*) of compound **5h**

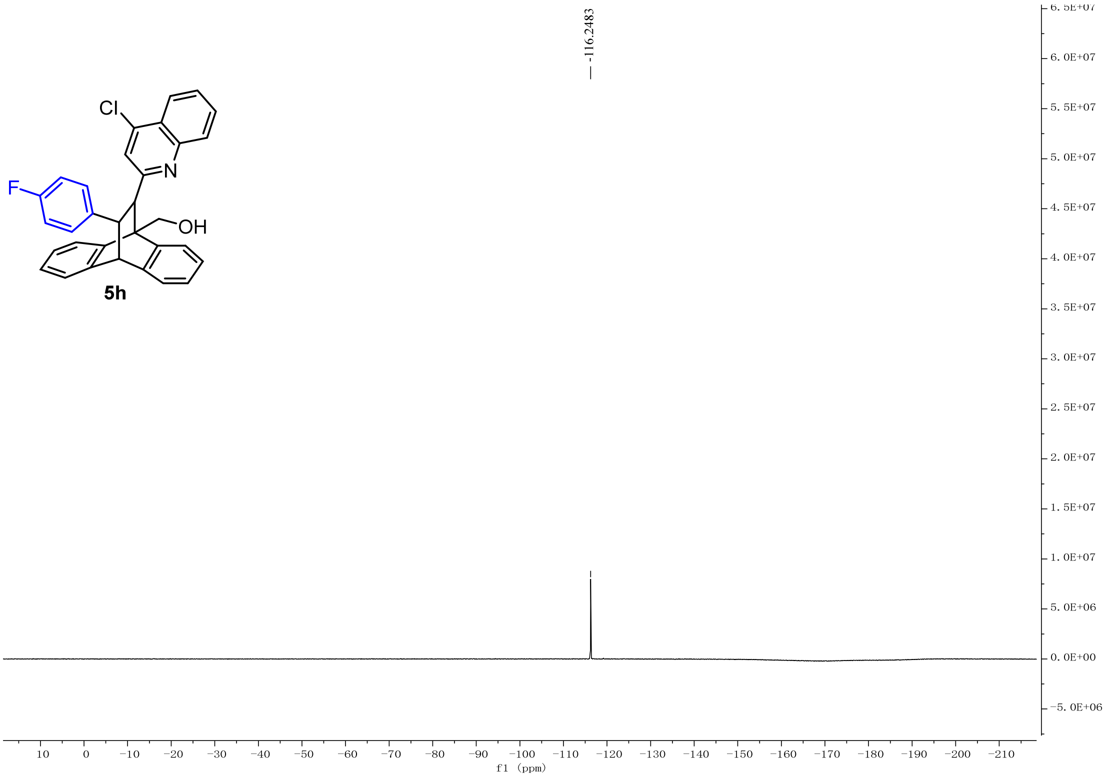

$^{1}$H NMR (300 MHz, Chloroform-*d*) of compound **5i**

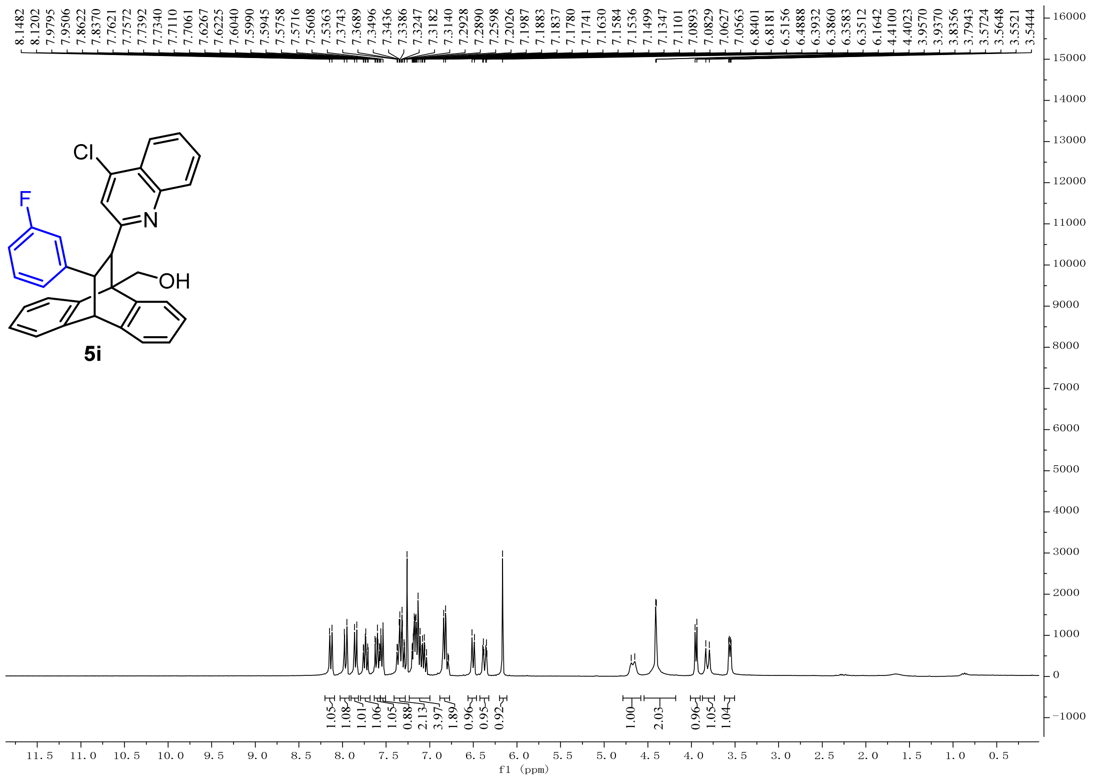

$^{13}$C NMR (75 MHz, Chloroform-*d*) of compound **5i**

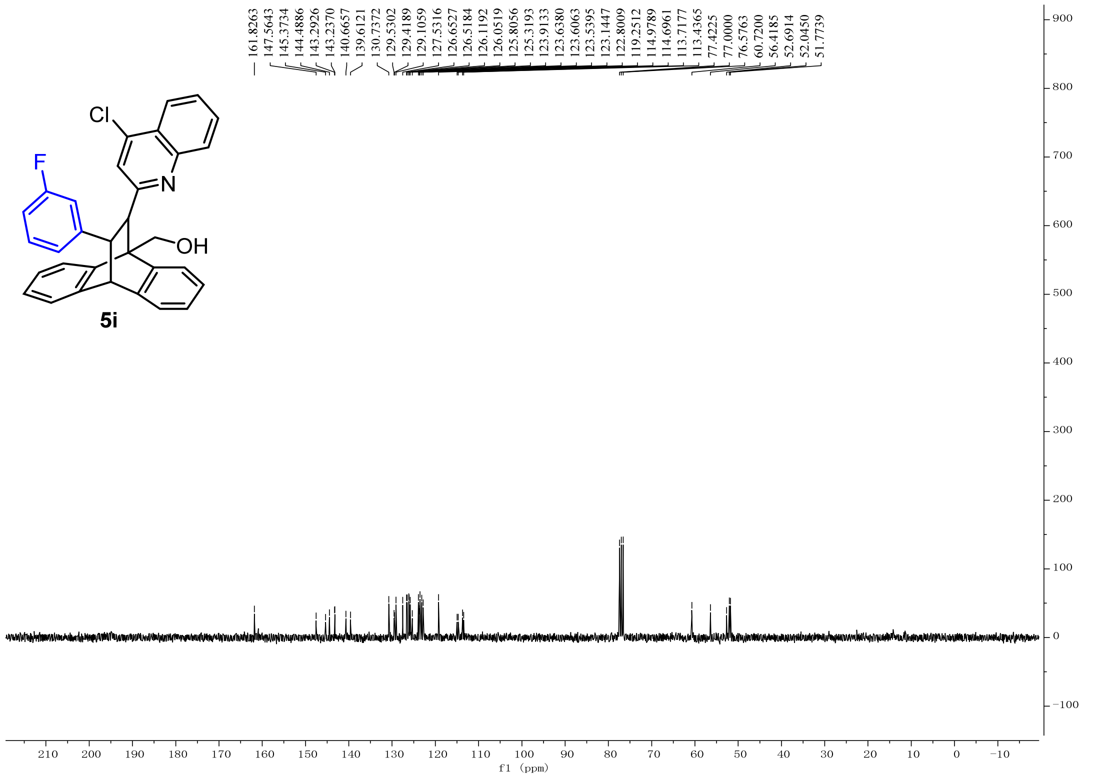

$^{19}F$ NMR (565 MHz, Chloroform-*d*) of compound **5i**

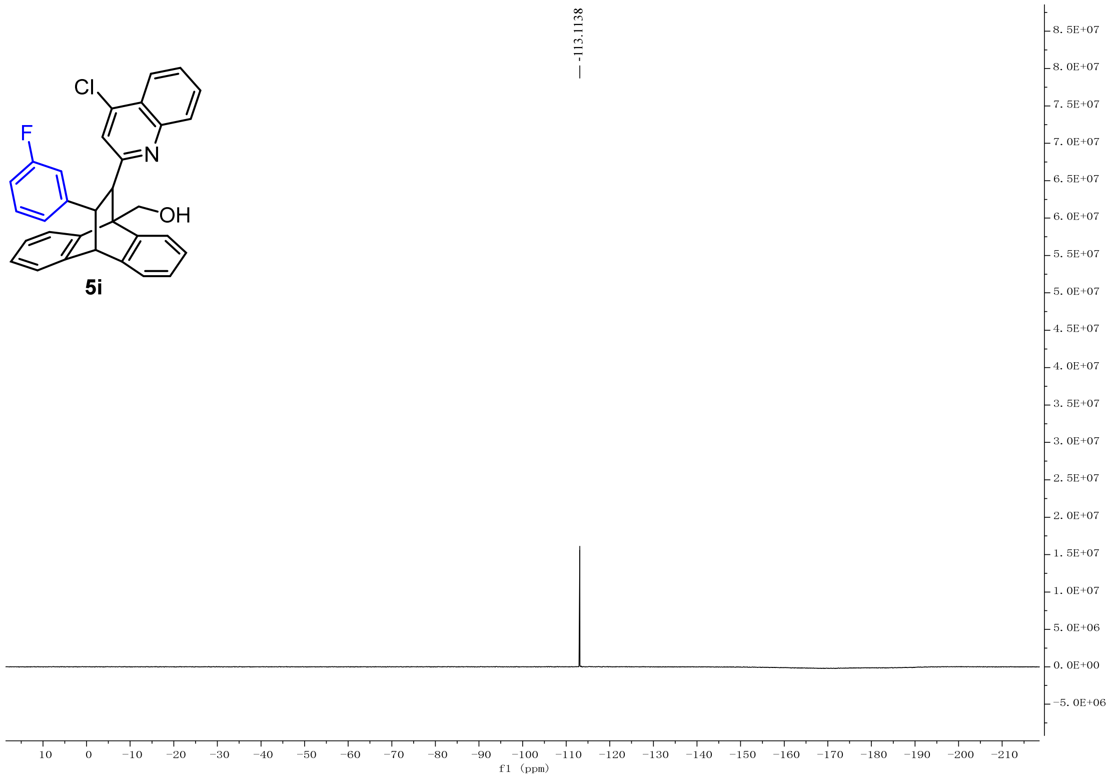


$^{1}H$ NMR (300 MHz, Chloroform-*d*) of compound **5j**

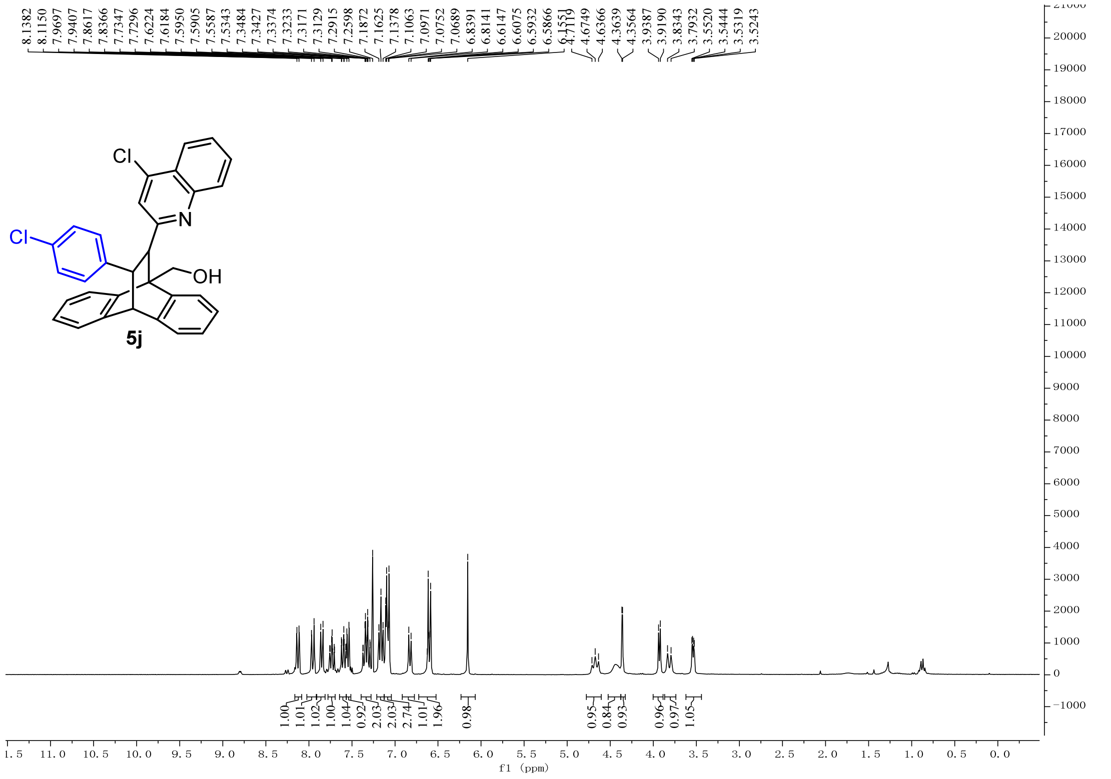

$^{13}$C NMR (75 MHz, Chloroform-*d*) of compound **5j**

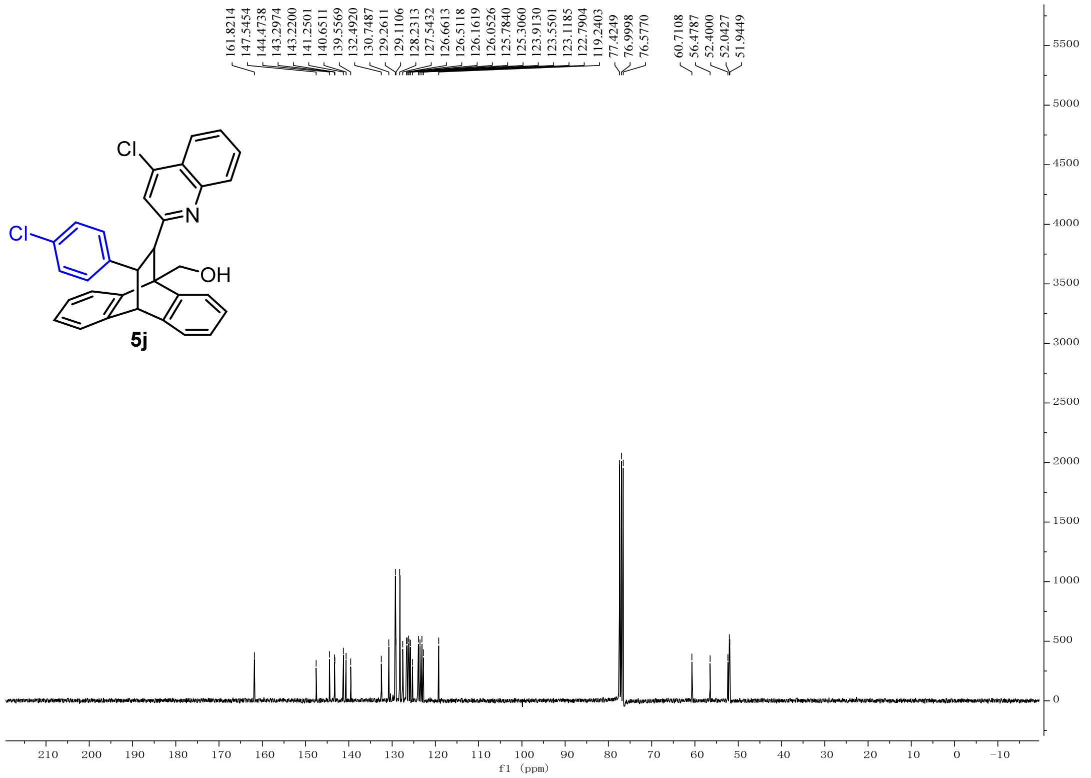


$^{1}$H NMR (300 MHz, Chloroform-*d*) of compound **5k**

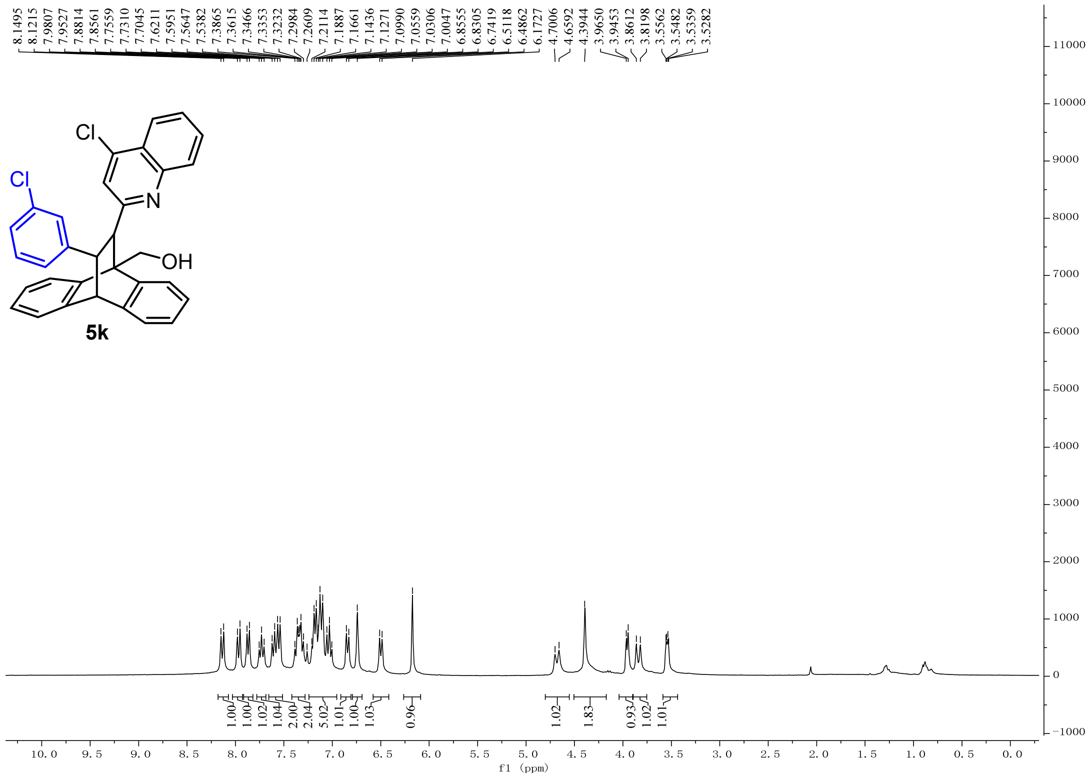

$^{13}$C NMR (75 MHz, Chloroform-*d*) of compound **5k**

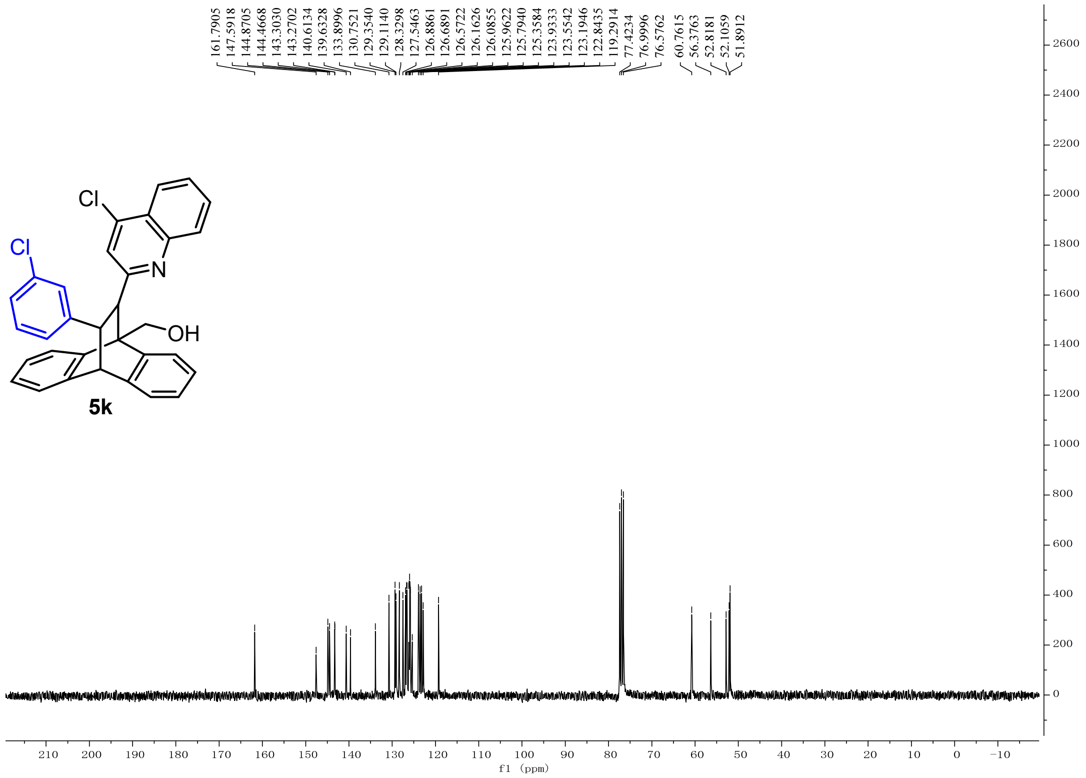


$^{1}$H NMR (300 MHz, Chloroform-*d*) of compound **5l**

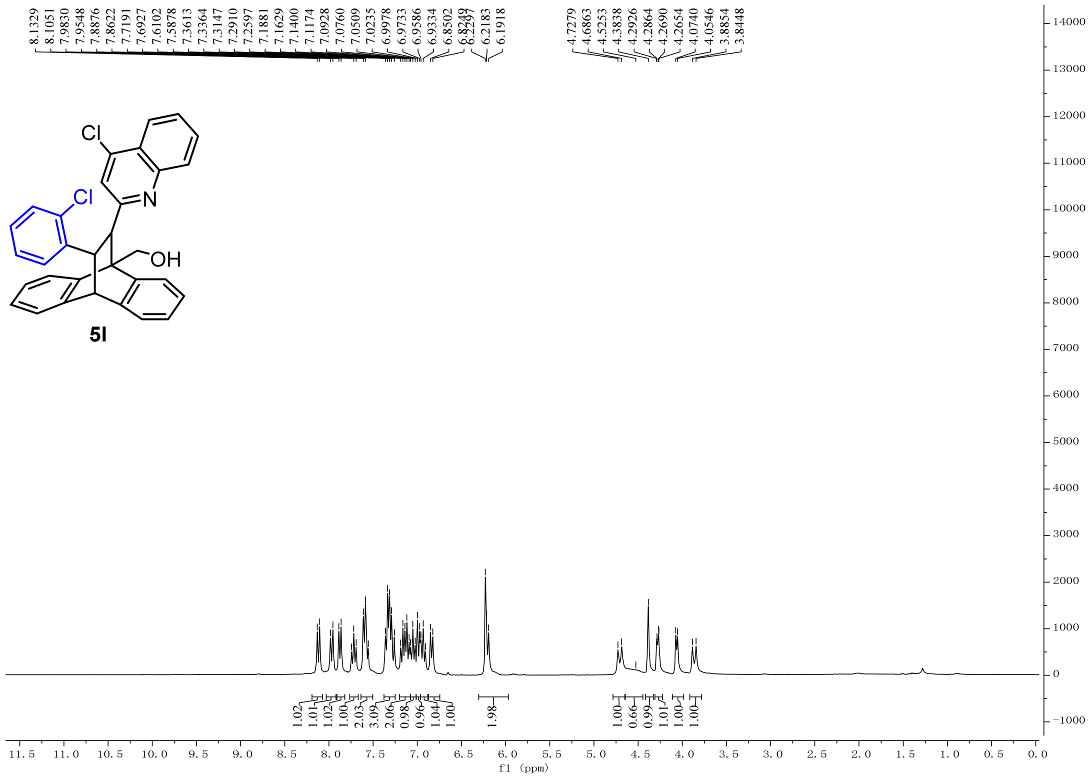

$^{13}C$ NMR (75 MHz, Chloroform-*d*) of compound **5l**

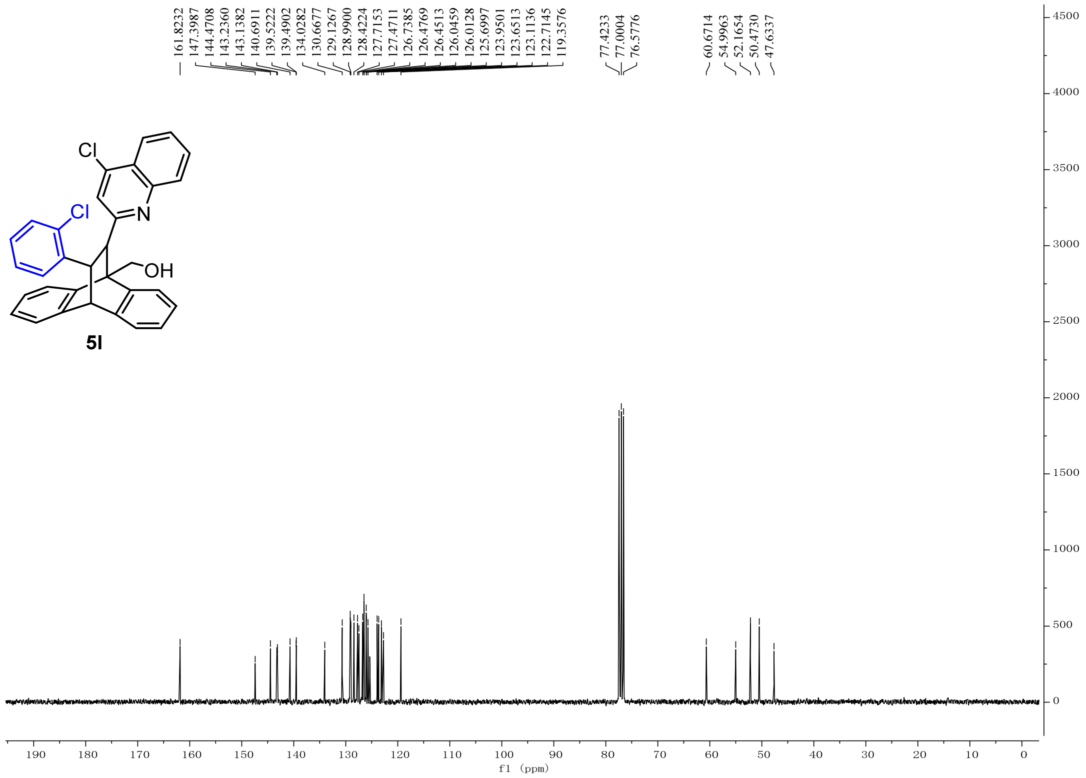


$^{1}H$ NMR (300 MHz, Chloroform-*d*) of compound **5m**

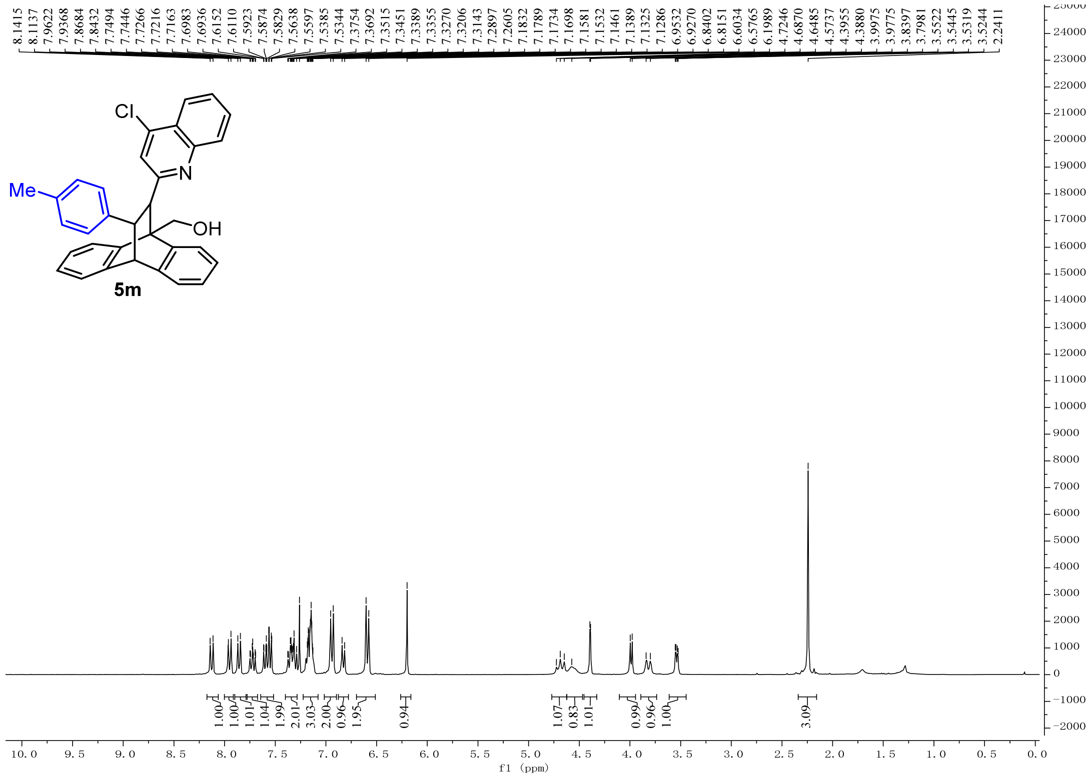

## $^{13}$C NMR (75 MHz, Chloroform-*d*) of compound **5m**

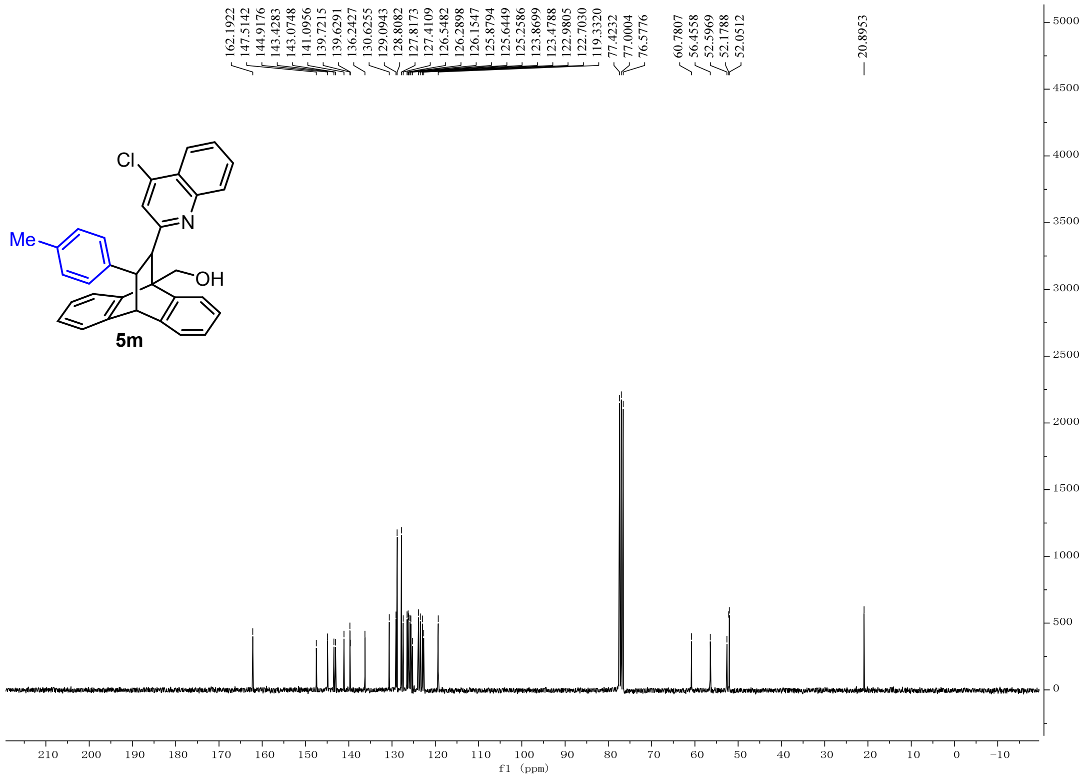


## $^{1}$H NMR (300 MHz, Chloroform-*d*) of compound **5n**

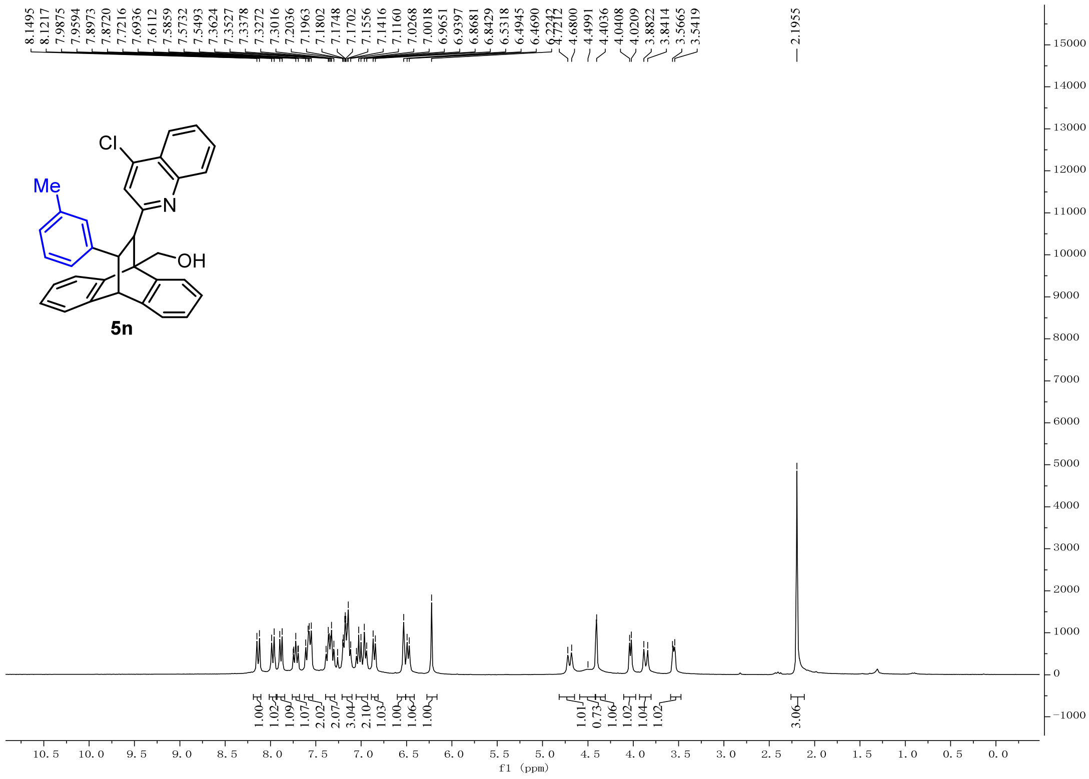

$^{13}$C NMR (75 MHz, Chloroform-*d*) of compound **5n**

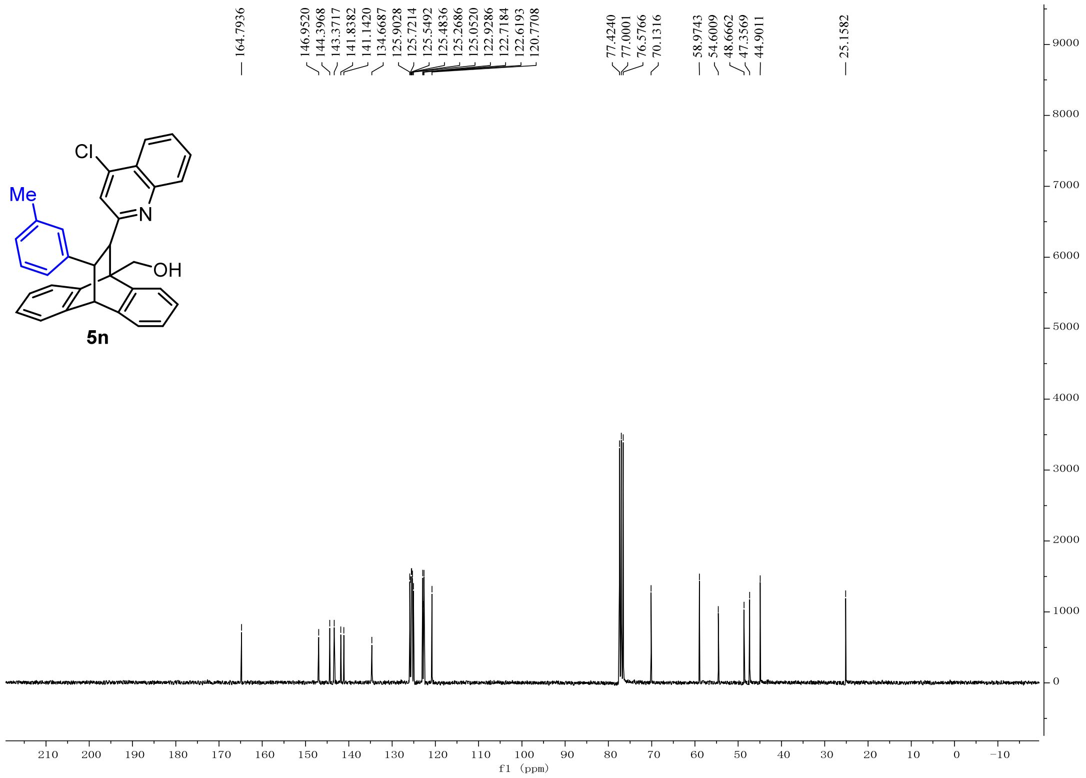


$^{1}$H NMR (300 MHz, Chloroform-*d*) of compound **5o**

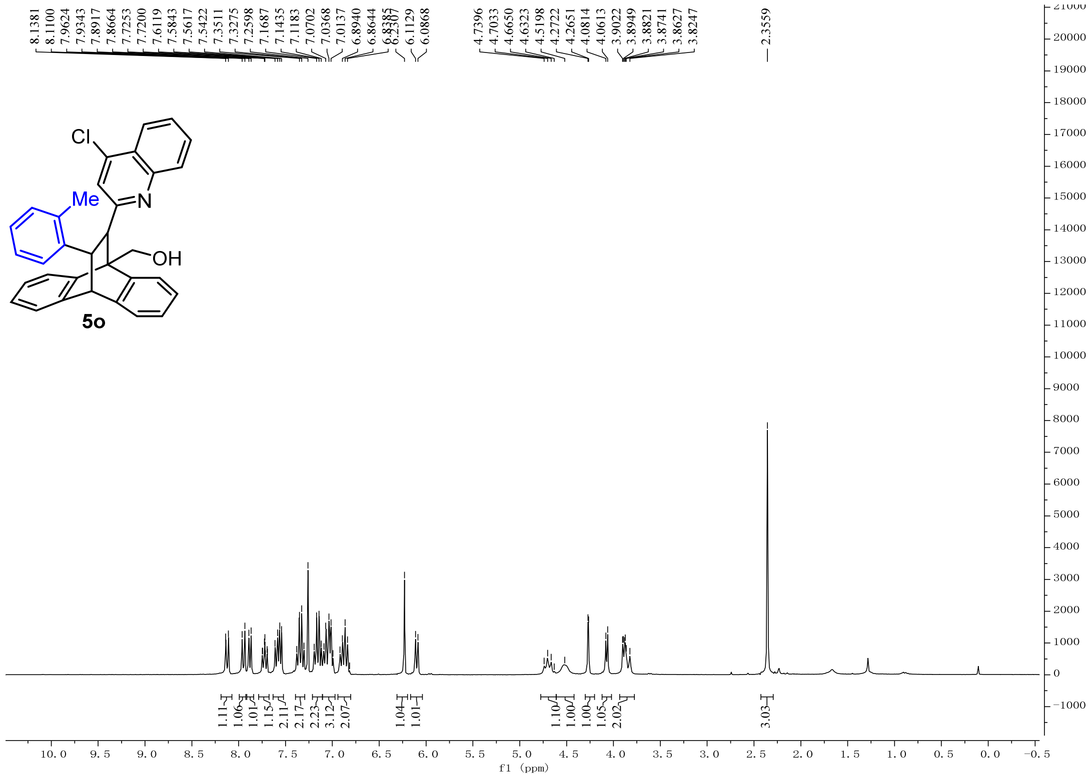

$^{13}$C NMR (75 MHz, Chloroform-*d*) of compound **5o**

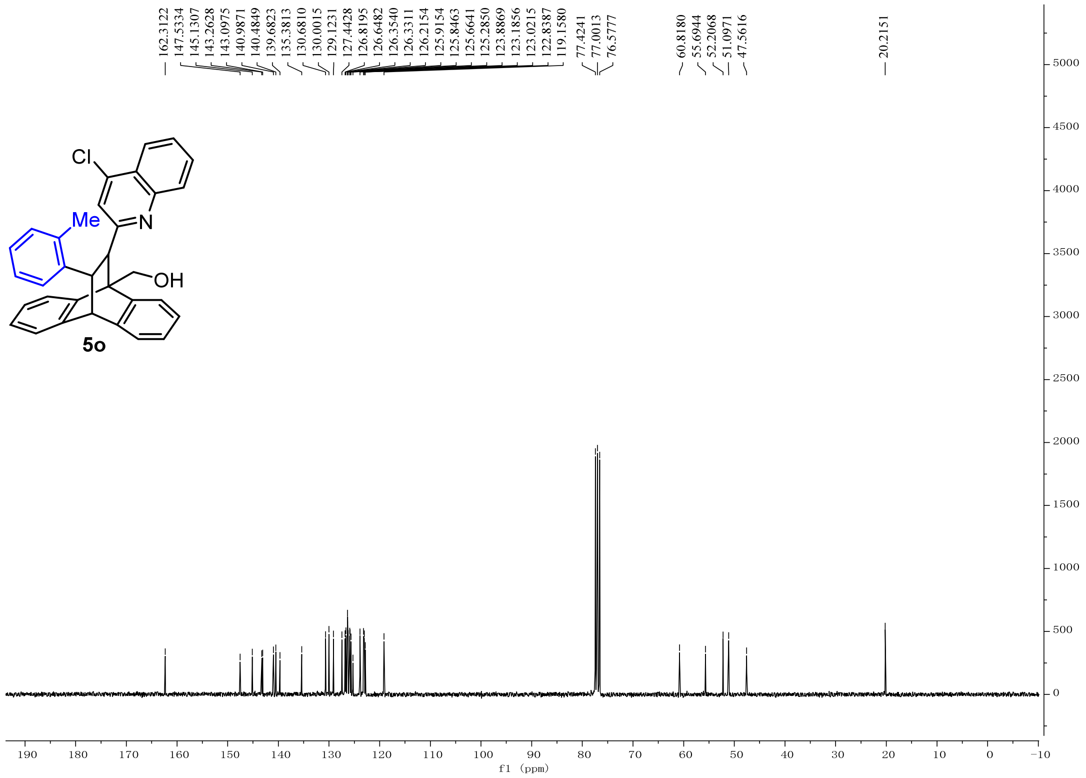


$^{1}$H NMR (300 MHz, Chloroform-*d*) of compound **5p**

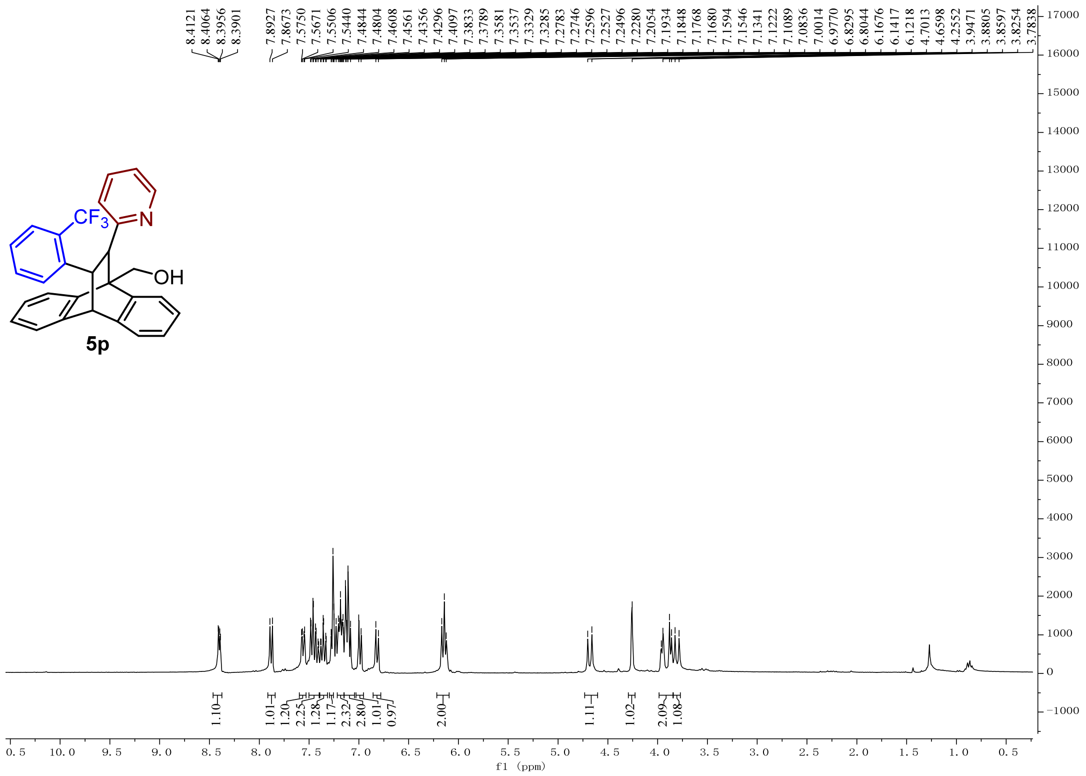

$^{13}C$ NMR (75 MHz, Chloroform-*d*) of compound **5p**

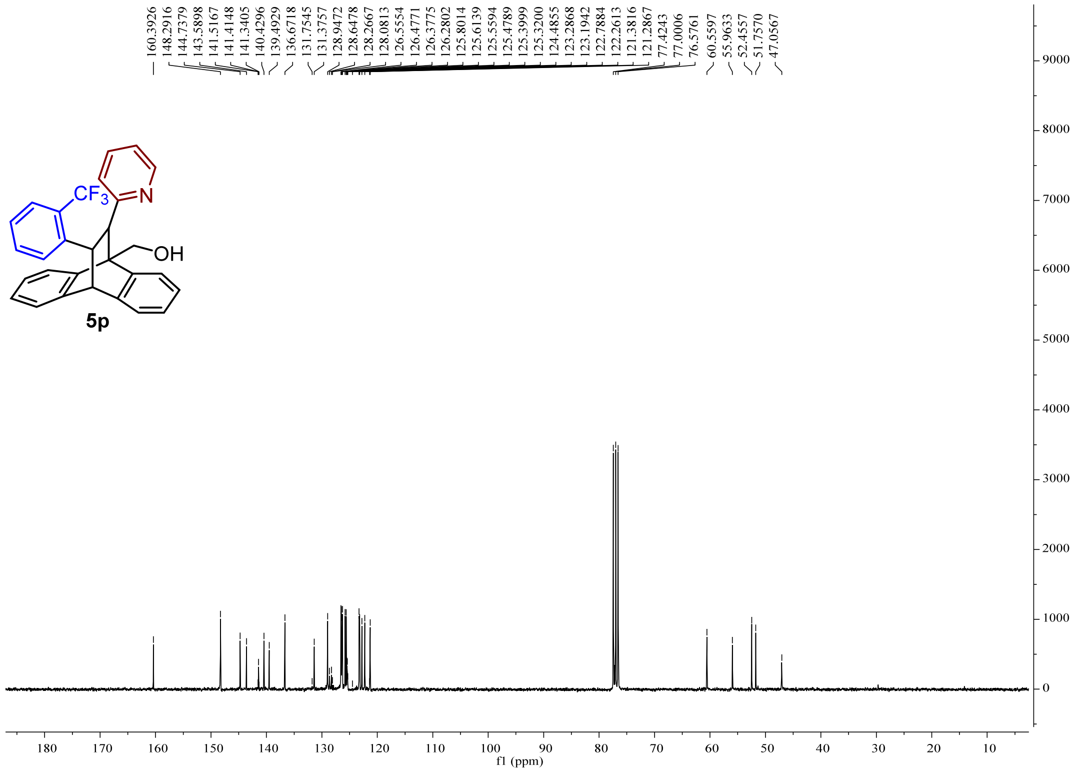


$^{19}F$ NMR (565 MHz, Chloroform-*d*) of compound **5p**

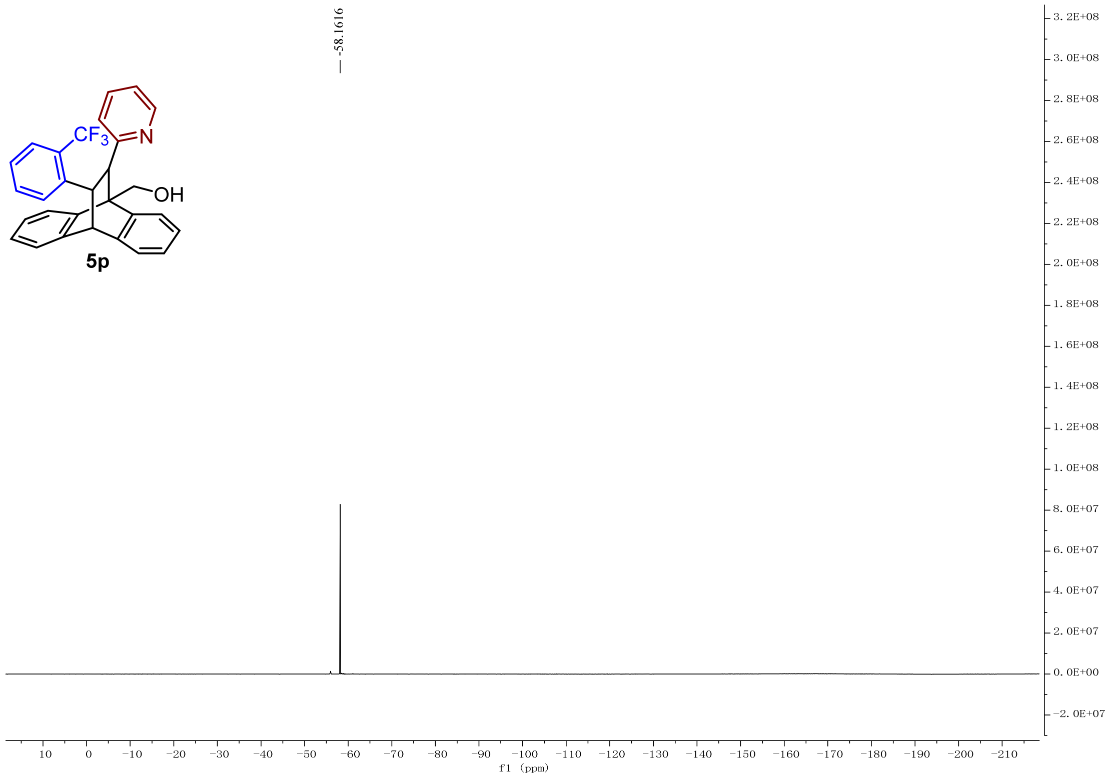

$^{1}$H NMR (300 MHz, Chloroform-*d*) of compound **5q**

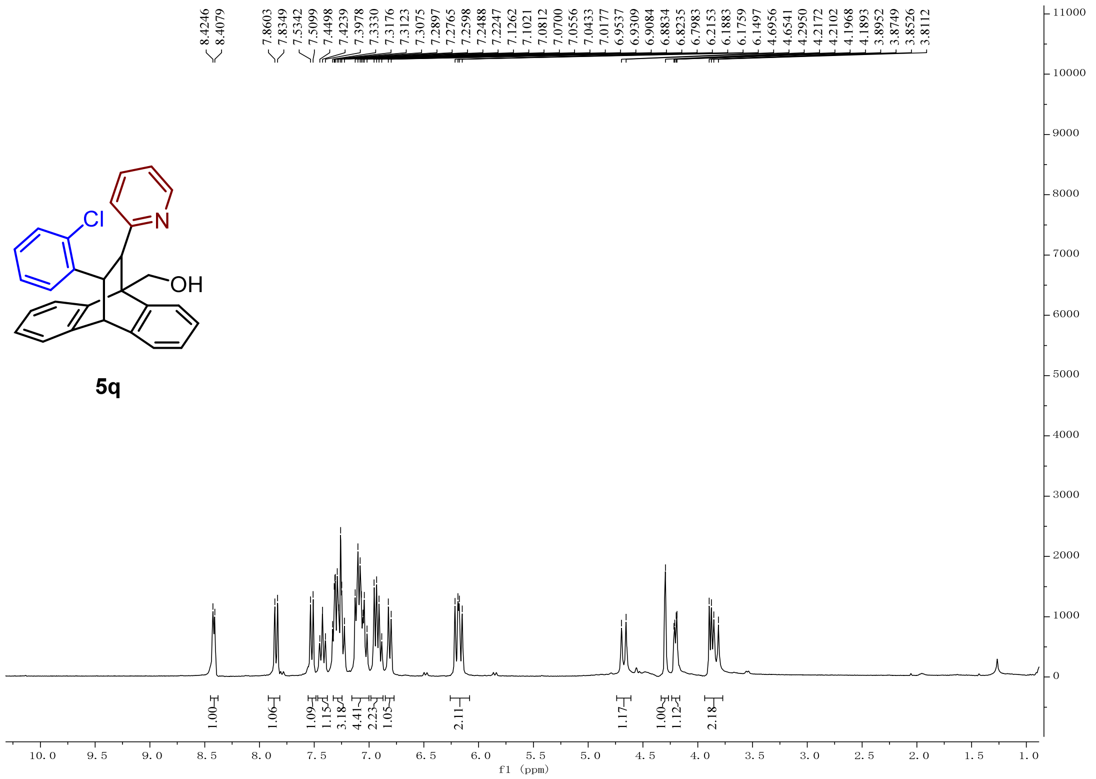


$^{13}$C NMR (75 MHz, Chloroform-*d*) of compound **5q**

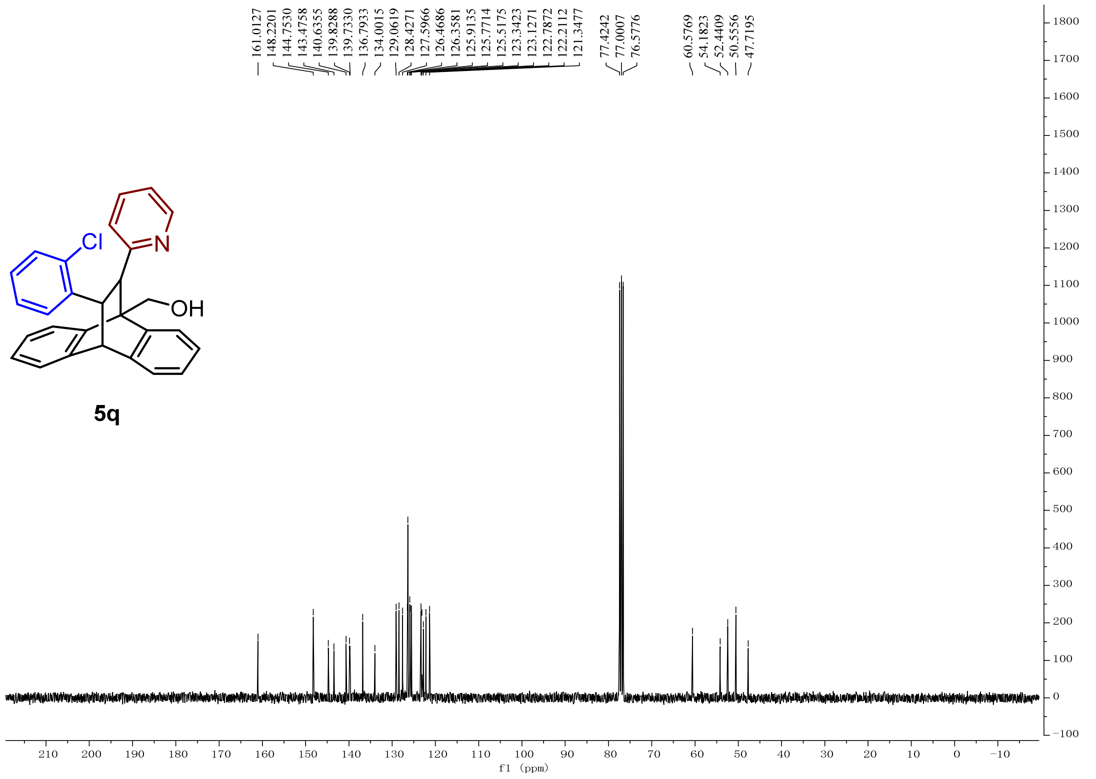

$^{1}$H NMR (300 MHz, Chloroform-*d*) of compound **5r**

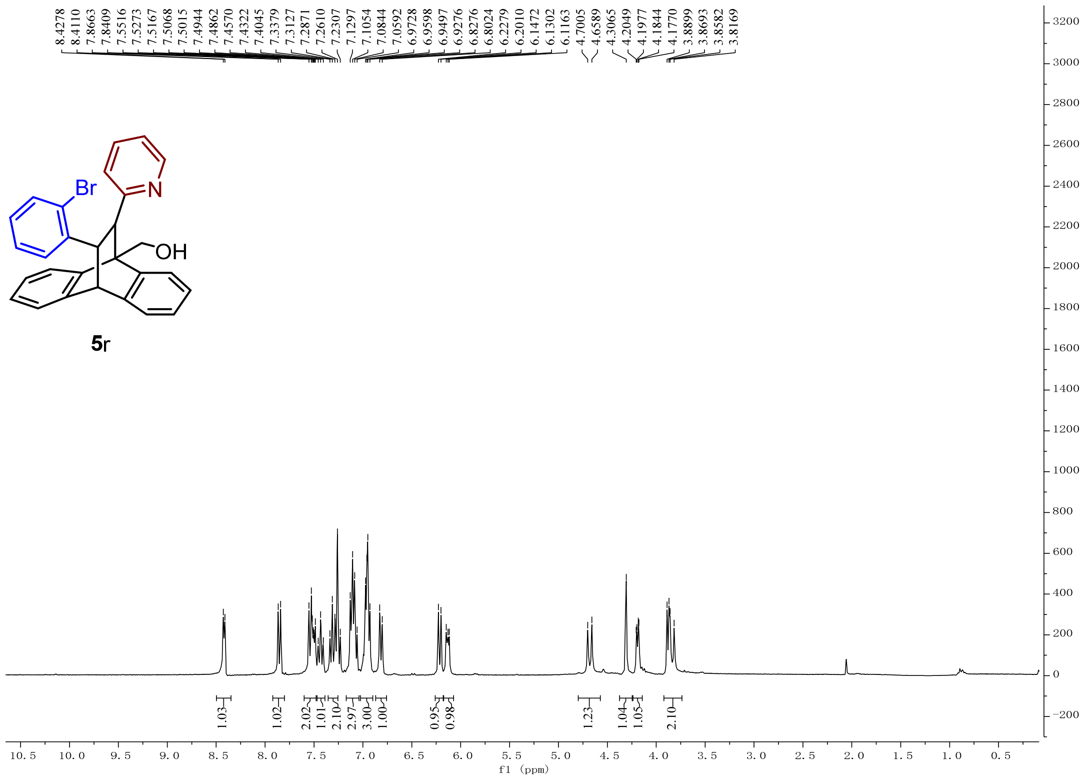


$^{13}$C NMR (75 MHz, Chloroform-*d*) of compound **5r**

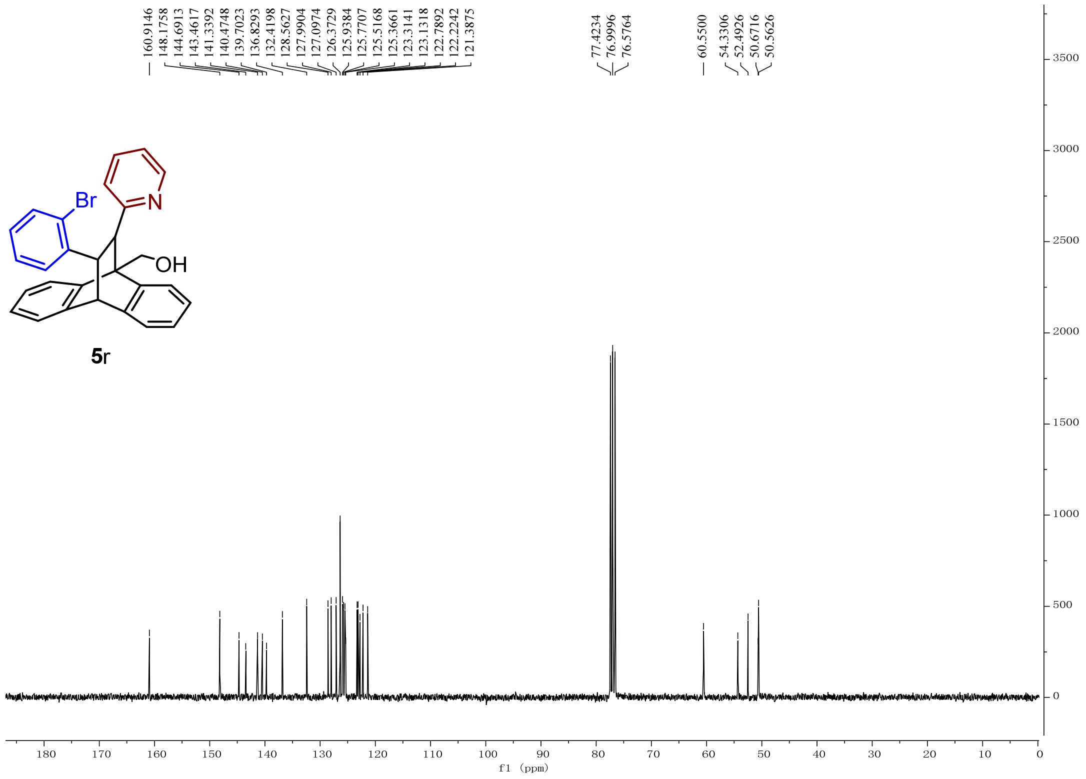

$^{1}$H NMR (300 MHz, Chloroform-*d*) of compound **7a**

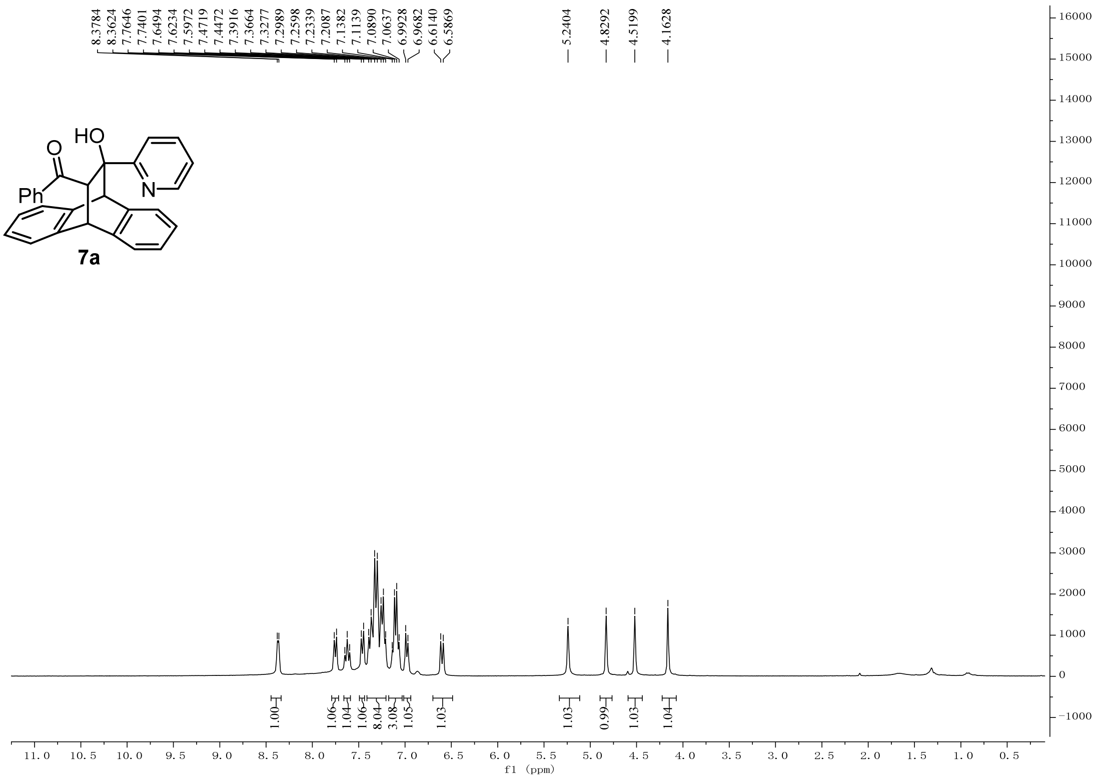


$^{13}$C NMR (75 MHz, Chloroform-*d*) of compound **7a**

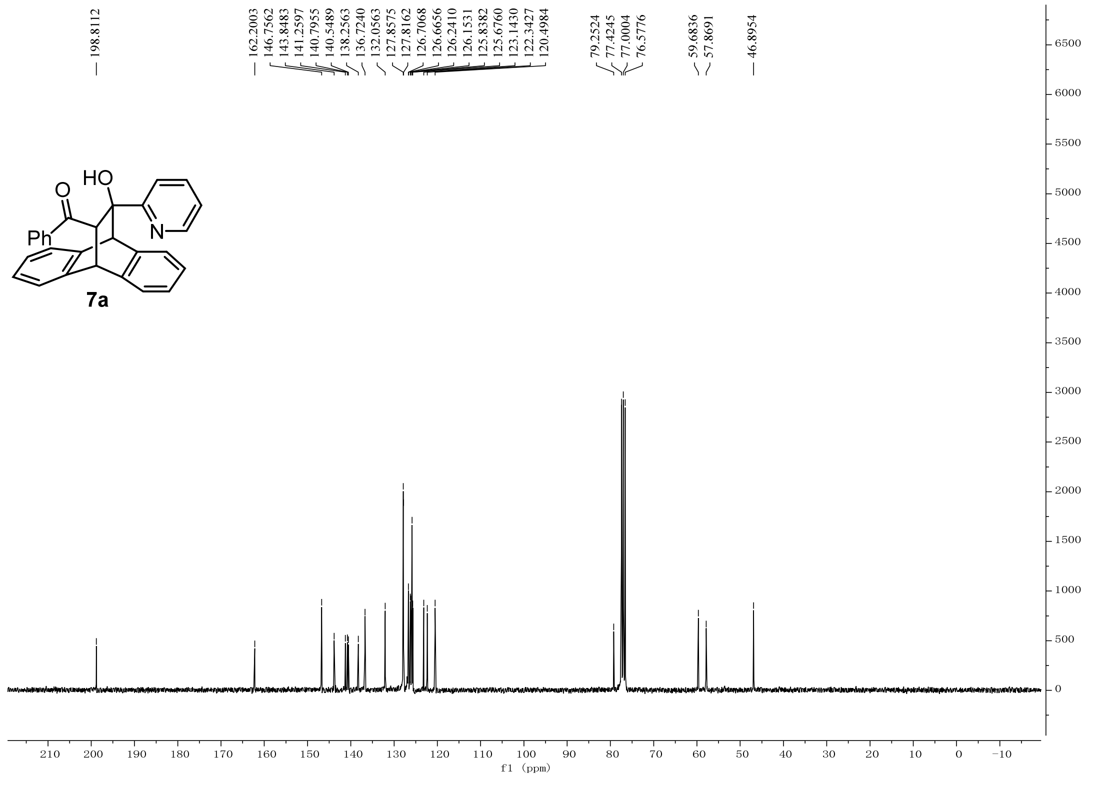

$^{1}$H NMR (300 MHz, Chloroform-*d*) of compound **7b**

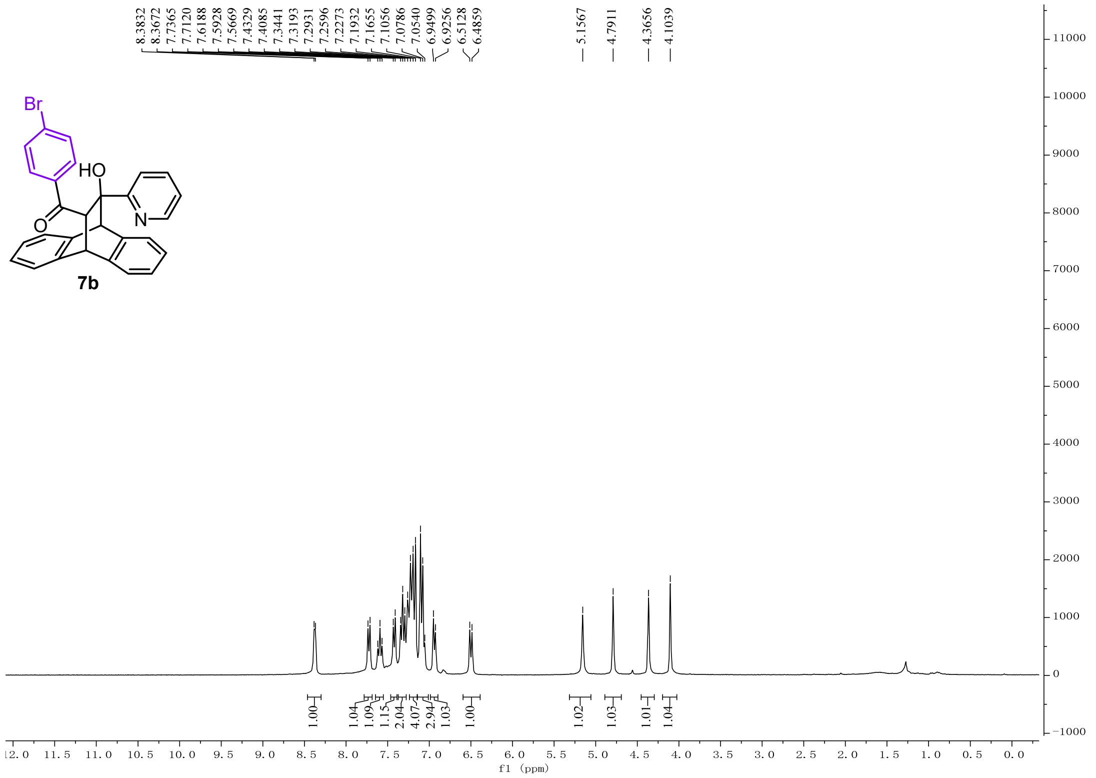


$^{13}$C NMR (75 MHz, Chloroform-*d*) of compound **7b**

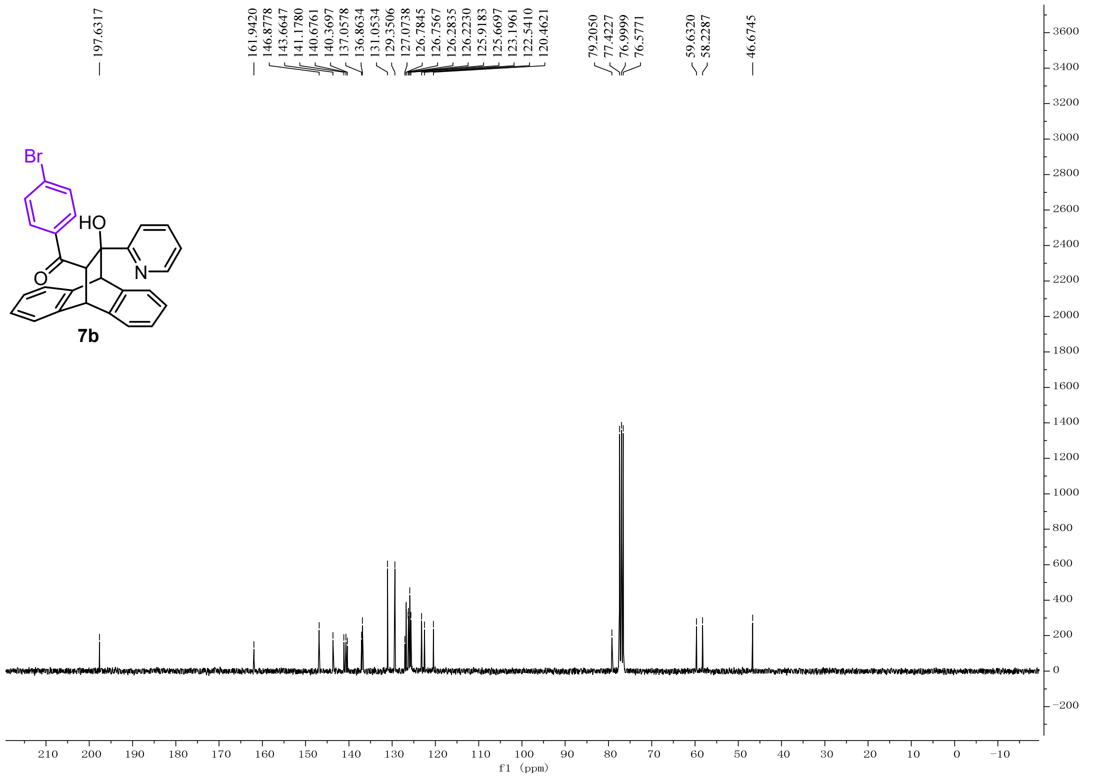

$^{1}$H NMR (300 MHz, Chloroform-*d*) of compound **7c**

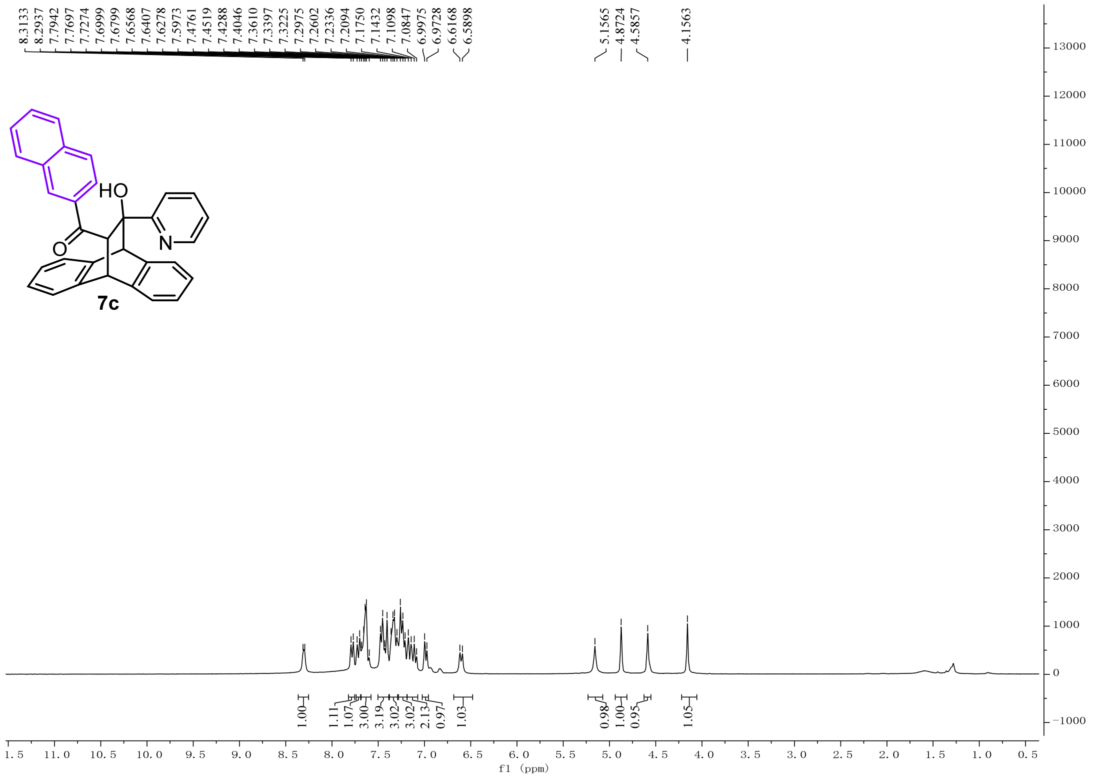

$^{13}$C NMR (75 MHz, Chloroform-*d*) of compound **7c**

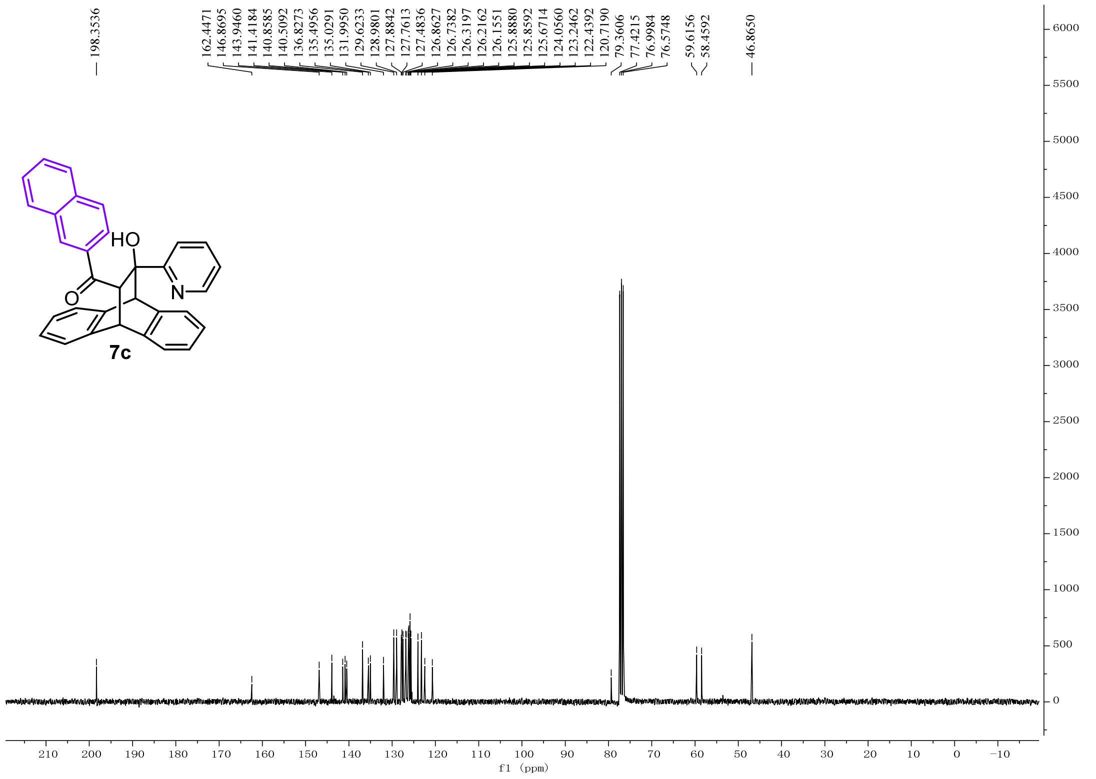

$^{1}$H NMR (300 MHz, Chloroform-*d*) of compound **7d**

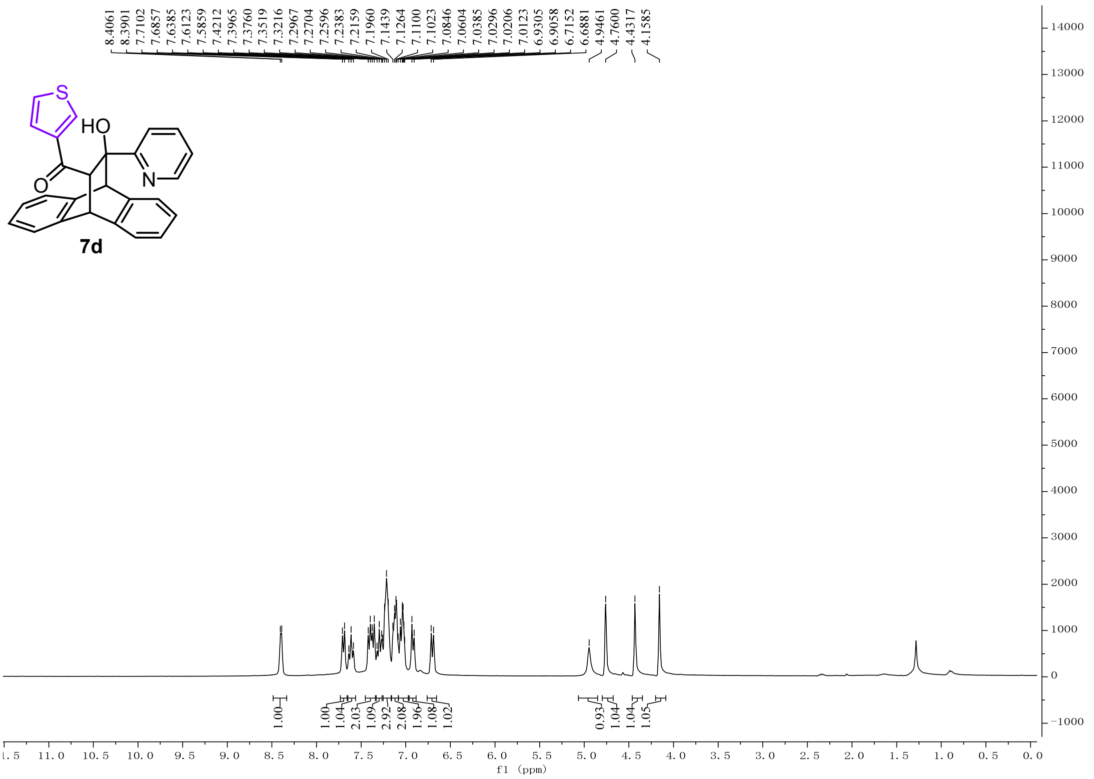

$^{13}$C NMR (75 MHz, Chloroform-*d*) of compound **7d**

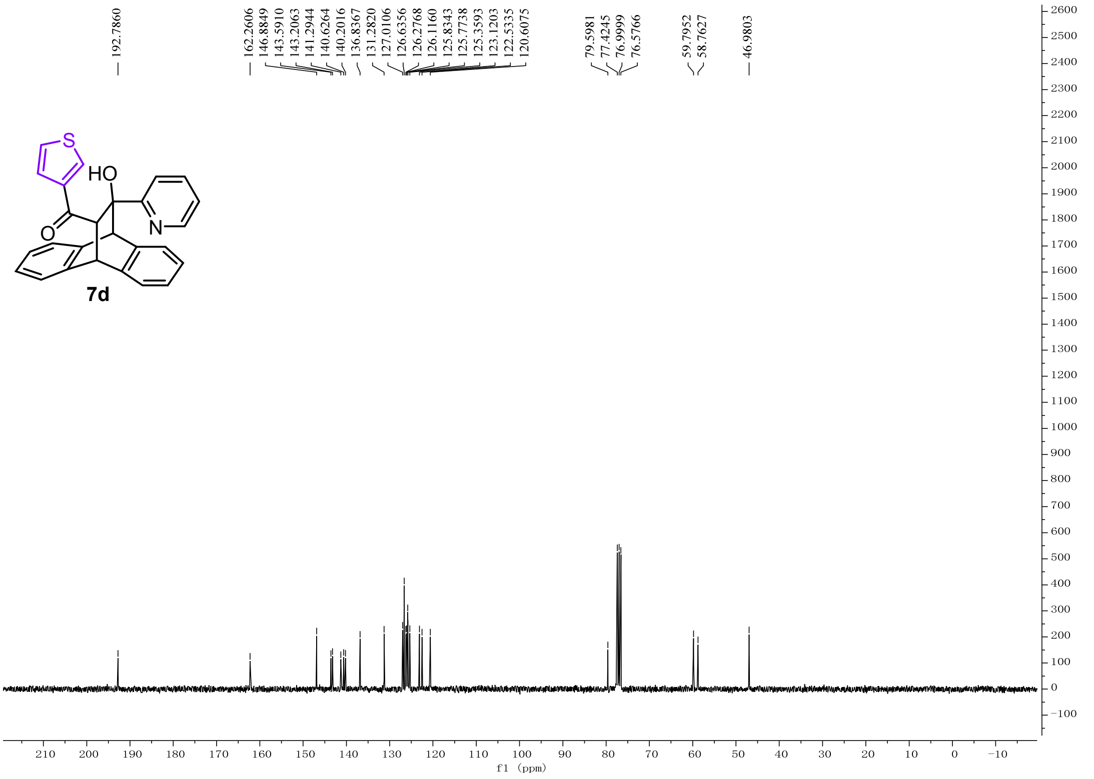

$^{1}$H NMR (300 MHz, Chloroform-*d*) of compound **7e**

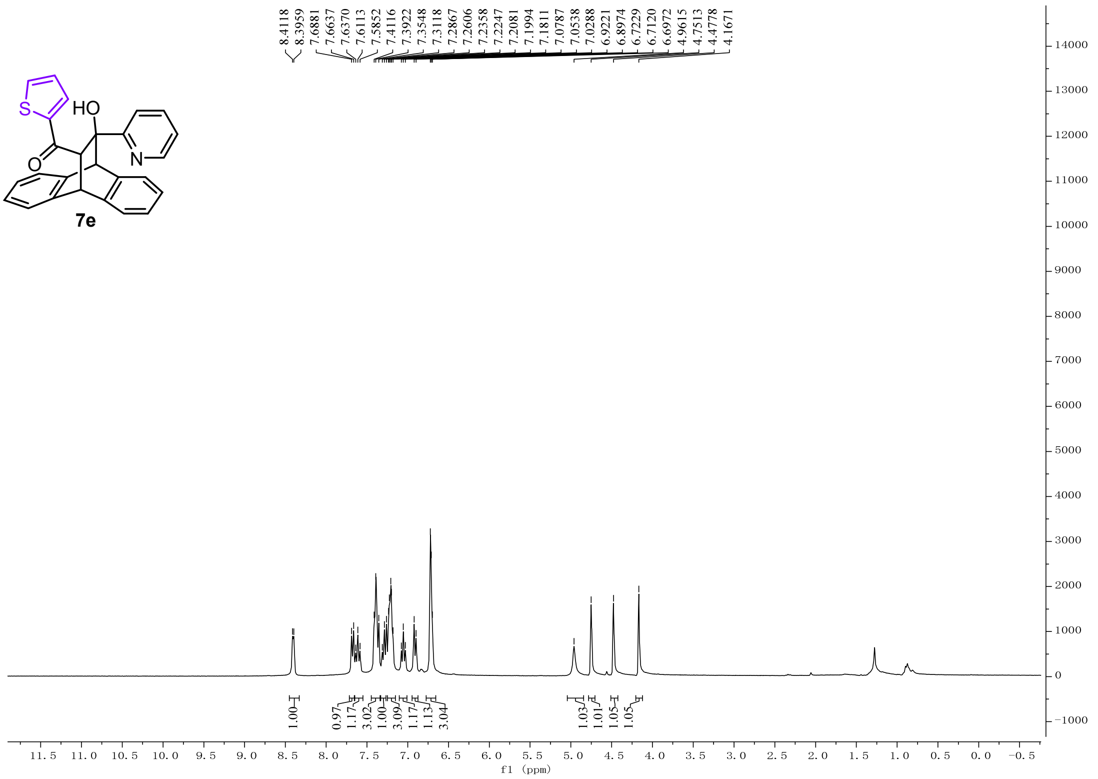


$^{13}$C NMR (75 MHz, Chloroform-*d*) of compound **7e**

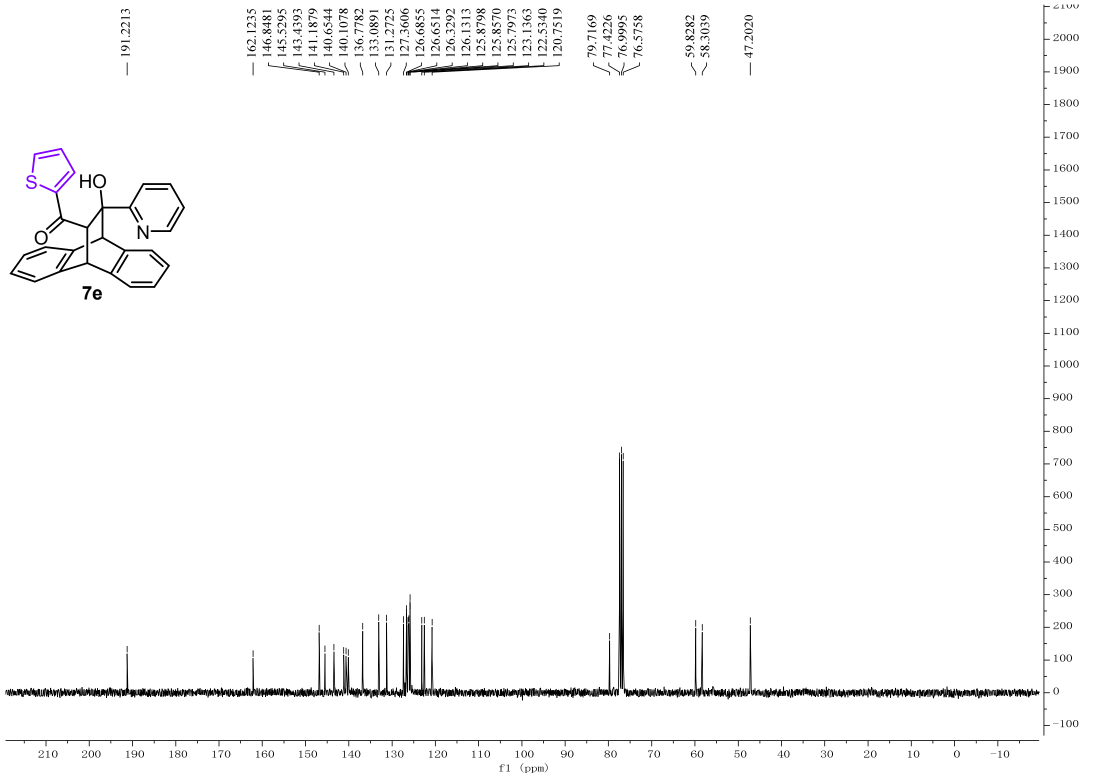

$^{1}$H NMR (300 MHz, Methylene Chloride-$d_2$) of compound **7f**

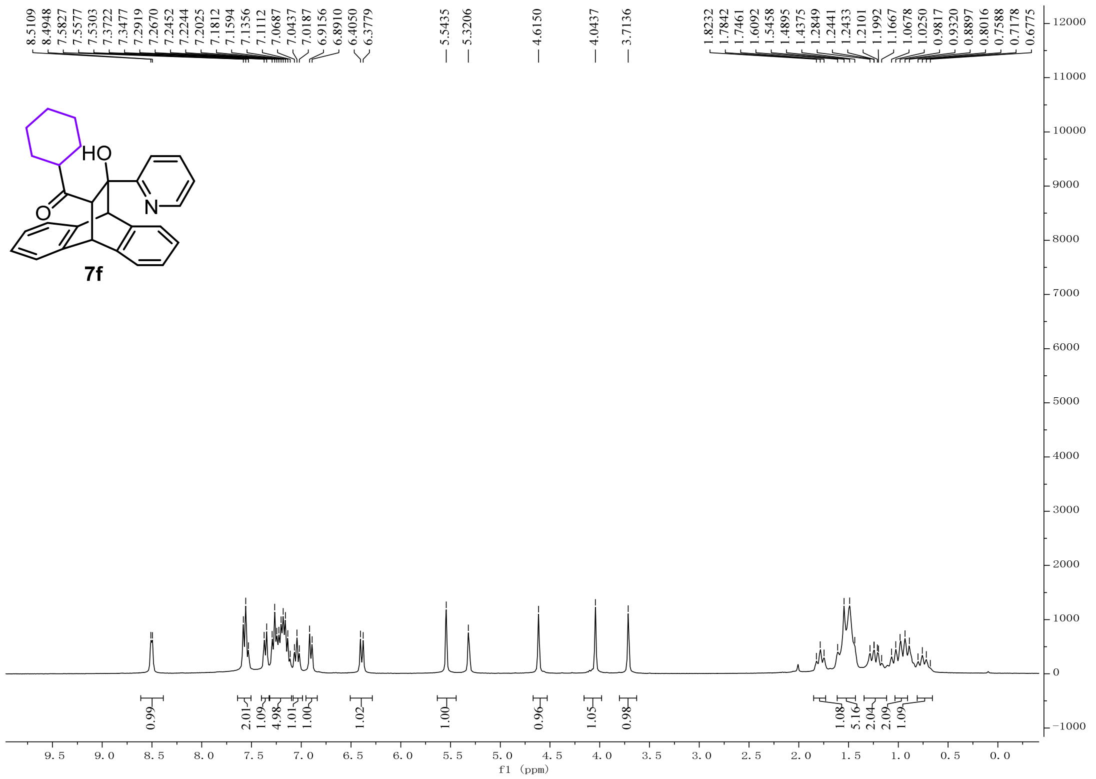

$^{13}$C NMR (75 MHz, Methylene Chloride-$d_2$) of compound **7f**

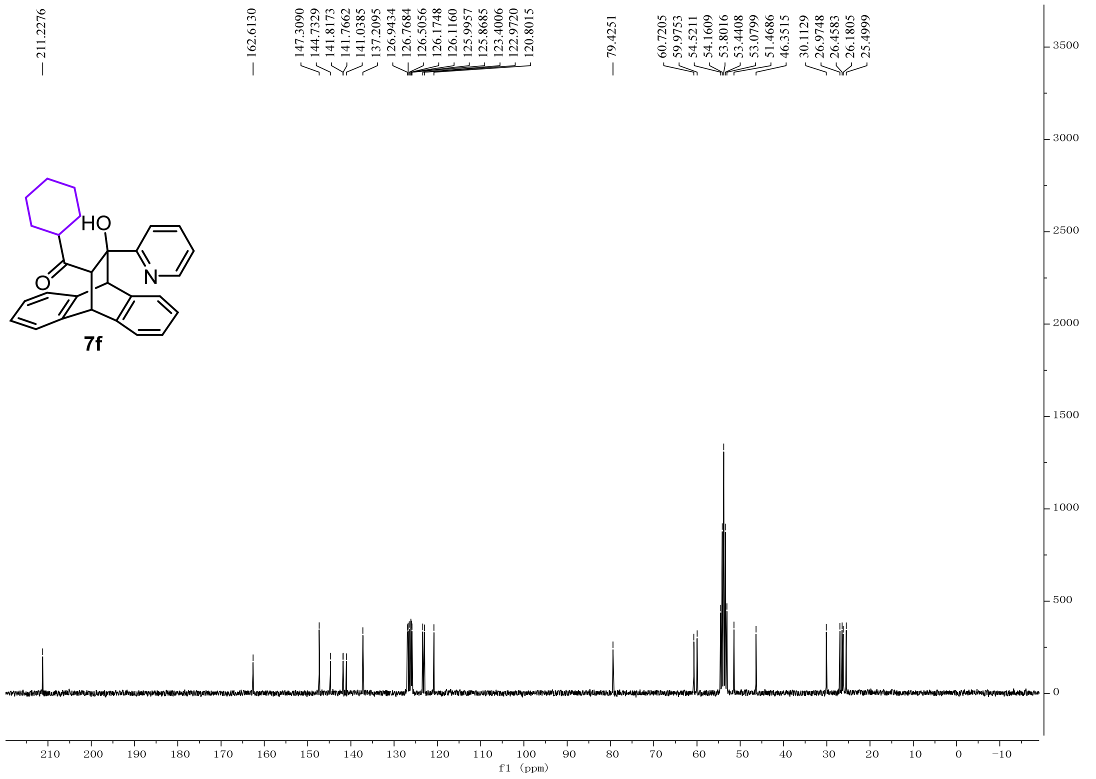

$^{1}$H NMR (300 MHz, Chloroform-*d*) of compound **7g**

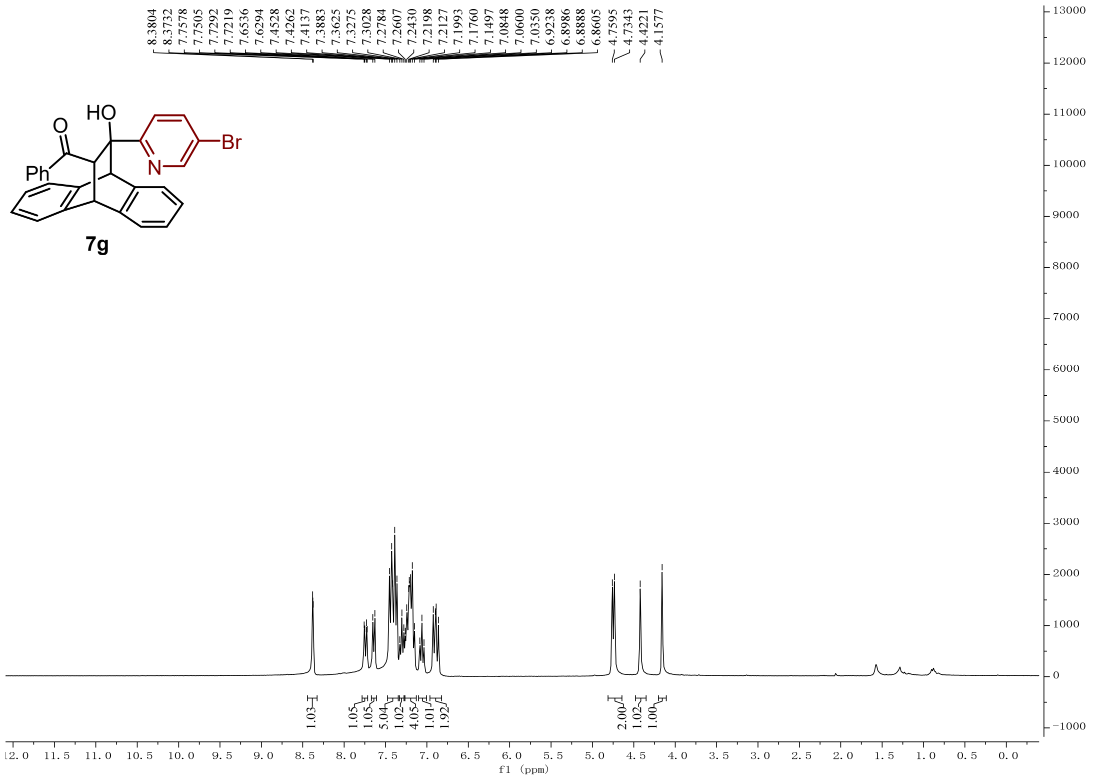


$^{13}$C NMR (75 MHz, Chloroform-*d*) of compound **7g**

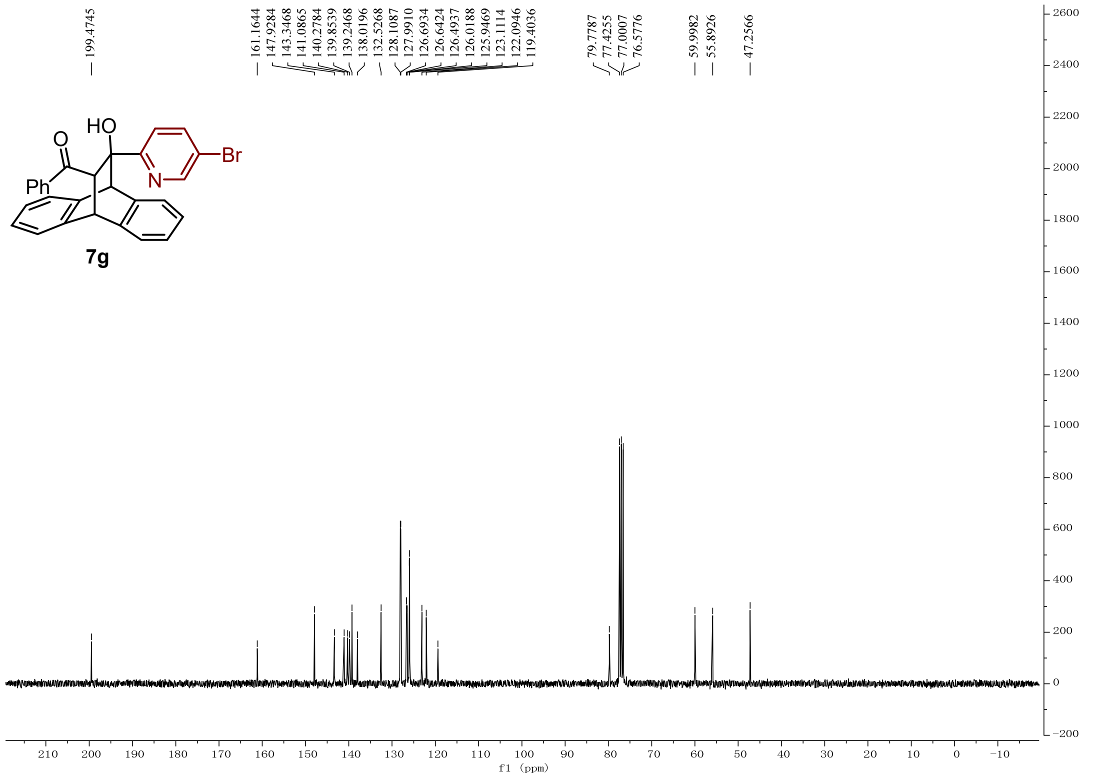

$^{1}$H NMR (300 MHz, Methylene Chloride-$d_2$) of compound **7h**

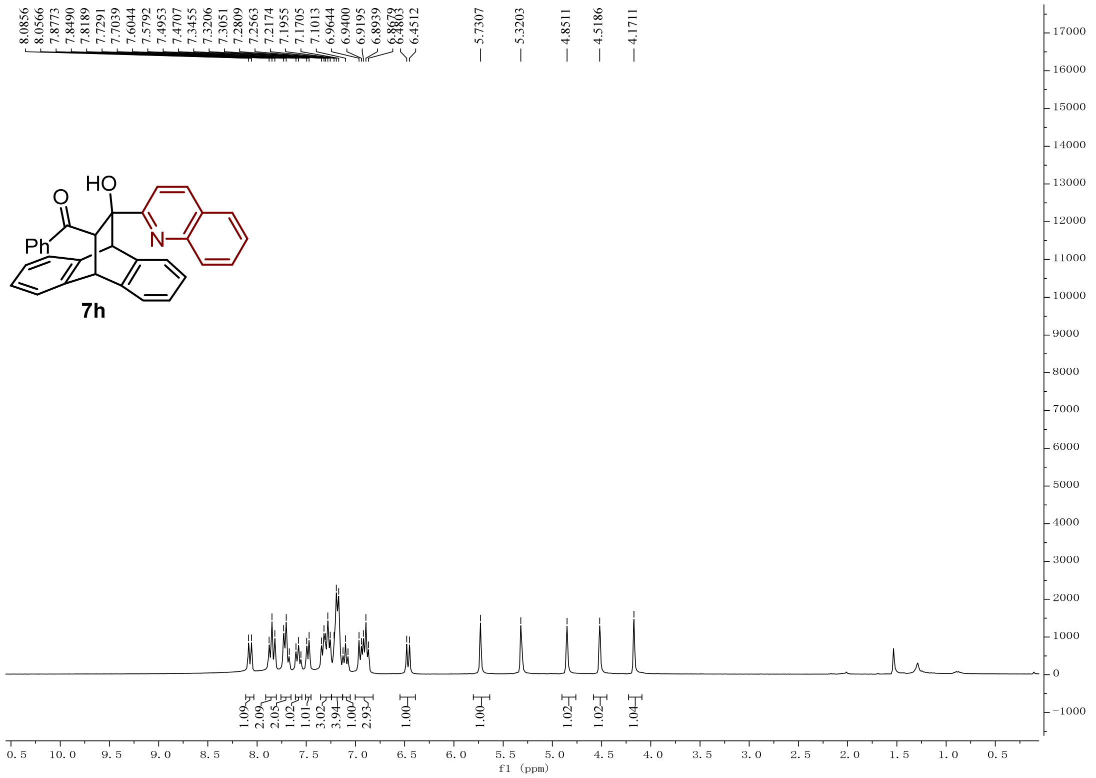


$^{13}$C NMR (75 MHz, Methylene Chloride-$d_2$) of compound **7h**

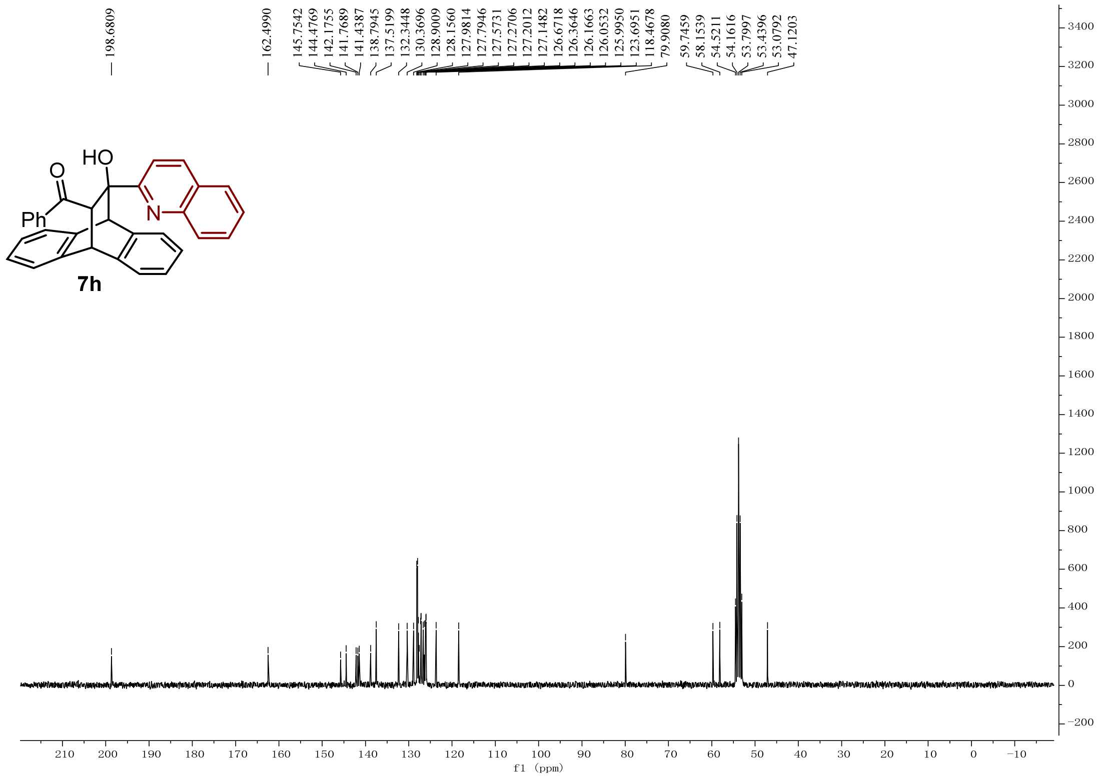

$^{1}$H NMR (300 MHz, Methylene Chloride-$d_2$) of compound **7i**

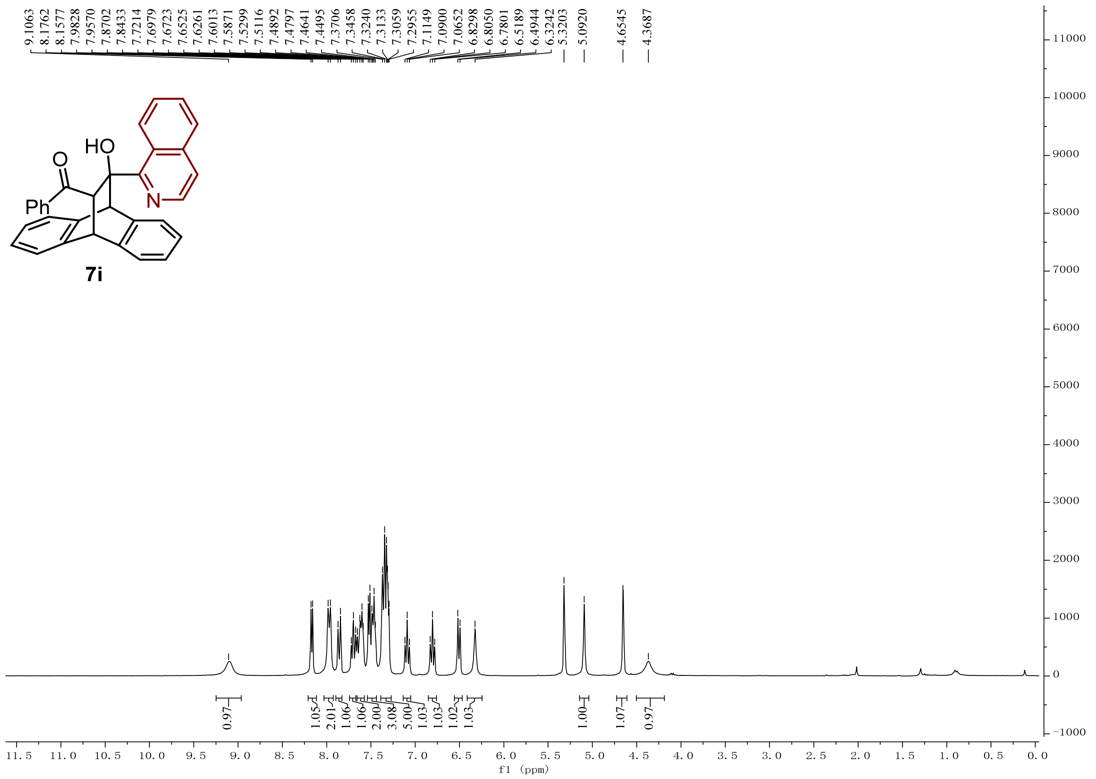

$^{13}$C NMR (75 MHz, Methylene Chloride-$d_2$) of compound **7i**

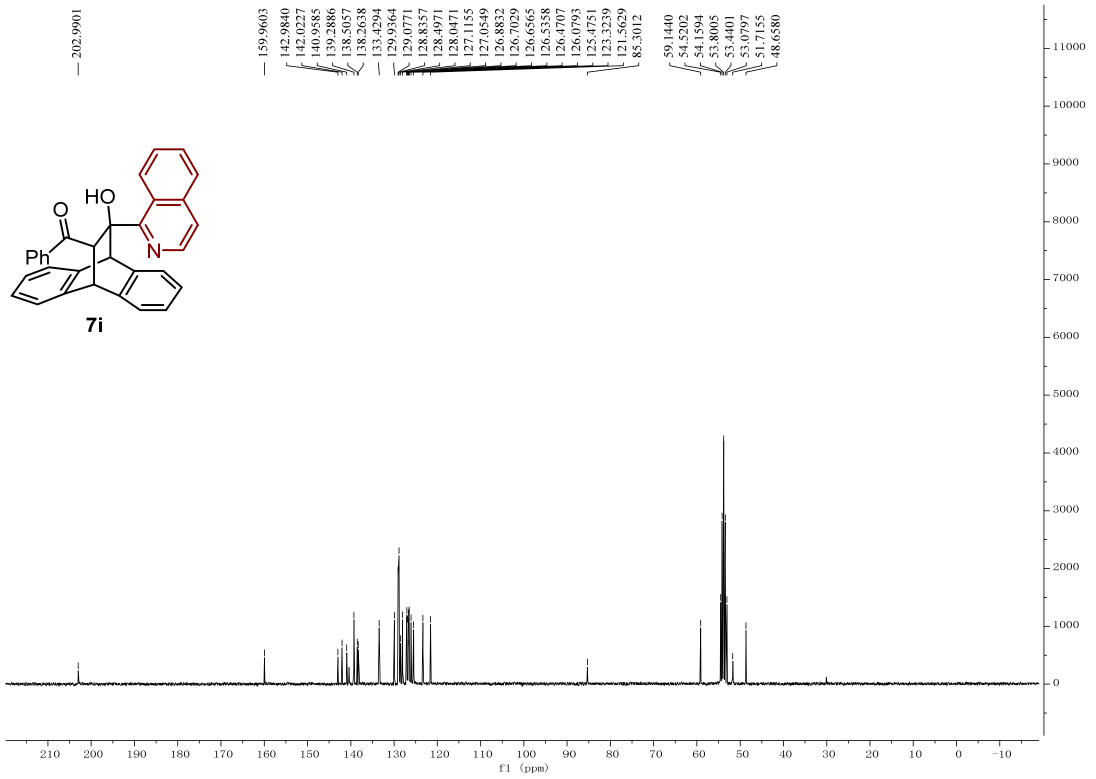

$^{1}$H NMR (300 MHz, Chloroform-$d$) of compound **7j**

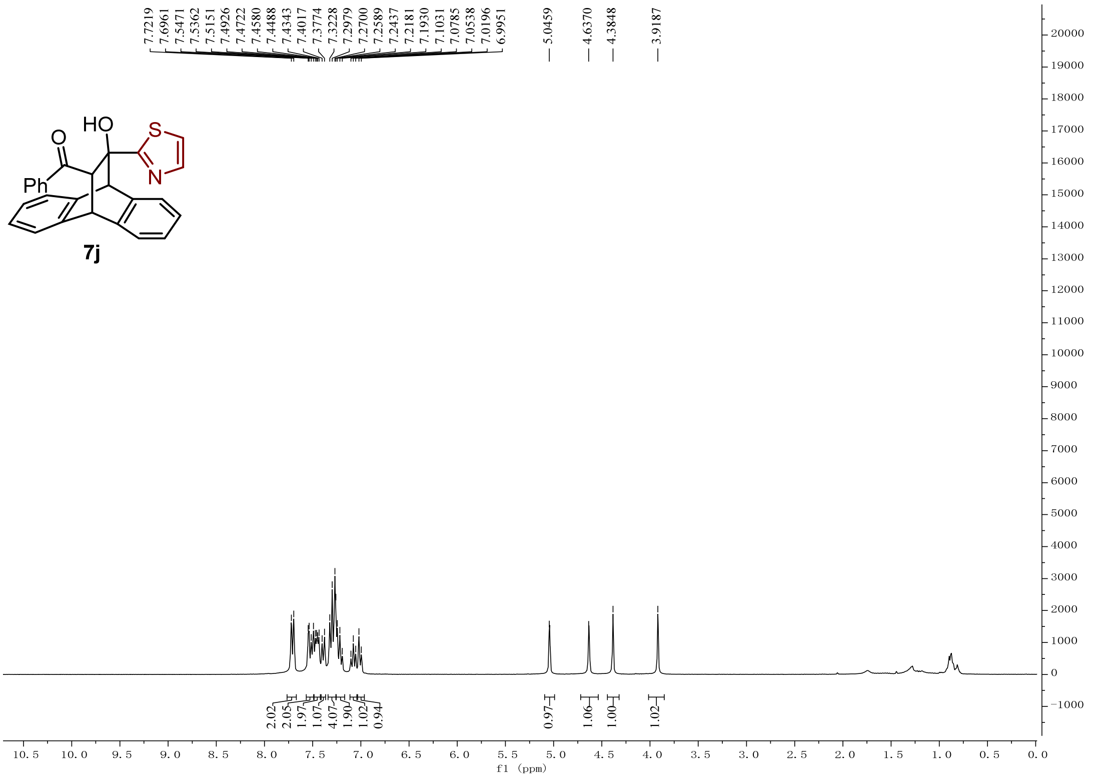

$^{13}$C NMR (75 MHz, Chloroform-$d$) of compound **7j**

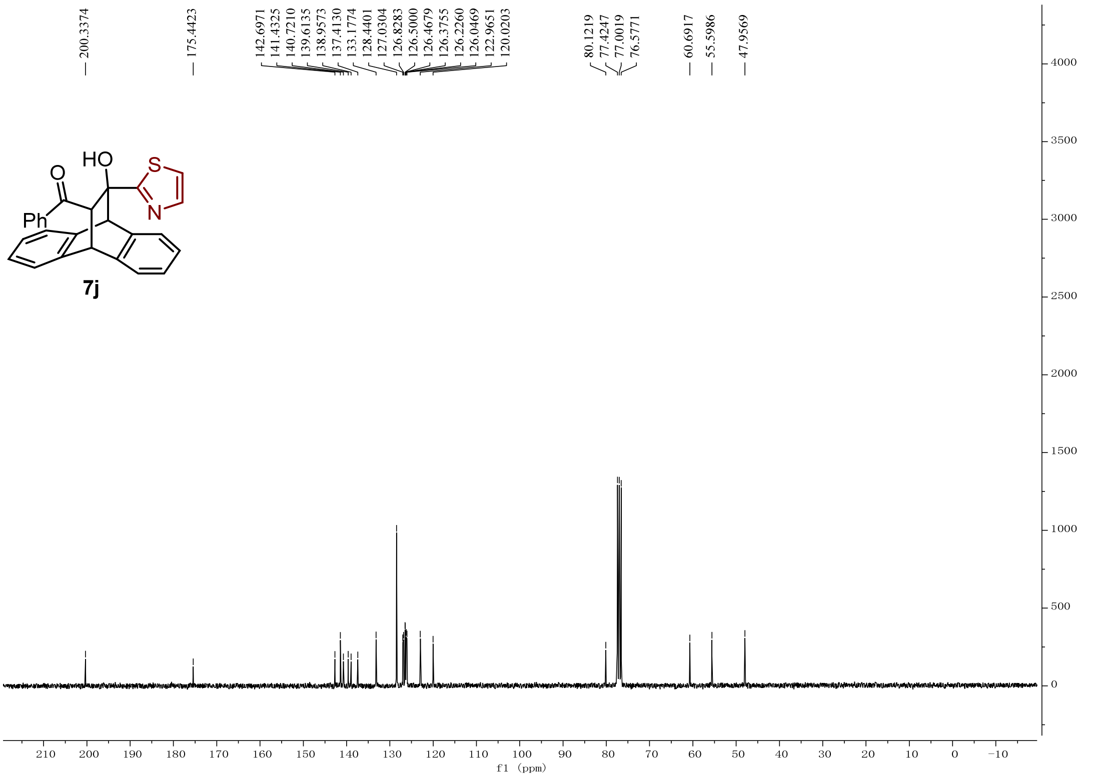

$^{1}$H NMR (300 MHz, Methylene Chloride-$d_2$) of compound **7k**

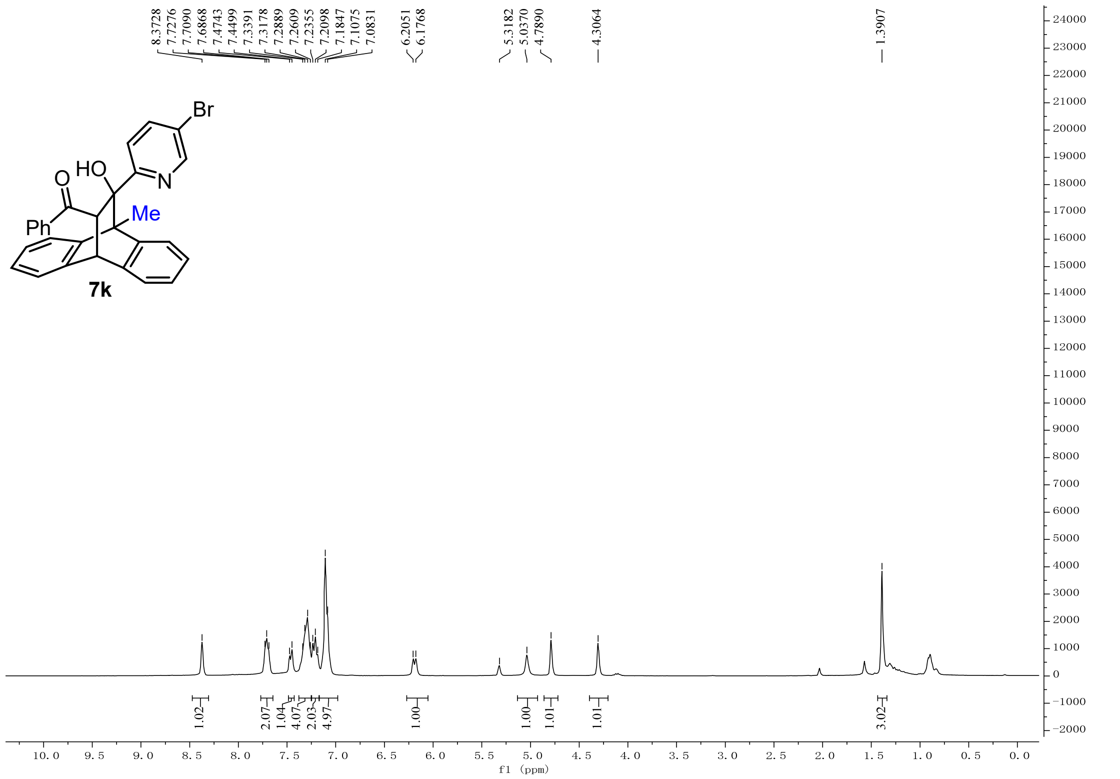


$^{13}$C NMR (75 MHz, Methylene Chloride-$d_2$) of compound **7k**

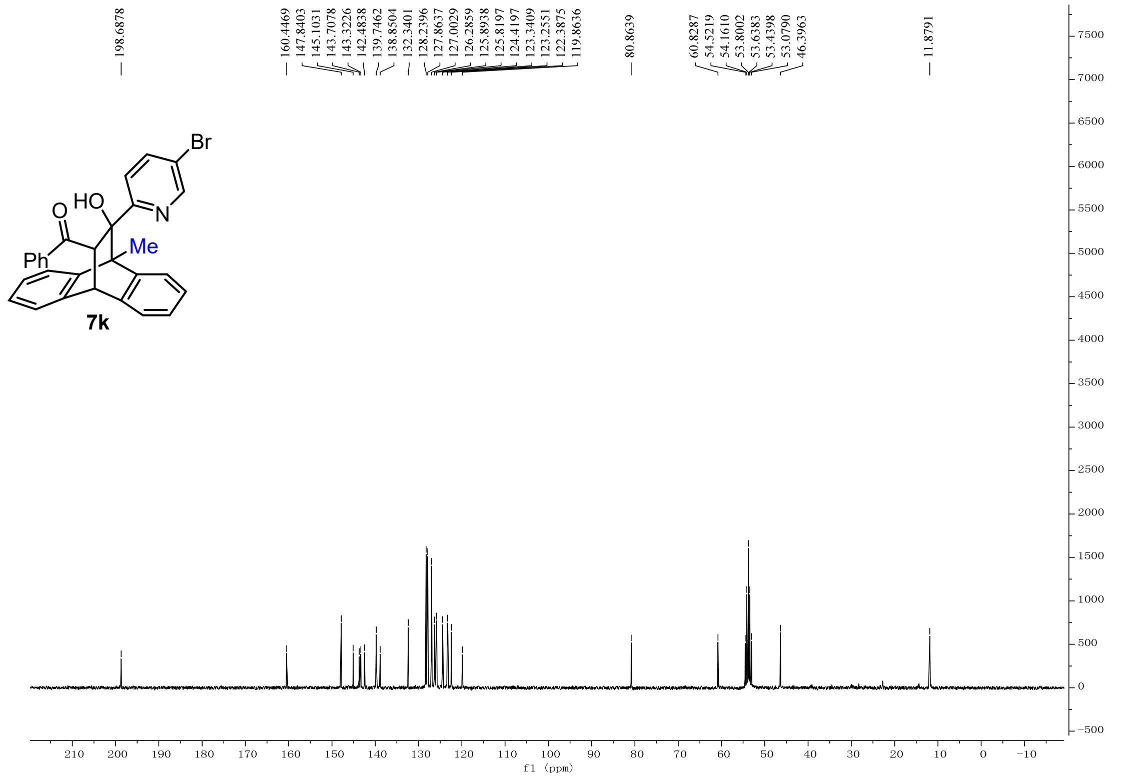

$^{1}$H NMR (300 MHz, Methylene Chloride-$d_2$) of compound **7l**

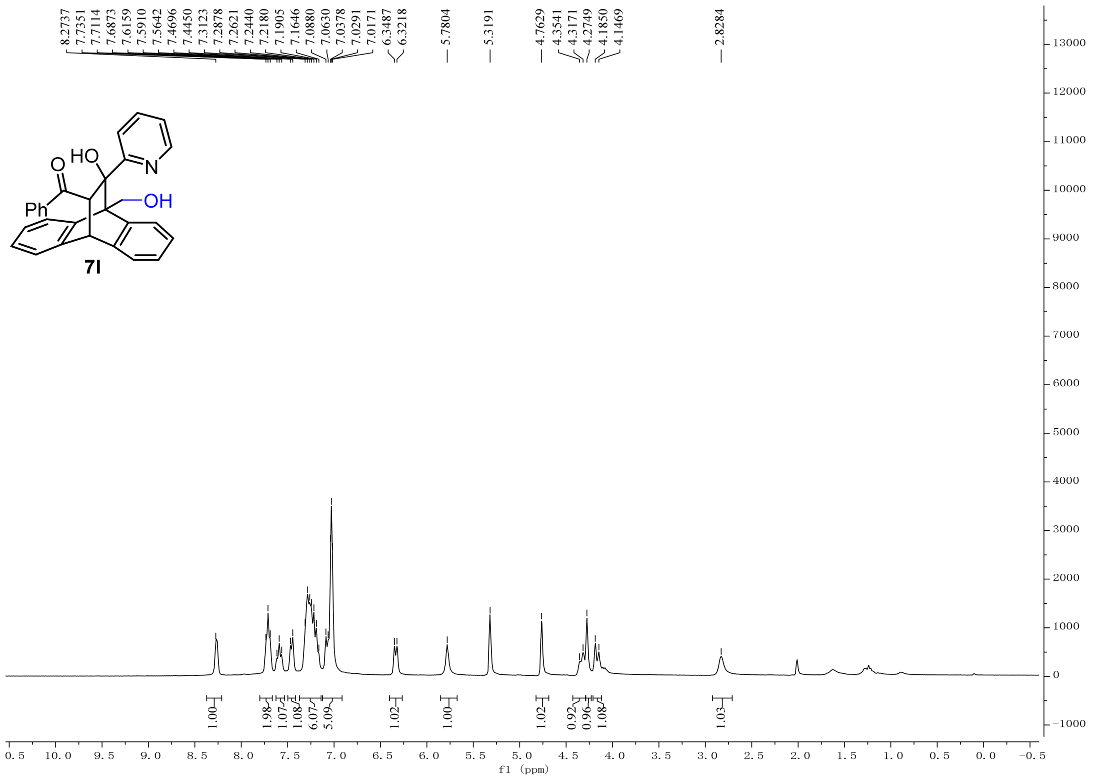

$^{13}$C NMR (75 MHz, Methylene Chloride-$d_2$) of compound **7l**

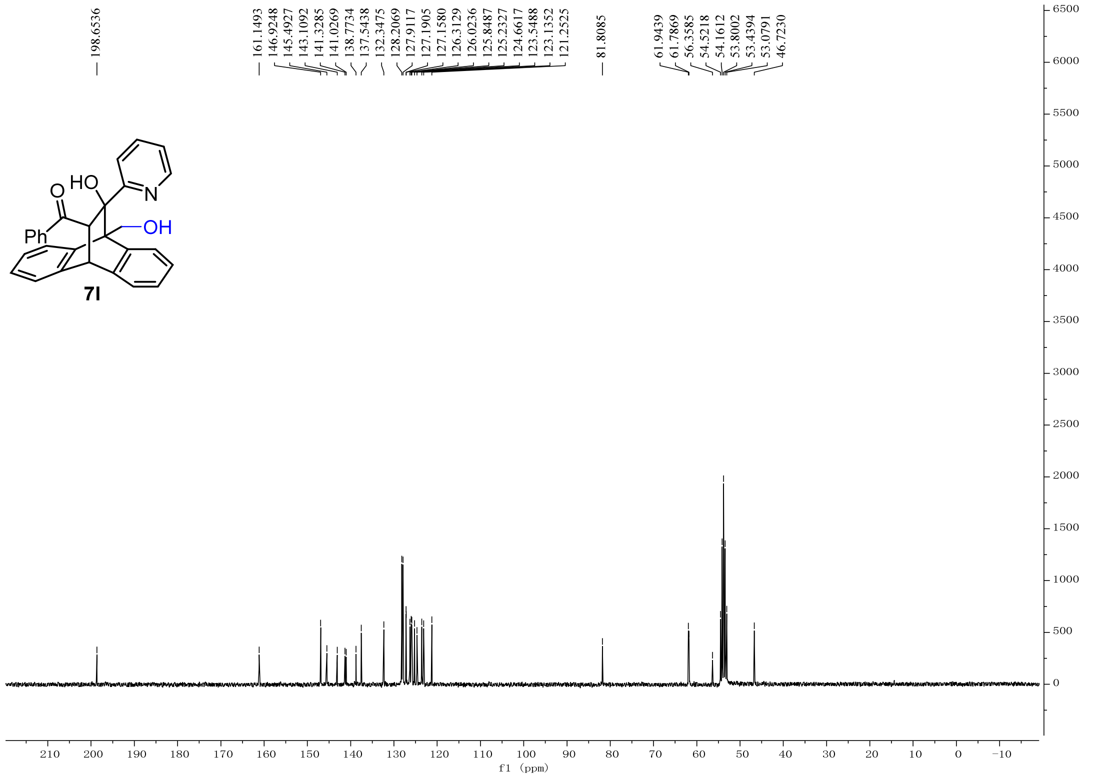

$^1H$ NMR (300 MHz, Chloroform-*d*) of compound **8**

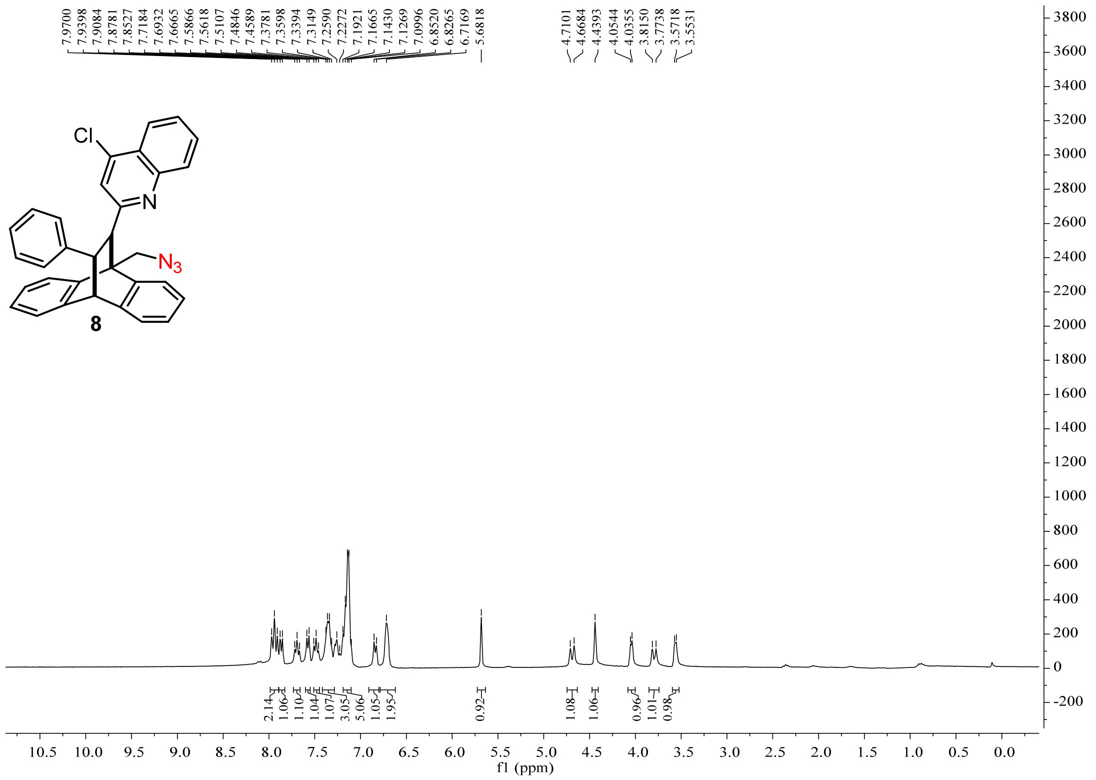

$^{13}C$ NMR (75 MHz, Chloroform-*d*) of compound **8**

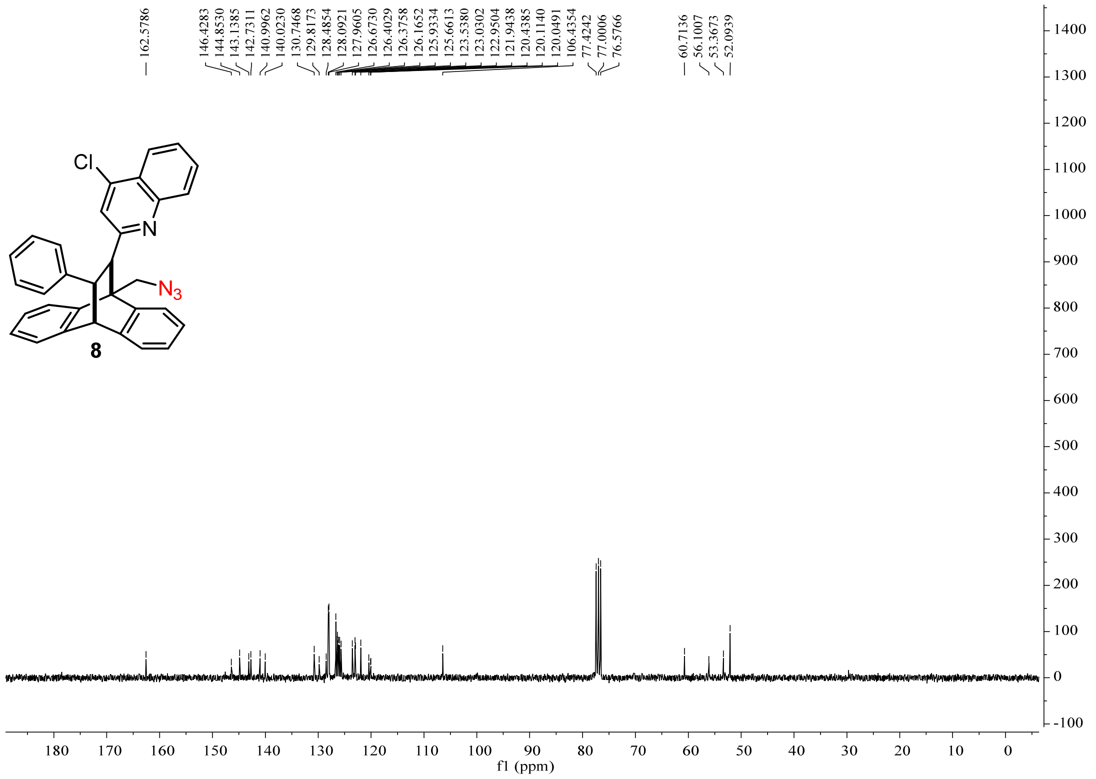

$^{1}$H NMR (300 MHz, Chloroform-*d*) of compound **9**

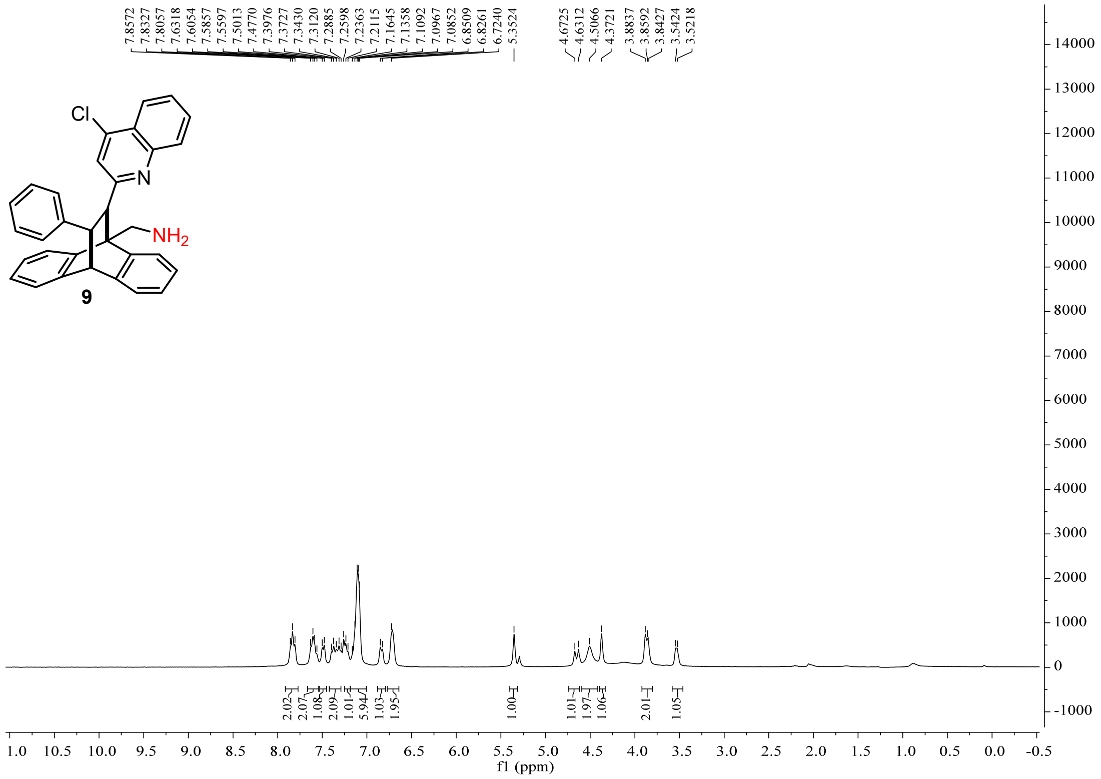


$^{13}$C NMR (75 MHz, Chloroform-*d*) of compound **9**

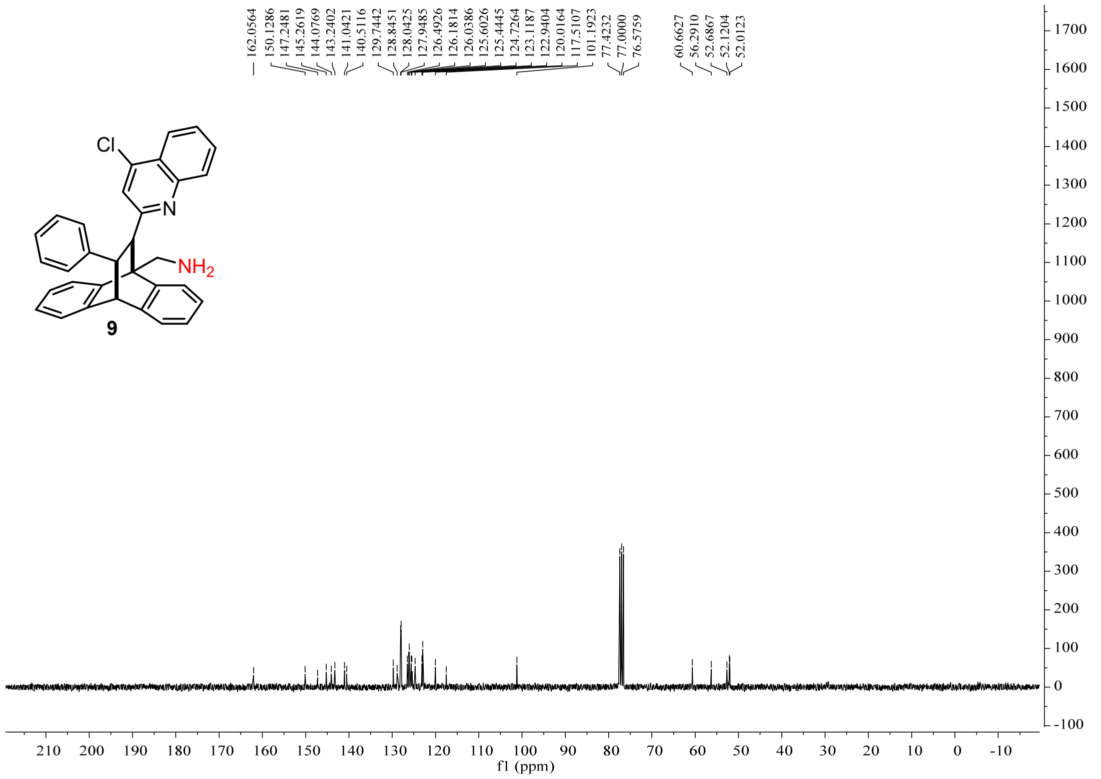

$^{1}$H NMR (300 MHz, Chloroform-*d*) of compound **11**

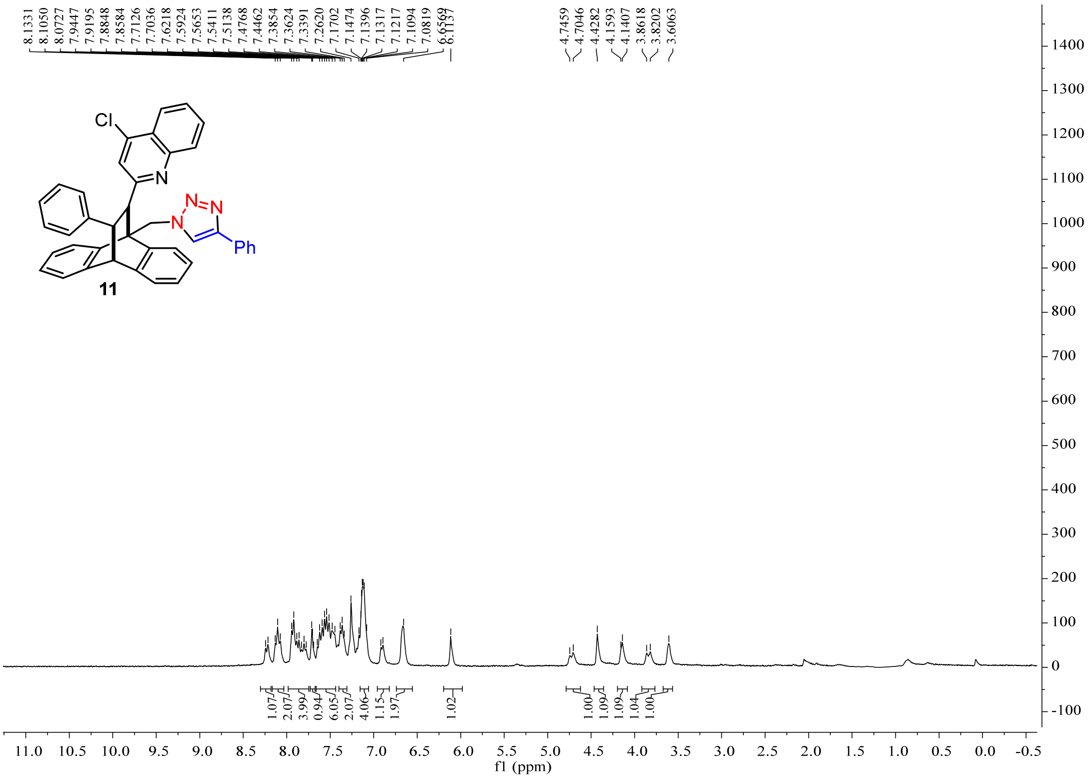

$^{13}$C NMR (75 MHz, Chloroform-*d*) of compound **11**

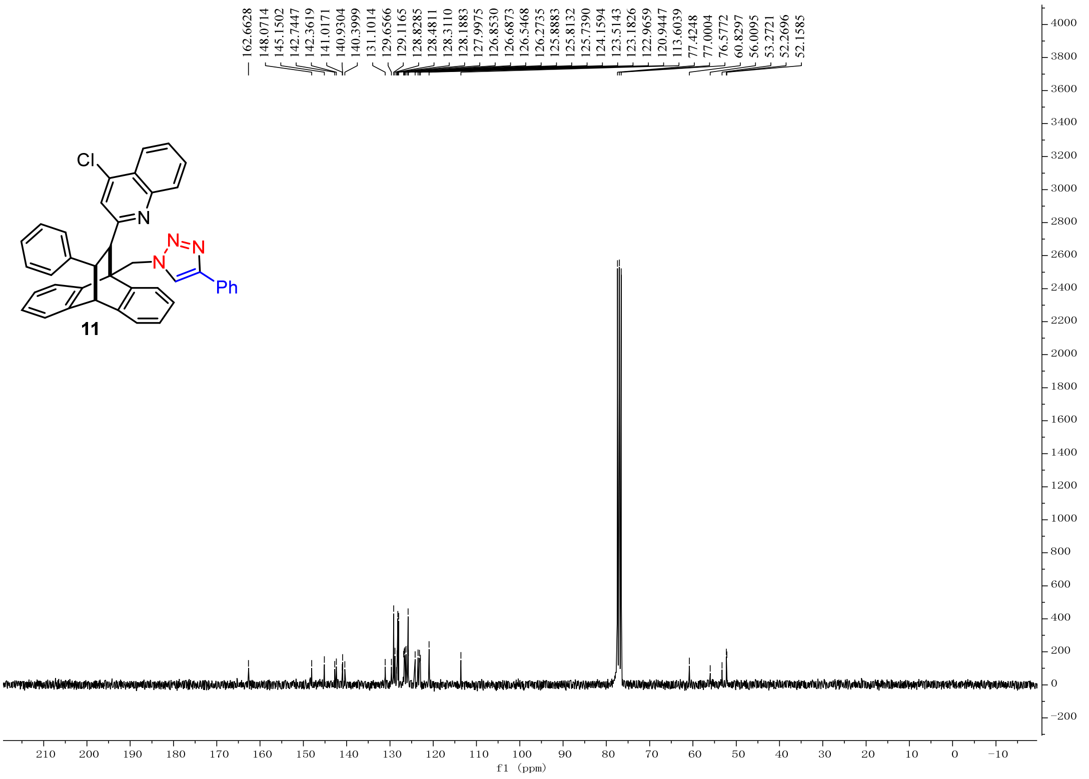

## 9. References


[1] Romero, N. A. & Nicewicz, D. A. *Chem. Rev.* **116**, 10075–10166 (2016).

[2] Ghosh, I., Shaikh, R. S. & König, B. *Angew. Chem. Int. Ed.* **56**, 8544−8549 (2017).
